# 36th International Symposium on Intensive Care and Emergency Medicine: Brussels, Belgium. 15-18 March 2016

**DOI:** 10.1186/s13054-016-1208-6

**Published:** 2016-04-20

**Authors:** R. M. Bateman, M. D. Sharpe, J. E. Jagger, C. G. Ellis, J. Solé-Violán, M. López-Rodríguez, E. Herrera-Ramos, J. Ruíz-Hernández, L. Borderías, J. Horcajada, N. González-Quevedo, O. Rajas, M. Briones, F. Rodríguez de Castro, C. Rodríguez Gallego, F. Esen, G. Orhun, P. Ergin Ozcan, E. Senturk, C. Ugur Yilmaz, N. Orhan, N. Arican, M. Kaya, M. Kucukerden, M. Giris, U. Akcan, S. Bilgic Gazioglu, E. Tuzun, R. Riff, O. Naamani, A. Douvdevani, R. Takegawa, H. Yoshida, T. Hirose, N. Yamamoto, H. Hagiya, M. Ojima, Y. Akeda, O. Tasaki, K. Tomono, T. Shimazu, S. Ono, T. Kubo, S. Suda, T. Ueno, T. Ikeda, T. Hirose, H. Ogura, H. Takahashi, M. Ojima, J. Kang, Y. Nakamura, T. Kojima, T. Shimazu, T. Ikeda, S. Suda, Y. Izutani, T. Ueno, S. Ono, T. Taniguchi, M. O, C. Dinter, J. Lotz, B. Eilers, C. Wissmann, R. Lott, M. M. Meili, P. S. Schuetz, H. Hawa, M. Sharshir, M. Aburageila, N. Salahuddin, V. Chantziara, S. Georgiou, A. Tsimogianni, P. Alexandropoulos, A. Vassi, F. Lagiou, M. Valta, G. Micha, E. Chinou, G. Michaloudis, A. Kodaira, T. Ikeda, S. Ono, T. Ueno, S. Suda, Y. Izutani, H. Imaizumi, M. V. De la Torre-Prados, A. Garcia-De la Torre, A. Enguix-Armada, A. Puerto-Morlan, V. Perez-Valero, A. Garcia-Alcantara, N. Bolton, J. Dudziak, S. Bonney, A. Tridente, P. Nee, G. Nicolaes, M. Wiewel, M. Schultz, K. Wildhagen, J. Horn, R. Schrijver, T. Van der Poll, C. Reutelingsperger, S. Pillai, G. Davies, G. Mills, R. Aubrey, K. Morris, P. Williams, P. Evans, E. G. Gayat, J. Struck, A. Cariou, N. Deye, B. Guidet, S. Jabert, J. Launay, M. Legrand, M. Léone, M. Resche-Rigon, E. Vicaut, A. Vieillard-Baron, A. Mebazaa, R. Arnold, M. Capan, A. Linder, P. Akesson, M. Popescu, D. Tomescu, C. L. Sprung, R. Calderon Morales, G. Munteanu, E. Orenbuch-Harroch, P. Levin, H. Kasdan, A. Reiter, T. Volker, Y. Himmel, Y. Cohen, J. Meissonnier, L. Girard, F. Rebeaud, I. Herrmann, B. Delwarde, E. Peronnet, E. Cerrato, F. Venet, A. Lepape, T. Rimmelé, G. Monneret, J. Textoris, N. Beloborodova, V. Moroz, A. Osipov, A. Bedova, Y. Sarshor, A. Pautova, A. Sergeev, E. Chernevskaya, J. Odermatt, R. Bolliger, L. Hersberger, M. Ottiger, M. Christ-Crain, B. Mueller, P. Schuetz, N. K. Sharma, A. K. Tashima, M. K. Brunialti, F. R. Machado, M. Assuncao, O. Rigato, R. Salomao, S. C. Cajander, G. Rasmussen, E. Tina, B. Söderquist, J. Källman, K. Strålin, A. L. Lange, J. S. Sundén-Cullberg, A. M. Magnuson, O. H. Hultgren, G. Davies, S. Pillai, G. Mills, R. Aubrey, K. Morris, P. Williams, P. Evans, S. Pillai, G. Davies, G. Mills, R. Aubrey, K. Morris, P. Williams, P. Evans, S. Pillai, G. Davies, G. Mills, R. Aubrey, K. Morris, P. Williams, P. Evans, P. Van der Geest, M. Mohseni, J. Linssen, R. De Jonge, S. Duran, J. Groeneveld, R. Miller, B. K. Lopansri, L. C. McHugh, A. Seldon, J. P. Burke, J. Johnston, R. Reece-Anthony, A. Bond, A. Molokhia, C. Mcgrath, E. Nsutebu, P. Bank Pedersen, D. Pilsgaard Henriksen, S. Mikkelsen, A. Touborg Lassen, R. Tincu, C. Cobilinschi, D. Tomescu, Z. Ghiorghiu, R. Macovei, M. A. Wiewel, M. B. Harmon, L. A. Van Vught, B. P. Scicluna, A. J. Hoogendijk, J. Horn, A. H. Zwinderman, O. L. Cremer, M. J. Bonten, M. J. Schultz, T. Van der Poll, N. P. Juffermans, W. J. Wiersinga, G. Eren, Y. Tekdos, M. Dogan, O. Acicbe, E. Kaya, O. Hergunsel, S. Alsolamy, G. Ghamdi, L. Alswaidan, S. Alharbi, F. Alenezi, Y. Arabi, J. Heaton, A. Boyce, L. Nolan, J. Johnston, A. Dukoff-Gordon, A. Dean, A. Molokhia, T. Mann Ben Yehudah, C. Fleischmann, D. Thomas-Rueddel, C. Haas, U. Dennler, K. Reinhart, O. Suntornlohanakul, B. Khwannimit, F. Breckenridge, A. Puxty, P. Szturz, P. Folwarzcny, J. Svancara, R. Kula, P. Sevcik, L. Caneva, A. Casazza, E. Bellazzi, S. Marra, L. Pagani, M. Vetere, R. Vanzino, D. Ciprandi, R. Preda, R. Boschi, L. Carnevale, V. Lopez, M. Aguilar Arzapalo, L. Barradas, A. Escalante, J. Gongora, M. Cetina, B Adamik, D Jakubczyk, A Kübler, A. Radford, T. Lee, J. Singer, J. Boyd, D. Fineberg, M. Williams, J. Russell, E. Scarlatescu, D. Tomescu, G. Droc, S. Arama, M. Müller, M. Straat, S. S. Zeerleder, N. P. Juffermans, C. F. Fuchs, C. S. Scheer, S. W. Wauschkuhn, M. V. Vollmer, K. M. Meissner, S. K. Kuhn, K. H. Hahnenkamp, S. R. Rehberg, M. G. Gründling, N. Yamamoto, M. Ojima, S. Hamaguchi, T. Hirose, Y. Akeda, R. Takegawa, O. Tasaki, T. Shimazu, K. Tomono, E. Gómez-Sánchez, M. Heredia-Rodríguez, E. Álvarez-Fuente, M. Lorenzo-López, E. Gómez-Pesquera, M. Aragón-Camino, P. Liu-Zhu, A. Sánchez-López, A. Hernández-Lozano, M. T. Peláez-Jareño, E. Tamayo, D. O. Thomas-Rüddel, C. Fleischmann, C. Haas, U. Dennler, K. Reinhart, V. Adora, A. Kar, A. Chakraborty, S. Roy, A. Bandyopadhyay, M. Das, T. Mann Ben Yehudah, G. BenYehudah, M. Salim, N. Kumar, L. Arabi, T. Burger, P. Lephart, E. Toth-martin, C. Valencia, N. Hammami, S. Blot, J. L. Vincent, M. L. Lambert, J. Brunke, T. Riemann, I. Roschke, R. Tincu, C. Cobilinschi, D. Tomescu, Z. Ghiorghiu, R. Macovei, S. Nimitvilai, K. Jintanapramote, S. Jarupongprapa, D. Adukauskiene, D. Valanciene, G. Bose, V. Lostarakos, B. Carr, S. Khedher, A. Maaoui, A. Ezzamouri, M. Salem, J. Chen, D. R. Cranendonk, L. A. Van Vught, M. A. Wiewel, O. L. Cremer, J. Horn, M. J. Bonten, M. J. Schultz, T. Van der Poll, W. J. Wiersinga, M. Day, G. Penrice, K. Roy, P. Robertson, G. Godbole, B. Jones, M. Booth, L. Donaldson, Y. Kawano, H. Ishikura, H. Al-Dorzi, M. Almutairi, B. Alhamadi, A. Crizaldo Toledo, R. Khan, B. Al Raiy, Y. Arabi, H. Talaie, J. A. Van Oers, A. Harts, E. Nieuwkoop, P. Vos, Y. Boussarsar, F. Boutouta, S. Kamoun, I. Mezghani, S. Koubaji, A. Ben Souissi, A. Riahi, M. S. Mebazaa, E. Giamarellos-Bourboulis, N. Tziolos, C. Routsi, C. Katsenos, I. Tsangaris, I. Pneumatikos, G. Vlachogiannis, V. Theodorou, A. Prekates, E. Antypa, V. Koulouras, N. Kapravelos, C. Gogos, E. Antoniadou, K. Mandragos, A. Armaganidis, A. R. Robles Caballero, B. Civantos, J. C. Figueira, J. López, A. Silva-Pinto, F. Ceia, A. Sarmento, L. Santos, G. Almekhlafi, Y. Sakr, H. Al-Dorzi, R. Khan, S. Baharoon, A. Aldawood, A. Matroud, J. Alchin, S. Al Johani, H. Balkhy, Y. Arabi, S. Alsolamy, S. Y. Yousif, B. O. Alotabi, A. S. Alsaawi, J. Ang, M. D. Curran, D. Enoch, V. Navapurkar, A. Morris, R. Sharvill, J. Astin, M. Heredia-Rodríguez, E. Gómez-Sánchez, M. T. Peláez-Jareño, E. Gómez-Pesquera, M. Lorenzo-López, P. Liu-Zhu, M. Aragón-Camino, A. Hernández-Lozano, A. Sánchez-López, E. Álvarez-Fuente, E. Tamayo, J. Patel, C. Kruger, J. O’Neal, H. Rhodes, J. Jancik, B. François, P. F. Laterre, P. Eggimann, A. Torres, M. Sánchez, P. F. Dequin, G. L. Bassi, J. Chastre, H. S. Jafri, M. Ben Romdhane, Z. Douira, S. Kamoun, M. Bousselmi, A. Ben Souissi, Y. Boussarsar, A. Riahi, M. S. Mebazaa, A. Vakalos, V. Avramidis, T. H. Craven, G. Wojcik, K. Kefala, J. McCoubrey, J. Reilly, R. Paterson, D. Inverarity, I. Laurenson, T. S. Walsh, S. Mongodi, B. Bouhemad, A. Orlando, A. Stella, G. Via, G. Iotti, A. Braschi, F. Mojoli, M. Haliloglu, B. Bilgili, U. Kasapoglu, I. Sayan, M. Süzer Aslan, A. Yalcın, I. Cinel, A. Vakalos, V. Avramidis, H. E. Ellis, K. Bauchmuller, D. Miller, A. Temple, J. Chastre, B. François, A. Torres, C. E. Luyt, M. Sánchez, M. Singer, H. S. Jafri, Y. Nassar, M. S. Ayad, A. Trifi, S. Abdellatif, F. Daly, R. Nasri, S. Ben Lakhal, B. Bilgili, M. Haliloglu, F. Gul, I. Cinel, A. Kuzovlev, A. Shabanov, S. Polovnikov, V. Moroz, N. Kadrichu, T. Dang, K. Corkery, P. Challoner, G. Li Bassi, E. Aguilera, C. Chiurazzi, C. Travierso, A. Motos, L. Fernandez, R. Amaro, T. Senussi, F. Idone, J. Bobi, M. Rigol, A. Torres, C. J. Hodiamont, N. P. Juffermans, J. M. Janssen, C. S. Bouman, R. A. Mathôt, M. D. De Jong, R. M. Van Hest, L. Payne, G. L. Fraser, B. Tudor, M. Lahner, G. Roth, C. Krenn, H. Talaie, P. Jault, J. Gabard, T. Leclerc, S. Jennes, Y. Que, A. Rousseau, F. Ravat, H. Al-Dorzi, A. Eissa, S. Al-Harbi, T. Aldabbagh, R. Khan, Y. Arabi, A. Trifi, S. Abdellatif., F. Daly, R. Nasri, S. Ben Lakhal, F. Paramba, N. Purayil, V. Naushad, O. Mohammad, V. Negi, P. Chandra, A. Kleinsasser, M. R. Witrz, J. F. Buchner-Doeven, A. M. Tuip-de Boer, J. C. Goslings, N. P. Juffermans, M. Van Hezel, M. Straat, A Boing, R Van Bruggen, N Juffermans, D. Markopoulou, K. Venetsanou, V. Kaldis, D. Koutete, D. Chroni, I. Alamanos, L. Koch, J. Jancik, H. Rhodes, E. Walter, K. Maekawa, M. Hayakawa, S. Kushimoto, A. Shiraishi, H. Kato, J. Sasaki, H. Ogura, T. Matauoka, T. Uejima, N. Morimura, H. Ishikura, A. Hagiwara, M. Takeda, O. Tarabrin, S. Shcherbakow, D. Gavrychenko, G. Mazurenko, V. Ivanova, O. Chystikov, C. Plourde, J. Lessard, J. Chauny, R. Daoust, S. Shcherbakow, O. Tarabrin, D. Gavrychenko, G. Mazurenko, O. Chystikov, A. Vakalos, V. Avramidis, L. Kropman, L. In het Panhuis, J. Konings, D. Huskens, E. Schurgers, M. Roest, B. De Laat, M. Lance, M. Durila, P. Lukas, M. Astraverkhava, J. Jonas, I. Budnik, B. Shenkman, H. Hayami, Y. Koide, T. Goto, R. Iqbal, Y. Alhamdi, N. Venugopal, S. Abrams, C. Downey, C. H. Toh, I. D. Welters, V. B. Bombay, J. M. Chauny, R. D. Daoust, J. L. Lessard, M. M. Marquis, J. P. Paquet, K. Siemens, D. Sangaran, B. J. Hunt, A. Durward, A. Nyman, I. A. Murdoch, S. M. Tibby, F. Ampatzidou, D. Moisidou, E. Dalampini, M. Nastou, E. Vasilarou, V. Kalaizi, H. Chatzikostenoglou, G. Drossos, S. Spadaro, A. Fogagnolo, T. Fiore, A. Schiavi, V. Fontana, F. Taccone, C. Volta, E. Chochliourou, E. Volakli, A. Violaki, E. Samkinidou, G. Evlavis, V. Panagiotidou, M. Sdougka, R. Mothukuri, C. Battle, K. Guy, G. Mills, P. Evans, J. Wijesuriya, S. Keogh, A. Docherty, R. O’Donnell, S. Brunskill, M. Trivella, C. Doree, L. Holst, M. Parker, M. Gregersen, J. Almeida, T. Walsh, S. Stanworth, S. Moravcova, J. Mansell, A. Rogers, R. A. Smith, C. Hamilton-Davies, A. Omar, M. Allam, O. Bilala, A. Kindawi, H. Ewila, F. Ampatzidou, D. Moisidou, M. Nastou, E. Dalampini, A. Malamas, E. Vasilarou, G. Drossos, G. Ferreira, J. Caldas, J. Fukushima, E. A. Osawa, E. Arita, L. Camara, S. Zeferino, J. Jardim, F. Gaioto, L. Dallan, F. B. Jatene, R. Kalil Filho, F. Galas, L. A. Hajjar, C. Mitaka, T. Ohnuma, T. Murayama, F. Kunimoto, M. Nagashima, T. Takei, M. Tomita, A. Omar, K. Mahmoud, S. Hanoura, S. Sudarsanan, P. Sivadasan, H. Othamn, Y. Shouman, R. Singh, A. Al Khulaifi, I. Mandel, S. Mikheev, I. Suhodolo, V. Kiselev, Y. Svirko, Y. Podoksenov, S. A. Jenkins, R. Griffin, M. S. Tovar Doncel, A. Lima, C. Aldecoa, C. Ince, A. Taha, A. Shafie, M. Mostafa, N. Syed, H. Hon, F. Righetti, E. Colombaroli, G. Castellano, F. Righetti, E. Colombaroli, M. Hravnak, L. C. Chen, A. D. Dubrawski, G. C. Clermont, M. R. Pinsky, S. Gonzalez, D. Macias, J. Acosta, P. Jimenez, A. Loza, A. Lesmes, F. Lucena, C. Leon, M. S. Tovar Doncel, C. Ince, C. Aldecoa, A. Lima, M. Bastide, J. Richecoeur, E. Frenoy, C. Lemaire, B. Sauneuf, F. Tamion, S. Nseir, D. Du Cheyron, H. Dupont, J. Maizel, M. Shaban, R. Kolko, N. Salahuddin, M. Sharshir, M. AbuRageila, A. AlHussain, P. Mercado, J. Maizel, L. Kontar, D. Titeca, F. Brazier, A. Riviere, M. Joris, T. Soupison, B. De Cagny, M. Slama, J. Wagner, A. Körner, M. Kubik, S. Kluge, D. Reuter, B. Saugel, E. Colombaroli, F. Righetti, G. Castellano, T. Tran, D. De Bels, A. Cudia, M. Strachinaru, P. Ghottignies, J. Devriendt, C. Pierrakos, Ó. Martínez González, R. Blancas, J. Luján, D. Ballesteros, C. Martínez Díaz, A. Núñez, C. Martín Parra, B. López Matamala, M. Alonso Fernández, M. Chana, W. Huber, M. Eckmann, F. Elkmann, A. Gruber, I. Klein, R. M. Schmid, T. Lahmer, P. W. Moller, S. Sondergaard, S. M. Jakob, J. Takala, D. Berger, D. Bastoni, H. Aya, L. Toscani, L. Pigozzi, A. Rhodes, M. Cecconi, C. Ostrowska, H. Aya, A. Abbas, J. Mellinghoff, C. Ryan, D. Dawson, A. Rhodes, M. Cecconi, M. Cronhjort, O. Wall, E. Nyberg, R. Zeng, C. Svensen, J. Mårtensson, E. Joelsson-Alm, M. Aguilar Arzapalo, L. Barradas, V. Lopez, M. Cetina, N. Parenti, C. Palazzi, L. A. Amidei, F. B. Borrelli, S. C. Campanale, F. T. Tagliazucchi, G. S. Sedoni, D. L. Lucchesi, E. C. Carella, A. L Luciani, M. Mackovic, N. Maric, M. Bakula, H. Aya, A. Rhodes, R. M. Grounds, N. Fletcher, M. Cecconi, B. Avard, P. Zhang, M. Mezidi, J. Charbit, M. Ould-Chikh, P. Deras, C. Maury, O. Martinez, X. Capdevila, P. Hou, W. Z. Linde-Zwirble, I. D. Douglas, N. S. Shapiro, A. Ben Souissi, I. Mezghani, Y. Ben Aicha, S. Kamoun, B. Laribi, B. Jeribi, A. Riahi, M. S. Mebazaa, C. Pereira, R. Marinho, R. Antunes, A. Marinho, M. Crivits, M. Raes, J. Decruyenaere, E. Hoste, V. Bagin, V. Rudnov, A. Savitsky, M. Astafyeva, I. Korobko, V. Vein, T. Kampmeier, P. Arnemann, M. Hessler, A. Wald, K. Bockbreder, A. Morelli, H. Van Aken, S. Rehberg, C. Ertmer, P. Arnemann, M. Hessler, T. Kampmeier, S. Rehberg, H. Van Aken, C. Ince, C. Ertmer, S. Reddy, M. Bailey, R. Beasley, R. Bellomo, D. Mackle, A. Psirides, P. Young, S. Reddy, M. Bailey, R. Beasley, R. Bellomo, D. Mackle, P. Young, H. Venkatesh, S. Ramachandran, A. Basu, H. Nair, S. Egan, J. Bates, S. Oliveira, N. R. Rangel Neto, F. Q. Reis, C. P. Lee, X. L. Lin, C. Choong, K. M. Eu, W. Y. Sim, K. S. Tee, J. Pau, J. Abisheganaden, K. Maas, H. De Geus, E. Lafuente, R. Marinho, J. Moura, R. Antunes, A. Marinho, T. E. Doris, D. Monkhouse, T. Shipley, S. Kardasz, I Gonzalez, S. Stads, A. J. Groeneveld, I. Elsayed, N. Ward, A. Tridente, A. Raithatha, A. Steuber, C. Pelletier, S. Schroeder, E. Michael, T. Slowinski, D. Kindgen-Milles, S. Ghabina, F. Turani, A. Belli, S. Busatti, G. Barettin, F. Candidi, F. Gargano, R. Barchetta, M. Falco, O. Demirkiran, M. Kosuk, S. Bozbay, V. Weber, J. Hartmann, S. Harm, I. Linsberger, T. Eichhorn, G. Valicek, G. Miestinger, C. Hoermann, S. Faenza, D. Ricci, E. Mancini, C. Gemelli, A. Cuoghi, S. Magnani, M. Atti, T. Laddomada, A. Doronzio, B. Balicco, M. C. Gruda, P. O’Sullivan, V. P. Dan, T. Guliashvili, A. Scheirer, T. D. Golobish, V. J. Capponi, P. P. Chan, K. Kogelmann, M. Drüner, D. Jarczak, F. Turani, A. B. Belli, S. M. Martni, V. C. Cotticelli, F. Mounajergi, R. Barchetta, S. Morimoto, H. Ishikura, I. Hussain, N. Salahuddin, A. Nadeem, K. Ghorab, K. Maghrabi, S. K. Kloesel, C. Goldfuss, A. Stieglitz, A. S. Stieglitz, L. Krstevska, G. Albuszies, M. Aguilar Arzapalo, L. Barradas, V. Lopez, A. Escalante, G. Jimmy, M. Cetina, J. Izawa, T. Iwami, S. Uchino, M. Takinami, T. Kitamura, T. Kawamura, J. G. Powell-Tuck, S. Crichton, M. Raimundo, L. Camporota, D. Wyncoll, M. Ostermann, A. Hana, H. R. De Geus, H. R. De Geus, A. Hana, M. Aydogdu, N. Boyaci, S. Yuksel, G. Gursel, A. B. Cayci Sivri, J. Meza-Márquez, J. Nava-López, R. Carrillo-Esper, A. Dardashti, A. Grubb, J. Maizel, M. Wetzstein, D. Titeca, L. Kontar, F. Brazier, B. De Cagny, A. Riviere, T. Soupison, M. Joris, M. Slama, E. Peters, H. Njimi, P. Pickkers, J. L. Vincent, M. Waraich, J. Doyle, T. Samuels, L. Forni, N. Desai, R. Baumber, P. Gunning, A. Sell, S. Lin, H. Torrence, M. O’Dwyer, C. Kirwan, J. Prowle, T. Kim, M. E. O’Connor, R. W. Hewson, C. J. Kirwan, R. M. Pearse, J. Prowle, S. Hanoura, A. Omar, H. Othamn, S. Sudarsanan, M. Allam, M. Maksoud, R. Singh, A. Al Khulaifi, M. E. O’Connor, R. W. Hewson, C. J. Kirwan, R. M. Pearse, J. Prowle, O. Uzundere, D. Memis, M. Ýnal, A. Gultekin, N. Turan, M. A. Aydin, H. Basar, I. Sencan, A. Kapuagasi, M. Ozturk, Z. Uzundurukan, D. Gokmen, A. Ozcan, C. Kaymak, V. A. Artemenko, A. Budnyuk, R. Pugh, S. Bhandari, T. Mauri, C. Turrini, T. Langer, P. Taccone, C. A. Volta, C. Marenghi, L. Gattinoni, A. Pesenti, L. Sweeney, A. O’Sullivan, P. Kelly, E. Mukeria, R. MacLoughlin, M. Pfeffer, J. T. Thomas, G. B. Bregman, G. K. Karp, E. K. Kishinevsky, D. S. Stavi, N. A. Adi, T. Poropat, R. Knafelj, E. Llopart, M. Batlle, C. De Haro, J. Mesquida, A. Artigas, D. Pavlovic, L. Lewerentz, A. Spassov, R. Schneider, S. De Smet, S. De Raedt, E. Derom, P Depuydt, S. Oeyen, D. Benoit, J. Decruyenaere, A. Gobatto, B. Besen, P. Tierno, L. Melro, P. Mendes, F. Cadamuro, M. Park, L. M. Malbouisson, B. C. Civantos, J. L. Lopez, A. Robles, J. Figueira, S. Yus, A. Garcia, A. Oglinda, G. Ciobanu, C. Oglinda, L. Schirca, T. Sertinean, V. Lupu, P. Kelly, A. O’Sullivan, L. Sweeney, R. MacLoughlin, A. O’Sullivan, P. Kelly, L. Sweeney, E. Mukeria, M. Wolny, R. MacLoughlin, A. Pagano, F. Numis, G. Visone, L. Saldamarco, T. Russo, G. Porta, F. Paladino, C. Bell, J. Liu, J. Debacker, C. Lee, E. Tamberg, V. Campbell, S. Mehta, A. Silva-Pinto, A. Sarmento, L. Santos, Ý. Kara, F. Yýldýrým, A. Zerman, Z. Güllü, N. Boyacý, B. Basarýk Aydogan, Ü. Gaygýsýz, K. Gönderen, G. Arýk, M. Turkoglu, M. Aydogdu, G. Aygencel, Z. Ülger, G. Gursel, N. Boyacý, Z. Isýkdogan, Ö. Özdedeoglu, Z. Güllü, M. Badoglu, U. Gaygýsýz, M. Aydogdu, G. Gursel, N. Kongpolprom, C. Sittipunt, A. Eden, Y. Kokhanovsky, S. Bursztein – De Myttenaere, R. Pizov, L. Neilans, N. MacIntyre, M. Radosevich, B. Wanta, V. Weber, T. Meyer, N. Smischney, D. Brown, D. Diedrich, A. Fuller, P. McLindon, K. Sim, M. Shoaeir, K. Noeam, A. Mahrous, R. Matsa, A. Ali, C. Dridi, S. Koubaji, S. Kamoun, F. Haddad, A. Ben Souissi, B. Laribi, A. Riahi, M. S. Mebazaa, A. Pérez-Calatayud, R. Carrillo-Esper, A. Zepeda-Mendoza, M. Diaz-Carrillo, E. Arch-Tirado, S. Carbognin, L. Pelacani, F. Zannoni, A. Agnoli, G. Gagliardi, R. Cho, A. Adams, S. Lunos, S. Ambur, R. Shapiro, M. Prekker, M. Thijssen, L. Janssen, N. Foudraine, C. J. Voscopoulos, J. Freeman, C. J. Voscopoulos, J. Freeman, E. George, C. J. Voscopoulos, D. Eversole, J. Freeman, E. George, S. Muttini, R. Bigi, G. Villani, N. Patroniti, G. Williams, C. J. Voscopoulos, J. Freeman, E George, A. Waldmann, S. Böhm, W. Windisch, S. Strassmann, C. Karagiannidis, A. Waldmann, S. Böhm, W. Windisch, S. Strassmann, C. Karagiannidis, C. K. Karagiannidis, A. W. Waldmann, S. B. Böhm, S. Strassmann, W. W. Windisch, P. Persson, S. Lundin, O. Stenqvist, G. Porta, F. Numis, C. S. Serra, A. P. Pagano, M. M. Masarone, L. R. Rinaldi, A. A. Amelia, M. F. Fascione, L. A. Adinolfi, E. R. Ruggiero, F. Asota, K. O’Rourke, S. Ranjan, P. Morgan, J. W. DeBacker, E. Tamberg, L. O’Neill, L. Munshi, L. Burry, E. Fan, S. Mehta, S. Poo, K. Mahendran, J. Fowles, C. Gerrard, A. Vuylsteke, R. Loveridge, C. Chaddock, S. Patel, V. Kakar, C. Willars, T. Hurst, C. Park, T. Best, A. Vercueil, G. Auzinger, A. Borgman, A. G. Proudfoot, E. Grins, K. E. Emiley, J. Schuitema, S. J. Fitch, G. Marco, J. Sturgill, M. G. Dickinson, M. Strueber, A. Khaghani, P. Wilton, S. M. Jovinge, C. Sampson, S. Harris-Fox, M. E. Cove, L. H. Vu, A. Sen, W. J. Federspiel, J. A. Kellum, C. Mazo Torre, J. Riera, S. Ramirez, B. Borgatta, L. Lagunes, J. Rello, A. K. Kuzovlev, V. Moroz, A. Goloubev, S. Polovnikov, S. Nenchuk, V. Karavana, C. Glynos, A. Asimakos, K. Pappas, C. Vrettou, M. Magkou, E. Ischaki, G. Stathopoulos, S. Zakynthinos, S. Spadaro, I. Kozhevnikova, F. Dalla Corte, S. Grasso, P. Casolari, G. Caramori, C. Volta, T. Andrianjafiarinoa, T. Randriamandrato, T. Rajaonera, S. El-Dash, E. L. V. Costa, M. R. Tucci, F Leleu, L Kontar, B. De Cagny, F. Brazier, D. Titeca, G. Bacari-Risal, J. Maizel, M. Amato, M. Slama, P. Mercado, J. Maizel, L. Kontar, D. Titeca, F. Brazier, A. Riviere, M. Joris, T. Soupison, B. De Cagny, S. El Dash, M. Slama, Remmington, A. Fischer, S. Squire, M. Boichat, H. Honzawa, H. Yasuda, T. Adati, S. Suzaki, M. Horibe, M. Sasaki, M. Sanui, R. Marinho, J. Daniel, H. Miranda, A. Marinho, K. Milinis, M. Cooper, G. R. Williams, E. McCarron, S. Simants, I. Patanwala, I. Welters, Y. Su, J. Fernández Villanueva, R. Fernández Garda, A. López Lago, E. Rodríguez Ruíz, R. Hernández Vaquero, S. Tomé Martínez de Rituerto, E. Varo Pérez, N. Lefel, F. Schaap, D. Bergmans, S. Olde Damink, M. Van de Poll, K. Tizard, C. Lister, L. Poole, D. Ringaitiene, D. Gineityte, V. Vicka, I. Norkiene, J. Sipylaite, A. O’Loughlin, V. Maraj, J. Dowling, M. B. Velasco, D. M. Dalcomune, E. B. Dias, S. L. Fernandes, T. Oshima, S. Graf, C. Heidegger, L. Genton, V. Karsegard, Y. Dupertuis, C. Pichard, N. Friedli, Z. Stanga, B. Mueller, P. Schuetz, L. Vandersteen, B. Stessel, S. Evers, A. Van Assche, L. Jamaer, J. Dubois, R. Marinho, H. Castro, J. Moura, J. Valente, P. Martins, P. Casteloes, C. Magalhaes, S. Cabral, M. Santos, B. Oliveira, A. Salgueiro, A. Marinho, R. Marinho, M. Santos, E. Lafuente, H. Castro, S. Cabral, J. Moura, P. Martins, B. Oliveira, A. Salgueiro, S. Duarte, S. Castro, M. Melo, P. Casteloes, A. Marinho, S. Gray, K. Maipang, R. Bhurayanontachai, L. G. Grädel, P. Schütz, P. Langlois, W. Manzanares, R. Tincu, C. Cobilinschi, D. Tomescu, Z. Ghiorghiu, R. Macovei, W. Manzanares, P. Langlois, M. Lemieux, G. Elke, F. Bloos, K. Reinhart, D. Heyland, P. Langlois, M. Lemieux, I. Aramendi, D. Heyland, W. Manzanares, Y. Su, R. Marinho, N. Babo, A. Marinho, M. Hoshino, Y. Haraguchi, S. Kajiwara, T. Mitsuhashi, T. Tsubata, M. Aida, T. Rattanapraphat, R. Bhurayanontachai, C. Kongkamol, B. Khwannimit, R. Marinho, M. Santos, H. Castro, E. Lafuente, A. Salgueiro, S. Cabral, P. Martins, J. Moura, B. Oliveira, M. Melo, B. Xavier, J. Valente, C. Magalhaes, P. Casteloes, A. Marinho, D. Moisidou, F. Ampatzidou, C. Koutsogiannidis, M. Moschopoulou, G. Drossos, G. Taskin, M. Çakir, AK Güler, A. Taskin, N. Öcal, S. Özer, L. Yamanel, J. M. Wong, C. Fitton, S. Anwar, S. Stacey, M. Aggou, B. Fyntanidou, S. Patsatzakis, E. Oloktsidou, K. Lolakos, E. Papapostolou, V. Grosomanidis, S. Suda, T. Ikeda, S. Ono, T. Ueno, Y. Izutani, S. Gaudry, V. Desailly, P. Pasquier, PB Brun, AT Tesnieres, JD Ricard, D. Dreyfuss, A. Mignon, J. C White, A. Molokhia, A. Dean, A. Stilwell, G. Friedlaender, M. Peters, S. Stipulante, A. Delfosse, AF Donneau, A. Ghuysen, C. Feldmann, D. Freitag, W. Dersch, M. Irqsusi, D. Eschbach, T. Steinfeldt, H. Wulf, T. Wiesmann, N. Kongpolprom, J. Cholkraisuwat, S. Beitland, E. Nakstad, H. Stær-Jensen, T. Drægni, G. Andersen, D. Jacobsen, C. Brunborg, B. Waldum-Grevbo, K. Sunde, K. Hoyland, D. Pandit, K. Hayakawa, E. Oloktsidou, K. Kotzampassi, B. Fyntanidou, S. Patsatzakis, L. Loukipoudi, E. Doumaki, V. Grosomanidis, H. Yasuda, M. M. Admiraal, M. Van Assen, M. J. Van Putten, M. Tjepkema-Cloostermans, A. F. Van Rootselaar, J. Horn, F. Ragusa, A. Marudi, S. Baroni, A. Gaspari, E. Bertellini, A. Taha, T. Abdullah, S. Abdel Monem, S. Alcorn, S. McNeill, S. Russell, W. Eertmans, C. Genbrugge, I. Meex, J. Dens, F. Jans, C. De Deyne, J. Cholkraisuwat, N. Kongpolprom, B Avard, R Burns, A. Patarchi, T. Spina, H. Tanaka, N. Otani, S. Ode, S. Ishimatsu, J. Cho, J. B. Moon, C. W. Park, T. G. Ohk, M. C. Shin, M. H. Won, S. Dakova, Z. Ramsheva, K. Ramshev, J. Cho, J. B. Moon, C. W. Park, T. G. Ohk, M. C. Shin, J. Cho, J. B. Moon, C. W. Park, T. G. Ohk, M. C. Shin, A Marudi, S Baroni, A Gaspari, E Bertellini, G. Orhun, E. Senturk, P. E. Ozcan, S. Sencer, C. Ulusoy, E. Tuzun, F. Esen, R. Tincu, C. Cobilinschi, D. Tomescu, Z. Ghiorghiu, R. Macovei, M. Van Assen, M. M. Admiraal, M. J. Van Putten, M. Tjepkema-Cloostermans, A. F. Van Rootselaar, J. Horn, M. Fallenius, M. B. Skrifvars, M. Reinikainen, S. Bendel, R. Raj, M. Abu-Habsa, C. Hymers, A. Borowska, H. Sivadhas, S. Sahiba, S. Perkins, J. Rubio, J. A. Rubio, R. Sierra, S. English, M. Chasse, A. Turgeon, F. Lauzier, D. Griesdale, A. Garland, D. Fergusson, R. Zarychanski, A. Tinmouth, C. Van Walraven, K. Montroy, J. Ziegler, R. Dupont Chouinard, R. Carignan, A. Dhaliwal, C. Lum, J. Sinclair, G. Pagliarello, L. McIntyre, S. English, M. Chasse, A. Turgeon, F. Lauzier, D. Griesdale, A. Garland, D. Fergusson, R. Zarychanski, A. Tinmouth, C. Van Walraven, K. Montroy, J. Ziegler, R. Dupont Chouinard, R. Carignan, A. Dhaliwal, C. Lum, J. Sinclair, G. Pagliarello, L. McIntyre, T. Groza, N. Moreau, D. Castanares-Zapatero, P. Hantson, M. Carbonara, F. Ortolano, T. Zoerle, S. Magnoni, S. Pifferi, V. Conte, N. Stocchetti, L. Carteron, T. Suys, C. Patet, H. Quintard, M. Oddo, J. A. Rubio, J. Rubio, R. Sierra, V. Spatenkova, E. Pokorna, P. Suchomel, N. Ebert, J. Jancik, H. Rhodes, T. Bylinski, C. Hawthorne, M. Shaw, I. Piper, J. Kinsella, A. K. Kink, I. R. Rätsep, A. Boutin, L. Moore, M. Chasse, R. Zarychanski, F. Lauzier, S. English, L. McIntyre, J. Lacroix, D. Griesdale, P. Lessard-Bonaventure, A. F. Turgeon, A. Boutin, L. Moore, R. Green, P. Lessard-Bonaventure, M. Erdogan, M. Butler, F. Lauzier, M. Chasse, S. English, L. McIntyre, R. Zarychanski, J. Lacroix, D. Griesdale, P. Desjardins, D. A. Fergusson, A. F. Turgeon, B. Goncalves, B. Vidal, C. Valdez, A. C. Rodrigues, L. Miguez, G. Moralez, T. Hong, A. Kutz, P. Hausfater, D. Amin, T. Struja, S. Haubitz, A. Huber, B. Mueller, P. Schuetz, T. Brown, J. Collinson, C. Pritchett, T. Slade, M. Le Guen, S. Hellings, R. Ramsaran, A. Alsheikhly, T. Abe, L. Kanapeckaite, M. Abu-Habsa, R. Bahl, M. Q. Russell, K. J. Real, M. Abu-Habsa, R. M. Lyon, N. P. Oveland, J. Penketh, M. Mcdonald, F. Kelly, M. Alfafi, S. Alsolamy, W. Almutairi, B. Alotaibi, A. E Van den Berg, Y. Schriel, L. Dawson, I. A. Meynaar, H. Talaie, D. Silva, S. Fernandes, J. Gouveia, J. Santos Silva, J. Foley, A. Kaskovagheorgescu, D. Evoy, J. Cronin, J. Ryan, M. Huck, C. Hoffmann, J. Renner, P. Laitselart, N. Donat, A. Cirodde, J. V. Schaal, Y. Masson, A. Nau, T. Leclerc, O. Howarth, K. Davenport, P. Jeanrenaud, S. Raftery, P. MacTavish, H. Devine, J. McPeake, M. Daniel, J. Kinsella, T. Quasim, S. Alrabiee, A. Alrashid, S. Alsolamy, O. Gundogan, C. Bor, E. Akýn Korhan, K. Demirag, M. Uyar, F. Frame, C. Ashton, L. Bergstrom Niska, P. Dilokpattanamongkol, T. Suansanae, C. Suthisisang, S. Morakul, C. Karnjanarachata, V. Tangsujaritvijit, S. Mahmood, H. Al Thani, A. Almenyar, A. Vakalos, V. Avramidis, R. Sharvill, J. Penketh, S. E. Morton, Y. S. Chiew, C. Pretty, J. G. Chase, G. M. Shaw, R. Knafelj, P. Kordis, S. Patel, V. Grover, I. Kuchyn, K. Bielka, Z. Aidoni, V. Grosomanidis, K. Kotzampassi, G. Stavrou, B. Fyntanidou, S. Patsatzakis, C. Skourtis, S. D. Lee, K. Williams, I. D. Weltes, S. Berhane, C. Arrowsmith, C. Peters, S. Robert, J. Caldas, R. B. Panerai, T. G. Robinson, L. Camara, G. Ferreira, E. Borg-Seng-Shu, M. De Lima Oliveira, N. C. Mian, L. Santos, R. Nogueira, S. P. Zeferino, M. Jacobsen Teixeira, F. Galas, L. A. Hajjar, P. Killeen, M. McPhail, W. Bernal, J. Maggs, J. Wendon, T. Hughes, L. U. Taniguchi, E. M. Siqueira, J. M. Vieira Jr, L. C. Azevedo, A. N. Ahmad, M. Abu-Habsa, R. Bahl, E. Helme, S. Hadfield, R. Loveridge, J. Shak, C. Senver, R. Howard-Griffin, P. Wacharasint, P. Fuengfoo, N. Sukcharoen, R. Rangsin, D. Sbiti-Rohr, P. Schuetz, H. Na, S. Song, S. Lee, E. Jeong, K. Lee, M. Cooper, K. Milinis, G. Williams, E. McCarron, S. Simants, I. Patanwala, I. D. Welters, E. Zoumpelouli, E. A Volakli, V. Chrysohoidou, S. Georgiou, K. Charisopoulou, E. Kotzapanagiotou, V. Panagiotidou, K. Manavidou, Z. Stathi, M. Sdougka, N. Salahuddin, B. AlGhamdi, Q. Marashly, K. Zaza, M. Sharshir, M. Khurshid, Z. Ali, M. Malgapo, M. Jamil, A. Shafquat, M. Shoukri, M. Hijazi, T. Abe, S. Uchino, M. Takinami, N. R. Rangel Neto, S. Oliveira, F. Q. Reis, F. A. Rocha, G. Moralez, K. Ebecken, L. S. Rabello, M. F. Lima, R. Hatum, F. V. De Marco, A. Alves, J. E. Pinto, M. Godoy, P. E. Brasil, F. A. Bozza, J. I. Salluh, M. Soares, J. Krinsley, G. Kang, J. Perry, H. Hines, K. M. Wilkinson, C. Tordoff, B. Sloan, M. C. Bellamy, E. Moreira, F. Verga, M. Barbato, G. Burghi, M Soares, U. V. Silva, L. C. Azevedo, A. P. Torelly, J. M. Kahn, D. C. Angus, M. F. Knibel, P. E. Brasil, F. A. Bozza, J. I. Salluh, M. B. Velasco, D. M. Dalcomune, R. Marshall, T. Gilpin, A. Tridente, A. Raithatha, D. Mota, B. Loureiro, J. Dias, O. Afonso, F. Coelho, A. Martins, F. Faria, H. Al-Dorzi, H. Al Orainni, F. AlEid, H. Tlaygeh, A. Itani, A. Hejazi, Y. Arabi, S. Gaudry, J. Messika, J. D. Ricard, S. Guillo, B. Pasquet, E. Dubief, D. Dreyfuss, F. Tubach, C. Battle, K. James, P. Temblett, L. Davies, C. Battle, C. Lynch, S. Pereira, S. Cavaco, J. Fernandes, I. Moreira, E. Almeida, F. Seabra Pereira, M. Malheiro, F. Cardoso, I. Aragão, T. Cardoso, M. Fister, R. Knafelj, P. Muraray Govind, N. Brahmananda Reddy, R. Pratheema, E. D. Arul, J. Devachandran, M. B. Velasco, D. M. Dalcomune, R. Knafelj, M. Fister, N. Chin-Yee, G. D’Egidio, K. Thavorn, D. Heyland, K. Kyeremanteng, A. G. Murchison, K. Swalwell, J. Mandeville, D. Stott, I. Guerreiro, H. Devine, P. MacTavish, J. McPeake, T. Quasim, J. Kinsella, M. Daniel, C. Goossens, M. B. Marques, S. Derde, S. Vander Perre, T. Dufour, S. E. Thiessen, F. Güiza, T. Janssens, G. Hermans, I. Vanhorebeek, K. De Bock, G. Van den Berghe, L. Langouche, H. Devine, P. MacTavish, T. Quasim, J. Kinsella, M. Daniel, J. McPeake, B. Miles, S. Madden, H. Devine, M. Weiler, P. Marques, C. Rodrigues, M. Boeira, K. Brenner, C. Leães, A. Machado, R. Townsend, J. Andrade, P. MacTavish, J. McPeake, H. Devine, J. Kinsella, M. Daniel, R. Kishore, C. Fenlon, T. Quasim, T. Fiks, A. Ruijter, M. Te Raa, P. Spronk, Y. S. Chiew, P. Docherty, J. Dickson, E. Moltchanova, C. Scarrot, C. Pretty, G. M. Shaw, J. G. Chase, T. Hall, W. C. Ngu, J. M. Jack, P. Morgan, B. Avard, A. Pavli, X. Gee, C. Bor, E. Akin Korhan, K. Demirag, M. Uyar, M. Shirazy, A. Fayed, S. Gupta, A. Kaushal, S. Dewan, A. Varma, E. Ghosh, L. Yang, L. Eshelman, B. Lord, E. Carlson, E. Helme, R. Broderick, S. Hadfield, R. Loveridge, J. Ramos, D. Forte, F. Yang, P. Hou, J. Dudziak, J. Feeney, K. Wilkinson, K. Bauchmuller, K. Shuker, M. Faulds, A. Raithatha, D. Bryden, L. England, N. Bolton, A. Tridente, K. Bauchmuller, K Shuker, A Tridente, M Faulds, A Matheson, J. Gaynor, D Bryden, S South Yorkshire Hospitals Research Collaboration, J. Ramos, B. Peroni, R. Daglius-Dias, L. Miranda, C. Cohen, C. Carvalho, I. Velasco, D. Forte, J. M. Kelly, A. Neill, G. Rubenfeld, N. Masson, A. Min, E. Boezeman, J. Hofhuis, A. Hovingh, R. De Vries, P. Spronk, G. Cabral-Campello, I. Aragão, T. Cardoso, M. Van Mol, M. Nijkamp, E. Kompanje, P. Ostrowski, A. Omar, K. Kiss, B. Köves, V. Csernus, Z. Molnár, Y. Hoydonckx, S. Vanwing, B. Stessel, A. Van Assche, L. Jamaer, J. Dubois, V. Medo, R. Galvez, J. P. Miranda, C. Stone, T. Wigmore, Y. Arunan, A. Wheeler, K. Bauchmuller, D. Bryden, Y. Wong, C. Poi, C. Gu, P. Molmy, N. Van Grunderbeeck, O. Nigeon, M. Lemyze, D. Thevenin, J. Mallat, J. Ramos, M. Correa, R. T. Carvalho, D. Forte, A. Fernandez, C. McBride, E. Koonthalloor, C. Walsh, A. Webber, M. Ashe, K. Smith, P. Jeanrenaud, A. Marudi, S. Baroni, F. Ragusa, E. Bertellini, E. A. Volakli, E. Chochliourou, M. Dimitriadou, A. Violaki, P. Mantzafleri, E. Samkinidou, O. Vrani, A. Arbouti, T. Varsami, M. Sdougka, J. A. Bollen, T. C. Van Smaalen, W. C. De Jongh, M. M. Ten Hoopen, D. Ysebaert, L. W. Van Heurn, W. N. Van Mook, K. Sim, A. Fuller, A. Roze des Ordons, P. Couillard, C. Doig, R. V. Van Keer, R. D. Deschepper, A. F. Francke, L. H. Huyghens, J. B. Bilsen, B. Nyamaizi, C. Dalrymple, A. Molokhia, A. Dobru, E. Marrinan, A. Ankuli, A. Molokhia, J. McPeake, R. Struthers, R. Crawford, H. Devine, P. Mactavish, T. Quasim, P. Morelli, M. Degiovanangelo, F. Lemos, V. MArtinez, F. Verga, J. Cabrera, G. Burghi, A. Rutten, S. Van Ieperen, S. De Geer, M. Van Vugt, E. Der Kinderen, A. Giannini, G Miccinesi, T Marchesi, E Prandi

**Affiliations:** 1 University of Western Ontario, London, Canada; 2 Hospital Dr Negrín, Las Palmas de GC, Spain; 3 Hospital San Jorge, Huesca, Spain; 4 Hospital Universitari del Mar, Barcelona, Spain; 5 Hospital Universitario de la Princesa, Madrid, Spain; 6 Hospital Clínico y Universitario de Valencia, Valencia, Spain; 7 Hospital Universitari Son Espases, Palma de Mallorca, Spain; 8 Medical Faculty of Istanbul, Istanbul University, Anesthesiology and Intensive Care, Istanbul, Turkey; 9 Medical Faculty of Istanbul, Physiology, Istanbul University, Istanbul, Turkey; 10 Institute of Experimental Medicine, Istanbul University, Neuroscience, Istanbul, Turkey; 11 Medical Faculty of Istanbul, Forensic Medicine, Istanbul University, Istanbul, Turkey; 12 Institute of Experimental Medicine, Immunology, Istanbul University, Istanbul, Turkey; 13 Ben-Gurion University of the Negev, Beer-Sheva, Israel; 14 Soroka Medical Center, Beer-Sheva, Israel; 15 Osaka University Graduate School of Medicine, Suita, Japan; 16 Nagasaki University Graduate School of Biomedical Sciences, Nagasaki, Japan; 17 Tokyo Medical University Hachioji Medical Center, Hachioji, Tokyo Japan; 18 National Defense Medical College, Tokorozawa, Saitama Japan; 19 Osaka University Graduate School of Medicine, Suita, Japan; 20 Hachiouji medical center, Tokyo medical university, Tokyo, Japan; 21 Kanazawa University, Kanazawa, Japan; 22 Kanazawa University Hospital, Kanazawa, Japan; 23 ThermoFisher, Hennigsdorf, Germany; 24 Institut für Klinische Chemie und Laboratoriumsmedizin, Mainz, Germany; 25 MVZ Labor Limbach Gruppe, Berlin, Germany; 26 Kantonsspital Aarau, Aarau, Switzerland; 27 King Faisal Specialist Hospital & Research Center, Riyadh, Saudi Arabia; 28 Saint Savvas Hospital, Athens, Greece; 29 Tokyo Medical University, Tokyo, Japan; 30 Tokyo Medical University, Hachioji Medical Center, Tokyo, Japan; 31 Hospital Virgen de la Victoria, Málaga, Spain; 32 St Helens and Knowsley NHS Trust, Merseyside, UK; 33 Maastricht University, Maastricht, Netherlands; 34 Center for Experimental and Molecular medicine, Amsterdam, Netherlands; 35 Academic Medical Center, Amsterdam, Netherlands; 36 NISCHR Haemostasis Biomarker Research Unit, Swansea, UK; 37 Hôpital Lariboisière, Paris, France; 38 Sphingotec, Berlin, Germany; 39 Hôpital Cochin, Paris, France; 40 Hôpital Saint-Antoine, Paris, France; 41 CHRU de Montpellier, Montpellier, France; 42 Hôpital Saint-Louis, Paris, France; 43 AP-HM, Marseille, France; 44 Hôpital Ambroise Paré, Paris, France; 45 Christiana Care Health System, Newark, USA; 46 Skane University Hospital, Lund University, Lund, Sweden; 47 Carol Davila University of Medicine and Pharmacy, Bucharest, Romania; 48 Hadassah-Hebrew University Medical Center, Jerusalem, Israel; 49 LeukoDx, Jerusalem, Israel; 50 Abionic SA, Lausanne, Switzerland; 51 Swiss Federal Laboratories (Empa), St. Gallen, Switzerland; 52 Pathophysiology of injury induced immunosuppression (PI3) Lab, Lyon 1 University / Hospices Civils de Lyon / bioMérieux, Lyon, France; 53 Negovsky V.A. Research Institute of General Reanimatology, Moscow, Russia; 54 Kantonsspital Aarau, Aarau, Switzerland; 55 University Hospital Basel, Basel, Switzerland; 56 UNIFESP, Sao Paulo, Brazil; 57 Albert Einstein Hospital, Sao Paulo, Brazil; 58 Sírio Libanês Hospital, Sao Paulo, Brazil; 59 Faculty of Medicine and Health Örebro University, Örebro, Sweden; 60 Department of Infectious Diseases, Karolinska University Hospital, Stockholm, Sweden; 61 Faculty of Medicine and Health, Örebro, Sweden; 62 Center for Infectious Medicine, Karolinska Institutet, Karolinska University Hospital, Stockholm, Sweden; 63 NISCHR Haemostasis Biomarker Research Unit, Swansea, UK; 64 NISCHR Haemostasis Biomarker Research Unit, Swansea, UK; 65 NISCHR Haemostasis Biomarker Research Unit, Swansea, UK; 66 Erasmus Medical Center, Rotterdam, Netherlands; 67 University Witten/Herdecke, Witten, Germany; 68 Maasstad Ziekenhuis, Rotterdam, Netherlands; 69 Intermountain Healthcare, Salt Lake City, USA; 70 Immunexpress, Seattle, USA; 71 Lewisham and Greenwich NHS Trust, London, UK; 72 Wirral trust, Merseyside, UK; 73 RLBUHT, Liverpool, UK; 74 Department of Emergency Medicine, Odense University Hospital, Odense C, Denmark; 75 Department of Respiratory Medicine, Odense University Hospital, Odense C, Denmark; 76 Department of Anaesthesiology and Intensive Care Medicine, Odense University Hospital, Odense C, Denmark; 77 Bucharest Clinical Emergency Hospital, Bucharest, Romania; 78 Fundeni Clinical Institute, Bucharest, Romania; 79 Amsterdam Medical Center, Amsterdam, Netherlands; 80 University Medical Center Utrecht, Utrecht, Netherlands; 81 Bakirkoy Dr.Sadi Konuk Training and Research Hospital, Istanbul, Turkey; 82 King Saud bin Abdulaziz University for Health Sciences and King Abdullah International Medical Research Center, Riyadh, Saudi Arabia; 83 Lewisham and Greenwich NHS Trust, London, UK; 84 Assaf Harofeh MC, Beer Yaakov, Israel; 85 Jena University Hopital, Jena, Germany; 86 Prince of Songkla University, Hat Yai, Thailand; 87 Division of Critical Care Medicine, Hat Yai, Thailand; 88 Glasgow Royal Infirmary, Glasgow, UK; 89 University Hospital and Faculty of Medicine Ostrava University, Ostrava, Czech Republic; 90 Institute of Biostatistics and analyses, Masaryk University, Brno, Czech Republic; 91 Università degli studi di Pavia, scuola di specialità: Anestesia e Rianimazione, Pavia, Italy; 92 UOC Anestesia e Rianimazione Ospedale Civile di Vigevano, AO Pavia, Vigevano, Italy; 93 Università degli studi di Pavia, Pavia, Italy; 94 Hospital O’horan, Mérida, Mexico; 95 Department of Anaesthesiology and Intensive Therapy, Medical University, Wroclaw, Poland; 96 AKPA, Waltham, USA; 97 St. Paul’s Hospital, Vancouver, Canada; 98 Fundeni Clinical Institute, Bucharest, Romania; 99 University of Medicine and Pharmacy “Carol Davila”, Bucharest, Romania; 100 Acedemisch Medisch Centrum, Amsterdam, Netherlands; 101 University Hospital of Greifswald, Greifswald, Germany; 102 Division of Infection Control and Prevention, Osaka University Graduate School of Medicine, Suita, Japan; 103 Department of Traumatology and Acute Critical Medicine, Osaka University Graduate School of Medicine, Suita, Japan; 104 Department of Emergency Medicine, Nagasaki University Graduate School of Biomedical Sciences, Nagasaki, Japan; 105 Hospital Clínico Universitario de Valladolid, Valladolid, Spain; 106 Jena University Hospital, Jena, Germany; 107 Medica Superspecialty Hospital, Kolkata, West Bengal India; 108 Assaf Harofeh MC, Beer Yaakov, Israel; 109 DMC, Detroit, USA; 110 Wayne State University, Detroit, USA; 112 Scientific Institute of Public Health (WIV-ISP), Brussels, Belgium; 113 Ghent University, Ghent, Belgium; 114 Erasme University, Brussels, Belgium; 115 QualityLabs Bt GmbH, Nuremberg, Germany; 116 B.Braun Melsungen AG, Melsungen, Germany; 117 Dr. Roschke medical marketing GmbH, Cologne, Germany; 118 Bucharest Clinical Emergency Hospital, Bucharest, Romania; 119 Fundeni Clinical Institute, Bucharest, Romania; 120 Nakhonpathom hospital, Nakhonpathom, Thailand; 121 Lithuanian University of Health Sciences, Kaunas,, Lithuania; 122 University Hospital North Midlands, Stoke-on-Trent, UK; 123 EPS Charles-Nicolle, Bab Saadoun, Tunisia; 124 Dalin Tzu Chi Hospital, Buddhist Tzu Chi Medical Foundation, Chiayi County, Taiwan; 125 Academic Medical Center, University of Amsterdam, Amsterdam, Netherlands; 126 Academic Medical Center, University of Amsterdam, Center for Experimental and Molecular Medicine, Amsterdam, Netherlands; 127 Department of Intensive Care Medicine, University Medical Center Utrecht, Utrecht, Netherlands; 128 Department of Intensive Care Medicine, Academic Medical Center, University of Amsterdam, Amsterdam, Netherlands; 129 Department of Medical Microbiology, University Medical Center Utrecht, Utrecht, Netherlands; 130 Glasgow Royal Infirmary, Glasgow, UK; 131 NHS Greater Glasgow and Clyde, Glasgow, UK; 132 Health Protection Scotland, Glasgow, UK; 133 Public Health England, London, UK; 134 Fukuoka University Hospital, Fukuoka, Japan; 135 King Saud bin Abdulaziz University for Health Sciences, Riyadh, Saudi Arabia; 136 Toxicological Research Center, Department of Clinical Toxicology, Loghman-Hakim Hospital, Shahid Beheshti University of Medical Sciences, Tehran, Iran; 137 St. Elisabeth Hospital, Tilburg, Netherlands; 138 Mongi Slim University Hospital, La Marsa, Tunisia; 139 University of Athens, Medical School, Athens, Greece; 140 Korgialeneion Benakeion Hospital, Athens, Greece; 141 University of Thrace, Alexandroupolis, Greece; 142 Aghios Dimitrios Hospital, Thessaloniki, Greece; 143 Tzaneion Hospital, Piraeus, Greece; 144 G.Gennimatas General Hospital, Thessaloniki, Greece; 145 University of Ioannina, Ioannina, Greece; 146 G.Papanikolaou General Hospital, Thessaloniki, Greece; 147 University of Patras, Patras, Greece; 148 Hospital Universitario La Paz, Madrid, Spain; 149 Centro Hospitalar São João, Porto, Portugal; 150 PSMMC, Riyadh, Saudi Arabia; 151 UNIKLINIKUM JENA, JENA, Germany; 152 King Saud bin Abdulaziz University for Health Sciences, Riyadh, Saudi Arabia; 153 King Saud bin Abdulaziz University for Health Sciences and King Abdullah International Medical Research Center, Riyadh, Saudi Arabia; 154 King Fahad Medical City, Riyadh, Saudi Arabia; 155 Addenbrooke’s Hospital, Cambridge, UK; 156 University of Cambridge, Cambridge, UK; 157 Royal United Hospital, Bath, UK; 158 Hospital Clínico Universitario de Valladolid, Valladolid, Spain; 159 Salford Royal Hospital, London, UK; 160 Hennepin County Medical Center, Minneapolis, USA; 161 CHU Dupuytren, Limoges, France; 162 St Luc University Hospital, Brussels, Belgium; 163 CHUV, Lausanne, Switzerland; 164 Hospital Clinic of Barcelona, Barcelona, Spain; 165 Hospital Clínico San Carlos, Madrid, Spain; 166 Université François Rabelais and CHU Bretonneau, Tours, France; 167 Groupe Hospitalier Pitié-Salpêtrière, Paris, France; 168 MedImmune, Gaithersburg, USA; 169 Mongi Slim University Hospital, La Marsa, Tunisia; 170 Xanthi General Hospital, Xanthi, Greece; 171 Royal Infirmary of Edinburgh, Edinburgh, UK; 172 Health Protection Scotland, Glasgow, UK; 173 Western General Hospital, Edinburgh, UK; 174 Fondazione IRCCS Policlinico S. Matteo, University of Pavia, Pavia, Italy; 175 Centre Hospitalier Universitaire Dijon, Dijon, France; 176 Marmara University Pendik Teaching and Research Hospital, Istanbul, Turkey; 177 Xanthi General Hospital, Xanthi, Greece; 178 Sheffield Teaching Hospital NHS Foundation Trust, Sheffield, UK; 179 Groupe Hospitalier Pitié-Salpêtrière, Paris, France; 180 CHU Dupuytren, Limoges, France; 181 Hospital Clinic of Barcelona, Barcelona, Spain; 182 Hospital Clínico San Carlos, Madrid, Spain; 183 University College, London, UK; 184 MedImmune, Gaithersburg, USA; 185 Cairo University, Giza, Egypt; 186 University hospital Center of La Rabta, Tunis, Tunisia; 187 Marmara University Pendik Teaching and Research Hospital, Istanbul, Turkey; 188 V.A. Negovsky Research Institute of General Reanimatology, Moscow, Russia; 189 N.V. Sklifosofsky Institute of Emergency Medicine, Moscow, Russia; 190 NN Burdenko Main Military Hospital, Moscow, Russia; 191 Novartis Pharmaceuticals, San Carlos, USA; 192 Nektar Therapeutics, San Francisco, CA USA; 193 Hospital Clinic, Barcelona, Spain; 194 Academic Medical Center, Amsterdam, Netherlands; 195 Maine Medical Center, Portland, USA; 196 Medical University of Vienna, Vienna, Austria; 197 Toxicological Research Center, Department of Clinical Toxicology, Loghman-Hakim Hospital, Shahid Beheshti University of Medical Sciences, Tehran, Iran; 198 HIA Percy, Clamart, France; 199 Pherecydes Pharma, Romainville, France; 200 Hôpital Reine Astrid, Brussels, Belgium; 201 CHUV, Lausanne, Switzerland; 202 CHU Liege, Liege, Belgium; 203 CH Saint Jospeh Saint Luc, Lyon, France; 204 King Saud bin Abdulaziz University for Health Sciences, Riyadh, Saudi Arabia; 205 University hospital center of La Rabta, Tunis, Tunisia; 206 Hamad Medical Corporation, Doha, Qatar; 207 MUI, Innsbruck, Austria; 208 Academic Medical Centre, Amsterdam, Netherlands; 209 Academic Medical Center Amsterdam, Amsterdam, Netherlands; 210 Sanquin, Amsterdam, Netherlands; 211 KAT Hospital Athens, Kifisia, Greece; 212 ICU-B, KAT Hospital Kifisia, Athens, Greece; 213 Hennepin County Medical Center, Minneapolis, USA; 214 Hokkaido University Hospital, Sapporo, Japan; 215 Tohoku University Graduate School of Medicine, Sendai, Japan; 216 Tokyo Medical and Dental University Hospital of Medicine, Tokyo, Japan; 217 National Hospital Organization Disaster Medical Center, Tokyo, Japan; 218 Keio University School of Medicine, Tokyo, Japan; 219 Osaka University Graduate School of Medicine, Osaka, Japan; 220 Rinku General Medical Center, Osaka, Japan; 221 Kinki University Faculty of Medicine, Osaka, Japan; 222 Yokohama City University Graduate School of Medicine, Yokohama, Japan; 223 Faculty of Medicine, Fukuoka University, Fukuoka, Japan; 224 National Center For Global Health and Medicine, Tokyo, Japan; 225 Tokyo Women’s Medical University, Tokyo, Japan; 226 Odessa National Medical University, Odessa, Ukraine; 227 Hopital Sacré-Coeur de Montréal, Montreal, Canada; 228 Odessa National Medical University, Odessa, Ukraine; 229 Xanthi General Hospital, Xanth, Greece; 230 Maastricht UMC, Maastricht, Netherlands; 231 Second Faculty of Medicine, Charles University and University Hospital Motol, Prague, Czech Republic; 232 Sechenov First Moscow Stat Medical University, Moscow, Russia; 233 Sheba Medical Center, Tel-Hashomer, Israel; 234 Yokohama Municipal Citizen’s Hospital, Yokohama, Japan; 235 Hayama Heart Center, Hayama, Japan; 236 Yokohama City University Hospital, Yokohama, Japan; 237 Institute of Ageing and Chronic Disease, Liverpool, UK; 238 Institute of Infection and Global Health, Liverpool, UK; 239 Department of Haematology, Royal Liverpool University Hospital (RLUH), Liverpool, UK; 240 Hôpital du Sacré-Coeur de Montréal, Montreal, Canada; 241 Evelina London Children’s Hospital, London, UK; 242 St Thomas Hospital, London, UK; 243 G.Papanikolaou General Hospital, Thessaloniki, Greece; 244 Intensive Care Unit, University of Ferrara, Italy, Ferrara, Italy; 245 Department of Intensive Care, Erasme Hospital, Université Libre de Bruxelles, Bruxelles, Belgium; 246 Hippokration General Hospital Thessaloniki, Thessaloniki, Greece; 247 Morriston Hospital, Swansea, UK; 248 Central London School of Anaesthesia and Intensive Care Medicine, London, UK; 249 University of the Sunshine Coast, Maroochydore, Australia; 250 University of Edinburgh, Edinburgh, UK; 251 John Radcliffe Hospital, Oxford, UK; 252 Copenhagen University Hospital, Copenhagen, Denmark; 253 Peterborough NHS Trust, Peterborough, UK; 254 Aarhus University, Aarhus, Denmark; 255 Hospital de Sao Paulo, Sao Paulo, Brazil; 256 Royal Brompton & Harefield NHS Trust, London, UK; 257 Barts Heart Centre, London, UK; 258 Hamad medical corporation, Doha, Qatar; 259 G.Papanikolaou General Hospital, Thessaloniki, Greece; 260 University of Sao Paulo, Brazi, Sao Paulo, Brazil; 261 Juntendo University Hospital, Tokyo, Japan; 262 Saitama Medical Center, Jichi Medical University, Saitama, Japan; 263 Gunma University Hospital, Maebashi, Japan; 264 Yokohama City Minato Red Cross Hospital, Yokohama, Japan; 265 Tokyo Medical and Dental University Hospital of Medicine, Tokyo, Japan; 266 Hamad medical corporation, Doha, Qatar; 267 Research Institution for Cardiology, Tomsk, Russia; 268 Siberian State Medical University, Tomsk, Russia; 269 The Hillingdon Hospitals NHS Foundation Trust, Middlesex, UK; 270 University Hospital Rio Hortega, Valladolid, Spain; 271 Erasmus MC, Rotterdam, Netherlands; 272 Sheikh Khalifa Medical City, Abu Dhabi, United Arab Emirates; 273 Intensive Care Unit, St. Boniface Hospital, Verona, Italy; 274 Intensive Care Unit, St. Boniface Hospital, Verona, Italy; 275 University of Pittsburgh, Pittsburgh, USA; 276 Carnegie Mellon University, Pittsburgh, USA; 277 Valme University Hospital, Seville, Spain; 278 University Hospital Rio Hortega, Valladolid, Spain; 279 Erasmus MC, Rotterdam, Netherlands; 280 CHU Amiens, Amiens, France; 281 CH Beauvais, Beauvais, France; 282 Réanimation polyvalente, Le Havre, France; 283 CH Roubaix, Roubaix, France; 284 CH Cotentin, Cherbourg, France; 285 CHU Rouen, Rouen, France; 286 CHU Lille, Lille, France; 287 CHU Caen, Caen, France; 288 King Faisal Specialist Hospital & Research Center, Riyadh, Saudi Arabia; 289 CHU Amiens, Amiens, France; 290 University Medical Center Hamburg-Eppendorf, Hamburg, Germany; 291 Intensive Care Unit, St. Boniface Hospital, Verona, Italy; 292 Brugmann Hospital, Brussels, Belgium; 293 Hospital Universitario del Tajo, Aranjuez, Spain; 294 Hospital Universitario Príncipe de Asturias, Alcalá de Henares, Spain; 295 Hospital Universitario de San Carlos, Madrid, Spain; 296 Klinikum rechts der Isar, Technical University of Munich, Munich, Germany; 297 Department of Intensive Care Medicine, University Hospital Bern, Bern, Switzerland; 298 Institute of Clinical Sciences at the Sahlgrenska Academy, University of Gothenburg, Sahlgrenska University Hospital, Gothenburg, Sweden; 299 St George’s Healthcare NHS Trust, London, UK; 300 St George’s Healthcare NHS Trust, London, UK; 301 Karolinska Institutet, Stockholm, Sweden; 302 Karolinska Institutet, Stockholm, Sweden; 303 Wenzhou Medical University, Wenzhou, Zheijang China; 304 Department of Intensive Care, Austin Hospital, Melbourne, VIC Australia; 305 Hospital O’horan, Mérida, Mexico; 306 Policlinico Modena, Bologna, Italy; 307 Clinical Hospital Sveti Duh, Zagreb, Croatia; 308 St George’s Healthcare NHS Trust, London, UK; 309 The Canberra Hospital, Hughes, ACT Australia; 310 Australian National University Medical School, Canberra, Australia; 311 Lapeyronie University Hospital, Montpellier, France; 312 Brigham and Women’s Hospital, Boston, USA; 313 Trexin Medical, Chicago, USA; 314 University of Colorado and Denver Health, Denver, USA; 315 Beth Isreal Deaconess, Boston, USA; 316 Mongi Slim University Hospital, La Marsa, Tunisia; 317 Centro Hospitalar do Porto, Porto, Portugal; 318 University Hospital, Ghent, Belgium; 319 City Clinical Hospital 40, Yekaterinburg, Russia; 320 University Hospital Muenster, Muenster, Germany; 321 Marienhospital Osnabrück, Osnabrück, Germany; 322 Charité, University of Berlin, Berlin, Germany; 323 Sapienza University of Rome, Rome, Italy; 324 University Hospital of Greifswald, Greifswald, Germany; 325 University Hospital of Muenster, Muenster, Germany; 326 University Hospital of Greifswald, Greifswald, Germany; 327 Erasmus MC University Hospital Rotterdam, Rotterdam, Netherlands; 328 Medical Research Institute of New Zealand, Wellington, New Zealand; 329 Monash University, Melbourne, Australia; 330 Austin Hospital, Melbourne, Australia; 331 Wellington Regional Hospital, Wellington, New Zealand; 332 Medical Research Institute of New Zealand, Wellington, New Zealand; 333 Australian and New Zealand Intensive Care Research Centre, Melbourne, Australia; 334 Austin Hospital, Melbourne, Australia; 335 Princess of Wales Hospital, Bridgend, UK; 336 Imperial College London, London, UK; 337 Glan Clwyd Hospital, Bodelwyddan, UK; 338 University Hospital Galway, Galway, Ireland; 339 Albert Schweitzer State Hospital, Rio de Janeiro, Brazil; 340 Tan Tock Seng Hospital, Singapore, Singapore; 341 National Neuroscience Institute, Singapore, Singapore; 342 Erasmus Medical Centre, Rotterdam, Netherlands; 343 Centro Hospitalar Tamega e Sousa, Penafiel, Portugal; 344 Centro Hospitalar do Porto, Porto, Portugal; 345 Unidade Local de Saude do Alto Minho, Viana do Castelo, Portugal; 346 South Tees NHS Trust, Middlesbrough, UK; 347 Ikazia Hospital, Rotterdam, Netherlands; 348 Erasmus MC, Rotterdam, Netherlands; 349 Sheffiled Teaching Hospitals, Sheffield, UK; 350 Whiston Hospital, St Helens & Knowsley, UK; 351 University Hospital Duesseldorf, Düsseldorf, Germany; 352 Charite University Hospital, Berlin, Germany; 353 Royal London Hospital, London, UK; 354 Aurelia and European Hospital, Rome, Italy; 355 Istanbul University Cerrahpasa Medical School, Istanbul, Turkey; 356 Danube University Krems, Krems, Austria; 357 University Hospital St. Poelten, St. Poelten, Austria; 358 Teaching Hospital Policlinico S.Orsola-Malpighi, Bologna, Italy; 359 Department of Nephrology, Dialysis, Hypertension, Bologna, Italy; 360 Science and Technology Park for Medicine, Mirandola, Italy; 361 Aferetica, Bologna, Italy; 362 San Marco Hospital, Zingonia, Italy; 363 CytoSorbents, Monmouth Junction, USA; 364 Klinikum Emden, Emden, Germany; 365 University Hospital Eppendorf, Hamburg, Germany; 366 Aurelia Hospital /European Hospital, Rome, Italy; 367 Fukuoka University hospital, Fukuoka, Japan; 368 King Faisal Specialist Hospital and Research Center, Riyadh, Saudi Arabia; 369 GPR Klinikum Ruesselsheim, Ruesselsheim, Hessen Germany; 370 Hospital O’horan, Mérida, Mexico; 371 Jikei University School of Medicine, Tokyo, Japan; 372 Kyoto University, Kyoto, Japan; 373 Osaka University, Osaka, Japan; 374 Guy’s & St Thomas’ NHS Foundation Trust, London, UK; 375 King’s College, London, UK; 376 Hospital de Santa Maria, Lisbon, Portugal; 377 ErasmusMC, Rotterdam, Netherlands; 378 ErasmusMC, Rotterdam, Netherlands; 379 Gazi University Medical Faculty, Ankara, Turkey; 380 Gazi University School of Medicine, Critical Care Fellowship Programme, Ankara, Turkey; 381 Gazi University School of Medicine Biochemistry Department, Ankara, Turkey; 382 Fundación Clínica Medica Sur, Mexico City, Mexico; 383 Lund University and Skane University Hospital, Lund, Sweden; 384 CHU Amiens, Amiens, France; 385 Radboudumc, Nijmegen, Netherlands; 386 Erasme Hospital, Université Libre de Bruxelles, Brussels, Belgium; 387 The Royal Surrey County Hospital, Guildford, UK; 388 Royal National Orthopaedic Hospital, Middlesex, UK; 389 Barts Health NHS Trust, London, UK; 390 Queen Mary University of London, London, UK; 391 Asan medical center, Seoul, South Korea; 392 Barts Health NHS Trust, London, UK; 393 Queen Mary University of London, London, UK; 394 Hamad medical corporation, Doha, Qatar; 395 Barts Health NHS Trust, London, UK; 396 Queen Mary University of London, London, UK; 397 Trakya Univ, Edirne, Turkey; 398 General Directorate of Health Services, Ministry of Health, Ankara, Turkey; 399 AnkaraNKARA Research and Training Hospital, Ankara, Turkey; 400 Department of Biostatistics, Faculty of Medicine, Ankara University, Ankara, Turkey; 401 Ankara Research and Training Hospital, Ankara, Turkey; 402 MC Into-Sana, Odessa, Ukraine; 403 Glan Clwyd Hospital, Rhyl, UK; 404 Fondazione IRCCS Ca’ Granda, Maggiore Policlinico Hospital, Milan, Italy; 405 University of Ferrara, Sant’Anna Hospital, Ferrara, Italy; 406 Aerogen, Galway, Ireland; 407 Kaplan Medical Centre, Rehovot, Israel; 408 West Virginia University, Morgantown, West Virginia USA; 409 Rihard Knafelj, Ljubljana, Slovenia; 410 Corporació Sanitària i Universitària Parc Taulí, Universitat Autònoma de Barcelona, CIBER Enfermedades Respiratorias, Sabadell, Barcelona, Spain; 411 Dalhousie University, Halifax, Canada; 412 Ernst-Moritz-Arndt-Universität, Greifswald, Germany; 413 Ghent University Hospital, Gent, Belgium; 414 Universidade de São Paulo, São Paulo, Brazil; 415 Hospital La Paz, Madrid, Spain; 416 Institute of Mother and Child, Chisinau mun., Moldova; 417 Institute of Emergency Medicine, Chisinau mun., Moldova; 418 State University of Medicine and Pharmacy, Chisinau mun., Moldova; 419 Aerogen, Galway, Ireland; 420 Aerogen, Galway, Ireland; 421 Cardarelli Hospital, Naples, Italy; 422 San Paolo Hospital, Naples, Italy; 423 Cardarelli Hospital, Naples, Italy; 424 National University of Ireland, Galway, Galway City, Ireland; 425 Mount Sinai Hospital, Toronto, Canada; 426 Centro Hospitalar São João, Porto, Portugal; 427 Gazi University School of Medicine Respiratory Medicine and Critical Care Department, Ankara, Turkey; 428 Gazi University School of Medicine, Internal Medicine Critical Care Department, Ankara, Turkey; 429 Gazi University School of Medicine, Geriatrics Department, Ankara, Turkey; 430 Gazi University School of Medicine Respiratory Medicine and Critical Care Department, Ankara, Turkey; 431 Chulalongkorn University, Bangkok, Thailand; 432 Carmel, Lady Davis Medical Center, Haifa, Israel; 433 Duke University, Durham, USA; 434 Mayo Clinic, Rochester, USA; 435 St Helens and Knoowsley, Prescot, UK; 436 Royal Infirmary, Edinburgh, UK; 437 Alexandria Universitry General Hospital, Alexandria, Egypt; 438 University Hospital North Middlands, Stoke On Trent, UK; 439 Mongi Slim University Hospital, La Marsa, Tunisia; 440 Medica Sur, Mexico City, Mexico; 441 Instituto Nacional de Rehabilitacion, Mexico City, Mexico; 442 University of Padua, Padua, Italy; 443 Sant Antony hospital, Padua, Italy; 444 HCMC, Minneapolis, USA; 445 University of Minnesota, Minneapolis, USA; 446 Viecuri Medical Center, Venlo, Netherlands; 447 University of Hawaii, John A. Burns School of Medicine, Honolulu, USA; 448 Respiratory Motion Inc., Waltham, USA; 449 University of Hawaii, John A. Burns School of Medicine, Honolulu, USA; 450 Respiratory Motion Inc., Waltham, USA; 451 Massachusetts General Hospital, Boston, USA; 452 University of Hawaii, John A. Burns School of Medicine, Honolulu, USA; 453 Respiratory Motion Inc., Waltham, USA; 454 Massachusetts General Hospital, Boston, USA; 455 AO Desio e Vimercate, Vimercate, Italy; 456 AO Crema, Crema, Italy; 457 University of Milan-Bicocca, Monza, Italy; 458 UT Health Science Center at Houston, Houston, TX USA; 459 University of Hawaii, John A. Burns School of Medicine, Honolulu, HI USA; 460 Respiratory Motion Inc., Waltham, MA USA; 461 Massachusetts General Hospital, Boston, MA USA; 462 Swisstom AG, Landquart, Switzerland; 463 Kliniken der Stadt Köln, Pneumology and Critical Care Medicine, Witten / Herdecke University, Ostmerheimer Str. 200, 51109 Cologne, Germany; 464 Swisstom AG, Landquart, Switzerland; 465 Kliniken der Stadt Köln, Pneumology and Critical Care Medicine, Witten/ Herdecke University, Ostmerheimer Str. 200, 51109 Cologne, Germany; 466 ARDS and ECMO Center Köln-Merheim, Cologne, Germany; 467 Swisstom, Chur, Switzerland; 468 Sahlgrenska Univ Hospital, Göteborg, Sweden; 469 Second University of Naples, Naples, Italy; 470 San Paolo Hospital, Naples, Italy; 471 University of Sassari, Sassari, Italy; 472 Cardarelli Hospital, Naples, Italy; 473 University of Saerno, Salerno, Italy; 474 East Surrey Hospital, Surrey and Sussex NHS Trust, Surrey, UK; 475 Mount Sinai Hospital and University of Toronto, Toronto, Canada; 476 University Health Network-Toronto General Hospital and Univeristy of Toronto, Toronto, Canada; 477 University of Cambridge, Cambridge, UK; 478 Papworth Hospital, Cambridge, UK; 479 King’s College Hospital NHS Foundation Trust, London, UK; 480 Meijer Heart & Vascular Institute, Grand Rapids, USA; 481 Glenfield Hospital, UHL, Leicester, UK; 482 National University Hospital, Singapore, Singapore; 483 National University Singapore, Singapore, Singapore; 484 Mayo Clinic, Phoenix, AZ USA; 485 University of Pittsburgh, Pittsburgh, PA USA; 486 Vall D’Hebron University Hospital, Barcelona, Spain; 487 Universitat Autonoma de Barcelona, Barcelona, Spain; 488 V.A. Negovsky Research Institute of General Reanimatology, Moscow, Russia; 489 NN Burdenko Main Military Hospital, Moscow, Russia; 490 George P. Livanos and Marianthi Simou Laboratories, Athens, Greece; 491 University of Patras, Rio, Achaia Greece; 492 University of Ferrara, Ferrara, Italy; 493 University of Bari, Bari, Italy; 494 CHU HJRA, Antananarivo, Madagascar; 495 Réanimation médicale, Centre Hospitalier Universitaire, Amiens, France; 496 Cardio-Pulmonary Department, Pulmonary Division, Heart Institute (Incor), University of São Paulo, São Paulo, Brazil; 497 CHU Amiens, Amiens, France; 498 Royal Brompton Hospital, London, UK; 499 Musashino Red Cross Hospital, Tokyo, Japan; 500 JSEPTIC Clinical Trial Group, Tokyo, Japan; 501 Centro Hospitalar do Porto, Porto, Portugal; 502 University of Liverpool, Liverpool, UK; 503 Royal Liverpool University Hospital, Liverpool, UK; 504 Dalin Tzu Chi Hospital, Buddhist Tzu Chi Medical Foundation, Chiayi County, Taiwan; 505 Hospital Clínico Universitario de Santiago de Compostela, Santiago de Compostela, Spain; 506 MUMC, Maastricht, Netherlands; 507 Royal Liverpool University Hospital, Liverpool, UK; 508 Vilnius University Hospital Santariskiu Clinics, Vilnius, Lithuania; 509 Vilnius University, Faculty of Medicine, Vilnius, Lithuania; 510 Mercy University Hospital, Cork, Ireland; 511 Hospital Meridional S.A., Cariacica, Brazil; 512 Geneva Universtiy Hospital, Geneva, Switzerland; 513 Medical University Department, Kantonsspital Aarau, Aarau, Switzerland; 514 Department of Endocrinology, Diabetes and Clinical Nutrition, University Hospital Bern, Bern, Switzerland; 515 Jessa Ziekenhuis, Hasselt, Belgium; 516 Centro Hospitalar do Porto, Porto, Portugal; 517 Unidade Local de Saude do Alto Minho, Viana do Castelo, Portugal; 518 Unidade de Saude Local de Castelo Branco, Castelo Branco, Portugal; 519 Centro Hospitalar e Universitario de Coimbra, Coimbra, Portugal; 520 Centro Hospitalar de Vila Nova de Gaia, Vila Nova de Gaia, Portugal; 521 Centro Hospitalar do Algarve, Faro, Portugal; 522 Instituto Portugues de Oncologia do Porto, Porto, Portugal; 523 Faculdade de Ciencias da Nutricao e Alimentacao da Universidade do Porto, Porto, Portugal; 524 Centro Hospitalar do Porto, Porto, Portugal; 525 Faculdade de Ciencias da Nutrição e Alimentação da Universidade do Porto, Porto, Portugal; 526 Centro Hospitalar Tamega e Sousa, Penafiel, Portugal; 527 Instituto Português de Oncologia do Porto, Porto, Portugal; 528 Unidade Local de Saude do Alto Minho, Viana do Castelo, Portugal; 529 Centro Hospitalar e Universitário de Coimbra, Coimbra, Portugal; 530 Unidade de Saude Local de Castelo Branco, Castelo Branco, Portugal; 531 Centro Hospitalar do Algarve, Faro, Portugal; 532 Centro Hospitalar do Baixo Vouga, Aveiro, Portugal; 533 Centro Hospitalar de Vila Nova de Gaia, Vila Nova de Gaia, Portugal; 534 Nottingham University Hospital NHS Trust, Nottingham, UK; 535 Prince of Songkla University, Songkla, Thailand; 536 Kantonsspital Aarau, Aarau, Switzerland; 537 Université de Sherbrooke, Sherbrooke, Canada; 538 University Hospital, Montevideo, Uruguay; 539 Bucharest Clinical Emergency Hospital, Bucharest, Romania; 540 Fundeni Clinical Institute, Bucharest, Romania; 541 University Hospital, Montevideo, Uruguay; 542 Université de Sherbrooke, Sherbrooke, Canada; 543 Queen’s University, Kingston, Canada; 544 University Medical Center Schleswig-Holstein, Kiel, Germany; 545 University of Jena, Jena, Germany; 546 Université de Sherbrooke, Sherbrooke, Canada; 547 Queen’s University, Kingston, Canada; 548 University Hospital, Montevideo, Uruguay; 549 Dalin Tzu Chi Hospital, Buddhist Tzu Chi Medical Foundation, Chiayi County, Taiwan; 550 Centro Hospitalar do Porto, Porto, Portugal; 551 Aida Hospital, Fukushima, Japan; 552 Keiyo Hospital, Tokyo, Japan; 553 Shisei Hospital, Saitama, Japan; 554 Prince of Songkla University, Hat Yai City, Songkhla Province Thailand; 555 Centro Hospitalar do Porto, Porto, Portugal; 556 Faculdade de Ciencias da Nutrição e Alimentação da Universidade do Porto, Porto, Portugal; 557 Centro Hospitalar Tamega e Sousa, Penafiel, Portugal; 558 Centro Hospitalar e Universitário de Coimbra, Coimbra, Portugal; 559 Instituto Português de Oncologia do Porto, Porto, Portugal; 560 Unidade Local de Saude do Alto Minho, Viana do Castelo, Portugal; 561 Centro Hospitalar Baixo Vouga, Aveiro, Portugal; 562 Unidade de Saude Local de Castelo Branco, Castelo Branco, Portugal; 563 Centro Hospitalar do Algarve, Faro, Portugal; 564 Centro Hospitalar de Vila Nova de Gaia, Vila Nova de Gaia, Portugal; 565 Department of Cardiothoracic Surgery, George Papanikolaou General Hospital, Thessaloniki, Greece; 566 Gülhane Military Medical Academy, Ankara, Turkey; 567 Ankara Mevki Military Hospital, Ankara, Turkey; 568 St Bartholomew’s Hospital, London, UK; 569 Aristotle Medical School, Thessaloniki, Greece; 570 Tokyo Medical University Hachiosi Medical Center, Tokyo, Japan; 571 Hôpital Louis Mourier, Colombes, France; 572 Laboratoire ILUMENS, Paris, France; 573 Institut de Hauts de Seine, Nanterre, France; 574 University Hospital Lewisham, London, UK; 575 Queen Elizabeth Hospital, London, UK; 576 Minet Green Health Practice, London, UK; 577 Department of Public Health, University of Liège, Liège, Belgium; 578 Department of Emergency Medicine, University Hospital of Liège, Liège, Belgium; 579 Department of Medical Biostatistics, University of Liège, Liège, Belgium; 580 University Hospital, Philipps University Marburg, Marburg, Germany; 581 Chulalongkorn University, Bangkok, Thailand; 582 Oslo University Hospital, Oslo, Norway; 583 William Harvey Hospital, Ashford, UK; 584 Kansai Medical University Takii Hospital, Moriguchi, Japan; 585 Aristotle Medical School, Thessaloniki, Greece; 586 Japanese Red Cross Musashino Hospital, Tokyo, Japan; 587 Academic Medical Center, Amsterdam, Netherlands; 588 Medisch Spectrum Twente, Enschede, Netherlands; 589 University Modena, Modena, Italy; 590 Nuovo Ospedale Civile Sant’Agostino Estense, Modena, Italy; 591 Faculty of Medicine Alexandria University, Alexandria, Egypt; 592 Victoria Hospital, Kirkcaldy, UK; 593 Ziekenhuis Oost-Limburg, Genk, Belgium; 594 Chulalongkorn University, Bangkok, Thailand; 595 The Canberra Hospital, Hughes, ACT Australia; 596 ANU Medical School, Canberra, Australia; 597 “Spirito Santo” Hospital, Pescara, Italy; 598 St Lukes International Hospital, Akashi-Chou Chuo-Ku, Japan; 599 Kangwon National University, Chuncheonsi, South Korea; 600 Military Medical Academy, Sofia, Bulgaria; 601 Kangwon National University, Chuncheonsi, South Korea; 602 Kangwon National University, Chuncheonsi, South Korea; 603 Nuovo Ospedale Civile Sant’Agostino Estense, Modena, Italy; 604 University Modena, Modena, Italy; 605 Istanbul University, Medical Faculty of Istanbul, Anesthesiology and Intensive Care, Istanbul, Turkey; 606 Istanbul University, Medical Faculty of Istanbul, Department of Neuroradiology, Istanbul, Turkey; 607 Istanbul University, Institute of Experimental Medicine, Neuroscience, Istanbul, Turkey; 608 Bucharest Clinical Emergency Hospital, Bucharest, Romania; 609 Fundeni Clinical Institute, Bucharest, Romania; 610 Academic Medical Center, Amsterdam, Netherlands; 611 Medisch Spectrum Twente, Enschede, Netherlands; 612 Helsinki University Hospital, Helsinki, Finland; 613 North Karelia Central Hospital, Joensuu, Finland; 614 Kuopio University Hospital, Kuopio, Finland; 615 King’s College Hospital, London, UK; 616 Hospital Universitario Puerta del Mar, Cadiz, Spain; 617 Hospital Infanta Cristina, Badajoz, Spain; 618 The Ottawa Hospital, Ottawa, Canada; 619 Ulaval, Quebec City, Canada; 620 UBC, Vancouver, Canada; 621 UManitoba, Winnipeg, Canada; 622 OHRI, Ottawa, Canada; 623 The Ottawa Hospital, Ottawa, Canada; 624 Ulaval, Quebec City, Canada; 625 UBC, Vancouver, Canada; 626 UManitoba, Winnipeg, Canada; 627 Ottawa Hospital Research Institute, Ottawa, Canada; 628 Cliniques St Luc, Université catholique de Louvain, Brussels, Belgium; 629 Fondazione IRCCS Ca’ Granda Ospedale Maggiore Policlinico, Milan, Italy; 630 Milan University, Milan, Italy; 631 CHUV, Lausanne, Switzerland; 632 CHU de Nice, Nice, France; 633 Hospital Infanta Cristina, Badajoz, Spain; 634 Hospital Universitario Puerta del Mar, Cadiz, Spain; 635 Regional Hospital, Liberec, Czech Republic; 636 Institute of Experimental Medicine, Prague, Czech Republic; 637 Hennepin County Medical Center, Minneapolis, USA; 638 University of Glasgow, Glasgow, UK; 639 Institute of Neurological Sciences, NHSGGC, Glasgow, UK; 640 Department of Clinical Physics and Bioengineering, NHSGGC, Glasgow, UK; 641 Academic Unit of Anaesthesia, Pain and Critical Care Medicine, University of Glasgow, Glasgow, UK; 642 Smartimplant Ltd., Tallinn, Estonia; 643 NEMC, Tallinn, Estonia; 644 Universite Laval, Quebec, Canada; 645 University of Manitoba, Winnipeg, Canada; 646 Ottawa Hospital Research Institute, Ottawa, Canada; 647 Universite de Montreal, Montreal, Canada; 648 University of British Columbia, Vancouver, Canada; 649 Universite Laval, Quebec, Canada; 650 Dalhousie University, Halifax, Canada; 651 Ottawa Hospital Research Institute, Ottawa, Canada; 652 University of Manitoba, Winnipeg, Canada; 653 Universite de Montreal, Montreal, Canada; 654 University of British Columbia, Vancouver, Canada; 655 Hospital Estadual Getulio Vargas, Rio de Janeiro, Brazil; 656 Yonsei University College of Medicine, Seoul, South Korea; 657 Kantonsspital Aarau, Aarau, Switzerland; 658 Emergency Department, Groupe Hospitalier Pitié-Salpêtrière, Paris, France; 659 Morton Plant Hospital, Clearwater, USA; 660 Department of Laboratory Medicine, Kantonsspital Aarau, Aarau, Switzerland; 661 Royal Cornwall Hospital Trust, Truro, UK; 662 Manchester Royal Infirmary, Manchester, UK; 663 Hamad Medical Corporation, Doha, Qatar; 664 University of Tsukuba, Tsukuba Medical Center Hospital, Tsukuba, Japan; 665 King’s College Hospital, London, UK; 666 Kent, Surrey & Sussex Air Ambulance Trust, Kent, UK; 667 Prometheus Delta-Tech, Herefordshire, UK; 668 King’s College Hospital, London, UK; 669 Stavanger University Hospital, Stavanger, Norway; 670 Royal United Hospital, Bath, UK; 671 Aseer Central Hospital, Abha, Saudi Arabia; 672 King Saud bin Abdulaziz University for Health Sciences and King Abdullah International Medical Research Center, Riyadh, Saudi Arabia; 673 King Fahad Medical City, Riyadh, Saudi Arabia; 674 HagaZiekenhuis, Den Haag, Netherlands; 675 Reinier de Graaf Gasthuis, Delft, Netherlands; 676 Toxicological Research Center, Department of Clinical Toxicology, Loghman-Hakim Hospital, Shahid Beheshti University of Medical Sciences, Tehran, Iran; 677 Santa Maria University Hospital, Lisboa, Portugal; 678 St. Vincent’s University Hospital, Dublin 4, Ireland; 679 Percy Military Teaching Hospital, Clamart, France; 680 Whiston Hospital, Prescot, UK; 681 Glasgow Royal Infirmary, Glasgow, UK; 682 King Abdulaziz Medical City, National Guard Hospital, Riyadh, Saudi Arabia; 683 Department of Management, College of Business Administration, King Saud University, Saudi Arabia, Riyadh, Saudi Arabia; 684 King Saud bin Abdulaziz University for Health Sciences and King Abdullah International Medical Research Center, Riyadh, Saudi Arabia; 685 Ege University Hospital, Izmir, Turkey; 686 Katip Celebi University, Health Sciences Faculty, Izmir, Turkey; 687 Milton Keynes University Hospital NHS Foundation Trust, Milton Keynes, UK; 688 Faculty of Pharmacy, Mahidol University, Bangkok, Thailand; 689 Faculty of Medicine, Ramathibodi Hospital, Mahidol University, Bangkok, Thailand; 690 Specialist Anesthesia, Doha, Qatar; 691 Xanthi General Hospital, Xanthi, Greece; 692 Royal United Hospital, Bath, UK; 693 University of Canterbury, Christchurch, New Zealand; 694 Christchurch Hospital, Christchurch, New Zealand; 695 Rihard Knafelj, Ljubljana, Slovenia; 696 Clinical Center Ljubljana, Ljubljana, Slovenia; 697 The Royal Marsden Hospital, London, UK; 698 Bogomolets National Medical University, Kiev, Ukraine; 699 Aristotle Medical School, Thessaloniki, Greece; 700 Royal Liverpool Intensive Care Unit, Liverpool, UK; 701 University of Liverpool, Liverpool, UK; 702 Homerton University Hospital, London, UK; 703 University of Sao Paulo, Sao Paulo, Brazil; 704 University of Leicester, Leicester, UK; 705 King’s College Hospital, London, UK; 706 Research and Education Institute, Sao Paulo, Brazil; 707 King’s College Hospital, London, UK; 708 Ipswich Hospital NHS Trust, England, UK, Ipswich, UK; 709 Phramongkutklao Hospital, Bangkok, Thailand; 710 Phramongkutklao College of Medicine, Bangkok, Thailand; 711 Kantonsspital Aarau, Aarau, Switzerland; 712 Pusan National University Hospital, Busan, South Korea; 713 Royal Liverpool University Hospital, Liverpool, UK; 714 University of Liverpool, Liverpool, UK; 715 PICU, Hippokration General Hospital, Thessaloniki, Greece; 716 King Faisal Specialist Hospital & Research Centre, Riyadh, Saudi Arabia; 717 The Jikei University School of Medicine, Tokyo, Japan; 718 Albert Schweitzer State Hospital, Rio de Janeiro, Brazil; 719 PPG Internal Medicine, Federal University of Rio de Janeiro, Rio de Janeiro, Brazil; 720 Hospital Esperanca Recife, Recife, Brazil; 721 Hospital Total Cor, Rio de Janeiro, Brazil; 722 Hospital viValle, São José dos Campos, Brazil; 723 Hospital Rios DOr, Rio de Janeiro, Brazil; 724 Hospital Norte DOr, Rio de Janeiro, Brazil; 725 Hospital Esperanca Olinda, Olinda, Brazil; 726 DOr Institute for Research and Education - IDOR, Rio de Janeiro, Brazil; 727 Stamford Hospital, Stamford, USA; 728 Darent Valley Hospital, Dartford, UK; 729 William Harvey Hospital, Ashford, UK; 730 St James’s University Hospital, Leeds, UK; 731 Hospital Maciel, Montevideo, Uruguay; 732 DOr Institute for Research and Education - IDOR, Rio de Janeiro, Brazil; 733 Hospital de Cancer de Barretos, Barretos, Brazil; 734 Hospital Sírio Libanês, Sao Paulo, Brazil; 735 Sta. Casa de Porto Alegre, Porto Alegre, Brazil; 736 University of Pittsburgh Medical Center, Pittsburgh, USA; 737 Hospital Sao Lucas, Rio de Janeiro, Brazil; 738 Hospital Meridional S.A., Cariacica, Brazil; 739 Sheffield Teaching Hospitals, Sheffield, UK; 740 Whiston Hospital, St Helens & Knowsley, UK; 741 Centro Hospitalar e Universitário de Coimbra, Coimbra, Portugal; 742 Instituto Português de Oncologia Francisco Gil - Porto, Porto, Portugal; 743 King Saud bin Abdulaziz University for Health Sciences, Riyadh, Saudi Arabia; 744 Hôpital Louis Mourier, Colombes, France; 745 Hôpital Bichat, Paris, France; 746 Morriston Hospital, Swansea, UK; 747 Abertawe Bro Morgannwg University Health Board, Swansea, UK; 748 Centro Hospitalar Porto, Porto, Portugal; 749 Rihard Knafelj, Ljubljana, Slovenia; 750 Apollo Speciality Hospital - OMR, Chennai, India; 751 Hospital Meridional S.A., Cariacica, Brazil; 752 Rihard Knafelj, Ljubljana, Slovenia; 753 The Ottawa Hospital, Ottawa, Canada; 754 Kingston General Hospital, Kingston, Canada; 755 Milton Keynes Hospital, Milton Keynes, UK; 756 Imperial College, London, UK; 757 Buckinghamshire Healthcare NHS Trust, Aylesbury, UK; 758 IPO -Porto, Porto, Portugal; 759 Glasgow Royal Infirmary, Glasgow, UK; 760 KU Leuven, Leuven, Belgium; 761 Glasgow Royal Infirmary, Glasgow, UK; 762 Glasgow Royal Infirmary, Glasgow, UK; 763 Ernesto Dornelles Hospital, Porto Alegre, Brazil; 764 Glasgow Royal Infirmary, Glasgow, UK; 765 Gelre Ziekenhuizen, Apeldoorn, Netherlands; 766 University of Canterbury, Christchurch, New Zealand; 767 Christchurch Hospital, Christchurch, New Zealand; 768 Surrey and Sussex NHS Trust, Redhill, UK; 769 The Canberra Hospital, Hughes, ACT Australia; 770 ANU Medical School, Canberra, Australia; 771 Ege University School of Medicine, Izmir, Turkey; 772 katip Celebi University, Izmir, Turkey; 773 Alexandria University Faculty of medicine, Alexandria, Egypt; 774 Fortis Escorts Heart Institute, New Delhi, India; 775 FMRI, Gurgaon, India; 776 Philips Research North America, Cambridge, USA; 777 King’s College Hospital NHS Foundation Trust, London, UK; 778 Hospital Sao Rafael, Salvador, Brazil; 779 Hospital das Clinicas, Sao Paulo, Brazil; 780 Brigham and Women’s Hospital, Boston, USA; 781 St Helens and Knowsley, Liverpool, UK; 782 STH, Sheffield, UK; 783 Sheffield Teaching Hospitals, Sheffield, UK; 784 Whiston Hospital, Prescot, UK; 785 Freeman Hospital, Newcastle upon Tyne, UK; 786 Hospital Sao Rafael, Salvador, Brazil; 787 Hospital das Clinicas, Sao Paulo, Brazil; 788 Hospital Nove de Julho, Sao Paulo, Brazil; 789 University Hospital Coventry and Warwickshire, Coventry, UK; 790 Sunnybrook Health Sciences Centre, Toronto, Canada; 791 NHS Scotland, Glasgow, UK; 792 Royal College of Surgeons of Ireland, Dublin, Ireland; 793 University Leiden, Leiden, Netherlands; 794 Gelre Hospitals, Apeldoorn, Netherlands; 795 VU University Amsterdam, Amsterdam, Netherlands; 796 Hospital de Santo António, Oporto Hospital Center, Porto, Portugal; 797 Erasmus MC, Rotterdam, Netherlands; 798 Faculty of Psychology and Educational Sciences, Heerlen, Netherlands; 799 University of Toronto at Scarborough, Scarborough, ON Canada; 800 Hamad medical corporation, Doha, Qatar; 801 University of Szeged, Szeged, Hungary; 802 Jahn Ferenc Hospital, Budapest, Hungary; 803 University of Pécs, Pécs, Hungary; 804 Jessa Ziekenhuis, Hasselt, Belgium; 805 Hospital Clinico Universidad de Chile, Santiago, Chile; 806 Royal Marsden Hospital, London, UK; 807 Sheffield Teaching Hospitals, Sheffield, UK; 808 Tan Tock Seng hospital, Singapore, Singapore; 809 Centre Hospitalier de Lens, Lens, France; 810 Hospital Sao Rafael, Salvador, Brazil; 811 Hospital das Clinicas, Sao Paulo, Brazil; 812 Ntra Sra de Candelaria University Hospital, Santa Cruz de Tenerife, Spain; 813 University of Texas at Austin, San Antonio, USA; 814 Beaumont Hospital, Dublin, Ireland; 815 St Helens and Knowsley teaching hospitals, Liverpool, UK; 816 Nuovo Ospedale Civile Sant’Agostino Estense, Modena, Italy; 817 PICU, Hippokration General Hospital, Thessaloniki, Greece; 818 Maastricht University, Maastricht, Netherlands; 819 Maastricht University Medical Center, Maastricht, Netherlands; 820 University Hospital Antwerp, Edegem, Belgium; 821 Academic Medical Center, Amsterdam, Netherlands; 822 St Helens and Knoowsley, Prescot, UK; 823 University of Calgary, Calgary, Canada; 824 Vrije Universiteit Brussel, Brussel, Belgium; 825 EMGO+/VU University medical center, Utrecht, Netherlands; 826 Universitair Ziekenhuis Brussel, Brussel, Belgium; 827 University Hospital Lewisham, London, UK; 828 Queen Elizabeth Hospital, London, UK; 829 Lewisham & Greenwich NHS Trust, London, UK; 830 University of Glasgow, Glasgow, UK; 831 NHS Greater Glasgow and Clyde, Glasgow, UK; 832 National Burn Unit, Montevideo, Uruguay; 833 Hospital Maciel, Montevideo, Uruguay; 834 St Elisabeth Ziekenhuis, Tilburg, Netherlands; 835 Fondazione IRCCS Ca’ Granda - Ospedale maggiore Policlinico, Milan, Italy; 836 Istituto per lo Studio e la Prevenzione Oncologica, Florence, Italy

## Abstract

P001 - Sepsis impairs the capillary response within hypoxic capillaries and decreases erythrocyte oxygen-dependent ATP efflux

R. M. Bateman, M. D. Sharpe, J. E. Jagger, C. G. Ellis

P002 - Lower serum immunoglobulin G2 level does not predispose to severe flu.

J. Solé-Violán, M. López-Rodríguez, E. Herrera-Ramos, J. Ruíz-Hernández, L. Borderías, J. Horcajada, N. González-Quevedo, O. Rajas, M. Briones, F. Rodríguez de Castro, C. Rodríguez Gallego

P003 - Brain protective effects of intravenous immunoglobulin through inhibition of complement activation and apoptosis in a rat model of sepsis

F. Esen, G. Orhun, P. Ergin Ozcan, E. Senturk, C. Ugur Yilmaz, N. Orhan, N. Arican, M. Kaya, M. Kucukerden, M. Giris, U. Akcan, S. Bilgic Gazioglu, E. Tuzun

P004 - Adenosine a1 receptor dysfunction is associated with leukopenia: A possible mechanism for sepsis-induced leukopenia

R. Riff, O. Naamani, A. Douvdevani

P005 - Analysis of neutrophil by hyper spectral imaging - A preliminary report

R. Takegawa, H. Yoshida, T. Hirose, N. Yamamoto, H. Hagiya, M. Ojima, Y. Akeda, O. Tasaki, K. Tomono, T. Shimazu

P006 - Chemiluminescent intensity assessed by eaa predicts the incidence of postoperative infectious complications following gastrointestinal surgery

S. Ono, T. Kubo, S. Suda, T. Ueno, T. Ikeda

P007 - Serial change of c1 inhibitor in patients with sepsis – A prospective observational study

T. Hirose, H. Ogura, H. Takahashi, M. Ojima, J. Kang, Y. Nakamura, T. Kojima, T. Shimazu

P008 - Comparison of bacteremia and sepsis on sepsis related biomarkers

T. Ikeda, S. Suda, Y. Izutani, T. Ueno, S. Ono

P009 - The changes of procalcitonin levels in critical patients with abdominal septic shock during blood purification

T. Taniguchi, M. O

P010 - Validation of a new sensitive point of care device for rapid measurement of procalcitonin

C. Dinter, J. Lotz, B. Eilers, C. Wissmann, R. Lott

P011 - Infection biomarkers in primary care patients with acute respiratory tract infections – Comparison of procalcitonin and C-reactive protein

M. M. Meili, P. S. Schuetz

P012 - Do we need a lower procalcitonin cut off?

H. Hawa, M. Sharshir, M. Aburageila, N. Salahuddin

P013 - The predictive role of C-reactive protein and procalcitonin biomarkers in central nervous system infections with extensively drug resistant bacteria

V. Chantziara, S. Georgiou, A. Tsimogianni, P. Alexandropoulos, A. Vassi, F. Lagiou, M. Valta, G. Micha, E. Chinou, G. Michaloudis

P014 - Changes in endotoxin activity assay and procalcitonin levels after direct hemoperfusion with polymyxin-b immobilized fiber

A. Kodaira, T. Ikeda, S. Ono, T. Ueno, S. Suda, Y. Izutani, H. Imaizumi

P015 - Diagnostic usefullness of combination biomarkers on ICU admission

M. V. De la Torre-Prados, A. Garcia-De la Torre, A. Enguix-Armada, A. Puerto-Morlan, V. Perez-Valero, A. Garcia-Alcantara

P016 - Platelet function analysis utilising the PFA-100 does not predict infection, bacteraemia, sepsis or outcome in critically ill patients

N. Bolton, J. Dudziak, S. Bonney, A. Tridente, P. Nee

P017 - Extracellular histone H3 levels are inversely correlated with antithrombin levels and platelet counts and are associated with mortality in sepsis patients

G. Nicolaes, M. Wiewel, M. Schultz, K. Wildhagen, J. Horn, R. Schrijver, T. Van der Poll, C. Reutelingsperger

P018 - Il-8: is this a more reliable biomarker for sepsis severity than CRP, Procalcitonin, E-selectin, IL-6 and TNF-[alpha]

S. Pillai, G. Davies, G. Mills, R. Aubrey, K. Morris, P. Williams, P. Evans

P019 - Relation between adrenomedullin and short-term outcome in ICU patients: Results from the frog ICU study

E. G. Gayat, J. Struck, A. Cariou, N. Deye, B. Guidet, S. Jabert, J. Launay, M. Legrand, M. Léone, M. Resche-Rigon, E. Vicaut, A. Vieillard-Baron, A. Mebazaa

P020 - Impact of disease severity assessment on performance of heparin-binding protein for the prediction of septic shock

R. Arnold, M. Capan, A. Linder, P. Akesson

P021 - Kinetics and prognostic value of presepsin (sCD14) in septic patients. A pilot study

M. Popescu, D. Tomescu

P022 - Comparison of CD64 levels performed by the facs and accellix systems

C. L. Sprung, R. Calderon Morales, G. Munteanu, E. Orenbuch-Harroch, P. Levin, H. Kasdan, A. Reiter, T. Volker, Y. Himmel, Y. Cohen, J. Meissonnier

P023 - Diagnosing sepsis in 5 minutes: Nanofluidic technology study with pancreatic-stone protein (PSP/ reg)

L. Girard, F. Rebeaud

P024 - How nanotechnology-based approaches could contribute to sepsis prevention, diagnosis and treatment

I. Herrmann

P025 - Il7r transcriptional expression analysis during septic shock

B. Delwarde, E. Peronnet, E. Cerrato, F. Venet, A. Lepape, T. Rimmelé, G. Monneret, J. Textoris

P026 - Disbalance of microbial metabolites of aromatic acids affects the severity in critically ill patients

N. Beloborodova, V. Moroz, A. Osipov, A. Bedova, Y. Sarshor, A. Pautova, A. Sergeev, E. Chernevskaya

P027 - Copeptin predicts 10-year all-cause mortality in community patients

J. Odermatt, R. Bolliger, L. Hersberger, M. Ottiger, M. Christ-Crain, B. Mueller, P. Schuetz

P028 - Identification of differential proteomic response in septic patients secondary to community and hospital acquired pneumonia

N. K. Sharma, A. K. Tashima, M. K. Brunialti, F. R. Machado, M. Assuncao, O. Rigato, R. Salomao

P029 - Monocyte HLA-DR expression in community-acquired bacteremic sepsis - dynamics associated to aetiology and prediction of secondary sepsis

S. C. Cajander, G. Rasmussen, E. Tina, B. Söderquist, J. Källman, K. Strålin

P030 - Soluble B- and T-lymphocyte attenuator: A possible prognostic marker in sepsis

A. L. Lange, J. S. Sundén-Cullberg, A. M. Magnuson, O. H. Hultgren

P031 - Fractal dimension: A new biomarker for quantifying clot microstructure in patients across the sepsis spectrum

G. Davies, S. Pillai, G. Mills, R. Aubrey, K. Morris, P. Williams, P. Evans

P032 - Comparison between the new biomarker for coagulation, clot microstructure (Df) with rotational thromboelastometry (ROTEM) in patients across the sepsis spectrum

S. Pillai, G. Davies, G. Mills, R. Aubrey, K. Morris, P. Williams, P. Evans

P033 - Changes in fibrinolysis across the sepsis spectrum: The use of rotational thromboelastometry (ROTEM) lysis index (LI60) and D-Dimer concentration

S. Pillai, G. Davies, G. Mills, R. Aubrey, K. Morris, P. Williams, P. Evans

P034 - The intensive care infection score – a promising marker for the prediction of infection and its severity.

P. Van der Geest, M. Mohseni, J. Linssen, R. De Jonge, S. Duran, J. Groeneveld

P035 - Challenges in the clinical diagnosis of sepsis

R. Miller III, B. K. Lopansri, L. C. McHugh, A. Seldon, J. P. Burke

P036 - Does zero heat flux thermometry more accurately identify sepsis on intensive care?

J. Johnston, R. Reece-Anthony, A. Bond, A. Molokhia

P037 - Advancing quality (AQ) sepsis programme: Improving early identification & treatment of sepsis in North West England.

C. Mcgrath, E. Nsutebu

P038 - Prehospital transport of acute septic patients

P. Bank Pedersen, D. Pilsgaard Henriksen, S. Mikkelsen, A. Touborg Lassen

P039 - Vasodilatory plant extracts gel as an alternative treatment for fever in critically ill patients

R. Tincu, C. Cobilinschi, D. Tomescu, Z. Ghiorghiu, R. Macovei

P040 - Host response and outcome of hypothermic sepsis

M. A. Wiewel, M. B. Harmon, L. A. Van Vught, B. P. Scicluna, A. J. Hoogendijk, J. Horn, A. H. Zwinderman, O. L. Cremer, M. J. Bonten, M. J. Schultz, T. Van der Poll, N. P. Juffermans, W. J. Wiersinga

P041 - Septic shock alert over SIRS criteria has an impact on outcome but needs to be revised

G. Eren, Y Tekdos, M. Dogan, O. Acicbe, E. Kaya, O. Hergunsel

P042 - Association between previous prescription of βblockers and mortality rate among septic patients: A retrospective observational study

S. Alsolamy, G. Ghamdi, L. Alswaidan, S. Alharbi, F. Alenezi, Y. Arabi

P043 - Recognition and treatment of sepsis on labour ward– teaching & information resources can improve knowledge

J. Heaton, A. Boyce, L. Nolan, J. Johnston, A. Dukoff-Gordon, A. Dean, A. Molokhia

P044 - Culture negative sepsis in the ICU – what is unique to this patient population?

T. Mann Ben Yehudah

P045 - Organ dysfunction in severe sepsis patients identified in administrative data in Germany, 2007-2013

C. Fleischmann, D. Thomas-Rueddel, C. Haas, U. Dennler, K. Reinhart

P046 - A comparison of residents’ knowledge regarding; the Surviving Sepsis Campaign 2012 guideline

O. Suntornlohanakul, B. Khwannimit

P047 - Effectiveness of a septic shock bundle to improve outcomes in the ICU

F. Breckenridge, A. Puxty

P048 - Dose of norepinephrine in the first 24 hours as a parameter evaluating the effectiveness of treatment in patients with severe sepsis and septic shock

P. Szturz, P. Folwarzcny, J. Svancara, R. Kula, P. Sevcik

P049 - Norepinephrine or vasopressin + norepinephrine in septic shock. A retrospective series of 39 patients

L. Caneva, A. Casazza, E. Bellazzi, S. Marra, L. Pagani, M. Vetere, R. Vanzino, D. Ciprandi, R. Preda, R. Boschi, L. Carnevale

P050 - Methylene blue effectiveness as contributory treatment in patients with septic shock

V. Lopez, M. Aguilar Arzapalo, L. Barradas, A. Escalante, J. Gongora, M. Cetina

P051 - Coagulation disorders in patients with severe sepsis and DIC evaluated with thromboelastometry.

B Adamik, D Jakubczyk, A Kübler

P052 - Frequency and outcome of early sepsis-associated coagulopathy

A. Radford, T. Lee, J. Singer, J. Boyd, D. Fineberg, M. Williams, J. Russell

P053 - Assessment of coagulopathy in cancer patients with severe sepsis or septic shock. A case-control pilot study

E. Scarlatescu, D. Tomescu, G. Droc, S. Arama

P054 - Thromboelastometry in critically ill patients with disseminated intravascular coagulation

M. Müller, M. Straat, S. S. Zeerleder, N. P. Juffermans

P055 - Cessation of a preexisting chronic antiplatelet therapy is associated with increased mortality rates in severe sepsis and septic shock

C. F. Fuchs, C. S. Scheer, S. W. Wauschkuhn, M. V. Vollmer, K. M. Meissner, S. K. Kuhn, K. H. Hahnenkamp, S. R. Rehberg, M. G. Gründling

P056 - Neutrophil Extracellular Traps (NETs) production under hypoxic condition

N. Yamamoto, M. Ojima, S. Hamaguchi, T. Hirose, Y. Akeda, R. Takegawa, O. Tasaki, T. Shimazu, K. Tomono

P057 - Impact of ultraviolet air sterilizer in intensive care unit room, and clinical outcomes of patients

E. Gómez-Sánchez, M. Heredia-Rodríguez, E. Álvarez-Fuente, M. Lorenzo-López, E. Gómez-Pesquera, M. Aragón-Camino, P. Liu-Zhu, A. Sánchez-López, A. Hernández-Lozano, M. T. Peláez-Jareño, E. Tamayo

P058 - Focus of infection in severe sepsis - comparison of administrative data and prospective cohorts from Germany

D. O. Thomas-Rüddel, C. Fleischmann, C. Haas, U. Dennler, K. Reinhart

P059 - “Zero CLABSI” – can we get there? Obstacles on the 4 year journey and our strategies to overcome them – experience from an Indian ICU

V. Adora, A. Kar, A. Chakraborty, S. Roy, A. Bandyopadhyay, M. Das

P060 - Novel molecular techniques to identify central venous catheter (CVC) associated blood stream infections (BSIs)

T. Mann Ben Yehudah, G. Ben Yehudah, M. Salim, N. Kumar, L. Arabi, T. Burger, P. Lephart, E. Toth-martin

P061 - Zero clabsi” – can we get there? Obstacles on the 4 year journey and our strategies to overcome them – experience from an Indian ICU

R. Rao, A. Kar, A. Chakraborty

P062 - Prevention of central line-associated bloodstream infections in intensive care units: An international online survey

C. Valencia, N. Hammami, S. Blot, J. L. Vincent, M. L. Lambert

P063 - 30 days antimicrobial efficacy of non-leaching central venous catheters

J. Brunke, T. Riemann, I. Roschke

P064 - Efficacy of noble metal alloy-coated catheter in prevention of bacteriuria

R. Tincu, C. Cobilinschi, D. Tomescu, Z. Ghiorghiu, R. Macovei

P065 - Predicting bacteremic urinary tract infection in community setting: A prospective observational study

S. Nimitvilai, K. Jintanapramote, S. Jarupongprapa

P066 - Eight-year analysis of acinetobacter spp. monobacteremia in surgical and medical intensive care units at university hospital in Lithuania

D. Adukauskiene, D. Valanciene

P067 - Group A and group B streptococcal infections in intensive care unit – our experience in a tertiary centre

G. Bose, V. Lostarakos, B. Carr

P068 - Improved detection of spontaneous bacterial peritonitis by uritop + tm strip test and inoculation of blood culture bottles with ascitic fluid

S. Khedher, A. Maaoui, A. Ezzamouri, M. Salem

P069 - Increased risk of cellulitis in patients with congestive heart failure: a population based cohort study

J. Chen

P070 - Outcomes of severe cellulitis and necrotizing fasciitis in the critically ill

D. R. Cranendonk, L. A. Van Vught, M. A. Wiewel, O. L. Cremer, J. Horn, M. J. Bonten, M. J. Schultz, T. Van der Poll, W. J. Wiersinga

P071 - Botulism outbreak associated with people who inject drugs (PWIDs) in Scotland.

M. Day, G. Penrice, K. Roy, P. Robertson, G. Godbole, B. Jones, M. Booth, L. Donaldson

P072 - Surveillance of ESBL-producing enterobacteriaceae fecal carriers in the ICU

Y. Kawano, H. Ishikura

P073 - Prevalence of ESBL and carbapenemase producing uropathogens in a newly opened hospital in south India

S. Sreevidya, N. Brahmananda Reddy, P. Muraray Govind, R. Pratheema, J. Devachandran

Apollo Speciality Hospital - OMR, Chennai, India

P074 - Prevalence, risk factors and outcomes of methicillin-resistant staphylococcus aureus nasal colonization in critically ill patients

H. Al-Dorzi, M. Almutairi, B. Alhamadi, A. Crizaldo Toledo, R. Khan, B. Al Raiy, Y. Arabi

P075 - Multidrug-resistant Acinetobacter baumannii infection in intensive care unit patients in a hospital with building construction: Is there an association?

H. Talaie

P076 - Multidrug-resistant organisms in a Dutch ICU

J. A. Van Oers, A. Harts, E. Nieuwkoop, P. Vos

P077 - Epidemiology and risk factors of ICU acquired infections caused by multidrug-resistant gram negative bacilli

Y. Boussarsar, F. Boutouta, S. Kamoun, I. Mezghani, S. Koubaji, A. Ben Souissi, A. Riahi, M. S. Mebazaa

P078 - Improving outcomes of severe infections by multidrug-resistant pathogens with polyclonal IgM-enriched immunoglobulins

E. Giamarellos-Bourboulis, N. Tziolos, C. Routsi, C. Katsenos, I. Tsangaris, I. Pneumatikos, G. Vlachogiannis, V. Theodorou, A. Prekates, E. Antypa, V. Koulouras, N. Kapravelos, C. Gogos, E. Antoniadou, K. Mandragos, A. Armaganidis

P079 - Must change the medical practice in ICU?

A. R. Robles Caballero, B. Civantos, J. C. Figueira, J. López

P080 - Mediterranean spotted fever in an infectious diseases intensive care unit

A. Silva-Pinto, F. Ceia, A. Sarmento, L. Santos

P081 - Clinical features and outcomes of patients with Middle East respiratory syndrome requiring admission to a saudi intensive care unit: A retrospective analysis of 31 cases

G. Almekhlafi, Y. Sakr

P082 - The ICU response to a hospital outbreak of Middle East respiratory syndrome coronavirus infection

H. Al-Dorzi, R. Khan, S. Baharoon, A. Aldawood, A. Matroud, J. Alchin, S. Al Johani, H. Balkhy, Y. Arabi

P083 - Middle East respiratory syndrome: Surveillance data analysis

S. Alsolamy, S. Y. Yousif, B. O. Alotabi, A. S. Alsaawi

P085 - Use of Taqman array card molecular diagnostics in severe pneumonia: A case series

J. Ang, MD Curran, D. Enoch, V. Navapurkar, A. Conway Morris

P086 - ‘BUNS’: An investigation protocol improves the ICU management of pneumonia

R. Sharvill, J. Astin

P087 - Pneumonia in patients following secondary peritonitis: epidemiological features and impact on mortality

M. Heredia-Rodríguez, E. Gómez-Sánchez, M. T. Peláez-Jareño, E. Gómez-Pesquera, M. Lorenzo-López, P. Liu-Zhu, M. Aragón-Camino, A. Hernández-Lozano, A. Sánchez-López, E. Álvarez-Fuente, E. Tamayo

P088 - The use of the “CURB-65 score” by emergency room clinicians in a large teaching hospital

J. Patel, C. Kruger

P089 - Incidence of community acquired pneumonia with viral infection in mechanically ventilated patients in the medical intensive care unit

J. O’Neal, H. Rhodes, J. Jancik

P090 - The SAATELLITE Study: Prevention of S aureus Nosocomial Pneumonia (NP) with MEDI4893, a Human Monoclonal Antibody (mAb) Against S aureus

B. François, P. F. Laterre, P. Eggimann, A. Torres, M. Sánchez, P. F. Dequin, G. L. Bassi, J. Chastre, H. S. Jafri

P091 - Risk factors and microbiological profile for nosocomial infections in trauma patients

M. Ben Romdhane, Z. Douira, S. Kamoun, M. Bousselmi, A. Ben Souissi, Y. Boussarsar, A. Riahi, M.S. Mebazaa

P092 - Correlation between percentages of ventilated patients developed vap and use of antimicrobial agents in ICU patients.

A. Vakalos, V. Avramidis

P093 - A comparison of two ventilator associated pneumonia surveillance techniques

T. H. Craven, G. Wojcik, K. Kefala, J. McCoubrey, J. Reilly, R. Paterson, D. Inverarity, I. Laurenson, T. S. Walsh

P094 - Lung ultrasound before and after fiberbronchoscopy - modifications may improve ventilator-associated pneumonia diagnosis

S. Mongodi, B. Bouhemad, A. Orlando, A. Stella, G. Via, G. Iotti, A. Braschi, F. Mojoli

P095 - Comparing the accuracy of predictors of mortality in ventilator-associated pneumonia

M. Haliloglu, B. Bilgili, U. Kasapoglu, I. Sayan, M. Süzer Aslan, A. Yalcın, I. Cinel

P096 - Impact of pRBCs transfusion on percentage of ventilated patients developed VAP in ICU patients

A. Vakalos, V. Avramidis

P097 - The impact of a series of interventions on the rate of ventilator associated pneumonia in a large teaching hospital

H. E. Ellis, K. Bauchmuller, D. Miller, A Temple

P098 - The EVADE study: Prevention of Nosocomial Pneumonia (NP) caused by P aeruginosa with MEDI3902, a Novel Bispecific Monoclonal Antibody, against P aeruginosa virulence factors

J. Chastre, B. François, A. Torres, C. E. Luyt, M. Sánchez, M. Singer, H. S. Jafri

P099 - Short-term inhaled colistin adjunctive therapy for ventilator-associated pneumonia

Y. Nassar, M. S. Ayad

P100 - Effect of aerosolised colistin on weaning from mechanical ventilation

A. Trifi, S. Abdellatif, F. Daly, R. Nasri, S. Ben Lakhal

P101 - Septic shock is an independent risk factor for colistin-induced severe acute kidney injury: a retrospective cohort study

B. Bilgili, M. Haliloglu, F. Gul, I. Cinel

P102 - Nosocomial pneumonia - emphasis on inhaled tobramycin

A. Kuzovlev, A. Shabanov, S. Polovnikov, V. Moroz

P103 - In vitro evaluation of amikacin inhale and commercial nebulizers in a mechanical ventilator

N. Kadrichu, T. Dang, K. Corkery, P. Challoner

P104 - The effects of nebulized amikacin/fosfomycin and systemic meropenem on severe amikacin-resistant meropenem-susceptible P.aeruginosa pneumonia

G. Li Bassi, E. Aguilera, C. Chiurazzi, C. Travierso, A. Motos, L. Fernandez, R. Amaro, T. Senussi, F. Idone, J. Bobi, M. Rigol, A. Torres

P105 - Optimization of gentamicin peak concentrations in critically ill patients

C. J. Hodiamont, N. P. Juffermans, J. M. Janssen, C. S. Bouman, R. A. Mathôt, M. D. De Jong, R. M. Van Hest

P106 - Systematic review of cefepime induced neurotoxicity

L. Payne, G. L. Fraser

P107 - Unasyn® causes QT prolongation during treatment of intensive care patients

B. Tudor, M. Lahner, G. Roth, C. Krenn

P108 - Comparative study between teicoplanin and vancomycin in methicillin-resistant staphylococcus aureus (mrsa) infectious of toxicological intensive care unit (ticu) patients – Tehran, Iran

H. Talaie

P109 - Phage therapy against antimicrobial resistance, design of the first clinical study phagoburn

P. Jault, J. Gabard, T. Leclerc, S. Jennes, Y. Que, A. Rousseau, F. Ravat

P110 - Antibiotic dosing errors in critically ill patients with severe sepsis or septic shock

H. Al-Dorzi, A. Eissa, S. Al-Harbi, T. Aldabbagh, R. Khan, Y. Arabi

P111 - Does empiric antifungal therapy improve survival in septic critically ill patients? (immunocompromised excluded)

A. Trifi, S. Abdellatif, F. Daly, R. Nasri, S. Ben Lakhal

P112 - Neurocysticercosis-Qatar experience

F. Paramba, N. Purayil, V. Naushad, O. Mohammad, V. Negi, P. Chandra

P113 - Early indicators in acute haemorrhagic shock

A. Kleinsasser

P114 - Filtering of red blood cells reduces the inflammatory response of pulmonary cells in an in vitro model of mechanical ventilation

M. R. Witrz, J. F. Buchner-Doeven, A. M. Tuip-de Boer, J. C. Goslings, N. P. Juffermans

P115 - Microparticles from red blood cell transfusion induce a pro-coagulant and pro-inflammatory endothelial cell response

M. Van Hezel, M. Straat, A Boing, R Van Bruggen, N Juffermans

P116 - The contribution of cytokines on thrombosis development during hospitalization in ICU

D. Markopoulou, K. Venetsanou, V. Kaldis, D. Koutete, D. Chroni, I. Alamanos

P117 - Prophylactic enoxaparin dosing and adjustment through anti-xa monitoring in an inpatient burn unit

L. Koch, J. Jancik, H. Rhodes, E. Walter

P118 - Determination of optimal cut-off values of haemoglobin, platelet count and fibrinogen at 24 hours after injury associated with mortality in trauma patients

K. Maekawa, M. Hayakawa, S. Kushimoto, A. Shiraishi, H. Kato, J. Sasaki, H. Ogura, T. Matauoka, T. Uejima, N. Morimura, H. Ishikura, A. Hagiwara, M. Takeda

P119 - Trauma-induced coagulopathy - prothrombin complex concentrate vs fresh frozen plasma

O. Tarabrin, S. Shcherbakow, D. Gavrychenko, G. Mazurenko, V. Ivanova, O. Chystikov

P120 - First study to prove the superiority of prothrombin complex concentrates on mortality rate over fresh frozen plasma in patients with acute bleeding

C. Plourde, J. Lessard, J. Chauny, R. Daoust

P121 - Prothrombin complex concentrate vs fresh frozen plasma in obstetric massive bleeding

S. Shcherbakow, O. Tarabrin, D. Gavrychenko, G. Mazurenko, O. Chystikov

P122 - Impact of FFP transfusion on VAP in ICU patients

A. Vakalos, V. Avramidis

P123 - Preoperative platelet function test and the thrombin generation assay are predictive for blood loss after cardiac surgery

L. Kropman, L. In het Panhuis, J. Konings, D. Huskens, E. Schurgers, M. Roest, B. De Laat, M. Lance

P124 - Rotational thromboelastometry versus standard coagulation tests before surgical interventions

M. Durila, P. Lukas, M. Astraverkhava, J. Jonas

P125 - Correction of impaired clot quality and stability by fibrinogen and activated prothrombin complex concentrate in a model of severe thrombocytopenia

I. Budnik, B. Shenkman

P126 - Assessment of point-of-care prothrombin time analyzer as a monitor after cardiopulmonary bypass

H. Hayami, Y. Koide, T. Goto

P127 - Disseminated intravascular coagulation (dic) is underdiagnosed in critically ill patients: do we need d-dimer measurements?

R. Iqbal, Y. Alhamdi, N. Venugopal, S. Abrams, C. Downey, C. H. Toh, I. D. Welters

P128 - Validity of the age-adjusted d-dimer cutoff in patients with COPD

B. Bombay, J. M. Chauny, R. D. Daoust, J. L. Lessard, M. M. Marquis, J. P. Paquet

P129 - A scoping review of strategies for prevention and management of bleeding following paediatric cardiopulmonary bypass surgery

K. Siemens, D. Sangaran, B. J. Hunt, A. Durward, A. Nyman, I. A. Murdoch, S. M. Tibby

P130 - Nadir hemoglobulin during cardiopulmonary bypass: impact on postoperative morbidity and mortality

F. Ampatzidou, D. Moisidou, E. Dalampini, M. Nastou, E. Vasilarou, V. Kalaizi, H. Chatzikostenoglou, G. Drossos

P131 - Red blood cell transfusion do not influence the prognostic value of RDW in critically ill patients

S. Spadaro, A. Fogagnolo, T. Fiore, A. Schiavi, V. Fontana, F. Taccone, C. Volta

P132 - Reasons for admission in the paediatric intensive care unit and the need for blood and blood products transfusions

E. Chochliourou, E. Volakli, A. Violaki, E. Samkinidou, G. Evlavis, V. Panagiotidou, M. Sdougka

P133 - The implementation of a massive haemorrhage protocol (mhp) for the management of major trauma: a ten year, single-centre study

R. Mothukuri, C. Battle, K. Guy, G. Mills, P. Evans

P134 - An integrated major haemorrhage protocol for pre-hospital and retrieval medical teams

J. Wijesuriya, S. Keogh

P135 - The impact of transfusion thresholds on mortality and cardiovascular events in patients with cardiovascular disease (non-cardiac surgery): a systematic review and meta-analysis

A. Docherty, R. O’Donnell, S. Brunskill, M. Trivella, C. Doree, L. Holst, M. Parker, M. Gregersen, J. Almeida, T. Walsh, S. Stanworth

P136 - The relationship between poor pre-operative immune status and outcome from cardiac surgery is specific to the peri-operative antigenic threat

S. Moravcova, J. Mansell, A. Rogers, R. A. Smith, C. Hamilton-Davies

P137 - Impact of simple clinical practice guidelines for reducing post-operative atrial fibrillation after cardiac surgery.

A. Omar, M. Allam, O. Bilala, A. Kindawi, H. Ewila

P138 - Dexamethasone administration during cardiopulmonary bypass has no beneficial effects on elective postoperative cardiac surgery patients

F. Ampatzidou, D. Moisidou, M. Nastou, E. Dalampini, A. Malamas, E. Vasilarou, G. Drossos

P139 - Intra-aortic balloon counterpulsation in patients undergoing cardiac surgery (IABCS): preliminary results

G. Ferreira, J. Caldas, J. Fukushima, E. A. Osawa, E. Arita, L. Camara, S. Zeferino, J. Jardim, F. Gaioto, L. Dallan, F. B. Jatene, R. Kalil Filho, .F Galas, L. A. Hajjar

P140 - Effects of low-dose atrial natriuretic peptide infusion on cardiac surgery-associated acute kidney injury

C. Mitaka, T. Ohnuma, T. Murayama, F. Kunimoto, M. Nagashima, T. Takei, M. Tomita

P141 - Acute kidney injury influence on high sensitive troponin measurements after cardiac surgery

A. Omar, K. Mahmoud, S. Hanoura, S. Sudarsanan, P. Sivadasan, H. Othamn, Y. Shouman, R. Singh, A. Al Khulaifi

P142 - Complex evaluation of endothelial dysfunction markers for prognosis of outcomes in patients undergoing cardiac surgery

I. Mandel, S. Mikheev, I. Suhodolo, V. Kiselev, Y. Svirko, Y. Podoksenov

P143 - New-onset atrial fibrillation in intensive care: incidence, management and outcome

S. A. Jenkins, R. Griffin

P144 - One single spot measurement of the sublingual microcirculation during acute pulmonary hypertension in a pig model of shock

M. S. Tovar Doncel, A. Lima, C. Aldecoa, C. Ince

P145 - Assessment of levosimendan as a therapeutic option to recruit the microcirculation in cardiogenic shock – initial experience in cardiac ICU

A. Taha, A. Shafie, M. Mostafa, N. Syed, H. Hon

P146 - Terlipressin vs. norepinephrine in the Potential Multiorgan Donor(PMD)

F. Righetti, E. Colombaroli, G. Castellano

P147 - Echocardiography in the potential heart donor exposed to substitution hormonotherapy

F. Righetti, E. Colombaroli

P148 - Machine learning can reduce rate of monitor alarms

M. Hravnak, L. C. Chen, A. D. Dubrawski, G. C. Clermont, M. R. Pinsky

P149 - Peripherally inserted central catheters placed in the ICU

S. Gonzalez, D. Macias, J. Acosta, P. Jimenez, A. Loza, A. Lesmes, F. Lucena, C. Leon

P150 - Recordings of abnormal central venous pressure waveform morphology during an episode of pulmonary hypertension in a porcine shock model

M. S. Tovar Doncel, C. Ince, C. Aldecoa, A. Lima

P151 - Ultrasound guided central venous access technique among French intensivists

M. Bastide, J. Richecoeur, E. Frenoy, C. Lemaire, B. Sauneuf, F. Tamion, S. Nseir, D. Du Cheyron, H. Dupont, J. Maizel

P152 - Predictive ability of the Pv-aCO2 gap in patients with shock

M. Shaban, R. Kolko, N. Salahuddin, M. Sharshir, M. AbuRageila, A. AlHussain

P153 - Comparison of echocardiography and pulmonary artery catheter measurements of hemodynamic parameters in critical ill patients

P. Mercado, J. Maizel, L. Kontar, D. Titeca, F. Brazier, A. Riviere, M. Joris, T. Soupison, B. De Cagny, M. Slama

P154 - The volume clamp method for noninvasive cardiac output measurement in postoperative cardiothoracic surgery patients: a comparison with intermittent pulmonary artery thermodilution

J. Wagner, A. Körner, M. Kubik, S. Kluge, D. Reuter, B. Saugel

P155 - Hemodynamic monitoring in patients with septic shock (SS) – CPCCO (continuous pulse contour cardiac output) vs. TEE (transesophageal echocardiography)

E. Colombaroli, F. Righetti, G. Castellano

P156 - Cardiac output measurement with transthoracic echocardiography in critically ill patients: a pragmatic clinical study

T. Tran, D. De Bels, A. Cudia, M. Strachinaru, P. Ghottignies, J. Devriendt, C. Pierrakos

P157 - Left ventricular outflow tract velocity time integral correlates with stroke volume index in mechanically ventilated patients

Ó. Martínez González, R. Blancas, J. Luján, D. Ballesteros, C. Martínez Díaz, A. Núñez, C. Martín Parra, B. López Matamala, M. Alonso Fernández, M. Chana

P158 - Transpulmonary thermodilution (TPTD) derived from femoral vs. jugular central venous catheter: validation of a previously published correction formula and a proprietary correction formula for global end-diastolic volume index (GEDVI)

W. Huber, M. Eckmann, F. Elkmann, A. Gruber, I. Klein, R. M. Schmid, T. Lahmer

P160 - Dynamic arterial elastance calculated with lidcoplus monitor does not predict changes in arterial pressure after a fluid challenge in postsurgical patients

D. Bastoni, H. Aya, L. Toscani, L. Pigozzi, A. Rhodes, M. Cecconi

P159 - Venous return driving pressure and resistance in acute blood volume changes

P. W. Moller, S. Sondergaard, S. M. Jakob, J. Takala, D. Berger

P160 - Dynamic arterial elastance calculated with lidcoplus monitor does not predict changes in arterial pressure after a fluid challenge in postsurgical patients

D. Bastoni, H. Aya, L. Toscani, L. Pigozzi, A. Rhodes, M. Cecconi

P161 - Analysis of duration of post-operative goal-directed therapy protocol

C. Ostrowska, H. Aya, A. Abbas, J. Mellinghoff, C. Ryan, D. Dawson, A. Rhodes, M. Cecconi

P162 - Hemodynamic optimization – back to square one?

M. Cronhjort, O. Wall, E. Nyberg, R. Zeng, C. Svensen, J. Mårtensson, E. Joelsson-Alm

P163 - Effectiveness of fluid thoracic content measurement by bioimpedance guiding intravascular volume optimization in patients with septic shock

M. Aguilar Arzapalo, L. Barradas, V. Lopez, M. Cetina

P164 - A systematic review on the role of internal jugular vein ultrasound measurements in assessment of volume status in critical shock patients

N. Parenti, C. Palazzi, L. A. Amidei, F. B. Borrelli, S. C. Campanale, F. T. Tagliazucchi, G. S. Sedoni, D. L. Lucchesi, E. C. Carella, A. L Luciani

P165 - Importance of recognizing dehydration in medical Intensive Care Unit

M. Mackovic, N. Maric, M. Bakula

P166 - Effect of volume for a fluid challenge in septic patients

H. Aya, A. Rhodes, R. M. Grounds, N. Fletcher, M. Cecconi

P167 - Fluid bolus practices in a large Australian intensive care unit

B. Avard, P. Zhang

P168 - Liberal late fluid management is associated with longer ventilation duration and worst outcome in severe trauma patients: a retrospective cohort of 294 patients

M. Mezidi, J. Charbit, M. Ould-Chikh, P. Deras, C. Maury, O. Martinez, X. Capdevila

P169 - Association of fluids and outcomes in emergency department patients hospitalized with community-acquired pneumonia

P. Hou, W. Z. Linde-Zwirble, I. D. Douglas, N. S. Shapiro

P170 - Association of positive fluid balance with poor outcome in medicosurgical ICU patients

A. Ben Souissi, I. Mezghani, Y. Ben Aicha, S. Kamoun, B. Laribi, B. Jeribi, A. Riahi, M. S. Mebazaa

P171 - Impact of fluid balance to organ dysfunction in critically ill patients

C. Pereira, R. Marinho, R. Antunes, A. Marinho

P172 - Volume bolus in ICU patients: do we need to balance our crystalloids?

M. Crivits, M. Raes, J. Decruyenaere, E. Hoste

P173 - The use of 6 % HES solution do not reduce total fluid requirement in the therapy of patients with burn shock

V. Bagin, V. Rudnov, A. Savitsky, M. Astafyeva, I. Korobko, V. Vein

P174 - Electron microscopic assessment of acute kidney injury in septic sheep resuscitated with crystalloids or different colloids

T. Kampmeier , P. Arnemann, M. Hessler, A. Wald, K. Bockbreder, A. Morelli, H. Van Aken, S. Rehberg, C. Ertmer

P175 - Alterations of conjunctival microcirculation in a sheep model of haemorrhagic shock and resuscitation with 0.9 % saline or balanced tetrastarch

P. Arnemann, M. Hessler, T. Kampmeier, S. Rehberg, H. Van Aken, C. Ince, C. Ertmer

P176 - A single centre nested pilot study investigating the effect of using 0.9 % saline or Plasma-Lyte 148 ® as crystalloid fluid therapy on gastrointestinal feeding intolerance in mechanically ventilated patients receiving nasogastric enteral nutrition

S. Reddy, M. Bailey, R. Beasley, R. Bellomo, D. Mackle, A. Psirides, P. Young

P177 - A single centre nested pilot study investigating the effect on post-operative bleeding of using 0.9 % saline or Plasma-Lyte® 148 as crystalloid fluid therapy in adults in ICU after heart surgery

S. Reddy, M. Bailey, R. Beasley, R. Bellomo, D. Mackle, P. Young

P178 - Extreme hypernatremia and sepsis in a patient with Huntington’s dementia: a conundrum in fluid management

H. Venkatesh, S. Ramachandran, A. Basu, H. Nair

P179 - Diagnosis and management of severe hypernatraemia in the critical care setting

S. Egan, J. Bates

P180 - Correlation between arterial blood gas and electrolyte disturbances during hospitalization and outcome in critically ill patients

S. Oliveira, N. R. Rangel Neto, F. Q. Reis

P181 - Missing the “I” in MUDPILES – a rare cause of high anion gap metabolic acidosis (HAGMA)

C. P. Lee, X. L. Lin, C. Choong , K. M. Eu, W. Y. Sim , K. S. Tee, J. Pau , J. Abisheganaden

P182 - Plasma NGAL and urinary output: potential parameters for early initiation of renal replacement therapy

K. Maas, H. De Geus

P183 - Renal replacement therapy for critically ill patients: an intermittent continuity

E. Lafuente, R. Marinho, J. Moura, R. Antunes, A. Marinho

P184 - A survey of practices related to renal replacement therapy in critically ill patients in the north of England.

T. E. Doris, D. Monkhouse, T. Shipley, S. Kardasz, I Gonzalez

P185 - High initiation creatinine associated with lower 28-day mortality in critically ill patients necessitating continuous renal replacement therapy

S. Stads, A. J. Groeneveld

P186 - The impact of Karnofsky performance scale on outcomes in acute kidney injury patients receiving renal replacement therapy on the intensive care unit

I. Elsayed, N. Ward, A. Tridente, A. Raithatha

P187 - Severe hypophosphatemia during citrate-anticoagulated CRRT

A. Steuber, C. Pelletier, S. Schroeder, E. Michael, T. Slowinski, D. Kindgen-Milles

P188 - Citrate regional anticoagulation for post dilution continuous renal replacement therapy

S. Ghabina

P189 - Citrate 18 mmol/l improves anticoagulation during RRT with adsorbing filters

F. Turani, A. Belli, S. Busatti, G. Barettin, F. Candidi, F. Gargano, R. Barchetta, M. Falco

P190 - Calcium gluconate instead of calcium chloride in citrate-anticoagulated CVVHD

O. Demirkiran, M. Kosuk, S. Bozbay

P191 - Enhanced clearance of interleukin-6 with continuous veno-venous haemodialysis (CVVHD) using Ultraflux EMiC2 vs. Ultraflux AV1000S

V. Weber, J. Hartmann, S. Harm, I. Linsberger, T. Eichhorn, G. Valicek, G. Miestinger, C. Hoermann

P192 - Removal of bilirubin with a new adsorbent system: in vitro kinetics

S. Faenza, D. Ricci, E. Mancini, C. Gemelli, A. Cuoghi, S. Magnani, M. Atti

P193 - Case series of patients with severe sepsis and septic shock treated with a new extracorporeal sorbent

T. Laddomada, A. Doronzio, B. Balicco

P194 - In vitro adsorption of a broad spectrum of inflammatory mediators with CytoSorb® hemoadsorbent polymer beads

M. C. Gruda, P. O’Sullivan, V. P. Dan, T. Guliashvili, A. Scheirer, T. D. Golobish, V. J. Capponi, P. P. Chan

P195 - Observations in early vs. late use of cytosorb therapy in critically ill patients

K. Kogelmann, M. Drüner, D. Jarczak

P196 - Oxiris membrane decreases endotoxin during rrt in septic patients with basal EAA > 0,6

F. Turani, A. B. Belli, S. M. Martni, V. C. Cotticelli, F. Mounajergi, R. Barchetta

P197 - An observational prospective study on the onset of augmented renal clearance: the first report

S. Morimoto, H. Ishikura

P198 - An ultrasound- guided algorithm for the management of oliguria in severe sepsis

I. Hussain, N. Salahuddin, A. Nadeem, K. Ghorab, K. Maghrabi

P199 - Ultrasound in acute kidney injury (aki). First findings of farius, an education-programme in structural ultrasonography

S. K. Kloesel, C. Goldfuss, A. Stieglitz, A. S. Stieglitz, L. Krstevska, G. Albuszies

P200 - Effectiveness of renal angina index score predicting acute kidney injury on critically ill patients

M. Aguilar Arzapalo, L. Barradas, V. Lopez, A. Escalante, G. Jimmy, M. Cetina

P201 - Time length below blood pressure thresholds and progression of acute kidney injury in critically ill patients with or without sepsis: a retrospective, exploratory cohort study

J. Izawa, T. Iwami, S. Uchino, M. Takinami, T. Kitamura, T. Kawamura

P202 - Anaemia does not affect renal recovery in acute kidney injury

J. G. Powell-Tuck, S. Crichton, M. Raimundo, L. Camporota, D. Wyncoll, M. Ostermann

P203 - Estimated glomerular filtration rate based on serum creatinine: actual practice in Dutch ICU’s

A. Hana, H. R. De Geus

P204 - Comparison of estimated glomerular filtration rate calculated by mdrd, ckd-epi-serum-creatinine and ckd-epi-cystatin-c in adult critically ill patients

H. R. De Geus, A. Hana

P205 - Early diagnosis of septic acute kidney injury in medical critical care patients with a urine cell cycle arrest marker: insulin like growth factor binding protein-7 (IGFBP-7)

M. Aydogdu, N. Boyaci, S. Yuksel, G. Gursel, A. B. Cayci Sivri

P206 - Urinary neutrophil gelatinase-associated lipocalin as early biomarker of severe acute kidney injury in intensive care

J. Meza-Márquez, J. Nava-López, R. Carrillo-Esper

P207 - Shrunken pore syndrome is associated with a sharp rise in mortality in patients undergoing elective coronary artery bypass grafting

A. Dardashti, A. Grubb

P208 - The biomarker nephrocheck™ can discriminate the septic shock patients with an akin 1 or 2 acute renal failure who will not progress toward the akin 3 level

J. Maizel, M. Wetzstein, D. Titeca, L. Kontar, F. Brazier, B. De Cagny, A. Riviere, T. Soupison, M. Joris, M. Slama

P209 - A worldwide multicentre evaluation of acute kidney injury in septic and non-septic critically ill patients: the intensive care over nations (icon) audit

E. Peters, H. Njimi, P. Pickkers, J. L. Vincent

P210 - Does enhanced recovery after surgery reduce the incidence of acute kidney injury in those undergoing major gynae-oncological surgery?

M. Waraich , J. Doyle, T. Samuels, L. Forni

P211 - Identification of risk factors for the development of acute kidney injury after lower limb arthroplasty

N. Desai, R. Baumber, P. Gunning, A. Sell

P212 - Incidences and associations of acute kidney injury after major trauma

S. Lin, H. Torrence, M. O’Dwyer, C. Kirwan, J. Prowle

P213 - Acute kidney injury of major trauma patients

T Kim

P214 - Trajectory of serum creatinine after major surgery and the diagnosis of acute kidney injury

M. E. O’Connor, R. W. Hewson, C. J. Kirwan, R. M. Pearse, J. Prowle

P215 - Epidemiology of acute kidney injury after cardiac surgery. A single center retrospective study

S. Hanoura , A. Omar, H. Othamn, S. Sudarsanan , M. Allam, M. Maksoud, R. Singh, A. Al Khulaifi

P216 - Post-operative acute kidney injury after major non-cardiac surgery and its association with death in the following year

M. E. O’Connor, R. W. Hewson, C. J. Kirwan, R. M. Pearse, J. Prowle

P217 - Factors affecting acute renal failure in intensive care unit and effect of these factors on mortality

O. Uzundere, D. Memis , M. Ýnal, A. , N. Turan

P218 - Results of the live kidney transplantations according to national data of turkish organ and tissue information system

M. A. Aydin, H. Basar, I. Sencan, A. Kapuagasi, M. Ozturk, Z. Uzundurukan, D. Gokmen, A. Ozcan, C. Kaymak

P219 - Anaesthesia procedure and intensive therapy in patients with neck phlegmon

V. A. Artemenko, A. Budnyuk

P220 - Nasal high flow oygen for acute respiratory failure: a systematic review

R. Pugh , S. Bhandari

P221 - Setting optimal flow rate during high flow nasal cannula support: preliminary results

T. Mauri, C. Turrini, T. Langer, P. Taccone, C. A. Volta, C. Marenghi, L. Gattinoni, A. Pesenti

P222 - Dose to dose consistency across two different gas flow rates using cystic fibrosis and normal adult breathing profiles during nasal high flow oxygen therapy

L. Sweeney, A . O’ Sullivan, P. Kelly, E. Mukeria, R. MacLoughlin

P223 - Final results of an evaluation of airway medix closed suction system compared to a standard closed suction system

M. Pfeffer, J. T. Thomas, G. B. Bregman, G. K. Karp, E. K. Kishinevsky, D. S. Stavi, N. A. Adi

P224 - Different cuff materials and different leak tests - one size does not fit all

T. Poropat, R. Knafelj

P225 - Observational study on the value of the cuff-leak test and the onset of upper airway obstruction after extubation

E. Llopart, M. Batlle, C. De Haro, J. Mesquida, A. Artigas

P226 - A device for emergency transtracheal lung ventilation

D. Pavlovic, L. Lewerentz, A. Spassov, R. Schneider

P227 - Long-term outcome and health-related quality of life in patients discharged from the intensive care unit with a tracheostomy and with or without prolonged mechanical ventilation

S. De Smet, S. De Raedt, E. Derom, P Depuydt, S. Oeyen, D. Benoit, J. Decruyenaere

P228 - Ultrasound-guided percutaneous dilational tracheostomy versus bronchoscopy-guided percutaneous dilational tracheostomy in critically ill patients (trachus): a randomized clinical trial

A. Gobatto, B. Bese, P. Tierno, L. Melro, P. Mendes, F. Cadamuro, M. Park, L. M. Malbouisson

P229 - Is it safe to discharge patients with tracheostomy from the ICU to the ward?

B. C. Civanto, J. L. Lopez, A. Robles, J. Figueira, S. Yus, A. Garcia

P230 - The application of tracheostomy in children in ICU

A. Oglinda, G. Ciobanu, C. Oglinda, L. Schirca, T. Sertinean, V. Lupu

P231 - The impact of passive humidifiers on aerosol drug delivery during mechanical ventilation

P. Kelly, A. O’Sullivan, L. Sweeney, R. MacLoughlin

P232 - Evaluation of vibrating mesh and jet nebuliser performance at two different attachment setups in line with a humidifier nebuliser system

A. O’Sullivan, P. Kelly, L. Sweeney, E. Mukeria, M. Wolny , R. MacLoughlin

P233 - Psv-niv versus cpap in the treatment of acute cardiogenic pulmonary edema

A. Pagano, F. Numis, G. Vison, L. Saldamarco, T. Russo, G. Porta, F. Paladino

P234 - Noninvasive ventilation in patients with haematologic malignancy: a retrospective review

C. Bell, J. Liu, J. Debacker, C. Lee, E. Tamberg, V. Campbell, S. Mehta

P235 - Use of non-invasive ventilation in infectious diseases besides classical indications

A. Silva-Pinto, A. Sarmento, L. Santos

P236 - The impact of fragility on noninvasive mechanical ventilation application and results in the ICU

Ý. Kara, F. Yýldýrým, A. Zerman, Z. Güllü, N. Boyacý, B. Basarýk Aydogan, Ü. Gaygýsýz, K. Gönderen, G. Arýk, M. Turkoglu, M. Aydogdu, G. Aygencel, Z. Ülger, G. Gursel

P237 - Effects of metabolic alkalosis on noninvasive ventilation success and ICU outcome in patients with hypercapnic respiratory failure

N. Boyacý, Z. Isýkdogan, Ö. Özdedeoglu, Z. Güllü, M. Badoglu, U. Gaygýsýz, M. Aydogdu, G. Gursel

P238 - Asynchrony index and breathing patterns of acute exacerbation copd patients assisted with noninvasive pressure support ventilation and neurally adjusted ventilatory assist

N. Kongpolprom, C. Sittipunt

P239 - High frequency jet ventilation for severe acute hypoxemia

A. Eden, Y. Kokhanovsky, S. Bursztein – De Myttenaere, R. Pizov

P240 - HFOV revisited: a 7 year retrospective analysis of patients receiving HFOV who met oscillate trial entry criteria

L. Neilans, N. MacIntyre

P241 - Implementation of a goal-directed mechanical ventilation order set driven by respiratory therapists can improve compliance with best practices for mechanical ventilation

M. Radosevich, B. Wanta, V. Weber, T. Meyer, N. Smischney, D. Brown, D. Diedrich

P242 - A reduction in tidal volumes for ventilated patients on ICU calculated from IBW. can it minimise mortality in comparison to traditional strategies?

A . Fuller, P. McLindon, K. Sim

P243 - Predictive value of lung aeration scoring using lung ultrasound in weaning failure

M. Shoaeir, K. Noeam, A. Mahrous, R. Matsa, A. Ali

P244 - Conventional versus automated weaning from mechanical ventilation using SmartCare™

C. Dridi, S. Koubaji, S. Kamoun, F. Haddad, A. Ben Souissi, B. Laribi, A. Riahi, M. S. Mebazaa

P245 - Ultrasonographic evaluation protocol for weaning from mechanichal ventilation

A. Pérez-Calatayud, R. Carrillo-Esper, A. Zepeda-Mendoza, M. Diaz-Carrillo, E. Arch-Tirado

P246 - Diaphragm ultrasonography: a method for weaning patients from mechanical ventilation

S. Carbognin, L. Pelacani, F. Zannoni, A. Agnoli, G. Gagliardi

P247 - Dorsal diaphragmatic excursion tracks transpulmonary pressure in ventilated ARDS patients: a potential non-invasive indicator of lung recruitment?

R. Cho, A. Adams , S. Lunos, S. Ambur, R. Shapiro, M. Prekker

P248 - Pulse oximetry in the icu patient: is the perfusion index of any value?

M. Thijssen, L. Janssen, N. Foudraine

P249 - Ventilation is a better assessment of respiratory status than EtCO2

C. J. Voscopoulos, J. Freeman

P250 - Evaluation of the relationship between non-invasive minute ventilation and end-tidal CO2 in patients undergoing general vs spinal anesthesia

C. J. Voscopoulos, J. Freeman, E. George

P251 - Respiratory volume monitoring provides early warning of respiratory depression and can be used to reduce false alarms in non-intubated patients

C. J. Voscopoulos, D. Eversole, J. Freeman, E. George

P252 - P/i index: a predictive edi-derived weaning index during nava

S. Muttini, R. Bigi, G. Villani, N. Patroniti

P253 - Adequacy of ventilation in patients receiving opioids in the post anesthesia care unit: minute ventilation versus respiratory rate

G. Williams, C. J. Voscopoulos, J. Freeman, E. George

P254 - Comparison of regional and global expiratory time constants measured by electrical impedance tomography (EIT)

A. Waldmann, S. Böhm, W. Windisch, S. Strassmann, C. Karagiannidis

P255 - Electrical impedance tomography: robustness of a new pixel wise regional expiratory time constant calculation

A. Waldmann, S. Böhm, W. Windisch, S. Strassmann, C. Karagiannidis

P256 - Validation of regional and global expiratory time constant measurement by electrical impedance tomography in ards and obstructive pulmonary diseases

C. K. Karagiannidis, A. W. Waldmann, S. B. Böhm, S. Strassmann, W. W. Windisch

P257 - Transpulmonary pressure in a model with elastic recoiling lung and expanding chest wall

P. Persson, S. Lundin, O. Stenqvist

P258 - Lactate in pleural and abdominal effusion

G. Porta, F. Numis, C. S. Serra, A. P. Pagano, M. M. Masarone, L. R. Rinaldi, A. A. Amelia, M. F. Fascione, L. A. Adinolfi, E. R. Ruggiero

P259 - Outcome of patients admitted to the intensive care with pulmonary fibrosis

F. Asota, K. O’Rourke, S. Ranjan, P. Morgan

P260 - Sedation and analgesia practice in extra-corporeal membrane oxygenation (ECMO)-treated patients with acute respiratory distress syndrome (ARDS): a retrospective study

J. W. DeBacker, E. Tamberg, L. O’Neill, L. Munshi, L. Burry, E. Fan, S. Mehta

P261 - Characteristics and outcomes of patients deemed not eligible when referred for veno-venous extracorporeal membrane oxygenation (vv-ECMO)

S. Poo, K. Mahendran, J. Fowles, C. Gerrard, A. Vuylsteke

P262 - The SAVE SMR for veno-arterial ECMO

R. Loveridge, C. Chaddock, S. Patel, V. Kakar, C. Willars, T. Hurst, C. Park, T. Best, A. Vercueil, G. Auzinger

P263 - A simplified score to predict early (48 h) mortality in patients being considered for VA-ECMO

A. Borgman, A. G. Proudfoot, E. Grins, K. E. Emiley, J. Schuitema, S. J. Fitch, G. Marco, J. Sturgill, M. G. Dickinson, M. Strueber, A. Khaghani, P. Wilton, S. M. Jovinge

P264 - Lung function six months post extra corporeal membrane oxygenation (ECMO) for severe acute respiratory failure in adult survivors

C. Sampson, S. Harris-Fox

P265 - Bicarbonate dialysis removes carbon dioxide in hypoventilated rodents.

M. E. Cove, L. H. Vu, A. Sen, W. J. Federspiel, J. A. Kellum

P266 - Procalcitonin as predictor of primary graft dysfunction and mortality in post-lung transplantation

C. Mazo Torre, J. Riera, S. Ramirez, B. Borgatta, L. Lagunes, J. Rello

P267 - New molecular biomarkers of acute respiratory distress syndrome in abdominal sepsis

A. K. Kuzovlev, V. Moroz, A. Goloubev, S. Polovnikov, S. Nenchuk

P268 - Tight junction’s proteins claudin -5 and regulation by tnf in experimental murine lung injury model of ali/ards

V. Karavana, C. Glynos, A. Asimakos, K. Pappas, C. Vrettou, M. Magkou, E. Ischaki, G. Stathopoulos, S. Zakynthinos

P269 - Cell counts in endobronchial aspirate to assess airway inflammation in ARDS patients: a pilot study

S. Spadaro, I. Kozhevnikova, F. Dalla Corte, S. Grasso, P. Casolari, G. Caramori, C. Volta

P270 - Epidemiological and clinical profile of patients with acute respiratory distress syndrome in the surgical intensive care unit surgical, hospital JRA, Antananarivo

T. Andrianjafiarinoa, T. Randriamandrato, T. Rajaonera

P271 - Effect of high PEEP after recruitment maneuver on right ventricular function in ARDS. Is it good for the lung and for the heart?

S. El-Dash, ELV Costa, MR Tucci, F Leleu, L Kontar, B. De Cagny, F. Brazier, D. Titeca, G. Bacari-Risal, J. Maizel, M. Amato, M. Slama

P272 - Effect of recruitment maneuver on left ventricular systolic strain

P. Mercado, J. Maizel, L. Kontar, D. Titeca, F. Brazier, A. Riviere, M. Joris, T. Soupison, B. De Cagny, S. El Dash, M. Slama

P273 - Inhaled nitric oxide – is switching supplier cost effective?

Remmington, A. Fischer, S. Squire, M. Boichat

P274 - Epidemiological study of severe acute pancreatitis in Japan, comparison of the etiology and the patient outcomes on 1159 patients.

H. Honzawa, H. Yasuda, T. Adati, S. Suzaki, M. Horibe, M. Sasaki, M. Sanui

P275 - Extracorporeal liver support therapy. Experience in an intensive care unit

R. Marinho, J. Daniel, H. Miranda, A. Marinho

P276 - Accuracy of mortality prediction models in acute versus acute-on-chronic liver failure in the intensive care setting

K. Milinis, M. Cooper, G. R. Williams, E. McCarron, S. Simants, I. Patanwala, I. Welters

P277 - Risk of coronary artery disease in patients with chronic liver disease: a population based cohort study

Y. Su

P278 - 20 years of liver transplantation in Santiago de Compostela (Spain). Experience review

J. Fernández Villanueva, R. Fernández Garda, A. López Lago, E. Rodríguez Ruíz, R. Hernández Vaquero, S. Tomé Martínez de Rituerto, E. Varo Pérez

P279 - Diarrhea is a risk factor for liver injury and may lead to intestinal failure associated liver disease in critical illness

N. Lefel, F. Schaap, D. Bergmans, S. Olde Damink, M. Van de Poll

P280 - Bowel care on the intensive care unit: constipation guideline compliance and complications

K. Tizard, C. Lister, L. Poole

P281 - Malnutrition assessed by phase angle determines outcomes in low risk cardiac surgery patients

D. Ringaitiene, D. Gineityte, V. Vicka, I. Norkiene, J. Sipylaite

P282 - Preoperative fasting times in an irish hospital

A. O’Loughlin, V. Maraj, J. Dowling

P283 - Costs and final outcome of early x delayed feeding in a private Brazil ICU

M. B. Velasco, D. M. Dalcomune, E. B. Dias, S. L. Fernandes

P284 - Can ventilator derived energy expenditure measurements replace indirect calorimetry?

T. Oshima, S. Graf, C. Heidegger, L. Genton, V. Karsegard, Y. Dupertuis, C. Pichard

P285 - Revisiting the refeeding syndrome: results of a systematic review

N. Friedli, Z. Stanga, B. Mueller, P. Schuetz

P286 - Compliance with the new protocol for parenteral nutrition in our ICU

L. Vandersteen, B. Stessel, S. Evers, A. Van Assche, L. Jamaer, J. Dubois

P287 - Nutrition may be another treatment in the intensive care unit where less is more?

R. Marinho, H. Castro, J. Moura, J. Valente, P. Martins, P. Casteloes, C. Magalhaes, S. Cabral, M. Santos, B. Oliveira, A. Salgueiro, A. Marinho

P288 - Should we provide more protein to critically ill patients?

R. Marinho, M. Santos, E. Lafuente, H. Castro, S. Cabral, J. Moura, P. Martins, B. Oliveira, A. Salgueiro, S. Duarte, S. Castro, M. Melo, P. Casteloes, A. Marinho

P289 Protein provision in an adult intensive care unit

S. Gray

P290 - Prevalence and clinical outcomes of vitamin d deficiency in the medical critically ill patients in Songklanagarind hospital

K. Maipang, R. Bhurayanontachai

P291 - Vitamin d deficiency strongly predicts adverse medical outcome across different medical inpatient populations: results from a prospective study

L. G. Grädel, P. Schütz

P292 - Omega-3 fatty acids in patients undergoing cardiac surgery: a systematic review and meta-analysis

P. Langlois, W. Manzanares

P293 - Can 5-hydroxytriptophan prevent post-traumatic stress disorder in critically ill patients?

R. Tincu, C. Cobilinschi, D. Tomescu, Z. Ghiorghiu, R. Macovei

P294 - Parenteral selenium in the critically ill: an updated systematic review and meta-analysis

W. Manzanares, P. Langlois, M. Lemieux, G. Elke, F. Bloos, K. Reinhart, D. Heyland

P295 - Probiotics in the critically ill: an updated systematic review and meta-analysis

P. Langlois, M. Lemieux, I. Aramendi, D. Heyland, W. Manzanares

P296 - Diabetes with hyperglycemic crisis episodes may be associated with higher risk of pancreatic cancer: a population-based cohort study

Y. Su

P297 - Incidence of hypoglycemia in an intensive care unit depending on insulin protocol

R. Marinho, N. Babo, A. Marinho

P298 - Severity of the diseases is two-dimensionally correlated to blood glucose, including blood glucose variability, especially in moderately to severely ill patients with glucose intolerance.

M. Hoshino, Y. Haraguchi, S. Kajiwara, T. Mitsuhashi, T. Tsubata, M. Aida

P299 - A study of glycemic control by subcutaneous glargine injection transition from continuous regular insulin infusion in critically ill patients

T. Rattanapraphat, R. Bhurayanontachai, C. Kongkamol, B. Khwannimit

P300 - Glycemic control in Portuguese intensive care unit

R. Marinho, M. Santos, H. Castro, E. Lafuente, A. Salgueiro, S. Cabral, P. Martins, J. Moura, B. Oliveira, M. Melo, B. Xavier, J. Valente, C. Magalhaes, P. Casteloes, A. Marinho

P301 - Impact of hyperglycemia duration on the day of operation on short-term outcome of cardiac surgery patients

D. Moisidou, F. Ampatzidou, C. Koutsogiannidis, M. Moschopoulou, G. Drossos

P302 - Lactate levels in diabetic ketoacidosis patients at ICU admissions

G. Taskin, M. Çakir, AK Güler, A. Taskin, N. Öcal, S. Özer, L. Yamanel

P303 - Intensive care implications of merging heart attack centre units in London

J. M. Wong, C. Fitton, S. Anwar, S. Stacey

P304 - Special characteristics of in-hospital cardiac arrests

M. Aggou, B. Fyntanidou, S. Patsatzakis, E. Oloktsidou, K. Lolakos, E. Papapostolou, V. Grosomanidis

P305 - Clinical evaluation of ICU-admitted patients who were resuscitated in the general medicine ward

S. Suda , T. Ikeda, S. Ono, T. Ueno, Y. Izutani

P306 - Serious game evaluation of a one-hour training basic life support session for secondary school students: new tools for future bystanders

S. Gaudry, V. Desailly, P. Pasquier, PB Brun, AT Tesnieres, JD Ricard, D. Dreyfuss, A. Mignon

P307 - Public and clinical staff perceptions and knowledge of CPR compared to local and national data

J. C White, A. Molokhia, A. Dean, A. Stilwell, G. Friedlaender

P308 Dispatcher-assisted telephone cardiopulmonary resuscitation using a French-language compression-ventilation pediatric protocol

M. Peters, S. Stipulante, A. Delfosse, AF Donneau, A. Ghuysen

P309 Dantrolene versus amiodarone for resuscitation – an experimental study

C. Feldmann, D. Freitag, W. Dersch, M. Irqsusi, D. Eschbach, T. Steinfeldt, H. Wulf, T. Wiesmann

P310 Long term survival and functional neurological outcome in comatose survivors undergoing therapeutic hypothermia

N. Kongpolprom, J. Cholkraisuwat

P311 Impact of kidney disease on mortality and neurological outcome in out-of-hospital cardiac arrest: a prospective observational study

S. Beitland , E. Nakstad, H. Stær-Jensen , T. Drægni , G. Andersen , D. Jacobsen , C. Brunborg, B. Waldum-Grevbo , K. Sunde

P312 ICU dependency of patients admitted after primary percutaneous coronary intervention (PPCI) following out of the hospital cardiac arrest

K. Hoyland, D. Pandit

P313 Prognostic indicators and outcome prediction model for patients with return of spontaneous circulation from cardiopulmonary arrest: comprehensive registry of in-hospital intensive care on OHCA survival (critical) study in Osaka, Japan

K. Hayakawa

P314 Cerebral oxygen saturation during resuscitation in a porcine model of cardiac arrest

E. Oloktsidou, K. Kotzampassi, B. Fyntanidou, S. Patsatzakis, L. Loukipoudi, E. Doumaki, V. Grosomanidis

P315 Presumption of cardiopulmonary resuscitation for sustaining cerebral oxidation using regional cerebral saturation of oxygen: observational cohort study (press study)

H. Yasuda

P316 EEG reactivity in patients after cardiac arrest: a close look at stimuli

MM Admiraal, M. Van Assen, MJ Van Putten, M. Tjepkema-Cloostermans, AF Van Rootselaar, J. Horn

P317 Prognostic value of neuron-specific enolase after cardiac arrest

F. Ragusa, A. Marudi , S. Baroni, A. Gaspari, E. Bertellini

P318 Correlation between electroencephalographic findings and serum neuron specific enolase with outcome of post cardiac arrest patients

A. Taha, T. Abdullah, S. Abdel Monem

P319 Introduction of a targeted temperature management strategy following cardiac arrest in a district general hospital intensive care unit.

S. Alcorn, S. McNeill, S. Russell

P320 The evolution of cerebral oxygen saturation in post-cardiac arrest patients treated with therapeutic hypothermia

W. Eertmans, C. Genbrugge, I. Meex, J. Dens, F. Jans, C. De Deyne

P321 Prognostic factors and neurological outcomes of therapeutic hypothermia in comatose survivors from cardiac arrest: 8-year single center experience

J. Cholkraisuwat, N. Kongpolprom

P322 Adherence to targeted temperature management after out of hospital cardiac arrest

B. Avard, R. Burns

P323 Implementation of a therapeutic hypothermia protocol for comatose survivors of out-of-hospital cardiac arrest.

A. Patarchi, T. Spina

P324 Factors associated with ventilator weaning after targeted temperature management for cardiac arrest patients in japan

H. Tanaka, N. Otani, S. Ode, S. Ishimatsu

P325 Differential activation of c-fos in paraventricular nuclei of the hypothalamus and thalamus of the rat following myocardial infarction

J. Cho, J. B. Moon, C. W. Park, T. G. Ohk, M. C. Shin, M. H. Won

P326 Monitoring of cTroponin I in patients with acute ischemic stroke - predictor of inhospital mortality

S. Dakova, Z. Ramsheva, K. Ramshev

P327 Hyperthermic preconditioning severely accelerates neuronal damage in the gerbil ischemic hippocampal dentate gyrus via decreasing sods expressions

J. Cho, J. B. Moon, C. W. Park, T. G. Ohk, M. C. Shin

P328 Failure in neuroprotection of remote limb ischemic post conditioning in the hippocampus of a gerbil model of transient cerebral ischemia

J. Cho, J. B. Moon, C. W. Park, T. G. Ohk, M. C. Shin

P329 Brain death and admission diagnosis in neurologic intensive care unit, a correlation?

A Marudi, S Baroni, A Gaspari, E Bertellini

P330 Brain magnetic resonance imaging findings in patients with septic shock

G. Orhun, E. Senturk, P. E. Ozcan, S. Sencer, C. Ulusoy, E. Tuzun, F . Esen

P331 Benefits of L-carnitine in valproic acid induced encephalopathy

R. Tincu, C. Cobilinschi, D. Tomescu, Z. Ghiorghiu, R. Macovei

P332Automatic analysis of EEG reactivity in comatose patients

M. Van Assen, M. M. Admiraal, M. J. Van Putten, M. Tjepkema-Cloostermans, A. F. Van Rootselaar, J. Horn

P333 Usefulness of common ICU severity scoring systems in predicting outcome after spontaneous intracerebral hemorrhage

M. Fallenius, M. B. Skrifvars, M. Reinikainen, S. Bendel, R. Raj

P334 Evalution of patients with suspected subarachnoid haemorrhage and negative ct imaging

M. Abu-Habsa, C. Hymers, A. Borowska, H. Sivadhas, S. Sahiba, S. Perkins

P335 Timing of endovascular and surgical treatment for aneurysmal subarachnoid haemorrhage: early but not so fast.

J. Rubio, J. A. Rubio, R. Sierra

P336 Red blood cell transfusion in aneurysmal subarachnoid hemorrhage – the Sahara cohort study

S. English, M. Chasse, A. Turgeon, F. Lauzier, D. Griesdale, A. Garland, D. Fergusson, R. Zarychanski, A. Tinmouth, C. Van Walraven, K. Montroy, J. Ziegler, R. Dupont Chouinard, R. Carignan, A. Dhaliwal, C. Lum, J. Sinclair, G. Pagliarello, L. McIntyre

P337 - Aneurysmal subarachnoid hemorrhage and anemia: a canadian multi-centre retrospective cohort study

S. English, M. Chasse, A. Turgeon, F. Lauzier, D. Griesdale, A. Garland, D. Fergusson, R. Zarychanski, A. Tinmouth, C. Van Walraven, K. Montroy, J. Ziegler, R. Dupont Chouinard, R. Carignan, A. Dhaliwal, C. Lum, J. Sinclair, G. Pagliarello, L. McIntyre

P338 - Does the neutrophil-to-lymphocyte (NLR) ratio predict symptomatic vasospasm or delayed cerebral ischemia (DCI) after aneurysmal subarachnoid haemorrhage (SAH)?

T. Groza, N. Moreau, D. Castanares-Zapatero, P. Hantson

P339 - ICU-acquired infections in aneurysmal subarachnoid hemorrhage patients: impact on ICU and hospital length of stay

M. Carbonara , F. Ortolano, T. Zoerle, S. Magnoni, S. Pifferi, V. Conte, N. Stocchetti

P340 - Cerebral metabolic effects of normobaric hyperoxia during the acute phase of aneurysmal subarachnoid hemorrhage

L. Carteron, T. Suys, C. Patet, H. Quintard, M. Oddo

P341 - Postoperative care for elective craniotomy: where is best done?

J. A. Rubio, J. Rubio, R. Sierra

P342 - 5-year follow-up of patients after transplantation of organs from donors from neurocritical care

V. Spatenkova, E. Pokorna, P. Suchomel

P343 - Evaluation of levetiracetam pharmacokinetics after severe traumatic brain injury in neurocritical care patients at a level one trauma center

N. Ebert, J. Jancik, H. Rhodes

P344 - Model based time series cluster analysis to determine unique patient states in traumatic brain injury

T. Bylinski, C. Hawthorne, M. Shaw, I. Piper, J. Kinsella

P345 - Brain compartment monitoring capabilities from ICP to BI (bioimpedance) during HS (hypertonic saline) administration. State of art simulation outcome depending on brain swelling type

A. K. Kink , I. R. Rätsep

P346 - Transfusion of red blood cells in patients with traumatic brain injury admitted to Canadian trauma health centers: a multicenter cohort study

A. Boutin, L. Moore, M. Chasse, R. Zarychanski, F. Lauzier, S. English, L. McIntyre, J. Lacroix, D. Griesdale, P. Lessard-Bonaventure, A. F. Turgeon

P347 - Hemoglobin thresholds and red blood cell transfusions in adult patients with moderate or severe traumatic brain injury: a retrospective cohort study

A. Boutin, L. Moore, R. Green, P. Lessard-Bonaventure, M. Erdogan, M. Butler, F. Lauzier, M. Chasse, S. English, L. McIntyre, R. Zarychanski, J. Lacroix, D. Griesdale, P. Desjardins, D. A. Fergusson, A. F. Turgeon

P348 - Characteristics of patients with gunshot wounds to the head - an observational Brazilian study

B. Goncalves, B. Vidal, C. Valdez, A. C. Rodrigues, L. Miguez, G. Moralez

P349 - Base excess as predictor for ICU admission and the injury severity in blunt trauma patients

T. Hong

P350 - Enhancement of usual emergency department care with proadrenomedullin to improve outcome prediction - Results from the multi-national, prospective, observational TRIAGE study

A. Kutz, P. Hausfater, D. Amin, T. Struja, S. Haubitz, A. Huber, B. Mueller, P. Schuetz

P351 - Developing an innovative emergency medicine point-of-care simulation programme

T. Brown, J. Collinson, C. Pritchett, T. Slade

P352 - The InSim program: an in situ simulation program for junior trainees in intensive care

M. Le Guen, S. Hellings, R. Ramsaran

P353 - Impact of excessive and inappropriate troponin testing in the emergency setting how good are we

A. Alsheikhly

P354 - The development of time tracking monitor at emergency department

T. Abe

P355 - Role of focussed echocardiography in emergency assessment of syncope

L. Kanapeckaite, M. Abu-Habsa, R. Bahl

P356 - Insertion of an open-ended 14-gauge catheter through the chest wall causes a significant pneumothorax in a self-ventilating swine model

M. Q Russell, K. J. Real, M. Abu-Habsa , R. M. Lyon, N. P. Oveland

P357 - Ez-io® intraosseous access teaching in the workplace using a mobile ‘tea trolley’ training method

J. Penketh, M. Mcdonald, F. Kelly

P358 - Black widow envenomation in Saudi Arabia: a prospective observational case series

M. Alfafi, S. Alsolamy, W. Almutairi, B. Alotaibi

P359 - Mechanical ventilation in patients with overdose not yet intubated on icu admission

A. E. Van den Berg, Y. Schriel, L. Dawson, I. A. Meynaar

P360 - Central nervous system depressants poisoning and ventilator associated pneumonia: an underrated risk factor in toxicological intensive care unit

H. Talaie

P361 - Acute barium intoxication treated with hemodiafiltration

D. Silva, S. Fernandes, J. Gouveia, J. Santos Silva

P362 - Major trauma presenting to the emergency department. the spectrum of cycling injuries in Ireland

J. Foley, A. Kaskovagheorgescu, D. Evoy, J. Cronin, J. Ryan

P363 - Burns from French military operations: a 14-year retrospective observational analysis.

M. Huck, C. Hoffmann, J. Renner, P. Laitselart, N. Donat, A. Cirodde, J. V. Schaal, Y. Masson, A. Nau, T. Leclerc

P364 - A comparison of mortality scores in burns patients on the intensive care unit.

O. Howarth, K. Davenport, P. Jeanrenaud, S. Raftery

P365 - Clasification of pain and its treatment and an intensive care rehabiliation clinic

P. MacTavish, H. Devine, J. McPeake, M. Daniel, J. Kinsella, T. Quasim

P366 - Pain management adequacy in critical care areas ,the process and the barriers perceived by critical care nurses

S. Alrabiee, A. Alrashid , S. Alsolamy

P367 - Pain assessment in critically ill adult patients: validation of the Turkish version of the critical-care pain observation tool

O. Gundogan, C. Bor, E. Akýn Korhan, K. Demirag , M. Uyar

P368 - An audit of pain and sedation assessments in the intensive care unit: recommendations for clinical practice

F. Frame, C. Ashton, L. Bergstrom Niska

P369 - Impact of pharmaceutical care on treatment of pain and agitation in medical intensive care unit

P. Dilokpattanamongkol, T. Suansanae, C. Suthisisang, S. Morakul, C. Karnjanarachata, V. Tangsujaritvijit

P370 - Agitation in trauma ICU, prevention and outcome

S. Mahmood, H. Al Thani, A. Almenyar

P371 Correlation between percentages of ventilated patients developed vap and use of sedative agents in icu patients.

A. Vakalos , V. Avramidis

P372 - Improving recording of sedation events in the Emergency Department: The implementation of the SIVA International Taskforce adverse event reporting tool for procedural sedation

R. Sharvill, J. Penketh

P373 - Impact of sedative drug use on the length of mechanical ventilation

S. E. Morton, Y. S. Chiew, C. Pretty, J. G. Chase, G. M. Shaw

P374 - Co-administration of nitric oxide and sevoflurane using anaconda

R. Knafelj, P. Kordis

P375 - A retrospective study of the use of Dexmedetomidine in an oncological critical care setting

S. Patel, V. Grover

P376 - Dexmedetomidine and posttraumatic stress disorder incidence in alcohol withdrawal icu patients

I. Kuchyn, K. Bielka

P377 - Hemodynamic effects of dexmedetomidine in a porcine model of septic shock

Z. Aidoni, V. Grosomanidis, K. Kotzampassi, G. Stavrou, B. Fyntanidou, S. Patsatzakis, C. Skourtis

P378 - Ketamine for analgosedation in severe hypoxic respiratory failure

S. D. Lee, K. Williams, I. D. Weltes

P379 - Madness from the moon? lunar cycle and the incidence of delirium on the intensive care unit

S. Berhane, C. Arrowsmith, C. Peters, S. Robert

P380 - Impaired dynamic cerebral autoregulation after coronary artery bypass grafting and association with postoperative delirium

J. Caldas, R. B. Panerai, T. G. Robinson, L. Camara, G. Ferreira, E. Borg-Seng-Shu, M. De Lima Oliveira, N. C. Mian, L. Santos, R. Nogueira, S. P. Zeferino, M. Jacobsen Teixeira, F. Galas, L. A. Hajjar

P381 - Risk factors predicting prolonged intensive care unit length of stay after major elective surgery.

P. Killeen, M. McPhail, W. Bernal, J. Maggs, J. Wendon, T. Hughes

P382 - Systemic inflammatory response syndrome criteria and hospital mortality prediction in a brazilian cohort of critically ill patients

L. U. Taniguchi, E. M. Siqueira, J. M. Vieira Jr, L. C. Azevedo

P383 - Evaluating the efficacy of a risk predictor panel in identifying patients at elevated risk of morbidity following emergency admission

A. N. Ahmad, M. Abu-Habsa, R. Bahl, E. Helme, S. Hadfield, R. Loveridge

P384 - A retrospective comparison of outcomes for elective surgical patients admitted post-operatively to the critical care unit or general ward

J. Shak, C. Senver, R. Howard-Griffin

P385 - Effect of obesity on mortality in surgical critically ill patients.

P. Wacharasint, P. Fuengfoo, N. Sukcharoen, R. Rangsin

P386 - The national early warning score (news) reliably improves adverse clinical outcome prediction in community-acquired pneumonia - results from a 6 year follow-up

D. Sbiti-Rohr, P. Schuetz

P387 - Clinical usefulness of the charlson¡¯s weighted index of comorbidities _as prognostic factor in patients with prolonged acute mechanical ventilation

H. Na, S. Song, S. Lee, E. Jeong, K. Lee

P388 - Comparison of mortality prediction scoring systems in patients with cirrhosis admitted to general intensive care unit

M. Cooper, K. Milinis, G. Williams, E. McCarron, S. Simants, I. Patanwala, I. D. Welters

P389 - Impact of admission source and time of admission on outcome of pediatric intensive care patients: retrospective 15 years study

E. Zoumpelouli, EA Volakli, V. Chrysohoidou, S. Georgiou, K. Charisopoulou, E. Kotzapanagiotou, V. Panagiotidou, K. Manavidou, Z. Stathi, M. Sdougka

P390 - Heart rate variability and outcomes prediction in critical illness

N. Salahuddin, B. AlGhamdi, Q. Marashly, K. Zaza, M. Sharshir, M. Khurshid, Z. Ali, M. Malgapo, M. Jamil, A. Shafquat, M. Shoukri, M. Hijazi

P391 - The incidence and outcome of hyperlactatemia in the post anaesthesia care unit

T. Abe, S. Uchino, M. Takinami

P392 - Correlation between arterial blood gas disturbances and arterial lactate levels during hospitalization and outcome in critically septic patients

N. R. Rangel Neto, S. Oliveira, F. Q. Reis, F. A. Rocha

P393 - External validation of saps 3 and mpm iii scores in 48,816 patients from 72 brazilian icus

G. Moralez, K. Ebecken, L. S. Rabello, M. F. Lima, R. Hatum, F. V. De Marco, A. Alves, J. E. Pinto, M. Godoy, P. E. Brasil, F. A. Bozza, J. I. Salluh, M. Soares

P394 - The frailty penalty: pre-admission functional status confounds mortality prediction models in critically ill patients

J. Krinsley, G. Kang

P395 - ‘sooner rather than later”: how delayed discharge from critical care leads to increased out of hours discharges and subsequent increase in in-hospital mortality.

J. Perry, H. Hines

P396 - Identifying poor outcome patient groups in a resource-constrained critical care unit

K. M. Wilkinson, C. Tordoff, B. Sloan, M. C. Bellamy

P397 - Effects of icu weekend admission and discharge on mortality.

E. Moreira, F. Verga, M. Barbato, G. Burghi

P398 - Organizational factors, outcomes and resource use in 9,946 cancer patients admitted to 70 ICUs

M Soares, U. V. Silva, L. C. Azevedo, A. P. Torelly, J. M. Kahn, D. C. Angus, M. F. Knibel, P. E. Brasil, F. A. Bozza, J. I. Salluh

P399 - Evaluation of oncological critically ill patients, severity score and outcome compared to not oncological in a particular hospital cti.

M. B. Velasco, D. M. Dalcomune

P400 - Outcomes of patients admitted to a large uk critical care department with palliative oncological diagnoses

R. Marshall, T. Gilpin, A. Tridente, A. Raithatha

P401 - Predictors of mortality in febrile neutropenic patients with haematological malignancies admitted to an intensive care unit of a cancer center

D. Mota, B. Loureiro, J. Dias, O. Afonso, F. Coelho, A. Martins, F. Faria

P402 - Patients with hematologic malignancies requiring invasive mechanical ventilation: characteristics and predictors of mortality

H. Al-Dorzi, H. Al Orainni , F. AlEid, H. Tlaygeh, A. Itani, A. Hejazi, Y. Arabi

P403 - Patient-important outcomes in randomized controlled trials in critically ill patients: a systematic review

S. Gaudry, J. Messika, J. D. Ricard, S. Guillo, B. Pasquet, E. Dubief, D. Dreyfuss, F. Tubach

P404 - Alopecia in survivors of critical illness: a qualitative study

C . Battle, K. James, P. Temblett

P405 - The impact of mental health on icu admission

L. Davies, C. Battle, C. Lynch

P406 - Cognitive impairment 5 years after ICU discharge

S. Pereira, S. Cavaco, J. Fernandes, I. Moreira, E. Almeida, F. Seabra Pereira, M. Malheiro, F. Cardoso, I. Aragão, T. Cardoso

P407 - Apache ii versus apache iv for octagenerians in medical icu

M. Fister, R. Knafelj

P408 - Outcomes of octagenarians in an indian icu

P. Muraray Govind, N. Brahmananda Reddy, R. Pratheema, E. D. Arul, J. Devachandran

P409 - Mortality and outcomes in elderly patients 80 years of age or older admitted to the icu

M. B. Velasco , D. M. Dalcomune

P410 - Octagenerians in medical icu - adding days to life or life to days?

R. Knafelj, M. Fister

P411 - The very elderly admitted to intensive care unit: outcomes and economic evaluation

N. Chin-Yee, G. D’Egidio, K. Thavorn, D. Heyland, K. Kyeremanteng

P412 - The very elderly in intensive care: relationship between acuity of illness and long-term mortality

A. G. Murchison, K. Swalwell, J. Mandeville, D. Stott

P413 - Acquired weakness in an oncological intensive care unit

I. Guerreiro

P414 - Musculoskeletal problems in intensive care unit (ICU) patients post-discharge

H. Devine, P. MacTavish, J. McPeake, T. Quasim, J. Kinsella, M. Daniel

P415 - Premorbid obesity, but not nutrition, prevents critical illness-induced muscle wasting and weakness

C. Goossens M. B. Marques, S. Derde, S. Vander Perre, T. Dufour, S. E. Thiessen, F. Güiza, T. Janssens, G. Hermans, I. Vanhorebeek, K. De Bock, G. Van den Berghe, L. Langouche

P416 - Physical outcome measures for critical care patients following intensive care unit (icu) discharge

H. Devine, P. MacTavish, T. Quasim, J. Kinsella, M. Daniel, J. McPeake

P417 - Improving active mobilisation in a general intensive care unit

B. Miles , S. Madden, H. Devine

P418 - Mobilization in patients on vasoactive drugs use – a pilot study.

M. Weiler, P. Marques, C. Rodrigues, M. Boeira, K. Brenner, C. Leães, A. Machado, R. Townsend, J. Andrade

P419 - Pharmacy intervention at an intensive care rehabilitation clinic

P. MacTavish, J. McPeake, H. Devine, J. Kinsella, M. Daniel, R. Kishore, C. Fenlon, T. Quasim

P420 - Interactive gaming is feasible and potentially increases icu patients’ motivation to be engaged in rehabilitation programs

T. Fiks, A. Ruijter, M. Te Raa, P. Spronk

P421 - Simulation-based design of a robust stopping rule to ensure patient safety

Y. S. Chiew, P. Docherty, J. Dickson, E. Moltchanova, C. Scarrot, C. Pretty, G. M. Shaw, J. G. Chase

P422 - Are daily blood tests on the intensive care unit necessary?

T. Hall, W. C. Ngu, J. M. Jack, P. Morgan

P423 - Measuring urine output in ward patients: is it helpful?

B. Avard, A. Pavli, X. Gee

P424 - The incidence of pressure ulcers in an adult mixed intensive care unit in turkey

C . Bor, E. Akin Korhan, K. Demirag, M. Uyar

P425 - Intensivist/patient ratios in closed ICUs in Alexandria, Egypt; an overview

M. Shirazy, A. Fayed

P426 - Eicu (electronic intensive care unit): impact on ALOS (average length of stay) in a developing country like India

S. Gupta, A. Kaushal, S. Dewan, A. Varma

P427 - Predicting deterioration in general ward using early deterioration indicator

E. Ghosh, L. Yang, L. Eshelman, B. Lord, E. Carlson

P428 - High impact enhanced critical care outreach - the imobile service: making a difference

E. Helme, R. Broderick, S. Hadfield, R. Loveridge

P429 - Impact of bed availability and cognitive load on intensive care unit (ICU) bed allocation: a vignette-based trial

J. Ramos, D. Forte

P430 - Characteristics of critically ill patients admitted through the emergency department

F. Yang, P. Hou

P431 - Admission to critical care: the quantification of functional reserve

J. Dudziak, J. Feeney, K. Wilkinson, K. Bauchmuller, K. Shuker, M. Faulds, A. Raithatha, D. Bryden, L. England, N. Bolton, A. Tridente

P432 - Admission to critical care: the importance of frailty

K. Bauchmuller, K Shuker, A Tridente, M Faulds, A Matheson, J. Gaynor, D Bryden, S South Yorkshire Hospitals Research Collaboration

P433 - Development of an instrument to aid triage decisions for intensive care unit admission

J. Ramos, B. Peroni, R. Daglius-Dias, L. Miranda, C. Cohen, C. Carvalho, I . Velasco, D. Forte

P434 - Using selective serotonin re-uptake inhibitors and serotonin-norepinephrine re-uptake inhibitors in critical care: a systematic review of the evidence for benefit or harm

J. M. Kelly, A. Neill, G. Rubenfeld, N. Masson, A. Min

P435 - Measuring adaptive coping of hospitalized patients with a severe medical condition:the sickness insight in coping questionnaire (sicq)

E. Boezeman, J. Hofhuis , A. Hovingh, R. De Vries, P. Spronk

P436 - Results of a national survey regarding intensive care medicine training

G. Cabral-Campello, I. Aragão, T. Cardoso

P437 - Work engagement among healthcare professionals in the intensive care unit

M. Van Mol, M. Nijkamp, E . Kompanje

P438 - Empowering the intensive care practitioners. is it a burnout ameliorating intervention?

P. Ostrowski, A. Omar

P439 - Icu patients suffer from circadian rhythm desynchronisation

K. Kiss , B. Köves, V. Csernus, Z. Molnár

P440 - Noise reduction in the ICU: feasible ?

Y. Hoydonckx, S. Vanwing, B. Stessel, A. Van Assche, L. Jamaer, J. Dubois

P441 - Accidental removal of invasive devices in the critical patient into the bed-washing. does the presence of professional nurse modify his incidence?

V. Medo, R. Galvez, J. P. Miranda

P442 - Deprivation of liberty safeguards (dols): audit of compliance in a of a 16-bed specialist cancer critical care unit.

C. Stone, T. Wigmore

P443 - Use of a modified cristal score to predict futility of critical care in the elderly

Y. Arunan, A. Wheeler, K. Bauchmuller, D. Bryden

P444 - Improvement of Referral Rate to Palliative Care for Patients with Poor Prognosis in Neurosurgical Intensive Care Unit

Y. Wong, C. Poi, C. Gu

P445 - Factors associated with limitation of life supporting care (lsc) in a medico-surgical intermediate care unit, and outcome of patients with lsc limitation: a monocentric, six-month study.

P. Molmy, N. Van Grunderbeeck, O. Nigeon, M. Lemyze, D. Thevenin, J. Mallat

P446 - Palliative care consultation and intensive care unit admission request: a cohort study

J. Ramos, M. Correa, R. T. Carvalho, D. Forte

P447 - Nursing and medicine together in postsurgical intensive care unit: situations of prognostic conflict at the end of life. our critical care nurses suffer with our medical activism?

A. Fernandez, C. McBride

P448 - End of life who may decide

E. Koonthalloor, C. Walsh

P449 - Correctly diagnosing death

A. Webber, M. Ashe, K. Smith, P. Jeanrenaud

P450 - Skin procurement performed by intensive care physicians: yes, we can.

A. Marudi , S. Baroni, F. Ragusa, E. Bertellini

P451 - Death analysis in pediatric intensive care patients

E. A. Volakli , E. Chochliourou, M. Dimitriadou, A. Violaki, P. Mantzafleri, E. Samkinidou, O. Vrani, A. Arbouti, T. Varsami, M. Sdougka

P452 - The potential impact of euthanasia on organ donation: analysis of data from belgium

J. A. Bollen, T. C. Van Smaalen, W. C. De Jongh, M. M. Ten Hoopen, D. Ysebaert, L. W. Van Heurn, W. N. Van Mook

P453 - Communication within an intensive care setting

K. Sim, A. Fuller

P454 - Development and implementation of a longitudinal communication curriculum for critical care medicine fellows

A. Roze des Ordons, P. Couillard, C. Doig

P455 - Staff-family conflict in a multi-ethnic intensive care unit

R. V. Van Keer, R. D. Deschepper, A. F. Francke, L. H. Huyghens, J. B. Bilsen

P456 - Does the source of admission to critical care affect family satisfaction?

B. Nyamaizi, C. Dalrymple, A. Molokhia, A. Dobru

P457 - A simple alternative to the family satisfaction survey (fs-icu)

E. Marrinan, A. Ankuli, A. Molokhia

P458 - A study to explore the experiences of patient and family volunteers in a critical care environment: a phenomenological analysis

J. McPeake, R. Struthers, R. Crawford , H. Devine , P. Mactavish , T. Quasim

P459 - Prevalence and risk factors of anxiety and depression in relatives of burn patients.

P. Morelli, M. Degiovanangelo, F. Lemos, V. MArtinez, F. Verga, J. Cabrera, G. Burghi

P460 - Guidance of visiting children at an adult intensive care unit (icu)

A. Rutten , S. Van Ieperen, S. De Geer, M. Van Vugt, E. Der Kinderen

P461 - Visiting policies in Italian pediatric ICUs: an update

A. Giannini, G Miccinesi, T Marchesi, E Prandi

## P001 Sepsis impairs the capillary response within hypoxic capillaries and decreases erythrocyte oxygen-dependent ATP efflux

### R. M. Bateman, M. D. Sharpe, J. E. Jagger, C. G. Ellis

#### University of Western Ontario, London, Canada

**Introduction:** A hallmark of sepsis is early onset microvascular dysfunction. However, the mechanism responsible for maldistribution of capillary blood flow is not understood. Evidence suggests red blood cells (RBC) can sense local oxygen (O2) conditions and signal the vasculature, via adenosine triphosphate (ATP), to increase capillary flow. We hypothesized that sepsis impaired microvascular autoregulation over the entire capillary network, within a capillary and within the RBC. Study objectives were to: 1) measure capillary response time within hypoxic capillaries (capillaries with RBC hemoglobin oxygen saturation (SO2) < 20 %), 2) test the null hypothesis that sepsis had no effect on RBC O2-dependent ATP efflux and 3) develop a pathophysiological model.

**Methods:** Hypotensive sepsis was studied in male Sprague-Dawley rats using cecal ligation and perforation, with a 6-hour end point. Rat hindlimb skeletal muscle microcirculation was imaged using a dual wavelength spectrophotometric intravital microscopy system, and capillary RBC supply rate (SR = RBC/s), RBC SO2 and oxygen supply rate (qO2 = pLO2/s) were quantified. Arterial NOx (nitrite + nitrate) and RBC O2-dependent ATP efflux were measured using a nitric oxide (NO) analyzer and gas exchanger, respectively.

**Results:** Compared to control, sepsis increased capillary stopped flow and plasma lactate (p < 0.05). Increased plasma NOx (p < 0.001) was related to increased capillary RBC SR (p = 0.027). Analysis of 30-second capillary SR-SO2-qO2 profiles, revealed a shift towards decreased (p < 0.05) oxygen supply rates in some capillaries. Moreover, capillary response time within hypoxic capillaries (time to restore capillary RBC SO2 > 20 %) increased 3-4 fold (p < 0.05). And, consistent with impaired microvascular autoregulation, the RBC’s response to a hypoxic environment, measured as RBC O2-dependent ATP efflux, decreased by 62.5 % (p < 0.001).

**Conclusions:** Sepsis impaired microvascular autoregulation at the capillary and erythrocyte level. Impaired autoregulation was manifested by increased capillary stopped-flow, increased capillary response time within hypoxic capillaries, decreased capillary oxygen supply rate and decreased RBC O2-dependent ATP efflux. This loss of local microvascular control was partially off-set by increased capillary RBC supply rate, which correlated with increased plasma NOx.

**Reference**

Bateman RM, Sharpe MD, Jagger JE, Ellis CG: Critical care 2015, 19(1):389.

## P002 Lower serum immunoglobulin G2 level does not predispose to severe flu

### J. Solé-Violán^1^, M. López-Rodríguez^1^, E. Herrera-Ramos^1^, J. Ruíz-Hernández^1^, L. Borderías^2^, J. Horcajada^3^, N. González-Quevedo^1^, O. Rajas^4^, M. Briones^5^, F. Rodríguez de Castro^1^, C. Rodríguez Gallego^6^

#### ^1^Hospital Dr Negrín, Las Palmas de GC, Spain; ^2^Hospital San Jorge, Huesca, Spain; ^3^Hospital Universitari del Mar, Barcelona, Spain; ^4^Hospital Universitario de la Princesa, Madrid, Spain; ^5^Hospital Clínico y Universitario de Valencia, Valencia, Spain; ^6^Hospital Universitari Son Espases, Palma de Mallorca, Spain

**Introduction:** IgG2 deficiency has been suggested to predispose to severe H1N1 infection (1). However, contradictory findings have been reported (2) and, therefore, further replication of these findings in different cohorts need to be performed. The purpose of this study is to assess the immunoglobulins and IgG subclasses levels, in a cohort of patients with influenza.

**Methods:** We studied 137 Spanish patients who developed flu (80.3 % by H1N1pdm virus). Diagnosis was confirmed by the detection of Influenza virus in nasopharyngeal swabs using the Real-Time polymerase chain reaction. Immmunoglobulins and IgG subclasses (Binding Site Human IgG Subclass kits) were measured in both acute and convalescent phase and their correlation with severity was examined. We analyzed genetic variants at IGHG2 in order to evaluated their role in the IgG2 levels. Clinical and immunological characteristics were compared using the Chi-squared test or Fisher’s exact test when needed.

**Results:** Ninety-three patients were hospitalized and 49 required admission to intensive care unit. Sixty-four patients developed viral pneumonia and 26 acute respiratory distress syndrome. No differences in the serum levels of IgG, IgA, IgM or IgG subclasses were observed in the different subgroups according to severity of disease. Genotype G2m n-/n- was associated with lower serum IgG2 levels in the convalescent phase. Patients homozygous for the G2m(n-) allele had significantly lower serum IgG2 levels (256.7 +/- 121.3 mg/dL, N = 16) than individuals carrying the G2m(n-) allele (n+/n- and n-/n- genotypes, 334,5 +/- 120,4 mg/dL, N = 31) (p = 0.042). However no association of IgG2m genotypes and severity of flu was observed.

**Conclusions:** In our study we have not been able to replicate previously reported observations. IgG2 deficiency does not appear to be a significant risk factor for severity of flu.

**References**

1. Gordon C. et al. Association between severe pandemic 2009 influenza A (H1N1) virus infection and immunoglobulin G(2) subclass deficiency. Clin Infect Dis. 2010;50(5):672-8.

2. Chan JF et al. The lower serum immunoglobulin G2 level in severe cases than in mild cases of pandemic H1N1 2009 influenza is associated with cytokine dysregulation. Clin Vaccine Immunol 2011; 18:305-10.

## P003 Brain protective effects of intravenous immunoglobulin through inhibition of complement activation and apoptosis in a rat model of sepsis

### F. Esen^1^, G. Orhun^1^, P. Ergin Ozcan^1^, E. Senturk^1^, C. Ugur Yilmaz^2^, N. Orhan^3^, N. Arican^4^, M. Kaya^2^, M. Kucukerden^3^, M. Giris^3^, U. Akcan^3^, S. Bilgic Gazioglu^5^, E. Tuzun^3^

#### ^1^Istanbul University; Medical Faculty of Istanbul, Anesthesiology and Intensive Care, Istanbul, Turkey; ^2^Istanbul University; Medical Faculty of Istanbul, Physiology, Istanbul, Turkey; ^3^Istanbul University; Institute of Experimental Medicine, Neuroscience, Istanbul, Turkey; ^4^Istanbul University; Medical Faculty of Istanbul, Forensic Medicine, Istanbul, Turkey; ^5^Istanbul University; Institute of Experimental Medicine, Immunology, Istanbul, Turkey

**Introduction:** Intravenous(IV) immunoglobulin(Ig) treatment is known to alleviate behavioral deficits in the experimentally induced model of sepsis. To delineate the mechanisms by which IVIg treatment prevents neuronal dysfunction, an array of immunological and apoptosis markers was investigated.

**Methods:** Sepsis was induced by cecal ligation perforation(CLP) in rats. The animals were divided into five groups; sham, control, CLP + saline, CLP + immunoglobulin G IgG(250 mg/kg,iv), and CLP + immunoglobulins enriched with immunoglobulin M- IgGAM(250 mg/kg,iv). Blood and brain samples were taken in two sets of experiments after CLP to see the early(24 hrs) and late(10 days) effects of treatment. Total complement activity, complement 3(C3) and soluble complement C5b-9 levels were measured in sera of rats using ELISA-based methods. Cerebral complement content was analyzed by Western Blot. Immune cell infiltration and gliosis were examined by immunohistochemistry using cluster of differentiation 3, CD4, CD8, CD11b, CD19 and glial fibrillary acidic protein antibodies. Apoptotic neuronal death was investigated by TUNEL staining and Western Blot-based semi-quantitative evaluation of brain homogenates by bax and bcl-2 antibodies.

**Results:** IV IgG and IgGAM administration significantly reduced systemic complement activity but increased serum C3 and soluble C5b-9 levels. Likewise, Western Blot data showed slightly increased C5b-9 expression and significantly reduced C1q expression in brain samples of IgGAM-treated but not IgG-treated septic rats especially in the first day of administration. No cerebral cellular infiltrates were observed in treated and non-treated septic rats. By contrast, IV IgG and IgGAM treatment induced considerable amelioration in glial cell proliferation which was increased in non-treated rats. IgG and IgGAM treated rats exhibited significantly reduced numbers of apoptotic neurons and cerebral expression levels of bax and bcl-2 as compared to non-treated rats.

**Conclusions:** We suggest that IV IgG and IgGAM administration ameliorates neuronal dysfunction and behavioral deficits by reducing apoptotic cell death and glial cell proliferation. IgGAM treatment might be suppressing classical complement pathway by reducing C1q expression.

## P004 Adenosine a1 receptor dysfunction is associated with leukopenia: A possible mechanism for sepsis-induced leukopenia

### R. Riff^1^, O. Naamani^1^, A. Douvdevani^2^

#### ^1^Ben-Gurion University of the Negev, Beer-Sheva, Israel; ^2^Soroka Medical Center, Beer-Sheva, Israel

**Introduction:** Most patients survive the initial hyper-inflammatory phase of severe sepsis and reach an intensive care unit with immunosuppression. Adenosine, a potent modulator of in fl;ammation and immunity is strongly elevated in blood of septic patients. We have previously shown that adenosine A1 receptor (A1R, Gi receptor) dominates the pro-inflammatory phase of bacterial peritonitis and that activation of this receptor by a specific agonist induces A2AR (Gs) expression and dominance during the resolution phase of inflammation. In this study we aimed to elucidate the role of adenosine and its receptors in sepsis-associated leukopenia. We hypothesized that elevated adenosine levels in sepsis affects the normal development of lymphocytes through down-regulation of A1R.

**Methods:** Polymicrobial severe sepsis was induced in C57BL/6 mice by cecal ligation and puncture (CLP) with an 18G needle. Blood and bone marrow (BM) cell counts, as well as flow cytometry were performed at 24 h post sepsis induction.

**Results:** CLP-treated mice exhibited significantly lower number of WBC compared to sham controls (9.15 ± 2.65 vs. 3.21 ± 1.58 cells x10^3/μl). Sham A1R-/- mice showed lower WBC counts compared to sham WT littermate (4.5 ± 3.24 cells x10^3/μl, p < .05). No significant difference was observed in WBC count between the sham A1R-/- and CLP WT groups. Similarly, desensitization of A1R by agonist (CCPA) or elimination of A1R with A1R antagonist (DCPCX) were associated with leukopenia. The T-cell was the main cell population affected by CLP and A1R elimination. Apoptotic rate of nucleated BM cells in sham A1R-/- mice was similar to the rate observed in WT CLP mice, and almost 2-fold greater than the early apoptosis rate (Annexin V+, 7-ADD-) shown in WT sham group (3.64 ± 1.23 % vs. 1.86 ± 0.59 %, respectively, p < .05). Interestingly, CLP-A1R-/- mice were shown to produce less interleukin (IL)-15 in lavage fluid compared to CLP-WT mice (8.8 ± 6.0 vs. 35.3 ± 9.8 pg/ml, respectively p < .001).

**Conclusions:** The similarities in the phenotype induced by sepsis and suppression of A1R support our hypothesis that dysfunction/down-regulation of the Gi-A1R at the onset of sepsis changes the effect of adenosine towards Gs-A2AR/A2BR-mediated leukopenia and immune paralysis. Suppression of IL-15 might be a part of the mechanism of leukopenia observed in CLP-treated mice and A1R-/- mice.

## P005 Analysis of neutrophil by hyper spectral imaging - A preliminary report

### R. Takegawa^1^, H. Yoshida^1^, T. Hirose^1^, N. Yamamoto^1^, H. Hagiya^1^, M. Ojima^1^, Y. Akeda^1^, O. Tasaki^2^, K. Tomono^1^, T. Shimazu^1^

#### ^1^Osaka University Graduate School of Medicine, Suita, Japan; ^2^Nagasaki University Graduate School of Biomedical Sciences, Nagasaki, Japan

**Introduction:** Neutrophil play an important role as the first line of innate immune defense. Activated neutrophils become segmented and cytoplasm grows larger including granules. However, sometimes it is difficult to diffentiate whether the segmented neutrophils are on the process to grow up or not, and to decide to stop antibiotics or not. There are some reports that hyper spectral imaging (HSI) is available for analysis including diagnosis of malaria infected cells and distinction of the cells to produce antibody. In this study, we evaluated serial changes of HSI spectrum in patients with infection, and examined whether we could distinguish activated neutrophils or not.

**Methods:** Sputum and urine samples were collected from the clinically ill three patients with pulmonary infection(n = 2) and urinary tract infection(UTI; n = 1). These samples were smeared on glass slides and gram stain was conducted. We observed the slides with HSI to get the hyper spectral data 10 times from each cytoplasm of neutrophil, and analyzed hyper spectral data with the software system, MultiSpec®.

**Fig. 1 (Abstract P005).**
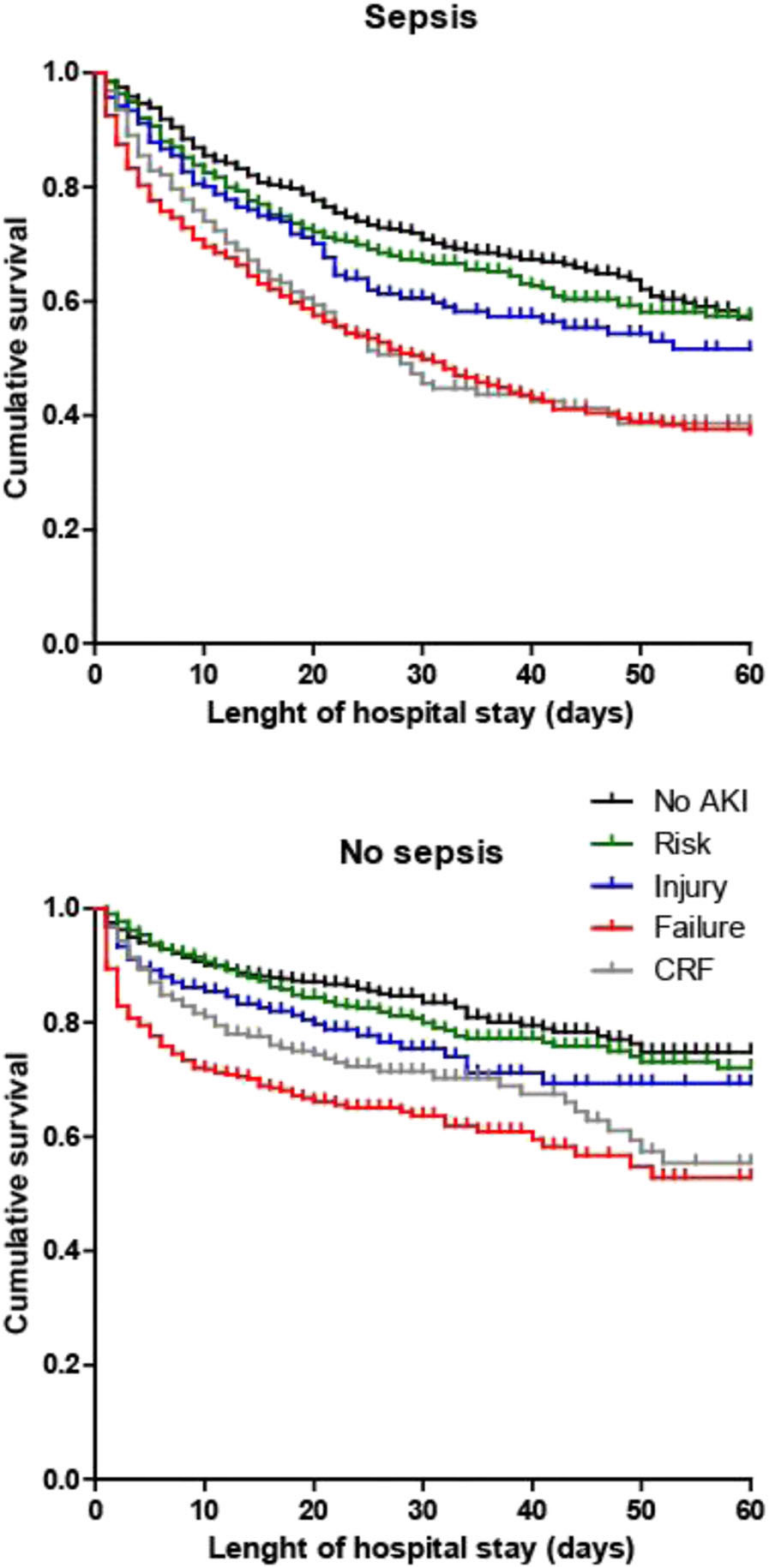
ᅟ

**Results:** We could find that neutrophils have different spectrum in each infectious stage, especially during from 400 to 700 nm wavelength. Representative image of a patient with UTI was shown in Fig. 1. When the neutrophils became segmented and had phagocytosis, the cytoplasm of neutrophil enlarged and showed strong intensity (black lines) compared to when microorganism disappeared and the size of cytoplasm decreased (blue and red lines).

**Conclusions:** We could find the serial changes of spectrum of neutrophils during each infectious stage. HSI may be available to decide start or termination of antibiotics. Further study is required to establish the cut off intensity level of the activated neutrophils.

## P006 Chemiluminescent intensity assessed by eaa predicts the incidence of postoperative infectious complications following gastrointestinal surgery

### S. Ono^1^, T. Kubo^2^, S. Suda^1^, T. Ueno^1^, T. Ikeda^1^

#### ^1^Tokyo Medical University Hachioji Medical Center, Hachioji, Tokyo, Japan; ^2^National Defense Medical College, Tokorozawa, Saitama, Japan

**Introduction:** Recent studies have demonstrated that the endotoxin activity levels, which were analyzed using the Endotoxin Activity Assay (EAA), correlated with the severity of sepsis in patients admitted to the ICU. On the other hand, there are several reports that they dispute the clinical utility of the EAA. We focused on chemiluminescent intensity (CI) in response to lipopolysaccharide (LPS) by EAA and evaluated the predictive value for the incidence of postoperative infectious complications following elective gastroenterological surgery.

**Methods:** Forty eight patients who underwent elective surgery for gastrointestinal cancer were enrolled in this study. Blood samples were taken on the previous day (before surgery), and on the first (POD1) and third postoperative days (POD3). All blood samples were analyzed using the EAA system. We focused on CI max, which is maximally stimulated with LPS and the minimum CI, which is only stimulated with opsonized zymosan.

**Results:** Postoperative infectious complications occurred in 23 of the 48 patients. There were significant differences in the EA levels between patients who developed postoperative infectious complications and patients who did not on POD3. Minimum CI was significantly higher in the patients who developed postoperative infectious complications at all points compared to those from the patients who did not. In addition, the ratio of maximal CI to minimum CI, which reflects the neutrophil function against maximal LPS, was significantly lower before surgery and on POD1 in patients who developed postoperative infectious complications. Although a significant positive correlation was observed between the neutrophil counts and maximal CI or minimum CI, no correlation was observed between the neutrophil counts and the ratio of maximal CI to minimum CI.

**Conclusions:** Chemiluminescent intensities, especially the ratio of maximal CI to minimum CI, could be an early predictive marker for the development of postoperative infectious complications.

## P007 Serial change of c1 inhibitor in patients with sepsis – A prospective observational study

### T. Hirose, H Ogura, H Takahashi, M Ojima, J Kang, Y Nakamura, T Kojima, T Shimazu

#### Osaka University Graduate School of Medicine, Suita, Japan

**Introduction:** C1 inhibitor (C1INH), belonging to the superfamily of serin protease inhibitors, regulates not only complement system, but also plasma kallikrein-kinin system, fibrinolytic system and coagulation system. The biologic activities of C1INH can be divided into the regulation of vascular permeability and anti-inflammatory functions. The objective of this study was to clarify the serial change in C1INH in patients with sepsis and evaluate the impact of C1INH on their clinical course.

**Methods:** This study was a single center prospective observational study. We serially examined C1INH activity values (normal range 70-130 %) in patients with sepsis admitted into the intensive care unit of the Trauma and Acute Critical Care Center at Osaka University Hospital (Osaka, Japan) during the period between January 2014 and August 2015. We defined refractory shock as septic shock unresponsive to the conventional therapy such as adequate fluid resuscitation and vasopressor therapy to maintain hemodynamics.

**Results:** The serial change of C1INH was evaluated in 40 patients with sepsis (30 male and 10 female; 30 survivors and 10 non-survivor; mean age, 70+/-13.5 years). We divided patients into three groups such as (i) non-shock group (n = 14), (ii) non-refractory shock group (n = 13), (iii) refractory shock group (n = 13, survivors; n = 3, non survivors; n = 10). In non-shock group, C1INH were 107.3+/-26.5 % at admission and 104.2+/-22.3 % at day1, and it increased after day1 (128.1+/-26.4 % at day3, 138.3+/-21.2 % at day 7, 140.3+/-12.5 % at day 14)(p = 0.0040). In non-refractory shock group, C1INH were 113.9+/-19.2 % at admission and it increased after admission (120.2+/-23.0 % at day1, 135.7+/-19.9 % at day3, 138.8+/-17.2 % at day 7, 137.7+/-10.7 % at day 14)(p = 0.0029). In refractory shock group, C1INH were 96.7+/-15.9 % at admission and 88.9+/-22.3 % at day1 and it increased after day1 (119.8+/-39.6 % at day3, 144.4+/-21.1 % at day 7, 140.5+/-24.5 % at day 14)(p < 0.0001). The difference between these three groups was statistically significant (p = 0.0039). C1INH in non-survivors did not increase significantly during their clinical course (p = 0.0773).

**Conclusions:** In refractory shock patients with sepsis, the values of C1INH were low (especially in non survivors) at admission and day 1. The validity of C1INH replacement therapy in patients with septic shock may lead to a new strategy for management in sepsis.

## P008 Comparison of bacteremia and sepsis on sepsis related biomarkers

### T. Ikeda, S. Suda, Y. Izutani, T. Ueno, S. Ono

#### Hachiouji medical center, Tokyo medical university, Tokyo, Japan

**Introduction:** When septic patients progress to endotoxin shock, they become subject to high mortality rate. The mortality rate of septic patients with multiple organ failure has been reported to be 30-80 %. Recently, much sepsis related biomarkers have been measured for diagnosis of sepsis.

**Methods:** We would like to clarify the difference of patient fs background and various biomarkers (Procalcitonin:, Presepsin., Interleukin-6, Endotoxin activity assay:EAA,Angiopoietin-2) and 28-days mortality rate in sepsis and bacteremia. Patients were classified as a group in which blood culture was positive (BC-positive group: N = 31) or a group in which blood culture was negative (BC-negative group: N = 31).Blood cultures (blood samples were drown from different two sites) were performed immediately after ICU admission due to sepsis or septic shock. Results were expressed as mean+/-SD (median). Statistical analysis was used with Mann-Whitney U-test and Chi square test or Fisher’s test.

**Results:** Procalcitonin of BC-positive and BC-negative patients were 35.9}75.3 (7.8) and 17.1}30.9 (2.0) respectively. There are no significant differences between the patients. Presepsin of BC-positive and BC-negative patients were 2726}3208 i2147 jand 2733}2558 i2281 jrespectively, also, no significant difference between the groups.

EAA level of BC-positive patients was 0.54}0.20 (0.58), while that in BC-negative patients was 0.37}0.26 (0.36), and the difference between the groups was statistically significant (p < 0.05). Angiopoietin-2 of BC-positive and BC-negative patients were 16943}22793 i12000 jand 7457}7068 i5080 jrespectively. There is also a significant difference (p < 0.05) between the groups. IL-6 of BC-positive and BC-negative patients were 2988}10602 i212 jand 516}1703 i107 jrespectively, but, no difference between the groups. 28-days survival rate was 90.9 % in BC-positive patient and 87.1 % in BC- negative patient. There were no significant data in the two groups.

**Conclusions:** The values of EAA and Angiopooitin-2 of BC-positive patients were significant higher than BC-negative patients. But, no significant differences on 28-days mortality between the groups were existed. These results showed discrepancy to our previous report1). We shold be more evaluating about the patient’s basic disease.

**Reference**

1) T. Ikeda, K. Ikeda, S. Suda, et al.: Usefulness of the endotoxin activity assay as a biomarker to assess the severity of endotoxemia in critically ill patients. Innate immunity, 2014, DOI: 10.1177/1753425913516885

## P009 The changes of procalcitonin levels in critical patients with abdominal septic shock during blood purification

### T. Taniguchi^1^, M. Okajima^2^

#### ^1^Kanazawa University, Kanazawa, Japan; ^2^Kanazawa University Hospital, Kanazawa, Japan

**Introduction:** Procalcitonin (PCT) is an early, sensitive and accurate marker for sepsis. Especially, several studies indicated that PCT is an important indicator to diagnose infection, predict outcomes or guide treatment of abdominal sepsis. Recently, blood purification (BF) such as endotoxin absorption therapy (polymyxin B [PMX] hemoperfusion) and continuous renal replacement therapy (CRRT) has been carried out for abdominal septic shock. However, there are few studies about the changes of PCT levels in abdominal septic shock during BF. Therefore, we retrospectively evaluated the changes of PCT levels in critical patients with abdominal septic shock during BF.

**Methods:** Twenty-four patients (M/F 17/7, mean age 61 years) with abdominal septic shock underwent BF (PMX and CRRT). PMX was undergone twice and CRRT was undergone for 5 days. The changes of PCT levels and other inflammatory mediators such as blood WBC counts, CRP levels and lactate levels during BF were measured for 5 days. Moreover, SOFA and survival rate 14 days after ICU admitted were measured.

**Results:** Hemodynamics in all patients improved at 8 hrs after BF. The PCT levels were improved (66.0 to 12.7 ng/mL; p < 0.05), but WBC counts (8,420 to 10,380 /uL) and CRP levels (9.3 to 9.0 mg/dL) were not improved 5 days after BF. SOFA scores decreased (12.5 to 7.0; p < 0.05) at 5 days after BF. All patients recovered and discharged in ICU. There was significantly correlation between PCT and SOFA scores (Y = 9.04 + 0.02X; R2 = 0.112, p < 0.0001), but there were no correlation between PCT and other mediators.

**Conclusions:** In the present study, the PCT levels in critical patients with abdominal septic shock improved after BF and there was significantly correlation between PCT and SOFA scores.

**Fig. 2 (Abstract P009):**
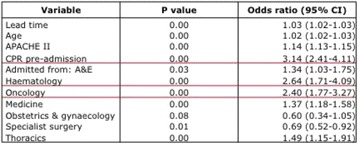
ᅟ

## P010 Validation of a new sensitive point of care device for rapid measurement of procalcitonin

### C. Dinter ^1^, J. Lotz^2^, B. Eilers^3^, C. Wissmann^1^, R. Lott^2^

#### ^1^ThermoFisher, Hennigsdorf, Germany; ^2^Institut für Klinische Chemie und Laboratoriumsmedizin, Mainz, Germany; ^3^MVZ Labor Limbach Gruppe, Berlin, Germany

**Introduction:** Procalcitonin (PCT), a highly sensitive and specific biomarker for bacterial infections, is increasingly being used in the ED and ICU for the diagnostic work up of patients with LRTI and sepsis. Recently, Samsung IB BRAHMS PCT, a new point of care (POC) test, has been developed for a fast PCT measurement of whole blood and EDTA samples with a measuring range of 0.08 - 10 μg/L. The objective of this evaluation study was to determine the correlation of the Samsung IB BRAHMS PCT test to established reference methods.

**Methods:** This study was conducted to examine the correlation of the Samsung IB BRAHMS PCT to the established reference methods BRAHMS PCT sensitive Kryptor and BRAHMS PCT Elecsys at two German Laboratories. The design was based on the related CLSI Guidelines EP09-A3 (Measurement Procedure Comparison and Bias Estimation Using Patient Samples). The study compared PCT values using EDTA plasma determined with reference methods with results determined with Samsung IB BRAHMS PCT using EDTA whole blood and EDTA plasma over the whole measuring range of the assay in patients undergoing routine care PCT measurement.

**Results:** 256 patients were included over a 6 weeks period. The correlation between EDTA whole blood or plasma and the reference method was r- = 0.97, the correlation of plasma and whole blood samples, both determined on the Samsung analyzer, was r = 0,98. The clinical concordance was 94 % related to a 0.5 μg/L threshold in whole blood. No significant bias was observed (0.033 and 0.034 for venous whole blood and plasma respectively, compared to the reference method). No difference was observed comparing imprecision of whole blood and plasma. The total CV was < 14.5 % for concentrations between 0.45 and 4.54 μg/L. The time to result of the Samsung IB BRAHMS PCT was 20 min.

**Conclusions:** This study found an excellent correlation and diagnostic accuracy of the new, sensitive POC device for rapid measurement of procalcitonin. The Samsung IB BRAHMS PCT test allows an accurate measurement of PCT at a point of care, where near patient testing is practical, that is comparable to established lab based BRAHMS PCT assays.

## P011 Infection biomarkers in primary care patients with acute respiratory tract infections – Comparison of procalcitonin and C-reactive protein

### M. M. Meili, P. S. Schuetz

#### Kantonsspital Aarau, Aarau, Switzerland

**Introduction:** There is a lack of studies comparing the utility of C-reactive protein (CRP) with procalcitonin (PCT) for the management of patients with acute respiratory tract infections (ARI) in primary care. Our aim was to study first the correlation between these markers, and second to compare their predictive accuracy in regard to clinical outcome prediction.

**Methods:** This is a secondary analysis using clinical and biomarker data of 458 primary care patients with pneumonic and non-pneumonic ARI. We used correlation statistics (spearman’s rank test) and multivariable regression models to assess association of markers with adverse outcome, namely days with restricted activities and ongoing discomfort at day 14.

**Results:** At baseline, CRP and PCT did not correlate well in the overall population (r2 = 0.16 and r2 = 0.04) and particularly in the subgroup of patients with non-pneumonic ARI. Low correlations were also found comparing cut-off ranges, day seven levels and biomarker changes from baseline to day seven. High admission levels of CRP (>100 mg/dL, regression coefficient 1.7, 95%CI 0.6 to 2.8) as well as PCT (>0.5ug/L regression coefficient 2.3, 95%CI 0.3 to 4.3) were significantly associated with days with restricted activities. There were no associations of both markers regarding ongoing discomfort at day 14.

**Conclusions:** CRP and PCT levels do not well correlate and have both have moderate prognostic accuracy in primary care patients with ARI to predict clinical outcomes. The low correlation between both markers calls for interventional research comparing these markers head to head in regard to their ability to guide antibiotic decisions.

## P012 Do we need a lower procalcitonin cut off?

### H. Hawa, M. Sharshir, M. Aburageila, N. Salahuddin

#### King Faisal Specialist Hospital & Research Center, Riyadh, Saudi Arabia

**Introduction:** Procalcitonin (PCT) has been proposed as a helpful tool to guide treatment with antibiotics and to improve antibiotics stewardship. [1,2,3] However the cut off for the PCT level has not been agreed as different studies have suggested different thresholds to predict the need for antibiotics, these cut off points ranged from 0.25 ng/mL – 1 ng/mL. In our hospital the reference range is set at 0.5 ng/mL. This study aimed at identifying the best PCT level to rule out sepsis.

**Methods:** We have retrospectively reviewed 53 intensive care unit patients, who had serial PCT tests as part of their daily blood tests. Three serial readings of PCT were collected in addition to microbiology culture results and whether the patient met the definition criteria for sepsis as set out in the 2001 International Sepsis Definition conference.

**Results:** Out of the 53 patients, 26 (49.05 %) patients had negative microbiological cultures with no evidence of sepsis.

**Fig. 3 (Abstract P012).**
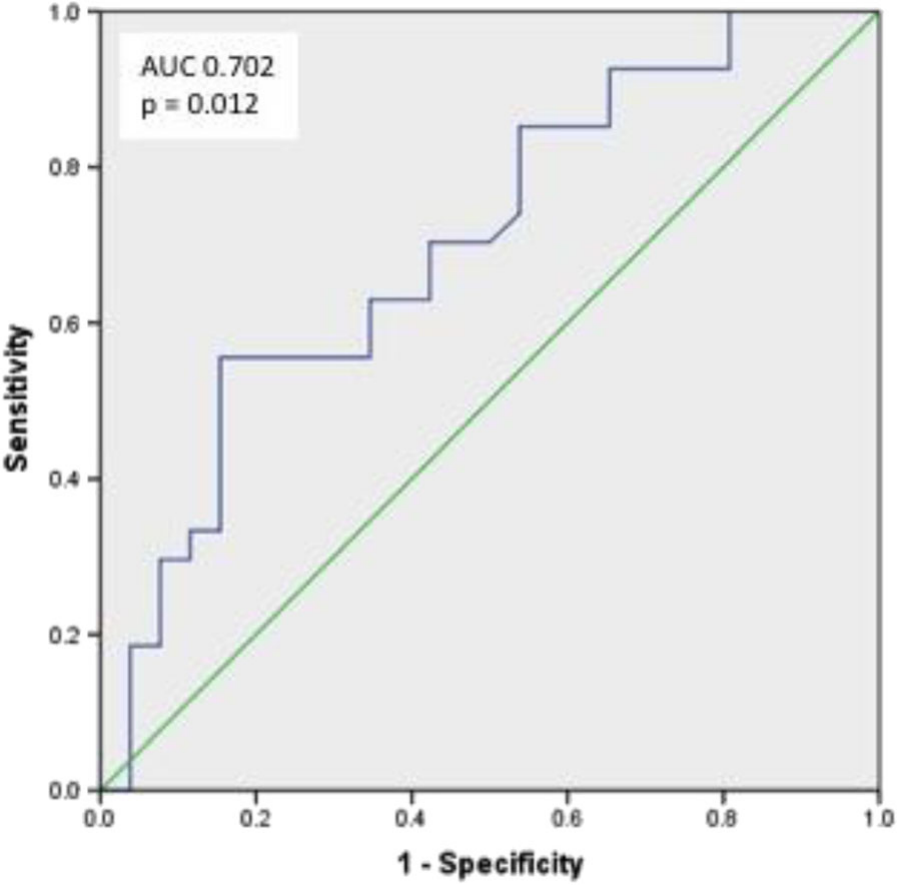
ᅟ

PCT test level of 0.13 ng/mL had 100 % sensitivity as no patient below this cut off had evidence of sepsis or positive microbiology culture. The Area under the ROC was 0.702 with 95 % Confidence interval of 0.56-0.84 and a p value of 0.012 (Fig. 3).

**Conclusions:** Using lower PCT cut off would further enhance the ability of PCT to rule out sepsis in the critically ill patient.

**References**

1. Bouadma et al, Lancet. 2010 Feb 6;375(9713):463-74.

2. Burkhardt et al, Eur Respir J. 2010 Sep;36(3):601-7.

3. Hochreiter M, Critical care, 2009; 13(3): R83.

## P013 The predictive role of C-reactive protein and procalcitonin biomarkers in central nervous system infections with extensively drug resistant bacteria

### V. Chantziara, S. Georgiou, A. Tsimogianni, P. Alexandropoulos, A. Vassi, F. Lagiou, M. Valta, G. Micha, E. Chinou, G. Michaloudis

#### Saint Savvas Hospital, Athens, Greece

**Introduction:** Central Nervous System (CNS) infections after neurosurgical procedures with extensively drug resistant bacteria (XDR), in intensive care unit (ICU) patients, is a life threatening complication requiring early identification and immediate action. Increased number of white blood cells (WBC), high temperature and circulating acute phase proteins are common in those patients making timely diagnosis challenging. We sought to investigate the reliability of serum C-Reactive protein (CRP) and procalcitonin (PCT) to early identify and monitor CNS infections.

**Methods:** From January 2013 to November 2015 all cases with CNS infections were recorded. Inclusion criteria were the presence of fever > 38.5oC and compatible lumbar puncture findings (increased number of polymorphonuclear leukocytes, increased protein and low glucose compared to serum levels). The WBC, CRP and PCT levels before (when the inclusion criteria were met) and after the infection (last intrathecal administration of Collistin 300.000 IU, Amikacin 25 mg and Vancomycin 25 mg) were compared.

**Results:** Twelve patients (mean age 45.7 ± 18.1) were studied. WBC remained high before and after infection. Conversely, CRP values were statistically different before and after the infection whereas the PCT ones were not (Table 1).

**Conclusions:** Based on our findings CRP carries a better predictive value than PCT as biomarker for early diagnosis and monitoring of CNS infections in ICU. The small number of patients is a limitation for these results.

**Table 1 (Abstract P013).** ᅟ

	Before CNS infection	After CNS infection	P value
WBC	13000 ± 6500	9700 ± 4200	0,12
CRP	18 ± 9.2	7.7 ± 4.7	0,06
PCT	13.4 ± 9.3	9 ± 2.2	0,2

## P014 Changes in endotoxin activity assay and procalcitonin levels after direct hemoperfusion with polymyxin-b immobilized fiber

### A. Kodaira^1^, T. Ikeda^2^, S. Ono^2^, T. Ueno^2^, S. Suda^2^, Y. Izutani^2^, H. Imaizumi^1^

#### ^1^Tokyo Medical University, Tokyo, Japan; ^2^Tokyo Medical University, Hachioji Medical Center, Tokyo, Japan

**Introduction:** This study aimed to clarify changes in the endotoxin activity assay( EAA )and (PCT) levels before and after direct hemoperfusion with polymyxin-B immobilized fiber(PMX-DHP).

**Methods:** We studied 212 patients [survival (S) group: 146; non-survival (NS) group: 66] in whom PMX-DHP was performed for severe sepsis and septic shock and who were admitted to the ICU. At the same time, blood biochemistry and interleukin-6 (IL-6) levels were measured before and after PMX-DHP. A double-lumen catheter was inserted into the patient fs femoral vein, and PMX-DHP was performed using a blood pump to remove endotoxins. The blood access for hemoperfusion consisted of a venous-to-venous system. Nafamostat mesilate was administered as a 40 mg bolus followed by 30-40 mg/hour continuous intravenous infusion as an anticoagulant. Extracorporeal circulation was performed for 2 hours. Results were expressed as mean SD (median). The Mann-Whitney U-test and chi-square test or Fisher fs test were used for statistical analysis.

**Results:** Regarding background factors, the APACHE2 scores in the S-group and NS-group were 23.9 } 8.0 and 32.8 } 9.0, and the SOFA scores were 10.0 } 9.0 and 12.9 } 3.4, respectively. The blood pressure and PaO2/FIO2 ratio markedly improved immediately after PMX-DHP. The IL-6 levels decreased immediately after PMX-DHP in the S-group, but the levels showed no significant difference between the groups before PMX-DHP. The PCT levels before PMX-DHP were 59.7 } 95.2 (24.7) in the S-group and 45.3 } 54.8 (24.0) in the NS-group. Immediately after PMX-DHP, these levels became 48.6 } 69.4 (25.5) in the S-group and 51.9 } 67.5 (19.9) in the NS-group, respectively. The EAA levels before PMX-DHP were 0.50 } 0.22 (0.46) in the S-group and 0.59 } 0.26 (0.59) in the NS-group. Immediately after PMX-DHP, these levels became 0.50 } 0.21 (0.53) in the S-group and 0.53 } 0.27 in the NS-group, respectively. These values showed no significant differences before and after PMX-DHP.

**Conclusions:** This study showed that the PCT and EAA levels had no significant changes after PMX-DHP.

**Reference**

Toshiaki Ikeda, Kazumi Ikeda, Shingo Suda, et al.: Usefulness of the endotoxin activity assay as a biomarker to assess the severity of endotoxemia in critically ill patients. Innate Immun. 2014; 20(8):881-7. doi: 10.1177/1753425913516885. Epub 2014 Jan 7.

## P015 Diagnostic usefullness of combination biomarkers on ICU admission

### M. V. De la Torre-Prados, A. Garcia-De la Torre, A. Enguix-Armada, A. Puerto-Morlan, V. Perez-Valero, A. Garcia-Alcantara

#### Hospital Virgen de la Victoria, Málaga, Spain

**Introduction:** The purpose of the study was to determine the accuracy of CRP, PCT and presepsin (PSP) and the combination of them, measured in the first 24 h after admission for diagnosis of severe sepsis( SS) and septic shock (SSh).

**Methods:** Case/control study admitted in the Intensive Care Unit, during 12 months. Criteria of SS or SSh was according to the Surviving Sepsis Campaign and controls were enrolled in the Coronary Unit.

Biomarkers were measured within the first 24 h after admission. The accuracy for diagnosis was determined using receiver operating characteristic (ROC) and logistic regression was performed to assess the combination of studied biomarkers could improve the diagnostic value of each one separately.

**Results:** We analyzed 388 whom 142 were controls. 61.8 % of patients were males, mean age: 63 years, interquartile range (IQR) (18–90) with APACHE II of 26 (21-29) and SOFA of 9 (7-11); and in controls 67.8 % were men, mean age: 63 years, IQR (39–91). The main sources of infection were respiratory tract 47.8 % and intra-abdomen 19.8 %.

AUC, cutoff value, sensitivity and specificity, positive predictive value (PPV) and negative predictive value (NPV) of the biomarkers used in the study are shown Table 2.

Comparisons between AUCs were significantly different (p <0.01) in all cases except for PCT versus PCT + PSP (p < 0.163), however, the logistic regression showed that the combination of PCT and PSP improved the diagnostic value of each one separately.

**Conclusions:** PCT and PSP levels measured on admission showed high diagnosis efficacy to predict SS and SSh. Using both markers simultaneously increases diagnostic accuracy in septic patients, improving the classification of patients.

**Table 2 (Abstract P015).** Diagnostic Biomarkers in Severe Sepsis and Septic Shock

Parameters	AUC;CI 95 %	Cut-off	Sens;CI95%
PCT, μg/L	0.989(0.96–0.998)	0.28	92(88.6–95.8)
PSP, pg/mL	0.948(0.9–0.98)	101.6	82(73.7–81.9)
PCT + PSP	0.998(0.973-1)		98(93.7-99.7)

## P016 Platelet function analysis utilising the PFA-100 does not predict infection, bacteraemia, sepsis or outcome in critically ill patients

### N. Bolton, J. Dudziak, S. Bonney, A. Tridente, P. Nee

#### ^1^St Helens and Knowsley NHS Trust, Merseyside, UK

**Introduction:** Sepsis is known to be associated with impaired platelet function (1). The PFA-100 (Siemens Healthcare, Dade International, Miami, Fla., USA) is a device in which a citrated whole blood sample is aspirated through an aperture coated with agonists inducing platelet activation. Platelet aggregation leads to occlusion of the aperture and blood flow ceases over an interval termed the closure time (CT).

Aim:

The aim of the present study was to determine whether CT, measured by the PFA-100, could be used to determine the likelihood of infection, sepsis, and bacteraemia or hospital mortality.

**Methods:** A single centre, prospective, observational cohort study. Subjects were 101 pyrexial patients in a mixed adult ICU. Blood samples were drawn for cultures, full blood count, CRP and PFA analysis. Admission diagnosis, demographic data, laboratory variables, APACHE II scores and outcome data (infection, sepsis, bacteraemia and mortality) were collected from the clinical records. Logistic regression analysis was undertaken using Stata/IC (StataCorp, 4905 Lakeway Drive, College Station, Texas 77845 USA)

**Results:** Median age was 60 (Interquartile Range = IQR 46-71), 57 patients (56.4 %) were male, median APACHE II score was 16 (IQR 10-19). Of the recruited patients, 78 (77.2 %) had an infective process, and 70 (69.3 %) met SIRS criteria for sepsis; 12 patients (11.9 %) had positive blood cultures, 19 patients (18.8 %) did not survive to hospital discharge.

Logistic regression analysis, adjusted for age, gender and baseline APACHE II score found that CT did not predict any of the outcomes. Odd Ratios were 0.99 (95 % confidence interval = 95 % CI 0.98-1, p = 0.2) for infection, 0.99 (95 % CI 0.98-1, p = 0.37) for sepsis, 0.99 (95 % CI 0.98-1, p = 0.64) for bacteraemia and 1.00 (95 % CI 0.99-1.01, p = 0.71) for mortality, respectively.

**Conclusions:** Platelet aggregation measured by closure time utilising the PFA-100 system did not correlate with infection, sepsis, bacteraemia or mortality in our sample of febrile patients.

**Reference**

(1) Katz JN, Kolappa KP, Becker RC. Beyond thrombosis: the versatile platelet in critical illness. Chest 2011;139:658–668.

## P017 Extracellular histone H3 levels are inversely correlated with antithrombin levels and platelet counts and are associated with mortality in sepsis patients

### G. Nicolaes^1^, M. Wiewel^2^, M. Schultz^3^, K. Wildhagen^1^, J. Horn^3^, R. Schrijver^1^, T. Van der Poll^2^, C. Reutelingsperger^1^

#### ^1^Maastricht University, Maastricht, Netherlands; ^2^Center for Experimental and Molecular medicine, Amsterdam, Netherlands;^3^Academic Medical Center, Amsterdam, Netherlands

**Introduction:** Extracellular histones are cytotoxic compounds capable of mediating death in murine sepsis. Furthermore, circulating nucleosome levels predict mortality in human inflammation and sepsis. Whether or not extracellular histone H3 correlates with other plasma parameters and/or ICU scoring systems has not been completely established, nor if levels of circulating extracellular histones can be used as predictive markers for clinical outcome in sepsis.

**Methods:** Histone H3 (H3) plasma levels of 43 sepsis patients who were admitted to the Intensive Care Unit were measured and we determined the correlation of H3 levels with disease severity, organ failure, mortality and coagulation- and tissue homeostasis parameters including LDH levels, thrombin potential (ETP), prothrombin levels, antithrombin levels and platelet counts.

**Results:** For sepsis patients at the ICU we found that median H3 levels were significantly increased in non-survivors as compared to survivors with levels found being 3.15 μ g/ml versus 0.57 μg/ml respectively, P = 0.04. H3 levels are positively correlated with lactate dehydrogenase (LDH) activity (Spearman’s rho = 0.49, P < 0.001), and negatively correlated with antithrombin levels (rho = -0.34, P = 0.027) and platelet counts (rho = -0.33, P = 0.031).

**Conclusions:** We conclude that circulating H3 levels correlate with mortality in sepsis patients and inversely correlate with antithrombin levels and platelet counts.

## P018 Il-8: is this a more reliable biomarker for sepsis severity than CRP, Procalcitonin, E-selectin, IL-6 and TNF-[alpha]

### S. Pillai, G. Davies, G. Mills, R. Aubrey, K. Morris, P. Williams, P. Evans

#### NISCHR Haemostasis Biomarker Research Unit, Swansea, UK

**Introduction:** Sepsis is a systemic inflammatory response syndrome (SIRS) with an evidence of infection. Numerous inflammatory markers have been studied to assess the severity of sepsis. However, current biomarkers are insufficient to accurately determine stage and prognosis [1]. This study aimed to assess the cytokine Interleukin-8 (IL-8) for determining the stage and severity of sepsis. A comparison was made with C-reactive protein (CRP), Procalcitonin (PCT), E-selectin, Interleukin-6 (IL-6) and Tumour Necrosis Factor-α (TNF-α ).

**Methods:** This study had full ethical approval from the South West Wales Research Ethics Committee. Patients with a diagnosis of sepsis were recruited from the Emergency Department and Intensive Care Unit of a large teaching hospital in South Wales. Blood samples were taken to determine IL-8, CRP, PCT, E-selectin, IL-6 and TNF-α.

**Results:** 65 patients were included in the study: 40 with sepsis, 13 with severe sepsis and 12 with septic shock. PCT concentration was significantly increased in subjects with septic shock when compared to sepsis (Table 3,*p = 0.001, Kruskal-Wallis test). IL-8 concentration was significantly increased in septic shock compared to both sepsis and severe sepsis groups (Table 3, **p < 0.001 and p = 0.013 respectively, Kruskal-Wallis test). Using ROC analysis, IL-8 was found to be a significant predictor of mortality (AUC 0.92, p < 0.001).

**Conclusions:** Both IL-8 and PCT significantly distinguished septic shock from sepsis and severe sepsis. IL-8 was shown to be a promising inflammatory marker for assessing the severity and prognostication in patients across the sepsis spectrum.

**Reference**

1. Pierrakos C & Vincent JL. Crit Care. 2010;14:R15.

**Table 3 (Abstract P018).** Inflammatory markers in different sepsis groups

	Sepsis (n = 40)	Severe Sepsis (n = 13)	Septic Shock (n = 12)
IL-8 (pg/mL)	61 (21, 176)	71 (41, 104)	526 (136, 7998)**
CRP (mg/dL)	79 (40, 222)	193 (124, 422)	128 (21, 179)
PCT (pg/mL)	308 (108, 1038)	702 (385, 1637)	1258 (230, 17296)*

*p = 0.001, **p < 0.001

## P019 Relation between adrenomedullin and short-term outcome in ICU patients: Results from the frog ICU study

### E. G. Gayat^1^, J. Struck^2^, A. Cariou^3^, N. Deye^1^, B. Guidet^4^, S. Jabert^5^, J. Launay^1^, M. Legrand^6^, M. Léone^7^, M. Resche-Rigon^6^, E. Vicaut^1^, A. Vieillard-Baron^8^, A. Mebazaa^1^

#### ^1^Hôpital Lariboisière, Paris, France; ^2^Sphingotec, Berlin, Germany; ^3^Hôpital Cochin, Paris, France; ^4^Hôpital Saint-Antoine, Paris, France; ^5^CHRU de Montpellier, Montpellier, France; ^6^Hôpital Saint-Louis, Paris, France; ^7^AP-HM, Marseille, France; ^8^Hôpital Ambroise Paré, Paris, France

**Introduction:** Adrenomedullin (ADM) is a peptide with 52 amino acids which has strong vasodilator activity. Elevated plasma ADM levels have been detected in a wide variety of physiological and pathological conditions, with the highest elevations observed in septic shock. Marino et al. demonstrated a strong association of admission ADM levels with the severity of sepsis, supporting an earlier report describing elevated ADM levels in those with severe sepsis and those with septic shock [1]. By using a novel assay specific for bioactive ADM (bio-ADM) as Marino et al.the aim of the study was to assess the relation between ADM measured at admission and in-ICU mortality in consecutive patients regardless the cause of admission.

**Methods:** The French and euRopean Outcome reGistry in Intensive Care Units (FROG-ICU) study was a multicenter observational study, including 2087 consecutive patients followed up to one year for those who survived to ICU stay. The protocol has previously been described [2]. Plasma were collected at admission for all patients and at discharge for in-ICU survivors. ADM was measured in all plasma using a sandwich assay specific for bio-ADM. The association between in-ICU mortality and the level of ADM was assessed by univariate analysis and adjusted analysis for severity at admission measured by the SAPS-II. Improvement in area under the ROC curve and reclassification indices were assessed.

**Results:** 2087 patients have been included, 65 % male with a median age of 63 (51-74), a median Charlson score of 3 (1-5) and a median SAPS-II 49 (36-63). Septic shock was present in 488 (23 %) patients. Median (and interquartile range) of ADM in-ICU survivors and non-survivors was 57 pg/mL [30-114] and 110 pg/mL [63-220], respectively (p < 0.001). Hazard ratio of in-ICU death for patients with a level of ADM higher than the median value was 2.12 (95%CI: 1.73-2.60) and 1.68 (95%CI: 1.36-2.07) when adjusted for SAPS-II. Area under the ROC curve of SAPS-II was significantly improved by the addition of ADM (0.653 [0.624 - 0.682] to 0.702 [0.675 - 0.729], p = 0.01).

**Conclusions:** In the present study, ADM was independently associated with in-ICU mortality and improved prognostic prediction. The clinical and therapeutic implication of these findings need to be further investigated.

**References**

[1] R Marino et al. Critical Care 2014, 18:R34

[2] Mebazaa et al. BMC Anesthesiology (2015) 15:143

## P020 Impact of disease severity assessment on performance of heparin-binding protein for the prediction of septic shock

### R. Arnold^1^, M. Capan^1^, A. Linder^2^, P. Akesson^2^

#### ^1^Christiana Care Health System, Newark, USA; ^2^Skane University Hospital, Lund University, Lund, Sweden

**Introduction:** Prognostic biomarkers for sepsis are described irrespective of patient severity. Our objective was to describe the ability of Heparin-Binding Protein (HBP) to predict the development of septic shock relative to the PIRO score.

**Methods:** Design: A secondary analysis of a multi-centered observational study. Inclusion: 1. adults >17 years with acute infection, 2. hospital admission and 3. two measurements of HBP. Exclusion: Hypotension within the first 12 hours. Initial PIRO scores calculated on arrival. Outcome: The development of delayed hypotension (dShock) = sBP <90 after initial assessment. Analysis: Subjects were grouped according to PIRO score range using previously defined cutoffs, and outcomes for each group were summarized. Average values and change in HBP were described across each group.

**Fig. 4 (Abstract P020).**
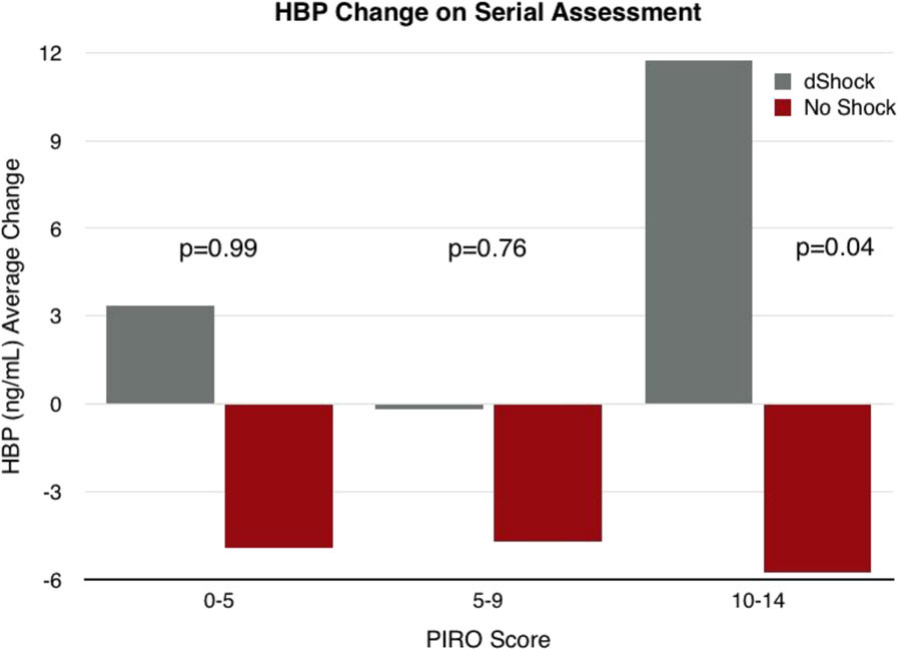
Serial change in HBP in subjects who develop delayed shock (dShock).

**Results:** 367 subjects were identified from original 759 meeting all study criteria. Frequency by disease severity: PIRO 0-4: 109 (30 %); PIRO 5-9: 191 (52 %); PIRO 10-14: 67 (18 %). There was a progressive increase in the frequency of dShock by increasing severity (37 %, 51 %, and 61 %). HBP was significantly elevated in dShock subjects across all subgroups (0-4:66 v 25; 5-9:75 v 39; 10-14:87 v 36, p < 0.05). The change in HBP was significant at the high-severity subgroup (Fig. 4).

**Conclusions:** There is an increased predictive performance of HBP associated with worsening sepsis severity. Assessment of serial HBP provided additional benefit at the high-severity subgroup.

## P021 Kinetics and prognostic value of presepsin (sCD14) in septic patients. A pilot study

### M. Popescu, D. Tomescu

#### Carol Davila University of Medicine and Pharmacy, Bucharest, Romania

**Introduction:** sCD14 represents a key receptor for Lipopolysaccharide - Lipopolysaccharide binding protein complexes and activation of TLR-4 cascade. Our aim was to investigate the potential prognostic role of this new marker on a cohort of patients with severe sepsis and septic shock.

**Methods:** We prospectively included 148 patients admitted to the ICU with the suspected diagnosis of sepsis. Of these, 28 patients met the inclusion criteria and were included in the final analysis. sCD14, procalcitonin (PCT), C-reactive protein (CRP) were recorded at admission ICU day 1 and ICU day 3. Organ dysfunction, sepsis severity scores (SOFA scores, Apache II score), vital parameters and management were recorded during ICU stay.

**Results:** sCD14 at admission was associated with cardiovascular dysfunction: decreased mean arterial pressure (correlation coefficient = -0.552, p = 0.048) and increased vasopressor support (correlation coefficient = 0.761, p = 0.035). Patients with increase sCD4 had a more severe hepatocytolysis as demonstrated by increased levels of ALT (p = 0.019) and AST levels (p = 0.040). ICU length of stay correlated with sCD14 levels at admission (p = 0.011) and ICU day 1 (p = 0.012). Patients with sCD14 levels < 2000 at admission had the lowest mortality rates and patients with presepsin levels > 6000 had the highest mortality. Statistically significant differences were observed between sCD14 between survival and non-survivals on admission (p = 0.042), ICU day 1 (p = 0.036) and ICU day 3 (p = 0.042).

**Conclusions:** Although sCD14 is associated with cardiovascular and hepatic dysfunction, the exact dynamics of sCD14 are still unknown. We observed a good correlation between presepsin levels at the time of admission and ICU survival, demonstrating a good prognostic role of this new inflammatory marker.

## P022 Comparison of CD64 levels performed by the facs and accellix systems

### C. L. Sprung^1^, R. Calderon Morales ^1^, G. Munteanu^1^, E. Orenbuch-Harroch ^1^, P. Levin^1^, H. Kasdan^2^, A. Reiter^2^, T. Volker^2^, Y. Himmel ^2^, Y. Cohen^2^, J. Meissonnier ^2^

#### ^1^Hadassah-Hebrew University Medical Center, Jerusalem, Israel; ^2^LeukoDx, Jerusalem, Israel

**Introduction:** Differentiating patients who are infected or not in the intensive care unit (ICU) can be very difficult. Present diagnostic tests remain inadequate. CD64 has been found to be a potentially useful marker to identify infected patients. Unfortunately, CD64 measured by standard flow cytometers in a laboratory takes hours to perform. The purpose of this study was to evaluate the Accellix CD64 instrument which provides infection assessment in 30 minutes in ICU patients.

**Methods:** Infected (ICUi) and non-infected ICU patients (ICU Control-ICUc) and normal volunteers (C) had CD64 levels measured by the Accellix CD64 instrument. Measurements were calculated as ‘CD64 index’, i.e. the ratio between the median fluorescence of the PMN population and the median fluorescence of the control beads population. Infection assessment was categorized per the CDC and ISF criteria as having a DEFINITE, PROBABLE, POSSIBLE, or NO INFECTION.

**Fig. 5 (Abstract P022).**
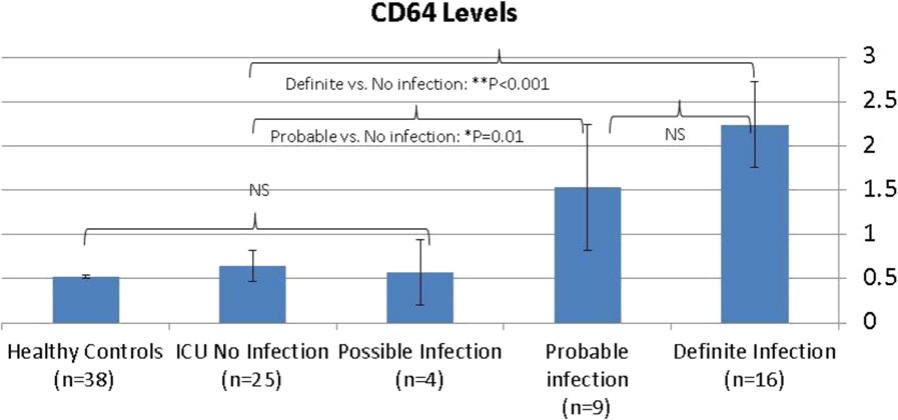
ᅟ

**Results:** 92 subjects were studied (ICU- 54 and C-38). CD64 Index levels were higher (mean ± SEM) in ICU definite and probable infection patients than ICU no infection and normal control patients (Definite Infection 2.24 ± 0.48, Probable Infection 1.54 ± 0.7, Possible Infection 0.57 ± 0.37, No Infection 0.65 ± 0.17, Healthy 0.53 ± 0.02). Results can be seen in Fig. 5.

**Conclusions:** CD64 Index levels are higher in infected than non-infected ICU patients. Accellix CD64 is a promising instrument to differentiate infected from non-infected ICU patients in a timely manner.

## P023 Diagnosing sepsis in 5 minutes: Nanofluidic technology study with pancreatic-stone protein (PSP/ reg)

### L. Girard, F. Rebeaud

#### Abionic SA, Lausanne, Switzerland

**Introduction:** Current biomarkers for sepsis diagnosis are neither sufficiently specific nor sensitive. Moreover, comprehensive diagnosis is today delayed by the long time-to-result required for the quantitative measurement of blood proteins in centralized laboratories. Therefore, the need to combine a rapid, near-patient diagnostic platform with the accurate measurement of proteins in complex matrices is the key to improve sepsis patient outcome. We developed nanofluidic biosensors that accelerate molecular interaction and thereby reduces incubation time from hours to minutes. Biosensors are analyzed in the abioSCOPE, a miniaturized automated fluorescence microscope. Fluorescent antibodies specific for the tested protein are mixed with 50 μ l of blood collected at the patient’s fingertip. Complexes of detecting antibodies and analytes are captured on the sensing area of the biosensors and, upon excitation, emit a signal proportional to the concentration of the analyte. As a proof-of-concept, we prepared biosensors for the quantification of pancreatic stone protein (PSP/reg), a promising biomarker that showed its superior ability to predict the outcome of patients affected by sepsis in several clinical studies [1].

**Methods:** PSP/reg biosensors were analyzed in the abioSCOPE to determine the analytical performances of the test. PSP/reg levels measured in a panel of serum samples from patients who underwent surgery were measured in the abioSCOPE and compared to the concentration measured in a microtiter plate ELISA.

**Results:** High analytical specificity was showed in a competitive inhibition study. Analytical sensitivity of the test is inferior to the mean PSP/reg value of a cohort of healthy individuals and the test is linear up to 1000 ng/ml of PSP/reg, thereby meeting clinical requirements. In a small-size comparison study, a good agreement was observed for PSP/reg values measured in microtiter ELISA and in the abioSCOPE.

**Conclusions:** The analytical performances of the PSP/reg test in nanofluidic biosensors highlight that rapid and accurate quantitative measurement of low abundance proteins can be achieved at no quality costs. More data is however needed to better evaluate the diagnostic performance of PSP/reg in various settings. Quantification of PSP/reg in 5 minutes in the abioSCOPE, together with the evaluation of other specific clinical signs performed at the bedside, will hopefully improve the decision-making process of patient with suspected sepsis and improve patient’s management in various clinical scenarios.

**Reference**

[1] Busani S et al. Critical Care 16:143, 2012

## P024 How nanotechnology-based approaches could contribute to sepsis prevention, diagnosis and treatment

### I. Herrmann

#### Swiss Federal Laboratories (Empa), St. Gallen, Switzerland

**Introduction:** Sepsis remains a major cause of mortality in intensive care units and its incidence is increasing along with the antibiotic resistance of the causing microorganisms. The management of sepsis is technically demanding and costly with only few therapeutic options available. Prompt diagnosis and hence early treatment has a major impact on patient survival. The advent of nanotechnology has brought with it an astonishing number of novel tools enabling novel technological solutions [1].

**Methods:** Here I will discuss how nanotechnology-enabled approaches could contribute to the prevention, diagnosis and treatment of bacterial infections.

**Results:** I will present nanoparticle-based approaches for ultrasensitive detection of analytes in body fluids and the rapid removal of pathogens from whole blood using magnetic nanoparticles [2]. Additionally, potential hurdles encountered when translating nanomaterial-based approaches into clinical settings will be illustrated.

**Conclusions:** This presentation will critically discuss the opportunities and challenges of particle-enabled approaches for the diagnosis and treatment of bacterial infections.

**References**

1.) Herrmann IK, Critical Care 19 (1), 239, 2015.

2.) Herrmann IK, Nanoscale 5 (18), 8718-8723, 2013.

## P025 Il7r transcriptional expression analysis during septic shock

### B. Delwarde, E. Peronnet, E. Cerrato, F. Venet, A. Lepape, T. Rimmelé, G. Monneret, J. Textoris

#### Pathophysiology of injury induced immunosuppression (PI3) Lab, Lyon 1 University / Hospices Civils de Lyon / bioMérieux, Lyon, France

**Introduction:** Despite therapy improvement septic shock mortality is still around 30 %. Better understanding of immunosuppression mechanisms led to new therapeutics perspectives such as recombinant IL-7 [1]. However it remains to better identify patients eligible for such immunotherapy. The soluble form of IL-7 receptor seems to be a promising candidate biomarker for this purpose since an association between its plasmatic concentration and mortality has been reported [2]. As we have no data on the transcriptional regulation of IL7R gene expression in septic shock, the aim of this study was to describe, in this pathology, the expression of several mRNA splicing variants of IL7R and to assess their association with mortality.

**Methods:** This retrospective study involved 30 ICU patients with septic shock. Whole blood sample was collected on the first (D1) and the third day (D3) after septic shock diagnosis. Expression levels of IL7R variants were measured using appropriate RT-qPCR designs: one specific of the variant encoding the membrane form of IL-7R, one specific of a variant corresponding to a potential soluble form and one covering all known splicing variants, including the 2 mentioned previously. Expression levels of these variants were described according to time and compared to 19 healthy volunteers. The association between our candidate biomarkers and day 28 status was assessed by logistic regression analyses. ROC curves were performed in order to evaluate performances.

**Results:** We noticed a decrease of expression levels of all IL7R splicing variants in patients compared to controls. An association between mortality and IL7R decreased expression was found for measurements at D3 and the ratio of expression D3/D1. This association was observed whatever the transcription variant used, in particular at D3 for the membrane form and for the potential soluble form of IL7R (OR = 0.28 [0.09–0.86], p = 0.026 and OR = 0.26 [0.07–0.93], p = 0.039, respectively). The ROC AUC for these models were 0.81 [0.65–0.97] and 0.84 [0.69–0.98].

**Conclusions:** This work put in highlight a marked decreased expression level of all IL7R splicing variants studied during septic shock. Persistence of lower expression on D3 was associated with higher mortality. The quantification of the expression of IL7R could provide an interesting biomarker to identify the most seriously ill patients who could benefit from new immunotherapies.

**References**

[1] Hotchkiss R et al.: Lancet Infect Dis 2013; 13: 260 – 8

[2] Demaret J et al.: Intensive Care Med 2014; 40: 1089 – 96

## P026 Disbalance of microbial metabolites of aromatic acids affects the severity in critically ill patients

### N. Beloborodova, V. Moroz, A. Osipov, A. Bedova, Y. Sarshor, A. Pautova, A. Sergeev, E. Chernevskaya

#### Negovsky V.A. Research Institute of General Reanimatology, Moscow, Russia

**Introduction:** Previously it was found in blood of septic patients a high level of three phenylcarboxylic acids (PhCAs) - phenyllactic (PLA), p-hydroxyphenyllactic (p-HPLA), p-hydroxyphenylacetic (p-HPAA). These metabolites of aromatic acids have predominately microbial origin in human body [1]. Experimentally demonstrated that PhCAs affect the function of mitochondria [2]. It was shown prognostic value of PhCAs at ICU admission [3]. In this study, we investigated in different ICUs the relationship of disbalance of PhCAs with severity and mortality.

**Methods:** Blood samples (n = 147) were collected in two medical centers from patients on the day of admission to ICU. Patients from mixed ICU (M-ICU), n = 89, APACHE II 12 [8-16] were with documented infection of various localization, average age 58 (47-65) years. Patients from surgical ICU (S-ICU), n = 58, APACHE II 8 [5-16]) were with intestine perforation (n = 35) and intestinal obstruction (n = 23) after emergency surgery, the average age 64 (52-78) years. Serum levels of PhCAs were measured using Gas-chromatography. Healthy adult donors (n = 72) were used as a control [4]. Data were compared by Mann–Whitney U-test, Chi-square test Pearson’s correlation coefficient (IBM SPSS Statistics 22).

**Results:** It is noted that phenylpropionic acid (PPA) disappeared in critical ill patients while always present in healthy. Levels of PLA, p-HPLA and p-HPAA were significantly higher in critically ill patients of both ICUs versus donors (p < 0,05). A positive correlation with total serum levels of the 3 PhCAs and APACHE II were found (rM-ICU = 0.7 and rS-ICU = 0,8, p < 0.001). PLA and p-HPLA was significantly higher in patients with arterial hypotension (MAP < 70 mm). Mortality was 31 % in ICUs. The total 3PhCAs serum level in died (n = 45) was higher 6 times versus surviving (n = 102), p < 0,001. Area under the prognostic ROC curve for APACHE II scale was 0,9 (p < 0,001), for ∑ 3PhCAs 0,9 (p < 0,001), this confirms the high predictive value of ∑ 3PhCAs.

**Conclusions:** We observed disbalance of aromatic acids metabolites in critically ill patients. Absence of serum PPA and high level of PLA, p-HPLA, p-HPAA are associated with severity and mortality. It is advisable intensify studies of the molecular mechanisms with participation of PhCAs in critically state.

Supported by Russian Science Foundation Grant ^1^15-15-00110.

**References**

1. Beloborodova NV et al. Biochem. (Mosc) 74:1350-55,2009

2. Fedotcheva NI et al. Toxicol Lett. 180(3):182-8, 2008

3. Beloborodova NV et al. Shock 44 (Supp 2): P13, 2015

4. Beloborodova N.V et.al. Biochem. (Mosc) 80(3):374-8,2015

## P027 Copeptin predicts 10-year all-cause mortality in community patients

### J. Odermatt^1^, R Bolliger^1^, L Hersberger^1^, M Ottiger^1^, M Christ-Crain^2^, B Mueller^1^, P Schuetz^1^

#### ^1^Kantonsspital Aarau, Aarau, Switzerland, ^2^University Hospital Basel, Basel, Switzerland

**Introduction:** We evaluated associations of copeptin levels with 10 year mortality in otherwise healthy patients visiting their general practitioner (GP) for a respiratory infection included in a previous trial (1).

Copeptin, the C-terminal part of the arginine vasopressin precursor peptide, is secreted in response to stress and correlates with adverse clinical outcomes in the acute-care hospital setting (2, 3). There are no comprehensive data regarding its prognostic value in the community.

**Methods:** This is a secondary analysis including data from 359 patients included in the PARTI trial. Copeptin was measured in batch-analysis on admission and after 7 days. We calculated cox regression models and area under the receiver operating characteristic curve (AUC) to assess an association of copeptin with mortality. Mortality data were collected through phone interviews 10 years after trial inclusion.

**Results:** After a median follow-up of 10.0 (interquartile range (IQR) 9.5-10.3) years, mortality was 9.8 %. Median admission copeptin levels were significantly elevated in non-survivors compared to survivors (13.8 pmol/l, IQR 5.9-27.8; vs. 6.3 pmol/l, IQR 4.1-11.5; p < 0.001), Admission copeptin levels were associated with 10-year all-cause mortality (age-adjusted hazard ratio 1.7 (95%CI, 1.2-2.5); p < 0.001, AUC 0.68). Results were similar for discharge copeptin levels. Admission and day 7 copeptin levels were also strong predictors for adverse outcome defined as death, pulmonary embolism and major adverse cardiac and cerebrovascular events.

**Conclusions:** In a sample of otherwise healthy patients visiting their GP for a respiratory infection, copeptin levels were strongly associated with 10-year all-cause mortality. In conjunction with traditional risk factors, this marker may help to better direct preventive measures in this population.

**References**

(1) Briel, M., et al., Procalcitonin-guided antibiotic use vs a standard approach for acute respiratory tract infections in primary care. Arch Intern Med, 2008. 168(18): p. 2000-7; discussion 2007-8.

(2) Nickel, C.H., R. Bingisser, and N.G. Morgenthaler, The role of copeptin as a diagnostic and prognostic biomarker for risk stratification in the emergency department. BMC Med, 2012. 10: p. 7.

(3) Iversen, K., et al., Risk stratification in emergency patients by copeptin. BMC Med, 2014. 12: p. 80.

## P028 Identification of differential proteomic response in septic patients secondary to community and hospital acquired pneumonia

### NK Sharma^1^, AK Tashima^1^, MK Brunialti^1^, FR Machado^1^, M Assuncao^2^, O Rigato^3^, R Salomao ^1^

#### ^1^UNIFESP, Sao Paulo, Brazil; ^2^Albert Einstein Hospital, Sao Paulo, Brazil; ^3^Sírio Libanês Hospital, Sao Paulo, Brazil

**Introduction:** Sepsis is a systemic inflammatory response caused by infection and leading cause of the death across the globe. Severity of infection increase if untreated and lead to more lethal conditions, such as severe sepsis and septic shock. There are several source of infection for development of sepsis; however, half of the cases start from lung and known as pneumonia. On the basis of infection site, pneumonia can be categorised as community acquired pneumonia (CAP) and hospital acquired pneumonia (HAP). To understand the response of body under CAP and HAP, we compare the plasma proteome of sepsis patients.

**Methods:** In the present study, we used quantitative proteomic approach (iTRAQ) to identify differential expression from survival and non-survival septic patients secondary to CAP and HAP. In brief, the plasma samples were depleted for high abundant proteins, trypsin digested and labelled with iTRAQ 8plex system. The tryptic peptides were separated by SCX chromatography and analysed with LC-MS. The collected data processed and submitted to mascot search engine with selection of swissprot database and 1 % FDR. The results from mascot were further analysed by isobaric Q.

**Results:** The proteome profile unable us to indentify total 64 and 61 differentially expressed proteins in CAP and HAP survival patients, while, 68 and 75 protein were identified in CAP and HAP non survival patients. Total 26 proteins were common in survival patients, whereas 38 and 35 proteins were specific for CAP and HAP, respectively. Total 33 proteins were common in non survival patients, whereas 35 and 42 proteins were specific for CAP and HAP, respectively. On the comparison of proteins across all groups, 21 proteins were common; 12 and 10 proteins were specific to CAP and HAP survival, while, 13 and 16 protein were unique to CAP and HAP non survival groups. These proteins are key molecules for various biological functions including inflammatory response as well as signalling cascade like acute phase response etc. The further analysis and validation is in progress.

**Conclusions:** This study provides the first molecular level evidence of differential response in septic patients secondary to CAP and HAP. The results show higher numbers of unique proteins to corresponding groups. Underlying diseases, previous therapeutic interventions and different etiology may underscore the differences observed at a protein level.

## P029 Monocyte HLA-DR expression in community-acquired bacteremic sepsis - dynamics associated to aetiology and prediction of secondary sepsis

### SC Cajander^1^, G Rasmussen^1^, E Tina^1^, B Söderquist^1^, J Källman^1^, K Strålin^2^

#### ^1^Faculty of Medicine and Health Örebro University, Örebro, Sweden; ^2^Department of Infectious Diseases, Karolinska University Hospital, Stockholm, Sweden

**Introduction:** Sepsis induced immunosuppression is an important risk factor for unfavourable outcome in severe sepsis. The monocyte HLA-DR expression (mHLA-DR) is suggested as a useful biomarker for immunosuppression. In this prospective study of bacteremic sepsis we aimed to; 1) assess the predictive value of mHLA-DR for secondary sepsis, and 2) compare mHLA-DR levels and dynamics of patients with different bacteremic aetiology of sepsis.

**Methods:** Septic patients with positive blood cultures (n = 111), 1-2 days after admission, were included. Sampling was additionally preformed on day 3, 7, 14, and 28. mHLA-DR was analysed by flow cytometry, using a standardized protocol [1]. Data on events of secondary bacteraemic sepsis were collected retrospectively.

**Fig. 6 (Abstract P029).**
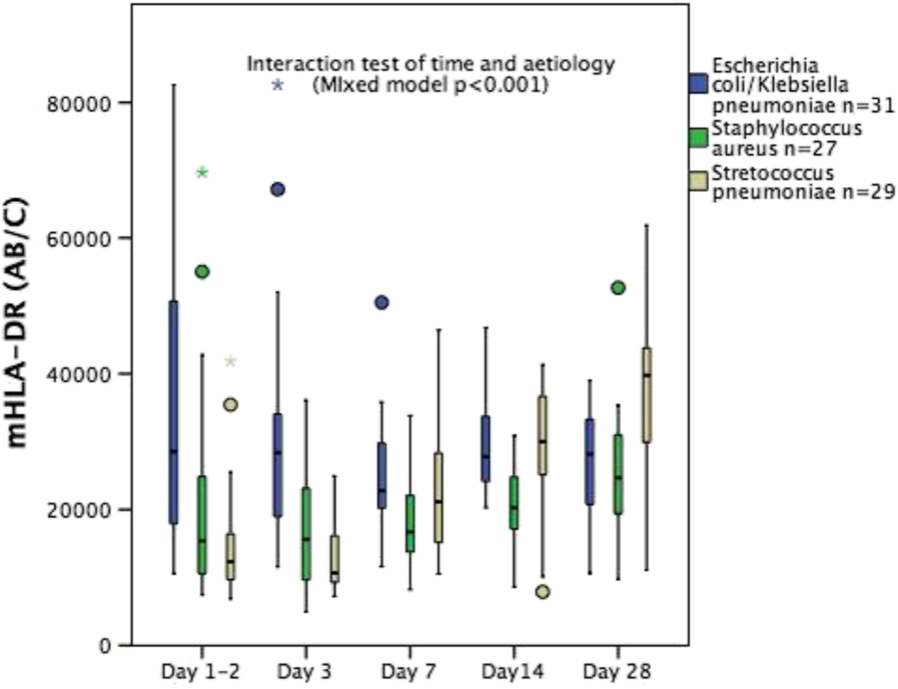
Dynamic expression of mHLA-DR in bacteremic sepsis

**Results:** Secondary sepsis occurred in 7 cases. The mHLA-DR levels on day 1-2 were significantly lower for cases who developed secondary sepsis; median 8990 vs. 18200 AB/C (p = 0.009), AUC 0.798. The negative predictive value (NPV) was 98 %, and the positive predictive value (PPV) was 22 %. The three most prevalent pathogens demonstrated differences in linear association over time, as shown in Fig. 6. The mean mHLA-DR increased significantly to day 7 for [i]S.pneumoniae[/i]. A modest increase was seen for [i]S.aureus[/i].

**Conclusions:** In this clinical setting, a cut-off value of 12500 AB/C on day 1-2 was associated with a high NPV for secondary sepsis. The mHLA-DR levels were lower in patients who acquired secondary sepsis. The aetiology of sepsis was associated with differences in mHLA-DR dynamics with the lowest initial values and greatest recovery demonstrated for [i]S.pneumoniae[/i]

**Reference**

[1] Döcke WD et al. Clin Chem. 2005;51:2341-7.

## P030 Soluble B- and T-lymphocyte attenuator: A possible prognostic marker in sepsis

### AL Lange ^1^, JS Sundén-Cullberg^2^, AM Magnuson^1^, OH Hultgren^1^

#### ^1^Faculty of Medicine and Health, Örebro, Sweden; ^2^Center for Infectious Medicine, Karolinska Institutet, Karolinska University Hospital, Stockholm, Sweden

**Introduction:** Upregulation of the expression of negative co-stimulatory molecules on T-cells is one of the mechanisms behind immunoparalysis in sepsis. We wanted to evaluate the prognostic capacity of the soluble isoform of the negative co-stimulatory receptor soluble B- and T-lymphocyte Attenuator (sBTLA) in severe sepsis and septic shock.

**Methods:** A prospective observational study was conducted in the mixed intensive care unit of Karolinska University Hospital, Huddinge, Sweden. 101 patients with severe sepsis and septic shock (sepsis cohort) and 28 patients with non-infectious critical illness (ICU controls) were included. 31 blood donors served as healthy controls. Blood samples taken on enrollment and at 24 and 48 hours were included in the analysis. Plasma concentration of sBTLA was measured with Enzyme-linked Immunosorbent Assay (ELISA). Patients were followed from inclusion until day 28 or the day of death. Prognostic capacity of sBTLA was evaluated on a categorical scale (the patients were divided into three categories with the same number of subjects, based on the concentration of sBTLA) with Cox Regression and adjusted for age. Comparison of plasma concentrations between survivors and non-survivors was made with the Mann-Whitney U test.

**Results:** sBTLA levels were statistically significantly increased in the sepsis cohort compared to ICU controls and blood donors. The overall 28 day mortality was 18 %. sBTLA levels were statistically significantly higher at the time of study inclusion and until 48 hours in 28 day sepsis non-survivors than in survivors, and did not change over time. 28 day mortality was 5-fold higher in patients with a baseline plasma sBTLA > 21 ng/mL compared to those with a level lower than 9 ng/mL (HR 5.0, 95 % CI 1.3-18, p = 0.02), but there was no increased risk for death for patients with intermediate (10-21 ng/mL) compared to low baseline sBTLA (HR 1.2, 95 % CI 0.2-5.8, p = 0.8).

**Conclusions:** Plasma concentrations of soluble BTLA were increased in severe sepsis/septic shock compared to in patients with non-infectious critical illness and healthy controls, and did not change significantly over the first 48 hours after study inclusion. A baseline sBTLA concentration >21 ng/mL was a negative prognostic indicator.

## P031 Fractal dimension: A new biomarker for quantifying clot microstructure in patients across the sepsis spectrum

### G Davies, S Pillai, G Mills, R Aubrey, K Morris, P Williams, P Evans

#### NISCHR Haemostasis Biomarker Research Unit, Swansea, UK

**Introduction:** It is well recognised that coagulation is altered across the sepsis spectrum (sepsis, severe sepsis and septic shock). This can range from an increased thrombotic risk to disseminated intravascular coagulation (DIC) contributing to increased morbidity and mortality. Previous studies have attempted to investigate these changes in coagulation using standard and global markers of coagulation [1]; however, clot microstructure has not been investigated. Recent research has led to the development of a new biomarker of clot microstructure, Df, which quantifies the fibrin network of the incipient clot [2]. This study aims to investigate the role of clot microstructure in sepsis, by using new quantitative biomarker of clot microstructure, Df.

**Methods:** This study had full ethical approval from the South West Wales Research Ethics Committee. Patients with a diagnosis of sepsis were recruited from the Emergency Department (ED) and Intensive Care Unit (ICU) of a large teaching hospital in South Wales. Blood samples were taken to determine Df, full blood count and standard coagulation screen. A healthy control group matched for age and gender was also recruited.

**Results:** 95 patients were included in the study: 49 with sepsis, 19 with severe sepsis and 27 with septic shock. 44 healthy volunteers were recruited as a matched control. Mean Df in the healthy control group was 1.74 ± 0.03. Mean Df in patients with sepsis and severe sepsis was significantly higher (1.78 ± 0.07 and 1.80 ± 0.05 respectively (p < 0.05, One-way ANOVA Post-hoc Bonferroni correction)). Mean Df in patients with septic shock was significantly lower compared to all other groups (1.66 ± 0.10 (p < 0.001, One-way ANOVA Post-hoc Bonferroni correction)). Df was also significantly lower in non-survivors than survivors at 28 days (1.66 ± 0.13 vs 1.76 ± 0.08, p = 0.006 (Students t-test)).

**Conclusions:** Our results indicate that patients with sepsis and severe sepsis form tight highly branched fibrin clots (as indicated by high Df). As the disease progresses to septic shock, much weaker clots are formed (as indicated by low Df). This may help to explain the dichotomy of thrombogenicity and bleeding diathesis in patients across the sepsis spectrum. The new functional biomarker, fractal dimension (Df), therefore can be used to quantify clot microstructure across the sepsis spectrum. It can also be used as an outcome measure, although this study was not powered for outcome.

**References**

1. Collins P, et al. BJH. 2006;135:220-7.

2. Evans PA, et al. Blood. 2010;116:3341-6.

## P032 Comparison between the new biomarker for coagulation, clot microstructure (Df) with rotational thromboelastometry (ROTEM) in patients across the sepsis spectrum

### S Pillai, G Davies, G Mills, R Aubrey, K Morris, P Williams, P Evans

#### NISCHR Haemostasis Biomarker Research Unit, Swansea, UK

**Introduction:** ROTEM is a viscoelastic test of coagulation, and has been investigated as a tool for evaluating haemostasis in sepsis [1]. It has been shown that a hypocoagulable viscoelastic profile is associated with poor outcomes in patients with severe sepsis [2]. ROTEM measures several parameters that provide information on the kinetics and structure of the developing clot. Clot microstructure (Df) on the other hand evaluates a single parameter that quantifies microstructural properties of the clot. The aim of this study was to compare the new biomarker, Df and ROTEM in patients across the sepsis spectrum (sepsis, severe sepsis and septic shock).

**Methods:** This study had full ethical approval from the South West Wales Research Ethics Committee. Patients with a diagnosis of sepsis (SIRS with infection) were recruited from the Emergency Department and Intensive Care Unit of a large teaching hospital in South Wales. Blood samples were taken to determine Df, and ROTEM.

**Results:** 95 patients were included in the study: 49 with sepsis, 19 with severe sepsis and 27 with septic shock. 44 healthy volunteers were recruited as a matched control. Df correlated significantly with both structural and kinetic ROTEM parameters (p < 0.05), however the correlations were weak (Pearson Correlation <0.4). The strongest correlation was observed between Df and INTEM Clot Formation Time (CFT), with a moderate correlation coefficient of -0.428. Df also correlated significantly with ROTEM lysis parameters (LI45, LI60), but these correlations were also weak.

**Conclusions:** Both Df and ROTEM appear to provide independent information about the coagulation system across the sepsis spectrum. This could be due to the difference in the activation of the coagulation pathway. Whereas whole unadulterated blood was used in determining Df (immediately activated through contact after collection of the sample), ROTEM utilises blood that is deactivated with sodium citrate, and reactivated through tissue factor or contact activation. This study concludes that ROTEM do not provide information about the clot microstructure.

**References**

1. Muller MC, et al. Crit Care. 2014;18:R30.

2. Ostrowski SR, et al. J Crit Care. 2013;28:317e1-317e11.

## P033 Changes in fibrinolysis across the sepsis spectrum: The use of rotational thromboelastometry (ROTEM) lysis index (LI60) and D-Dimer concentration

### S Pillai, G Davies, G Mills, R Aubrey, K Morris, P Williams, P Evans

#### NISCHR Haemostasis Biomarker Research Unit, Swansea, UK

**Introduction:** It is well recognised that coagulation is altered across the sepsis spectrum (sepsis, severe sepsis and septic shock). Sepsis is recognised to be a prothrombotic state, due to an increased procoagulant activity and impairment of the natural anticoagulants and fibrinolytic proteins [1]. Altered fibrinolysis could have a key role in the accumulation of microthrombi leading to disseminated intravascular coagulation (DIC). The aim of this study was to assess the changes in fibrinolysis across the sepsis spectrum using the Rotational Thromboelastometry (ROTEM) lysis index (LI60) and D-dimer concentration.

**Methods:** This study had full ethical approval from the South West Wales Research Ethics Committee. Patients with a diagnosis of sepsis were recruited from the Emergency Department and Intensive Care Unit of a large teaching hospital in South Wales. Blood samples were taken to determine ROTEM and D-Dimer concentration. Patients were followed up for 28 days to assess 28-day mortality.

**Results:** 95 patients were included in the study: 49 with sepsis, 19 with severe sepsis and 27 with septic shock. 44 healthy volunteers were recruited as a matched control. D-Dimer concentration was 422 (297, 881) ng/mL in sepsis, 789 (374, 1586) ng/mL in severe sepsis and 3073 (765, 7279) ng/mL in septic shock (normal range <250 ng/mL). Median EXTEM LI60 (%) in healthy volunteers was 92 (88.3, 94). ROTEM indicated fibrinolytic function that was comparable to healthy volunteers in the sepsis and severe sepsis groups (93 (90, 95) and 91 (87, 96) respectively), but fibrinolytic function was significantly impaired in septic shock compared to all other groups (97 (95, 98), p < 0.001, Kruskal-Wallis test with Bonferroni correction). Impaired fibrinolytic function was also significantly associated with mortality (INTEM LI60 of 94 (90.5, 96) in survivors vs 96 (94.8, 96) in non-survivors, p = 0.006, Mann-Whitney U Test).

**Conclusions:** Fibrinolytic activity was increased in patients with septic shock. However there was an impairment of the fibrinolytic function as measured by ROTEM. The exact mechanisms leading to this are not fully understood, however consumption of fibrinolytic factors could contribute as evidenced by elevated D-Dimer concentration. ROTEM lysis index (LI60) can potentially be used as a biomarker to identify septic shock and as an outcome measure, although this study was not powered for outcome.

**Reference**

1. Schouten M, et al. J Leukoc Biol. 2008;83:536-45.

## P034 The intensive care infection score – a promising marker for the prediction of infection and its severity

### P Van der Geest^1^, M Mohseni^1^, J Linssen^2^, R De Jonge^1^, S Duran^3^, J Groeneveld^1^

#### ^1^Erasmus Medical Center, Rotterdam, Netherlands; ^2^University Witten/Herdecke, Witten, Germany; ^3^Maasstad Ziekenhuis, Rotterdam, Netherlands

**Introduction:** The prediction of infection and its severity remains difficult in critically ill patients with suspected infection^1^. A novel, simple biomarker derived from five blood-cell derived parameters that characterize the innate immune response in routine blood samples, the intensive care infection score (ICIS), could be helpful in this respect ^2,3^. We therefore compared the predictive value of the ICIS with that of the white blood cell count (WBC), C-reactive protein (CRP) and procalcitonin (PCT) for infection and its severity in critically ill patients.

**Methods:** We performed a multi-center, cluster randomized, crossover study in critically ill patients between January 2013 and September 2014. Patients with a suspected infection for which blood cultures were taken by the attending intensivist were included. Blood was taken at the same time for WBC, ICIS, CRP and PCT measurements in the study periods. Patients were divided into groups of increasing likelihood of infection and invasiveness: Group 1 without infection or with possible infection irrespective of cultures, Group 2 with probable or microbiologically proven local infection without blood stream infection (BSI) and Group 3 with BSI irrespective of local infection.

**Results:** In total, 301 patients were enrolled with 452 suspected infection episodes (SIE), at more than 48 h intervals were analyzed. CRP, PCT and ICIS were higher in Group 2 and 3 than 1. The AUROC for the prediction of infection during the first SIE was 0.70 for CRP, 0.71 for PCT and 0.73 for ICIS (P < 0.001). For septic shock the AUROC was 0.73 for CRP, 0.85 for PCT and 0.76 for ICIS.

**Conclusions:** The data suggest that the ICIS is useful for the prediction of infection and its severity in critically ill patients. In contrast to CRP and PCT, the ICIS can be determined routinely without extra blood sampling or costs, yielding results within 15 minutes.

**References**

1. Vincent JL, Beumier M. Diagnostic and prognostic markers in sepsis (2013) Expert Rev Anti Ther 11(3): 265-275.

2. Nierhaus A, Linssen J, Wichmann D, et al (2012) Use of a weighted, automated analysis of the differential blood count to differentiate sepsis from non-infectious systemic inflammation: The Intensive Care Infection Score (ICIS). Inflamm Allergy Drug Targets 11:109-115.

3. Weimann K, Zimmermann M, Spies CD, et al (2015) Intensive Care Infection Score – A new approach to distinguish between infectious and noninfectious processes in intensive care and medicosurgical patients. J Int Med Res 43:435-451.

## P035 Challenges in the clinical diagnosis of sepsis

### R Miller III^1^, BK Lopansri^1^, LC McHugh^2^, A Seldon^2^, JP Burke^1^

#### ^1^Intermountain Healthcare, Salt Lake City, USA; ^2^Immunexpress, Seattle, USA

**Introduction:** Distinguishing sepsis from infection-negative systemic inflammation (SIRS) at admission based on clinical criteria poses a diagnostic dilemma that can lead to delayed therapy or overly aggressive treatment. This study quantified concordance of clinical impressions of sepsis.

**Methods:** A prospective, observational study was conducted at an academic medical center and a community hospital in Utah. Adults admitted to ICUs fulfilled SIRS criteria. The attending physician and a study investigator classified patients at admission as sepsis, SIRS, or indeterminate. The reference diagnosis was determined through independent review of all clinical data at discharge by two study physicians with adjudication by a third for discordant classifications. Inter-physician admission classifications at admission and discharge were compared.

**Fig. 7 (Abstract P035).**
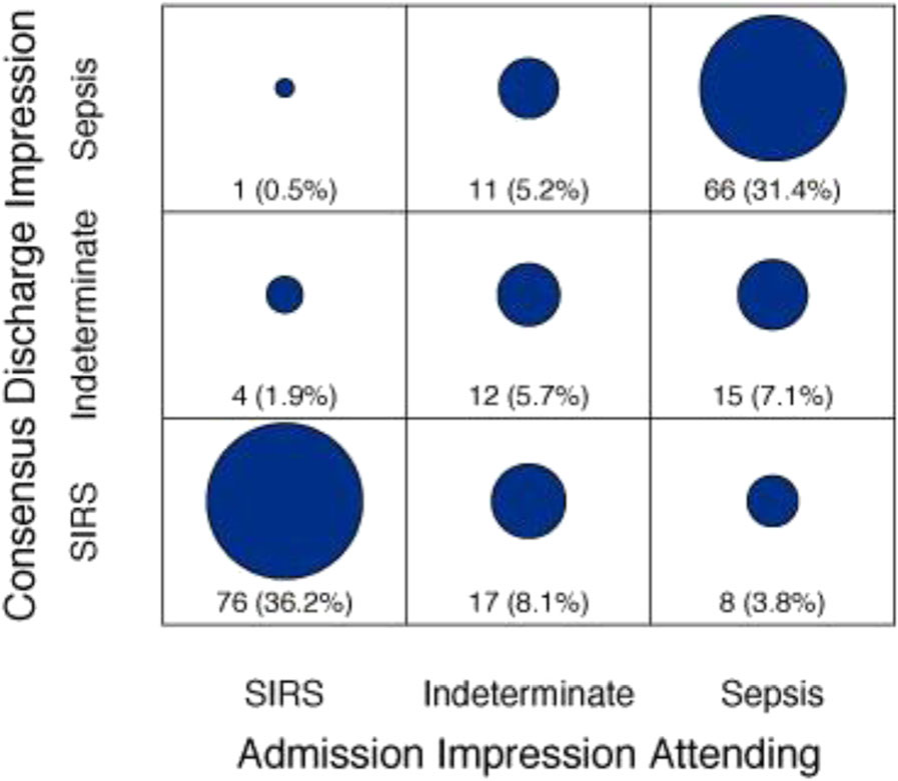
Classification of patients at admission and revised at discharge

**Results:** Of the 210 enrolled subjects, 58 (28 %) cases had at least one physician classify the patient as indeterminate, and in 9 (4 %) there was major disagreement with the classifications of SIRS and sepsis. At discharge, in 46 (22 %) cases, at least one classification was indeterminate and in 4 (2 %) there was major disagreement between classification of SIRS and sepsis. When compared to discharge classification (Fig. 7), 142 (68 %) cases initially classed as SIRS or sepsis by the attending physician remained unchanged. Of 89 patients initially diagnosed with sepsis on admission, 66 (74 %) were deemed to be correct, 8 (9 %) were deemed SIRS and 15 (17 %) indeterminate when compared to discharge classification. Of the 40 (19 %) subjects indeterminate on admission, 12 (30 %) remained so at discharge. 19 patients initially diagnosed with sepsis or SIRS became indeterminate at discharge. One patient initially classed as SIRS and 11 as indeterminate were ultimately discharged with a diagnosis of sepsis.

**Conclusions:** Considerable uncertainty exists with use of clinical criteria for sepsis in ICU patients. Accurate tests that improve early diagnostic accuracy are needed in the management of critically ill patients.

## P036 Does zero heat flux thermometry more accurately identify sepsis on intensive care?

### J Johnston, R Reece-Anthony, A Bond, A Molokhia

#### Lewisham and Greenwich NHS Trust, London, UK

**Introduction:** Temperature measurement is an essential component for decision making on intensive care both in the context of identifying and treating sepsis and also in accurate calibration of blood gas analysis. SpotOn™ a Zero Heat Flux Cutaneous thermometer has been validated as a non-invasive, continuous and accurate method of measuring temperature in patients undergoing cardiac surgery in the operative and peri-operative period.1 We compared SpotOn™ with Axilla thermometers, our current method of non-invasive temperature monitoring, to establish how inaccurate our current practice was.

**Methods:** 25 acute patients were selected on admission to intensive care at Lewisham Hospital, 17 male, 18 female, 16 medical and 9 surgical. Temperatures were recorded for an average of 47 hours on each patient using Covidien Fillac™ Axilla probes and SpotOn™ forehead probes as and when our current clinical practice indicated.

**Results:** A total of 435 comparative values were recorded over a range of 34.4C to 38.5C. On average the SpotOn™ thermometer recorded temperatures 0.14C above the Axilla (95 % CI +/-0.04 with a Wilcoxon matched-pairs signed rank test p < 0.0001). The average difference between SpotOn™ and Axilla over a normal range (36-37.4C) was 0.07 (95 % CI +/- 0.04) p = 0.0028. The average difference between SpotOn™ and Axilla at hypothermic temperatures (<36C) was -0.1 (95%CI +/-0.11 p = 0.13). The average difference between SpotOn™ and Axilla at hyperthermic temperatures (> = 37.5C) was 0.53C (95%CI +/- 0.11 p < 0.0001). There were 12 occasions when the SpotOn™ thermometer identified a SIRS defined temperature (<36 or > =38.3C) that the Axilla temperature did not, affecting 10 of the 25 patients.

**Conclusions:** Zero Heat Flux Cutaneous thermometers have separately been shown to accurately record core temperatures. We have highlighted that axilla probes are inaccurate in comparison to SpotOn™.

The Axilla probe appears to under-read at hyperthermic temperatures and over-read at hypothermic temperatures. The difference between the two methods appears most marked at hyperthermic temperatures.

As a consequence it is likely that axilla probes could fail to identify sepsis in patients on intensive care.

**Reference**

1. Eshraghi Y et al.: Anesth Analg 2014, XXX: 1-7

## P037 Advancing quality (AQ) sepsis programme: Improving early identification & treatment of sepsis in North West England.

### C. Mcgrath^1^, E. Nsutebu^2^

#### ^1^Wirral trust, Merseyside, UK; ^2^RLBUHT, Liverpool, UK

**Introduction:** AQ is based in the North West of England (population 9 M). The programme aims to improve sepsis care & clinical coding within the region. In the first 12 months the care of over 9500 patients with infection was examined.

**Methods:** A panel of clinical experts developed a sepsis measure set with the aid of BMJ evidence & identified a study population using 25 ICD10 sepsis codes. Patients with a sepsis code on discharge had their initial care examined against a series of evidence-based interventions. An Appropriate Care Score (ACS) measured compliance with the sepsis care measure set. The project is promoted by an ongoing series of collaborative events, where performance is published & best practice is shared. A healthy sense of competition drives improvement in performance & commissioners are able to monitor performance in their locality.

**Fig. 8 (Abstract P037).**
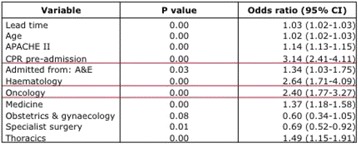
ᅟ

**Fig. 9 (Abstract P037).**
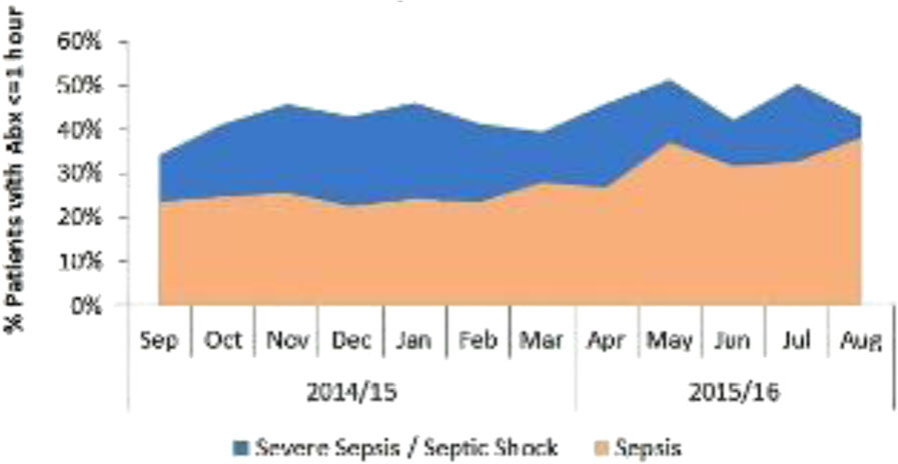
Graph showing monthly percentaghe of septic patients who receive antibiotics within 1 hour of presentation

**Results:** Figure 8 shows the increase in ICD-10 coding in AQ participating trusts in the region vs Non AQ participating trusts. Figure 9 shows that at the start of the programme 23.6 % of septic patients received antibiotics within 1 hour & in the last month this was up to 38.2 % of septic patients. ACSs have increased by 34 % when comparing the first 3 months & the last 3 months of the programme. There is a downward trend in mortality when patients have achieved all the measures within the measure set.

**Conclusions:** Use of the measure set and collaboration in the NW region of England appears to have improved early recognition & treatment of patients with sepsis. Results have demonstrated improvements in outcome measures, whilst recognising that improved coding & recording of sepsis may change the studied population.

## P038 Prehospital transport of acute septic patients

### P. Bank Pedersen^1^, D. Pilsgaard Henriksen^2^, S. Mikkelsen^3^, A. Touborg Lassen^1^

#### ^1^Department of Emergency Medicine, Odense University Hospital, Odense C, Denmark; ^2^Department of Respiratory Medicine, Odense University Hospital, Odense C, Denmark; ^3^Department of Anaesthesiology and Intensive Care Medicine, Odense University Hospital, Odense C, Denmark

**Introduction:** Sepsis is a systemic, potentially fatal host response to infection, in which the speed and appropriateness of the therapy administered in the initial hours are likely to influence outcome. Patients with sepsis are not always easily recognized prehospitally and septic patients thus may be transported to hospital either on their own accord, by ambulance or by physician assisted Mobile Emergency Care Unit (MECU). We wanted to address to which degree the type of transport from the prehospital scene and into the hospital is related to severity of sepsis and 30 day mortality.

**Methods:** We included all adult patients (> = 15 years) presenting to an emergency department between September 2010-August 2011 with a first-time admission of community-acquired sepsis of any severity. Cases and type of transport to the hospital were identified by manual chart review using predefined criteria.

**Results:** We included 1,713 patients. The median age was 72 years (IQR 57-81) and 793 (46.3 %) were men. 621 patients (36.3 %) were admitted with sepsis, 1,071 patients (62.5 %) with severe sepsis, and 21 patients (1.2 %) with septic shock.

Among patients with sepsis, 390 (62.8 %, 95%CI 58.9-66.6 %) transported themselves, 197 (31.7 %, 95%CI 28.1-35.5 %) were transported by ambulance, and 34 (5.5 %, 95%CI 3.8-7.7 %) by physician-assisted ambulance (MECU). Their 30-day mortality was 6.2 %, 5.6 % and 8.8 %.

Among patients with severe sepsis, 673 (62.8 %, 95%CI 59.9-65.7 %) transported themselves, 302 (28.2 %, 95%CI 25.5-31.0 %) were transported by ambulance and 96 (9.0 %, 95%CI 7.3-10.8 %) by the MECU. Their 30-day mortality was 16.2 %, 23.2 %, and 22.9 %.

Among patients septic shock, 10 transported themselves (47.7 %, 95%CI 59.9-65.7 %), 7 (33.3 %, 95%CI 14.6-57.0 %) were transported by ambulance, and 4 (19.0 %, 95%CI 5.4-41.9 %) by the MECU. Their 30-day mortality was 50.0 %, 12.5 %, and 37.5 %.

**Conclusions:** A substantial proportion of patients with sepsis arrive to the hospital by themselves, but the proportion of patients with ambulance or MECU transport increase by disease severity. This probably reflects the difficulty in diagnosing early sepsis.

## P039 Vasodilatory plant extracts gel as an alternative treatment for fever in critically ill patients

### R. Tincu^1^, C. Cobilinschi^1^, D. Tomescu^2^, Z. Ghiorghiu^1^, R. Macovei^1^

#### ^1^Bucharest Clinical Emergency Hospital, Bucharest, Romania; ^2^Fundeni Clinical Institute, Bucharest, Romania

**Introduction:** Fever is a sign that is commonly associated with syndromes in intensive care. Hyperthermia is initiated by the action of IL-1β at organum vasculosum laminae terminalis, where prostaglandin E2 is released. Ortogel is a gel which contains plant extracts (Capsicum, Arnica Montana, Symphytum Officinale, Juglans Regia, Tannus Communis, Sambucus, Armoracia Rusticana, Lavandula Angustifolia) with anti-inflammatory, analgesic and anti-pyretic effects, producing cutaneous vasodilation. The purpose of this study was to assess the anti-pyretic effects of Ortogel when compared to Acetaminophen administration in Intensive Care patients.

**Methods:** A randomized control trial was conducted in our Intensive Care Unit of the Clinical Emergency Hospital in Bucharest from January to June 2015. It included a total of 70 patients, divided in two equal groups, suffering from fever of infectious cause (cutaneous temperature of 38-39 degrees Celsius, measured using Nihon KOHDEN BSM 6000 monitor, in controlled room temperature). Hemodynamically unstable patients were excluded. The study group received 30 ml gel (Ortogel) with cutaneous administration while the control group received 1 g iv acetaminophen. Primary outcomes were difference in cutaneous temperature after 0.5, 1, 2 and 3 h from the baseline reading.

**Results:** Initially, the average temperature for the Ortogel group was 39.5 ± 0.11 degrees Celsius, with similar values in the control group (39.0 ± 0.14). Ortogel was able to significantly decrease hyperthermia at Δ T-max by 2.7 °C (p < 0.001), similar with paracetamol effects at Δ T-max of 3.2 °C (p < 0.001). The gel showed significant suppression in cutaneous temperature by 2.6 °C, from 39.5 ± 0.11 °C to 36.2 ± 0.32 °C (p < 0.001), and the percentage inhibition of fever was 6.8 %. The antipyretic effect started 45 min after administration (p < 0.01) and the reduction in rectal temperature was maintained for 3 h (p < 0.001).

**Conclusions:** The present study shows similar antipyretic effects of Ortogel when compared to intravenous Acetaminophen. The antipyretic effect installed in a similar time frame for the two groups. Ortogel may represent an alternative treatment provided for patients with Acetaminophen contraindications.

## P040 Host response and outcome of hypothermic sepsis

### M. A. Wiewel^1^, M. B. Harmon^1^, L. A. Van Vught^1^, B. P. Scicluna^1^, A. J. Hoogendijk^1^, J. Horn^1^, A. H. Zwinderman^1^, O. L. Cremer^2^, M. J. Bonten^2^, M. J. Schultz^1^, T. Van der Poll^1^, N. P. Juffermans^1^, W. J. Wiersinga^1^

#### ^1^Amsterdam Medical Center, Amsterdam, Netherlands; ^2^University Medical Center Utrecht, Utrecht, Netherlands

**Introduction:** Hypothermia is associated with adverse outcome in patients with sepsis. Knowledge on the pathophysiology is highly limited. The objective of this study was to establish whether hypothermia was associated with differences in the systemic host response to sepsis as reflected by cytokine profile, endothelial activation markers and cellular responsiveness towards lipopolysaccharide (LPS) during the first days of intensive care unit (ICU) admission.

**Methods:** A prospective observational study in patients with sepsis on ICUs of 2 tertiary hospitals in the Netherlands. Hypothermia was defined as the lowest body temperature measurement below 36 °C in the first 24 hours of ICU admission. Exclusion criteria were immunodeficiency, active cooling, readmission or admission from operating theatre or other ICU. Logistic regression was used to investigate the independent association of hypothermia with 90-day mortality. Plasma levels of endothelial markers and cytokine levels were measured upon ICU admission and at days 2 and 4 thereafter in all hypothermic and nonhypothermic patients with sepsis. Analyses were performed in the entire cohort as well as a cohort matched for disease severity. LPS ex vivo whole blood stimulation was performed on day 1 of admission in a subset of patients.

**Results:** Hypothermia was identified in 186 of 525 patients and was independently associated with increased mortality in multivariate analysis. At baseline, patients with hypothermic sepsis were significantly older, had a lower body mass index and increased incidence of cardiovascular disease. Levels of pro- and anti-inflammatory cytokines were not different between groups at all time points. Hypothermia was also not associated with an altered response to ex vivo stimulation with LPS in a subset of patients. Hypothermia was associated with sustained elevated levels of the endothelial activation marker fractalkine during the first 4 days of ICU stay compared to nonhypothermic patients in both the entire cohort and the cohort matched for disease severity.

**Conclusions:** Hypothermic sepsis is associated with increased mortality. Patients with hypothermia showed increased levels of fractalkine, irrespective of disease severity during the first 4 days of ICU stay.

This research was performed within the framework of CTMM, the Center for Translational Molecular Medicine (http://www.ctmm.nl), project MARS (grant 04I-201).

## P041 Septic shock alert over SIRS criteria has an impact on outcome but needs to be revised

### G. Eren, Y. Tekdos, M. Dogan, O. Acicbe, E. Kaya, O. Hergunsel

#### Bakirkoy Dr.Sadi Konuk Training and Research Hospital, Istanbul, Turkey

**Introduction:** Clinical information systems(CIS) through protocol-based alerts have long been used in intensive care units(ICU).They can serve for early recognition and intervention of sepsis and septic shock.We aimed to determine whether early response to “septic shock alert” has any impact on the true diagnosis and patient outcomes in septic shock.

**Methods:** 1384 patients admitted were reviewed over CIS(Metavision,iMDsoft) in Bakirkoy Dr.Sadi Konuk.An early warning was protocolized in the system relevant to international guidelines.Septic shock alert was activated if the condition as per “> = 2 systemic inflamatory response syndrome(SIRS) criteria together with SBP < 90 mmHg” was established.Patients were categorized into 3 based on intervals between first alert activation and initial response as per orders for diagnostics and interventions. Group I assigned for “early acknowledgement and response within 10 minutes after alert”, Group II as “response within 10-60 minutes after” and Group III as “late response after >60 minutes”. First acknowledgement of the alert, SOFA and APACHE 2 scores; and patient outcomes were analysed.

**Results:** 165 patients recieved alert, however only 33 were diagnozed with sepsis/septic shock on admission, on the other hand, later in course of disease, 112 patients were confirmed to have sepsis. Group I had the lowest mortality compared to others (Table 4).

**Conclusions:** CIS helps in early recognition and intervention of critical disease, thus has an impact in decreasing mortality.In order to further decrease mortality, training is needed to increase compliance to the alert systems.However, septic shock alert protocolized over SIRS criteria seems to lead false positive diagnosis in one third of patients.This reveals the need for revising the sepsis and septic shock definition.

**Table 4 (Abstract P041).** ᅟ

	Group I	Group II	Group III
No of patients	125	18	22
Age (yr) (mean ± SD)	56 ± 14	60 ± 16	44 ± 20
Female/Male (%)	25/75	37/63	33/67
First alert after admission (hr) (median)	5.65	10.23	4.26
SOFA (mean)	8.24	7.81	8.30
APACHE 2 (mean)	25.49	26.19	25.78
Mortality (%)	39.60	51.13	59.09

## P042 Association between previous prescription of βblockers and mortality rate among septic patients: A retrospective observational study

### S. Alsolamy, G. Ghamdi, L. Alswaidan, S. Alharbi, F. Alenezi, Y. Arabi

#### King Saud bin Abdulaziz University for Health Sciences and King Abdullah International Medical Research Center, Riyadh, Saudi Arabia

**Introduction:** Preclinical and clinical studies have investigated the role of β -blockers in sepsis, especially in early sepsis, which characterized by elevated sympathetic response. The purpose of this study was to examine the association between a previous prescription for β -blockers and mortality rate among septic patients.

**Methods:** We conducted a retrospective cohort study from January 1, 2003, to December 31, 2013, in a tertiary care academic medical center. We included all patients admitted to the intensive care unit (ICU) with severe sepsis and septic shock who were > =14 years of age. Patients were defined to have a previous prescription of β -blockers if the prescription was active for the 3 months prior to hospital admission.

**Results:** We identified 4,629 patients with severe sepsis and septic shock, 623 of whom had a previous prescription for β -blockers before hospital admission and 4006 of whom were not previously taking β -blockers. Common medications used by patients were metoprolol (77 %) and carvedilol (13 %). The overall mortality rate was 30 %. Among patients who were previously taking β -blockers, 181 (29.1 %) died in the ICU and 442 (70.9 %) were discharged alive. Of the patients who did not have a previous prescription for β -blockers, 1,231 (30.7 %) died in the ICU and 2,775 (69.3 %) were discharged alive. There was no statistically significant association between patients having a previous prescription for β -blockers and ICU mortality (risk ratio 0.94; 95 % confidence interval [CI] 0.82 to 1.08; p = .39). We further stratified patients on the basis of highest heart rate in the first 24 hours of ICU admission into those with a heart rate >95 beats per minute and those with a heart rate of < =95 beats per minute. There were no statistically significant association between patients who had a previous prescription for β -blockers and ICU mortality in either group (risk ratio 0.91; 95 % CI 0.77 to 1.06; p = 0.22) and (risk ratio 1.095; 95 % CI 0.78 to 0.1.54; p = 0.60) respectively.

**Conclusions:** Our study demonstrated no significant association between previous prescription for β -blockers and ICU mortality. This held true even after further stratification of patients on the basis of highest heart rate in the first 24 hours of ICU admission. This result may be due to lack of effect of β -blockers or short-term action of medication use.

## P043 Recognition and treatment of sepsis on labour ward– teaching & information resources can improve knowledge

### J. Heaton, A. Boyce, L. Nolan, J. Johnston, A. Dukoff-Gordon, A. Dean, A. Molokhia

#### Lewisham and Greenwich NHS Trust, London, UK

**Introduction:** The UK Sepsis Trust aims to raise awareness of sepsis and recommends an evidence based treatment bundle for severe sepsis known as ‘The Sepsis Six’ [1, 2]. An audit was completed on the Obstetric units of Lewisham and Greenwich NHS Trust, UK. We wanted to assess the impact of teaching and posters on improving knowledge.

**Methods:** We gave an anonymous questionnaire to maternity staff to assess baseline knowledge. This tested respondent’s on the diagnostic criteria for systemic inflammatory response syndrome (SIRS), sepsis, severe sepsis and their knowledge of the ‘Sepsis Six’ treatment bundle for severe sepsis [1]. Our interventions consisted of 1:1 teaching and the provision of an information poster. A re-audit was performed in the same units a week later.

**Results:** We initially audited 33 maternity staff and re-audited 27. Those re-audited reported that all staff had received 1:1 teaching and 96 % had seen the leaflet or poster.

SIRS: Initially, 82 % had heard of SIRS, could name on average of only 1.5/6 criteria and only 58 % knew 2 criteria were needed for the diagnosis. This improved to 100 %, 5.2/6 and 81 % respectively.

SEPSIS: 52 % could correctly define sepsis, only 3 % could define severe sepsis with staff able to name on average only 0.8 of the severe sepsis criteria. Their knowledge also improved to 70 %, 63 % and 2.4 respectively.

SEPSIS 6 MANAGEMENT: Staff knew 4.8/6 of the Sepsis Six steps for management and 88 % knew it was required within an hour. There was a slight improvement to 5.5/6 for management and a surprising decrease in initiation of treatment to 85 %.

**Conclusions:** Our maternity staff were initially aware of SIRS and Sepsis and simple interventions required to diagnose and treat patients, however their baseline knowledge of details in diagnosis and management was poor. We demonstrated that simple interventions such as 1:1 teaching, a poster and readily available leaflet can dramatically improve knowledge.

**References**

1. UK Sepsis Trust. Clinical Toolkit. http://sepsistrust.org/clinical-toolkit/ (Accessed 01/11/2015)

2. Daniels R et al. The sepsis six and the severe sepsis resuscitation bundle: a prospective observational cohort study. Emergency Medicine Journal 2011; 28(6): 459-46

## P044 Culture negative sepsis in the ICU – what is unique to this patient population?

### T. Mann Ben Yehudah

#### Assaf Harofeh MC, Beer Yaakov, Israel

**Introduction:** There is paucity of data concerning the group of patients with a clinical syndrome of sepsis, suspected clinical infection and negative cultures regarding disease severity, end organ failure and outcomes. It is also not clear whether patients with only negative blood cultures (BCs) but other positive cultures have a different disease entity than patients who have no growth in all of the cultures taken. Our study attempts to further define true culture negativity and its effect on disease severity and outcomes.

**Methods:** All blood cultures results taken in the Detroit Medical Center ICUs from December 2012 to March 2013 were collected. Any patient who had a negative BC was reviewed. All cultures taken within 10 days of the negative BCs were further reviewed. Patients were then divided into 3 groups: 1.BC positive group- BCs within 10 days of the negative BC were positive. 2. Non BC positive group - other cultures within 10 days of the negative BC were positive. 3. All culture negative. The 3 groups were compared.

**Results:** During the study period, 300 patients had at least one negative blood culture. 132 had all cultures negative, 76 had positive BCs, and 92 had other positive cultures. The BC positive group resembled the negative group in all baseline characteristics. There was a significant difference in the admission scores, with higher SOFA and APACHE2 scores of the BC positive group. SOFA score was higher for the positive group throughout the ICU stay. There was no difference in SIRS in both groups. However, there was more shock, need for vasopressors, renal failure, respiratory failure and neurological alterations in the BC positive group. The BC positive group was more frequently started on antibiotics and had more antibiotic days then the negative group. They also had worse outcomes with a higher mortality in the ICU (40 % in the positive vs. 6.8 % in the negative, p < 0.0001) and in the hospital (47.4 % vs. 9.9 % respectively, p < 0.0001). In a multivariate analysis of ICU death predictors, the only independent predictors were APACHE2 > 25 and positive BC group. Disease severity was also worse in non-blood positive culture group when compared with the negative group; scores were higher, there was more end organ dysfunction, antibiotic treatment was longer and Outcomes were worse.

**Conclusions:** We have shown that the culture negative patients have lower disease severity, end organ failure and better outcomes, and therefore should be considered for shorter antimicrobial treatments and early de-escalation. Culture positivity indicate worse prognosis even for non BCs.

## P045 Organ dysfunction in severe sepsis patients identified in administrative data in Germany, 2007-2013

### C. Fleischmann, D. Thomas-Rueddel, C. Haas, U. Dennler, K. Reinhart

#### Jena University Hopital, Jena, Germany

**Introduction:** In administrative data, severe sepsis cases can be identified by different ICD code abstraction strategies. Comparing those strategies, there is a substantial variability in incidence and mortality of severe sepsis depending on the codes used. To understand which mechanisms depend the precision of case identification, we aimed to investigate coding of organ dysfunction in patients with severe sepsis hospitalized in Germany between 2007-2013 comparing administrative coding with prospective data from a national cohort study.

**Methods:** Severe sepsis patients (>18 y) were identified in a nation-wide database of hospital discharge data (DRG statistics) using ICD-10 codes for I) sepsis + organ dysfunction (explicit coding strategy) and II) infection + organ dysfunction (implicit coding strategy). Explicit sepsis codes included 26 ICD-codes. Infection codes were adapted from Angus et al. (2001, Crit Care Med). Organ dysfunctions were identified by 27 organ failure codes. Septic shock was defined by code R57.2, introduced in 2010. Comparative organ dysfunction data was extracted from a German ICU cohort study (1).

**Results:** Between 2007-2013, we identified I) 941 957 severe sepsis patients using explicit and II) 4 785 511 severe sepsis patients using implicit coding strategies, including 18,2 % and 3,5 % of patients with septic shock, respectively (112 787 patients 2010-2013). Respiratory failure was the leading organ dysfunction coded (56,4 % of explicitly vs. 59,6 % of implicitly identified cases). Renal failure was identified more often when using explicit coding strategies (44,7 % vs. 26,5 %). This was also true for coagulopathy (23,5 % vs. 12,5 %) and metabolic alterations (13,1 vs. 6,1 %). Hypotension was coded in 18,7 % (explicit) and 5,1 % (implicit) of cases. Compared to a prospective cohort of ICU patients with severe sepsis, distribution of organ dysfunctions was similar to the one identified by explicit coding strategies (respiratory failure: 52 %, renal failure: 42,2 %, encephalopathy: 27,7 %, coagulopathy: 22,2 %, metabolic acidosis: 17,8 %, septic shock 50,8 %).

**Conclusions:** Pattern of organ dysfunctions in severe sepsis patients differ depending on the coding strategy used. Hypotension and the direct code for septic shock is coded in a similar percentage of patients, but in much fewer cases compared to a prospective cohort study of ICU patients. The quality of organ dysfunction coding has substantial influence on the accuracy of coding strategies for severe sepsis in administrative data and therefore requires further evaluation.

**Reference**

Engel C et al. Intensive Care Med. 33(4):606-18, 2007

## P046 A comparison of residents’ knowledge regarding; the Surviving Sepsis Campaign 2012 guideline

### O. Suntornlohanakul^1^, B. Khwannimit^2^

#### ^1^Prince of Songkla University, Hat Yai, Thailand; ^2^Division of Critical Care Medicine, Hat Yai, Thailand

**Introduction:** There were improvements in the knowledge about sepsis care and also well-established guidelines. However, the morbidity and mortality of septic patients remains unacceptably high. Our objective is to evaluate the knowledge of residents in different departments regarding the; Surviving Sepsis Campaign (SSC) 2012.

**Methods:** A cross-sectional descriptive study via a 15 question, questionnaire which was distributed to residents in Songklnagarind hospital, a-tertiary referral university teaching hospital in southern Thailand. The interns as well as, the training residents in the Department of Internal medicine, Surgery and Emergency medicine were included in our study.

**Results:** The response rate was 136 (89 %) from 153 residents. The residents included 46 (33 %) interns, 42 (30 %) internal medicine residents, 41 (30 %) surgical residents and 7 (5 %) emergency residents. Regarding the definition of sepsis, severe sepsis and septic shock, only 44 (32.3 %) residents were able to differentiate the severity of sepsis. Internal medicine residents have a significantly higher rate of correct answers than non-medicine and surgical residents (45.2 % vs. 26.6 %, P = 0.03 and 45.2 % vs. 12.2 %, P = 0.001). Only 77 (51 %) residents would measure blood lactate in sepsis patients, and there is no difference in overall knowledge about lactate measurement and interpretation between, internal medicine residents compared with other residents. In fluid resuscitation, all residents (95.6 %) chose normal saline solution as their first choice. However, in respects to the dose of fluid resuscitation, only 28 (20.5 %) residents gave the recommended fluid bolus (30 mL/kg) and internal medicine residents had a higher percentage of correct answer than surgical residents (28.6 % vs.7.32 %, P = 0.01). One hundred and fifteen (85 %) residents and 123 (90 %) residents used appropriate target mean arterial pressure and vasopressors, respectively. Most residents could give antimicrobial agents (73.5 %) and steroid (93.4 %) appropriately in patients with sepsis and septic shock. However, only half of the residents knew the target range of blood sugar control in sepsis patients, and internal medicine residents have a better knowledge than the other residents

**Conclusions:** Our residents’ knowledge about SSC 2012 is not satisfactory. Teaching couple with the learning process of sepsis management should be further provided. Because, an improvement in knowledge will surely decrease morbidity and mortality in sepsis patients.

## P047 Effectiveness of a septic shock bundle to improve outcomes in the ICU

### F. Breckenridge, A. Puxty

#### Glasgow Royal Infirmary, Glasgow, UK

**Introduction:** The formation of the Surviving Sepsis Campaign in 2002 [1] led to the introduction of various sepsis management bundles, with evidence of improved outcome [2]. In 2014 our ICU introduced a quality improvement project to implement our septic shock bundle, encompassing early central line insertion, dynamic fluid boluses, mean arterial pressure >60 mmHg, measuring central venous oxygen saturations, blood cultures and antibiotics. We aimed to investigate if the introduction of the bundle was associated with improved patient outcomes.

**Methods:** A retrospective search of the WardWatcher^TM^ database identified patients admitted to our ICU with septic shock, from 2009-2011 (Group 1) and after introduction of the quality improvement bundle from 2014-2015 (Group 2). Patients with a significant underlying renal or vascular diagnosis were excluded. Acute physiology and chronic health evaluation II scores (APACHE II) and outcome measures including length of ICU stay, duration of cardiovascular support, need for renal replacement therapy (RRT) and ICU survival were noted.

**Results:** A total of 171 patients were included; 88 patients in group 1 and 83 in group 2. Median compliance with all aspects of the bundle was 60 % for group 2. Mortality was 43.6 % in group 1 compared to 38.6 % in group 2 (p = 0.64). Median APACHE II scores were similar and duration of cardiovascular support was not significantly different between the two groups. Length of ICU stay (median [interquartile range]) was longer in group 2 at 6 days [3-12] compared to 3 [1-7] (p < 0.001) however this significance was lost when non-survivors were excluded. There was a significantly lower requirement for RRT amongst ICU survivors in group 2 at 15.7 % compared to 34 % (p = 0.0078).

**Conclusions:** We have shown a significant improvement in rate of RRT associated with implementation of our sepsis resuscitation bundle. Whilst implementation of care bundles has been associated with improved patient outcome, the basis of this relationship remains unclear [2]. Our work fails to demonstrate a significant reduction in mortality however we acknowledge the limitations of a small sample size. Median bundle compliance is higher than in other published work [2] however ongoing quality improvement work may increase this further. We identified a longer length of stay in group 2. This may represent either a longer period of treatment before deciding to change the focus of care to palliation or a faster decline in patients within group 1.

**References**

1. Dellinger et al.: Crit Care Med 2004; 32: 858-73

2. Levy et al.: Intensive Care Med 2010; 36: 222-231

## P048 Dose of norepinephrine in the first 24 hours as a parameter evaluating the effectiveness of treatment in patients with severe sepsis and septic shock

### P. Szturz ^1^, P. Folwarzcny^1^, J. Svancara^2^, R. Kula^1^, P. Sevcik^1^

#### ^1^University Hospital and Faculty of Medicine Ostrava University, Ostrava, Czech Republic; ^2^Institute of Biostatistics and analyses, Masaryk University, Brno, Czech Republic

**Introduction:** Vasopressor therapy is required to sustain life and maintain perfusion in the face of life-threatening hypotension occurring with severe sepsis and septic shock, even when hypovolemia has not yet been resolved. [1] We hypothesized that a dose of norepinephrine administered in the first 24 hours correlates with the outcome of treatment in patients with severe sepsis and septic shock.

**Methods:** We analysed a total of 632 consecutive patients with septic shock (sepsis-induced hypotension persisting despite adequate fluid resuscitation) from the EPOSS database (Data-based Evaluation and Prediction of Outcome in Severe Sepsis), which was developed to monitor and assess the treatment efficacy in patients with severe sepsis and septic shock. Patients were admitted to participating intensive care units (twelve hospitals – seventeen high-volume care units) in the Czech Republic from 1st of January 2011 to 5th of November 2013. The patients were divided into two groups: survivors (n = 316) and nonsurvivors (n = 316).

**Results:** The groups of survivors vs. nonsurvivors were similar in: age 66.0 (39.0; 84.0) vs. 68.0 (34.0;86.0) p = 0.162, men 184 (58.2 %) vs. 190 (60.1 %) p = 0.343, APACHE II score 26.0 (14.0; 39.0) vs. 28.0 (14.0; 40.0) p = 0.565 and the SOFA score 10.0 (4.0 – 16.0) vs. 10.0 (3.0 – 16.0) p = 0.912. Administration of the dose of 30 mL/kg of crystalloids for hypotension or lactate 4 mmol/L (3 hours) and application of vasopressors (6 hours) was performed in both groups, without statistically significant differences. Statistically significant differences between survivors vs. nonsurvivors were found in the parameter “Dose of norepinephrine in the first 24 hours”, 0.211 (0.022 - 1.104) vs. 0.333 (0.039 - 2.333), p < 0.001, OR (95 % IS) 2.009 (1.362; 2.965), p < 0.001

**Conclusions:** We found that a dose of norepinephrine affects the outcome of human septic shock. Early administered and adequate dose of vasopressors is an important predictor of survival, similarly to a suitable initial fluid resuscitation and adequate antimicrobial therapy.

**Reference**

1. Dellinger RP, et al.: 2012 Crit care med 2013; Feb;41(2):580-637

## P049 Norepinephrine or vasopressin + norepinephrine in septic shock. A retrospective series of 39 patients

### L. Caneva^1^, A. Casazza^2^, E. Bellazzi^2^, S. Marra^3^, L. Pagani^3^, M. Vetere^2^, R. Vanzino^2^, D. Ciprandi^2^, R. Preda^2^, R. Boschi^2^, L. Carnevale^2^

#### ^1^Università degli studi di Pavia, scuola di specialità: Anestesia e Rianimazione, pavia, Italy; ^2^UOC Anestesia e Rianimazione Ospedale Civile di Vigevano, AO Pavia, Vigevano, Italy; ^3^Università degli studi di Pavia, Pavia, Italy

**Introduction:** Low-dose vasopressin (VP) recently emerged as a promising therapy for septic shock[1]. The rationale for its use is the relative VP deficiency in patients with septic shock and VP ability to restore vascular tone and blood pressure, reducing the need for cathecolamines[2]; however VP outcome effects in septic patients remain unclear[3-4].

**Methods:** We retrospectively analyzed patients admitted to our general ICU for septic shock in the last 23 months (between 1/2014 and 11/2015) and treated with Norepinephrine (NE) or with the association NE + VP.

Patients were treated with NE after adequate fluid expansion. VP was added (0.02-0.03U/min) in case of MAP < 60 mmHg with NE dosage > =0,4mcg/kg/min. We analyzed severity scores and plasma lactates at ICU admission, mortality in ICU, urinary output during the first 24 hours of vasopressor and need for RRT during ICU stay. Mann-Whitney and Chi-Square tests were used for statistical analysis.

**Results:** 39 patients were enrolled, 15 patients received NE + VP (NV group), 24 received NE (NE group). Overall mortality rate was 46.1 %: 53.3 % in NV and 42 % in NE group respectively (p = 0,47). The need of RRT was greater in NV than in NE group (40vs20%, p = 0,19). Urinary output in the first 24 hours of vasopressor was lower in NV group (0,7vs1 ml/kg/h, p = 0,47). NV group patients had more severe haemodynamic impairment and also worse severity score (SOFA = 11,8vs9,9; p = 0,03), worse renal function (AKIN 3vs2) at ICU admission and higher plasma lactates levels (3,9vs3,6; p = 0,39) NV group had greater incidence of thrombocytopenia (105vs207, p = 0,03).

**Conclusions:** We didn’t find any statistically significant difference between NV and NE groups in ICU mortality, despite NV had a significantly higher predicted mortality according to the SOFA. Renal function impairment wasn’t significantly different in the two groups. The greater incidence of thrombocytopenia (p = 0.03) observed in NV group is in line with other studies.

**References**

[1] Delmas et al, Crit Care April 2005 Vol 9 N2

[2] Landry et al, Circulation1997;95:1122–5

[3] Gordon et al, BMJ Open November 25-2015

[4] Russell et al, NEJM 2008;358:877–87

## P050 Methylene blue effectiveness as contributory treatment in patients with septic shock

### V. Lopez^1^, M. Aguilar Arzapalo^2^, L. Barradas^1^, A. Escalante^1^, J. Gongora^1^, M. Cetina^1^

#### ^1^Hospital O’horan, Mérida, Mexico; ^2^Hospital O’horan, Mérida, Mexico

**Introduction:** Generalized vasodilation with nonresponding hypotension is present in half death cases due to septicaemia. Methylene blue could be used as a valuable complement in refractory hypotension treatment. The aim of this study was to determine the effectiveness of methylene blue as contributory treatment in patients with septic shock

**Methods:** A controlled, randomized, double blinded, clinical trial was performed. 60 patients were divided in two groups. A Group received a single dose of methylene blue calculated 2 mg/kg per body weight diluted in 100 cc of 5 % dextrose infused in 60 min. and C Group, (control) received 100 cc of 5 % dextrose infused in 60 min. Basal measurements of study variables were taken (MBP, lactate, base deficit, central venous saturation and CO2 delta) prior blue methylene administration and every hour afterwards, until MBP >65 mmHg without vasopressor or 72 hours passed after shock began. Data about total noradrenaline dose in mg, length of stay, mechanical ventilation length and mortality was recorded

**Results:** MBP increased progressively first 6 hours after methylene blue infusion in A Group 22 % and C Group 9.2 % (p:< 0.05), steadily until 72 hour follow up. Noradrenaline dose decreased in the first 6 hours, on A Group an 86 %, C Group was 56 % (p:<0.05). Lactate clearance first 6 hours was 62 % in A Group, in contrast with C Group with 33 % clearance (p:<0.05). Mortality at ICU discharge on A Group was 20.0 % and C Group was 36.6 % (p: <0.05) without variation at 21 days

**Conclusions:** Methylene blue is effective as contributory in septic shock treatment.

**References**

1. Edmund S, Kwok M, Daniel H. Use of Methylene Blue in Sepsis: A Systematic Review. Journal of Intensive Care Medicine 2006; 21(6):359-363.

2. Alderton W, Cooper C, Knowles G. Nitric Oxide synthases: structure, function and inhibition. Biochem. J. 2001; 357:593-615.

**Fig. 10 (Abstract P050).**
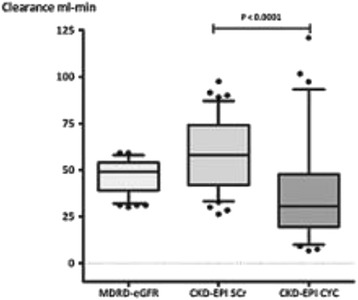
MAP In The Two Groups

## P051 Coagulation disorders in patients with severe sepsis and DIC evaluated with thromboelastometry

### B. Adamik, D. Jakubczyk, A. Kübler

#### Department of Anaesthesiology and Intensive Therapy, Medical University, Wroclaw, Poland

**Introduction:** Coagulation disorders are common in septic patients and constitute a considerable diagnostic and therapeutic challenge in the ICU. The excessive activation of coagulation involves consumption of coagulation factors and platelets which may even lead to the development of disseminated intravascular coagulation (DIC). In the present study we investigated the usefulness of the thromboelastometry method for bedside monitoring of coagulation abnormalities in patients with DIC treated at the ICU.

**Methods:** In the observational study, patients with a diagnosis of severe sepsis and overt DIC on admission to the ICU were included. DIC was recognized using a scoring system based on the criteria proposed by the International Society of Thrombosis and Hemostasis [1]. The results of thromboelastometry tests (ROTEM) performed on the day of admission (day 1) and then daily for the next 3 days were recorded. Clotting time (CT), clot formation time (CFT), maximum clot firmness MCF), alpha angle, lysis index at 60 mi, and maximum lysis index (ML) were measured in EXTEM and INTEM tests.

**Results:** Among 51 patients admitted with severe sepsis, 16 (31 %) had a diagnosis of overt DIC. Out of 16 DIC patients, 5 patients died (NS; DIC score 5 ± 0.4, 6 ± 1.0, 5 ± 0.0, and 6 ± 0.0 points on day 1, 2, 3, and 4 and 11 survived (S; DIC score 5 ± 0.4, 4 ± 1.2, 4 ± 2.1, and 3 ± 1.8 points). On admission, major coagulation abnormalities identified by thromboelastometry, indicating a hypocoagulable pattern of abnormalities, were recorded for patients who did not survive: increased CT (p = 0.005 in EXTEM; p = 0.51 in INTEM) and CFT (p = 0.02 in EXTEM; 0.05 in INTEM), a lower alpha angle (p = 0.008 in EXTEM; p = 0.02 in INTEM), and decreased MCF (p = 0.03 in EXTEM; p = 0.31 in INTEM), in comparison to the results recorded for survivors. Differences in thromboelastometry parameters for groups of NS and S were also observed on the 2nd, 3rd and 4th days. Fibrinolysis was inhibited in nonsurvivors (LI 60 = 100 % and ML = 0 % in both EXTEM and INTEM) during the entire time of observation.

**Conclusions:** Thromboelastometry used as a point-of-care assay made it possible to accurately monitor patients with DIC. The presence of coagulation disorders indicated by thromboelastometry identifies a high-risk subpopulation of critically ill patients.

**Reference**

1. Toh CH, Hoots WK; SSC on Disseminated Intravascular Coagulation of the ISTH. J Thromb Haemost. 2007;5(3):604-6.

## P052 Frequency and outcome of early sepsis-associated coagulopathy

### A. Radford^1^, T. Lee^2^, J. Singer^2^, J. Boyd^2^, D. Fineberg^1^, M. Williams^1^, J. Russell^2^

#### ^1^AKPA, Waltham, USA; ^2^St. Paul’s Hospital, Vancouver, Canada

**Introduction:** The frequency of early (within the first three days of onset) sepsis-associated coagulopathy (SAC) and its association with clinical outcomes varies depending on the definition(s) of coagulopathy. Furthermore, the frequency of SAC may have decreased with more effective use of sepsis bundles (early antibiotics, fluids, and vasopressors). Accordingly, we sought to determine the frequency and outcome of early SAC.

**Methods:** We reviewed all patients admitted to the medical-surgical ICU of St. Paul’s Hospital, a tertiary care hospital in Vancouver, Canada from January 2011 to July 2013. We included patients who met SAC inclusion criteria: sepsis and platelet count less than 150,000 (or a decrease of at least 30 %) and INR greater than 1.2 within the first three days of onset of sepsis. We assessed the presence and severity of SAC and the association of SAC with hospital mortality and need for vasopressors, ventilation and renal replacement therapy (RRT).

**Results:** Of 1,397 ICU admissions 517 had sepsis and of these, 373 (27 % of ICU admissions, 72 % of septic patients) had at least 1 INR and platelet count at any point from day 1 to 3 of ICU admission. 20 to 35 % of septic patients met various criteria for SAC. Presence of SAC at 12 hours or at 24 hours was associated with significantly increased mortality (Odds Ratio (OR) = 2.3 & 1.94 at 12 and 24 hrs respectively) and need for vasopressors (OR = 4.6 & 6.4), ventilation (OR = 1.68 & 2.13) and RRT (OR = 1.71 & 2.94) in both unadjusted and adjusted analyses. Increasing severity of the combination of abnormal INR and platelets was associated with significantly higher mortality and greater need for vasopressors and RRT but not ventilation. Increasingly abnormal INR was associated with increasing mortality in a monotonic, increasing fashion whereas only severe thrombocytopenia (platelets (<80,000) was associated with significantly increased mortality.

**Conclusions:** SAC is common (20 – 35 % of septic patients) and is associated with increased mortality and need for vasopressors, ventilation and renal replacement therapy. Accordingly, there is a need for therapies that decrease the severity of SAC to attempt to decrease mortality and organ dysfunction in sepsis.

## P053 Assessment of coagulopathy in cancer patients with severe sepsis or septic shock. A case-control pilot study

### E. Scarlatescu^1^, D. Tomescu^1^, G. Droc^1^, S. Arama^2^

#### ^1^Fundeni Clinical Institute, Bucharest, Romania; ^2^University of Medicine and Pharmacy “Carol Davila”, Bucharest, Romania

**Introduction:** Hypercoagulability has been described both in cancer patients and also in sepsis, leading to microcirculatory failure and organ dysfunction in the latter. However, this is often overlooked by standard coagulation tests (SCTs).The aim of this pilot study was to compare hemostasis in cancer patients in the early stage of severe sepsis/septic shock to cancer patients without sepsis

**Methods:** Adult patients operated for solid tumors (in the first 30 days after surgery) admitted in the ICU with severe sepsis/septic shock were included in the study group (SG). Patients scheduled for elective surgery due to intra-abdominal or pelvic malignancies were included in the control group (CG). Exclusion criteria were: liver diseases, chronic kidney failure, hematologic diseases, pregnancy, chronic anticoagulant/antiplatelet therapy and blood derivates or procoagulant treatments in the last 7 days. In both groups SCTs, plasma levels of coagulation factors and rotation thromboelastometry (ROTEM®, Germany) were determined in the first 24-36 hours after admission in the ICU (SG) and just before surgery in the CG. The following indices were calculated from the first derivative of the clot firmness curve: Maximum Velocity (MaxVel), Time to Maximum Velocity of clot formation (t-MaxVel) and area under the curve (AUC) [1].

**Results:** After Ethics Committee approval, 32 patients in the SG and 26 patients in the CG were included. Patients in the SG had lower platelet number (p = 0.001), prolonged SCTs (p < 0.001 for PT and aPTT) and lower II,V, VII, X, protein C, S (p < 0.001) and antithrombin III(p = 0.015) levels. In the SG, ROTEM showed delayed activation of hemostasis, prolonged clotting times (p < 0.001) and increased t-MaxVel (p = 0.001). But clot formation was similar in both groups with non-significant differences in maximum clot firmness (MCF), MaxVel and AUC. In the SG patients with APACHEII > =25 had higher MCF (p = 0.026) and AUC (p = 0.003) as compared to patients with APACHE < 25.

**Conclusions:** Our study showed that cancer patients with severe sepsis/septic shock had similar hypercoagulability after a delayed clot initiation as compared to cancer patients without sepsis, despite thrombocytopenia and prolonged SCTs. These abnormalities were only detected by dynamic hemostasis measurements but not with SCTs. For firm conclusions the completion of this pilot study is required.

**Reference**

1. Sorensen et al Journal of Thrombosis and Haemostasis,1,551–558,2003.

## P054 Thromboelastometry in critically ill patients with disseminated intravascular coagulation

### M. Müller, M. Straat, S. S. Zeerleder, N. P. Juffermans

#### Acedemisch Medisch Centrum, Amsterdam, Netherlands

**Introduction:** DIC has a high prevalence among the critically ill, but a specific diagnostic test is lacking. We aimed to assess whether ROTEM is of additional value to discriminate patients with and without DIC and whether it correlates with conventional and experimental markers/tests of DIC.

**Methods:** In ICU patients with a coagulopathy (INR 1.5-3.0), DIC was defined as a score of > = 5 points on the ISTH DIC score scale. Concomitantly with conventional coagulation tests (INR, aPTT, fibrinogen, d-dimer and platelet count) ROTEM was performed. In addition, levels of coagulation factors II, V and VII, antithrombin, protein C activity and protein S were determined. Statistics by Mann Whitney and Spearman’s rho.

**Results:** 23 patients were included of which 13 had overt DIC, the majority was admitted to the ICU due to sepsis. Patients with DIC had lower platelet count and lower levels of fibrinogen, factors II, VII and VIII compared to patients without DIC. Endogenous anticoagulants; antithrombin, protein C and S were also reduced in DIC patients. Thromboelastometry profiles were more hypocoagulable in DIC patients compared to those without DIC: EXTEM CFT: 183 [142-240] in DIC vs 72 [45-177] in non-DIC (p < 0.05), alpha: 60 [51-69] in DIC vs 79 [66-81] in non-DIC(p < 0.01) and MCF: 49 [43-58] in DIC vs. 63 [58-73] in non-DIC patients (p < 0.05). ROTEM EXTEM variables indicating hypocoagulability correlated with DIC scores and with low levels of endogenous anticoagulants (Table 5).

**Conclusions:** In ICU patients with DIC, ROTEM showed hypocoagulable profiles and correlated with DIC score and low levels of endogenous anticoagulants. Thereby, ROTEM may be a useful tool in diagnosing DIC in the critically ill.

**Table 5 (Abstract P054).** Correlation of EXTEM ROTEM with coagulation tests/DIC markers

	CFT	Alpha	MCF
DIC score	0.731**	-0.807**	-0.705**
INR	0.159	-0.162	0.280
Platelet count	-0.787**	0.755**	0.558**
Fibrinogen (g/L)	-0.642**	0.648**	0.680**
Factor II (%)	-0.527*	0.582**	0.497*
Antithrombin (%)	-0.505*	0.497*	0.299
Protein C (%)	-0.626**	0.631**	0.563*
Protein S (%)	-0.390	0.468*	0.258

*p < 0.05,**p < 0.01

## P055 Cessation of a preexisting chronic antiplatelet therapy is associated with increased mortality rates in severe sepsis and septic shock

### C. F. Fuchs, C. S. Scheer, S. W. Wauschkuhn, M. V. Vollmer, K. M. Meissner, S. K. Kuhn, K. H. Hahnenkamp, S. R. Rehberg, M. G. Gründling

#### University Hospital of Greifswald, Greifswald, Germany

**Introduction:** A prior use of antiplatelet agents was associated with a survival benefit in sepsis [1, 2]. But it remains unclear, if chronic antiplatelet therapy should be continued or not during severe sepsis and septic shock. In addition, the subsequent need of red blood cell (RBC) transfusion in patients with continued chronic antiplatelet therapy remains to be determined.

We hypothesized that the continuation of a preexisting antiplatelet medication (aspirin, clopidogrel, dipyridamole) during sepsis therapy reduces mortality rates of severe sepsis and septic shock without increasing RBC transfusions.

**Methods:** We performed a retrospective, single-center cohort study of intensive care (ICU) patients with severe sepsis or septic shock at the University Hospital of Greifswald, Germany, from January 2010 to December 2013. The local ethics committee approved the study (Identifier: BB 133/10) and waived a written informed consent because of the anonymous data collection and the quality saving and observing character of the study. The administration of antiplatelet agents before and during ICU stay, the number of transfused RBC concentrates and mortality rates up to 90 days were registered. Categorical data are represented as percentages and counts.

**Results:** Patients whose preexisting antiplatelet therapy was continued showed increased mortality rates in contrast to patients whose medication was discontinued. The ICU mortality was significantly reduced from 41.6 % (37/89) to 20.4 % (23/113)(OR 0.36; 95 % CI 0.19 – 0.67; p = 0.0013). The hospital mortality was as well reduced from 51.7 % (46/89) to 25.7 % (29/113)(OR 0.32; 95 % CI 0.18 – 0.58; p < 0.001). The mortality at 28 days was reduced from 46.1 % (41/89) to 20.7 % (23/111)(OR 0.31; 95 % CI 0.17 – 0.57; p < 0.001). The remarkable high mortality at 90 days was reduced from 63.6 % (56/88) to 35.5 % (39/110)(OR 0.31; 95 % CI 0.18 – 0.56; p < 0.001).

The need of RBC transfusion was not affected by the administration of antiplatelet agents (p > 0.05).

**Conclusions:** The continuation of a chronic antiplatelet therapy during severe sepsis and septic shock was associated with an improved ICU, hospital, 28- as well as 90-day mortality and did not increase the need of transfusion in critical ill patients.

**References**

1. Sossdorf M et al. Crit Care 17(1):402, 2012

2. Tsai M et al. Intensive Care Med 41(5):806–813, 2015

## P056 Neutrophil Extracellular Traps (NETs) production under hypoxic condition

### N. Yamamoto^1^, M. Ojima^2^, S. Hamaguchi^1^, T. Hirose^2^, Y. Akeda^1^, R. Takegawa^2^, O. Tasaki^3^, T. Shimazu^2^, K. Tomono^1^

#### ^1^Division of Infection Control and Prevention, Osaka University Graduate School of Medicine, Suita, Japan; ^2^Department of Traumatology and Acute Critical Medicine, Osaka University Graduate School of Medicine, Suita, Japan; ^3^Department of Emergency Medicine, Nagasaki University Graduate School of Biomedical Sciences, Nagasaki, Japan

**Introduction:** Neutrophil extracellular traps (NETs) are one of the immune systems to suppress dissemination of infection by the netted chromatin decorated with antibacterial molecules. We reported that NETs were not observed in the abscess, but a lot of NETs appeared after abscess drainage. Since closed wound area is known to be hypoxic, we hypothesized that NETs production was affected by the concentration of oxygen.

**Methods:** Blood samples were obtained from healthy volunteers and neutrophils were purified and stimulated with PMA. Incubation was performed for 4 hours in a CO[sub]2[/sub] incubator or in a box with oxygen scavenger. The specimens were stained with SYTOX Orange and examined by immunohistochemistry. We counted the numbers of NETs and analyzed its rate by Wilcoxon signed rank test.

**Fig. 11 (Abstract P056).**
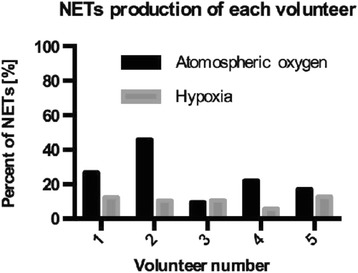
NETs production under hypoxic condition

**Results:** Five volunteers cooperated in this study. NETs were produced under both conditions. NETs amount decreased under hypoxia in the four out of five cases and the average reduction rate was 57.8 % (Fig. 11), although it was not statistically significant (p = 0.13).

**Conclusions:** These results indicated that NETs production might decrease under low concentration of oxygen. Wound drainage may promote NETs production by providing fresh oxygen to the infected area. Further analysis has to be made to clarify the relations between NETs production and oxygen.

## P057 Impact of ultraviolet air sterilizer in intensive care unit room, and clinical outcomes of patients

### E. Gómez-Sánchez, M. Heredia-Rodríguez, E. Álvarez-Fuente, M. Lorenzo-López, E. Gómez-Pesquera, M. Aragón-Camino, P. Liu-Zhu, A. Sánchez-López, A. Hernández-Lozano, M. T. Peláez-Jareño, E. Tamayo

#### Hospital Clínico Universitario de Valladolid, Valladolid, Spain

**Introduction:** Scarce clinical trials have been conducted to evaluate specifically the disinfectant activity of novel ultraviolet (UV) technology in hospital rooms and intensive care units (ICUs). Our objective was to evaluate the impact of an ultraviolet air sterilizer (UVAS) on clinical outcomes of patients in the ICU.

**Methods:** Prospective, comparative, randomized, and non-interventional study involving 1097 patients recovering in the ICU after cardiac surgery. The UVAS (Medixair®) was placed in half of the ICU rooms; while the remaining ones, identical to the others but without it, were used as control.

**Results:** The number of patients presenting gram-positive cocci, mainly S. aureus, was lower in patients with UVAS (12) than without it (4). The incidence of sepsis was lower with UVAS (3.4 % versus 6.7 %). The 30-day in-hospital mortality rate was significantly lower in patients with UVAS (3.8 %) than without it (6.4 %). Independent factors associated with mortality were age (OR 1.046; 95 % CI 1.007–1.087), emergency surgery (OR 4.684, 95 % CI 2.107–10.415), and the absence of the UVAS (OR 2.396; 95 % CI 1.132–5.074).

**Conclusions:** UV-C technology has demonstrated its effectiveness in critical clinical variables of patients in the ICU, such as presence of S. aureus in lungs, incidence of sepsis, or need of >48 hours of mechanical ventilation. Moreover, the UVAS contributed to reduce the 30-day mortality rate.

**References**

1- Tamayo E, Álvarez FJ, Martínez-Rafael B, et al. Ventilator-associated pneumonia is an important risk factor for mortality after major cardiac surgery. J Crit Care 2012;27:18–25

2- Weber DJ, Anderson D, Rutala WA. The role of the surface environment in healthcare associated infections. Curr Opin Infect Dis 2013;26:338–344

3- Hardy KJ, Oppenheim BA, Gossain S, et al. A study of the relationship between environmental contamination with methicillin-resistant Staphylococcus aureus (MRSA) and patients’ acquisition of MRSA. Infect Control Hosp Epidemiol 2006;27:127–132

4- Munoz-Price LS, Ariza-Heredia E, Adams S, et al. Use of UV powder for surveillance to improve environmental cleaning. Infect Control Hosp Epidemiol 2011;32:283–285

5- Rutala WA, Gergen MF, Weber DJ. Room decontamination with UV radiation. Infect Control Hosp Epidemiol 2010;31:1025–1029

## P058 Focus of infection in severe sepsis - comparison of administrative data and prospective cohorts from Germany

### D. O. Thomas-Rüddel, C. Fleischmann, C. Haas, U. Dennler, K. Reinhart

#### Jena University Hospital, Jena, Germany

**Introduction:** Administrative data is increasingly used in epidemiological research, but needs to be validated for this purpose. We therefore wanted to compare reported focus of infection in severe sepsis between administrative data and prospective cohort studies with chart review from Germany.

**Methods:** We identified hospital cases with severe sepsis between 2007-2013 from the “DRG-Statistik”, a database of nearly all German inpatient cases, by the specific ICD-10 code R65.1 (sepsis with organ dysfunction). Focus of infection was abstracted from documented diagnoses for each case. For comparison data on focus of infection was abstracted from a point prevalence study [1] and a prospective cohort study [2] of severe sepsis in German ICU’s. Patients could have more than one focus in all three groups.

**Results:** Relative frequency of the most important foci of infection is reported in Table 6.

**Conclusions:** There are striking differences in the reported foci of infection depending on sampling techniques and method of data abstraction. To improve the usefulness of administrative data for epidemiological research, further evaluation is needed. The surprisingly high incidence of device related severe sepsis warrants a closer look.

**References**

[1] Engel C et al. Intensive Care Med. 33(4):606-18, 2007

[2] Bloos F et al. Crit Care. 18(2):R42, 2014

**Table 6 (Abstract P058).** Frequency of source of infection depending on data source (n.r. not reported)

	Admininstrative data (n = 562,845)	Cohort I [1] (n = 415)	Cohort II [2] (n = 1011)
Respiratory	34 %	63 %	43 %
Abdominal	17 %	25 %	36 %
Genitourinary	26 %	7 %	12 %
Bones/soft tissue	6 %	9 %	7 %
Device related	8 %	n.r.	n.r.

## P059 “Zero CLABSI” – can we get there? Obstacles on the 4 year journey and our strategies to overcome them – experience from an Indian ICU

### V.R. Adora Rao, A. Kar, A. Chakraborty, S. Roy, A. Bandyopadhyay, M. Das

#### Medica Superspecialty Hospital, Kolkata, West Bengal, India

**Introduction:** CLABSI- a dreaded but preventable health care associated infection typically results in prolonged length of stay, morbidity, mortality and economic burden both on the facility and the patient. The aim post intervention was to achieve “Zero CLABSI” by implementing simple evidence based practices using available standard resources.

**Methods:** This Quasi experimental, interventional study enrolled patients (In house and Day Care) with Central Line devices in all units of our 355 bedded hospital from Jan 2012 to Oct 2015. Data collected prospectively by means of active surveillance (audits and HAI tracking sheet) on practices pertaining to Central Lines showed fluctuating CLABSI rates, above the NSHN benchmark 2.79 and consequent Root Cause Analysis identified non conformities with standard practices across all the units in the hospital. In response, a robust program was devised and implemented in the month of Jan 2013, targeting “Zero CLABSI”.

**Results:** see results

**Conclusions:** Achieving and sustaining “Zero CLABSI” is very challenging in view of non modifiable risk factors of the population at risk however strict compliance, continued surveillance and effective team work will facilitate the journey to “Zero or Very Low CLABSI”.

**References**

Device-associated Module BSI, January 2015 (Modified April 2015).

2011 CDC guideline for prevention of intravascular catheter-associated bloodstream infections.

**Table 7 (Abstract P059).** Results

Year	Average Device days	Average CLABSI rate/1000 catheter days	NSHN Benchmark	No of Central Lines Inserted
2012	821	4.2	2.79	968
2013	1069	3.5	2.79	1203
2014	1158	2.4	2.79	1469
2015	1327	1.2	2.79	1692

**Fig. 12 (Abstract P059).**
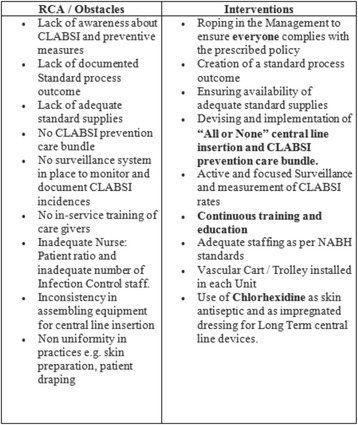
ᅟ

## P060 Novel molecular techniques to identify central venous catheter (CVC) associated blood stream infections (BSIs)

### T. Mann Ben Yehudah^1^, G. Ben Yehudah^1^, M. Salim^2^, N. Kumar^2^, L. Arabi^2^, T. Burger^2^, P. Lephart^2^, E. Toth-martin^3^

#### ^1^Assaf Harofeh MC, Beer Yaakov, Israel; ^2^DMC, Detroit, US,^3^Wayne State University, Detroit, USA

**Introduction:** Currently, CVC related BSIs are categorized as either CRBSI or CLABSI. Both methods have major diagnostic limitations. They both have low sensitivity, particularly among patients already receiving antibiotics. Another limitation is the prolonged time that it takes for diagnostic results to become available, which can adversely impact therapy and clinical outcomes. Molecular techniques to diagnose CRBSI are less established. No study to date has attempted to diagnose CVC related infections in septic ICU population using molecular techniques. Our study tested different molecular techniques designed specifically to diagnose CVC associated infections and compared them to standard CVC tip and blood cultures. We also attempted a new culture method for CVC tips using saline vortexing.

**Methods:** ICU patients with a CVC suspected of having sepsis were included. Blood was extracted for PCR and regular cultures. CVCs were extracted and tips were cultured by the semiquantitive method and also vortexed in saline which was subjected to PCR and regular cultures. PCR was done both by Biofire commercial kit (multiplex PCR) and by RT-PCR using unique primers targeting common ICU organisms. Organism load was quantified using threshold cycle (Ct) with a cutoff of 35 used for a positive result. All methods were compared.

**Results:** A total of 22 blood samples and CVC tips were tested. The Biofire kit and the RT-PCR identified as positive 75 % of positive CVC cultures.The Biofire kit failed to identify positive blood samples.The RT-PCR identified 66 % of them. The Biofire kit did not identify false positive samples. The RT-PCR had a false positive rate of 35 % of negative CVC tips and 67 % of negative blood samples. Blood for standard cultures was drawn prior to antibiotics in 41 % of patients, but almost all patients received antibiotics before blood was drawn for PCR. CVC line tip vortex fluid cultures showed excellent concordance with standard tip cultures.

**Conclusions:** These molecular systems show promise for rapid line sepsis identification. False negativity is probably related to prior antimicrobial treatment. The RT-PCR also had some false positivity possibly reflecting potential treatable organisms missed by standard cultures. The commercial multiplex PCR kit showed lower sensitivity than the RT-PCR.

## P062 Prevention of central line-associated bloodstream infections in intensive care units: An international online survey

### C. Valencia^1^, N. Hammami^1^, S. Blot^2^, J. L. Vincent^3^, M. L. Lambert^1^

#### ^1^Scientific Institute of Public Health (WIV-ISP), Brussels, Belgium; ^2^Ghent University, Ghent, Belgium,^3^Erasme University, Brussels, Belgium

**Introduction:** Central line-associated bloodstream infection (CLABSI), with a attributable mortality of 12 %-25%, is largely preventable. Monitoring processes and outcomes and ensuring compliance with clinical practice guidelines are important components of any intervention aiming at prevention. We documented attitudes and practices in intensive care units in 2015 in order to assess compliance with CLABSI prevention guidelines.

**Methods:** Between June and October 2015, we disseminated an online questionnaire, available in 10 languages, to medical doctors and nurses working in ICUs through national and international ICU societies. We investigated clinical practices related to central line (CL) insertion, maintenance and measurement of CLABSI-related data. Countries were categorized as high, middle, or low income according to World Bank definitions. We computed weighted estimates (% and standard error, SE) for countries providing at least 10 complete responses.

**Results:** 3,407 complete questionnaires were received from 99 countries. Selected results for 13 middle and 27 high-income countries are presented in Table 1. All low income countries provided less than 10 completed responses.

**Conclusions:** Adherence to international guidelines need to be reinforced at ICU level. Priorities in middle income countries should focus on improving 1) full barrier precautions and 2) using chlorhexidine >0.5 % for skin preparation. Reduction of device exposure through daily assessment of CL need remains a priority in both settings. Almost all respondents consider measurement of CLABSI key to quality improvement, however less than a quarter actually report their CLABSI rate. Our study was limited by a non-random sample of ICU doctors and nurses.

**Fig. 13 (Abstract P062).**
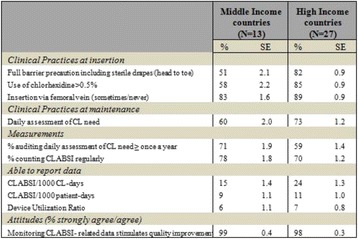
Weighted estimates of practices and attitudes reported by ICU doctors and nurses in high and middle income countries, 2015

## P063 30 days antimicrobial efficacy of non-leaching central venous catheters

### J. Brunke^1^, T. Riemann^2^, I. Roschke^3^

#### ^1^QualityLabs Bt GmbH, Nuremberg, Germany; ^2^B.Braun Melsungen AG, Melsungen, Germany; ^3^Dr. Roschke medical marketing GmbH, Cologne, Germany

**Introduction:** Central venous catheters (CVCs) are widely used in intensive care but increase the incidence of adverse events, especially catheter-related blood stream infections (CRBSIs). Coagulase-negative staphylococci, such as S. epidermidis and Staphylococcus aureus, are the most frequent cause of CRBSIs. These bacteria grow because a biofilm has developed. The inhibition of bacterial growth by antimicrobial catheters helps to prevent surface colonization and improve safety. Certofix® Protect is the third generation of CVC, with non-leaching additives and antimicrobial activity from the catheter tip to the connectors.

**Methods:** The antimicrobial performance (30 days) of non-leaching anti-microbial CVCs on 7 typical CVC-associated infection bacteria was tested with the “Roll-Out” method (Staphylococcus epidermidis, Staphylococcus aureus MRSA and E. coli, Enterococcus faecalis, Pseudomonas aerugionosa, Klebsiella pneumoniae and Candida albicans). After inoculation, washing, incubation at 37 °C, immersion in a minimum medium solution, and a second washing process, the catheter sample was placed on an agar plate and rolled 3 times over the agar plate to transfer surface bound bacteria to the agar medium. After overnight incubation (37 °C), bacterial growth was recorded by photography.

**Results:** The present in-vitro data demonstrate that non-leaching antimicrobial CVCs (e.g. Certofix® Protect, B.BRAUN) exhibit antimicrobial efficacy and prevent biofilm formation from gram-positive, gram-negative bacteria and fungi for up to 30 days. The study was performed in direct comparison with a non-antimicrobial control catheter, on which all 7 test strains were able to grow to an established surface biofilm.

**Conclusions:** This is the first in-vitro study to demonstrate antibacterial surface activity and prevention of biofilm formation with antimicrobial, non-leaching CVCs by using the “Roll-Out” method over a period of 30 days. These results demonstrate that non-leaching antimicrobial CVCs can prevent microbial colonization and infection.

## P064 Efficacy of noble metal alloy-coated catheter in prevention of bacteriuria

### R. Tincu^1^, C. Cobilinschi^1^, D. Tomescu^2^, Z. Ghiorghiu^1^, R. Macovei^1^

#### ^1^Bucharest Clinical Emergency Hospital, Bucharest, Romania; ^2^Fundeni Clinical Institute, Bucharest, Romania

**Introduction:** Urinary tract infections are common problems in patients in ICU with increased mortality, costs and hospitalization. The best way to treat urinary tract infections is to prevent them. The mechanism of action for preventing bacterial adhesion for noble metal alloy-coated urinary catheter is the generation of a galvanic effect. The aim of this study was to evaluate the benefits of noble metal coated-catheter compared to silicone Foley catheter in patients admitted to our Toxicology-Intensive Care Unit for drug poisoning, with short-term catheterization.

**Methods:** We enrolled 120 patients which were randomly assigned to one of the two groups: one group received noble metal alloy-coated catheter (Group 1) and the other one received silicon Foley catheter (Group 2). We excluded all patients with urinary tract pre-contamination. Urine full examination and urine culture was taken at admission and on day 3 of catheterization.

**Results:** The incidence of bacteriuria was 2 % with the noble metal alloy-coated catheter and 6.6 % with the silicone catheter (p < 0.05) after a mean period of 3 days’ catheterization time. Age over 65 years (odds ratio 6.08) was significant risk factors for bacteriuria. The Gram negative bacteria of Escherichia coli and Klebsiella pneumoniae were the most uropathogenic bacteria. We observed significant association between urinary tract infection caused by Escherichia coli and female gender (p < 0.05).

**Conclusions:** Noble metal alloy-coated catheters may decrease the incidence of urinary tract infections compared with silicon ones and in the meantime may lower the need for antibiotics. Also, we noticed that the incidence of bacteriuria increased with age in both groups, but remained lower in noble metal alloy-coated catheter group.

## P065 Predicting bacteremic urinary tract infection in community setting: A prospective observational study

### S. Nimitvilai, K. Jintanapramote, S. Jarupongprapa

#### Nakhonpathom hospital, Nakhonpathom, Thailand

**Introduction:** Urinary tract infection (UTI) is one of the most common source of bacteremia in community setting. Predicting risk of bacteremia can help the clinicians determine the appropriate site of care and therapy. Previous studies were performed on factors associated with bacteremia, however, produced inconclusive results. The aim of this study was to evaluate clinical characteristics to predict bacteremia in community acquired UTI.

**Methods:** This prospective observational study was conducted at Nakhonpathom hospital, a 670-bed tertiary care hospital in Thailand during December 2014 and August 2015. Inclusion criteria were age [>=] 18 years, fever, at least 1 symptom of UTI (dysuria, frequent or urgent urination, flank pain or costovertebral tenderness) and pyuria (defined by a presence of [>=] 5 WBC per high power field in a centrifuged sediment). Patients were excluded if they: (1) had healthcare associated infection; (2) had taken antibiotics during 72 h prior to hospitalization; (3) presented with severe sepsis/septic shock. Clinical factors associated with bacteremia were determined.

**Results:** There were 106 patients. The mean [SD] age was 44 [17] years and 93 % were female. Twenty-two patients (20.8 %) had diabetes melliutus, while 9 (8.5 %) and 4 (3.7 %) had stone and structural abnormality of urinary system, respectively. Bacteremia was found in 16 patients (15 %). [i]E.coli[/i] was the most common organism (13 cases), followed by MSSA (2 cases) and [i]Proteus mirabilis[/i] (1 case). Surprisingly, 6 of 13 isolates (46.2 %) of [i]E.coli[/i] were ESBL producer. Patients who had bacteremia were more likely to have older age (mean [SD] age, 54.0 [12.6] vs 42.3 [16.9] years, p 0.004), diabetes mellitus (40 % vs 16 %, p 0.04), chill (100 % vs 61 %, p 0.01), higher body temperature (mean [SD] temperature, 101.3 [33.6] vs 100.0 [34.0] F, p 0.04), BUN/Cr ratio [>=] 16 (67 % vs 41 %, p 0.07) and CRP [>=] 20 mg/L (92 % vs 61 %, p 0.05). Multivariate analysis of factors with p < 0.1 in univariate analysis showed that BUN/Cr ratio [>=] 16 (OR 4.5, 95%CI 1.1-18.7, p 0.04) and CRP [>=] 20 mg/L (OR 10.8, 95%CI 1.3-93.4, p 0.03) were associated with bacteremia.

**Conclusions:** BUN/Cr ratio and CRP level can be used to guide the probability of bacteremia in community acquired UTI.

**References**

1. Prevalence and predictive features of bacteremic urinary tract infection in emergency department patients. Eur J Clin Microbiol Infect Dis 2007

2. Are blood cultures necessary in the management of women with complicated pyelonephritis? J Infect 2006

## P066 Eight-year analysis of acinetobacter spp. monobacteremia in surgical and medical intensive care units at university hospital in Lithuania

### D. Adukauskiene, D. Valanciene

#### Lithuanian University of Health Sciences, Kaunas, Lithuania

**Introduction:** The aim of study was to analyze characteristics of monobacteremia caused by Acinetobacter spp. and associating factors with mortality in surgical and medical intensive care units.

**Methods:** The retrospective data analysis of patients treated in surgical and medical intensive care units with positive blood culture for Gram-negative rod during 2005-2012 years was carried out.

**Results:** There were found 430 cases of Gram-negative rod monobacteremia, 77 (17.9 %) of them caused by Acinetobacter spp. There was no difference in gender, age, comorbidities, source of bacteremia, length of stay in intensive care unit for Acinetobacter spp. bacteremia (P > 0.05). Primary bacteremia was associated with surgical procedures (OR = 4.677, P = 0.03) and previously treatment in other hospital departments (OR = 1.02, P = 0.04). Acinetobacter spp. strain was associated with high resistance to antibiotics (P > 0.05), as: cefotaxim (n = 75, 97,4 %), ampicillin (n = 74, 96.1 %), cefuroxim and ceftazidim (n = 66, 85.7 %), gentamicin (n = 65, 84.4 %), piperacillin (n = 65, 84.4 %), piperacillin with tazobactam (n = 62, 80.5 %), ciprofloxacin (n = 59, 76.6 %), amikacin (n = 41, 53.2 %), ampicillin with sulbactam (n = 33, 42.9 %). The low resistance to carbapenems (n = 6, 7.8 %, P = 0.03) was estimated. Acinetobacter spp. strains were found to be mostly multi-drug-resistant, as resistant to > = 3 classes of antibiotics (n = 74, 96.1 %, P = 0.02), especially in postoperative patients (n = 37, 92.5 %, OR = 4.042, P = 0.04) and previously treated in other hospital departments (n = 41, 100 %, OR = 4.701, P = 0.03). Overall mortality rate of Acinetobacter spp. bacteremia was 84.4 %. Lethal outcome was associated with mechanical ventilation (n = 65, 100 %, OR = 4.105, P = 0.05), multi-drug-resistant strain (n = 64, 98,5 %, OR = 1.182, P = 0.013), elderly (n = 36, 90 %, OR = 1.662, P = 0.016), resistance to cefalosporins (n = 57, 86.4 %, OR = 2.367, P = 0.04), and alcohol abuse (n = 9, 81.8 %, OR = 3.926, P = 0.05).

**Conclusions:** Acinetobacter spp. monobacteremia usually appears as caused by multi-drug-resistant > = 3 classes of antibiotics strain, but with low resistance to carbapenems, especially in postoperative patients and previously treated in other hospital departments. It has extremely high overall mortality rate. Mortality was associated with elderly, alcohol abuse, multi-drug-resistant strain and resistance to cefalosporins also mechanical ventilation.

## P067 Group A and group B streptococcal infections in intensive care unit – our experience in a tertiary centre

### G Bose, V Lostarakos, B Carr

#### University Hospital North Midlands, Stoke-on-Trent, UK

**Introduction:** To describe the incidence, clinical features and outcome of Group A and Group B streptococcal infections in our Intensive Care Unit (ICU).

**Methods:** Retrospective analysis was conducted on all patients admitted to ICU with Group A and Group B streptococcal infection from 2012 to 2015. Demographic and clinical information were obtained from the Intensive Care National Audit & Research Centre data.

**Results:** 22 cases were identified accounting for 0.8 % of all admissions in our unit. The pathogen was mainly isolated from soft tissue wounds and sputum. Median age of cases was 50 years. Organ support was as defined in the UK CCMDS [1] (Critical Care Minimum Dataset definitions). The table lists the number of patients with respiratory, cardiovascular and renal support, data on length of stay, illness severity as assessed by APACHE 2 score [2] and mortality rates.

**Conclusions:** Both Group A and Group B streptococcal infections were associated with significant morbidity and mortality. The APACHE 2 score appeared to predict the mortality reliably in patients with Group A Streptococcal infections. In patients with Group B Streptococcal infections however observed mortality was significantly higher than the predicted mortality from the APACHE 2 score. ICU and hospital length of stay were broadly comparable for those of the general ICU population over that period (means 216 and 648 hours respectively).

**References**

[1]. Clarke A et al. Critical Care. 2012; 16(Suppl 1): P510.

[2]. Vincent JL et al. Critical Care. 2010; 14(2): 207

**Table 8 (Abstract P067).** Group A and Group B Streptococcal infections

	Group A infection	Group B infection
Number of cases	10	12
Number of organ failure (mean)	3.5	3.0
Invasive mechanical ventilation	90%	83.3%
Cardiovascular support	90%	100%
Renal replacement therapy	20%	None
Length of stay in ICU in hours (median)	311	205.5
Length of stay in Hospital in hours (median)	869	480
APACHE II scores (median)	18.5	9
Probability of mortality (median)	25%	8%
Observed mortality	20%	16.6%

## P068 Improved detection of spontaneous bacterial peritonitis by uritop + tm strip test and inoculation of blood culture bottles with ascitic fluid

### S. Khedher, A. Maaoui, A. Ezzamouri, M. Salem

#### EPS Charles-Nicolle, Bab Saadoun, Tunisia

**Introduction:** The diagnosis of spontaneous bacterial peritonitis (SBP) is usually investigated by cytobacteriologic analysis.we purpose to evaluate the effectiveness of the urinary strips URITOP + TM for rapid diagnosis of SBP and emphasize the utility of inoculation of blood culture bottles withascitic fluid for an accurate identification of bacteria.

**Methods:** Our study included 44 patients with cirrhosis and tense ascites. Immediately, after paracentesis a urine test strips was performed searching visually the presence of leukocytes and nitrites on the ascitic fluid. The reaction between this fluid and the strip induced a color change. The color of the leukocyte reagent area was then compared with the color chart of the bottle and the result was scored in function of the number of crosses. Besides biochemical and bacteriological analysis of the ascitic fluid was done including bedside inoculation of five millimeters of this fluid into aerobic and anaerobic blood culture bottles. Cultures of blood were also obtained from all patients.

**Results:** SBP was diagnosed in 11 of the 44 samples. URITOP + TM test was positive in 8 cases. The reading of leukocytes strips revealed one cross, two crosses and three crosses in 15,4 %, 30,8 % and 15,4 % respectively. Nitrites were present in 69,5 % of cases. Blood and ascitic inoculation were positive in 46,2 % and 53,8 % respectively. The major bacteria isolated were gram-negative bacilli. We found a significant correlation between SBP and visual detection of white cells and nitrites (p < 10-3 for both parameters). A significant correlation was also found between SBP and both ascitic (p = 0,02) and blood (p = 0,006) inoculation. Both visual detection of leukocytes and asitic inoculation in blood culture bottles had a sensibility and a specificity of 100 %.

**Conclusions:** Our study showed that URITOP + TM is a rapid and effective method in the early diagnosis of SBP. Besides the inoculation of blood culture bottles with ascitic fluid improves the detection of SBP and ameliorate consequently the prognosis.

## P069 Increased risk of cellulitis in patients with congestive heart failure: a population based cohort study

### J. Chen

#### Dalin Tzu Chi Hospital, Buddhist Tzu Chi Medical Foundation, Chiayi County, Taiwan

**Introduction:** Although it is well known that lymphedema of limbs is associated with higher possibility of developing cellulitis, the relation between congestive heart failure and cellulitis has never been established.

We investigated the risks of cellulitis in patients hospitalized with congestive heart failure in Taiwan to evaluate whether the risk is higher compared to the general population.

**Methods:** The data from one million National Health Insurance (NHI) beneficiaries were utilized. All adult beneficiaries were followed from January 1, 2005 until December 31, 2012 to identify those who developed cellulitis. Cox regression models were applied to compare the hazards between patients with congestive heart failure and the unexposed group after controlling for selected covariates.

**Results:** We identified 4,842 patients hospitalized with congestive heart failure and 748,701 patients without congestive heart failure. After controlling for age, gender, urbanization level, socioeconomic status, diabetes, hypertension, hyperlipidemia, malignancies, liver cirrhosis, atrial fibrillation, smoking, obesity, peripheral artery disease and Charlson Comorbidity Index score, the adjusted hazard ratio was 1.65 (95 % confidence interval, 1.49¡X1.82) in patients with congestive heart failure.

**Conclusions:** Congestive heart failure may be associated with increased risk of cellulitis.

## P070 Outcomes of severe cellulitis and necrotizing fasciitis in the critically ill

### D. R. Cranendonk^1^, L. A. Van Vught^2^, M. A. Wiewel^2^, O. L. Cremer^3^, J. Horn^4^, M. J. Bonten^5^, M. J. Schultz^4^, T. Van der Poll^2^, W. J. Wiersinga^2^

#### ^1^Academic Medical Center, University of Amsterdam, Amsterdam, Netherlands; ^2^Academic Medical Center, University of Amsterdam, Center for Experimental and Molecular Medicine, Amsterdam, Netherlands; ^3^Department of Intensive Care Medicine, University Medical Center Utrecht, Utrecht, Netherlands; ^4^Academic Medical Center, University of Amsterdam, Department of Intensive Care Medicine, Amsterdam, Netherlands; ^5^University Medical Center Utrecht, Dept. of Medical Microbiology, Utrecht, Netherlands

**Introduction:** The skin is a common site of infection in intensive care units (ICU’s). However, in contrast to necrotizing fasciitis (NF), limited clinical data is available for severe cellulitis in the critically ill. The objective of this study was to describe the clinical presentation of patients admitted to the ICU for severe cellulitis, as compared to patients with NF.

**Methods:** A prospective observational cohort study included all consecutive patients admitted to two tertiary ICU’s between January 2011 and January 2014. From this cohort, patients with possible, probable or definite sepsis diagnosed within 24 h were selected. ICU readmissions during the same hospital admissions and readmissions within 30 days of the first hospital admission were excluded. Using set criteria, sepsis admissions attributed to cellulitis or soft tissue infections were retrospectively recategorized by an independent researcher as admissions for NF, severe (i.e. ICU necessitating) cellulitis, or another primary diagnosis but where cellulitis was present as a comorbidity.

**Results:** Among 2562 ICU sepsis admissions, 31 (1.2 %) patients were admitted for NF and 23 (0.9 %) for severe cellulitis. Cellulitis was diagnosed as a comorbidity in another 25 patients. Comparing severe cellulitis to NF, there were no differences in age, BMI, concurrent infections or length of hospital stay prior to ICU admission. Patients with severe cellulitis had lower rates of shock (30 % vs 61 %, p = 0.03) and need for mechanical ventilation within 24 h after admission (52 % vs 94 %, p = 0.003), but had more chronic comorbidities (87 % vs 55 %, p = 0.02), especially of cardiovascular origin (44 % vs 13 %, p = 0.01). With causative agents identified in 78 % of severe cellulitis patients, S. aureus was the dominant organism (present in 44 %), while 44 % had a Gram-negative infection and 26 % a polymicrobial infection. S. pyogenes was the primary organism in NF (61.3 %). Length of ICU stay was shorter for severe cellulitis patients (3 [2-5] vs 5 [3-11] days, p = 0.01). Hospital- and 90 day mortality were similar in the two groups (26.1 % vs 22.6 %, p > .99; 30.4 % vs 22.6 %, p = 0.54 respectively).

**Conclusions:** Although patients with severe cellulitis were less critically ill on ICU admission, when compared to patients with necrotizing fasciitis their mortality rates appear similar. This study is limited by the relatively small sample size and retrospective diagnosis recategorization.

## P071 Botulism outbreak associated with people who inject drugs (PWIDs) in Scotland.

### M. Day^1^, G. Penrice^2^, K. Roy^3^, P. Robertson^1^, G. Godbole^4^, B. Jones^1^, M. Booth^1^, L. Donaldson^1^

#### ^1^Glasgow Royal Infirmary, Glasgow, UK; ^2^NHS Greater Glasgow and Clyde, Glasgow, UK; ^3^Health Protection Scotland, Glasgow, UK; ^4^Public Health England, London, UK

**Introduction:** Botulism is a rare paralytic illness caused by the action of a potent neurotropic exotoxin produced by Clostridium botulinum. C. botulinum species produce seven serologically distinct toxins, A-G. Human botulism is primarily caused by toxin types A, B or E. In Europe, most wound botulism is now associated with injectable drug use. Symptoms are of descending paralysis, bulbar palsies and respiratory failure.

**Methods:** This is a review of the clinical, microbiological and public health aspects of an outbreak of botulism in Scotland from December 2014 to June 2015. Data was collected via prospective patient interview and clinical data collation.

**Results:** 47 cases of wound botulism were reported: 23 probable, 17 confirmed, 5 discounted and two remain possible; Median age 42, range 24-55 years, 65 % were male. 98 % of cases presented with bulbar palsy but very few with descending limb weakness. 52 % of patients required surgical drainage of wound-related abscesses and 65 % required mechanical ventilation. All patients received 3-doses of trivalent anti-botulinum toxin therapy within 24 hours of clinical diagnosis.

The diagnosis was microbiologically confirmed in 17 cases due to presence of C. botulinum type B neurotoxin detected by molecular assay in tissues and/or by serum bioassay. The C. botulinum strains from 10 cases showed a common profile on fAFLP typing, thereby confirming a link to a common source. All had a recent history of injecting heroin which was obtained either in, or sourced, via Glasgow. The source of infection remains unconfirmed but is thought to be due to contaminated heroin, or cutting agent. There were four deaths, botulism contributing to two.

Police Scotland was closely involved in risk management through increased drug seizures throughout the region, reducing the supply of potentially ‘contaminated’ heroin. Public health measures included; risk communication via distributing postcards widely to PWIDs via drug agencies and needle exchange centres to increase awareness of signs and symptoms, advising smoking drugs as an alternative form of administration and the avoidance of muscle popping.

**Conclusions:** This is the largest outbreak of botulism among PWIDs in Europe. Prompt identification of cases and appropriate clinical management resulted in low mortality amongst the cases. Inter-disciplinary cooperation was integral in management of the outbreak.

## P072 Surveillance of ESBL-producing enterobacteriaceae fecal carriers in the ICU

### Y. Kawano, H. Ishikura

#### Fukuoka University Hospital, Fukuoka, Japan

**Introduction:** Extended-spectrum-beta-lactamase-producing Enterobacteriaceae (ESBL-E) strains are considered to be important, since few antibiotics currently remain active against these bacteria. However, the implications associated with the surveillance of fecal carriers of ESBL-E among ICU patients are unclear. The aim of this study was to determine the efficacy of carrying out stool screening for ESBL-E.

**Methods:** We conducted a retrospective cohort study in the ICU of Fukuoka University Hospital from April 2013 to September 2014. The occurrence of infections or colonization with ESBL-E, the antimicrobial use density (AUD) values, and the clinical outcome were investigated for the described periods, and those parameters were compared between April-December 2013 (phase 1) and January-September 2014 (phase 2). Moreover, we routinely performed stool cultures for ESBL-E in phase 2.

**Results:** Of the 1,245 patients who were admitted to our ICU during the study period, we identified 670 patients (phase 1) and 575 patients (phase 2) respectively. The rates of patients either infected or colonized with ESBL-E were significantly higher in phase 2 than phase 1 (3 % versus 12.3 %, p < 0.05). However, the AUD values for fluoroquinolones (6.8 versus 10.4 defined daily doses/1,000 bed-days, p = 0.27), third-generation cephalosporins (19.9 versus 33.3 defined daily doses/1000 bed-days, p = 0.36), tazobactam/ piperacillin (48 versus 50.7 defined daily doses/1000 bed-days, p = 0.55), and carbapenems (27.3 versus 60.3 defined daily doses/1,000 bed-days, p = 0.06) were not significantly different in each subphase. Moreover ICU mortality (17.9 % versus 16.1 %, p = 0.3) was also not found to differ significantly in the different phases of the study.

**Conclusions:** The findings of this study suggest that the performance of routine stool cultures for ESBL-E in ICU patients had no effectiveness for improving the AUD values and clinical outcome.

**Reference**

1. Mark E. Rupp and Paul D. Fey: Extended Spectrum ƒÀ-Lactamase (ESBL)-Producing Enterobacteriaceae Considerations for Diagnosis, Prevention and Drug Treatment Drugs 2003; 63 (4): 353-365

## P073 Prevalence of ESBL and carbapenemase producing uropathogens in a newly opened hospital in south India

### S. Sreevidya, N. Brahmananda Reddy, P. Muraray Govind, R. Pratheema, J. Devachandran

#### Apollo Speciality Hospital - OMR, Chennai, India

**Introduction:** ESBL and carbapenemase producing gram-negative pathogens pose a major therapeutic challenge to the healthcare providers, both in hospital and in the community. Morbidity, mortality and the cost of health care delivery are all increased by the emergence of resistant pathogens. Therefore, it becomes important to have a good knowledge of local microbial spectrum and sensitivity profile, so appropriate prophylactic antibiotics could be administered. This retrospective review was conducted to assess the prevalence of both ESBL and carbapenemase producers among the uropathogens in a newly opened tertiary care hospital in Chennai, India.

**Methods:** We retrospectively analysed the urine culture reports in our hospital over a one-year period, from Sep 2014 to Oct 2015. Data on the total requests and samples received, number of samples tested positive, organisms isolated and their sensitivity pattern were all collected.

**Results:** During this period, the microbiology lab received a total of 1991 urine samples, 77 % (1533) from outpatients and 23 % (458) from inpatients. 18 % of the total samples received (358/1991) were culture positive (19 % in outpatients and 13 % in in-patients). 21 % of the outpatient and 63 % of the inpatient isolates were ESBL producers. E coli and Klebsiella were the commonest organisms isolated (290/358; 81 %). 92 % of the in-hospital and 73 % of the outpatient ESBL isolate was E. coli. 5 % of the in-hospital isolates were carbapenemase producers.

**Conclusions:** The incidence of ESBL and carbapenemase production among in-hospital uropathogens is very high at 63 % and 5 % respectively. This confirms the global increase in the incidence of ESBLs and carbapenemases. Although carbapenemase production in the community is only 0.6 %, sepsis caused by these isolates is very difficult to treat with high mortality rates. Appropriate antibiotic use, following a deescalation strategy and good antimicrobial stewardship are very important in improving outcomes and preventing the spread of further resistance. Instead of following the general antibiotic guidelines, developing robust local guidelines based on the microbiological data on antibiotic sensitivity of the organisms to guide prophylaxis will be helpful in achieving high rates of clinical cure.

**Reference**

1. Ranjini et al. Community Acquired Infection 2015;2:19-24.

## P074 Prevalence, risk factors and outcomes of methicillin-resistant staphylococcus aureus nasal colonization in critically ill patients

### H. Al-Dorzi, M. Almutairi, B. Alhamadi, A. Crizaldo Toledo, R. Khan, B. Al Raiy, Y. Arabi

#### King Saud bin Abdulaziz University for Health Sciences, Riyadh, Saudi Arabia

**Introduction:** The prevalence of methicillin-resistant Staphylococcus aureus (MRSA) has been increasing in the general population. In this study, we examined the prevalence of MRSA nasal colonization in patients admitted to the intensive care unit (ICU), its risk factors and the association with morbidity and mortality.

**Methods:** This was a retrospective cohort study of all patients who were admitted to the ICU of a tertiary-care hospital in Riyadh, Saudi Arabia in 2011, had nasal swab for MRSA colonization on admission and stayed in the ICU for >48 hours. We calculated the MRSA colonization prevalence and studied the association between MRSA colonization status and patient characteristics and outcomes, including infections due to MRSA and mortality.

**Results:** The cohort included 464 patients with the following characteristics: age = 51.7 ± 21.9 years, APACHE II score = 23.3 ± 8.7, 65.9 % males and mostly admitted from the emergency department (40.7 %) and wards (30.2 %). Thirty-one (6.7 %) patients were MRSA colonized and had similar age, gender distribution, chronic comorbidities and admission APACHE II score compared with the other patients. Patients referred from other hospitals had the highest prevalence (19.4 % versus 6.2 % for the other patients, p = 0.02). On multivariate analysis with age, gender, APACHE II score and source of admission (other hospitals versus other source) being the independent variables, only admission to the ICU from another hospital was associated with MRSA colonization (odds ratio, 4.01; 95 % confidence interval, 1.48-10.90; p = 0.006). Only 3 (0.6 %) patients in the cohort had MRSA bacteremia during ICU stay and all were MRSA colonized (p < 0.001). Furthermore, 14 patients had respiratory tract infection due to MRSA, 9 (64.3 %) of whom were MRSA colonized (p < 0.001). MRSA colonized patients had similar hospital mortality (38.7 % versus 41.8 %, p = 0.74) and length of stay (42 ± 43 versus 55 ± 97 days, p = 0.48) compared with the other patients.

**Conclusions:** MRSA nasal colonization was present in 6.7 % of ICU patients and was associated with MRSA bacteremia and respiratory tract infections, but not with mortality.

## P075 Multidrug-resistant Acinetobacter baumannii infection in intensive care unit patients in a hospital with building construction: Is there an association?

### H. Talaie

#### Toxicological Research Center, Department of Clinical Toxicology, Loghman-Hakim Hospital, Shahid Beheshti University of Medical Sciences, Tehran, Iran

**Introduction:** Here, we determined whether the Acinetobacter baumannii (A. baumannii) class 2 integron is in the soil around our hospital, and if the soil is the cause for increasing numbers of A. baumannii infections in our intensive care unit (ICU) patients. A. baumannii has emerged globally as a significant pathogen in hospitals. It is also present in soil and water. In a previous study, we discovered that the A. baumannii class 2 integron occurred most frequently.

**Methods:** This cross-sectional prospective study was conducted in two ICUs at Loghman-Hakim Hospital, Tehran, Iran, from November 2012 to March 2013. Patient, soil, and hospital environment samples were collected. All isolates were identified using standard bacteriologic and biochemical methods. The phenotypes and genotypes were characterized. The standard disc diffusion method was utilized to test antimicrobial susceptibility. Integron identification was performed by multiplex polymerase chain reaction.

**Results:** There were no A. baumannii bacteria isolated from the hospital environment or soil, although the bacterium was detected around the lip of one patient. Also, a total of 42 A. baumannii clinical strains were isolated from the lower respiratory tract (n = 20), blood (n = 6), urine (n = 8), and wound catheters and nasal swabs (n = 8). 65 % of the isolated species were classified as class 2 integrons. The strains were 100 % resistant to piperacillin, piperacillin-tazobactam, ceftazidime, ceftriaxone, cotrimoxazole, cefepime, ceropenem, and cefotaxime. However, all of the strains were sensitive to polymyxin B

**Conclusions:** Further research is necessary to establish a relationship between A. baumannii and soil, (especially in re¬gards to its bioremediation), as well as to determine its importance in nosocomial infections and outbreaks in the ICU.

## P076 Multidrug-resistant organisms in a Dutch ICU

### J. A. Van Oers, A. Harts, E. Nieuwkoop, P. Vos

#### St. Elisabeth Hospital, Tilburg, Netherlands

**Introduction:** The objective of this study was to evaluate the distribution of multidrug-resistant organisms (MDROs) in a Dutch ICU and compare outcome of MDRO-positive patients versus controls.

**Methods:** In a prospective single-centre study all patients, who spent > 48 hours in the ICU, where screened for MDROs during a 6 month period from September 2014-February 2015. The clinical setting was a 30-bed mixed ICU in the Netherlands. Baseline characteristics were collected. Surveillance screening included rectal cultures on admission and at dismissal and sputum cultures twice weekly. Clinical cultures only on indication. Outcome measures included ventilator time in days, median [range], VAP in patients, n (%), sepsis in patients, n (%), length of stay (LOS) ICU in days, median [range] and 30-day survival in patients, n (%). Statistics included the α -test and Mann-Whitney U-test.

**Results:** 311 patients were included, 173 men, median age 64 years [18-90], median APACHE II 18 [3-73]. A total of 27 MDROs were found in 23 patients. Rectal cultures on admission revealed 17 MDROs in 14 patients, 10 MDROs were cultured in predominately sputum cultures in 9 patients during ICU stay. MDROs included 15 E. coli, 4 Klebsiella spp., E. cloacae, M. morganii, Citrobacter spp., Pseudomonas spp. all 1, 2 S. Pneumoniae, 1 MRSA and 1 VRE. As only 1 patient had a MDRO positive blood culture colonisation appears more frequent than infection. MDRO+ patients were placed in full contact isolation. Ventilator time, VAP, sepsis and LOS ICU were significant higher in MDRO+ patients (Table 9). There was no significant difference in 30-day survival 19 (83 %) versus 220 (76 %), p = 0.50.

**Conclusions:** MDROs were found in a small percentage of the patients. As MDRO+ patients had a longer ventilator time and LOS ICU they may be more sensitive to VAP and sepsis. A large part of MDROs was imported into the ICU. This underscores the importance of our MDRO Prevention program: surveillance cultures, special attention to hand hygiene and washing with 2 % chlorhexidine cloths.

**Table 9 (Abstract P076):** Outcome

	MDRO+	MDRO-	P
Ventilator time	8 (1-33)	3 (0-43)	0.001
VAP	5 (22)	20 (7)	0.012
Sepsis	10 (44)	46 (16)	0.01
LOS ICU	10 (3-54)	5 (2-64)	0.00

## P077 Epidemiology and risk factors of ICU acquired infections caused by multidrug-resistant gram negative bacilli

### Y. Boussarsar, F. Boutouta, S. Kamoun, I. Mezghani, S. Koubaji, A. Ben Souissi, A. Riahi, M. S. Mebazaa

#### Mongi Slim University Hospital, La Marsa, Tunisia

**Introduction:** Multi-drug resistant (MDR) Bacteria are a worldwide threat especially for intensive care unit’s patients (ICU). In Gram Negative Bacilli, the emergence and spread of Extended-spectrum Beta-lactamase (ESBL) and carbapenemase-producing bacteria (CPB) is one of the common causes of morbidity and mortality associated with ICU-acquired infections (ICU AI).

The aim of this study was to determine the epidemiology and risk factors for ESBL and CPB infections as well as the resistance patterns of these bacteria isolated in a Tunisian multidisciplinary intensive care unit.

**Methods:** We conducted a retrospective, case-control study including all patients admitted between January and October 2015. ICU AI were defined as those acquired no less than 48 h after ICU admission. We did not include patients with no bacteriological evidence of infection. Differences in ICU mortality, length of stay and duration of mechanical ventilation (MV) were tested across patients with and without ICU acquired MDR bacteria infections. We also assessed infected sites, most frequent bacteria for each group (ESBL and CPB), and looked for risk factors.

**Results:** In the study period, 184 patients were admitted to the ICU, 67 (34,41 %) had ICU AI. From these 67 patients, 35 had an ESBL infection in 53 isolates, and 21 patients were infected with CPB isolated from 30 cultures. Global mortality in the study period was 44,59 %, the mortality associated to MDR infection was 51,16 %. In fact, mortality of ESBL infections was not so higher than the global one (48,57 %) whereas CPB infection weighed down mortality to reach 71,43 %. In the same way, length of stay was significantly longer in MDR infected patients (18,37 days ± 11.6 STD) than non-MDR infected ones (8,66 ± 4.60 STD, p < 0,001). MV duration was respectively 15,85 days (±12,51 STD) in MDR group and 6,62 days (±5,10 STD, p < 0,001) in the non-MDR infected group. Length of stay and duration of MV were found to be risk factors for acquiring MDR infection in ICU. Ventilator associated pneumonia was the most frequent acquired infection as well in non MDR infected patients as in both ESBL and CPB. Acinetobacter Baumannii was the leading isolate from CPB infection (60 %) followed by Klebsiella Pneumoniae (27 %). Concerning bacilli producing ESBL, Klebsiella Pneumoniae was the most frequently isolated (54,72 %) followed by Escherichia Coli and Enterobacter Cloacae (15,09 % each).

**Conclusions:** The prevalence of ESBL and CPB is increasing day by day in nearly every center of different countries and is responsible for large number of hospital-acquired and nosocomial infections, with very few, if any, therapeutic options. Necessary steps to prevent the spread and emergence of resistance should be taken.

## P078 Improving outcomes of severe infections by multidrug-resistant pathogens with polyclonal IgM-enriched immunoglobulins

### E. Giamarellos-Bourboulis^1^, N. Tziolos^1^, C. Routsi^1^, C. Katsenos^2^, I. Tsangaris^1^, I. Pneumatikos^3^, G. Vlachogiannis^4^, V. Theodorou^3^, A. Prekates^5^, E. Antypa^6^, V. Koulouras^7^, N. Kapravelos^8^, C. Gogos^9^, E. Antoniadou^6^, K. Mandragos^2^, A. Armaganidis^1^

#### ^1^University of Athens, Medical School, Athens, Greece; ^2^Korgialeneion Benakeion Hospital, Athens, Greece; ^3^University of Thrace, Alexandroupolis, Greece; ^4^Aghios Dimitrios Hospital, Thessaloniki, Greece; ^5^Tzaneion Hospital, Piraeus, Greece; ^6^G.Gennimatas General Hospital, Thessaloniki, Greece; ^7^University of Ioannina, Ioannina, Greece; ^8^G.Papanikolaou General Hospital, Thessaloniki, Greece; ^9^University of Patras, Patras, Greece

**Introduction:** The emergence of infections by multidrug-resistant (MDR) Gram-negative bacteria which is accompanied by poor outcome due to inappropriate therapy led to the investigation if adjunctive treatment with Pentaglobin, one IgM-enriched immunoglobulin preparation, may improve survival.

**Methods:** One hundred patients coming from the registry of the Hellenic Sepsis Study Group with microbiologically confirmed severe infections by MDR Gram-negative bacteria acquired after admission to the Intensive Care Unit and treated with Pentaglobin were retrospectively analyzed. Another 100 comparators well-matched for stage of sepsis, infection, appropriateness of antimicrobials and comorbidities coming from the same registry were selected. All-cause 28-day mortality was the primary endpoint.

**Fig. 14 (Abstract P078).**
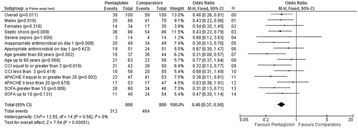
ᅟ

**Results:** 86/14 comparators and 86/14 Pentaglobin-treated cases had severe sepsis/septic shock; 51 and 51 respectively were treated with inappropriate antimicrobials; respective mean Charlson Comordibity Index (CCI) was 2.86 and 2.67. 28-day mortality was 58 % and 39 % respectively (p = 0.011). Post-hoc analysis of subgroups is shown in Fig. 14.

**Conclusions:** Results favor the use of Pentaglobin as an adjunctive to antimicrobial treatment for the management of severe infections caused by MDR Gram-negative bacteria. Most of survival benefit was shown in the subgroups of septic shock, age above 60 years, Charlson’s Comorbidity Index above or equal to 3, APACHE II above or equal to 20 and SOFA score above 10.

## P079 Must change the medical practice in ICU?

### A. R. Robles Caballero, B. Civantos, J. C. Figueira, J. López

#### Hospital Universitario La Paz, Madrid, Spain

**Introduction:** The prevalence of carbapenems resistant bacteria has increased since the first case reported, becoming a worldwide serious concern. This problem specially affects the intensive care units (ICUs).

Usually at ICU, staff and residents develop an exercise of learning and capture of decisions at the patient bedside. Probably this way of work should change.

These are the changes developed in our ICU since January 2014, when were identified the new generation of 11 cases of K.pneumonia OXA 48.

**Methods:** On February, an operative strategic group (GEO) was made and set up with the aim of eliminating and preventing new cabapenems resistant infection cases. The compulsory use of barrier methods was the keystone of all the process. A weekly meeting was performed to talk about the outbreak situation. The main changes carried out by the GEO were:

1) The disposable material (gloves and coats) were put outside of the patient room, no worker could entry without disposable dressing and had to leave it inside the room before exit. 2) Just one physician worked at the patient bedside, dressed with a disposable coat. (The discussions were made outside of the patients rooms) 3) Just one nurse and one attendant worked with each patient. 4) Porters and cleaners were specifically trained stable staff. 5) Colonized patients were grouped on one side of the unit. 6) Microbiology: Rectal samples were collected at admission and on a weekly basis. Direct communication among units with early notice of new cases. 7) Preventive department: Global observation of the compliance (defense standards), recommendation of caution at discharge for at least 7 days. 8) Development of hospital OXA record book.

**Results:** Since the formation of the GEO, number of new carbapenem-resistant cases decreased drastically. Three new cases were reported on February, two cases on March and one on April. Since May, the cases of colonization by carbapenem resistant bacteria within the ICU have been reduced to those cases imported from other units; there have been exceptional cases generated on the unit. In these ones the failure of defense was identified, reported, and measures were taken for further occasions.

**Conclusions:** The growing emergence of multirresistant bacteria is a real threat forcing us to adapt and change our way of working.

Good coordination among hospital workers is the most effective tool as a firewall against these infections. The implementation of the techniques previously described has proven to be an effective fighting technique against carbapenemases producing bacteria.

## P080 Mediterranean spotted fever in an infectious diseases intensive care unit

### A. Silva-Pinto, F. Ceia, A. Sarmento, L. Santos

#### Centro Hospitalar São João, Porto, Portugal

**Introduction:** Mediterranean Spotted Fever (MSF) is considered a benign disease characterized by fever, rash and eschar. However, in some cases it can have severe forms with purpuric rash and multiorganic failure. The objective of this study is to characterize and to delineate predictors of bad outcome in patients hospitalized in an Infectious Diseases Intensive/Intermediate Care Unit (ID-ICU).

**Methods:** We performed a cross-sectional study of adult patients admitted to the ID-ICU with the diagnosis of MSF between 1992 and 2014. The patients were identified in our department database and subsequently the clinical records were consulted. The primary outcome was death and secondary outcomes were its predictors. We used the most appropriate measure of central tendency and distribution and statistical test (significance level 0.05).

**Results:** We include 30 patients. The ICU admission rate was 33 % (comparing to MSF admissions to the Infectious Diseases Department in the same period). There was predominance of women (53 %). The patients were admitted to the ID-ICU with a median disease duration time of 6 days (interquartile range 4). Seven patients (23 %) had comorbidities. At admission, the most common analytic abnormalities were thrombocytopenia (93 %), transaminases elevation (90 %), elevation of the urea and creatinine (73 %), lactate dehydrogenase elevation (73 %), creatine phosphokinase elevation (60 %) and hyponatremia (53 %). The median SAPS II score was 33. Reported MSF complications were consciousness impairment (90 %), renal failure (70 %), pneumonitis (57 %), respiratory failure (53 %) and gastrointestinal bleeding (13 %). Four patients (13 %) needed renal support and five patients (17 %) needed invasive mechanic ventilation.

The mortality rate was 13 % (n = 4). We found a statistical significant association between the SAPS II score and mortality: higher scores are linked to death (Mann Whitney U test). We also found association between higher leukocyte count and prolonged activated partial thromboplastin time (aPTT) at admission with death (Mann Whitney U test). We did not find association between any other analytical abnormality at admission and at death. We also did not find any bad outcome association with patients’ symptoms, physical examination abnormalities, renal or ventilation support (Person’s Qui Squared Test).

**Conclusions:** Although MSF is generally considered a benign disease we had a high ICU admission rate and a considerable mortality rate. We found that the SAPS II score, the leukocytes count and aPTT are predictors of death in patients with MSF admitted to the ICU.

## P081 Clinical features and outcomes of patients with Middle East respiratory syndrome requiring admission to a saudi intensive care unit: A retrospective analysis of 31 cases

### G. Almekhlafi^1^, Y. Sakr^2^

#### ^1^PSMMC, Riyadh, Saudi Arabia; ^2^UNIKLINIKUM JENA, Jena, Germany

**Introduction:** We performed a retrospective study to describe the clinical features and outcomes of a cohort of patients admitted to our ICU with confirmed Middle East Respiratory Syndrome coronavirus (MERS-CoV) infection.

**Methods:** All adult (>18 years old) patients admitted to our 20-bed mixed medico-surgical ICU who were diagnosed with confirmed MERS-CoV between Oct 1, 2012 and May 31, 2014 were included. The diagnosis was confirmed using real-time reverse transcription polymerase chain reaction (RT-PCR) on respiratory samples.

**Results:** During the observation period, 31 patients were identified as having MERS-CoV infection (mean age 59 ± 20 years, 71 % males). The median number of concomitant comorbid conditions was 3 (IQ: 2-4). The initial clinical manifestations of the MERS-CoV infections had occurred at a median of 2 days (IQ: 2-4) prior to hospital admission. Cough and tachypnea were reported in all patients, and bilateral pulmonary infiltrates were evident in 77.4 % of cases. Invasive mechanical ventilation was required in 87.1 % of the patients during the ICU stay, vasopressor therapy in 80.6 %, and renal replacement therapy in 51.6 %. The overall ICU mortality rate was 74.2 %. The median ICU and hospital lengths of stay were 9 (IQ: 4-16) and 12 (IQ: 4-16) days, respectively. Nonsurvivors were older (63 ± 19 vs. 46 ± 20 years, p = 0.48), had greater APACHE II and SOFA scores on admission to the ICU, and were more likely to require invasive mechanical ventilation (95.7 vs. 62.5 %, p = 0.48) and vasopressor therapy (95.7 vs. 37.5 %, p = 0.002).

**Conclusions:** MERS-CoV infection requiring admission to the ICU is associated with high morbidity and mortality rates. Possible risk factors for death include older age, greater severity scores and the need for invasive mechanical ventilation and/or vasopressor therapy.

## P082 The ICU response to a hospital outbreak of Middle East respiratory syndrome coronavirus infection

### H. Al-Dorzi, R. Khan, S. Baharoon, A. Aldawood, A. Matroud, J. Alchin, S. Al Johani, H. Balkhy, Y. Arabi

#### King Saud bin Abdulaziz University for Health Sciences, Riyadh, Saudi Arabia

**Introduction:** The Middle East Respiratory Syndrome coronavirus (MERS-CoV) has caused several hospital outbreaks and frequently leads to severe critical illness. To learn from our experience, we described the response of our intensive care unit (ICU) to a MERS-CoV hospital outbreak.

**Methods:** This observational study was conducted at a 1000-bed tertiary-care hospital in Riyadh, Saudi Arabia which had a MERS-CoV outbreak in Aug-Sep 2015. Our Intensive Care Department covered 5 ICUs with 60 single-bedded rooms. We described qualitatively and, as applicable, quantitatively, the response of intensive care services to the outbreak. The clinical course and outcomes of hospital workers who had MERS were noted.

**Results:** A total of 62 critically ill MERS patients were cohorted in 3 ICUs during the outbreak with a peak census of 27 MERS patients on Aug 25, 2015 and the last new case on Sep 13, 2015. Most patients had multiorgan failure requiring support. Eight hospital employees had MERS requiring ICU admission for a median stay of 28 days: 7 developed acute respiratory distress syndrome, 4 were treated with proning, 4 needed continuous renal replacement therapy and one had extracorporeal membrane oxygenation. The hospital mortality of ICU MERS patients was 53 % (0 % for the hospital employees). All MERS patients were admitted in single negative pressure rooms, which were promptly increased from 14 to 38 rooms. The nurse-to-patient ratio was ~1.2:1. Infection prevention practices were intensified. For example, the consumption of surface disinfectants and hand hygiene gel increased by ~30 % and an average of 17 N95 masks were used per patient/day. Family visits were restricted to 2 hours/day and the ICU physicians communicated with the next of kin by phone daily. During the outbreak, 2 ICU nurses and 1 physician tested positive for MERS-CoV, had mild disease and recovered fully. Although most ICU staff expressed concerns about acquiring MERS, none refused to report to work. However, 27.0 % of nurses and 18.4 % of physicians working in the MERS ICUs had upper respiratory symptoms, but tested negative for MERS-CoV. The total sick leave duration was 138 days for nurses and 30 days for physicians.

**Conclusions:** Our hospital outbreak of MERS-CoV resulted in many patients requiring organ support and prolonged ICU stay. Their mortality rate was high but lower than previously reported. The response to the outbreak required facility and staff management and proper implementation of infection control and prevention practices.

## P083 Middle East respiratory syndrome: Surveillance data analysis

### S. Alsolamy^1^; S. Y. Yousif^1^; B. O. Alotabi^2^; A. S. Alsaawi^1^

#### ^1^King Saud bin Abdulaziz University for Health Sciences and King Abdullah International Medical Research Center, Riyadh, Saudi Arabia; ^2^King Fahad Medical City, Riyadh, Saudi Arabia

**Introduction:** The Middle East Respiratory Syndrome coronavirus (MERS-CoV) is one of the deadliest and least understood emerging pathogens in the 21st century and continues to pose a threat to global public health because of its high fatality rate. This abstract represents an attempt to analyze all publically available global surveillance data of MERS-CoV cases reported by the WHO to date.

**Methods:** We analyzed all outbreak news archives of MERS-CoV between September 23, 2012 and October 1, 2015(1). We included all cases containing data about age and gender (at minimum) and excluded cases reported as clusters. Data on age, gender, comorbidities, healthcare worker, contact with camels, contact with a laboratory confirmed MERS-CoV case, date of symptom onset and date of laboratory confirmation were retrieved and analyzed.

**Results:** A total of 219 outbreak news reports were released by the WHO, 977 cases were include in our study; 68.8 % males: mean (SD) age was 53.2 years (17.9), and 31.2 % females: mean (SD) age was 53.2 years (18.4), where the youngest patient reported was 10 month and the oldest patient reported was 109 year old. Comorbidities were reported in 46.9 %: mean (SD) age was 60.1 (15.4) and 71.8 % male and 28.2 % female. Healthcare workers were reported to be 15.6 %, mean (SD) age was 38.6 (11.3) and 53.3 % male, 46.7 % female and 19.7 % of them had comorbidities. 12.1 % reported with history of camel contact; mean (SD) age was 56.7 (15.1) and 91.7 % male, 8.3 % female and 77.1 % of them had comorbidities. 25.3 % were reported to have contact with a laboratory confirmed MERS case: mean (SD) age was 43.9 (17.0), 63.0 % male 37.0 % female, 36.0 % reported to have comorbidities and 30.5 % were healthcare workers. Date of symptoms onset and date of laboratory confirmation were reported in 86.2 % and 56.5 % respectively. The overall mean (SD) of time from symptoms onset to date of laboratory confirmation was 5.2 days (4.2) (95 % CI, 4.8–5.5).

**Conclusions:** Our study was based on publically available global surveillance data and has the advantages of a large sample demographic description.

**Reference**

1. World Health Organization. Disease outbreak news (MERS-CoV) [http://www.who.int/csr/don/archive/disease/coronavirus_infections/en/]. Last accessed October 1, 2015.

## P085 Use of Taqman array card molecular diagnostics in severe pneumonia: A case series

### J. Ang^1^, M. D. Curran^1^, D. Enoch^1^, V. Navapurkar^1^, A. Conway Morris^2^

#### ^1^Addenbrooke’s Hospital, Cambridge, UK; ^2^University of Cambridge, Cambridge, UK

**Introduction**

Pneumonia remains a common cause of both admission to and deterioration in the intensive care unit. In many cases no organism is identified, resulting in empiric treatment for the presumed causative pathogen. Even when an organism is identified, the delay in obtaining results is typically 48 to 72 hours. Both theses factors can lead to unnecessary antibiotics, and of even greater concern, inappropriate initial therapy. Molecular diagnostic techniques have the potential to address both these problems. We present here three cases which illustrate the utility of molecular diagnostics, using a Taqman array card (TAC).

**Methods**

Three patients admitted to a university hospital general intensive care unit with radiographically confirmed severe pneumonia underwent bronchoscopy and lavage, with the samples being analysed by both conventional microscopy and culture and nucleic acid extraction. The nucleic extract was then placed on a TAC able to detect 48 microbial genes using real time polymerase chain reaction (RT-PCR), directed towards a range of established respiratory pathogens. Results are available with a time to threshold value which gives an indication of the abundance of that gene in the sample.

**Cases**

The cases occurred amongst three adults (age range 19-49) who presented with clinical and radiographic evidence of pneumonia. Two cases were community -acquired with the third being hospital-acquired. The organisms identified by the TAC were *Mycoplasma pneumoniae, Aspergillus spp. Cryptosporidium parvum* and *Pneumocystis jiroveci* (PCJ), with one patient having two organisms detected. *M. pneumoniae* and PCJ were not identified on standard cultures, whilst *Crytposporidum* and *Aspergillus spp* and were both identified on conventional culture/microscopy 5 days after TAC identification. In all three cases, the results of the TAC assay resulted in change in management, with institution of new antimicrobials targeting the organisms identified combined with rationalisation of pre-existing antimicrobial treatments.

**Discussion**

These three cases illustrate how conventional cultures may miss important or unexpected pathogens, or return results late on in the course of the disease. We believe that this technology has the potential to significantly impact on the management of critically ill patients with lung infections, and are currently planning a larger scale evaluation of its use in critical care.

## P086 ‘BUNS’: An investigation protocol improves the ICU management of pneumonia

### R. Sharvill, J. Astin

#### Royal United Hospital, Bath, UK

**Introduction:** Pneumonia is a major cause for ICU admission and mortality. Prompt investigation facilitates tailored antimicrobial strategy, guides management, and aids prognostication. The British Thoracic Society (BTS)[1] suggest specific tests are performed for patients with severe pneumonia: Legionella and pneumococcal urinary antigens, sputum and blood cultures, respiratory viral PCR swabs, and atypical serology testing. We suspected these tests were inconsistently undertaken or delayed in our ICU, and introduced a computerised pneumonia screen, ‘BUNS’, (Blood cultures and viral serology/Urinary antigens/Nasal +/- endotracheal viral swab/Sputum sample) to consistently investigate this condition.

**Methods:** All patients with a primary diagnosis of pneumonia admitted to a UK district hospital ICU over a one-year period up to 31/10/14 were retrospectively reviewed to determine which investigations had been requested within 24 hours of admission. These were compared to the BTS guideline [1]. We subsequently implemented the ‘BUNS pneumonia screen’ within our electronic investigation system. A single click auto-generated request labels for all tests in the BTS guidelines. After implementation and staff education, we repeated the data collection between 1/2/15 and 1/11/15.

**Results:** See Table 10.

**Conclusions:** This study has shown that a computerised auto-generated set of investigation requests, aided by an easily remembered acronym, lead to increased proportion of patients (85 % vs 28 %) receiving prompt and consistent ‘gold standard’ investigations for pneumonia. We believe this reduced duplication and delay in obtaining diagnostic results, thereby improving patient care. We suggest the ‘Bundle of BUNS’ could be easily replicated in other ICUs, and a similar system could be introduced for other presenting conditions.

**Reference**

1. Baudouin S V, Thorax 64:iii1-iii5, 2009

**Table 10 (Abstract P086).** Tests requested within 24 hours of ICU admission

Test requested within 24 hours of admission	Pre-BUNS screen (n = 68)	Post BUNS screen (n = 65)
Blood cultures	48 (71%)	63 (97%)
Blood viral serology	37 (54%)	55 (85%)
Urinary antigens	43 (63%)	56 (86%)
Nasal +/- endotracheal viral swab	15 (22%)	57 (88%)
Sputum MC + S	20 (29%)	59 (91%)
All tests	19 (28%)	55 (85%)

## P087 Pneumonia in patients following secondary peritonitis: epidemiological features and impact on mortality

### M. Heredia-Rodríguez, E. Gómez-Sánchez, M. T. Peláez-Jareño, E. Gómez-Pesquera, M. Lorenzo-López, P. Liu-Zhu, M. Aragón-Camino, A. Hernández-Lozano, A. Sánchez-López, E. Álvarez-Fuente, E. Tamayo

#### Hospital Clínico Universitario de Valladolid, Valladolid, Spain

**Introduction:** Despite the significant impact of nosocomial infections on the morbidity and mortality of patients staying in the intensive care unit (ICU), no study over the past 20 years has focused specifically on pneumonia following secondary peritonitis. Our objective is to determine epidemiological features and in-hospital mortality of pneumonia in this kind of patients.

**Methods:** Prospective observational study involved 418 consecutive patients admitted in the ICU, who had undergone a laparotomy because of a secondary peritonitis. Univariate and multivariate analyses were performed to identify risk factors associated with mortality and development of pneumonia.

**Results:** The incidence of pneumonia following secondary peritonitis was 9.6 %. Risk factors associated with the development of pneumonia were hospital-acquired peritonitis, >48 h of mechanical ventilation, and SOFA score. The onset of pneumonia was late in majority of patients (about 16.8 days after the initiation of the peritonitis), and the etiological microorganisms responsible for it were different than for peritonitis. The 90-day in-hospital mortality rate was 47.5 % of pneumonia patients. Independent factors associated with 30–90-day in-hospital mortality were pneumonia and SOFA score

**Conclusions:** Pneumonia in patients who had undergone a surgery because of a secondary peritonitis, increases the 90-day mortality.

The onset of pneumonia following secondary peritonitis usually is late (16.8 days) and with etiological microorganisms different than those responsable for secondary peritonitis

**References**

1. Jang JY, Lee SH, Shim H, et al. Epidemiology and Microbiology of Secondary Peritonitis Caused by Viscus Perforation: A Single-Center Retrospective Study. Surg Infect (Larchmt) 2015:1–7

2. Mulier S, Penninckx F, Verwaest C, et al. Factors affecting mortality in generalized postoperative peritonitis: multivariate analysis in 96 patients. World J Surg 2003;27:379–384

3. Riché FC, Dray X, Laisné MJ, et al. Factors associated with septic shock and mortality in generalized peritonitis: comparison between community-acquired and postoperative peritonitis. Crit Care 2009;13:99

4. Heyland DK, Cook DJ, Griffith L, et al. The attributable morbidity and mortality of ventilator-associated pneumonia in the critically ill patient. Am J Respir Crit Care Med 1999;159:1249–1256

## P088 The use of the “CURB-65 score” by emergency room clinicians in a large teaching hospital

### J. Patel, C. Kruger

#### Salford Royal Hospital, London, UK

**Introduction:** The “CURB-65 score” is a convenient, well validated tool to assess disease severity and aid in the clinical management of community acquired pneumonia (CAP) [1]. Recent literature however demonstrates that its use is variable and often inaccurate. Failure to use this tool may lead to over or under treatment and may result in inappropriate patient admission thereby increasing healthcare costs [2].

The aim of the initial audit was to determine the frequency of the use of the score in emergency medical care and correlate this with clinical decision-making. The data was analysed and common pitfalls were identified. After the implementation of various tools designed to address these issues a repeat audit was performed to assess for any improvements.

**Methods:** 83 case notes of patients who presented with CAP were retrospectively reviewed between March-May 2014. Based on the data 3 key interventions were implemented. A teaching session regarding the calculation and practical application of the CURB-65 score was delivered to all emergency room clinicians. A CURB-65 pro forma was created and implemented for use in the electronic patient record system used at the hospital. Alongside this a pre-designed set of electronic antibiotic prescription sets were created and incorporated into the electronic prescribing system in use at the audit site. These accountated for allergies, patient weight and renal/hepatic function. A further 50 case notes of patients presenting with a CAP were then retrospectively reviewed during December 2014-Jan 2015 in order to assess the impact of the above interventions.

**Results:** There was a significant increase in the number of case records containing a documented CURB-65 score after the implementation of the above interventions (43.4 % VS 92.0 %). There was an associated improvement in the accuracy of calculated scores (77.8 % VS 92.0 %). In turn, more patients received appropriate antibiotic therapy (86.7 % VS 100 %). Of note, 6 patients prior to the interventions were admitted despite their calculated CURB-65 score being 0-1 without an alternate reason being documented. After the implementation of these interventions no patients were admitted with these scores.

**Conclusions:** Staff education, tools to aid accurate CURB 65 calculation and pre-created antibiotic order sets lead to improvements in the initial assessment and subsequent management of patients presenting with CAP. These changes should be consider for use on a wider scale.

**References**

1. Nadarajan et al, Ir Med Jour (2008)

2. Guo et al, Int J Tuberc Lung Dis (2011)

## P089 Incidence of community acquired pneumonia with viral infection in mechanically ventilated patients in the medical intensive care unit

### J. O’Neal, H. Rhodes, J. Jancik

#### Hennepin County Medical Center, Minneapolis, USA

**Introduction:** Community acquired pneumonia (CAP) guidelines acknowledge respiratory viruses as a cause for CAP, but provide few recommendations regarding diagnosis or treatment due to lack of data [1]. New technology to detect viral pathogens by highly sensitive nucleic amplification tests has allowed for identification of additional viruses through a small sample of respiratory secretions [2]. Our aim is to describe the incidence of viruses in mechanically ventilated patients with CAP in the ICU.

**Methods:** This retrospective chart review includes CAP patients admitted to the ICU and mechanically ventilated between January 2012 and September 2015. Type of virus and screening tool used for detection, confirmed bacterial co-infection, length of stay (LOS) in the ICU and hospital, mechanical ventilation days, and hospital mortality are described.

**Results:** Evaluation of 55 patients with respiratory viral panel screens resulted in 10 patients with positive findings. Seven patients had positive nasopharyngeal swabs and 3 patients had positive BAL findings. Influenza A was the most common and occurred in 3 patients. Rhinovirus occurred in 2 patients, coronavirus occurred in 2 patients with one of these patients also having parainfluenza virus 2. Influenza B, respiratory syncytial virus A, and respiratory syncytial virus B occurred in one patient each. 8 of these 10 patients had confirmed bacterial co-infections. Overall, median ICU LOS was 12.5 days, median mechanical ventilation was 12 days, median hospital LOS was 15.8 days, and mortality was 25 %. Mortality occurred in a patient with influenza A and in a patient with rhinovirus. Both had concomitant bacterial infections.

**Conclusions:** This study demonstrates bacterial and viral co-infection was common. Further research with larger sample size is needed to better characterize respiratory viral infection in the setting of CAP in the ICU.

**References**

1. IDSA/ATS Guidelines. CID. 44: 27-70, 2007.

2. Johnstone, et al. CHEST. 134 (6): 1141-1148, 2008.

## P090 The SAATELLITE Study: Prevention of S aureus Nosocomial Pneumonia (NP) with MEDI4893, a Human Monoclonal Antibody (mAb) Against S aureus

### B. François^1^, P. F. Laterre^2^, P. Eggimann^3^, A. Torres^4^, M. Sánchez^5^, P. F. Dequin^6^, G. L. Bassi^4^, J. Chastre^7^, H. S. Jafri^8^

#### ^1^CHU Dupuytren, Limoges, France; ^2^St Luc University Hospital, Brussels, Belgium; ^3^CHUV, Lausanne, Switzerland; ^4^Hospital Clinic of Barcelona, Barcelona, Spain; ^5^Hospital Clínico San Carlos, Madrid, Spain; ^6^Université François Rabelais and CHU Bretonneau, Tours, France; ^7^Groupe Hospitalier Pitié-Salpêtrière, Paris, France; ^8^MedImmune, Gaithersburg, USA

**Introduction:** Staphylococcus aureus pneumonia, especially within the hospitalized or intensive care unit (ICU) population, is a clinically significant and serious disease that contributes significantly to morbidity and mortality. MEDI4893 is a novel mAb that targets, binds, and functionally inhibits S aureus alpha toxin, a major determinant of S aureus virulence. The use of MEDI4893 to prevent S aureus pneumonia offers a novel paradigm for managing specific high-risk patients. We describe the rationale and design of the SATTELLITE study to investigate the use of MEDI4893 in mechanically-ventilated (MV) subjects.

This study (EudraCT 2014-001097-34) is being conducted through the Innovative Medicines Initiative Joint Undertaking in the EU and is a joint collaboration between Medimmune and the academic partners within the Combatting Bacterial Resistance in Europe (COMBACTE), which is a consortium of experts in the field of antibiotic-resistant bacteria and ventilator-associated pneumonia.

**Methods:** SAATELLITE is a randomized, double-blind, placebo-controlled, dose-ranging Phase 2 study to investigate the efficacy and safety of MEDI4893 in MV subjects. The primary efficacy endpoint is the incidence of NP caused by S aureus through 30 days postdose, proposed as a clinically meaningful, objective and reproduceable endpoint. Safety will be assessed through 360 days post-dose. Subjects will be randomized in a 1:1:1 ratio to receive a single intravenous infusion of Low-dose MEDI4893 or High-dose MEDI4893 or placebo and will be followed through 359 days post dose. Subjects enrolled are ICU patients aged [>=] 18 yrs intubated and on mechanical ventilator, whose lower respiratory tract samples are positive for S aureus, but have not been diagnosed with new onset of pneumonia prior to randomization.

**Results:** N/A

**Conclusions:** The SAATELLITE study represents an innovative pre-emptive therapy approach to prevent S aureus nosocomial pneumonia in MV patients, by utilizing the novel monoclonal antibody MEDI4893, thereby offering a new paradigm in S aureus infection management in high-risk patients.

## P091 Risk factors and microbiological profile for nosocomial infections in trauma patients

### M. Ben Romdhane, Z. Douira, S. Kamoun, M. Bousselmi, A. Ben Souissi, Y. Boussarsar, A. Riahi, M.S. Mebazaa

#### Mongi Slim University Hospital, La Marsa, Tunisia

**Introduction:** Nosocomial infections (NI) were a major cause of morbidity and mortality in trauma patients especially those hospitalized in ICU. The systemic inflammatory response that follows the different lesions is responsible of immune dysfunction promoting the emergence of pathogens and the occurrence of infection. The aim of this study is to identify risk factors for (NI), infection sites and microbiological profile in trauma patients.

**Methods:** We conducted a retrospective study in the Intensive Care Unit of Mongi Slim Hospital La Marsa from January 2015 to September 2015. All Trauma patients hospitalized for more than 48 hours at the ICU were included. Demographic data, type and number of surgeries, use of invasive devices, days under mechanical ventilation, site and number of NI were recorded. We calculate simple frequencies and relative frequencies (percentage) for qualitative variables. We calculate means and standard deviations for quantitative variables.

**Results:** A total of 42 trauma patients were admitted during this 9 month-period and 25 patients developed at least one NI. Infected patients and non-infected one was comparable concerning gravity, and mortality. Infected patients needed more transfusions than non-infected ones (Table 11). Infected patients were mostly associated with head trauma (73.8 %) and chest trauma (76.19 %). Ventilatory acquired pneumonia was the most common NI (73.8 %). The most common pathogens found were Klebsiella Pneumoniae 31 %, E Coli 17 % and Pseudomonas Aeroginosa 8.5 %. Patients with NI have had more invasive devices.

**Conclusions:** The infectious complications are the second leading cause of late mortality after brain injury. Injuries and invasive care promote pathogens penetration in the organism. That’s why, all trauma patients whose stay exceed 48 hours, should benefit from a number of established early precautions to prevent infections especially pneumonia.

**Table 11 (Abstract P091).** Summary of main results

	Infected patients (n = 25)	Non-infected patients (n = 17)
ISS	33	29.9
APACHE II	11.5	7.4
Length of stay (days)	25.0	6.1
Transfusion (%)	70,5	34.9
Mortality (%)	34.1	30.2

## P092 Correlation between percentages of ventilated patients developed vap and use of antimicrobial agents in ICU patients.

### A. Vakalos, V. Avramidis

#### Xanthi General Hospital, Xanthi, Greece

**Introduction:** The aim of our observation retrospective study was to test the hypothesis that a correlation exists between the percentages of ventilated patients developed VAP and the use of main end line antimicrobial agents in ICU patients.

**Methods:** From 2008 to 2012 admitted to our ICU 384 patients. We looked for the percentage of ventilated patients developed VAP as well as the use of the following agents as items per ventilation day per ventilated patient: Imipenem / cilastatin (vial 500 + 500 mg), Meropenem (vial 1000 mg), Tigecyclin (vial 50 mg), Linezoline (bag, 2 mg / ml x 300 ml), Colistin IV (vial 106 IU). Using linear correlation, we looked for linear slope, correlation coefficient (r), coefficient of determination (r2), r and p value.

**Results:** Table 12

**Conclusions:** According to our data, there was no statistically significant correlation detected between the percentage of ventilated patients developed VAP and use of any of the end line antimicrobial agents studied. This may be due to late de-escalation, to use these agents to treat other infections or to prevent spreading and colonization.

**Table 12 (Abstract P092).** Correlation between the percentage of ventilated patients developed VAP and use

	Slope	S. E.	r	r2	L. CI	U.CI	p value
Imipenem	-0.02	0.04	-0.32	0.10	-0.17	0.12	0.388
Meropenem	0.08	0.10	0.43	0.19	-0.24	0.42	0.460
Tigecyclin	0.10	0.06	0.66	0.43	-0.11	0.31	0.223
Linezoline	0.00	0.01	0.13	0.01	-0.03	0.04	0.834
Colistin	0.32	0.20	0.67	0.45	-0.32	0.96	0.210

## P093 A comparison of two ventilator associated pneumonia surveillance techniques

### T. H. Craven^1^, G. Wojcik^1^, K. Kefala^1^, J. McCoubrey^2^, J. Reilly^2^, R. Paterson^3^, D. Inverarity^1^, I. Laurenson^1^, T. S. Walsh^1^

#### ^1^Royal Infirmary of Edinburgh, Edinburgh, UK; ^2^Health Protection Scotland, Glasgow, UK; ^3^Western General Hospital, Edinburgh, UK

**Introduction:** Ventilator associated pneumonia (VAP) rates are a key quality metric and the subjectivity of surveillance has been criticized. The CDC introduced an objective algorithm to diagnose Possible VAP (PVAP) [1]. Local practice uses HELICS methodology to diagnose HVAP. We present preliminary results from a comparison of the techniques, funded by Health Protection Scotland.

**Methods:** Algorithm relevant data were recorded prospectively on all patients admitted to two ICUs for four months from June 2015. An infection surveillance team conducted HELICS surveillance independently. Ethics committee approval was not required. Information guardian approval was received. Non-parametric comparisons were used, p < 0.05 was considered significant.

**Fig. 15 (Abstract P093).**
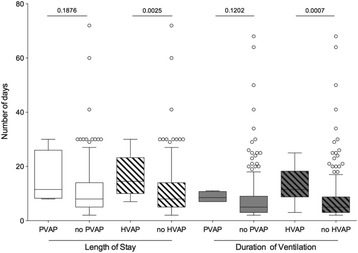
Tukey plot. VAP association with outcome. (p values shown, Mann-Whitney U)

**Results:** 266 patients were ventilated for more than two days. The HVAP and PVAP rates per 1000 at risk ventilation days (total diagnoses) were 5.0 (10) and 2.0 (4) respectively. Concordance between diagnoses was poor (Cohen’s Kappa -0.02 (95 % CI: -0.04 to -0.01)). HVAP was statistically associated with longer stay and ventilation, in contrast to PVAP (Fig. 15). Neither were associated with mortality (HVAP p = 0.22 and PVAP p = 0.13).

**Conclusions:** Our preliminary data illustrate poor concordance between CDC and HELICS algorithms. In contrast to PVAP, HVAP may reflect outcome. The lack of association with PVAP may be the result of a low event rate.

**Reference**

1 Magill SS et al: Developing a new, national approach to surveillance for ventilator-associated events. Am J Crit Care 22: 469–73, 2013

## P094 Lung ultrasound before and after fiberbronchoscopy - modifications may improve ventilator-associated pneumonia diagnosis

### S. Mongodi^1^, B. Bouhemad^2^, A. Orlando^1^, A. Stella^1^, G. Via^1^, G. Iotti^1^, A. Braschi^1^, F. Mojoli^1^

#### ^1^Fondazione IRCCS Policlinico S. Matteo, University of Pavia, Pavia, Italy; ^2^Centre Hospitalier Universitaire Dijon, Dijon, France

**Introduction:** Lung Ultrasound (LUS) is a validated tool for Ventilator-Associated Pneumonia (VAP) diagnosis [1] and monitoring[2]. Dynamic linear/arborescent air-bronchogram is specific for VAP.

**Methods:** 2 patients suspected for VAP were examined before and after fiberbronchoscopy (FBS) by LUS, focusing on subpleural consolidations, lobar/hemilobar consolidations and air-bronchogram. Bronchoalveolar-lavage (BAL) was performed.

**Fig. 16 (Abstract P094).**
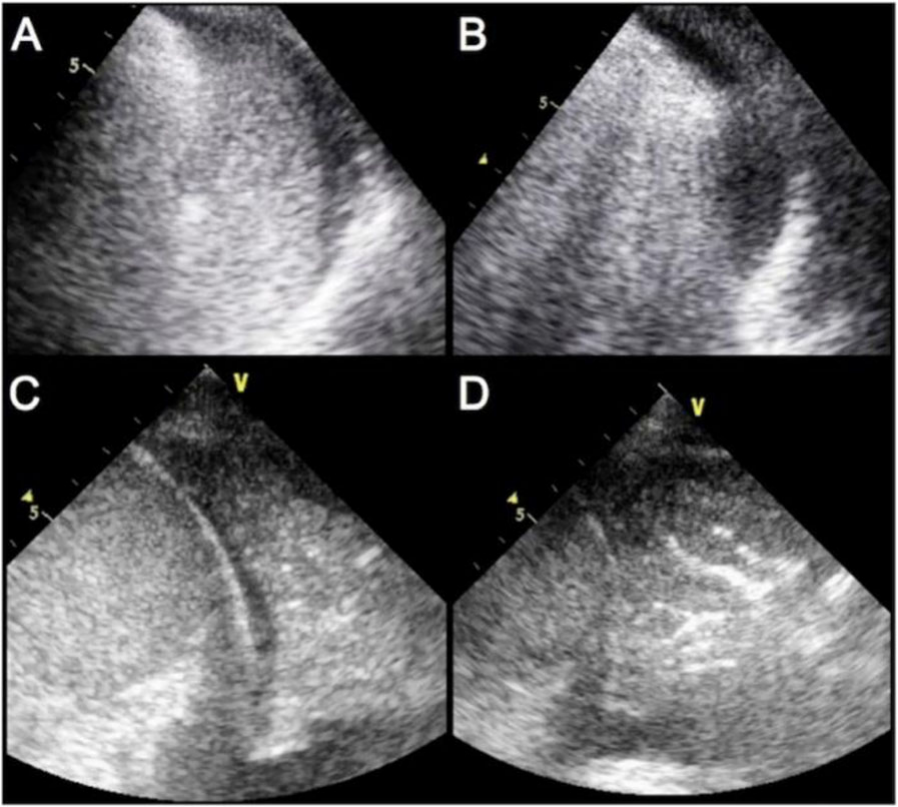
LUS findings before (A,C) and after (B,D) FBS (A-B patient 1, C-D patient 2)

**Results:** Before FBS, LUS pattern wasn’t specific for VAP: both patients had only bilateral consolidations with no air-bronchogram (Fig. 16a,c). After FBS, LUS pattern changed. In patient 1, the tissue-like pattern turned into B-lines (Fig. 16b). BAL was negative. In patient 2, the same tissue-like pattern was visualized after FBS; a dynamic arborescent air-bronchogram appeared (Fig. 16d). BAL had P.Aeruginosa 10(6) CFU/ml.

**Conclusions:** LUS allows early bedside VAP diagnosis. If the pattern is non specific, changes after FBS may be useful: reaeration orients to atelectasis; persistence of complete loss of aeration with dynamic air-bronchogram appearance orients to VAP.

**Reference**

1. Mongodi et al, Chest In press 2.Bouhemad et al, Crit Care Med 38;1:84-92

## P095 Comparing the accuracy of predictors of mortality in ventilator-associated pneumonia

### M. Haliloglu, B. Bilgili, U. Kasapoglu, I. Sayan, M. Süzer Aslan, A. Yalcın, I. Cinel

#### Marmara University Pendik Teaching and Research Hospital, Istanbul, Turkey

**Introduction:** Ventilator-associated pneumonia (VAP) remains an important cause of morbidity and mortality in critically ill patients. Objective tools for prognose assessment may improve treatment strategy. We sought to determine the prognostic value of procalcitonin (PCT), C-reactive protein (CRP), as well as clinical scores [ Sequential Organ Failure Assessment (SOFA), Acute Physiology And Chronic Health Evaluation (APACHE II), Clinical Pulmonary Infection Score (CPIS), Chest Echography and Procalcitonin Score (CEPPIS) ] in critically ill patients who developed VAP.

**Methods:** This retrospective study recruited patients admitted to the Intensive Care Unit of the Anesthesiology Department, Marmara University Hospital (Istanbul, Turkey), from January 2014 to September 2015. Patients’ progress was followed until the 28th day after the diagnosis of VAP, when they were considered survivors. Patients who died before the 28th day were non-survivors. Patients discharged from the ICU before the 28th day were also considered survivors. APACHE II score was assessed during first 24 h of admission; CPIS and CEPPIS score were assessed at the onset of VAP (day 1). SOFA score, serum CRP, serum PCT, pro-BNP were assessed on day 1,4, 7 of VAP diagnosis and were correlated with 28-day survival and mortality.

**Results:** A total of 44 patients were enrolled. Of them, 23 (52.2 %) died before day 28 after VAP diagnosis. The SOFA score in our study was significantly lower in survivors at day 1, 4 and 7 compared with nonsurvivors. In terms of APACHE II score, CPIS and cause of admission; the two groups were comparable. There were no significant difference between the nonsurvivors and survivors in terms of PCT, CRP, leucocyte count at days 1 and 7. However, the leucocyte count, PCT and CRP levels on day 4 were significantly higher in nonsurvivors than survivors. Also pro-BNP levels at days 4 and 7 were significantly higher in the nonsurvivors than survivors.

**Conclusions:** The biomarkers, PCT, CRP and pro-BNP, can predict mortality in VAP, as can SOFA score. Although, CPIS and CEPPIS has been described as useful scoring systems for diagnosis for VAP, they did no differentiate between survivors and nonsurvivors. In compare to CPIS, CEPPIS, a score based on chest ultrasonography and procalcitonin levels, may be a better predictor of diagnosis of VAP.

**References**

1. American journal of respiratory and critical care medicine. 2005;171(4):388-416.

2. American journal of respiratory and critical care medicine. 2002;165(7):867-903.

## P096 Impact of pRBCs transfusion on percentage of ventilated patients developed VAP in ICU patients

### A. Vakalos, V. Avramidis

#### Xanthi General Hospital, Xanthi, Greece

**Introductions:** The aim of our observation retrospective study was to test the hypothesis that a correlation exists between pRBCs transfusion and the percentage of ventilated patients developed VAP (% VP) in our both medical and surgical ICU served in community hospital.

**Methods:** From January 2006 to June 2014 admitted to our ICU 620 patients. We looked for the percentage of ventilated patients developed VAP and the following indexes according pRBCs transfusion per year from 2006 to 2014. Total, per patient, per hospitalization days (HD), per patient ventilated (pts V), per ventilation days (VD) Using linear correlation method, we looked for linear slope, correlation coefficient (r), and coefficient of determination (r2), and by linear regression method using ANOVA test we looked for p value, according % VP and pRBCs transfusion.

**Results:**

**Conclusions:** According to our data, there was no statistically significant correlation detected between the percentage of ventilated patients developed VAP and pRBC transfusion. Our data suggest that even though pRBC transfusion may have an impact on immunosuppression and infection disease developing, the impact on the percentage of ventilated patients developed VAP is not statistically significant.

**Table 13 (Abstract P096).** Correlation between the percentages of ventilated patients developed VAP and pRB

pRBCs	Slope	r	r2	St Error	L. CI	U.CI	p value
Total transfusions	2.487	0.139	0.019	0.685	-13.323	18.296	0.7209
Transfusions per pt	-0.084	-0.375	0.14	0.079	-0.272	0.102	0.3197
Transfusions per H.D.	-0.003	-0.265	0.07	0.005	-0.016	0.008	0.4902
Transfusions per Pt V.	-0.085	-0.33	0.123	0.085	-0.288	0.118	0.3546
Transfusions per V. Day	-0.008	-0.409	0.167	0.007	-0.025	0.008	0.2737

## P097 The impact of a series of interventions on the rate of ventilator associated pneumonia in a large teaching hospital

### H. E. Ellis, K. Bauchmuller, D. Miller, A. Temple

#### Sheffield Teaching Hospital NHS Foundation Trust, Sheffield, UK

**Introductions:** Ventilator associated pneumonia (VAP) has a reported incidence of between 9 – 27 % and a strong association with increased ICU length of stay and mortality[1]. Current evidence suggests that the application of a care ‘bundle’ may reduce the rate of VAP [2]. We aimed to assess the effects of a sequential multi-faceted care bundle and audit program on VAP rates in our institution.

**Methods:** The existing care bundle (head up positioning, daily sedation holds) was supplemented by a 3-step process of interventions between December 2008 and October 2015, each accompanied by a dedicated staff education initiative: 1) Guidance on stress ulcer prophylaxis, changing of respiratory circuitry and sedation holds; 2) Introduction of a new endotracheal tube with continuous subglottic suction and cuff pressure monitoring; 3) New oral hygiene guidance. Data was analysed using MS excel and a statistical process control chart designed for non-conformity attribute data with an unequal area of opportunity. Values are expressed as mean and upper control limits (UCLu).

**Fig. 17 (Abstract P097).**
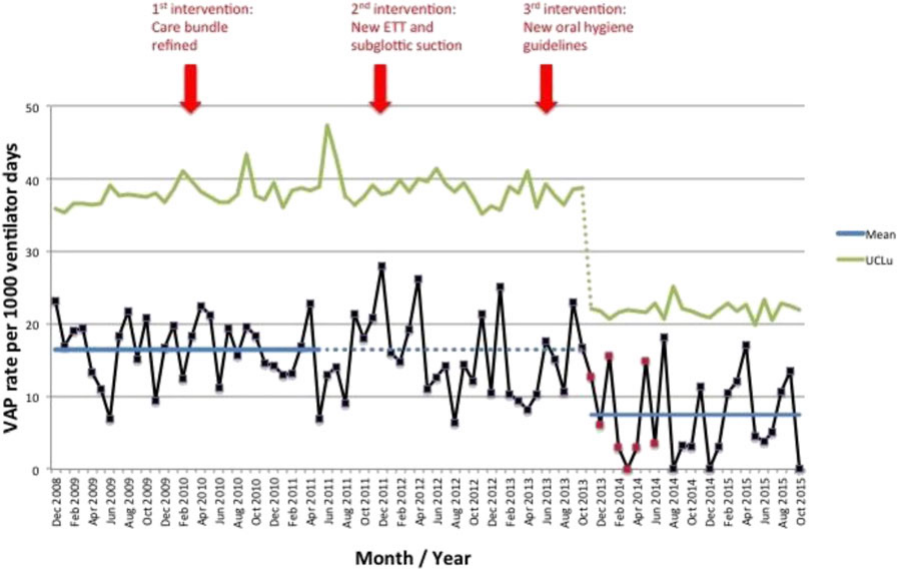
U chart: monthly VAP rates

**Results:** A total number of 26480 consecutive ventilator days were assessed. Monthly VAP rates remained stable around a constant mean of 16.45 per 1000 ventilator days between December 2008 and September 2013. Following introduction of oral hygiene guidance, a statistically significant shift in process occurred identifying a special cause. This represents a reduction of monthly VAP rate to 7.44 per 1000 ventilator days (Fig. 17).

**Conclusions:** Introduction of a multidisciplinary stepwise VAP prevention initiative led to a significant reduction in mean monthly VAP rates over time.

**References**

1. Hunter JD. BMJ 344:e3325, 2012.

2. Morris AC et al. Crit Care Med 39(10):2218-24, 2011

## P098 The EVADE study: Prevention of Nosocomial Pneumonia (NP) caused by P aeruginosa with MEDI3902, a Novel Bispecific Monoclonal Antibody, against P aeruginosa virulence factors

### J. Chastre ^1^, B. François^2^, A. Torres^3^, C. E. Luyt^1^, M. Sánchez^4^, M. Singer^5^, H. S. Jafri^6^

#### ^1^Groupe Hospitalier Pitié-Salpêtrière, Paris, France; ^2^CHU Dupuytren, Limoges, France; ^3^Hospital Clinic of Barcelona, Barcelona, Spain; ^4^Hospital Clínico San Carlos, Madrid, Spain; ^5^University College, London, UK; ^6^MedImmune, Gaithersburg, USA

**Introductions:** Pseudomonas aeruginosa is one of the leading causes of nosocomial infections in critically ill patients. The organism produces an array of factors that contribute to its virulence. Two such virulence factors are the Psl exopolysaccharide, which contributes to bacterial persistence, and the PcrV protein which contributes to the virulence of the organism. MEDI3902 is a novel bivalent, bispecific mAb that selectively binds to both factors, thereby inhibiting the cytotoxicity and immune evasion properties of the pathogen. Psl and PcrV target expression is independent of antibiotic susceptibility status; therefore, MEDI3902 also has the potential to be active against multi-drug resistant strains of P aeruginosa. MEDI3902 is being developed for the prevention of P aeruginosa pneumonia. We describe the rationale and design of the EVADE study to investigate the use of MEDI3902 in mechanically-ventilated (MV) subjects.

This study is being conducted through the Innovative Medicines Initiative Joint Undertaking in the EU and is a joint collaboration between Medimmune and the academic partners within the Combatting Bacterial Resistance in Europe – Molecules against Gram Negative Infections (COMBACTE-MAGNET), which is a consortium of experts in the field of antibiotic-resistant bacteria and ventilator-associated pneumonia.

**Methods:** EVADE is a randomized, double-blind, placebo-controlled, dose-ranging study to determine the efficacy, safety, and pharmacokinetic responses to MEDI3902 in adult ICU patients requiring MV and who are colonised with P aeruginosa in the lower respiratory tract (LRT). The primary efficacy endpoint is the incidence of NP caused by P aeruginosa, proposed as a clinically meaningful, objective and reproducible endpoint. The safety of MEDI3902 will be assessed through 49 days postdose. Subjects included in the study are ICU patients aged ≥18 yrs, intubated and on MV; have LRT sample positive for P aeruginosa, but have not been diagnosed with new onset of pneumonia prior to randomization.

**Conclusions:** The EVADE study represents an innovative pre-emptive therapy approach to prevent P aeruginosa nosocomial pneumonia in MV patients by utilizing the novel mAb, MEDI3902, thereby offering a new paradigm in P aeruginosa infection management in high-risk patients.

## P099 Short-term inhaled colistin adjunctive therapy for ventilator-associated pneumonia

### Y. Nassar, M. S. Ayad

#### Cairo University, Giza, Egypt

**Introductions:** Ventilator-associated pneumonia (VAP) is an important cause of prolonged intensive-care stay and mortality, particularly with the emergence of multi-drug resistant gram-negative bacteria.

The aim of this study is to investigate the role of inhaled colistin on the outcome of patients diagnosed with gram-negative VAP.

**Methods:** We recruited all patients with a confirmed gram-negative culture and sensitivity taken from Endotracheal (ETT) aspirates after > = 48 hrs of mechanical ventilation and pneumonia clinical criteria: radiological infiltrates, fever, leucocutosis or leucopenia, purulent secretions. Eighty five were randomized to enter either the study group of inhaled colistin (3x 106 IU/day, for 5 days) as an adjunctive therapy to conventional treatment of VAP (n = 52 pts) or enter the control group of conventional therapy only (n = 33 pts). Comparison included microbiological outcome (Day 6 ETT aspirate culture and sensitivity) as well as clinical outcome, clinical pulmonary infection score- CPIS.

**Results:** There was a higher organism clearance rate in the colistin compared to the control group (p = 0.02). Multi-drug resistant organisms were observed in [26 (50 %) vs 13 (39.4 %), p = 0.79 ] of the Colistin and Control groups respectively. There was a higher clearance rate of MDR organisms [21/26(81 %) vs 3/13(23 %), p = 0.0005] in Colistin vs. Control groups respectively.

There was statistically significant difference in (CPIS) between both groups. A score >6 represented ( 21.2 % vs 45.5 % , p = 0.01) in Colistin vs control group respectively. A smaller number of patients required mechanical ventilation > 15 days in the Colistin vs control group, (p = 0.013). The ICU mortality was (40 % vs 69.7 %, p = 0.008) in Colistin vs control group respectively. No significant change of renal function or occurrence of bronchospasm were observed between both groups.

**Conclusions:** Short duration of adjunctive inhaled colistin shows a significant clearance of the initial organisms, including multi-drug resistant groups, in patients with gram negative ventilator-associated pneumonia. This line of treatment shows a significant decrease in the duration of mechanical ventilation and ICU mortality when compared with conventional therapy, with no significant added adverse drug reactions. Adjunctive inhaled colistin is safe and effective as an additive therapy in patients with gram-negative ventilator-associated pneumonia.

## P100 Effect of aerosolised colistin on weaning from mechanical ventilation

### A. Trifi, S. Abdellatif, F. Daly, R. Nasri, S. Ben Lakhal

#### University hospital Center of La Rabta, Tunis, Tunisia

**Introductions:** Ventilator-associated pneumonia (VAP) due to multidrug-resistant (MDR), gram-negative bacteria (GNB) is responsible for a prolongation of ventilation days. Several recent studies focused in Aerosolized polymyxin emphasize the potential benefit of this modality. Indeed, by increasing bactericidal activity in situ, this modality led to faster resolution of clinical signs, and perhaps, accelerated weaning from mechanical ventilation (MV). The purpose was to determine whether aerosolized colistin (AS) would accelerate weaning from MV in VAP due to MDR GNB.

**Methods:** Prospective randomized trial from April 2013-April 2015. Included were patients who have MV over 48 h and developed a VAP defined as a CPIS (Clinical Pulmonary Infection Score) > 6. Exclusion criteria were septic shock and / or bacteremia. Included patients were divided into two randomized groups. The first group received colistin in AS as 4 million units (MU) by nebulisation 3 times per 24 h. The second group received colistin in IV as a loading dose of 9 MU followed by 4.5MU two times per 24 h. Colistin was given for 14 days or until extubation. Patients were followed for 28 days.

Outcome Measures: primary: clinical cure defined as pathogen eradication and improvement in oxygenation but mainly weaning from MV. Secondary: length of stay and 28-day mortality.

Ethical approval of the protocol was obtained from the institutional review board

**Results:** a total of 133 patients were analyzed. The cure rates were similar (71.2 % vs 68.6 %, p = 0.85). AS administration was superior in improvement of the PaO2/FiO2 ratio during evolution. Also, it was faster in pathogen eradication (p = 0.04). In survivors, weaning of ventilation was significantly earlier in AS group: 13.8 vs 19 d (p = 0.01). No difference for secondary outcome measures (Table 14)

**Conclusions:** In critically ill patients with VAP due to MDR GNB, AS colistin facilitate weaning from MV. This modality was superior in improvement of oxygenation and the time required for pathogen eradication.

**Table 14 (Abstract P100).** Outcome’s variables in both groups

	AS colistin group (n = 66)	IV colistin group (n = 67)	P value
Length of stay; days: mean+/- SD (median)	26+/-18 (20)	27+/-19 (21)	0.84
Duration of MV : mean+/-SD (median)	14.28+/-7 (13)	17+/-11 (15)	0.08
Duration of MV in survivors: mean+/-SD (median)	13.8+/-8 (12)	19+/-11 (17)	0.01
All-cause 28-day Mortality (%)	24.20%	26.80%	0.8

## P101 Septic shock is an independent risk factor for colistin-induced severe acute kidney injury: a retrospective cohort study

### B. Bilgili, M. Haliloglu, F. Gul, I. Cinel

#### Marmara University Pendik Teaching and Research Hospital, Istanbul, Turkey

**Introductions:** Colistin, a last-line therapeutic agent for the treatment of multidrug-resistance gram-negative infections, is limited in its use due to nephrotoxicity. This retrospective cohort study evaluates risk factors for colistin-induced severe acute kidney injury (AKI) in critically ill patients.

**Methods:** Patients admitted to a university hospital ICU, were without pre-existing kidney injury, and received colistin therapy 72 or more hours were included. Patient demographics, the source of infection and outcome were collected. AKI was evaluated by using the RIFLE criteria. Risk factors for the development of AKI were analyzed via logistic regression analysis.

**Results:** One-hundred and two patients were included in the study. The overall incidence of AKI was 77,5 % (n = 79), and that of severe AKI was 34,3 % (n = 35). On univariate analysis, age and septic shock correlated significantly with severe AKI patients (p = 0,042, p = 0,001, respectively). By multivariate logistic regression analysis, septic shock was the only independent risk factor for colistin-induced severe AKI (OR 8,580 [1,868 – 39,417]; p = 0,006).

**Conclusions:** A high incidence of colistin-induced AKI defined by RIFLE criteria is observed in critically ill patients. Septic shock is the only variable independently associated with severe AKI induced by colistin therapy.

**References**

1) Li J, Nation RL, Milne RW, Turnidge JD, Coulthard K: Evaluation of colistin as an agent against multi-resistant Gram-negative bacteria. International journal of antimicrobial agents 2005, 25(1):11-25.

2) Kim J, Lee KH, Yoo S, Pai H: Clinical characteristics and risk factors of colistin-induced nephrotoxicity. International journal of antimicrobial agents 2009, 34(5):434-438.

3) Rocco M, Montini L, Alessandri E, Venditti M, Laderchi A, De Pascale G, Raponi G, Vitale M, Pietropaoli P, Antonelli M: Risk factors for acute kidney injury in critically ill patients receiving high intravenous doses of colistin methanesulfonate and/or other nephrotoxic antibiotics: a retrospective cohort study. Critical care (London, England) 2013, 17(4):R174.

## P102 Nosocomial pneumonia - emphasis on inhaled tobramycin

### A. Kuzovlev^1^, A. Shabanov^2^, S. Polovnikov^3^, V. Moroz^1^

#### ^1^V.A. Negovsky Research Institute of General Reanimatology, Moscow, Russia; ^2^N.V. Sklifosofsky Institute of Emergency Medicine, Moscow, Russia;^3^NN Burdenko Main Military Hospital, Moscow, Russia

**Introductions:** Inhaled antibiotics as an adjunct to systemic antibiotics present as a significant treatment modality in ICU patients with nosocomial pneumonia (NP). The aim of this study was to investigate into the efficacy of inhaled tobramycin (IT) as an adjunct to systemic antibiotics in the treatment of NP in sepsis.

**Methods:** Sixty ICU ventilated septic patients with NP were enrolled in the retrospective study (all male, 58.4 ± 6.3 years old; primary reason for ICU stay - intraabdominal infections (80 %), mediastinitis (13 %), other (9 %)). Diagnosis of NP was made according to the standard clinical and CPIS criteria. Associations of multiresistant gram-negative bacteria (sensitive to tobramycin) were detected in bronchoalveolar lavage (BAL) of all patients. Patients were randomized into two groups: IT (n = 30), addition of IT to systemic antibiotics (carbapenems, aminoglycosides, protected penicillins); and no IT (n = 30), shift of systemic antibiotics according to sensitivity. Groups were comparable in APACHE II and CPIS scores. Inhaled tobramycin was administered 300 mg twice daily via nebulizer. NP resolution on day 5 after IT initiation was assessed as a primary outcome. The data were statistically analyzed by STATISTICA 7.0 (M, σ, Newman- Keuls test; p < 0.05).

**Results:** Administration of IT as an adjunct to systemic antibiotics was associated with a higher incidence of NP resolution (84 % vs. 52 %, p = 0,0322, χ criterion). The decrease of microbial titer to 103 to 104 CFU/ml was detected in both groups by days 5 to 7, but it was reliable in 73,6 % of the patients of group 1 (p < 0.02). Eradication of microbes in BAL on day 7 was achieved in 72 % of IT-treated patients and in 48 % of antibiotic shift patients. Treatment with IT made it possible to wean patients earlier: by day 8.2 ± 1.5 vs. 11.1 ± 2.8 in the other group (p = 0.001). Hearing loss and tinnitus were detected only in three patients treated with IT. There were no cases of bronchospasm during inhalation of IT. The mortality rates did not differ between the groups (16 % in IT-group vs. 12 % in antibiotic shift group).

**Conclusions:** Administration of IT as an adjunct to systemic antibiotics is efficient in the treatment of NP – it leads to a higher rate of NP resolution and an earlier weaning from ventilator.

## P103 In vitro evaluation of amikacin inhale and commercial nebulizers in a mechanical ventilator

### N. Kadrichu^1^, T. Dang^1^, K. Corkery^1^, P. Challoner^2^

#### ^1^Novartis Pharmaceuticals, San Carlos, USA; ^2^Nektar Therapeutics, San Francisco, CA, USA

**Fig. 18 (Abstract P103).**
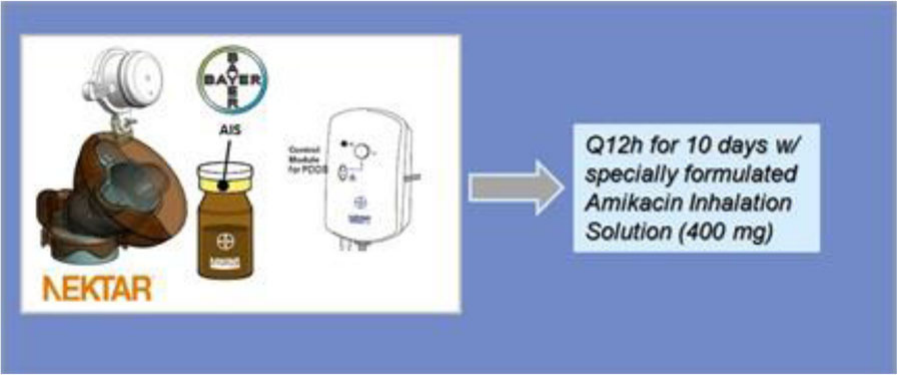
Amikacin Inhale, an integrated drug-device combination

**Introductions:** Amikacin Inhale is an integrated drug-device combination of: Pulmonary Drug Delivery System (PDDS), vibrating mesh nebulizer & specially formulated Amikacin Inhalation Solution (AIS) in development for Gram-negative pneumonia in intubated & mechanically ventilated patients (Fig. 18). This in vitro study compared the delivered dose of AIS from the PDDS vs commercially available nebulizers.^1^

**Methods:** Delivery of AIS (400 mg; 3.2 mL of 125 mg/mL) was compared for the PDDS vs commercially available jet, ultrasonic or conventional vibrating mesh nebulizers (CVMN). Each was tested at the ventilator location specified in its Instructions for Use (IFU). The CVMN was tested at 4 further positions. Except for the CVMN pre-humidifier, tests were performed without humidification.

**Fig. 19 (Abstract P103).**
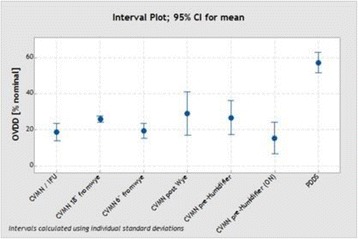
Performance of PDDS vs. CVMN

**Results:** Vibrating mesh nebulizers (PDDS, CVMN) achieved higher delivered doses than ultrasonic (4 %) and jet (11 %) nebulizers. The delivered dose of AIS post-ET tube was higher with the PDDS vs CVMN (57 % vs 19 % of nominal). Up to 60 % of AIS was collected in the expiratory filter with the CVMN vs 3 % for the PDDS. The CVMN had improved deposition (up to 29 %) at the extra locations relative to IFU position but this was still lower than with the PDDS (Fig. 19). Delivered dose with the CVMN was affected by humidity, which has previously been shown not to affect PDDS performance.^2^

**Conclusions:** Amikacin Inhale (AIS delivered by PDDS) provided a higher delivered dose of AIS vs commercially available nebulizers.

**References**

1. Kadrichu et al. J Aerosol Med Pulm Drug Del 2013;26:P054

2. Kadrichu et al. Intensive Care Med 2014:40(S1):P0867

## P104 The effects of nebulized amikacin/fosfomycin and systemic meropenem on severe amikacin-resistant meropenem-susceptible P.aeruginosa pneumonia

### G. Li Bassi, E. Aguilera, C. Chiurazzi, C. Travierso, A. Motos, L. Fernandez, R. Amaro, T. Senussi, F. Idone, J. Bobi, M. Rigol, A. Torres

#### Hospital Clinic, Barcelona, Spain

**Introductions:** Nebulization of antibiotics is a promising strategy for the treatment of severe pulmonary infections. We tested, in an animal model of severe pneumonia, short-term therapeutic efficacy of nebulized and systemic antibiotics.

**Methods:** Seventeen pigs (31.8 ± 1.9 Kg) were anesthetized and on MV for 78 hours. Multi-lobar pneumonia was developed by P. aeruginosa (1), resistant to amikacin (A), fosfomycin (F) and susceptible to meropenem (Mero). Following clinical diagnosis of pneumonia, animals were randomized to receive the following treatments: nebulized saline (control); nebulized AF; nebulized A; nebulized F; nebulized AF with Mero administered IV; Mero alone. Nebulization was performed through an in-line vibrating mesh nebulizer (PARI GmbH, Germany). Upon autopsy, lungs were weighed and the lobes biopsied for P.aeruginosa quantification.

**Fig. 20 (Abstract P104).**
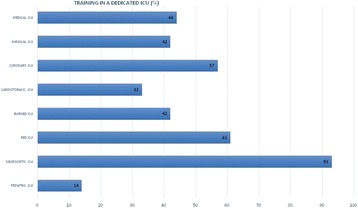
Median:plain line; Mean:dashed line. *p < 0.05 vs. Contr and Fosf; ^p < 0.05 vs. AF

**Results:** Upon autopsy, lungs weighed 732 ± 320gr in the control group, 546 ± 89gr in the AF, 560 ± 83gr in the A group, 519 ± 53gr in the F, 480 ± 85gr in AF + Mero and 537 ± 80gr in the Mero group (p = 0.591). As depicted in Fig. 20, P.aeruginosa concentration in lung tissue varied among groups (p < 0.001). In particular, in the AF + Merop group, the highest bactericidal efficacy was achieved.

**Conclusions:** In an animal model of amikacin-resistant P.aeruginosa pneumonia, the adjunctive use of nebulized AF and systemic meropenem achieves higher bactericidal efficacy vs. nebulized or systemic antibiotics alone.

**Reference**

1. Luna C et al. Chest 132, 2007: 523-31

## P105 Optimization of gentamicin peak concentrations in critically ill patients

### C. J. Hodiamont, N. P. Juffermans, J. M. Janssen, C. S. Bouman, R. A. Mathôt, M. D. De Jong, R. M. Van Hest

#### Academic Medical Center, Amsterdam, Netherlands

**Introductions:** Despite the administration of body-weight standardized starting doses of gentamicin, a wide range in peak concentration (Cpeak) is generally observed in critically ill patients resulting from variable pharmacokinetics of gentamicin between patients and within a patient over time. This may hamper the efficacy of gentamicin treatment. The aims of this study were 1) to determine the percentage of patients that reached a target Cpeak level of 15-20 mg/L after the first dose as well as after subsequent doses during which therapeutic drug monitoring (TDM) was applied and 2) to quantify the impact of several patient parameters on the volume of distribution (Vd) of gentamicin, which is correlated with Cpeak.

**Methods:** Blood samples were prospectively collected and analysed by nonlinear mixed-effects modelling (NONMEM) to estimate Vd and Cpeak for every administration during a course of gentamicin therapy. Clinical data and routinely collected serum gentamicin levels from critically ill patients were analysed retrospectively with NONMEM to quantify the impact of patient parameters on gentamicin Vd and Cpeak.

**Results:** With a median starting dose of 4.9 mg/kg, 39 % of Cpeak were subtherapeutic after the first dose (n = 59 patients). In total, 52 % of 131 Cpeak were not within the therapeutic range of 15-20 mg/L during the course of therapy. Of the 20 cases with subsequent Cpeak values and no dose adjustment, 6 (30 %) changed from non-therapeutic to therapeutic and 3 (15 %) changed from therapeutic to non-therapeutic Cpeak. Even after performing TDM, 24 % of subsequent doses resulted in subtherapeutic and 24 % in supratherapeutic levels. Analysis was performed retrospectively on 303 gentamicin concentration measurements from 44 critically ill patients receiving 174 doses. Albumin serum level was significantly (p < 0.001) associated with Vd and with Cpeak. Most patients with albumin levels below 15 mg/L did not obtain therapeutic Cpeak.

**Conclusions:** 39 % of gentamicin Cpeak were below the therapeutic range after a first median dose of 4.9 mg/kg in critically ill patients, suggesting that higher doses are indicated. Patients with hypoalbuminaemia are at increased risk for achieving suboptimal gentamicin Cpeak, suggesting that gentamicin dose should be increased for patients with hypoalbuminaemia. Since gross fluctuations in Cpeak were observed over time within patients, Cpeak should be measured repeatedly when patients receive gentamicin treatment for more than 2 days.

## P106 Systematic review of cefepime induced neurotoxicity

### L. Payne, G. L. Fraser

#### Maine Medical Center, Portland, USA

**Introductions:** Cefepime is a widely used antibiotic with the potential for significant, but under-appreciated neurotoxicity due to its ability to cross the blood brain barrier and antagonize GABA activity in a concentration dependent fashion. Data suggest that 15 % of ICU patients treated with cefepime will experience neurotoxicity with symptoms that range from encephalopathy, myoclonus, seizures and coma. Recognition of this adverse event is difficult since these symptoms are commonly experienced by the critically ill. We reviewed published literature to further characterize this adverse drug event.

**Methods:** A librarian-assisted search was conducted to create a list of all publications associated with cefepime associated neurotoxicity patients from 1980-2015. Our search employed the CINAHL and MEDLINE databases for article identification and the PRISMA-P protocol was followed to promote robust reporting of data. To determine eligibility for study inclusion, identified articles were independently assessed by two reviewers.

**Results:** Thirty-five identified publications described cefepime induced neurotoxicity in 137 patients. Patient characteristics included both ICU (17 %) and non-ICU patients (4 %), but patient location was unspecified in most cases (79 %). Symptoms included encephalopathy (75 %), myoclonus (41 %), seizures (11 %), and coma (1 %). Renal dysfunction was present in 93 (68 %) patients and cefepime dosing was appropriate, excessive, or unable to be assessed for 20 %, 41 %, 39 % respectively. Symptom onset was between 1-15 days (median 4) after initiation of therapy with resolution within 3 days (median 2) of cessation of cefepime. Emergent hemodialysis was employed in one case, and 55 patients (40 %) required treatment with an antiepileptic medication. Serum levels were collected in 12 patients and were exceeding toxic thresholds (<20 mcg/ml) in 11 patients (mean 64 ug/mL). Ninety-one patients (66 %) survived, 13 patients died (9 %), and the individual outcomes of the remaining patients were unreported. Complete neurologic recovery was reported in 62 patients (45 %) and symptom improvement in 27 (20 %). All patients with reported neurologic recovery did not improve until cefepime was discontinued.

**Conclusions:** Cefepime associated neurotoxicity is an under-appreciated adverse drug event that is difficult to recognize in the critically ill. Early recognition of cefepime neurotoxicity is critical and may prevent potentially devastating consequences that occur with continued drug administration.

## P107 Unasyn® causes QT prolongation during treatment of intensive care patients

### B. Tudor, M. Lahner, G. Roth, C. Krenn

#### Medical University of Vienna, Vienna, Austria

**Introductions:** Patients admitted to an intensive care unit have a multifactorial disorder based on diseases in which cascades of immunomodulation mediators may be released as response to pathogenic organisms. To treat the inflammatory reaction an antibiotic therapy is fundamental [1]. Effects of disease pattern and intensive care measures (e.g. sepsis) on heart rate and variability are poorly understood.

Haran B described that with a high resolution ECG even in patients without prior heart pathologies an electrical instability could be detected during repolarisation shortly before cardiac arrest, not recordable with a conventional ECG [2].

Therefore we analysed changes of beat-to-beat cardiac activity during antibiotic therapy of intensive care patients with a high resolution electrocardiogram. Obtained results may offer new insights in the development of alterations in cardiac electrical activity of critical ill patients due to antibiotic therapy.

**Methods:** Administrated at 1000 Hz sampling rate the cardiac electric activity of 14 patients of the intensive care unit were analysed during their antibiotic therapy. The patients received a Unasyn®-infusion, which contains 1 g Sulbactam, 2 g Ampicillin and 230 mg sodium. Obtaining continuous ten-minute recordings (Lab SystemTh Pro - Bard electrophysiology U.S.A.) ten electrodes were fixed on the prepared skin for recording the leads I, II, III and V1 to V6 and reconstruct pursuant to Einthoven’s equation aVR, aVL, aVF.

**Results:** Results obtained from 14 treatments with Unasyn® demonstrate that from the onset of the infusion the QT-interval increases additionally up to 39 ms (p < 0,05). This variation persisted for the first three minutes of therapy and returns during the next two minutes to their pre-values. Other ECG data remained unchanged during the time of treatment.

**Conclusions:** Haemodynamic alterations – QT-interval prolongation - could be detected with onset of antibiotic treatment with Unasyn®. The similar antibiotic Tazonam® showed in another study of us no significant beat-to-beat changes. With regard to comorbidities of ICU patients, it seems reasonable that changes in cardiac electric activity might be observed even earlier during their ICU stay.

**References**

1 Weimann K, J Int Med Res. 2015.

2 Haran B., Journal of Electrocardiology. 2006.

## P108 Comparative study between teicoplanin and vancomycin in methicillin-resistant staphylococcus aureus (mrsa) infectious of toxicological intensive care unit (ticu) patients – Tehran, Iran

### H. Talaie

#### Toxicological Research Center, Department of Clinical Toxicology, Loghman-Hakim Hospital, Shahid Beheshti University of Medical Sciences,Tehran, Iran

**Introductions:** Our objective is to compare efficacy and safety of vancomycin versus teicoplanin in Methicillin-resistant staphylococcus aureus (MRSA) infections among poisoned patients of toxicological ICU of Loghman Hakim hospital.

**Methods:** Safety and efficacy of vancomycin versus teicoplanin has been assessed in 104 patients consisted of 54 patients treated by teicoplanin and 50 patients treated by vancomycin. Based on the manufacturer’s instruction and kidney function in each patient, drug dose was adjusted. Teicoplanin was administered at a loading dose of 6 mg/kg (400 mg maximum dose) for three loading doses every 12 hrs and then every 24 hrs for 7 to 10 days. Vancomycin was administered at a loading dose of 20 mg/kg every 12 hrs (maximum dose 2gr/day). Blood, urine and tracheal samples were cultured. Chest X-ray and routine Para clinical studies have been done in all cases. The study populations were assessed during 3 visits and one month follow up. Patients with fever and positive tracheal cultures (TC) ± abnormal WBC at the end of treatment, have been reported as failure of treatment.

**Results: S**eventy eight (75 %) out of the 104 eligible patients, were male. The mean age ± SD of patients was 36.1 ± 16.8 and 39 ± 13.4 in teicoplanin and vancomycin groups, respectively. Most common drug toxicities were opium, TCA (tricyclic antidepressant), methadone. Mortality rate in teicoplanin group was 16.6 % but in vancomycin was 22 %.Complications during respiratory infection process were seen in 5/50 and 9/54 in vancomycin and teicoplanin groups respectively, including ARDS 2 (40 %) in vancomycin group and 7 (77.8 %) in teicoplanin group, pleural effusion in 2 (40 %) in vancomycin group versus 1 patient (11.1 %) in teicoplanin group. One patient in teicoplanin group had empyema and 1 (20 %) in vancomycin group had chronic obstructive pulmonary disease (COPD). Treatment failure in vancomycin group was 5/50 (10 %) and in teicoplanin group was 4/47(8.5 %) (P value > 0.05) and all positive TC in both groups were polymicrobial. Nephrotoxicity and bicytopenia, as the adverse effects had significant differences between two groups.

**Conclusions:** Teicoplanin should be considered as an effective alternative to vancomycin in Methicillin-resistant staphylococcus aureus (MRSA) infections treatment. Adverse effects such as nephrotoxicity and bicytopeniasignificantly were decreased in teicoplanin therapy.

## P109 Phage therapy against antimicrobial resistance, design of the first clinical study phagoburn

### P. Jault^1^, J. Gabard^2^, T. Leclerc^1^, S. Jennes^3^, Y. Que^4^, A. Rousseau^5^, F. Ravat^6^

#### ^1^HIA Percy, Clamart , France; ^2^Pherecydes Pharma, Romainville, France; ^3^Hôpital Reine Astrid, Brussels, Belgium; ^4^CHUV, Lausanne, Switzerland; ^5^CHU Liege, Liege, Belgium; ^6^CH Saint Jospeh Saint Luc, Lyon, France

**Introductions:** Bacteriophages are environmental viruses. Lytic phages have proprieties to penetrate and multiply inside their targeted bacteria until the release of new virions. Many clinical empirical data are available from Georgia and Poland, but no control randomized clinical trial is available. The phagoburn study assess efficacy and tolerance of two cocktails of bacteriophages against Escherichia coli and Pseudomonas aeruginosa.

**Methods:** Natural phages were isolated from hospital and Paris sewages. They were featured by phenotype and genotype to exclude transfer of antibiotic resistance genes and lysogenic proprieties by Pherecydes-Pharma. PP0121 is a cocktail of 12 phages against E coli, and PP1131 was performed against P. aeruginosa.

Cocktails are applied by a topical way in burn patients infected by E. coli or P. aeruginosa applied through an alginate gauze (Algosteril) onto infected wounds. The control arm is treated by Silver Sulfadiazine (standard treatment).

The main goal is the comparison of time to reduce bacterial load of 2 quadrants on plate by daily eSwabs.

220 patients are expected. All patients are hospitalized in a burn unit to ensure a safety environment.

**Results:** Phagoburn was funded by European Commission (FP7) in 2013 for 3,8 million Euros. After transfer from research and development to a Master Phage Bank, bioproduction of phages was performed in GMP-like conditions by Clean Cells (Nantes-France). As a systemic absorption of phages is supposed, an isolator was necessary to ensure a sterile and apyrogenic solution. The 3 european regulators (French agency ANSM, AFMPS for Belgium, and Swiss-Medic for Switzerland) allowed the first inclusion in July 2015. In December, 15 patients were already included in 11 investigators centers.

**Conclusions:** One century after phages discovery, Phagoburn is the first mutlicentric control randomized trial ever done in the world about phage therapy in humans. Results will be available in 2016. Phages could be an old but innovative way in the war against antimicrobial resistance. Antimicrobial resistance could be the leading cause of mortality in 2050.

**References**

Nature Reviews,Volume 14, August 2015

The Lancet infectious diseases,Volume 15, No. 12, p1384–1385, December 2015

Nature Reviews Microbiology, AOP, published online 9 November 2015; doi:10.1038/nrmicro3564

## P110 Antibiotic dosing errors in critically ill patients with severe sepsis or septic shock

### H. Al-Dorzi, A. Eissa, S. Al-Harbi, T. Aldabbagh, R. Khan, Y. Arabi

#### King Saud bin Abdulaziz University for Health Sciences , Riyadh, Saudi Arabia

**Introductions:** Effective antibiotic therapy, which can be defined as the timely administration of the antibiotic to which the causative microorganism is susceptible using the right dose, has been associated with improved outcomes in severe sepsis or septic shock. However, antibiotic dosing errors are not uncommon. This study evaluated the frequency and the risk factors of antibiotic dosing errors in patients with severe sepsis or septic shock.

**Methods:** This was a prospective observational study of all adult patients with severe sepsis or septic shock who were admitted to the ICU between 01/10/2013 and 30/04/2014. Using Micromedex as the reference, the doses of administered intravenous antibiotics on the first ICU day were compared to the recommended doses and were classified as under-dosed, over-dosed or appropriate. We excluded antibiotics which usually does not need dose adjustment. Multivariate logistic regression analysis was performed to assess predictors of antibiotic dosing error (under- or over-dosing). Age, gender, height, weight, chronic cardiovascular and renal diseases, estimated glomerular filtration rate and presence of shock were the independent variables.

**Results:** During the study period, 189 patients had severe sepsis (62 %) or septic shock (38 %). We evaluated 263 antibiotic prescriptions. Most (62 %) patients received appropriate antibiotic doses. However, 39 (21 %) patients were under-dosed, 30 (16 %) were over-dosed, and 3 (2 %) had combination of over-dosing and under-dosing. Septic patients with chronic cardiovascular disease (p = 0.035) and chronic renal failure (p = 0.021) were more likely to have antibiotic dosing errors. However, respiratory failure, liver failure, immunodeficiency state and diabetes mellitus were not clearly associated with dposing errors. Vancomycin was associated with the highest dosing error rate (43 %). The other antibiotics (pipercillin/tazobactam, meropenem, imipenem, ceftriaxone, ciprofloxacin, colistin and gentamicin) were dosed appropriately in >70 % of prescriptions. The multivariate analysis showed that the estimated glomerular filtration rate was the only variable associated with dosing error (odds ratio, 0.98 per 1 ml/min increment; 95 % confidence interval, 0.97-0.99).

**Conclusions:** Antibiotic dosing errors, which include under- and over-dosing, were common in patients with severe sepsis or septic shock. Vancomycin had the highest rate of inappropriate dosing. Lower glomerular filtration rate was a significant predictor of dosing error and should be routinely considered for antibiotic dosing.

## P111 Does empiric antifungal therapy improve survival in septic critically ill patients? (immunocompromised excluded)

### A. Trifi, S. Abdellatif , F. Daly, R. Nasri , S. Ben Lakhal

#### University hospital center of La Rabta. , Tunis, Tunisia

**Introductions:** The management of invasive candidiasis (IC) remains a major challenge. Delayed antifungal therapy is known as an independent mortality factor in Septic shock attributed to IC [1]. Thus, empiric antifungal therapy (EAFT) can be indicated in septic patients at risk of IC. However, the unreasonable administration of antifungal is implicated in emergence of resistant candida strains [2]. The purpose was to evaluate the impact of an EAFT on 28-day survival in septic patients without documented Candida infection.

**Methods:** a retrospective cohort study. Two groups of septic patients without a documented fungal infection were compared according to whether they were treated or not by an EAFT. Included were hospitalized patients more than seven days that developed a sepsis and even the sepsis origin has not been determined. Excluded were patients treated with an antifungal adapted to documented fungal infection and immunocompromised. The analysis consisted in evaluating the impact of an EAFT on 28-day survival. The analysis was adjusted on the following confounding factors: the Acute Physiology And Chronic Health Evaluation II (APACHE II) score, the candida score, invasive ventilation and central catheterisation.

**Results:** 83 patients were included. 48 in the EAFT + group and 35 in the EAFT- group. The patient’s baseline characteristics were comparable in severity illness, underlying diseases and risk factors for IC. Moreover, the EAFT + group had a younger age (48 vs 58, p = 0.03) and higher candida score (3.08 vs 2.31, p = 0.01). It has not been demonstrated an improvement of 28-day survival of an EAFT when administered to septic patients without documented fungal infection. These results were in accordance both in crude analysis and after adjusting on APACHE II score, candida score, invasive ventilation and central catheterisation with OR = 0.73 ; CI 95 % [0.28; 1.91]; p value = 0.53. However, improved survival by an EAFT was showed in patients with an APACHEII score < 16: OR = 0.62; CI95% [0.39; 0.97]; p =0.036. Similar results were objectified by the Kaplan-Meier survival curves.

**Conclusions:** no beneficial impact of an EAFT on at 28-day survival apart moderately ill patients with APACHE II <16.

**References**

[1] Kollef M et al. Clin Infect Dis. 12: 1739–1746, 2012.

[2] Fekkar A et al. Eur J Clin Microbiol Dis. 33 :1489-1496, 2014.

## P112 Neurocysticercosis-Qatar experience

### F. Paramba, N. Purayil, V. Naushad, O. Mohammad, V. Negi, P. Chandra

#### Hamad Medical Corporation, Doha, Qatar

**Introductions:** Neurocysticercosis (NCC) is the most common parasitic disease of the central nervous system. Every newly diagnosed patient with NCC has probably been infected by someone harboring tapeworm in patient’s immediate environment. On the basis of an incorrect assumption that human NCC does not occur in countries in which law prohibit swine breeding and consumption of pork, the disease is has been considered nonexistent in Arab world.

**Methods:** A retrospective study was carried out in the Emergency department, Alkhor hospital, HMC from April 2014 to May 2015 (14170/14). All patients above age of 18 yrs diagnosed to have Neurocysticercosis from August 2005 to December 2013 were included in the study. Data were retrieved from medical record department and electronic data base. This included baseline demography, clinical presentation and radiological findings.

**Results:** Out of total 137 subjects enrolled 9 were excluded. All the 128 subjects were male Majority of the subjects was in the age group 21-30 yrs (n = 86)-. Majority were from Nepal 76 (58.1 %). 30 (20.3 %) patients gave history of previous episodes of seizure, however only 8 of them were on regular antiepileptic medication. 107 patients presented to A &E with seizures (83.5 %). Among which GTCS was the most common form 85 %. Use of tobacco was seen in 20 subjects (13.6 %) and Alchohol in 10 (6.8 %). 9 subjects reported to use both. Radiological examination, CT scan revealed solitary lesion in 84 (65.5 %) subjects and majority were calcified 39.8 %. Perilesional edema was noted in 100 subjects.

114 patients were admitted to the hospital out of which 5 required ICU care. Anti-epileptic medication was initiated in all patients except 10 patients. Reason could not be identified. Except for 13 patients all other patients received short course of oral steroids.

**Conclusions:** 1) Majority of the patients are young males from Asian countries.

2) GTCS is the most common mode of presentation.

3) Solitary lesion is the most common radiological findings.

The increased ease of international travel and increasing number of immigration from developing countries have led to widespread recognition of NCC developed countries.

**Reference**

1) Growing frequency of Neurocysticercosis in Spain. Esquivel A. Neurologia 2005,20(3):116-120

## P113 Early indicators in acute haemorrhagic shock

### A. Kleinsasser

#### MUI, Innsbruck, Austria

**Introductions:** Acute haemorrhage in the CCU may be hard to perceive. Variables other than blood pressure or haemoglobin are sought after.

**Methods:** In this study we examined how acute haemorrhage affected cardiovascular, blood gas, laboratory and pulmonary gas exchange variables before, right after and 60 minutes after a 65 % blood loss in a model with anesthetized pigs. Blood pressure, lactate concentration, base deficit and blood glucose may all act as indicators for acute haemorrhage.

**Results:** All animals had comparable variables at baseline. Right after exsanguination animals expectedly showed depression of cardiovascular variables. In addition, lactate and blood glucose were increased directly after blood loss. The drop in haemoglobin concentration still remained insignificant at 60 minutes after blood loss. Base deficit was only different 60 minutes post bleed.

**Conclusions:** Glucose and lactate are earlier indicators than Hb or BE.

**Fig. 21 (Abstract P113).**
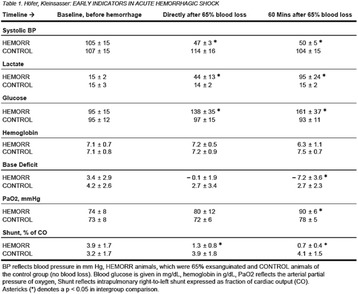
ᅟ

## P114 Filtering of red blood cells reduces the inflammatory response of pulmonary cells in an in vitro model of mechanical ventilation

### M. R. Witrz, J. F. Buchner-Doeven, A. M. Tuip-de Boer, J. C. Goslings, N. P. Juffermans

#### Academic Medical Centre, Amsterdam, Netherlands

**Introductions:** Red blood cell (RBC) transfusion is associated with acute lung injury, but the causative factor is not known. Possibly, mechanical ventilation and red blood cell (RBC) transfusion are synergistic in the induction of lung injury. We hypothesize that filtering of RBCs or depletion of RBCs from microvesicles decreases the pulmonary inflammatory reaction during mechanical ventilation.

The objective of this study is to investigate the effects of filtering of RBCs or depletion of RBCs from microvesicles on the production of IL-8 in an in vitro model of mechanical ventilation.

**Methods:** Alveolar epithelial type II A549 cells were cultured on an elastic membrane and incubated with cells or supernatant from stored (35-42 days) RBC products. RBCs were optimized by filtering of the supernatant using a 0.2micron filter, or by depletion of the supernatant from microvesicles using high-speed centrifugation (N = 4 per group). Cyclic stretch of 25 % was applied to the cells for 24 hours using a cell-stretcher at 37˚C in the presence of 5 % CO2. IL-8 production was measured using ELISA. Controls are untreated products and non-stretched cells.

**Results:** Stretched cells showed higher levels of IL-8 compared to non-stretched cells (198.2 ± 11.7 vs 62.0 ± 1.9 pg/ml, p < 0.05). Incubation with untreated RBC products further spiked IL-8 production compared to cells that were only stretched (892.8 ± 235.8 vs 198.2 ± 11.7 pg/ml, p = 0.027). Both filtering of supernatant and depletion of microvesicles resulted in reduction in levels of IL-8, compared to samples to which untreated RBCs were added (respectively 306.6 ± 73.1 vs 340.2 ± 24.4 vs 892.8 ± 235.8 pg/ml, p = 0.02).

**Conclusions:** Stretching of alveolar cells and RBC transfusion have a synergistic effect on the production of IL-8. Furthermore, our experiments indicate that supernatant of RBCs contain causative agents for IL-8 production. Possibly, microvesicles are responsible for this reaction. Filtering of RBCs might be a reasonable and feasible procedure to remove microvesicles from the products.

## P115 Microparticles from red blood cell transfusion induce a pro-coagulant and pro-inflammatory endothelial cell response

### M. Van Hezel^1^, M. Straat^1^, A. Boing^1^, R. Van Bruggen^2^, N. Juffermans^1^

#### ^1^Academic Medical Center Amsterdam, Amsterdam, Netherlands; ^2^Sanquin, Amsterdam, Netherlands

**Introductions:** Red blood cell (RBC) transfusion is associated with increased morbidity and mortality in the critically ill. We hypothesized that MPs from stored RBC bags can induce endothelial activation, either directly or mediated by immune cells.

**Methods:** MPs were isolated by high speed centrifugation from RBC transfusion bags. Human umbilical vascular endothelial cells (HUVECS) were incubated for 6 hours with supernatant from RBC bags either containing MPs or depleted from MPs, with or without addition of monocytes or granulocytes. Controls were incubated with PBS as a negative and TNF as a positive control. Expression of adhesion markers were measured on flow cytometry and markers of coagulation and inflammation were measured in the culture medium. Monocytes were cultured with stained MPs and adherence was studied using confocal microscopy.

**Results:** Supernatant from RBCs containing MPs up-regulate endothelial expression of ICAM and E-selectin with 7.8 [2.8 – 22.9]% and 2.6 [-1 – 7.7]% resp. compared to baseline. Up-regulation was absent when stimulated with RBC supernatant depleted from MPs. Also, up-regulation depended on the presence of monocytes. MPs adhere to monocytes, which was partly abrogated after co-incubation with antiCD11b or anti-CD18 antibodies (from 100 to 67[57-72]% and to 56[47-67]% of positive cells resp.). RBC-derived EVs also strongly induce endothelial shedding of vWF.

**Conclusions:** Up-regulation of endothelial cell adhesion markers and shedding of vWF antigen following RBC transfusion is caused by MP, and requires the presence of monocytes. Thereby, MPs from RBC units induce a pro-inflammatory and pro-coagulant endothelial cell response.

## P116 The contribution of cytokines on thrombosis development during hospitalization in ICU

### D. Markopoulou^1^, K. Venetsanou^1^, V. Kaldis^1^, D. Koutete^1^, D. Chroni^1^, I. Alamanos^2^

#### ^1^KAT Hospital Athens, Kifisia, Greece; ^2^ICU-B, KAT Hospital Kifisia, Athens, Greece

**Introductions:** The development of thrombosis in ICU patients is one of the most frequent causes for unfavourable outcome. The different types severe illness are accompanied by activation of cellular and biochemical processes. The aim of the study is to investigate the possible effect of cytokines on thrombosis in trauma and neurosurgical patients in ICU.

**Methods:** Eighty-four patients, 46 with trauma and 38neurosurgical included in the study, divided into two groups according to thrombosis incidence. Demographic and clinical data were recorded and a single blood sample collected on admission. Interleukins (IL-) -6,-8,-10 and Tumor necrosis factor (TNF)-a, were determined in serum by ELISA. Statistics performed with Graphpad 5.0.

**Results:** Patients with thrombosis showed statistically significant elevated proinflammatory cytokines (TNF-a, p < 0.01, IL-6, p < 0.05) and lower anti-inflammatory IL-10 release (p < 0.05) on admission, compared to those without thrombosis. IL-8 admission levels, had no difference between groups (p > 0.05), due to later expression. There were no significant differences between age and hospitalization days in both groups. Thrombosis found to be correlated with poor outcome (p < 0.05).

**Conclusions:** The early activation of cytokines in trauma and neurosurgical patients seems to play an important role on thrombosis development and further patient’s outcome.

**Reference**

Markopoulou D, Venetsanou K et al Examining venous thromboembolic disease in postoperative neurosurgical and trauma patients in ICU. Critical care 2015, 19 (Suppl 1):P 325

## P117 Prophylactic enoxaparin dosing and adjustment through anti-xa monitoring in an inpatient burn unit

### L. Koch, J. Jancik, H. Rhodes, E. Walter

#### Hennepin County Medical Center, Minneapolis, USA

**Introductions:** The incidence of venous thromboembolism (VTE) in acute burn patients has been reported to range from 0.4 %-23%. [1] Standard enoxaparin prophylaxis dosing has been shown to provide anti-Xa levels below goal in a significant proportion of burn patients, resulting in a potential increase in thrombotic risk. [2] The objectives of this study were to evaluate the effectiveness of standard prophylaxis enoxaparin dosing within an inpatient burn unit and identify patient characteristics associated with lower initial anti-Xa levels.

**Methods:** Patients admitted to the burn unit between November 2009 and July 2015 with an appropriately measured anti-Xa level were examined through retrospective chart review. Levels were considered appropriate if drawn 3-5 hours after at least three consecutive enoxaparin doses. Anti-Xa levels ranging from 0.1-0.4U/mL were considered to be within the goal prophylactic range. Patient demographics and injury data including age, sex, admission weight, and burn percentage of total body surface area (%TBSA) were documented. The incidence of VTE and adverse events, including bleeding events attributed to enoxaparin, were also recorded.

**Results:** Thirty-three acute burn injury patients treated with enoxaparin had at least one measured anti-Xa level during admission. Only 21 of these patients (63.6 %) had an initial anti-Xa level within the specified goal range. An additional 6 patients (18.2 %) were initially subtherapeutic, but achieved therapeutic levels with dose adjustment, while 4 patients (12.1 %) never had a measured anti-Xa within goal range prior to the discontinuation of enoxaparin or before discharge. The median enoxaparin dose required to achieve goal anti-Xa levels was 40 mg every 12 hours. Patients with an initial therapeutic level had an average burn size of 23.2 % TBSA and mean admission weight of 106.7 kg, while those with an initial subtherapeutic level had an average burn size of 40 % TBSA and mean admission weight of 114.1 kg. There was one VTE event among the study population, occurring in a patient with therapeutic anti-Xa levels. There were no documented bleeding events leading to the discontinuation of enoxaparin in any of the study participants.

**Conclusions:** Standard enoxaparin dosing was shown to provide initial subtherapeutic anti-Xa levels in many patients with acute burn injury. In the overall study population, the enoxaparin dosing strategy was associated with a low incidence of VTE and bleeding complications.

**References**

1. Faucher LD, et al. J Burn Care Res. 2007; 28: 661-3

2. Lin H, et al. J Trauma. 2011; 71:1557-61

## P118 Determination of optimal cut-off values of haemoglobin, platelet count and fibrinogen at 24 hours after injury associated with mortality in trauma patients

### K. Maekawa^1^, M. Hayakawa^1^, S. Kushimoto^2^, A. Shiraishi^3^, H. Kato^4^, J. Sasaki^5^, H. Ogura^6^, T. Matauoka^7^, T. Uejima^8^, N. Morimura^9^, H. Ishikura^10^, A. Hagiwara^11^, M. Takeda^12^

#### ^1^Hokkaido University Hospital, Sapporo, Japan; ^2^Tohoku University Graduate School of Medicine, Sendai, Japan; ^3^Tokyo Medical and Dental University Hospital of Medicine, Tokyo, Japan; ^4^National Hospital Organization Disaster Medical Center, Tokyo, Japan; ^5^Keio University School of Medicine, Tokyo, Japan; ^6^Osaka University Graduate School of Medicine, Osaka, Japan; ^7^Rinku General Medical Center, Osaka, Japan; ^8^Kinki University Faculty of Medicine, Osaka, Japan; ^9^Yokohama City University Graduate School of Medicine, Yokohama, Japan; ^10^Faculty of Medicine, Fukuoka University, Fukuoka, Japan; ^11^National Center For Global Health and Medicine, Tokyo, Japan; ^12^Tokyo Women’s Medical University, Tokyo, Japan

**Introductions:** The purpose of this study was to determine optimal cut-off values of haemoglobin, platelet count and fibrinogen at 24 hours after injury associated with mortality in trauma patients.

**Methods:** We performed a retrospective analysis of patients survived over 24 hours after injury from J-OCTET (Japanese Observational study for Coagulation and Thrombolysis in Early Trauma) database. J-OCTET was a retrospective multicenter study to investigate disorders of coagulation and thrombolysis in patients with severe trauma. Multivariable logistic regression models were developed to determine optimal cut-off values of hemoglobin, platelet count and fibrinogen at 24 hours after injury. We validated the models internally with bootstrapping to assess potential overfitting.

**Results:** There were 722 trauma patients included, with median age of 57 years, median injury severity score of 22, median revised trauma score of 7.84, and an overall mortality of 6.5 %. The optimal models associated with mortality were hemoglobin < 10.0 g/dL (c-statistic 0.77, 95%CI　0.69-0.85), platelet count < 10.0x10^4^ /μ L (0.80, 0.72-0.87), fibrinogen < 200 mg/dL (0.82, 0.72-0.92). After 200 cycles of bootstrapping, the average optimisms were 0.03, 0.03 and 0.01, respectively.

**Conclusions:** A hemoglobin < 10.0 g/dL, platelet count < 10.0x10^4^ /μ L and fibrinogen < 200 mg/dL at 24 hours after injury were associated with mortality. The impact of correction of these values with blood products warrants further validation.

**References**

1) Spahn DR, Bouillon B, Cerny V, et al: Management of bleeding and coagulopathy following major trauma: an updated European guideline. Crit Care 2013;17:R76.

2) Morel N, Delaunay F, Dubuisson V: Management of bleeding following major trauma: is a target haemoglobin of 7 to 9 g/dl high enough? Crit Care. 2013;17:442.

## P119 Trauma-induced coagulopathy - prothrombin complex concentrate vs fresh frozen plasma

### O. Tarabrin, S. Shcherbakow, D. Gavrychenko, G. Mazurenko, V. Ivanova, O. Chystikov

#### Odessa National Medical University, Odessa, Ukraine

**Introductions:** The mortality in the in patients with traumatic injuries in a case of bleeding is the most frequent cause of preventable death after severe injury.

**Methods:** The study involved 91 patients who entered the Odessa Regional Hospital with traumatic injuries: concomitant skeletal trauma, fractures of femur and humerus. Patients were divided into 2 groups: 1st group (n = 46) as a treatment was received PCC in a dose of 1 ml/kg (25 IU/kg) and TXA in a loading dose of 1 g during 10 minutes followed by an infusion of 1 g during 8 hours at time of admission to the intensive care unit (ICU); 2nd group (n = 45) received FFP in a dose of 15 ml/kg and TXA in a loading dose of 1 g during 10 minutes followed by an infusion of 1 g during 8 hours. Evaluation of the functional state of the hemostasis system was carried out using low-frequency piezoelectric thromboelastography (LPTEG) on admission to hospital and 24 hours after the patient’s admission to the ICU.

**Results:** According to LPTEG indicators traumatic injuries patients has a statistically significant abnormalities in all parts of hemostatic system: platelet aggregation - Intensity of contact coagulation (ICC), the coagulation - Intensity of coagulation drive (ICD), clot maximum density (MA) and fibrinolytic activity - Index of retraction and clot lysis (IRCL). ICC in patients with traumatic injuries was reduced by 29.59 %, ICD was less than normal at 37.59 %, MA was reduced by 74.71 %, which showed coagulopathy, IRCL was 90,78 % above the norm, which stands expressed hyperfibrinolysis. Patients of 1st group according to LPTEG had significant changes in all parts of coagulation 24 hours after the intensive care. Indicators of platelet hemostasis characterized by persistence of hypoagregation: ICC was reduced by 24.71 %, compared to the norm; parameters of coagulation and fibrinolysis have reliable trend toward normal and decreasing the activity of fibrinolysis index reaches normal reference values. Patients of 2nd group have hypoagregation and hypocoagulation state with decreased active of fibrinolysis: ICC was reduced by 24.72 %, ICD reduced by 20.76 %, MA was reduced by 23.54 %, IRCL was in the normal range. Clinically, patients of the 1st group had reducing the volume of blood transfusions as opposed to the 2nd group.

**Conclusions:** Patients with trauma-induced coagulopathy have violation in all parts of hemostatic system. The use of prothrombin complex concentrate and tranexamic acid can reduce the severity of pathological changes in the hemostasis in patients with traumatic injuries.

## P120 First study to prove the superiority of prothrombin complex concentrates on mortality rate over fresh frozen plasma in patients with acute bleeding

### C. Plourde, J. Lessard, J. Chauny, R. Daoust

#### Hopital Sacré-Coeur de Montréal, Montreal, Canada

**Introductions:** Prothrombin complex concentrates (PCC) proved its efficiency over fresh frozen plasma (FFP) in achieving faster coagulopathy reversal and many other factors. However, a recent Cochrane review/analysis found no superiority of PCC over FFP on mortality. Additional research is needed to determine the impact of PCC on mortality.

**Methods:** This retrospective cohort study enrolled adults presenting to the emergency department of a tertiary-care center between March 2008 and February 2015. All patients had evidence of acute bleeding from any source, were anticoagulated with vitamin K-antagonist and received vitamin K upon arrival. We compared mortality rates between FFP and PCC using the Kaplan Meier survival curve and a Cox multivariate regression.

**Results:** We identified 633 patients: 289 (45.7 %) received FFP and 344 (54.3 %) received PCC. Subjects treated with the FFP compare to PCC increase their probability of mortality during the follow up period (HR = 1.6 (95%CI: 1.2-2.1); p = 0.002) after controlling for confounding variables.

**Conclusions:** This study confirms the decrease in mortality over a 3 years follow up associated with PCC use compared to FFP in patients presenting for any type of acute bleeding. PCC should be used as a first line therapy for anticoagulation reversal of vitamin k antagonist.

**References**

Josepg B, Neurosurgery, 2015

Goldstein J, Lancet ,2015

Johansen M, The Cochrane Database, 2015

**Fig. 22 (Abstract P120).**
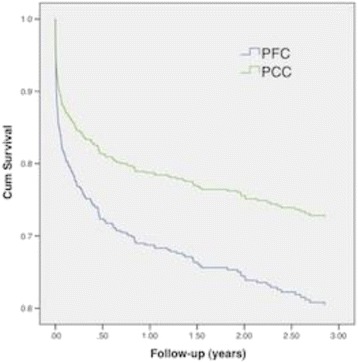
ᅟ

## P121 Prothrombin complex concentrate vs fresh frozen plasma in obstetric massive bleeding

### S. Shcherbakow, O. Tarabrin, D. Gavrychenko, G. Mazurenko, O. Chystikov

#### Odessa National Medical University, Odessa, Ukraine

**Introductions:** In the developing world about 1.2 % of deliveries are associated with postpartum haemorrhage (PPH) and when PPH occurred about 3 % of women died.

**Methods:** Our research involved 51 patients with massive postpartum bleeding after Ñesarean section that were divided into 2 groups: 1st group contained 10 patients as a treatment of massive bleeding with coagulopathy was scheduled PCC in a dose of 1 ml/kg (25 IU/kg), packed red blood cells (PRBC), 2nd group (41 patients) received fresh frozen plasma(FFP) in a dose of 20 ml/kg and PRBC. The functional state of the hemostasis system was carried out using low-frequency pyezoelectric thromboelastography(LPTEG) on admission to hospital and every 2 hours after admission.

**Results:** According to LPTEG indicators patients with massive postpartum bleeding had abnormality in all parts of hemostatic system: platelet aggregation - Intensity of contact coagulation(ICC) was reduced by 45.64 %, the coagulation - Intensity of coagulation drive(ICD) was less than normal at 59.32 %, clot maximum density (MA) was reduced by 88.15 % and fibrinolytic activity - Index of retraction and clot lysis(IRCL) was 86,16 % above the norm. Indicators of platelet hemostasis in patients of 1st group characterized by persistence of hypoagregation: ICC was reduced by 18.69 %, compared to the norm; parameters of coagulation and fibrinolysis have reliable trend toward normal and decreasing the activity of fibrinolysis index reaches normal reference values (ICD was less than normal at 10.65 %, MA was reduced by 19.31 %, IRCL was 15,21 % above the norm) 2 hours after, and became to the normal 4 hours after infusion of PCC. Patients of 2nd group had hypoagregation and mild hypocoagulation state with increased active of fibrinolysis: ICC was reduced by 22.79 %, ICD reduced by 20.79 %, MA was reduced by 30.54 %, IRCL was above the norm to 25.46 % 4 hours after, and became to the normal 6 hours after infusion of FFP. Clinically patients of the 1st group had reducing signs blood loss, decreased volume of transfusion PRBC for 11 % and decreasing volume of infusion therapy for 19 % compared to patients of 2 ng group. There was 1 case of transfusion related lung injury in 2nd group.

**Conclusions:** Obstetric patients with massive postpartum bleeding have violation in all parts of hemostatic system. The use of prothrombin complex concentrate can reduce the level of blood loss decrease volume of transfusion packed red blood cells and infusion therapy. Reducing the use of blood components in the intensive care unit of massive bleeding can be a method of preventing the development of TRALI-syndrome.

## P122 Impact of FFP transfusion on VAP in ICU patients

### A. Vakalos , V. Avramidis

#### Xanthi General Hospital, Xanthi , Greece

**Introductions:** The aim of our observation retrospective study was to test the hypothesis that a correlation exists between FFP transfusion and incidence of VAP in our both medical and surgical ICU served in community hospital.

**Methods:** From January 2006 to June 2014 admitted to our ICU 620 patients. From our database we looked for incidence of VAP (% ventilation days) and the following values and indexes according FFP transfusion per year from 2006 to 2014 (mean values). Total, per patient, per hospitalization days (HD), per patient under mechanical ventilation (pts V) and per ventilation days (VD) Using linear correlation method, we looked for linear slope, correlation coefficient (r), and coefficient of determination (r2), and by linear regression method using ANOVA test we looked for p value, according VAP and FFP transfusion.

**Results:**

**Conclusions:** According to our data, there was no statistically significant correlation detected between VAP and FFP transfusion indexes. Our data suggest that even though FFP transfusion may have an impact on immunosuppression and infection disease developing, the impact on VAP is not statistically significant.

**Table 15 (Abstract P122).** Correlation between VAP and FFP transfusion indexes

FFP	Slope	r	r2	St. Error	L. CI	U.CI	p value
Total transfusions	-0.236	-0.0151	0.0002	5.624	-13.527	13.075	0.9691
Transfusions per pt	-0.077	-0.3074	0.0944	0.0906	-0.2917	0.1368	0.421
Transfusions per H.D.	-0.001	-0.0802	0.0064	0.0073	-0.0088	0.0157	0.8574
Transfusions per Pt V.	-0.072	-0.2749	0.0755	0.0964	-0.301	0.1551	0.4741
Transfusions per V. Day	-0.005	-0.2188	0.0078	0.0095	-0.0283	0.017	0.5716

## P123 Preoperative platelet function test and the thrombin generation assay are predictive for blood loss after cardiac surgery

### L. Kropman, L. In het Panhuis, J. Konings, D. Huskens, E. Schurgers, M. Roest, B. De Laat, M. Lance

#### Maastricht UMC, Maastricht, Netherlands

**Introductions:** Peri- and postoperative bleeding is a major cause of morbidity and mortality in cardiac surgery. This study evaluates the value of thrombin generation (TG) and a new platelet function analysis in the prediction of haemostatic problems.

**Methods:** We studied 82 patients undergoing cardiac surgery in 2015.

Blood samples were collected before surgery (T1) (n = 82) and upon arrival at the ICU (T2) (n = 67).

TG was measured by Calibrated Automated Thrombography (CAT). Blood loss was recorded until 24 hours after surgery.

Platelet function was assessed by a platelet activation test (PAC-t-UB) which estimates P-selectin expression and binding of fibrinogen to activated alpha_IIb_beta_3_ after platelet activation of GPVI, PAR-1 and P2Y12 receptors, by specific agonists: P1Y12-ADP, PAR-1-TRAP, GPVI-CRP.

Patients were divided in a high and low blood loss group, with a cut-off value of 1 liter during ICU stay.

**Results:** TG parameters lagtime and time-to-peak in pre-operatively sampled whole blood, showed significant prolonging in the high blood loss group. (2.53 ± 0,52 vs 2.88 ± 0,87: p = 0.027 and 4,67 ± 0,61 vs 5,12 ± 1,00: p = 0.017 respectively).

The PAC-t-UB test showed significant differences between groups in P-selectin expression by GPVI, PAR-1 and P2Y12 agonists at T1 (Table 16) but not in fibrinogen binding.

**Conclusions:** Preoperative assessment of TG measured by CAT was predictive for blood loss following cardiac surgery.

These findings may help in identifying patients at risk for postoperative bleeding complications.

**Table 16 (Abstract P123).** Significant differences of agonists in P-selectin expression

Platelet test before operation	Mean (relative expression)	<999 ml (n = 63)	> = 1000ml (n = 19)	P-value t-test
ADP A P-selectin	5342 ± 1836	5582 ± 1830	4574 ± 1677	0,035
CRP A P-selectin	6184 ± 2049	6455 ± 2020	5283 ± 1931	0,028
TRAP P-selectin	7366 ± 1942	7623 ± 1813	6513 ± 2158	0,028

## P124 Rotational thromboelastometry versus standard coagulation tests before surgical interventions

### M. Durila, P. Lukas, M. Astraverkhava, J. Jonas

#### Second Faculty of Medicine, Charles University and University Hospital Motol, Prague, Czech Republic

**Introductions:** Prolonged value of prothrombin time/activated partial thromboplastin time (PT/APTT) are often found in patients undergoing surgical procedure. Thromboelastometry as a global test using whole blood might yield different results. The aim of our study was to compare coagulation profile as shown by conventional coagulation tests versus thromboelastometry before surgical procedures and to find out which test better reflects patient’s clinical condition in perioperative period.

**Methods:** In patients undergoing surgical procedures with prolonged PT/APTT blood sample was obtained for thromboelastometry-ROTEM-EXTEM. In case of normal result the patient underwent the procedure without administration of clotting factors and bleeding complication was observed in perioperative period.

**Results:** In 65 patients with PT-INR 1.46 ± 0.19 (min-1.22, max-2.22) and APTT 1.17 ± 0.18 (min-0.83, max-1.52) the procedure was performed without bleeding complication when ROTEM-EXTEM was in normal values for all parameters.

**Conclusions:** Our data suggest that surgical procedures can be done without bleeding complication despite prolonged PT/APTT in case of normal ROTEM-EXTEM. This approach can reduce FFP administration in daily practice.

**References**

1. Mohammed M, Fayed N, Hassanen A, Ahmed F, Mourad W, El Sheikh M, Abofetouh F, Yassen K, Khalil M, Marwan I, Tanaka K. Rotational thromboelastometry and standard coagulation tests for live liver donors. Clin Transplant 2013;27:E101-8.

2. Urwyler N, Theiler L, Hirschberg M, Kleine-Brueggeney M, Colucci G, Greif R. Standard vs. point-of-care measurement of fibrinogen: potential impact on clinical decisions. Minerva Anestesiol 2012;78:550-5.

3. Haas T, Spielmann N, Mauch J, Madjdpour C, Speer O, Schmugge M, Weiss M. Comparison of thromboelastometry (ROTEM(R)) with standard plasmatic coagulation testing in paediatric surgery. Br J Anaesth 2012;108:36-41.

4. Keene DD, Nordmann GR, Woolley T. Rotational thromboelastometry-guided trauma resuscitation. Curr Opin Crit Care 2013;19:605-12.

5. Gorlinger K, Saner FH. Prophylactic plasma and platelet transfusion in the critically Ill patient: just useless and expensive or even harmful? BMC Anesthesiol 2015;15:86.

## P125 Correction of impaired clot quality and stability by fibrinogen and activated prothrombin complex concentrate in a model of severe thrombocytopenia

### I Budnik^1^, B Shenkman^2^

#### ^1^Sechenov First Moscow Stat Medical University, Moscow, Russia; ^2^Sheba Medical Center, Tel-Hashomer, Israel

**Introductions:** Bleedings in severe thrombocytopenia (TCP) vary from mild to severe and may be even life-threatening when accompanied with hemodilution as a result of trauma and subsequent fluid resuscitation. The aim of this study was to evaluate the effect of fibrinogen and activated prothrombin complex concentrate (FEIBA) on clot quality and resistance to fibrinolysis in a model of TCP and hemodilution.

**Methods:** Blood was obtained from healthy volunteers who signed an informed consent form approved by the local research ethics committee. TCP [(16 ± 4) x 10^6^ mL^-1^] was created by mixing platelet-poor plasma with packed cells. The samples were diluted or not to 60 % with TRIS/saline buffer and subjected to clotting in the presence of tPA. Blood was spiked with fibrinogen (3.0 mg/mL) and/or FEIBA (1 U/mL). Maximum clot firmness (MCF, mm) and lysis onset time (LOT, min) were evaluated using rotational thromboelastometry and presented as Mean ± SD.

**Results:** MCF in nondiluted TCP blood was 18 ± 5. Spiking the blood with fibrinogen and FEIBA increased it to 29 ± 4 and 27 ± 7, respectively (P < 0.001). Combined use of these agents increased MCF to 34 ± 4 (P < 0.01). Dilution of TCP blood reduced MCF to 9 ± 5 (P < 0.001). Spiking diluted blood with fibrinogen increased MCF to 15 ± 4 (P < 0.01), spiking with FEIBA increased it to 22 ± 4 (P < 0.01), and combination of both agents had the greatest effect on MCF that reached 30 ± 6 (P < 0.01) and did not differ from its level in nondiluted blood following combined spiking. LOT in nondiluted TCP blood was 13.1 ± 5.1. Spiking the blood with fibrinogen had no effect on LOT while spiking with FEIBA prolonged it to 21.5 ± 7.3 (P < 0.05). Combined use of both agents further increased LOT to 33.0 ± 4.6 (P < 0.01). Dilution of blood as well as spiking diluted blood with fibrinogen had no significant effect on LOT while spiking with FEIBA prolonged it to the same extent as in non-diluted blood (P < 0.05). Compared to FEIBA, combination of both agents slightly prolonged LOT, but the difference did not reach statistical significance.

**Conclusions:** Both in nondiluted and diluted TCP blood: (1) fibrinogen and FEIBA synergistically improve clot quality (firmness); (2) fibrinogen alone does not increase clot resistance to fibrinolysis but potentiates such effect of FEIBA if added in combination. Both agents could serve as alternative therapeutic approach to bleeding patients with severe TCP.

## P126 Assessment of point-of-care prothrombin time analyzer as a monitor after cardiopulmonary bypass

### H. Hayami^1^, Y. Koide^2^, T. Goto^3^

#### ^1^Yokohama Municipal Citizen’s Hospital, Yokohama, Japan; ^2^Hayama Heart Center, Hayama, Japan; ^3^Yokohama City University Hospital, Yokohama, Japan

**Introductions:** Activated clotting time (ACT) is currently used for dose adjustment of heparin during CPB, and has been thought as gold standard in this field. However, in difficult case, such as prolonged cardiopulmonary bypass, consumption coagulopathy might also occur, and there might be need for blood components. Monitoring ACT only is not adequate in such case. The CoaguChek XS system is a portable instrument for monitoring oral anticoagulation therapy. It determines the PTINR value from a drop of capillary blood specimen, and it has been reported that accuracy seems to be better for hematocrit (Ht) >30 %. We compared PTINR measured by CoaguChek XS (CCINR) with PTINR analyzed by laboratory test (LabINR) after CPB.

**Methods:** 34 patients who underwent CPB were enrolled. After weaning from CPB and heparin reversal by protamine administration, CCINR, LabINR, ACT, aPTT and Ht were measured. The correlations between CCINR and LabINR, ACT and aPTT, were assessed.

**Results:** CCINR correlated well with labINR (r = 0.81, Y = -0.163 + 1.191 X). Moreover, evaluating subgroup whose Ht <30 % (N = 27, mean Ht 24.6 %), correlation was thought to be acceptable (r = 0.706, Y =0.047 + 1.058 X). On the other hand, correlation between ACT and aPTT, widely considered as “the Gold standard h was inferior (r = 0.567, Y =0.047 + 1.058 X).

**Conclusions:** CCINR is an useful alternative to labINR for guiding blood component therapy after CPB.

**Fig. 23 (Abstract P126).**
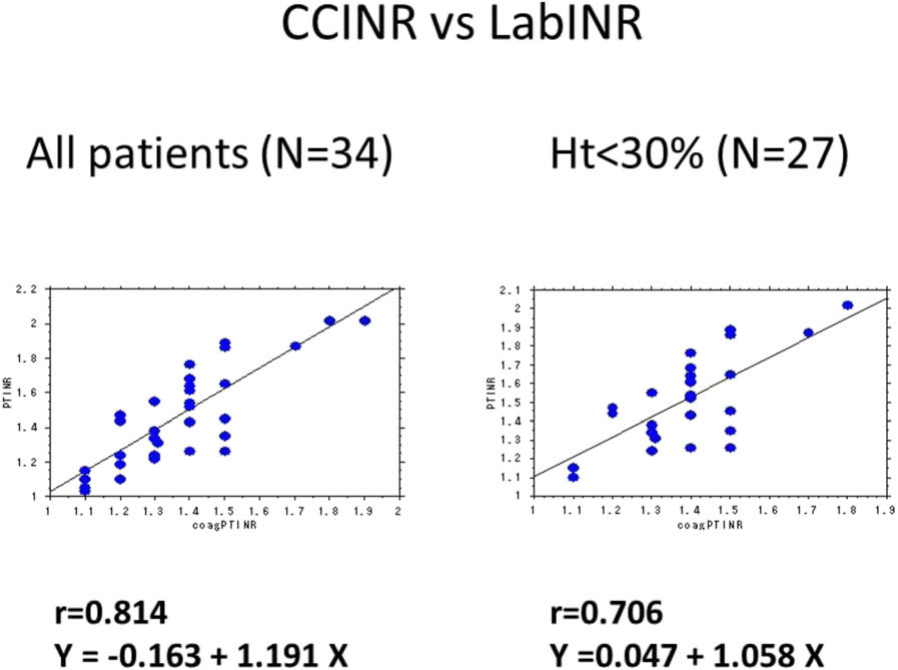
ᅟ

## P127 Disseminated intravascular coagulation (dic) is underdiagnosed in critically ill patients: do we need d-dimer measurements?

### V. B. Bombay, J. M. Chauny, R. D. Daoust, J. L. Lessard, M. M. Marquis, J. P. Paquet

#### ^1^Institute of Ageing and Chronic Disease, Liverpool, UK; ^2^Institute of Infection and Global Health, Liverpool, UK; ^3^Department of Haematology, Royal Liverpool University Hospital (RLUH), Liverpool, UK

**Introductions:** The International Society of Thrombosis and Haemostasis (ISTH) DIC scoring system has been developed as a simple tool to diagnose DIC. It is based on platelet count, prothrombin time (PT), fibrinogen level and fibrin degradation products (FDP) or D-Dimer results. However, the score is less useful in critical illness as D-Dimers and FDP are usually elevated due to the underlying disease. Hence neither DIC scoring nor FDP or D-Dimer measurements are routinely performed in ICU. Triggers to request D-Dimer measurements have not been established.

**Methods:** This retrospective study included ICU patients admitted to the RLUH over a 4-year period. Demographic details were collected at admission along with routine blood results for the first 7 days. A truncated DIC score was calculated for all patients who had coagulation tests done for 7 days: Platelet count >100x10^9^/L = 0 points, >50 to <100x10^9^/L = 1 point, < 50x10^9^/L = 2 points, PT <3 s prolonged = 0 points, >3 s but <6 s = 1 point, >6 s = 2 points, fibrinogen >1.5 g/L = 0 points, <1.5 g/L = 1 point. Patients with a truncated DIC score of ≥2 were considered high risk for DIC. In this patient cohort, highly elevated D-Dimers would establish a diagnosis of overt DIC. Patients admitted after elective surgery and those who stayed on ICU <7 days were excluded.

**Results:** 464 patients were included in the analysis. ICU mortality was 25.4 %. Mean APACHE II was 19.8 ± 7.1. 272 patients had a maximum DIC score of 0 (167 patients) or 1 (105 patients) and were not at risk for overt DIC. However, 192 patients had a maximum DIC score of ≥2, with 14 patients displaying a DIC score of 5 even without D-Dimer results available. In none of the patients with a DIC score of ≥2, D-Dimer or FDPs were analysed. Only one patient with a DIC score of ≥2 had normal platelet counts and two patients showed mild thrombocytopenia. All patients with severe thrombocytopenia and 73 of 122 patients with moderate thrombocytopenia had a DIC score of ≥2.

**Conclusions:** DIC, although associated with outcome, remains underdiagnosed in critically ill patients. In patients with moderate or severe thrombocytopenia a truncated DIC score should be calculated. If the score is between 2 and 4, measurement of D-Dimers or FDP should be requested to fully establish the diagnosis of DIC.

## P128 Validity of the age-adjusted d-dimer cutoff in patients with COPD

### B. Bombay, J. M. Chauny, R. D. Daoust, J. L. Lessard, M. M. Marquis, J. P. Paquet

#### Hôpital du Sacré-Coeur de Montréal, Montreal, Canada

**Introductions:** Diagnosing thromboembolic disease represents a challenge in older patients with COPD. The ADJUST-PE study has recently validated a new age-adjusted D-dimer cutoff (age x 10) in patients older than 50 years old. When the results of this study are cautiously analyzed, it seems that the validity of this cutoff in patients with COPD is unreliable. The objective of the current study was to specifically validate or invalidate the use of the age-adjusted D-dimer cutoff in patients with COPD aged 50 or older.

**Methods:** Files from patients who visited the emergency department of Hôpital Sacré-Coeur de Montréal between March 2008 and May 2014 were retrospectively reviewed. Patients aged 50 and older with COPD and with a non-high pretest probability of thromboembolic disease were included. These patients all had a D-dimer measurement and a radiologic exam (pulmonary angioscan, ventilation/perfusion lung scan or a lower extremity doppler). The results are presented in proportions with 95 % confidence intervals without continuity correction and with sensitivity measurements.

**Results:** 195 patients were included according to those criteria. 5 were excluded from analysis because of a non-confirmed diagnosis. Among the 190 patients analyzed, 15 had a diagnosis of thromboembolic disease, resulting in a prevalence of 7,89 % (IC95% = 4,49 %-12,69 %). 4 had a negative D-dimer level with the conventional cutoff (<500 μg/L) and 15 (all of them) had a negative d-dimer level with the age-adjusted cutoff. The sensitivity of the conventional cutoff was 73,33 % (IC95% = 44,90 %-92,21 %) and the sensitivity of the age-adjusted cutoff was 0 % (IC95% = 0,00 %-21,80 %).

**Conclusions:** In conclusion, the new age-adjusted D-dimer cutoff cannot be used safely in patient with COPD older than 50 years old who have a non-high pretest probability of thromboembolic disease.

**References**

Righini, JAMA 2014. 311 (11) : 1117-1124.

Schouten, BMJ 2013. 346:f2492.

## P129 A scoping review of strategies for prevention and management of bleeding following paediatric cardiopulmonary bypass surgery

### K. Siemens^1^, D. Sangaran^1^, B. J. Hunt^2^, A. Durward^1^, A. Nyman^1^, I. A. Murdoch^1^, S. M. Tibby^1^

#### ^1^Evelina London Children’s Hospital, London, UK; ^2^St Thomas Hospital, London, UK

**Introductions:** To systematically review literature reporting strategies for prevention and management of mediastinal bleeding post paediatric cardiopulmonary bypass (CPB) surgery.

**Methods:** Scoping review of publications (1980-2015) reporting the effect of any intervention on outcomes for postoperative bleeding (mediastinal drain loss, transfusion requirement, coagulation parameter). Inclusions: <18 years, cardiac surgery on CPB. Exclusions: haematological disorders. Databases included MEDLINE and EMBASE.

**Fig. 24 (Abstract P129).**
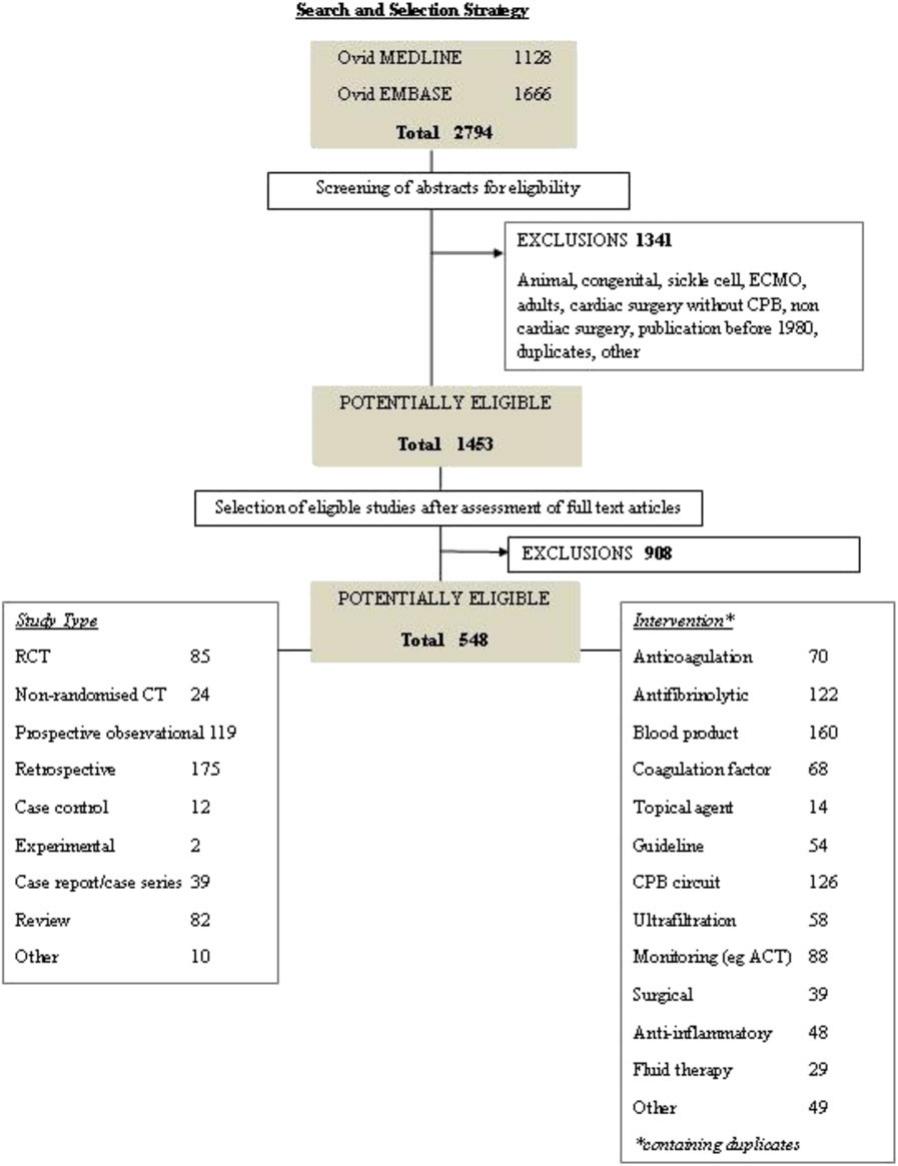
ᅟ

**Results:** 548/2794 screened articles were included: 466 (85.0 %) original research, predominantly retrospective 175 (37.6 %) and prospective 119 (25.5 %) observational studies, a smaller proportion of randomised controlled trials (RCTs) of 85 (18.2 %) and non-randomised controlled trials 24 (5.2 %) (Fig. 24). Study sizes ranged 5 - 22,258 (median 62). Most frequently evaluated interventions: blood products (17.3 %), CPB circuit modification (13.6 %), antifibrinolytics (13.2 %) (Fig. 24). Publication numbers increased at a mean rate of 6 per year from 14 (1980-85) to 229 (2010-15). Within this group the raise of increase of RCTs was 0 (1980-85) to 35 (2010-15).

**Conclusions:** A variety of interventions for bleeding prevention/treatment are available. Trial sizes are often small, quality of evidence low. There is a trend towards studies of higher quality (RCTs) in recent years.

## P130 Nadir hemoglobulin during cardiopulmonary bypass: impact on postoperative morbidity and mortality

### F. Ampatzidou , D. Moisidou , E. Dalampini, M. Nastou, E. Vasilarou, V. Kalaizi, H. Chatzikostenoglou, G. Drossos

#### G.Papanikolaou General Hospital, Thessaloniki, Greece

**Introductions:** Aim of this study was to evaluate the impact of low hemoglobulin (<7 g/dl) during cardiopulmonary bypass (CPB) because of hemodilution, on postoperative mortality and morbidity.

**Methods:** A total of 667 patients who underwent elective cardiac surgery procedures under the use of CPB in our department during a 2 years period were retrospectively studied. The lowest value of hemoglobulin (Nadir Hb) throughout CPB was recorded. Group A consisted of patients with Hb < 7 g/dl, while group B was the control group. The following perioperative outcome indices were compared between 2 groups: Increase of Lactate (Lac) value at the end of CPB, acute kidney injury (AKI) defined by RIFLE criteria, postoperative use of non invasive ventilation (NIV), prolonged mechanical ventilation (>48 hours), perioperative infarction (PMI), stroke, total red blood transfusion > 3units, atrial fibrillation(AF) and mortality. Statistical analysis was based on chi-square test.

**Results:** 162 patients consist group A and 505 control group.

Results are shown on Table 17.

**Conclusions:** Optimal hemoglobulin during CPB has not been defined. Severe hemodilutional anemia (Hb < 7 g/dl) has statistical significant correlation with increase of Lac value at the end of CPB, postoperative AKI, use of NIV, prolonged mechanical ventilation, transfusion with >3 red blood cell units and has strong correlation with mortality.

**Table 17 (Abstract P130).** ᅟ

	Group A No of pts (%)	Control Group No of pts(%)	p value
Lac increase	63 (38.9)	134 (26.5)	0.03
AKI	35 (21.6)	63 (12.5)	0.04
NIV	30 (18.5)	54 (10.7)	0.026
Prolonged ventilation	17 (10.7)	19 (3.8 )	0.01
PMI	3 (1.9)	4(0.8)	0.29
Stroke	3(1.9)	6(1.2)	0.46
RBC >3	86 (53.1)	105(20.8)	<0.01
AF	60(37)	156(30.9)	0.14
Deaths	9(5.6)	5(1)	<0.01

## P131 Red blood cell transfusion does not influence the prognostic value of RDW in critically ill patients

### S. Spadaro^1^, A. Fogagnolo^1^, T. Fiore^1^, A. Schiavi^1^, V. Fontana^1^, F. Taccone^2^, C. Volta^1^

#### ^1^Intensive Care Unit, University of Ferrara, Italy, Ferrara, Italy; ^2^Department of Intensive Care, Erasme Hospital, Université Libre de Bruxelles, Bruxelles, Belgium

**Introduction:** Some studies have found a correlation between increased RDW and mortality in ICU patients. However, RDW values can be increased by transfusion (RBCT), erythropoiesis or hemolysis. In this study we investigated whether the prognostic value of RDW was influenced by RBCT in ICU patients.

**Methods:** All patients admitted to ICU over 8 months were enrolled in the study. Patients were analyzed in two groups (A if no RBCT was given and B receiving RBCT before ICU admission). We measured RDW, reticulocyte index (RI) and haptoglobin on admission and 48 h thereafter. RDW was categorized in tertiles: <14 %; 14,1-15,8 %; >15,8 %. We collected 28-day mortality, changes in SOFA score and length of stay in ICU

**Fig. 25 (Abstract P131).**
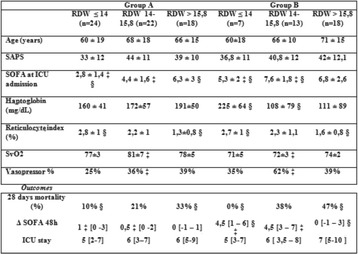
‡ = p < 0,05 compared to the other group § = p < 0,05 compared the same group

**Fig. 26 (Abstract P131).**
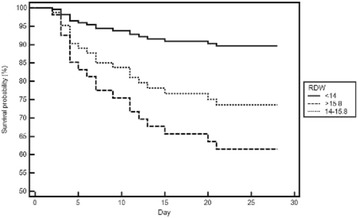
ᅟ

**Results:** 102 patients were enrolled(64 in group A and 38 in group B).Clinical variables are shown in Fig. 25. Cox-regression analysis shows that high RDW values were significantly associated with 28-day mortality (Fig. 26). Patients in Group B had higher level of RDW on admission (16.7 ± 2 % vs. 15.1 ± 2.2 %; p = 0.03). Nevertheless, the accuracy of RDW to predict mortality was similar in the two groups (ROC curve 0.697 for group A and 0.711 for group B). Patients with higher RDW had also a lower RI but similar levels of haptoglobin when compared to others.

**Conclusions:** Among critically ill patients RBCT were associated with higher RDW but this did not influence the prognostic value of RDW. High RDW values could not be secondary to an increased erythropoiesis, as suggested by low RI.

## P132 Reasons for admission in the paediatric intensive care unit and the need for blood and blood products transfusions

### E. Chochliourou, E. Volakli, A. Violaki, E. Samkinidou, G. Evlavis, V. Panagiotidou, M. Sdougka

#### Hippokration General Hospital Thessaloniki, Thessaloniki, Greece

**Introductions:** An important part in the puzzle of treatment of Pediatric Intensive Care Unit (PICU) patients (pts) is the transfusions of blood and blood products in order to stabilize the critically ill children, and improve their outcome. The purpose of the present study is to investigate the reasons for admission and the need for blood products transfusions in a cohort of PICU pts, and in specific diagnostic categories (sepsis, traumatic brain injuries, etc)

**Methods:** We studied retrospectively all consecutive admissions in the PICU during a two years period (from 01.01.2012 to 31.12.2013). Data were collected through chart reviews for the following parameters: Demographics, reason for admission, underlying diseases, treatment characteristics, blood and blood products transfusions, and outcome of the patients. The criterion for transfusions followed the Sepsis Surviving Campaign 2012 guidelines for the pediatric population and the 2013 European guidelines for the Management of Bleeding and Coagulopathy following major trauma. In special cases the criterion for transfusion was clinically significant hemorrhage. The definitions of transfusion units were equivalent to 10 ml/kg for Red Packed Cells (RPC) and Fresh Frosen Plasma (FFP), and 0.1 unit/kg for Platelets (PLT) and Cryoprecipitate (CP).

**Results:** A total of 233 PICU patients (124 boys and 109 girls) were recorded. There were 53 children with respiratory failure, 39 children with pathology of the nervous system, 31 with sepsis, mainly admitted from the pediatric oncology department, 24 with traumatic brain injuries, and others with miscellaneous reasons for admission. Almost 25 % of the children with respiratory and neurologic failure suffered from underlying diseases. A total of 1795 units (U) of transfusions were given which were grouped as follow: RPC (385 U), FFP (453 U), PLT (804 U), CP (153 U).

**Conclusions:** The most common reasons for admission in the pediatric intensive care unit were respiratory failure and pathology of the nervous system. These critically ill children presented in many occasions with anemia and thrombocytopenia, hemodynamic instability, clotting and bleeding disorders (while they were in infection) and the administration of blood and blood product derivatives were paramount to them. The immediate recognition of transfusion indications, and the early prompt interventions lead to better and faster recovery of small patients.

## P133 The implementation of a massive haemorrhage protocol (mhp) for the management of major trauma: a ten year, single-centre study

### R. Mothukuri, C. Battle, K. Guy, G. Mills, P. Evans

#### Morriston Hospital, Swansea, UK

**Introductions:** Massive haemorrhage is a leading cause of death in major trauma. About one-third of all bleeding trauma patients present with a coagulopathy as a result of physiologic derangements such as acidosis, hypothermia, or haemodilution related to fluid administration.(1) In 2011, Morriston Hospital introduced a Massive Haemorrhage Protocol (MHP) in the Emergency Department (ED). The aim of this study was to evaluate the impact of the MHP.

**Methods:** All adult trauma patients admitted to the ED (2005 to 2015) were studied, using the data collated for the Trauma Audit and Research Network. Variables included sex, age, Injury Severity score (ISS), PS14 code (injury severity score), injury mechanism, use of Tranexamic acid (TXA), volume of fluids/blood components given at the scene or in the ED, in-hospital mortality hospital and ICU and hospital length of stay. Patients were divided into two groups; patients admitted pre-MHP and patients admitted post-MHP introduction. Differences were analysed using the Fisher Exact test and Mann-Whitney U tests.

**Results:** A total of 731 patients were included, 376 in the pre-MHP and 355 in the post-MHP group. There were no significant differences in age, sex, ISS, PS14 code, injury mechanism and mortality between the two groups. The use of TXA was significantly higher in the post-MHP group (p < 0.001). The median amount of fluids given at the scene and in the ED was significantly higher (1.5 litres versus 1.0 litre) in the pre-MHP group (p < 0.001). A significantly higher number of units of prothrombin complex concentrate (PCC) and fresh frozen plasma (FFP) were given in the post-MHP group in the ED (both p < 0.05). There was no significant difference in the number of units of blood / plasma reduced cells given in the ED between the two groups. Hospital and ICU length of stay were significantly reduced following the introduction of the MHP (both p < 0.001).

**Conclusions:** The results of this study have demonstrated that a well-defined MHP may contribute to reducing both ICU and hospital length of stay. In the post-MHP group, patients received significantly lower amounts of fluids at the scene and in the ED and there was a significant increase in the use of TXA. A significant increase in the amount of PCC and FFP given to patients in the ED was reported, with no associated increase in blood/plasma reduced cells.

**Reference**

1) Spahn D, Bouillon B, Cerny V et al. Management of bleeding and coagulopathy following major trauma. Crit Care 2013;17:R76

## P134 An integrated major haemorrhage protocol for pre-hospital and retrieval medical teams

### J. Wijesuriya^1^, S. Keogh^2^

#### ^1^Central London School of Anaesthesia and Intensive Care Medicine, London, UK; ^2^University of the Sunshine Coast, Maroochydore , Australia

**Introductions:** Irrespective of aetiology, major haemorrhage is associated with poor patient outcome and its management presents considerable clinical and logistic challenges. The introduction of in-hospital major haemorrhage protocols, following damage control resuscitation (DCR) principles, has demonstrated improved patient outcome and blood product delivery with reduced wastage of blood products.^1,2^ However, simple adaptation of hospital-derived protocols is inadequate for use by retrieval teams and a tailored approach is required to address the specific challenges encountered.

**Methods:** A protocol for major haemorrhage management by retrieval services was developed, based on the best available evidence. Within the protocol integrated guidance was provided for retrieval teams and clinical coordinators.

**Results:** The clinical challenge of inter-mission variability of blood product availability was addressed by the use of two regimes within one retrieval team algorithm. The “Primary” regime is based upon the resources carried by the retrieval team and represents a minimum standard of major haemorrhage management for all patients. The “Inter-Facility Transfer” (IFT) regime provides guidance on delivering hospital-standard DCR, where possible, using locally available resources.

The logistic challenges encountered by retrieval teams are extensive. The retrieval clinical coordinator is instrumental in reducing delays, maximising use of resources and maintaining handover of information. Their role was clearly defined and a checklist developed in order to avoid omissions.

The protocol was adopted by Retrieval Services Queensland for state-wide use by fixed and rotary wing retrieval teams.

**Conclusions:** This novel protocol addresses many of the specific clinical and logistic challenges faced by retrieval teams. The protocol contains specific guidance yet provides flexibility to encompass the variety of major haemorrhage scenarios encountered, which may range from roadside polytrauma to the inter-facility transfer of an intensive care unit patient. The goal is seamless transition of care with ongoing damage control resuscitation from point of referral, during transfer, and upon arrival at the receiving centre.

**References**

1) Khan S. A major haemorrhage protocol improves the delivery of blood component therapy and reduces waste in trauma massive transfusion. Injury. 2013, 44(5):587-92

2) Pham HP and Shaz BH. Update on massive transfusion. Br J Anaesth. 2013 111(Suppl 1):i71-i82

## P135 The impact of transfusion thresholds on mortality and cardiovascular events in patients with cardiovascular disease (non-cardiac surgery): a systematic review and meta-analysis

### A. Docherty^1^, R. O’Donnell^1^, S. Brunskill^2^, M. Trivella^2^, C. Doree^2^, L. Holst^3^, M. Parker^4^, M. Gregersen^5^, J. Almeida^6^, T. Walsh^1^, S. Stanworth^2^

#### ^1^University of Edinburgh, Edinburgh, UK; ^2^John Radcliffe Hospital, Oxford, UK, ^3^Copenhagen University Hospital, Copenhagen, Denmark, ^4^Peterborough NHS Trust, Peterborough, UK, ^5^Aarhus University, Aarhus, Denmark, ^6^Hospital de Sao Paulo, Sao Paulo, Brazil

**Introductions:** Restrictive red cell transfusion policies are recommended as safe for the majority of hospital patients with anaemia. Uncertainty exists for patients with cardiovascular disease, in whom the heart may be more susceptible to limited coronary oxygen supply.

**Methods:** We performed a systematic review with meta-analyses of in-hospital randomised controlled trials that evaluated a restrictive vs liberal transfusion threshold and that included patients with cardiovascular disease. A comprehensive search of databases (including CENTRAL, MEDLINE and Embase) was performed on 02/11/2015. Data extraction was completed in duplicate. Risk of bias was assessed using Cochrane methodology. Mantel-Haenzel random effects models were used with relative risk ratios (95 % CI). Outcome measures were thirty-day mortality, and cardiovascular events.

**Fig. 27 (Abstract P135).**
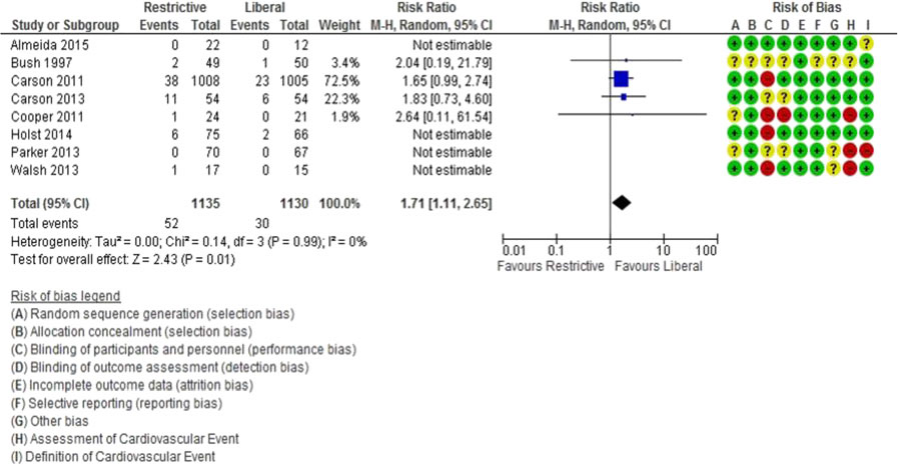
ᅟ

**Results:** We identified 41 trials of which 11 trials enrolling cardiovascular patients (n = 3033) were included for meta-analysis (restrictive n = 1514 patients; liberal n = 1519). There was no association between transfusion thresholds and 30 day mortality RR 1.10 (95 % CI 0.88-1.50, P = 0.30, I2 = 14 %). There was an increased risk of acute coronary syndrome (ACS) in patients in the restrictive transfusion threshold (9 trials; RR 1.71, 95 % CI 1.11-2.65, P = 0.01, I2 = 0 %) (Fig. 27).

**Conclusions:** Restrictive transfusion practice is associated with higher rates of ACS in patients with cardiovascular disease. This supports the uncertainty highlighted in current guidelines, and indicates the need for further research into best practice for this group.

## P136 The relationship between poor pre-operative immune status and outcome from cardiac surgery is specific to the peri-operative antigenic threat

### S. Moravcova^1^, J. Mansell^2^, A. Rogers^2^, R. A. Smith^2^, C. Hamilton-Davies^2^

#### ^1^Royal Brompton & Harefield NHS Trust, London, UK; ^2^Barts Heart Centre, London, UK

**Introductions:** We have previously demonstrated that there is an inverse relationship between pre-operative levels of endotoxin core antibodies (EndoCAb) and the development of complications following cardiac surgery[1,2]. It is not known whether this relationship is due to a specific protective effect against specific antigens or if it is a reflection of a generalised hypoimmunity. During the peri-operative period patients are at risk of exposure to endotoxin and staphylococcus with an incidence of surgical site infection of approximately 1-3 %.

**Methods:** 62 patients scheduled to undergo first time aortic valve replacement had serum analysed for levels of EndoCAb, antibodies to staphylococcus (teichoic acid, á-toxin) and also to varicella as a non-specific immune measure. The primary outcome variable was post-operative length of stay with secondary measures of incidence of post-operative infection. All patients were risk scored by means of Euroscore.

**Results:**

**Conclusions:** An inverse relationship is observed as previously between pre-operative EndoCAb levels and outcome. A similar relationship can be seen also between the anti-staphylococcal antibody levels and outcome with a more pronounced infective component. The absence of a relationship between the varicella antibody levels may imply from this study that low pre-operative immunity relating to post-operative morbidity is specific to the peri-operative threat.

**References**

1. Hamilton-Davies et al. CHEST;112:1189-96.

2. Bennett-Guererro et al. JAMA;277:646-50.

**Fig. 28 (Abstract P136).**
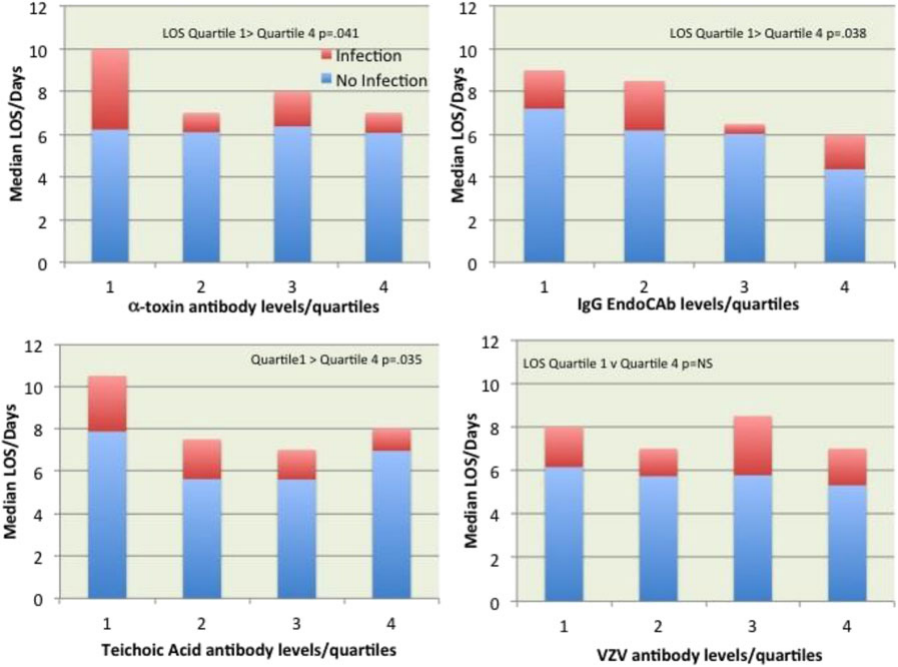
ᅟ

## P137 Impact of simple clinical practice guidelines for reducing post-operative atrial fibrillation after cardiac surgery

### A. Omar , M. Allam, O. Bilala, A. Kindawi, H. Ewila

#### Hamad medical corporation, Doha, Qatar

**Introductions:** In view the adverse consequence of post-operative atrial fibrillation (POAF) encompassing hemodynamic deterioration, thromboembolic hazards beside the intolerable fast rate, and mortality; preventing POAF seems to be an ideal goal. The POAF events incidence reported to be 30 and 40 % in coronary artery bypass and valvular surgeries respectively. [1, 2]. We aim to assess the impact of a simple clinical practice guideline for POAF events prevention and highlighting the incidence in Asian population.

**Methods:** Single center retrospective study conducted over one year, we enrolled all patients subjected to cardiac surgery (267), and patients with existing or prior AF were excluded. Patients were divided into two groups (group I) before and (group II) after implementing the CPG.

**Results:** Both groups were matched regarding the age, gender, smoking history, preoperative ejection fraction, EuroSCORE, urgency of surgery and underlying surgery type valvular or coronary. There was a statically significant difference in POAF events was14.9 % in group I and vs 8.3 % in group II (P = 0.05). The overall total rate of POAF in all patients was 11.4 % that is below the rate in European centers. [1] Patients with POAF significantly required more inotropic support (p = 0.04), in addition they were associated with prolonged length of mechanical ventilation and ICU stay (p = 0.001 and 0.02 respectively).

**Conclusions:** The argument of rhythm control in POAF would favor preventive measures that reduce post-operative transition from sinus rhythm with expected favorable effects of lessening the blood stasis and left atrial thrombosis chances.

**References**

1) Shrivastava R, Smith B, Caskey D & Reddy P. Atrial fibrillation after cardiac surgery: does rophylactic therapy decrease adverse outcomes associated with atrial fibrillation. J Intensive Care Med. 2009; 24(1), 18-25

2) Tapio H, Jari H, Kimmo M & Juha H. Prevention of atrial fibrillation after cardiac surgery. Scandin Cardiovasc J. 2007; 41(2), 72-78.

## P138 Dexamethasone administration during cardiopulmonary bypass has no beneficial effects on elective postoperative cardiac surgery patients

### F. Ampatzidou , D. Moisidou , M. Nastou, E. Dalampini, A. Malamas, E. Vasilarou, G. Drossos

#### G.Papanikolaou General Hospital, Thessaloniki, Greece

**Introductions:** Aim of this study was to investigate any beneficial effect on postoperative mortality and morbidity when a single dose of dexamethasone is administered during cardiopulmonary bypass (CPB) in cardiac surgery patients.

**Methods:** A total of 782 patients who underwent elective cardiac surgery procedures under the use of CPB in our department during 28 months period were retrospectively studied. In 499 patients a single dose of 8 mg dexamethasone was given during CPB( group A) while it was not administered in 283 patients (group B). The following factors were compared between 2 groups: Low cardiac output syndrome (LCOS), prolonged mechanical ventilation (>48 hours), septicaemia, postoperative stroke, acute kidney injury (AKI) defined by RIFLE criteria, AKI requiring dialysis treatment, atrial fibrillation(AF), sternal wound infections and mortality. Statistical analysis was based on chi-square test

**Results:** Group A consist of 499 pts, mean age 65.38 ± 9.8, mean EURO score II,1.86 ± 1.9. Group b consist of 283 pts, mean age 65.33 ± 11.7, mean EURO score II 1.7 ± 1.4.

Results are shown on Table 18.

**Conclusions:** Dexamethasone administration during CPB, has no protective impact on postoperative LCOS, septicemia, stroke, AKI, atrial fibrillation, sternal infections and mortality. There is a tendency for correlation with prolonged mechanical ventilation protection and the severe form of AKI requiring dialysis but not statistical significant.

**Table 18 (Abstract P138).** ᅟ

	Group A No of pts (%)	Control Group No of pts(%)	p value
LCOS	21(4.2)	17(6)	0.263
Prolonged ventilation	17 (3.4)	18(6.4)	0.055
Septicemia	9(1.8)	6(2.1)	0.756
Stroke	5(1)	5(1.8)	0.36
AKI	68(13.6)	46(16.3)	0.317
Dialysis	12(2.4)	14(4.89)	0.057
AF	159 (31.7)	89(31.4)	0.951
Sternal infections	9(1.8)	6(2.1)	0.756
Deaths	9(1.8)	6(2.1)	0.756

## P139 Intra-aortic balloon counterpulsation in patients undergoing cardiac surgery (IABCS): preliminary results

### G. Ferreira, J. Caldas, J. Fukushima , E. A. Osawa , E. Arita , L. Camara , S. Zeferino, J. Jardim, F. Gaioto, L. Dallan , F. B. Jatene , R. Kalil Filho , F. Galas, L. A. Hajjar

#### University of Sao Paulo, Brazi, Sao Paulo, Brazil

**Introductions:** The intra-aortic balloon pump (IABP) is used in a variety of clinical settings in which myocardial function is reduced. In cardiac surgery, its role on clinical outcomes is debated due to conflicting results of retrospective analysis and limitations of a recent prospective study. The IABCS study aims to evaluate the effectiveness of prophylactic IABP in high-risk patients undergoing cardiac surgery.

**Methods:** The IABCS study is a prospective, single-center, randomized controlled trial in high-risk patients scheduled to elective cardiac surgery at the Heart Institute/University of São Paulo. Inclusion criteria were additive EuroSCORE > = 6 or left ventricular ejection fraction (LVEF) < = 40 %. Eligible patients were randomly assigned, in a 1:1 ratio, to IABP group or control group. Removal of IABP catheter was accomplished after 24 hours of the procedure under the following circumstances: cardiac index > = 2.2 L/min/m2 and dobutamine infusion dose < = 5 μg/kg/min. The catheter was immediately removed if a severe adverse event related to the procedure was detected. The primary outcome was the composite endpoint of mortality and major morbidity in 30 days after cardiac surgery, according to the modified Society of Thoracic Surgeons definition, which included: prolonged mechanical ventilation (>24 hours), stroke, mediastinitis, need for reoperation, cardiogenic shock, and acute renal failure.

**Results:** A total of 116 patients were enrolled from April 2014 to September 2015. Fifty-two patients were assigned to IABP group and 64 patients to control group. The mean age was 64 ± 8 years in the IABP group and 67 ± 9 years in the control group (P = 0.06). The median LVEF was 40 % (31-45) in the IABP group and 40 % (35-55) in the control group (P = 0.873) and the median EuroSCORE was 6 (4-7) vs. 6 (4-7), P = 0.873, respectively. The primary outcome was observed in 40.4 % in the IABP group and 37.5 % in the control group (P = 0.751). Mortality rate was similar in both groups (15.4 % in the IABP group vs. 10.9 % in the control group, P = 0.478). Patients from the IABP group had a greater duration of inotrope use (3215 minutes [2003-5670] vs. 2110 minutes [1521-3355], P = 0.006) and longer ICU length of stay (5 [4-9] vs. 4 [3-6], P = 0.008).

**Conclusions:** Preliminary data demonstrated a similar occurrence of the primary outcome in both groups, with greater use of inotropes and longer ICU stay in the IABP group. Our results suggest that a tailored approach for IABP placement should be incorporated in future cardiac surgery studies.

## P140 Effects of low-dose atrial natriuretic peptide infusion on cardiac surgery-associated acute kidney injury

### C. Mitaka^1^, T. Ohnuma^2^, T. Murayama^2^, F. Kunimoto^3^, M. Nagashima^4^, T. Takei^4^, M. Tomita^5^

#### ^1^Juntendo University Hospital, Tokyo, Japan; ^2^Saitama Medical Center, Jichi Medical University, Saitama, Japan; ^3^Gunma University Hospital, Maebashi, Japan; ^4^Yokohama City Minato Red Cross Hospital, Yokohama, Japan; ^5^Tokyo Medical and Dental University Hospital of Medicine, Tokyo, Japan

**Introductions:** Acute kidney injury (AKI) after cardiovascular surgery is associated with an increase in morbidity and mortality. Atrial natriuretic peptide (ANP) is a potent endogenous natriuretic, diuretic, and vasorelaxant peptide. However, its effectiveness on AKI is uncertain. The objective of this study was to evaluate the effects of ANP on renal function and medical costs in patients with AKI undergoing cardiovascular surgery.

**Methods:** We conducted a multicenter prospective randomized placebo-controlled study in patients with AKI who underwent cardiovascular surgery including coronary-artery bypass grafting, valve replacement, and thoracic aorta replacement. This trial was conducted at eleven centers in Japan from May 2012 to March 2015. Definition of AKI used in this study was an increase in serum creatinine level > =0.3 mg/dl from preoperative level within 48 h after surgery. The patients were randomly assigned to receive a continuous infusion of low-dose ANP (0.02microg/kg/min) or placebo. The infusion of ANP or placebo continued until the serum creatinine level decreased to preoperative level. The primary endpoints were 1) changes in renal function during 90 days measured by serum levels of creatinine and cystatin C, and creatinine clearance or estimated glomerular filtration rate, and 2) need for renal replacement therapy during 90 days. The secondary endpoints were 1) length of ICU, 2) length of hospital stay, and 3) medical costs during 90 days.

**Results:** Of the 77 enrolled patients, 37 were assigned to the ANP group (median age 72 yrs, male:female 24:13) and 40 to the placebo group (median age 74 yrs, male:female 31:9). ANP infusion did not significantly increase creatinine clearance or estimated glomerular infiltration rate compared to placebo. There was no significant difference in serum levels of creatinine and cystatin C, renal replacement therapy rate (1 of 37 patients in the ANP group vs 3 of 40 patients in the placebo group), length of ICU and hospital stay, or medical costs for 90 days.

**Conclusions:** Low-dose ANP infusion did not show renoprotective effect nor cost-saving effect in the treatment of cardiac surgery-associated AKI.

Clinical trial registration number (UMIN 000006812).

## P141 Acute kidney injury influence on high sensitive troponin measurements after cardiac surgery

### A. Omar , K. Mahmoud, S. Hanoura , S. Sudarsanan , P. Sivadasan, H. Othamn, Y. Shouman, R. Singh, A. Al Khulaifi

#### Hamad medical corporation, Doha, Qatar

**Introductions:** The risk assessment of cardiac troponin and other cardiac biomarkers in end-stage renal disease is not equivalent where clinical decision making in patients with renal diseases based on cardiac biomarkers needs justification in relation to patient management or outcomes [1]. Long-term outcome could be influenced by acute kidney injury (AKI) in cardiac surgery [2], but cardiac troponins need exploration in theses settings. We aim at assessing the diagnostic performance of high sensitive troponin T (hsTnT) in the settings of cardiac surgery-induced AKI.

**Methods:** Single center observational retrospective study. A database was available for all patients. Based on the AKI Network definition patients were divided into 2 groups, group I without AKI (259 patients) and group II with AKI (100 patients) where serial of hsTnT and creatine kinase (CK)-MB followed. Both groups compared and statistically analyzed. We enrolled 359 patients, patients with ESRD were excluded.

**Results:** A total of 359 patients were enrolled with mean age of 55.1 ± 10.2 years. Both groups were matched regarding the age, gender, body mass index, the association of diabetes or hypertension, and Euro score. AKI group had significantly higher mortality 6 % vs group I 1.7 % (p = 0.026). The hsTnT significant changes between both groups remain all over the course, which unparalleled to those of CK-MB. The AKI group with more associated with heart failure 17.9 % vs 4.9 % (p = .000); and post-operative atrial fibrillation, 12.4 % vs 2.9 % (p = 0.005). Lengths of ventilation, stays in ICU and in hospital were significantly higher in the AKI group.

**Conclusions:** Unlike the CK-MB profile, the hsTnT showed significant changes between both groups all over the course denoting possible delayed clearance in patients with AKI that needs to put in consideration in interpreting post-operative myocardial events.

**References**

1) Vesely DL. Natriuretic peptides and acute renal failure. Am J Physiology-Renal Physiology 2003; 285(2), F167-F177.

2) Prowle JR & Kirwan CJ. Acute Kidney Injury After Cardiac Surgery: The Injury That Keeps on Hurting?*. Critical Care Med. 2014; 42(9), 2142-2143.

## P142 Complex evaluation of endothelial dysfunction markers for prognosis of outcomes in patients undergoing cardiac surgery

### I. Mandel^1^, S. Mikheev^1^, I. Suhodolo^2^, V. Kiselev^1^, Y. Svirko^1^, Y. Podoksenov^1^

#### ^1^Research Institution for Cardiology, Tomsk, Russia; ^2^Siberian State Medical University, Tomsk, Russia

**Introductions:** The specific role of endothelin include vasoconstriction, cardiac hypertrophy and remodeling [1]. Endothelial dysfunction is reasonable to believe that there is diagnostic and prognostic value in its evaluation for outcome [2].

**Methods:** In a prospective trial (30 male of 54 ± 5) the method of prognosis of outcomes was examined. The dynamics of endothelin-1 (ET-1) level, metabolites of nitric oxide were analyzed in perioperative period; we studied it’s correlations with clinical and laboratory data. Statistical analysis was carried out in SPSS 17.0.

**Results:** Group I included 5 patients with multiple organ dysfunction syndrome in the early postoperative period. Group II included 25 patients with uncomplicated postoperative course (Table 19). The high concentration of ET-1 and low level of nitric oxide metabolites in perioperative period indicates the predominance of vasoconstrictor effect, which in its turn leads to vascular wall damage and hypoperfusion of organs and tissues.

**Conclusions:** High levels of ET-1 and nitric oxide metabolites imbalance, which characterize the functional state of the endothelium, are predictors of postoperative morbidity in cardiac surgery.

**References**

1. Rodríguez-Pascual F. et al. Pharmacol. Research.–2011.-63.-463–472.

2. Esper R.J. et al. Cardiovasc. Diabetol.–2006.-^1^5.–ð.4-22.

**Table 19 (Abstract P142).** The data is presented as Me (Q25 – Q75)

Parameter	Group I (n = 5)	Group II (n = 25)
ET-1, before surgery, fmol/ml	9.01 (5.86 – 12.48)	1.83 (0.87 – 3.12)
ET-1, the end of surgery, fmol/ml	9.23 (6.03 – 12.96)	1.53 (0.79 – 2.96)
ET-1, 24 h after surgery, fmol/ml	8.68 (5.64 – 12.06)	1.57 (0.81 – 3.07
NO3.endo, before surgery, μmol/l	9.23 (6.48 – 12.53)	13,98 (10.96 – 18.45)
NO3.endo, the end of surgery, μmol/l	3.98 (1.96 – 5.82)	10.53 (7.39 – 14.15)
NO3.endo, 24 h after surgery, μmol/l	25.9 (22.85 – 29.93)	11.28 (8.08 – 14.96)
NO2.endo, before surgery, μmol/l	1.28 (1.04 – 1.46)	1.32 (1.04 – 1.61)
NO2.endo, the end of surgery, μmol/l	0.34 (0.18 – 0.53)	1.12 (0.85 – 1.40)
NO2.endo, 24 h after surgery, μmol/l	0.86 (0.67 – 1.02)	1.37 (1.10 – 1.66)

## P143 New-onset atrial fibrillation in intensive care: incidence, management and outcome

### S. A. Jenkins, R. Griffin

#### The Hillingdon Hospitals NHS Foundation Trust, Middlesex, UK

**Introductions:** Atrial fibrillation (AF) is a common arrhythmia in critically ill patients. However, data evaluating AF in the non-cardiac intensive care setting are limited. The objective of this study was to describe the incidence, management and outcome of new-onset AF in patients admitted to non-cardiac intensive care.

**Methods:** We performed a retrospective review of consecutive patients admitted to the intensive care unit (ICU) of a district general hospital over a 10-month period. Patients with pre-existing AF and ICU admissions for routine postoperative monitoring were excluded.

**Results:** The study population consisted of 330 critically ill patients admitted to the ICU during the 10-month period. The incidence of new-onset AF was 10 % (n = 33). 55 % of patients with new-onset AF were male and mean age was 73 ± 11 years. The medical notes were available for review for 26/33 patients. Patients with new-onset AF frequently had evidence of sepsis (81 %), respiratory failure (62 %), circulatory shock (38 %) and acute kidney injury (31 %). Mean ICU day when AF occurred was 2.6 (range 1 - 10). Rhythm control strategy was used as first-line therapy for the majority of patients: 81 % received intravenous amiodarone and 12 % underwent electrical cardioversion for haemodynamic instability. 23 % received beta-blockers and 23 % digoxin as second-line therapy. 69 % of patients were anticoagulated with heparin infusion or therapeutic-dose low-molecular-weight-heparin. 31 % remained in AF at time of ICU discharge. New-onset AF was associated with longer ICU length of stay (median 6 days versus 3 days, p = 0.04) but not hospital mortality.

**Conclusions:** New-onset AF occurs in 10 % of patients admitted to non-cardiac intensive care and is associated with longer ICU length of stay. Further multicentre studies are needed to determine the optimal management and anticoagulation strategies for new-onset AF in critically ill patients.

## P144 One single spot measurement of the sublingual microcirculation during acute pulmonary hypertension in a pig model of shock

### M. S. Tovar Doncel^1^, A. Lima^2^, C. Aldecoa^1^, C. Ince^2^

#### ^1^University Hospital Rio Hortega, Valladolid, Spain; ^2^Erasmus MC, Rotterdam, Netherlands

**Introductions:** The effect of pulmonary hypertension in the sublingual microcirculation (mci) remains unclear. We described in a pig model of shock the effects of the hemodynamic changes during acute pulmonary hypertension in conjunction with sublingual mci alterations measured with the Cytocam-IDF (Indident dark-field illumination) imaging fixed in one sublingual spot.

**Methods:** Pulmonary hypertension was unexpectedly observed as a complication after intravenous infusion of contrast agent through the central venous catheter. This contrast agent can induce allergic reactions leading to massive blockage of the pulmonary arteries and increase pressure in the pulmonary capillaries. A similar response was observed in three other animals. Mean arterial pressure (MAP), right atrial pressure (RAP), and pulmonary artery pressure (mPAP) were recorded continuously. One single spot measurement of the sublingual mci was performed with the Cytocam-IDF imaging fixed on a metal arm support.

**Fig. 29 (Abstract P144).**
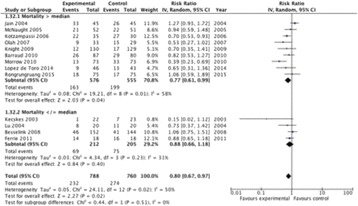
Changes in the Sublingual Microcirculation during Acute Pulmonary Hypertension

**Results:** Acute pulmonary hypertension was characterized by an increase in the mPAP, followed by high RAP and low MAP. The time course of the sublingual mci alterations was recorded simultaneously with the hemodynamic changes (Fig. 29). The progressive raise in RAP induced blood to flow backward to the large veins, inducing venous stasis and reducing microcirculatory perfusion. Despite the adrenergic response with normalization of MAP, no recovery in sublingual mci was observed.

**Conclusions:** Abnormalities in the sublingual mci during severe pulmonary hypertension were mainly associated with decrease perfusion pressure due to high RAP and hypotension. These different phases of acute hemodynamic changes could be detected with Cytocam-IDF imaging fixed in one sublingual spot.

This study was supported in part by an Innovation grant from the Dutch Kidney Foundation (grant nr. 14OIP11).

## P145 Assessment of levosimendan as a therapeutic option to recruit the microcirculation in cardiogenic shock – initial experience in cardiac ICU

### A. Taha, A. Shafie, M. Mostafa, N. Syed, H. Hon

#### Sheikh Khalifa Medical City, Abu Dhabi, United Arab Emirates

**Introductions:** The presences of microcirculatory inadequacy despite resuscitation of hemodynamics strongly correlate with microcirculatory failure and poor outcome. (1) We sought to evaluate the response of the levosimendan, as an inotrope and vasodilator, that can improve perfused capillary density in previous studies.(2) and whether these beneficial effect can be utilized during cardiogenic shock state. Recruitment of microcirculation can be detected by veno- arterial pCO2 difference (Δ pCO2) which increases earlier as the intracapillary blood velocity falls and could reflect the adequacy of microvascular blood flow. (2)

**Methods:**

Retrospective observational study including 40 patients with cardiogenic shock admitted to our cardiac ICU were enrolled between January 2013 and March 2015. Arterial and mixed-venousblood gases and hemodynamic variables were obtained at admission and every 2 hours for intial 6 hours. dPCO2, Ca-CvO2, dPCO2/Ca-CvO2 and Cv-aCO2/Da-vO2 quotients was calculated.

**Results:** Completed data sets were obtained from 40 patients , CI, cardiac output (CO), systemic vascular resistance (SVR), serum lactate, mixed venous saturation, arterio venous oxygen content difference and dPCO2. Subsequently, Ca-CvO2, (Cv-aCO2/Da-vO2) and the CI was examined and both found to be statistically significant different after starting levosimendan in cardiogenic shock patients (p < 0.001), comparing the serial measurements show statistical significant difference between initial samples and after 6 hrs. From starting levosimendan (P < 0.05)

**Conclusions:** In our clinical series, dPCO2, Ca-CvO2 in patients with cardiogenic shock was significantly improved after starting levosimendan in cardiogenic shock patients, within 6 hours of initial resuscitation; therefore, it might be reasonable to consider levosimendan as a potential therapeutic option for microcirculatory recruitment during cardiogenic shock state. Further studies needed to confirm these findings

**References**

1] De Backer D, Ospina-Tascon G, Salgado D, Favory R, Creteur J, Vincent JL (2010) Monitoring the microcirculation in the critically ill patient: current methods and future approaches. Intensive Care Med 36:1813–1825.

2] Gustavo A, Ospina-Tascon, Umaña M, et al. (2015) Can venous-to-arterial carbon dioxide differences reflect microcirculatory alterations in patients with septic shock? Intensive Care Med. 17 November 2015.

## P146 Terlipressin vs. norepinephrine in the Potential Multiorgan Donor(PMD)

### F. Righetti, E. Colombaroli, G. Castellano

#### Intensive Care Unit, St. Boniface Hospital, Verona, Italy

**Introductions:** Brain death is a serious disease that affects brain parenchyma but also all other organs. Intensivist’s task is to maintain organic homeostasis to allow PMD to reach withdrawal multiorgan in the best conditions, protecting from possible damage post neurovegetative storm (NS). NS causes severe hemodynamic instability resulting in peripheral hypoperfusion, hypotension, high values of lactates (LA), polyuria, depletion of catecholamine and stunning heart. Aim of our prospective study was to evaluate efficacy of terlipressin vs. norepinephrine in maintaining hemodynamic stability after NS [1].

**Methods:** 10 adult patients hospitalized in intensive care with acute brain injury, diagnosis of brain death, post NS, mean arterial pressure less than 65 mmHg. The patients were divided into two groups: 5 patients, group norepinephrine (infusion at 0.1 mcg/kg/min for 6 hours) and 5 patients, group terlipressin (infusion at 1.5 mcg/kg/h for 6 hours). These parameters were collected for each patients: mean arterial pressure (MAP), values of LA. The results were expressed as mean with standard deviation. For the comparison between the groups was used the Fisher test, considering significant for a p < =0.05.

**Results:** After 6 hours of treatment group norepinephrine: MAP 85 ± 4mmgh, LA 4.1 ± 0.5mMol/L while the group terlipressin: MAP 108 ± 4mmhg, LA 2.1 ± 0.2mMol/L. Comparing the two groups the p-value for each variable was: MAP p = 0.034, LA p = 0.021.

**Conclusions:** We have shown that the use of terlipressin has improved patient’s hemodynamic stability in brain death after NS, with an increased mean arterial pressure and decrease of lactates, compared to the use of norepinephrine. This has allowed an improvement in peripheral perfusion. Comparing the two groups we have reached statistical significance for each variable calculated.

**Reference**

1. Russel JA. Et al.: Vasopressin versus norepinephrine infusion in patients with septic shock. N Engl J Med. 2008 Feb 28;358(9):877-87.

## P147 Echocardiography in the potential heart donor exposed to substitution hormonotherapy

### F. Righetti, E. Colombaroli

#### Intensive Care Unit, St. Boniface Hospital, Verona, Italy

**Introductions:** The brain death is a serious disease that affects the brain parenchyma and also the other organs. Intensivist’s task is to maintain a normal organic homeostasis for the potential donor. In this way, it is possible to reach multiorgan withdrawal optimally protecting from possible damage. The aim of this prospective randomized study was the evaluation of cardiac function of potential heart donor underwent substitution hormonotherapy by serial echocardiography [1].

**Methods:** 40 adult patients(pts), admitted to the intensive care unit with acute brain injury, bilateral non reactive mydriasis, Glasgow Coma Scale 3, absence of any reflection and potential progression to brain death. Pts were divided randomly into two groups: Hormonotherapy group (20 pts) treated with substitution hormonotherapy (triiodothyronine, vasopressin, methylprednisolone, insulin) and Control group (20 pts) with standard treatment. In all pts serial transesofageal echocardiography evaluations were performed with focus on systolic function, evaluated with ejection fraction of the left ventricle with Simpson’s method. The echocardiographic exams were performed at entrance of the pts, post neurovegetative storm and within 12 hours after diagnosis brain death. The results were expressed as mean with standard deviation. For the comparison between the two groups was used the Fisher test considered significant with p ≤ 0.05.

**Results:** Pts in the two groups were statistically matched for sex (p = 0.28) and age (p = 0.37). All patients have evolved in brain death. In Hormonotherapy group baseline ejection fraction was 54 % ± 3%, 28 % ± 8% post neurovegetative storm, and 43 % ± 6% within 12 hours after brain death. In the control group the baseline ejection fraction was 49 % ± 7%, post neurovegetative storm 25 % ± 6%, and 34 % ± 5% within 12 hours after brain death. Comparing the two groups we have reached statistical significance (p = 0.028).

**Conclusions:** Comparing the two groups, we noticed both starting from a similar baseline cardiac function. During the neurovegetative storm, we recorded a similar myocardial stunning. However, in group who used substitution hormonotherapy is shown a recovery of systolic function to almost basal level within 12 hours after brain death while in the control group remains a dysfunction in systolic function. Considering these pts as potential heart donors we have shown that substitution hormonotherapy improves systolic function, despite the stunning post neurovegetative storm.

**Reference**

1. Echocardiography in the potential heart donor. Venkateswaran RV et al.: Trasplantation 2010; 89: 894-901

## P148 Machine learning can reduce rate of monitor alarms

### M. Hravnak^1^, L. C. Chen^2^, A. D. Dubrawski^2^, G. C. Clermont^1^, M. R. Pinsky^1^

#### ^1^University of Pittsburgh, Pittsburgh, USA; ^2^Carnegie Mellon University, Pittsburgh, USA

**Introductions:** Bedside alarms sound when vital sign (VS) exceed thresholds due to real instability (alerts) or artifact, leading to alarm fatigue. We used machine-learning (ML) algorithms to classify alarms as alerts or artifacts in VS data streams to assess impact of such a filter on alert rate.

**Methods:** Patients had VS monitoring data (heart rate [HR], respiratory rate [RR], oximetry [SpO2]) recorded at 1/20Hz. We partitioned data into training/validation (294 admissions; 22,980 hrs) and test sets (2,057 admissions; 156,177 hrs). Alarms are VS deviations beyond stability thresholds, and annotated as real alerts or artifact. We trained a random forest ML algorithm to discriminate alerts from artifact for long alarms (>3 min) and evaluated performance in the test set on both long and short alarms (<3 min) according to how many artifact alarms can be reduced among the total number of alarms (M1); and how many artifact alarms can be correctly filtered from the total artifact alarms (M2).

**Fig. 30 (Abstract P148).**
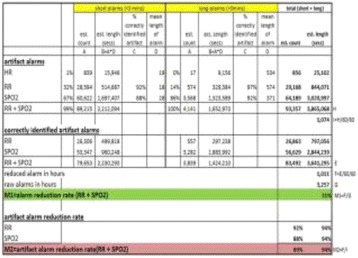
Calculation of M1 (total alarm reduction) and M2 (artifact alarm reduction)

**Fig. 31 (Abstract P148).**
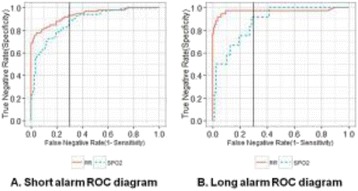
ROC diagram for ML algorithm for short (A) and long (B) alarms

**Results:** Figure 30 shows calculation of M1 (overall alarm duration reduction = 31 %) and M2 (artifact alarm duration reduction = 94 %) The number of artifact alarms in the complete data set is estimated from artifact prevalence in the annotated sample. Figure 31 is the ROC diagram for ML algorithm for both short and long alarms. The vertical line indicates proportion of artifact alarms correctly identified at a false negative rate of 30 % (i.e. % of real alerts incorrectly filtered out).

**Conclusions:** This ML filter algorithm can reduce about 94 % of artifact alarms and 31 % of overall alarms, potentially reducing alarm fatigue if applied clinically. FUNDING NIH RO1 R01NR013912.

## P149 Peripherally inserted central catheters placed in the ICU

### S. Gonzalez, D. Macias, J. Acosta, P. Jimenez, A. Loza, A. Lesmes, F. Lucena, C. Leon

#### Valme University Hospital, Seville, Spain

**Introductions:** The use of peripherally-inserted central catheters (PICCs) has notably increased in hospitalized, critically ill and ambulatory patients. However, it is important to understand the rationale and risks associated with this new technology. The aim of this study was to describe patterns of use and complications of PICCs, which were placed in the ICU setting.

**Methods:** Prospective observational study carried out in a 500-bed general hospital with a 14-bed ICU, in which a specialized unit for catheter insertion is available. PICCs were implanted always in the middle third of the arm, following a protocol that includes strict sterile insertion procedure and echo-radiological control. Data of patients with PICCs inserted from January 2013 to April 2015 were collected, including demographics, characteristics of insertion, complications and outcomes. Descriptive data included frequencies and percentages for categorical variables and mean and standard deviation (SD) or median and interquartile range (IQR) (25th-75th percentile) for quantitative variables.

**Results:** A total of 677 PICCs were placed. The mean age of the patients was 59 (15) years, with a 50.2 % of males. Patients came from a hospital ward (n = 402, 59.4 %) and the remaining from home (n = 275, 40.6 %). More than half of the patients (n = 351, 51.8 %) belonged to the Haemato-Oncology Service, 79 (11.7 %), to Internal Medicine and 46 (6.8 %) to ICU. 293 (43.3 %) patients had solid tumours, 128 (18.8 %) haematological neoplasms and 43 (6.4 %) inflammatory bowel disease. PICCs were mainly used for chemotherapy and parenteral nutrition in 347 (51.3 %) and 156 (23 %) patients, respectively. No major complication related to insertion was registered. The mean length of use was 70 (19-188) days with a sum of 79,537 days of catheter placement. Reasons for withdrawal included end of treatment in 334 (49.3 %), death in 160 (23.6 %), catheter-related bloodstream infection (CRBSI) 41 (6.1 %), and deep venous thrombosis (DVT) in 5 (0.7 %). A total of 57 CRBSI were recorded with incidence density rates of 0.71‰ catheter-days. A total of 15 symptomatic DVT (2.21 %) were recorded.

**Conclusions:** Many patients in non-intensive care unit settings receive PICCs; most of them were haemato-oncological patients, mainly for the administration of chemotherapy. There were no relevant complications related to catheter insertion. The mean time of use was 70 days, and the reason for withdrawal was the end of treatment or death. An incidence density of CRBSI of 0.7‰ catheter-days was recorded with few symptomatic DVT.

## P150 Recordings of abnormal central venous pressure waveform morphology during an episode of pulmonary hypertension in a porcine shock model

### M. S. Tovar Doncel^1^, C. Ince^2^, C. Aldecoa^1^, A. Lima^2^

#### ^1^University Hospital Rio Hortega, Valladolid, Spain; ^2^Erasmus MC, Rotterdam, Netherlands

**Introductions:** A normal central venous pressure (CVP) waveform contains five components. These components include three peaks (a, c, v) and two descents (x, y). The characteristics and amplitude of the CVP waveform components change significantly during cardiovascular distress and its visualization can provide invaluable additional hemodynamic information. We described CVP tracings recorded during an episode of pulmonary hypertension in a porcine model of shock, aiming to characterize the CVP waveform morphology in conjunction with the simultaneous hemodynamic alterations.

**Methods:** This is an animal case-report in which the CVP waveform was recorded during an ongoing experimental porcine shock model monitored with Swan-Ganz catheter. Pulmonary hypertension was unexpectedly observed as a complication after intravenous infusion of a contrast agent through the central venous catheter. A similar response was observed in three other animals. Together with the CVP tracings, recordings also included cardiac output, systolic/diastolic arterial pressure, heart rate, and systolic/diastolic arterial pulmonary pressures.

**Fig. 32 (Abstract P150).**
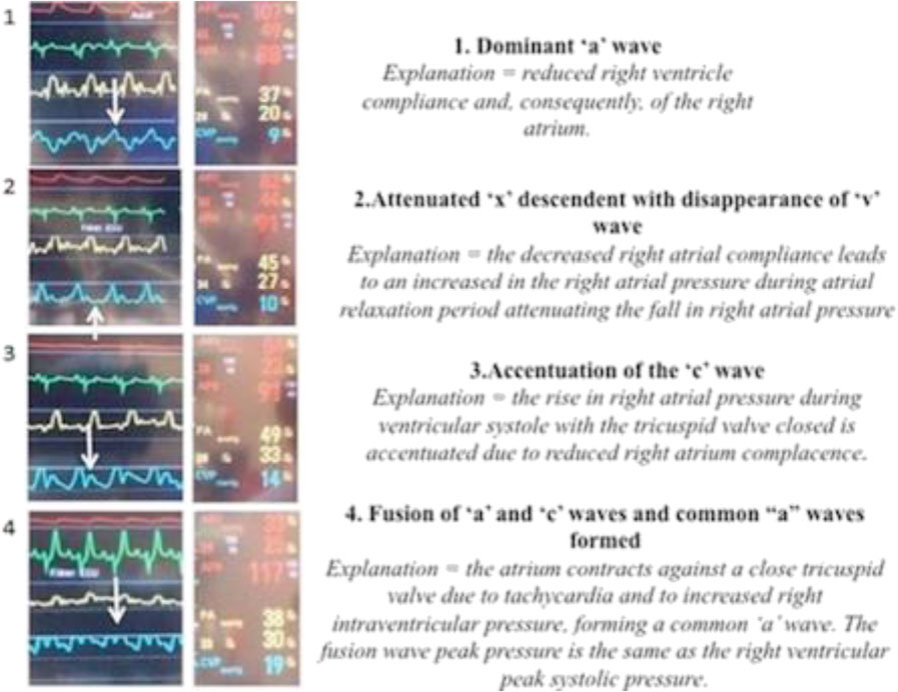
Central venous pressure waveform tracings

**Results:** The episode of pulmonary hypertension lasted for 3-5 min with spontaneous recovery. The CVP changed from a baseline value of 4-6 mmHg to 16-18 mmHg. The CVP waveform tracings showed abnormal large ‘a’ wave complex resembling a cannon ‘a’ wave of CVP. Figure 32 shows the different patterns of the CVP morphology with the different components of the tracing recorded during the entire episode of pulmonary hypertension. A simultaneous ECG is shown to demonstrate the timing of the different components.

**Conclusions:** The abnormal CVP waveform morphology during an episode of pulmonary hypertension shows a series of waves that can provide invaluable additional hemodynamic information.

This study was supported in part by an Innovation grant from the Dutch Kidney Foundation (grant nr. 14OIP11).

## P151 Ultrasound guided central venous access technique among French intensivists

### M. Bastide^1^, J. Richecoeur^2^, E. Frenoy^3^, C. Lemaire^4^, B. Sauneuf^5^, F. Tamion^6^, S. Nseir^7^, D. Du Cheyron^8^, H. Dupont^1^, J. Maizel^1^

#### ^1^CHU Amiens; Amiens, France; ^2^CH Beauvais, Beauvais, France; ^3^Réanimation polyvalente, Le Havre, France; ^4^CH Roubaix, Roubaix, France; ^5^CH Cotentin, Cherbourg, France; ^6^CHU Rouen, Rouen, France; ^7^CHU Lille, Lille, France; ^8^CHU Caen, Caen, France

**Introductions:** The use of ultrasound is recommended to secure central venous catheter placement in the ICU. However several surveys showed that only 50 % of the CVC procedures are performed using the ultrasound guided technique. The reason why physicians continue to use landmarks were the lack of formation and the absence of ultrasound device. We designed a survey to elicit information on physician’s characteristics, experience in CVC placement, training on ultrasound technique, the use of ultrasound for CVC placement, reasons for non-use of ultrasound and their opinion on the necessity to continue to teach the landmark technique to residents.

**Methods:** This survey (14 questions) has been electronically addressed by Email to every physician belonging to the BoReal group of research (8 university’s and 20 community’s ICU in the North-west of France). The survey was sent to the 289 physicians (166 seniors and 121 residents) working in those ICUs.

**Results:** We received 190 responses (response rate 66 %). Among the respondents, 66 % were less than 40 years old, 34 % were residents, 41 % presented an experience in CVC placement < =5 years and 53 % declared putting more than 1 CVC per week. 71 % of the residents declared having learned both landmark and ultrasound guided techniques during their residency. Only 18 % reported using always the ultrasound to put CVC. The main reasons why they don’t use ultrasound were 1) they think they don’t need it (36 %), 2) the ultrasound was not available (33 %) while 3 % declare the absence of ultrasound device in their setting and 3) 11 % reported the absence of formation on ultrasound technique. Coagulation abnormalities (64 %), obesity (54 %) and anatomical difficulties (52 %) were the main motivations to use ultrasound reported. 53 % of the respondents declared having been confronted during the last 12 months to an urgent situation where the ultrasound was not available quickly enough. Finally 91 % think that the landmark technique should still be taught to the residents.

**Conclusions:** Our survey shows that a large majority of the intensivists still perform landmark procedures while almost every physician received a formation on the ultrasound technique. Ultrasound device is present in the large majority of the institution however their availability is still scarce. Despite the several guidelines, physicians still don’t use ultrasound for every procedure and think that landmark technique should still be taught to residents.

## P152 Predictive ability of the Pv-aCO2 gap in patients with shock

### M. Shaban, R. Kolko, N. Salahuddin, M. Sharshir, M. AbuRageila, A. AlHussain

#### King Faisal Specialist Hospital & Research Center, Riyadh, Saudi Arabia

**Introductions:** Central venous-to-arterial carbon dioxide difference (Pv-aCO2 or CO2 gap) is recognized as an index of global tissue perfusion. Additionally, the ratio of Pv-aCO2 by arterial-to-central venous O2 content difference (Ca-vO2), has been described as indicative of ¡®anerobiosis¡¯ in shock. The aim of this study was to demonstrate the predictive abilities of the Pv-aCO2 gap and Pv-aCO2/Ca-vO2 ratio in patients undergoing resuscitation from shock.

**Methods:** Critically ill patients with shock who required vasopressors or inotropes were included in a prospective, observational study. Serial measurements of Pv-aCO2 gap, lactate, and Ca-vO2 were obtained at the time of starting the vasopressor/inotrope (time 0), then at times 3, 6 and 12 hours. The Study protocol was approved by the Hospital Research Ethics Committee (RAC No. 2151 048).

**Results:** Fifty patients were enrolled. The mean APACHE II score was 26.9 ¡À 7.3. Thirty patients (60 %) had septic shock, 15 patients (30 %) had combined septic and cardiogenic shock, 3 patients (6 %) had hemorrhagic shock, and two patients (4 %) had cardiogenic shock. 28-day mortality rate was 62 % (31 patients). Fifteen patients (30 %) developed acute kidney injury.

Patients surviving to day-28 had a lower Pv-aCO2 gap at time 0 (7.5, IQR 7 vs. 4.8, IQR 5, p = 0.007), and demonstrated greater clearance of the Pv-aCO2 gap during resuscitation (-0.75 IQR 4.8 vs. +0.75 IQR 5.2, p =0.024). A cut-off value of ¡Ý 6 mmHg significantly predicted 28 day mortality (75 % vs. 45.5 %, p =0.033). In a subgroup of patients in whom a resuscitative target of ScvO2 70 % was achieved, a higher Pv-aCO2 gap was significantly associated with lower 28-day survival (6.7 IQR 6 vs. 3.7 IQR 4, p =0.009).

Pv-aCO2/Ca-vO2 ratios were significantly lower in patients surviving the ICU and until day-28 of follow up; this relationship was observed both at time 0 (0.21,IQR 0.17 vs. 0.27, IQR 0.22, p =0.042) and at time 3 (0.19, IQR 0.1 vs. 0.24,IQR 0.07, p =0.047) for ICU mortality, and (0.27, IQR 0.19 vs. 0.18,IQR 0.12, p =0.013) and (0.27, IQR 0.08 vs. 0.19, IQR 0.10,p = 0.011) for 28-day survival.

**Conclusions:** This study suggests that Pv-aCO2 gap and Pv-aCO2/Ca-vO2 ratio can be used to predict mortality in shock, especially in patients for whom the usual goals of resuscitation have been achieved. A Pv-aCO2 gap value of 6 mmHg may be used as a reasonable cut-off point.

## P153 Comparison of echocardiography and pulmonary artery catheter measurements of hemodynamic parameters in critical ill patients

### P. Mercado, J. Maizel, L. Kontar, D. Titeca, F. Brazier, A. Riviere, M. Joris, T. Soupison, B. De Cagny, M. Slama

#### CHU Amiens, Amiens, France

**Introductions:** Measurements of cardiac output (CO), pulmonary artery occlusion pressure (PAOP) and systolic pulmonary artery pressure (SPAP) remain important parameters for the management of critically ill patients. Pulmonary artery catheter (PAC) with thermodilution technique has been considered the clinical standard in measurements of CO. However, it is an invasive technique that can be associated with complications. Transthoracic echocardiography (TTE) is a non-invasive technique allowing to calculate the CO and the PAPS. We decided to compare the CO and SPAP measured by TTE and by PAC.

**Methods:** We prospectively included all patients monitored with PAC, sedated and under mechanical ventilation in our ICU. We excluded patients with arrhythmias, severe valvulophathy and poor echogenicity.

The CO, PAOP, right auricular pressure (RAP) and SPAP were measured using the PAC (-PAC). CO-PAC was an average of three thermodilutions. The TTE CO (COTTE) was derived from the Doppler stroke volume, the E/A ratio from the transvalvular mitral flow and the Ea from the mitral annular TDI. SPAP was estimated from tricuspid gradient (SPAPecho) plus RAP (estimated by inferior vena cava diameter). Measurements made with TTE and PAC were performed simultaneously by two experienced operators. A linear regression and a Bland-Altman analysis were performed.

**Results:** 22 patients (36 measurements) were monitored with PAC, age 64y/o(60-70), SAPS 61(60-79), ejection fraction (EF) 58 %(46-61). The linear regression between COTTE and COPAC was R2 = 0.78 (P < 0.001). The Bland and Altman analysis showed a bias of -0.4 l/min and limits of agreement from -2.8 to 1.9 l/min. The linear regression between SPAPecho and SPAP-PAC was R2 = 0.52 (P < 0.001), the linear regression improves when SPAPecho was calculated from tricuspid gradient plus RAP measured by PAC R2 = 0.62 (P < 0.001). The Bland and Altman analysis showed a bias of -3.7 mmHg and limits of agreement from -18.1 to 10.7 mmHg. Linear regression between E/A and PAOP, E/Ea and PAOP were weak, R2 = 0.33 (P < 0.001) and R2 = 0.42 (P < 0.001) respectively.

**Conclusions:** This study confirms that COTTE correlates well with the measurement of COPAC in critical care patients sedated under mechanical ventilation. The TTE estimation of SPAP appears also reliable. But the TTE estimation of PAOP (E/Ea and E/A) in this population of patient with unselected EF is not consistent.

## P154 The volume clamp method for noninvasive cardiac output measurement in postoperative cardiothoracic surgery patients: a comparison with intermittent pulmonary artery thermodilution

### J. Wagner, A. Körner, M. Kubik, S. Kluge, D. Reuter, B. Saugel

#### University Medical Center Hamburg-Eppendorf, Hamburg, Germany

**Introductions:** The CNAP technology (CNSystems Medizintechnik AG, Graz, Austria) enables noninvasive continuous recording of the arterial pressure waveform based on the volume clamp method. Recently, the manufacturer released a novel algorithm for measuring cardiac output (CO) using pulse contour analysis of the arterial waveform. In this study, we compared CO measurements with CNAP (CNCO) with invasive CO measurements using intermittent pulmonary artery thermodilution (pulmonary artery catheter (PAC); PAC-CO) in postoperative cardiothoracic surgery patients.

**Methods:** In this interim analysis, simultaneously obtained CNCO and PAC-CO measurements were analyzed in 30 patients during the first hours after cardiothoracic surgery. We performed 3 independent sets of 5 consecutive thermodilution measurements each per patient. The average of the 3 closest of the 5 PAC-CO measurements was used for comparison with the average of the corresponding CNCO values.

Three pairs of measurements were excluded due to artifacts resulting in 87 paired measurements for analysis. In addition, 25 cardiac output–modifying maneuvers were analyzed to evaluate trending ability. We conducted 2 separate comparative analyses: 1) CNCO calibrated to the first simultaneously measured PAC-CO value (CNCOcal) vs. PAC-CO and 2) CNCO auto-calibrated to biometric patient data (CNCObio) vs. PAC-CO. Agreement between the two methods was statistically assessed by Bland-Altman analysis and by calculating the percentage error (PE). For evaluating trending ability, the concordance rate (CCR) was calculated. An exclusion zone of 0.5 L/min was applied.

**Results:** For CNCOcal, the Bland-Altman analysis resulted in a mean difference of -0.2 L/min, a standard deviation of ±0.5 L/min and limits of agreement of -1.1 to +0.7 L/min. The PE and CCR were 18 % and 100 %, respectively. For CNCObio, the Bland-Altman analysis resulted in a mean difference of +0.5 L/min, a standard deviation of ±1.1 L/min and limits of agreement of -1.6 to +2.6 L/min. The PE and CCR were 43 % and 94 %, respectively.

**Conclusions:** In this clinical study in patients after cardiothoracic surgery, CNCOcal showed good agreement (PE 18 %) and good trending ability (CCR 100 %) when compared with intermittent pulmonary artery thermodilution. For CNCObio, we observed a higher PE (43 %) but acceptable trending ability (CCR 94 %).

## P155 Hemodynamic monitoring in patients with septic shock (SS) – CPCCO (continuous pulse contour cardiac output) vs. TEE (transesophageal echocardiography)

### E. Colombaroli, F. Righetti, G. Castellano

#### Intensive Care Unit, St. Boniface Hospital, Verona, Italy

**Introductions:** A reliable hemodynamic monitoring and a correct interpretation of the data are of crucial importance in the patient (pts) with SS to choose the strategy of treatment in the Intensive Care Unit (ICU). The aim of this prospective study was to compare two techniques of hemodynamic monitoring to assess which is more reliable in the management of pts in SS[1].

**Methods:** 20 adult pts in ICU for SS. All pts were monitored with CPCCO Pulsion Medical Systems and serial TEE have been performed. The cardiac output(CO;L/min) was measured continuously with the method CPCCO(triple injection of 15 ml of 0.9 % saline solution at 4 °C in the CVC, thermo dilution curve measured in the femoral artery through the catheter thermistor dedicated) and simultaneously with TEE(LVOT area multiplied the integral speed/time of aortic flow multiplied by the heart rate) at the entrance, after fluid challenge with 500 ml of crystalloid and during the use of vasopressors and/or inotropic drug(VId). Same operator measured echocardiographic CO without being aware of the CO supplied by CPCCO. The results were expressed as mean with standard deviation. For comparison and correlation between measurements were used linear regressions according to the Bland-Altman method. Statistical significance was considered at p ≤ 0.05.

**Results:** 120 measurements were performed. 60 with CPCCO and 60 with the TEE. 40 measurements were realized at the entrance, 20 with CPCCO(1.8 ± 0.2) and 20 with TEE(1.75 ± 0.3), 40 after fluid challenge, 20 with CPCCO(2.5 ± 0.3) and 20 with TEE(2.7 ± 0.2), 40 during the use of vasopressors and/or inotropic drug including 20 with CPCCO(4.5 ± 0.3) and 20 with TEE(4.4 ± 0.2). There is a good correlation and a limited bias between measurements provided by CPCCO and by TEE, both at the entrance(r = 0.992,bias = 0.15), after fluid challenge (r = 0.954,bias = 0.24), both during the use of VId (r = 0.967,bias = 0.33). These values were statistically significant (p = 0.026).

**Conclusions:** This study showed that there is a good correlation between the data provided by CPCCO and TEE. Both methods can be used reliably for hemodynamic monitoring in SS.

**Reference**

1. V. De Castro et al. Comparison of stroke volume and stroke volume respiratory variation measured by the axillary artery pulse-contour method and aortic Doppler echocardiography in patients undergoing aortic surgery. Br J Anaesth 2006; 97:605-10

## P156 Cardiac output measurement with transthoracic echocardiography in critically ill patients: a pragmatic clinical study

### T. Tran, D. De Bels, A. Cudia, M. Strachinaru , P. Ghottignies, J. Devriendt, C. Pierrakos

#### Brugmann Hospital, Brussels, Belgium

**Introductions:** The assessment of cardiac output by echocardiography is a frequent clinical practice. However, this method shares important limitations which can lead to inappropriate therapies.

**Methods:** We retrospectively evaluated transthoracic echocardiography examinations of critically ill patients performed between 2013-2014. The stroke volume (SV) was calculated using left ventricular outflow tract diameter and flow velocity time integral (DT) and was compared to stroke volume estimated from the difference between the end diastolic and end systolic volume measured by Simpson biplane method (S2D). We excluded patients with atrial fibrillation and any significant valvulopathy. Low end diastolic value (EDV) was considered as a volume <70 cc and low ejection fraction (EF) was defined <45 %.

**Results:** We evaluated 147 exams. Twenty patients were excluded because of atrial fibrillation and 41 because of a significant valvulopathy. A total 86 pairs of stroke volumes measured by the two methods were evaluated. The r value was 0.51, r^2 = 0.25 and p < 0.01 by regression analysis. The mean bias of stroke volume measured with DT over that estimated with S2D was 29 % (95 % CI 21.4-37.5, p < 0.01). There was a negative correlation of this bias with mean SV (r^2 = 0.09, p < 0.01). A negative linear correlation was found between the bias and the end-diastolic volume and EF but not with end-systolic volume (r^2 = 0.21 p < 0.01, r^2 = 0.15 p < 0.01 and r^2 = 0.01 p = 0.93, respectively). A higher bias was observed in patients with low EF (46 % ± 33 vs 25 % ± 36 p0.03) and in patients with low end-diastolic volume (64 % ± 33 vs 20 % ± 31 p < 0.01).

**Conclusions:** A significant overestimation of stroke volume measured with Doppler ultrasound by transthoracic echocardiography compared to S2D was observed in critically ill patients. This overestimation was more prominent between the patients with low end-diastolic volume and/or low EF. Given that none of the two methods that we used can be considered as gold standard a further evaluation of DT should be made in this group of patients. Conversely, in every day clinical practice, the combination of these two methods may be an easy way to identify possible errors in cardiac output measurements.

## P157 Left ventricular outflow tract velocity time integral correlates with stroke volume index in mechanically ventilated patients

### Ó. Martínez González^1^, R. Blancas^1^, J. Luján^2^, D. Ballesteros^1^, C. Martínez Díaz^2^, A. Núñez^3^, C. Martín Parra^1^, B. López Matamala^1^, M. Alonso Fernández^1^, M. Chana^1^

#### ^1^Hospital Universitario del Tajo, Aranjuez, Spain; ^2^Hospital Universitario Príncipe de Asturias, Alcalá de Henares, Spain; ^3^Hospital Universitario de San Carlos, Madrid, Spain

**Introductions:** Ultrasound is a non invasive and bedside accessible method of cardiac function monitoring. Left ventricular outflow tract (LVOT) velocity time integral (VTI) can be measured in most critical care patient and is an useful tool for this purpose. Calculations of cardiac output needing aortic valve area are strongly dependent on skills and observers. We hypothesize that LVOT-VTI is an accurate estimation of stroke volume index (SVI). The objective of the study was to assess the correlation between SVI and LVOT-VTI.

**Methods:** Three ICU from different university hospitals participated in the study. Consecutive patients with haemodynamic monitoring on mechanical ventilation were included. Patient with aortic valve regurgitation or dynamic stenosis on the LVOT were excluded from the study. LVOT-VTI was measured by pulsatile doppler echocardiography. Five measurements of LVOT-VTI were obtained on each patient. Mean and maximum values were recorded. Simultaneously, five measurement of SVI by floating pulmonary artery catheter (PAC) or Pulse Induced Contour Cardiac Output (PiCCO) thermodilution methods were obtained and the mean was recorded. Mean and maximum LVOT-VTI were correlated with mean invasive SVI. Statistical analysis: correlation was assessed by Pearson correlation index and intraclass correlation coefficient (ICC).

**Results:** One hundred and two paired measurements were recorded in thirty two patients. One patient was excluded from the study due to the difficulty to obtain an adequate acoustic window. Twenty one patients were monitorized by PiCCO and eleven by PAC. The Pearson correlation index between LVOT-VTI en SVI was r = 0,770 (p < 0,001). This correlations was similar for measurements taken by PiCCO (r = 0,736, p < 0,001) but worsened when PAC was used to calculate SVI (r = 0,470, p = 0,004). Correlation was acceptable for patients on atrial fibrillation (r = 0,619, p < 0,001).

**Conclusions:** LVOT-VTI could be useful as a non-invasive simple method to estimate SVI in most of mechanically ventilated patients. Larger studies are needed to assess factors influencing on this correlations, such as the measurements performed by PAC.

**Reference**

Evangelista A, et al. Cardiac index quantification by Doppler ultrasound in patients without left ventricular outflow tractabnormalities. J Am Coll Cardiol 1995; 25: 7106

## P158 Transpulmonary thermodilution (TPTD) derived from femoral vs. jugular central venous catheter: validation of a previously published correction formula and a proprietary correction formula for global end-diastolic volume index (GEDVI)

### W. Huber, M. Eckmann, F. Elkmann, A. Gruber, I. Klein, RM Schmid, T. Lahmer

#### Klinikum rechts der Isar; Technical University of Munich, Munich, Germany

**Introductions:** Usually TPTD for measurement of global end-diastolic volume index (GEDVI) is performed by indicator injection via the jugular or subclavian vein. If this access is not feasible, femoral access can be used. However, several studies demonstrated significant overestimation of GEDVI due to the additional volume of V. cava inferior. One of these studies provided a correction formula (1). As a consequence, one of the manufacturers of commercially available TPTD-devices requires information about the venous catheter position, and correction of GEDVI in case of femoral access can be assumed.

**Methods:** It was the aim of our study to compare GEDVI derived from TPTD using jugular venous access (GEDVI-jug) to uncorrected (GEDVI-fem-uncor) and corrected (GEDVI-fem-cor) GEDVI as calculated by the PiCCO device and to GEDVI-form calculated from the previously suggested formula (1).

We recorded 31 datasets in 15 patients equipped with both jugular and femoral venous access. Each dataset consisted of three triplicate TPTDs using the jugular venous access as the gold standard and the femoral access with and without the information about the femoral indicator injection. (Bland-Altman analysis; Wilcoxon-test for unpaired samples; SPSS 23.0).

**Results:** 15 patients, 9 male, 6 female, age 60 ± 15 years, 174 ± 9 cm, 83 ± 17 kg. GEDVI-fem-uncor was significantly higher compared to GEDVI-jug (1026 ± 263 vs. 765 ± 145 ml/m^2^; p < 0.001) with a bias of +260 ± 182 ml/m^2^ and a percentage error (PE) of 40.6 %. GEDVI-fem-cor was slightly different to GEDVI-jug (811 ± 159 vs. 765 ± 145 ml/m^2^; p = 0.025) with a PE of 29.6 % and a bias of 46 ± 117 ml/m^2^ which was significantly lower (p < 0.001) than the bias for GEDVI-fem-uncor. GEDVI-form was not significantly different from GEDVI-jug (797 ± 162 vs. 765 ± 145 ml/m^2^; p = 0.063) with a PE of 26.1 % and a bias of 31 ± 108 ml/m^2^ which was significantly lower (p < 0.001) than the bias for GEDVI-fem-uncor, but not than the bias for GEDVI-fem-cor (p = 0.557).

**Conclusions:** This study confirms that GEDVI derived from uncorrected femoral indicator injection TPTD markedly overestimates GEDVI and also results in inacceptable imprecision. Correction by the PiCCO-device and by the formula recently suggested (1) result in acceptable accuracy and precision of GDVII derived from femoral TPTD. The recently suggested formula provided the lowest bias and PE values.

**Reference**

(1) Saugel, Huber et al., Crit Care 2010; 14:R95

## P159 Venous return driving pressure and resistance in acute blood volume changes

### P. W. Moller^1^, S. Sondergaard^2^, S. M. Jakob^1^, J. Takala^1^, D. Berger^1^

#### ^1^Department of Intensive Care Medicine, University Hospital Bern, Bern, Switzerland; ^2^Institute of Clinical Sciences at the Sahlgrenska Academy, University of Gothenburg, Sahlgrenska University Hospital, Gothenburg, Sweden

**Introductions:** Guyton’s venous return model helps to interpret circulatory response to blood volume changes. The model implies that venous return depends on the venous return driving pressure (VRdp), i.e. the difference between mean systemic filling pressure (MSFP) and right atrial pressure (RAP), and resistance to venous return (RVR). We studied VRdp and RVR response to hypo- and hypervolemia in pigs with intact circulation.

**Methods:** We measured in anesthetized pigs (n = 10; 2 early deaths) pulmonary artery blood flow (Q_PA_; ultrasonic flow probe), RAP and MSFP (venous pressure at zero flow during right atrial balloon occlusion; MSFP_RAO_) at *baseline*, after bleeding of 9 mL/kg body weight (*hypovolemia*) and after retransfusion of the shed blood and an equal amount of hydroxyethyl starch (*hypervolemia*). Blood volume (BV; indocyanine green dye dilution) was measured at *baseline* and *hypervolemia* and calculated for *hypovolemia* as BV_*baseline*_ minus shed volume. Stressed volume (V_s_) was defined from systemic vascular compliance (C_vasc_ = Δ BV/Δ MSFP_RAO_) at MSFP_RAO_ = 0. Statistics: analysis of variance for repeated measures.

**Results:** [mean (SD); n = 8]. BV (mL/kg) was 98 (16) at *baseline*, 89 (15) in *hypovolemia* and 113 (21) in *hypervolemia* (p = .001). The respective MSFP_RAO_ (mmHg) was 13.0 (2.8), 10.8 (2.2) and 16.4 (3.0), VRdp 7.0 (2.4), 5.7 (1.7) and 8.2 (2.2); Q_PA_ changed in parallel (p < .001 for all). RVR did not change (p = .49). C_vasc_ was 3.2 (.8) mL × mmHg^-1^ × kg^-1^; linear regression r^2^ = .96 (.04). The respective V_s_ before bleeding was 42 (9) mL × kg^-1^, or 43 (10)% of the total blood volume.

**Conclusions:** The parallel changes in BV, VRdp and Q_PA_, estimated by mutually independent methods, support the Guytonian model and stress the importance of RAP as backpressure to venous return. The linear relationship between changes in BV and MSFP_RAO_ indicate a constant C_vasc_ and that changes in BV translated fully into change in V_s_.

## P160 Dynamic arterial elastance calculated with lidcoplus monitor does not predict changes in arterial pressure after a fluid challenge in postsurgical patients

### D. Bastoni, H. Aya, L. Toscani, L. Pigozzi, A. Rhodes, M. Cecconi

#### St George’s Healthcare NHS Trust, London, UK

**Introductions:** Dynamic elastance (EaDyn), calculated as the ratio between Pulse Pressure Variation (PPV) on Stroke Volume Variation (SSV) has been proposed as a predictor of change in arterial pressure in response to a fluid challenge. We want to assess the predictive value of EaDyn calculated with LiDCOplus cardiac output monitor in post-operative patients.

**Methods:** In this prospective observational study 38 fluid responsive patients were monitored with an arterial catheter, cardiac output (CO) monitor (LiDCOplus). Haemodynamic data was recorded one minute before the fluid challenge and one minute after. 250 mL of crystalloids were infused intravenously over 5 minutes. Changes in mean arterial pressure (∆MAP) systolic arterial pressure (∆Sys) and diastolic arterial pressure (∆Dias) were analysed in a regression models against the values of EaDyn , at baseline, adjusted by baseline values of the haemodynamic of interest (i.e. MAP) and the presence of mechanical ventilation.

**Results:** There is no evidence that EaDyn at baseline predicts the ∆MAP (p = 0.17), ∆Sys (p = 0.21) or ∆Dias (p = 0.17). The mechanical ventilation was a significant predictor of changes in MAP (p = 0.003), Systolic (p = 0.001) and Diastolic (p = 0.001).

**Conclusions:** EaDyn calculated with LiDCOplus and adjusted by baseline arterial pressure does not predict changes in arterial pressure after a fluid challenge in post-operative patients.

## P161 Analysis of duration of post-operative goal-directed therapy protocol

### C. Ostrowska, H. Aya, A. Abbas, J. Mellinghoff, C. Ryan, D. Dawson, A. Rhodes, M. Cecconi

#### St George’s Healthcare NHS Trust, London, UK

**Introductions:** The purpose of this study is to describe the timing for achieving targets of the post-operative goal-directed therapy (GDT) protocol in postoperative patients admitted to ICU in St George’s Hospital, in order to explore if the duration of this intervention can be reduced.

**Methods:** This is a retrospective study of the post-operative GDT protocol, which basically consist of administration of fluids and inotropes in order to achieve an oxygen delivery index of 700 mL/min/m2. Data from a randomly selected month were collected and compared with the data from the audit in 2009. Data was graphically and analytically explored. Continuous variables are presented as means and standard deviation, categorical data is presented as number and proportions. Means are compared with student t test. Statistical significance is considered with p < 0.05.

**Results:** 83 patients (74.1 %) were considered suitable for GDT. The surgery name or type of surgery was not documented in 55.4 %. Oxygen delivery index (DO2I) significantly increased from baseline to the maximal level (559.27 ± 182.31 to 679.91 ± 216.27, p = < 0.0001), but did not change significantly from baseline to the value at the end of the protocol (559.3 ± 21.05 vs 548.4 ± 22.37, p = 0.7243). Maximal DO2I was spontaneously achieved by 21.5 % of cases. 13.9 % of patients achieved the maximal DO2I in the 8th hour. In general, the maximal DO2I was achieved at the 4th hour, which is the same timing observed in 2009.

**Conclusions:** The current post-operative optimisation protocol is safe and effective. At the current stage, duration of the protocol cannot be safely reduced.

## P162 Hemodynamic optimization – back to square one?

### M. Cronhjort^1^, O. Wall^2^, E. Nyberg^2^, R. Zeng^3^, C. Svensen^2^, J. Mårtensson^4^, E. Joelsson-Alm^2^

#### ^1^Karolinska Institutet, Stockholm, Sweden; ^2^Karolinska Institutet, Stockholm, Sweden; ^3^Wenzhou Medical University, Wenzhou, Zheijang, China; ^4^Department of Intensive Care, Austin Hospital, Melbourne, VIC, Australia

**Introductions:** The aim of the study was to assess whether hemodynamic optimization by protocols based on hemodynamic monitoring reduces mortality in critically ill patients.

**Methods:** We performed a systematic review and meta-analysis according to the Cochrane Handbook for Systematic Reviews of Interventions. The study was registered in the PROSPERO database (2015:CRD42015019539). We searched the PubMed, Embase and CENTRAL databases for articles in English. Inclusion criteria were RCTs studying adult patients with critical illness, treated in an intensive care unit. The intervention should be protocolized and based on results from hemodynamic measurements, defined as cardiac output, stroke volume, stroke volume variation, oxygen delivery, central venous oxygenation (ScvO2) or mixed venous oxygenation (SvO2). The control group should be treated without any structured intervention mentioned above. Exclusion criteria were pediatric, animal and perioperative studies. Primary outcome was mortality; secondary outcomes were weight gain or fluid balance. Risk of bias was assessed independently of each other by two investigators. Statistical analysis was performed by RevMan 5.3 software.

**Results:** Preliminary results: Out of 575 screened papers, eight RCTs meeting the inclusion criteria were found. None of the eight studies were completely blinded. A total of 5323 patients were enrolled in the included studies. There was no statistically significant difference in mortality between the groups. Mortality in the hemodynamic intervention group was 23.6 % (630/2664 patients) vs. 24.1 % (641/2659 patients) in the control group, OR 0.96 (95 % CI 0.82-1.11) P = 0.95. I2 = 19 %.

**Conclusions:** There is no evidence in the literature that hemodynamic monitoring combined with a structured intervention reduces mortality in critically ill patients. To investigate if mortality can be reduced by individualized hemodynamic management the monitoring has to be reliable, the intervention must be directed towards a meaningful treatment goal and the controls should receive standard of care.

## P163 Effectiveness of fluid thoracic content measurement by bioimpedance guiding intravascular volume optimization in patients with septic shock

### M. Aguilar Arzapalo, L. Barradas, V. Lopez, M. Cetina

#### Hospital O’horan, Mérida, Mexico

**Introductions:** Appropriate resuscitation and intravascular volume optimization are treatment cornerstone in septic shock. Optimization requires invasive monitoring and static measurements, due to it, is necessary a non invasive, fast and dynamic method. Thoracic fluid content (TFC) by thoracic bioimpedance surge as a monitoring system that fulfils the requirements: non invasive, easy placement and dynamic.

**Methods:** Sixty patients were enrolled, divided in two groups with 30 subjects each, admitted at ICU with septic shock diagnosis. F Group was formed by patients in whom TFC was measured by thoracic bioimpedance, in order to guide intravascular volume optimization; C Group patients were optimized following “Surviving Sepsis Campaign Guidelines”, recording mortality at ICU discharge and at 28 days, mechanical ventilation length and ICU length of stay.

**Results:** Based on TFC F Group obtained a faster and higher Mean Arterial Pressure (MAP) increasing up to 42.5 % at 6 hours, compared to C Group where MAP arise only 19.5 % at 6 hours (p:<0.05). Due to lactate clearance F Group, obtained a total clearance of 54.4 % and 66.6 % at 6 and 12 hours, on C Group clearance just came up to 25.3 % and 35.8 % at 6 and 12 hours (p: 0.001). A higher mortality at ICU discharge was reach on C Group with 33.4 % vs 26.7 % (p: 0.001) from F Group, 28 day mortality was 36.5 % and 27.8 % (p:0.003) on C and F Group respectively.

**Conclusions:** Measurement of thoracic fluid content by thoracic bioimpedance is effective while optimizing intravascular volume in septic shock.

**Reference**

Facchini C, Malfatto G, et al. Lung ultrasound and transthoracic impedance for noninvasive evaluation of pulmonary congestion in heart failure. JCM 2015 Ene;(10);2459

**Fig. 33 (Abstract P163).**
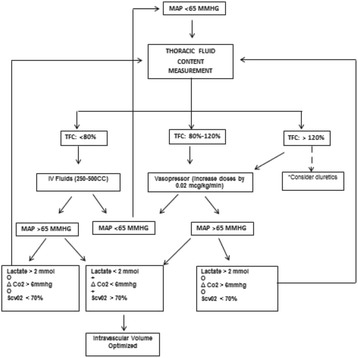
Intravascular Optimization Algorithim

## P164 A systematic review on the role of internal jugular vein ultrasound measurements in assessment of volume status in critical shock patients

### N. Parenti, C. Palazzi, L. A. Amidei, F. B. Borrelli, S. C. Campanale, F. T. Tagliazucchi, G. S. Sedoni, D. L. Lucchesi, E. C. Carella, A. L. Luciani

#### Policlinico Modena, Bologna, Italy

**Introductions:** Previous studies tested the role of the internal jugular vein ultrasound exam (IJVUE) in the prediction of volume status but there are divergent conclusions on the its effectiveness in shock patients. For this reason we conducted a systematic review to check the level of reliability, validity and correlation with Central Venous Pressure (CVP) of the IJV ultrasound measures and the quality of reporting of literature on this topic.

**Methods:** This review was based on the PRISMA guideline. The systematic search of the literature published from 1941 through 30 June 2015 explored the PubMed, Cochrane Library, Web of Knowledge, and Scopus databases. Inclusion criteria were studies who investigated the reliability, the accuracy in predicting low CVP and the correlation with CVP of the IJV ultrasound measures in adult (>18 yrs) spontaneously breathing patients in shock. Three researchers selected studies using inclusion criteria and then assessed their quality using the STARD and QUADAS guidelines. The key words for literature search were: internal jugular veins, ultrasonography, volume status, central venous pressure, shock.

**Results:** We collected 219 studies: 20 excluded with reasons, 194 because duplicates. Five studies were included for the final analysis: 3 on reliability , 4 on correlation with CVP , 5 on validity. The IJV ultrasound measures (IJV ratio, anterior-posterior IJV diameter [AP-IJVD], IJV area) showed an inter-rater agreement range from moderate to excellent . The AP-IJVD had a significant high correlation with CVP; there were divergent conclusions on IJV area and poorly correlation for IJV ratio. The IJV ratio showed a moderate-good validity in predicting low CVP; the AP-IJV showed an high sensitivity and specificity for hyper and hypovolemia; an increase of IJV area effectively ruled out elevated CVP. Two studies respected more than 80 % of the STARD items and four more than 80 % of QUADAS items.

**Conclusions:** Because few reports have been published on this topic the conclusions of this review should be confirmed. Anyway the quality of reporting and methodology of the studies collected were good. The IJVUE seems to have a good reliability and accuracy in predicting low or high CVP . The AP-IJVD shows the best correlation with CVP.

## P165 Importance of recognizing dehydration in medical Intensive Care Unit

### M. Mackovic, N. Maric, M. Bakula

#### Clinical Hospital Sveti Duh, Zagreb, Croatia

**Introductions:** Dehydration is a relatively unrecognized disorder in medical ICU especially among older patients. Our aim was to determine the incidence of dehydration upon admission as well as the risk of developing dehydration during hospitalization and its effect on patient outcome. Moreover, several lab parameters were studied in order to assess the correlation to the diagnosis.

**Methods:** All patients entering ICU in a 6 month period in 2015 were enrolled and screened for dehydration on day 1 and day 3 using clinical and lab parameters (BUN, Cr, BUN/Cr ratio, Hct, serum Na, urine specific gravity). BUN/Cr >20 was used as a diagnostic criteria. Serum osmolality was not used due to unconsistent eligibility. In total of 332 patients, 38 were excluded due to GI haemorrhage. Finally, 294 patients were included, M/F 152/142, median age 74. Statistical methods used were McNemar test (differences in dehydration on day 1 and day 3), binary logistic analysis (association of lab parameters with dehydration) and multivariate adjusted binary logistic regression model (outcome).

**Results:** Of 294 patients 126 (42.9 %) were dehydrated upon admission. Dehydration clinically diagnosed at admission and defined by BUN/Cr >20 were significantly associated (McNemar test; χ ^2^ = 42.01; n = 246; p < 0.001; ϕ =0.24). Dehydration was correctly clinically recognised in 45/126 (35.7 %). Patients with BUN/Cr >20 had 3 times higher odds to be accurately clinically diagnosed. No correlation could be found between dehydration and values of Hct, serum Na and urine specific gravity. Statistically significant positive association was demonstrated for BUN (OR = 1.08; 95 % CI 1.04-1.11; p < 0.001) and Cr (OR = 1.00; 95 % CI 1.00-1.00; p = 0.048). On day 3 133/249 (54.1 %) patients were dehydrated which represents significant increase (McNemar test, χ ^2^ with continuity correction = 7.01; n = 246; p = 0.008; ϕ = 0.45). Overall, dehydration at any point of stay was associated with adverse outcome (death) (OR_adj_ = 2.45, 95%CI 1.38-4.34, p = 0.002).

**Conclusions:** Dehydration is a common finding in ICU and can be clinically easily overlooked due to insensitivity of clinicians for milder forms of dehydration which could explain a relatively high rate of misclassification. Clinical and lab parameters are insufficient for diagnosis which is consistent with previous observations. According to these data, hydration status changes during ICU stay and it’s fluctuations are associated with increased mortality suggesting that maintaining an adequate hydration status is of core interest for patient outcome.

## P166 Effect of volume for a fluid challenge in septic patients

### H. Aya, A. Rhodes, R. M. Grounds, N. Fletcher, M. Cecconi

#### St George’s Healthcare NHS Trust, London, UK

**Introductions:** An effective fluid challenge should increase the mean systemic filling pressure (Pmsf) in order to increase the venous return. The object of this study was to test the effect of several doses of intravenous fluids on the change of Pmsf in septic patients.

**Methods:** Patients were randomly allocated to receive 2, 3, 4 or 5 ml /kg (body weight) of intravenous crystalloid over five minutes. Pmsf was measured using the arterial pressure after stopping blood flow in the arm with a pneumatic tourniquet inflated for one minute. Mean arterial pressure (MAP), central venous pressure (CVP), cardiac output (CO) and stroke volume (SV) were recorded at baseline and immediately after the fluid infusion. CO was measured with LiDCOplus monitor. A positive response was defined as an increase of 10 % from baseline. From previous data, the least significant change (LSC) for Pmsf was 14 %. Medians were compared using the independent samples media test, and proportions were compared using an exact fisher test. Statistical significance was considered when p < 0.05

**Results:** 40 patients were included, 40 % of them were responders. The changes (Δ ) of Pmsf and CO in each group are described in Table 20. There is marginal evidence of Δ Pmsf is related to the dose of fluids, but the Δ CO and the proportion of responders are not affected by the dose of fluids infused

**Conclusions:** The dose of fluids does not affect the change in CO or the proportion of responders to a fluid challenge in septic patients.

**Table 20 (Abstract P166).** Changes of Pmsf-arm, cardiac output and percentage of responders in each group. Data presented as median (interquartile range)

	2 ml / Kg (n = 10)	3 ml / Kg (n = 12)	4 ml / Kg (n = 10)	5 ml / Kg (n = 8)	P
Δ .Pmsf-arm (%)	9.8 (6.9 - 13.9)	6.1 (3.9 - 9.5)	9.5 (-1.4 - 14.5)	18.8 (13.9 - 8.4)	0.06
Δ .CO (%)	2.9 (-9.0 - 16.9)	3.3 (-1.7- 10.4)	14.0 (2.1 - 3.7)	6.2 (1.7 - 21.4)	0.3
Responders (%)	40	25	60	37.5	0.4

## P167 Fluid bolus practices in a large Australian intensive care unit

### B. Avard^1^, P. Zhang^2^

#### ^1^The Canberra Hospital, Hughes, ACT, Australia; ^2^Australian National University Medical School, Canberra, Australia

**Introductions:** Fluid bolus administration is a ubiquitous therapy within Intensive Care, however recent evidence has suggested a more judicious approach may improve patient outcome. We aimed to describe the fluid bolus practices within our large Australian Intensive Care Unit to inform future focus of education of staff.

**Methods:** We conducted a retrospective audit of 234 critically ill patients who received fluid boluses and collected information on fluid type, volume, time administered, patient severity and prescriber details. Ethics approval was obtained. Data was analysed using IBM SPSS Stat 21.

**Results:** We found that a balanced salt solution was the most commonly used for fluid bolus therapy in our unit, independent of patient disease or severity. The majority of fluid was prescribed by junior doctors between 4 pm and 8 am, within 48 hours of ICU admission, though there was an additional peak of bolus administration at day 5 for those who remained in the unit at this time. Patients received on average 1.3 L of fluid in boluses.

**Conclusions:** Fluid bolus use in the clinical environment is highly variable and does not always align with theoretical principles.

**References**

Myburgh

**Fig. 34 (Abstract P167).**
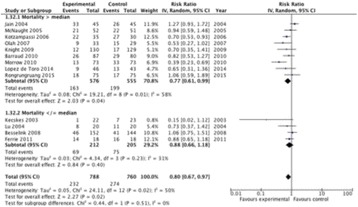
Fluid type

**Fig. 35 (Abstract P167).**
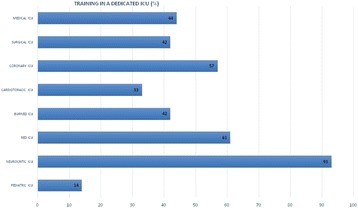
When bolused

## P168 Liberal late fluid management is associated with longer ventilation duration and worst outcome in severe trauma patients: a retrospective cohort of 294 patients

### M. Mezidi, J. Charbit, M. Ould-Chikh, P. Deras, C. Maury, O. Martinez, X. Capdevila

#### Lapeyronie University Hospital, Montpellier, France

**Introductions:** Liberal late fluid management (LFM) was associated with a higher morbi-mortality in different critically ill populations. In trauma, restrictive fluid strategy is largely recommended early resuscitation phase, however LFM has not been really studied. The main goal of present study was to assess in a severe trauma population the impact of liberal LFM on mechanical ventilation duration.

**Methods:** A retrospective analysis of all consecutive severe trauma patients admitted to our regional trauma center from 2010 to 2013 was performed. All patients with an ISS ≥16 and a length of ICU stay ≥7 days were included. Main admission characteristics, injuries, transfusions and management were collected, as well as fluid therapy and mechanical ventilation during ICU stay. Patients were classified in conservative LFM group if they had at least two consecutive days with a negative fluid balance between Day 3 and Day 7; the other patients belonged in liberal LFM group. These two groups were compared, especially in terms of fluid balance and mechanical ventilation requirements.

**Results:** During study period, 294 patients were included: median age 40 (IQR 25–58), ISS 29 (21–58), SAPS II 34(25–45), 76 % of male, 15 % of massive transfusion and 52 % of severe trauma head injury. After analysis of LFM,157 patients (53 %) were classified as conservative LFM and 137 (47 %) as liberal LFM. These two groups did not significantly differ in term of baseline characteristics, severe injuries, severity criteria or transfusion needs. Daily and cumulative fluid balances were also identical between these groups from Day 0 to Day 2. Conversely, liberal LFM was significantly associated with more ventilation days (11 vs 8.5 days; P = 0.02), less ventilator-free days at Day 30 (19 vs 21 days; P = 0.03), longer ICU stay (19 vs 16 days; P = 0.03) and hospital stay (30 vs 25 days; P = 0.04). Mortality rates were comparable between both groups (6 %). Multivariate analysis also highlighted that liberal LFM was independent risk factor for a reduced ventilator-free days at Day 30 (B = -2.1 [95%CI -4.1–-0.1], P = 0.042), as well as ISS score, severe trauma head injury and massive transfusion needs.

**Conclusions:** Liberal LFM was associated in our series with higher morbidity in the severe trauma patient; longer ventilation durations, and longer ICU and hospital stay. These results were observed despite a similar admission severity and early fluid management.

## P169 Association of fluids and outcomes in emergency department patients hospitalized with community-acquired pneumonia

### P. Hou^1^, W. Z. Linde-Zwirble^2^, I. D. Douglas^3^, N. S. Shapiro^4^

#### ^1^Brigham and Women’s Hospital, Boston, USA; ^2^Trexin Medical, Chicago, USA, ^3^University of Colorado and Denver Health, Denver, USA, ^4^Beth Isreal Deaconess, Boston, USA

**Introductions:** Emergency department (ED) patients admitted to the hospital with community-acquired pneumonia (CAP) may be at particular risk for volume overload related complications due to their infected lungs. This study used the Premier administrative database containing 17 % of US hospital discharges in 2013 to examine the association between a higher amount of fluids administered on day 1, hospital mortality and ventilator free days (VFD).

**Methods:** CAP was defined as a bacterial pneumonia diagnosis present on admission in ED patients receiving chest x-ray and parenteral antibiotics on Day 1. Day 1 total fluid volume was stratified into quartiles. The primary outcome was hospital mortality, and secondary outcome was VFDs over a 30 day period. To adjust for potential confounds, patients were stratified into 5 severity groups based on their modelled predicted mortality, and a propensity model for giving fluids was built using clinical characteristics, severity of illness scores, and ICD-9-CM codes present on admission for important acute and chronic conditions. Patients were then grouped by severity of illness quintiles of predicted risk of death for the CAP population. The association of fluid administration with outcome was assessed within each quintile of mortality risk using chi-square analysis.

**Results:** 192,806 adult ED patients with CAP were analyzed. Patients were 51.3 % female with a mean age of 69. Overall mortality rate was 7.4 % with an average of 24.6 VFDs. After propensity adjustment by severity of illness, mean amount of fluid administered on day 1 ranged from 842 ml to 2,189 ml. Within the fifth severity quintile we found a significant difference in the hospital (22.5 %) vs. predicted (18.3 %) mortality rate in the highest fluid quartile. Similarly, within the fifth severity quintile, we found a significant difference in the number of VFDs in the highest fluid quartile (16.1) compared to the rest of the quartiles (19.3-20.3).

**Conclusions:** For adult ED patients with CAP, we found significant associations between day 1 fluids and increased mortality and decreased VFDs in the highest fluid quartile and severity group, after adjusting for severity of illness and acuity level. This study may have identified the subset of ED patients who may benefit from a more restrictive fluid strategy when presenting to the hospital with CAP.

## P170 Association of positive fluid balance with poor outcome in medicosurgical ICU patients

### A. Ben Souissi, I. Mezghani, Y. Ben Aicha, S. Kamoun, B. Laribi, B. Jeribi, A. Riahi, M. S. Mebazaa

#### Mongi Slim University Hospital, La Marsa, Tunisia

**Introductions:** Administration of fluids is routine in management of ICU patients. However, controversies still exist with regard to the ideal type, dose and timing of iv fluid and a large amounts of evidence has shown that fluids can have deleterious effects on several organ functions, both from excessive amounts and from their non-physiological electrolyte composition (1). The aim of our study was to assess the negative impact of positive fluid balance on outcome in critically ill patients.

**Methods:** Retrospective study for one year (July 2014 to June 2015), including all patients admitted in medico-surgical ICU for more than 48 hours. Demographic data, SAPS II and APACHE II scores were registered at the first day. Length of stay, duration of mechanical ventilation, use of extra renal therapy, RIFLE score and mortality were noted. Daily and cumulative fluid balance was registered at 48 h, 7th, 14th, 21st days and at discharge.

**Results:** A total of 183 patients were included. Mortality rate was 41 %. Severity scores were 49 ± 17 and 19 ± 9 points respectively in SAPS II and APACHE II. Table 21 resume cumulative fluid balance. We noted a higher rate of septic shock, longer length of stay and mechanical ventilation duration in non-survivors group. The use of extra renal therapy was higher in non-survivors group (1 vs 11 p = 0.01). RIFLE score and diuretics use were also higher in non-survivors group (1 vs 2 p = 0.03 end 7 vs 16 p = 0.041). Non-survivors also showed higher accumulated positive fluid balance.

**Conclusions:** Fluid accumulation is very common throughout the course of a patient’s ICU stay with the evidence of the potentially deleterious effects of fluids and electrolytes on many organ systems. A more conservative approach regarding fluid management strategy in ICU patient seems to be less deleterious, but randomized controlled trials addressing this issue were needed.

**Reference**

1. Besen BA. World J Crit Care Med 2015;. 4:116–129.

**Table 21 (Abstract P170).** Comparative cumulated fluid balance in mL

	Survivors (n = 108)	Non-survivors (n = 75)	p
48 hours	1923 ± 1575	3038 ± 1235	0.02
Day 7	5663 ± 2401	6947 ± 3005	0.02
Day 14	4024 ± 1986	4673 ± 2265	0.03
Day 21	5042 ± 1967	7243 ± 3480	0.02
Total fluid balance	4982 ± 3087	9949 ± 3665	0.02

## P171 Impact of fluid balance to organ dysfunction in critically ill patients

### C. Pereira, R. Marinho, R. Antunes, A. Marinho

#### Centro Hospitalar do Porto, Porto, Portugal

**Introductions:** Fluids are a cornerstone of the management of critically ill patients with systemic inflammatory response syndrome who are at risk of multiple organ dysfunction syndrome. However, as with any therapy, fluids can be associated with harm, such as added or worsening organ dysfunctions. This study aimed to determine the impact of fluid balance (FB) in the respiratory, cardiovascular, renal systems.

**Methods:** Single-center prospective and observational study between June and December of 2014. All patients staying in ICU for more than 8 days were collected. Data on demographics, clinical characteristics, the total amount of fluids administered and excreted, blood creatinine and urea, the need and dose of vasopressors, PaO2, FiO2, MAP were collected regarding the first 8 days in the ICU. SPSS was used for statistical analysis.

**Results:** 122 patients were enrolled, where 64,3 % were men, with an average age of 58 ± 16, mortality rate was 22,3 %. The average daily FB in the eight days was 520.3 ± 1549.3 ml. There was a lower daily FB among survivors compared to nonsurvivors (433.8 ± 1483.7 ml vs 876.9 ± 1755.8 ml, p0.005). The cumulative fluid balance (cFB) difference between survivor / nonsurvivors was significant at the 8th day of ICU (p = 0.037). Regarding the cFB and the respiratory system, it was found that patients with a ratio PO2/FiO2 < =200 had a higher cFB between the 4th and 8th day. In the group of patients who required vasopressor support, cFB was higher in the 2nd and 3rd days (p = 0.04 and p = 0.036, respectively). The group of patients with a GFR <75 ml/min/1.73 m2 presented cFB slightly higher than the group with higher GFR (2nd and 4th days p = 0.049 e p = 0.041, respectively).

**Conclusions:** This study demonstrated that the cumulative fluid balance was higher in patients who had more severe dysfunctions of the systems reviewed, and these were associated with increased mortality rate. What remains to be established is whether fluid overload is only a biomarker that puts patients under an increased risk of death or an iatrogenic condition from critical care that should be considered in daily care and actively treated and avoided.

## P172 Volume bolus in ICU patients: do we need to balance our crystalloids?

### M. Crivits, M. Raes, J. Decruyenaere, E. Hoste

#### University Hospital, Ghent, Belgium

**Introductions:** Normal saline contains 154 mmol of Na and Cl, and may therefore induce hypernatremia, hyperchloremia and acidosis. Observational studies showed that resuscitation with normal saline when compared to balanced crystalloids, is associated with acute kidney injury and death. In these studies a large volume of saline was administered. A 1 liter (L) fluid bolus is often administered in ICU patients, e.g. for hypotension or oliguria. It remains uncertain if this limited volume is also associated with adverse effects. The aim of this study was to evaluate the effects of a fluid bolus of saline (S) and Plasma-Lyte (PL), a balanced crystalloid, on electrolytes, acid-base status, hemodynamics and urine output (UO).

**Methods:** We conducted a prospective randomized single blinded study comparing a 1 L fluid bolus of S and PL administered over 60 min, on a ICU cohort of 51 patients between 21th July 2015 and 3rd November 2015.

We included adult patients, who were prescribed a 1 L fluid bolus, and who had an arterial line and bladder catheter. Patients on dialysis were excluded. Data were recorded right before fluid infusion (T0), directly after (T1) and three hours (T2) after.

**Results:** The data of one patient was excluded from analysis because of a lack of informed consent. The remaining 50 patients were included, 23 (46 %) received S, 27 PL (54 %). There were no significant differences in baseline characteristics (T0) between both groups. S patients had higher Cl at T1 and T2 (110 vs. 109 mmol/L; p = 0.032 , resp. 110 vs. 108 ; p = 0.038), and higher Na at T1 (138 vs. 135 mmol/L; p = 0.034). Otherwise, no differences were noted. In addition, S patients had immediately after infusion an increase of Na (+0.4 vs. -0.8 mmol/L) , Cl (+3 vs. -1 mmol/L) (both p < 0.001), and a decrease of Strong Ion Difference (SIDa) (-2.3 vs. -0.1; p < 0.001), Strong Ion Gap (SIG) (-0.7 vs. +0.4; p = 0.015), and anion gap (AG) (-1.5 vs. -0.4; p = 0.018). This persisted at T2 for Cl (+2 vs. -1 mmol/L; p < 0.001) and SIDa (-2.1 vs. 0.4; p = 0.018). We found no difference in UO and blood pressure.

**Conclusions:** Compared to Plasma-Lyte, a 1 L fluid bolus of saline was at short term associated with a small increase of Na and Cl, and decrease in SID, SIG, and AG. These changes were statistically significant, but limited and were not associated with differences in hemodynamics or UO.

## P173 The use of 6 % HES solution do not reduce total fluid requirement in the therapy of patients with burn shock

### V. Bagin, V. Rudnov, A. Savitsky, M. Astafyeva, I. Korobko, V. Vein

#### City Clinical Hospital 40, Yekaterinburg, Russia

**Introductions:** Burn injury is included in the list of contraindication to HES use, but there is no convincing evidence base to such statement because of the small population of burn patients and absence of high-quality studies. Aim of this study was to access the efficacy and safety of HES in the therapy of patients with burn shock.

**Methods:** Design: retrospective cohort controlled study. Inclusion criteria: age > 18 years, TBSA burned 20 %-80%, absence of AKI at the admission. Patients were divided in two groups: HES group: 24 patients received 6%HES 130/0,4 in addition to base fluid therapy with Ringer solution and control group: 25 patients received only Ringer solution.

**Results:** Baseline characteristics were well balanced between groups. Hospital mortality was not significantly different: 45,8 % and 28,0 % in HES group and control group, respectively, p = 0,31. Rate of AKI was 29,2 % in HES group and 20,5 % in control group, p = 0,5201. 2 patients in HES group and 1 patient in control group required RRT. Independent risk factors for AKI were TBSA burned (OR 1,09 (1,01-1,19), p = 0,04), index Baux (OR 1,09 (1,01-1,19), p = 0,02) and need for catecholamines (OR 12,7 (1,2-144,9), p = 0,04). There wasn’t significant difference between groups in total volume of fluids administered in the first 3 days (Image 1).

**Conclusions:** This study did not show any negative influence of 6 % HES solution on mortality, incidence of AKI and need for RRT. Addition of HES solution in the early infusion therapy is not associated with the reduce of total fluid requirement.

**Fig. 36 (Abstract P173).**
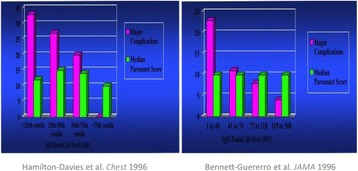
Box plots depicting daily cumulative fluid balance between groups

## P174 Electron microscopic assessment of acute kidney injury in septic sheep resuscitated with crystalloids or different colloids

### T. Kampmeier ^1^, P. Arnemann^1^, M. Hessler^1^, A. Wald^2^, K. Bockbreder^3^, A. Morelli^4^, H. Van Aken^1^, S. Rehberg^5^, C. Ertmer^1^

#### ^1^University Hospital Muenster, Muenster , Germany; ^2^Marienhospital Osnabrück, Osnabrück, Germany; ^3^Charité, University of Berlin, Berlin, Germany; ^4^Sapienza University of Rome, Rome, Italy; ^5^University Hospital of Greifswald, Greifswald, Germany

**Introductions:** The influence of intravenous fluids on kidney function and injury, especially in septic patients, is still being controversially discussed. We investigated the impact of different intravenous preparations on ultrastructural kidney damage (USKD) in a clinical relevant model of ovine peritoneal septic shock.

**Methods:** After approval by the local veterinary authorities, 20 healthy, female sheep underwent induction of peritoneal septic shock following an established model and consecutive randomization to one of the study groups: 1) control (no fluid therapy), 2) balanced crystalloids (Jonosteril©), 3) 5 % human albumin (Humanalbin©), 4) balanced 6 % HES 130/0.4 (Volulyte©) [each n = 5]. For dynamic detection of USKD, sequential sonography-guided kidney needle biopsies were performed at both healthy and septic baseline (BL1, BL2), as well as 6 h, 12 h and 24 h after the start of resuscitation. Analyses of the n = 1000 electronic microscopy pictures (10 pictures per time point (n = 5) and animal (n = 20)) were performed by two blinded, independent investigators following the “electron microscopic tubular injury“ (EMTI) score. The score contains the parameters 1) vacuolar alterations, 2) dissociation of epithelium and basal membrane, 3) epithelial cell injury, 4) intratubular precipitation and 5) intact microvilli cuticular layer. Each component of the score was quantified from 0 (no damage) to 3 (severe damage).

**Results:** All groups developed USKD over time with consecutive increases of EMTI scores. At BL2 + 6 h, the EMTI score of the HES-group was significantly lower as compared to crystalloid- and control-group (each p < 0.05) and at BL2 + 12 h the EMTI scores of albumin- and HES-groups were significantly lower as compared to the control group (each p < 0.01). 24 h after BL2, the EMTI score of the human albumin-group was significantly lower as compared to the control- and crystalloid-group (each p < 0.05). There were no significant differences between albumin- and HES-group at any time.

**Conclusions:** In the present ovine model of septic shock, all groups developed sepsis-associated USKD. Animals of the colloid-groups (albumin and 6 % HES 130/0.4) showed significantly decreased injury scores as compared to the animals of the control- and crystalloid-group. Ultrastructural kidney damage was most pronounced within crystalloid- and control-group in this study.

## P175 Alterations of conjunctival microcirculation in a sheep model of haemorrhagic shock and resuscitation with 0.9 % saline or balanced tetrastarch

### P. Arnemann^1^, M. Hessler^1^, T. Kampmeier^1^, S. Rehberg^2^, H. Van Aken^1^, C. Ince^3^, C. Ertmer^1^

#### ^1^University Hospital of Muenster, Muenster, Germany; ^2^University Hospital of Greifswald, Greifswald, Germany; ^3^Erasmus MC University Hospital Rotterdam, Rotterdam, Netherlands

**Introductions:** The present study was designed to compare the impact of resuscitation with either 0.9 % sodium chloride (0.9 % NaCl) or a balanced hydroxyethylstarch (HES) solution (6 % HES 130/0.4, Volulyte®, Fresenius Kabi, Bad Homburg, Germany) on the conjunctival microcirculation in a sheep model of haemorrhagic shock.

**Methods:** After approval by the local veterinary authorities, 10 female sheep were anaesthetized. After Baseline measurements 3x 10 ml blood per kg body weight were withdrawn to establish haemorrhagic Shock. Sheep were randomized to receive either 0,9 % NaCl (Saline group, n = 5) or Volulyte® (HES group, n = 5) for Resuscitation until Baseline values of mean arterial pressure (MAP) were reached again. At Baseline, Shock and after Resuscitation haemodynamic variables and 5 videos of conjunctival microcirculation were recorded (CytoCam®, Braedius, Huizen, The Netherlands). The perfused vessel density (PVD) and microvascular flow index (MFI) were calculated using a dedicated software (AVA Software v3.2, MicroVisionMedical, Amsterdam, The Netherlands). All values are presented as Median [IQR]. Mann-Whitney-U-Test was used for comparisons between groups. A p-value < 0.05 was considered statistically significant.

**Results:** MAP decreased in both groups from Baseline to Shock and increased after Resuscitation (Saline: from 89 mmHg [82;92] to 34 mmHg [32;46] to 72 mmHg [72;78]; HES: from 86 mmHg [71;97] to 43 mmHg [36;46] to 83 mmHg [67;92]; non-significant (ns) between groups). In the Saline group 4980 ml [3312;5700] and in the HES group 610 ml [489;615] of the respective fluid was needed (p = 0.009). In both groups, PVD decreased from Baseline to Shock (Saline: from 100 % to 83 % of Baseline value [49;86]; HES: from 100 % to 74 % [61;80]; ns between groups). While PVD increased after Resuscitation in the HES group to 125 % [120;147] it decreased in the Saline group to 64 % [62;79] (p = 0.009). MFI decreased from Baseline to Shock in both groups (Saline: from 3.1 [2.5;3.3] to 2.0 [1.6;2.3]; HES: from 2.9 [2.9;3.1] to 2.5 [2.3;2.7]; ns between groups). After Resuscitation MFI increased significantly higher in the HES (3.4 [3.2;3.5]) than in the Saline (2.7 [2.4;2.8]) group (p = 0.012).

**Conclusions:** Balanced 6 % HES 130/0.4 recruited more perfused vessels and improved microvascular blood flow in the conjunctival microcirculation better than 0.9 % saline, while sparing considerable amounts of intravenous fluids.

## P176 A single centre nested pilot study investigating the effect of using 0.9 % saline or Plasma-Lyte 148 ® as crystalloid fluid therapy on gastrointestinal feeding intolerance in mechanically ventilated patients receiving nasogastric enteral nutrition

### S. Reddy^1^, M. Bailey^2^, R. Beasley^1^, R. Bellomo^3^, D. Mackle^1^, A. Psirides^4^, P. Young^1^

#### ^1^Medical Research Institute of New Zealand, Wellington, New Zealand; ^2^Monash University, Melbourne , Australia; ^3^Austin Hospital, Melbourne, Australia; ^4^Wellington Regional Hospital, Wellington, New Zealand

**Introductions:** Early clinical trials and experimental data have reported that the use of unbuffered fluids may adversely influence gastrointestinal (GI) function when compared to buffered fluids. GI dysfunction and nutritional feeding intolerance is common amongst critical unwell patients. No previous studies have assessed the GI effects of different crystalloid fluids in critically ill patients.

Objective: To evaluate the effect of Plasma-Lyte 148 (PL-148) compared with 0.9 % saline (saline) on gastrointestinal (GI) feeding intolerance in mechanically ventilated ICU patients receiving nasogastric (NG) feeding.

**Methods:** We conducted a single centre pilot study nested within a multicentre, double-blind, cluster-randomised, double crossover trial. All adult patients who required crystalloid fluid as part of the SPLIT trial [1], expected to require mechanical ventilation for >48 hours and receiving enteral nutrition exclusively by a NG tube were eligible. The study ICU used either blinded saline or PL-148 for four alternating seven week blocks. The primary outcome was the proportion of patients with GI feeding intolerance, defined as high gastric residual volumes (GRV), diarrhoea or vomiting while receiving NG feeding in the ICU. The proportion of patients with each of high GRV, diarrhoea, and vomiting were secondary outcomes.

**Results:** 69 patients were enrolled, 35 assigned to PL-148 and 34 to saline. In the PL-148 group, 21 of 35 patients (60.0 %) developed GI feeding intolerance as compared to 22 of 34 patients (64.7 %) in the saline group (OR,0.82; 95 % CI, 0.31 to 2.17; P = 0.69). A high GRV was observed in 4 of 35 patients (11.4 %) and 11 of 34 patients (32.4 %) in the PL-148 and saline groups respectively (OR,0.27; 95 % CI, 0.08 to 0.96; P = 0.04).

**Conclusions:** Among mechanical ventilated patients receiving NG feeding, the use of PL-148 compared to saline did not reduce the proportion of patients developing GI feeding intolerance but was associated with a decreased incidence of high GRV.

**Reference**

[1] Young P, Bailey M, Beasley R, et al. Effect of a Buffered Crystalloid Solution vs Saline on Acute Kidney Injury Among Patients in the Intensive Care Unit: The SPLIT Randomized Clinical Trial.JAMA 2015:1-10

## P177 A single centre nested pilot study investigating the effect on post-operative bleeding of using 0.9 % saline or Plasma-Lyte® 148 as crystalloid fluid therapy in adults in ICU after heart surgery

### S. Reddy^1^, M. Bailey^2^, R. Beasley^1^, R. Bellomo^3^, D. Mackle^1^, P. Young^1^

#### ^1^Medical Research Institute of New Zealand, Wellington, New Zealand; ^2^Australian and New Zealand Intensive Care Research Centre, Melbourne , Australia; ^3^Austin Hospital, Melbourne, Australia

**Introductions:** Using buffered crystalloid fluids, such as Plasma-Lyte 148 (PL-148), may reduce post-operative blood loss and transfusion requirements compared to 0.9 % saline (saline) [2,3]. However, no studies have assessed the effect on postoperative blood loss of using different crystalloid fluids after heart surgery.

Objective: To evaluate the effect of PL-148 compared with saline on bleeding following heart surgery.

**Methods:** We conducted a single centre pilot study nested within a multicentre, double-blind, cluster-randomised, double crossover trial. All adult heart surgery patients who required crystalloid fluid therapy in ICU as part of the SPLIT trial [1] were eligible. The primary outcome was chest drain output from time of arrival to ICU until 12 hours post operatively. The key secondary outcomes were total volume of fluid in chest drains from arrival to ICU until 24 hours if still in ICU and proportions of patients requiring any of the following blood products (packed red blood cells, platelets, fresh frozen plasma or cryoprecipitate).

**Results:** 251 patients were enrolled, with 131 assigned to PL-148 and 120 to saline. There was no difference in postoperative chest drain output at 12 hours (ratio of geometric means, 1.06; 95 % CI, 0.93 to 1.20; P-value = 0.42) or at 24 hours. In the PL-148 group, 28 of 131 patients (30.5 %) required blood products in ICU compared with 22 of 120 patients (18.3 %) in the saline group (OR, 1.96; 95 % CI, 1.08 to 3.54; P = 0.03).

**Conclusions:** Among patients admitted to ICU following heart surgery, the use of PL-148 compared to saline did not reduce the amount of post-operative blood loss but was associated with increased use of bloods products.

**References**

[1] Young P, Bailey M, Beasley R, et al. Effect of a Buffered Crystalloid Solution vs Saline on Acute Kidney Injury Among Patients in the Intensive Care Unit: The SPLIT Randomized Clinical Trial. JAMA; 2015:1-10.

[2] Orbegozo Cortes D, Rayo Bonor A, Vincent JL: Isotonic crystalloid solutions: a structured review of the literature. Br J Anaesth 2014;112:968-981.

[3] Shaw AD, Bagshaw SM, Goldstein SL, et al: Major complications, mortality, and resource utilization after open abdominal surgery: 0.9 % saline compared to Plasma-Lyte. Ann Surg 2012;255:821-829.

## P178 Extreme hypernatremia and sepsis in a patient with Huntington’s dementia: a conundrum in fluid management

### H. Venkatesh^1^, S. Ramachandran^2^, A. Basu^3^, H. Nair^3^

#### ^1^Princess of Wales Hospital, Bridgend, UK; ^2^Imperial College London, London, UK; ^3^Glan Clwyd Hospital, Bodelwyddan, UK

**Introductions:** Approximately 1 to 2 % of patients attending the Emergency Department have hypernatremia(1). Hypernatremia over 160 mmol/L is considered to be very severe and as such needs to be treated with caution(2). Extreme cases of hypernatraemia are rare (where sodium levels are exceptionally high), especially in adults and the most common pathophysiology in such cases would be related to severe dehydration. We see this more in the elderly population, children, the institutionalised and patients with dementia.

**Methods:** We report a unique case of extreme hypernatremia of 196 mmol/L and severe sepsis in a young 39-year-old adult with Huntington’s dementia, which presented a challenge in fluid management.

**Results:** The hypernatraemia was thought to be caused by chronic severe dehydration from poor intake and the sepsis was thought to have started as an inadequately treated urinary tract infection. The patient was initially treated aggressively with hypotonic saline and intravenous antibiotics, but was subsequently managed using a slower correction rate after identifying the chronic nature of the natremia. To our knowledge, this is the first reported case of extreme hypernatremia and severe sepsis manifesting concomitantly in such a young patient.

**Conclusions:** We highlight the difficulties of balancing the risks and benefits of rapid versus slow fluid resuscitation in such complex clinical situations. We highlight the difficulty of balancing the risks and benefits of rapid fluid resuscitation, necessitated by severe sepsis and acute kidney injury, against the complexities of fluid resuscitation in correcting such an extreme hypernatremia.

We advocate early identification of chronic hypernatremia and effective administration of intravenous fluids in the Emergency Department that is decided on a case-by-case basis. In cases of severe and extreme hypernatraemia in particular, accurate calculation of free water deficit is essential to manage the patient effectively. In line with this, we must ensure that such patients are always weighed on admission.

**References**

(1) Arampatzis S, Exadaktylos A, Buhl D, et al. Dysnatraemias in the emergency room: Undetected, untreated, unknown? Wien Klin Wochenschr. 2012;124(5-6):181-183.

(2) Aiyagari V, Deibert E, Diringer MN. Hypernatremia in the neurologic intensive care unit: How high is too high? J Crit Care. 2006;21(2):163-172.

## P179 Diagnosis and management of severe hypernatraemia in the critical care setting

### S. Egan, J. Bates

#### University Hospital Galway, Galway, Ireland

**Introductions:** The development of hypernatraemia leads to hypertonic stress within the cells. Previous studies have shown that the presence of hypernatraemia on admission to Intensive Care is an independent risk factor for poor prognosis. While rapid correction of hypernatraemia can have devastating neurological consequences; a correction speed too slow is associated with an increased risk of death. The primary objective was to assess the frequency of severe hypernatraemia in patients admitted to our Critical Care Unit, located in a tertiary referral centre and university hospital. Time to sodium normalisation, presence/absence of neurological symptoms and patient outcome was also evaluated. Severe hypernatraemia was classified as plasma sodium ≥160 mmol per litre.

**Methods:** We retrospectively searched the Critical Care database for laboratory sodium values ≥160 mmol/L occurring between November 2013 and November 2015. Where appropriate, sodium was corrected for glucose. This identified all patients in whom severe hypernatremia was present on admission and those in whom it developed during their Critical Care stay.

**Results:** We identified 14 patients with severe hypernatraemia over the past two years in our Critical Care Unit. The plasma sodium range was 160 to 196 mmol/L. All values > 170 mmol/L occurred in patients with hyperglycaemic hyperosmolar syndrome (HHS). The underlying aetiology was as follows: 4 (28.6 %) patients HHS, 1 (7.1 %) patient diabetic ketoacidosis, 1 (7.1 %) patient lithium-induced diabetes insipidus and 8 (57.1 %) patients with dehydration secondary to unreplaced water loses. In no case was the rate of correction greater than 10 mmol/L per day. The average length of time for correction to normal sodium level of 145 mmol/L was 6 days. Three patients (21.4 %) died in Critical Care. Two patients (14.3 %) died following discharge to the ward. The remaining 9 patients (64.3 %) were discharged home to their primary residence. All patients had neurological symptoms on presentation, primarily confusion. One patient presented with seizures. All patients were treated with 5 % dextrose. One patient developed hypernatraemia in Critical Care. Moderately elevated sodium levels were untreated in this patient prior to the diagnosis of severe hypernatraemia. The remaining patients all presented with hypernatraemia.

**Conclusions:** Severe hypernatraemia is associated with a high mortality rate of 35.7 %. Physicians should be aware of the excessive risk associated with this condition and diagnosis should be followed by prompt rehydration and intensive clinical and biochemical monitoring.

## P180 Correlation between arterial blood gas and electrolyte disturbances during hospitalization and outcome in critically ill patients

### S. Oliveira, N. R. Rangel Neto, F. Q. Reis

#### Albert Schweitzer State Hospital, Rio de Janeiro, Brazil

**Introductions:** Critical illness is a life-threatening multisystem process that can result in significant morbidity or mortality. In most patients, critical illness is preceded by a period of physiological deterioration; but evidence suggests that the early signs of this are frequently missed.

**Methods:** In this cross-sectional study, the medical data of all critically ill patients admitted to the Albert Schweitzer Hospital were reviewed between January 2013 and September 2015. Data including age, gender, SAPS III score upon admission, arrival and 24 hour ABG and electrolytes values, as well as length of stay and in-hospital mortality rate were collected. A logistic regression model was used to determine the association between acid-base and electrolyte disturbances and in-hospital mortality after adjustment for potential confounding factors.

**Results:** Of the 4732 patients, 57 % were male; mean age of 58 years old, mean SAPS III score of 58 points and 33,8 % of them died in the hospital. Univariate analysis showed a significantly higher risk of mortality in patients who developed mixed metabolic acidosis plus respiratory acidosis on their admission day (OR = 3.45, p-value < 0.001). Multiple logistic regression analysis demonstrated that mixed metabolic acidosis plus respiratory acidosis (OR = 3.91, 95 % CI = 1.37-10.21, p-value = 0.003) and hyponatremia (OR = 0.58, 95 % CI = 0.31-0.65, p-value 0.01), patients with hyperkalemia had higher mortality (serum potassium over 5,1 mEq/L 43,4 % died and serum potassium over 6,0 mEq/L 76,5 % died; p 0.01 and p 0,002 respectively), were three significant predictors of mortality, regardless of other confounding variables.

**Conclusions:** The results show that in critically ill patients initial mixed metabolic acidosis plus respiratory acidosis, hyponatremia and hyperkalemia are significant predictors of mortality, but other factors after adjustment for potential confounding factors had no prognostic effect.

**References**

Lactate, pH, and blood gas analysis in critically ill patients. Waldau T ET AL Acta Anaesthesiol Scand Suppl. 1995 ;107:267-71.

Clinical significance of acid-base balance in an emergency setting in patients with acute heart failure. Shirakabe A et al.J Cardiol. 2012 Oct; 60(4):288-94. Epub 2012 Jul 20.

## P181 Missing the “I” in MUDPILES – a rare cause of high anion gap metabolic acidosis (HAGMA)

### C. P. Lee^1^, X. L. Lin^2^, C. Choong^1^, K. M. Eu^1^, W. Y. Sim^1^, K. S. Tee^1^, J. Pau^1^, J. Abisheganaden^1^

#### ^1^Tan Tock Seng Hospital, Singapore, Singapore; ^2^National Neuroscience Institute, Singapore, Singapore

**Introductions:** HAGMA is a commonly encountered acid-base disorder in clinic practice. Multiple mnemonics were proposed to cover the common underlying causes – “GOLDMARK(1)”, “KUSMALE”, “KARMEL” and, of course, “MUDPILES”. The “I” in “MUDPILES” represents some rare causes of HAGMA including “I”ron, “I”soniazid and “I”nborn errors of metabolism. We present a rare case of HAGMA secondary to isoniazid (INH) overdose.

**Methods:** A 41 year old male was intubated for status epilepticus. He had a medical history of pulmonary tuberculosis (pTB) and had been treated with a course of rifampicin, INH and ethambutol since 6 months ago.

**Results:** Arterial blood gas in the Emergency Department showed severe metabolic and respiratory acidosis; pH 6.76, pCO2 80, pO2 62, HCO3 11, BE -24. The initial anion gap was 26. Physical examination was unremarkable except for fever and hypotension. Neurological examination did not reveal any lateralising signs. CT scan of the brain was normal. Mild renal impairment, and lactate acidosis resolved rapidly with initial fluid resuscitation. Serum and urine toxicology for commonly encountered drugs were also negative. Patient regained orientation on day 3 of intubation and was promptly extubated. Further history revealed that he had consumed a bottle of isoniazid tablets after an altercation with his wife the same night.

**Conclusions:** Tuberculosis is endemic is South East Asia. INH is one of the four drugs used in the treatment of this disease. Side effects of isoniazid range from mild hepatotoxicity to the potentially fatal INH hepatitis. HAGMA refractory to conventional therapy is one of the hallmarks of INH toxicity. Though it is one of the rare causes of HAGMA, prompt diagnosis and treatment with high dose pyridoxine (2) reduces mortality and morbidity.

**References**

1. Mehta AN, Emmett JB, Emmett M. GOLD MARK: an anion gap mnemonic for the 21st century. Lancet. 2008;372(9642):892.

2. Orlowski JP, Paganini EP, Pippenger CE. Treatment of a potentially lethal dose isoniazid ingestion. Ann Emerg Med. 1988;17(1):73-6.

## P182 Plasma NGAL and urinary output: potential parameters for early initiation of renal replacement therapy

### K. Maas, H. De Geus

#### Erasmus Medical Centre, Rotterdam, Netherlands

**Introductions:** Acute kidney injury (AKI) is independently associated with increased mortality in the critically ill, especially when renal replacement therapy (RRT) is required. “Early initiation” of RRT and its possible beneficial effect on mortality, has been the focus of many research protocols. However, published papers show no uniformity in the definition of early initiation. The main problem to determine this best moment of initiation is the lack of a distinctive parameter that can predict RRT requirement. The question rises if differences between patients with any stage of AKI and patients with RRT-requiring AKIN stage III can be identified looking at the “classical parameters” (serum creatinine (SCr), urea, potassium, bicarbonate, pH, cumulative fluid balance), plasma Neutrophil Gelatinase-Associated Lipocalin (pNGAL) and urinary output (UO) at the time of first rise in SCr (first AKI).

**Methods:** This is a retrospective subset-analysis performed on the NGAL-study database [1] in adult critically ill patients with developing AKI. Data were collected and analyzed at the time of first AKI and at the highest level of serum creatinine (peak AKI). Mann-Whitney-U test was used to detect differences between both groups.

**Fig. 37 (Abstract P182).**
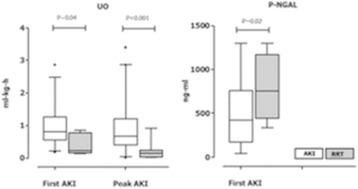
Levels of urinary output and pNGAL in patients with any AKI compared with RRT

**Results:** A total of 59 patients developed any stage of AKI during the first 7 days after ICU admission, 15 patients required RRT eventually. At the time of first AKI, pNGAL and UO were the only parameters that differed significantly (P = 0.02 and P = 0.04 respectively) between the two groups. The “classical parameters” showed a significant difference between groups later on at the time of peak AKI (=time of RRT initiation) (Fig. 37).

**Conclusions:** Critically ill patients with RRT requiring AKI have a significant lower urinary production and significant higher pNGAL concentration compared to patients with non-RRT requiring AKI at the time of first rise in SCr. The “classical parameters” fail to make this distinction at this very early stage of AKI. Therefore, plasma NGAL and UO might have the potential (perhaps in combination with the Furosemide stress test) to become valuable parameters in future prospective protocols that intend to study the value of early RRT initiation on hard outcome measures in critically ill patients.

**Reference**

1. De Geus HRH et al.: AJRCCM; 2011 183: 907-914

## P183 Renal replacement therapy for critically ill patients: an intermittent continuity

### E. Lafuente^1^, R. Marinho^2^, J. Moura^3^, R. Antunes^2^, A. Marinho^2^

#### ^1^Centro Hospitalar Tamega e Sousa, Penafiel, Portugal; ^2^Centro Hospitalar do Porto, Porto, Portugal; ^3^Unidade Local de Saude do Alto Minho, Viana do Castelo, Portugal

**Introductions:** The choice for the right renal replacement therapy (RRT) for severe acute kidney injury in critically ill patients has been investigated many times in the last two decades.

Although some questions have been answered, in current practice many different approaches are still used in the ICU. The authors review the treatment of Acute Kidney Injury (AKI) in critically ill patients, indications for SLED and continuous renal replacement therapy (CRRT) in critically ill patients in four Portuguese ICUs.

**Methods:** Prospective observational study conducted in 4 ICUs in 3 Portuguese hospitals for seven months. All patients with AKI who needed a renal replacement therapy were enrolled. Data on demographics and clinical characteristics of patients were collected at baseline.

**Results:** 127 patients were enrolled, with an average age of 61,3 ± 15,26, and mortality rate was 46 %. 42 patients performed SLED (33,07 %), and 85 % performed CRRT (63,97 %). The most common indications for CRRT was fluid overload (47,76 %). Most of the patients start the both procedures in the first two days in ICU. Most of the patients (59 Ð 60 %) were in organ failure, using the RIFLE criteria when they started RRT. Patients in CRRT were more hemodynamically unstable (norepinephrine in CRRT Ð 1,99 ± 5,36 and in SLED - 1,49 ± 4,56). Mortality rate Ð 52,94 % in CRRT and 30,95 in SLED.

**Conclusions:** CRRT seems to be the ideal mode of renal replacement therapy for hemodynamically unstable with fluid overloaded. However, in expert hands, the two treatments provide similar outcomes and can be complementary.

## P184 A survey of practices related to renal replacement therapy in critically ill patients in the north of England.

### T. E. Doris, D. Monkhouse, T. Shipley, S. Kardasz, I. Gonzalez

#### South Tees NHS Trust, Middlesbrough, UK

**Introductions:** The North of England Critical Care Network does not currently have an up- to- date guideline regarding the use of renal replacement therapy (RRT) within intensive care.

We wished to evaluate current practice in the region and consider the need for regional guidance on the management of acute kidney injury in our units.

**Methods:** An electronic survey was sent to all the consultants in intensive care within the region. Responses were anonymously collecting using internet-based survey software.

Questions were based upon the currently available guidance and evidence.

**Results:** Invitations were sent to 139 consultants who have some intensive care commitment. There were 55 responses giving a response rate of 39.6 %. Unit size varied greatly, from under 10 beds, to over 20. The majority (43.6 %) had between 10 and 20 beds. 46.3 % of respondents had less than 10 patients receiving RRT per month, 40.7 % had between 10 and 25 patients per month and 13 % had more than 25 patients per month. 43 % gave positive comments regarding the support they receive from renal services. Some areas where interactions between renal physicians and critical care could be improved were identified. Only 67 % of respondents were aware of an up to date guideline for the use of RRT in their unit.

1.9 % of respondents preferentially use the subclavian vein for siting renal access lines. 9.3 % preferentially used the femoral veins. 31 % never have visits from a nephrologist.

**Conclusions:** There is a wide variety in practice, with some areas where practices could be improved , notably awareness of guidelines available, choice of renal access line site, and working relationships between renal and critical care.

As part of a quality improvement programme, we plan to create a regional best practice recommendations document that is relevant to the systems in which we work and specific to critical care. By doing this, we hope to improve outcomes for those receiving renal replacement therapy in our units.

## P185 High initiation creatinine associated with lower 28-day mortality in critically ill patients necessitating continuous renal replacement therapy

### S. Stads^1^, A. J. Groeneveld^2^

#### ^1^Ikazia Hospital, Rotterdam, Netherlands, ^2^Erasmus MC, Rotterdam, Netherlands

**Introductions:** Acute kidney injury necessitating continuous renal replacement therapy (CRRT) in critically ill patients is associated with high mortality. Timing of CRRT remains a matter of debate. We investigated the effect of timing of CRRT on 28-day mortality.

**Methods:** A post-hoc analysis is performed on the multicenter data from the earlier published CASH study. In critically ill patients receiving CRRT, between 2005 and 2011, the effect of variables at initiation of CRRT on 28-day mortality were evaluated. Univariate and multivariate cox regression analysis were performed to determine renal and patient-specific variables associated with 28-day mortality.

**Results:** Of the 139 patients evaluated for inclusion, 13 patients were excluded because of a history of chronic kidney disease, 5 patients were excluded because no creatinine at CRRT initiation was available, and 5 patients were excluded because an admission creatinine below 50 μmol/L. In the 116 included patients, 28-day mortality was 37 (32 %). In univariate proportional hazards cox regression analysis, ICU admission after cardiopulmonary resuscitation ( HR 4.403, p = 0.043), SOFA score ( HR 1.117, p = 0.024) and creatinine at CRRT initiation (HR 0.997, p = 0.020) were associated with 28-day mortality. Multivariate analysis demonstrated that age (HR 1.092, p = 0.050), admission weight ( HR 1.036, p = 0.016) ICU admission because of respiratory failure (HR 9.275, p = 0.29) and creatinine at CRRT initiation (HR 0.990, p = 0.022) were associated with 28-day mortality. After correction for disease severity scores, such as APACHE 2 score and SOFA score, and markers for fluid overload, such as hematocrit and cumulative fluid balance, creatinine at initiation remained associated with lower 28-mortality. Even after correction for admission creatinine, only creatinine at initiation remained associated with 28-day mortality. In ROC curve analysis a CRRT initiation creatinin of 318 μmol/L was the best predictor for 28-day mortality.

**Conclusions:** In this multicenter cohort, creatinine at CRRT initiation is an independent predictor of 28-day mortality, even after correction for admission creatinine, disease severity and hemodilution. Therefore these data argue in favour of a time-dependent effect of CRRT on 28-day mortality. These data suggest that late CRRT initiation may have better outcome then earlier CRRT initiation. However further research is needed to confirm this hypothesis and reveal underlying mechanisms.

## P186 The impact of Karnofsky performance scale on outcomes in acute kidney injury patients receiving renal replacement therapy on the intensive care unit

### I. Elsayed^1^, N. Ward^1^, A. Tridente^2^, A. Raithatha^1^

#### ^1^Sheffiled Teaching Hospitals, Sheffield, UK; ^2^Whiston Hospital, St Helens & Knowsley, UK

**Introductions:** Karnofsky performance scale (KPS) [1] is being widely to describe patients’ functional status. Correlation between poor functional levels and adverse outcomes has been found. KPS is used in decision making surrounding starting aggressive therapy. We evaluated the impact of KPS on long term renal & patient survival in AKI patients, receiving renal replacement therapy (RRT) in intensive care (ICU).

**Methods:** We prospectively enrolled AKI ICU patients between October 2011 & October 2013. Demographics, APACHE II and SOFA scores, modality & dose of RRT, ICU length of stay (LOS) and KPS were collected. Data on renal and patient survival at ICU discharge, at 28 and 90 days and also 12 months were collected. Analysis used was multivariate logistic regression (Stata 14.1)

**Results:** 155 patients were recruited. 97 (62.6 %) were males. Median age was 62 (IQR 47-72). Median APACHE II score was 23 (IQR 18-27). Median SOFA score on admission was 10 (IQR 7-13). 101 (66 %) were mechanically ventilated, median LOS was 6.8 (IQR 2.7-14.9) days, median KPS was 80 (IQR 70-90), 79 (51 %) patients were dependent on RRT at ICU discharge, with 61 (39.4 %), 56 (36.1 %) and 56 (36.1 %) at 28 & 90 days and 12 months respectively. 105 (66.7 %) patients survived to ICU discharge, with 94 (61.4 %), 88 (57.5 %) and 80 (52.3 %) patients being alive at 28 & 90 days and 12 months respectively.

Adjusting for age and sex, APACHE II was the only factor associated with patient need for RRT at ICU discharge, 28 days, 90 days and 12 months (OR 1.09, 95 % CI: 1.03-1.15, p = 0.003; OR 1.1, 95%CI 1.04-1.16, p = 0.001; OR 1.1, 95%CI 1.04-1.17, p = 0.001 and OR 1.1, 95%CI 1.04-1.17, p = 0.001, respectively). After adjusting for age and sex, admission SOFA (OR 0.88, 95%CI 0.8-0.97, p = 0.008), APACHE II (OR 0.89, 95%CI 0.84-0.95, <0.001) and KPS (OR 1.03, 95%CI 1-1.06, p = 0.049) were associated with ICU survival. However at multivariate regression analysis, only APACHE II was independently associated with ICU survival (OR 0.91, 95%CI 0.85-0.97, p = 0.003).

**Conclusions:** Disease severity remains the main determinant of outcome in AKI patients receiving RRT on ICU. Despite the apparent relevance of KPS to outcomes on critical care, we could not in this instance show that KPS independently affected renal or patient survival. More studies on larger numbers of patients are required to clarify this issue.

**Reference**

[1] Oken M et al. Am J Clin Oncol. 1982;6:649-655

## P187 Severe hypophosphatemia during citrate-anticoagulated CRRT

### A. Steuber^1^, C. Pelletier^1^, S. Schroeder^1^, E. Michael^1^, T. Slowinski^2^, D. Kindgen-Milles ^1^

#### ^1^University Hospital Duesseldorf, Düsseldorf, Germany; ^2^Charite University Hospital, Berlin, Germany

**Introductions:** CRRT with regional citrate anticoagulation (RCA) provides long filter running times and effective dialysis doses. Thus, the risk of hypophosphatemia (hypoPO4) is high and PO4 substitution is required. We investigated whether separate infusion of PO4 or a PO4-containing dialysis fluid is more effective to avoid severe hypoPO4.

**Methods:** Retrospective-prospective observational study in a university hospital. CRRT with RCA-CVVHD (30 ml/kg/h). PO4 substitution via a separate infusion (PO4-I; retrospective data); use of a PO4-containing dialysate (CiCa dialysate K4, Fres. Medical Care)(PO4-D, prospective data). Primary outcome: incidence of severe hypoPO4. Secondary outcome: maintenance of normal PO4-levels, duration of mechan. ventilation and length stay in the ICU (with approval of local ethics committee, NCT01946113).

**Results:** We recruited 100 pts. for each group. Epidemiological data were not different in PO-I vs PO-D (mean age 69 ± 11 vs 68 ± 12 y; BW 81 ± 19 vs 86 ± 22 kg; male 66 vs 71 %). Major cause of AKI was sepsis (41 %). Incidence of severe hypophoph: PO-I 66 vs PO-D 12 pts. (p < 0.05). Duration of mechan. vent.:PO-I 592 ± 508 vs PO-D 428 ± 466 h(n.s.), ICU days PO-I 33 ± 30 d vs PO-D 26 ± 25 d (n.s.).

**Conclusions:** Despite rigorous efforts to maintain normal PO4-levels via PO4-infusion, severe hypophosphatemia occured in 66 % of all pts. In contrast, a PO4-containing dialysis fluid ensured normal PO4-levels in most pts. and significantly reduced the risk of severe hypoPO4. There was a trend towards reduced length of mech. ventilat. and ICU stay. Thus, a PO4-containg dialysate can be recommended to increase safety of CRRT in ICU patients.

**Fig. 38 (Abstract P187).**
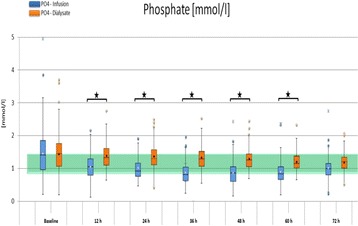
Hypophosphatemia during CRRT with PO4-supplementation via infusion or dialysate

## P188 Citrate regional anticoagulation for post dilution continuous renal replacement therapy

### S. Ghabina

#### Royal London Hospital, London, UK

**Introductions:** S Schwarze, A Beane, S Ramsay, C J Kirwan, J R Prowle

Regional citrate administration (RCA) is an effective anticoagulation technique for continuous renal replacement therapy (CRRT). Commonly, RCA protocols incorporate citrate within the replacement fluid adding calcium post filter. Following modification of the Aquarius haemofilter (Nikkiso), we designed and implemented a protocol for RCA with standalone citrate administration pre filter and post dilution CVVHF using calcium containing replacement fluid.

**Methods:** A prospective audit of the first 10 critically ill patients receiving RCA with post dilution CVVHF. Inclusion criteria: Adult critically ill patients requiring CRRT but contraindicated for heparin. Exclusion criteria:liver disease, liver injury, noradrenaline >0.5mcg/kg/min, ideal body weight >90 kg, receiving >6 u/hr of insulin, sodium <120 or >160 mmol/l, pH >7.5 or bicarbonate > 40 mmol/l. Primary outcome measure-filter time Secondary outcome measures:calcium replacement; Post filter ionised calcium (iCa); acid base status and serum sodium levels. A dose choice of 25 or 35 mls/kg/min with parallel changes to the filtration fraction from 20 to 25 % as required to manage metabolic alkalosis. If metabolic alkalosis persisted, RCA was stopped. Acid Citrate Dextrose Forumula-A (ACD-A) (113 mmol/L of citrate) is administered at a weight related rate aiming for a citrate concentration of 2.5-3 %. Replacement fluid is Accusol 35 (1.75 mmol/l calcium). A post filter solution of 10 mmol/l calcium chloride is administered according to post filter ionized calcium (iCa).Bicarbonate, iCa and sodium measured at 3 to 6 hourly intervals during therapy.

**Results:** 10 patients (6 male) aged 31-82, received RCA across 49 filters. Median filter lifespan 21 hours (1-72). Post filter iCa (mmol/L) 0.38 (0.28-0.64); Filter time (hr) 21 (1-72); Ca:iCa 1.83 (1.01-2.59) pH 7.37 - 7.45; Bicarbonate mmol/L 28.3 - 33.2; Sodium (mmol/L) 138 – 141. 8 of 49 filters (16 %) in 6 patients required calcium replacement (median 13.5 mmols (0.25-32.75)). Filter cessation: Planned 4;Set life expired (72 hours) 2;Clotted circuit 19; Pressure 12; Balance 2; Access 3; liver failure 1; Not documented 6.

**Conclusions:** CRRT and RCA with standalone citrate administration and post dilution is an effective and safe alternative where heparin anticoagulation is contraindicated. Standard calcium containing replacement fluid dramatically reduces the need for additional post filter calcium infusions which adds to potential cost saving.

## P189 Citrate 18 mmol/l improves anticoagulation during RRT with adsorbing filters

### F. Turani , A. Belli, S. Busatti, G. Barettin, F. Candidi, F. Gargano, R. Barchetta, M. Falco

#### Aurelia and European Hospital, Rome ,Italy

**Introductions:** Adsorbing filters with an anti-inflammatory action - Coupled plasma filtration and adsorption -(CPFA) and Cytosorb may be clinically useful during sepsis.

The aim of this study in a cohort of septic patients during RRT with CPFA or Cytosorb is to evaluate with tromboelastography monitoring (TEG) three different methods of anticoagulation 1-Heparin anticoagulation. 2-Citrate anticoagulation with 10 mmol / L Citrate. 3-Citrate anticoagulation with18 mmol/ L Citrate.

**Methods:** Thirty patients submitted to CPFA or Cytosorb had three different methods of anticoagulation :1-Unfractioned Heparin (group A). 2 – 10 mmol/L Citrate (group B) 3- 18 mmol/ Citrate. (Group C). All patients had monitoring of coagulation, ACT, and TEG at basal time (T0) and after 6 hours (T1). Blood samples for TEG were collected from the patient’s artery (site 1) and post hemofilter (CPFA) or post cartridge (Cytosorb) (site 2). All data are expressed as mean ± SD. ANOVA test was used for the statistical study.

**Results:** the TEG data is show in Table 22. Statistical comparison was made for the same site.

**Conclusions:** 1- During CPFA or Cytosorb RRT Heparine increased R (delay in activation of coagulation ) and decreased MA ( less cloth strength ) in patients , increasing the risk of bleeding. 2- 18 mmol/L citrate anticoagulation decreased MA and increased R in circuits better then in Group A and B, achieving an optimal extracorporeal anticoagulation. (1)

**Reference**

1- Crit Care Med 2015; 43:1622–1629

**Table 22 (Abstract P189).** ᅟ

	Group A site 1	Group A site 2	Group B site 1	Group B site 2	Group C site 1	Group C site 2
R	25 ± 3,2	11 ± 3	7,1 ± 2*	325 ± 22	7,8 ± 2	539 ± 25#
K	11 ± 2	3 ± 1	2,2 ± 0,9	7,4 ± 3	2,6 ± 2,1	0,1 ± 0,01
angle	33 ± 4	32 ± 3	62 ± 6	19 ± 2	69 ± 7	0,2 ± 0,01
MA	42 ± 3,2	34 ± 2,8	66 ± 9,8**	24 ± 4	57 ± 6	1,6 ± 0.3#

* p < 0,05 vs group A ** p < 0,01 vs Group A. #P < 0,01 vs. Group B and A

## P190 Calcium gluconate instead of calcium chloride in citrate-anticoagulated CVVHD

### O. Demirkiran, M. Kosuk, S. Bozbay

#### Istanbul University Cerrahpasa Medical School, Istanbul, Turkey

**Introductions:** The Ci-Ca CVVHD is a successfully implemented protocol in several European countries to conduct CRRT in patients with AKI [1]. This protocol recommends a calcium chloride solution (CaCl-S) for calcium supplementation. Due to non-availability of CaCl-S, we modified this approach and used a calcium gluconate solution (CaGl-S) instead.

**Methods:** The Ci-Ca CVVHD protocol [1] relies on modifying the calcium substitution to control s-iCa, which we applied analogously in our modified protocol. Further in the Ci-Ca CVVHD protocol, the ratio between dialysate flow (Qd) and blood flow (Qb) is linked to the effect on the acid-base status and typically set to 33 %, which we essentially applied throughout the study using 3 l/h dialysate flow at 150 ml/min blood flow. Systemic ionized Ca (s-iCa)and acid-base parameters were closely monitored to ensure safe application of our modified protocol. We retrospectively analyzed 14 critically ill patients.

**Fig. 39 (Abstract P190).**
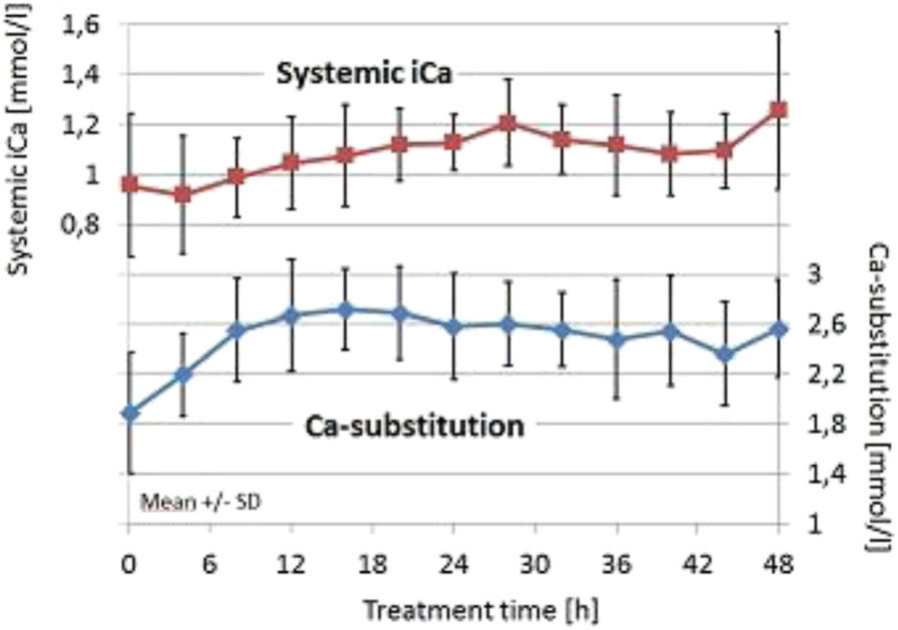
Systemic iCa and Ca substitution

**Fig. 40 (Abstract P190).**
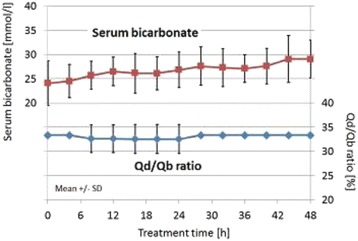
Serum bicarbonate and Qd/Qb ratio

**Results:** With respect to systemic iCa, our patients group had low values at start of Ci-Ca CVVHD, which might already explain the higher required amount of CaGl-S compared to literature data assuming that a calcium deficit in the patients is filled up, see Fig. 39. Concerning acid-base results, we did see a slight tendency to alkalosis after prolonged Ci-Ca CVVHD with CaGl-S, see Fig. 40. This might well be attributed to gluconate metabolism yielding some additional bicarbonate.

**Conclusions:** We successfully established use of a CaGl-S with the Ci-Ca CVVHD protocol. Noting the slight trend to alkalosis, we have decided to slightly reduce the blood flow to 130 ml/min. This will reduce citrate needs and less citrate will be metabolized by the patient to bicarbonate.

**Reference**

1 Slowinski et al. Crit Care 19:349

## P191 Enhanced clearance of interleukin-6 with continuous veno-venous haemodialysis (CVVHD) using Ultraflux EMiC2 vs. Ultraflux AV1000S

### V. Weber ^1^, J. Hartmann^1^, S. Harm ^1^, I. Linsberger ^1^, T. Eichhorn ^1^, G. Valicek^2^, G. Miestinger ^2^, C. Hoermann^2^

#### ^1^Danube University Krems , Krems, Austria; ^2^University Hospital St. Poelten , St. Poelten , Austria

**Introduction:** We aimed to compare the clearance of inflammatory mediators, specifically interleukin-6 (IL-6), in CVVHD using the high cut-off filter Ultraflux EMiC2 vs. the standard haemofilter AV1000S.

**Methods:** Clearance of IL-6 was compared for the two filters in an in vitro set-up using spiked human plasma (800 pg/ml IL-6) as well as in a controlled randomized clinical trial including 30 patients with sepsis and acute renal dysfuncion. The protocol was approved by the local Ethics Committee and CVVHD was performed using the multiFiltrate Acute Therapy System with Ci-Ca anticoagulation (Fresenius Medical Care). Plasma samples were taken at the start of CVVHD (0 h) and after 1, 24, and 48 h. A panel of 27 cytokines was determined in the plasma samples using a cytokine bead array. In addition, IL-6 and albumin were quantified in dialysate samples at the indicated time points.

**Results:** The in vitro clearance was significantly higher for EMiC2 as compared to AV1000S (5.00 vs. 0.03 ml/min). In clinical samples, IL-6 concentrations in the dialysate were 44.7 % vs. 30.5 %; 49.1 % vs. 14.3 %; 31.4 % vs. 7.1 %; and 29.9 % vs. 5.6 % relative to the levels in plasma at 0, 1, 24, and 48 h for EMiC2 and AV1000S, respectively. The filters did not differ significantly with respect to albumin removal.

**Conclusions:** Both in vitro and in vivo data show significantly higher clearance of IL-6 for the high cut-off filter Ultraflux EMiC2 vs. the standard haemofilter AV1000S.

## P192 Removal of bilirubin with a new adsorbent system: in vitro kinetics

### S. Faenza^1^, D. Ricci^2^, E. Mancini^2^, C. Gemelli^3^, A. Cuoghi^3^, S. Magnani^4^, M. Atti^4^

#### ^1^Teaching Hospital Policlinico S.Orsola-Malpighi, Bologna, Italy; ^2^Department of Nephrology, Dialysis, Hypertension, Bologna, Italy; ^3^Science and Technology Park for Medicine, Mirandola, Italy; ^4^Aferetica, Bologna, Italy

**Introduction:** A new sorbent (Cytosorb, Cytosorbents USA), based on blood-compatible porous polymer-beads able to adsorb hydrophobic molecules, could be a valid artificial support in many conditions of organ failure removing cytokines and toxic molecules directly from blood. At present, extracorporeal systems are based on plasma-adsorption. We performed an in vitro study on bilirubin kinetics removal to verify the system’s adsorption capacity and the ability to remove protein-bound solutes.

**Methods:** We performed 3 in vitro tests. Experiments 1&2 were done with equimolar solution of Albumin (Alb.)-Bilirubin (Bil.), with only Unconjugated Bil., strongly Alb.-bound [1], to verify the removal of protein-bound solutes. In the test 3, 24 h long, we tried to reproduce clinical conditions, with higher concentration of Bil. and lower of Alb. to study kinetics & mass balance. Solutions were recirculated in a circuit including a peristaltic pump and CytoSorb at a flow rate of 100 ml/min. Samples were collected pre and post cartridge at different times: 0, 15, 30 min and then every 30 min until the end.

**Results:** All of the experiments showed the adsorption capacity of the system concerning Bil. (Table 23) with a minimal loss of Alb. Experiments 1&2 demonstrated the capacity to adsorb protein-bound solutes. In this condition the removal of Bil. is possible only breaking the Alb.-Bil. complex. Test 3 showed a Bil. adsorption of 2.499 mg, equivalent to the removal of blood Bil. in a 70 kg patient with an initial concentration of 49,98 mg/dl. The major reduction was in the first 8 h but the cartridge maintained an adsorption capacity until the end. We could not demonstrate any release of the adsorbed Bil. in 24 h.

**Conclusions:** This in vitro study shows the effectiveness in removing Bil., any significant loss of Alb., the resin ability to break the Alb.-Bil complex and to adsorb irreversibly Bil. Cytosorb might represent a valid and simple aid in organ dysfunctions, without need of plasma separation. In vivo studies are ongoing to confirm the in vitro results.

**Reference**

[1] Weber et al. Biomacromolecules 2008, 9 1322-1328.

**Table 23 (Abstract P192).** ᅟ

	I°	II°	III°
Bilirubin, mmol/lt	0,4	0,8	0,8
Albumin, mmol/lt	0,4	0,8	0,4
Bilirubin Mass Balance, mg	572	1.020	2.499

## P193 Case series of patients with severe sepsis and septic shock treated with a new extracorporeal sorbent

### T. Laddomada, A. Doronzio, B. Balicco

#### San Marco Hospital, Zingonia, Italy

**Introduction:** Clinical studies have shown that the reduction of toxic levels of cytokines from blood with the use of a new sorbent, CytoSorb (CytoSorbents), could be useful to regain control during a complicated inflammatory condition in patients with severe sepsis/septic shock [1]. In this case series, we evaluated patients admitted to our ICU from Jan. to Nov. 2015 treated with CytoSorb. The aim was to analyse its influence in clinical outcomes, as mean arterial pressure (MAP), vasopressors need and inflammatory markers, like procalcitonin (PCT).

**Methods:** We included 8 patients (4 f, 4 m): 2 severe sepsis and 6 septic shock. Patients’ data are reported in Table 24 as median (lower and upper quartiles). All patients were non-responding to the Standard of Care for the treatment of severe sepsis/septic shock. Therefore, CytoSorb was used as adjunctive therapy in combination with continuous renal replace therapy (CRRT), in order to control the cytokines storm and improve the hemodynamic stability. It was installed in series connection after the dialyser in the CRRT circuit for 24 h (median duration of the treatment: 48 h). Clinical parameters were collected before and after every treatment with CytoSorb.

**Results:** 6 treated patients survived and during the treatment there was an overall improvement of MAP from 83 (73,5-89) to 88 (82-89,5) mmHg, with a rapid reduction in vasopressors dosages: noradrenaline decreased from 0,33 (0,15-0,46) to 0,13 (0,10-0,18) while dopamine from 7,5 (6-8) to 3 (1,5-5) Y/kg/min. Moreover, there was a markedly decrease of PCT levels from 14,53 (7,64-67,5) to 3,90 (1,62-23,05) ng/dl and an improvement in renal function, thanks to the combination of CytoSorb with CRRT. In non-survivors, MAP was hard to stabilize and decreased from 89,5 (77,75-101,25) to 69,5 (63,25-73,75) mmHg and there was an aggravation in overall patients’ conditions.

**Conclusions:** To our experience, a timely use of CytoSorb in combination with the standard therapy could have benefits in improving patients hemodynamic and helping a more rapid stabilization. However, more in vivo studies are

needed to confirm these results.

**Reference**

[1] Schadler et al. Critical Care 2013 17 (Suppl 2):P62

**Table 24 (Abstract P193).** ᅟ

Age, years	65,5 (52,25-67,25)
Type of Patient	6 Surgical /2 Medical
ICU Stay, days	17 (9,5- 27,5)
Vasopressors need, days	5 (3,25-10,5)

## P194 In vitro adsorption of a broad spectrum of inflammatory mediators with CytoSorb® hemoadsorbent polymer beads

### M. C. Gruda, P. O’Sullivan, V. P. Dan, T. Guliashvili, A. Scheirer, T. D. Golobish, V. J. Capponi, P. P. Chan

#### CytoSorbents, Monmouth Junction, USA

**Introduction:** This study quantifies the ability of CytoSorb® hemoadsorbent polymer beads to adsorb a broad selection of inflammatory pathogen- and damage-associated molecular pattern molecules (PAMPs & DAMPS) and cytokines from whole blood in an in vitro recirculation system. PAMPS cause either direct damage or trigger an immune response in the host to fight infection leading to the production of high levels of cytokines and the release of DAMPS into the bloodstream, which can trigger a maladaptive systemic inflammatory response syndrome (SIRS) that can contribute to organ injury. The benefits of cytokine reduction using extracorporeal blood filtration with hemoadsorbant porous polymer beads has been demonstrated in septic animals, yet the adsorption of other toxins and inflammatory factors may also contribute to the observed benefits.

**Methods:** Purified proteins were added to whole blood and recirculated through a CytoSorb® device or control device for 5 hours. Plasma was analyzed by ELISA.

**Results:** Hemoperfusion of whole blood through porous polymer bead devices for 5 hours removed substantial quantities of a broad spectrum of DAMPS, PAMPS (Table 25) and cytokines (ns). Levels of the inflammatory proteins were reduced by <20 % during the five hour hemoperfusion through a control device (ns).

**Conclusions:** This study demonstrates that CytoSorb® hemoadsorbent polymer beads are capable of reducing a broad range of toxic DAMPS and PAMPS from blood providing a means, in addition to cytokine reduction, of reducing the uncontrolled immune response that contributes to a maladaptive SIRS response, organ injury and death in patients with a broad range of life-threatening inflammatory conditions such as sepsis, trauma, lung injury and many others. Further study to elucidate the potential clinical impact is warranted.

**Table 25 (Abstract P194).** ᅟ

	Initial Conc. (ng/ml)	% Removed at 5 hours
C5a	25	98 ± 1.3
Procalcitonin	16	96 ± 0.7
S100A8	50	93 ± 0.5
HMGB-1	100	83 ± 7.4
Aflatoxin	10000	98 ± 0.1
TSST-1	2000	97 ± 4.5
alpha-toxin	1500	83 ± 3.2

## P195 Observations in early vs. late use of cytosorb therapy in critically ill patients

### K. Kogelmann^1^, M. Drüner^1^, D. Jarczak^2^

#### ^1^Klinikum Emden, Emden, Germany; ^2^University Hospital Eppendorf, Hamburg, Germany

**Introduction:** In several studies and in vitro data is demonstrated that the additional treatment with an extracorporal cytokine adsorption filter (Cytosorb, Cytosorbents corp.) may be helpful in patients with multiorgan failure due to increased cytokine levels [1]. CytoSorb is used as adjunctive therapy not only in septic multiorgan failure as well as in severe pancreatitis and other critical ill patients and is well tolerated and safe.

**Methods:** We collected data from 14 Patients treated additional with cytokine-haemadsorption filter (Cytosorb) as adjunctive therapy in septic shock or severe SIRS. The focus was abdominal (28.6 %), pneumonia (50 %) and pancreatitis (14.3 %). Aim of our case study was to show the effectiveness of Cytosorb treatment used as adjunctive therapy in critically ill patients. If there was no decrease of catecholamine demand and kidney injury persistent after initial therapy following actual guidelines [2], Cytosorb therapy was initiated. We collected data before, during and after treatment and calculated demand of norepinephrine (μg/h vs. thereby achieved mm Hg MAP).

**Results:** 42.8 % patients were female, mean age was 56,4 years, APACHE II Score was 37, overall survival was 35,7 %. In contrast, survival in Cytosorb therapy start delay 24 h was 66.7 %. Poor outcome increased in patients with late start of Cytosorb (survivors: therapy start delay < 48 h: 50 %, delay > 48 h: none). Cytosorb therapy start in nonsurvivors was by far later than in survivors (61,3 h vs 28,8 h). After Cytosorb therapy we observed pronounced decrease of catecholamine demand (Norepinephrine μg/h vs thereby achieved MAP): Catecholamine demand decreased 10-fold (84,81 vs 8,84). Blood lactate level was divided into halves (mg/dl: 42,7 vs 20,2).

**Conclusions:** In this case study the effects we could observe were pronounced decrease in catecholamine demand and blood lactate level. We could see increased survival if treatment with CytoSorb haemadsorption filter started between 24 – 48 h, patients who had a greater delay had poor outcome. Reasons for delay has been late acute renal failure or late admission to ICU. This implicates a therapy start not later than 24 – 48 hours.

**References**

[1] Hetz H, et al. Septic shock secondary to ß-hemolytic streptococcus-induced necrotizing fasciitis treated with a novel cytokine adsorption therapy. Int J Artif Organs 2014;37:422-6.

[2] Dellinger RP, et al. Surviving sepsis campaign guidelines for management of severe sepsis and septic shock. Intensive Care Med (2004) 30:536–555

## P196 Oxiris membrane decreases endotoxin during rrt in septic patients with basal EAA>0,6

### F. Turani, A. B. Belli, S. M. Martni, V. C. Cotticelli, F. Mounajergi, R. Barchetta

#### Aurelia Hospital /European Hospital, Rome, Italy

**Introduction:** Endotoxin > 0.6 is associated with mortality in critical care patients but few clinical studies evaluate whether Renal Replacement Therapy (RRT) with an adsorbing membrane may decrease endotoxin and modulate pro inflammatory mediators [1].

The aim of this study in a cohort of septic patients during CRRT is to evaluate. 1- The changes of endotoxin during RRT. 2- The changes of IL6 and Procalcitonin

**Methods:** Patients with renal failure and sepsis / septic shock submitted to RRT with an adsorbing membrane (oXiris , Gambro) were stratified in three groups according to basal EAA levels: Group A (EAA > 0,6 ) Group B (0,40-0,59) and Group C (0- 0,39).

All patients had monitoring of endotoxin -chemiluminiscent based endotoxin activity assay (EAA - Spectral Diagnostic, Ontario, Canada) IL6 (Elisa), procalcitonin (LUMI test PCT Braun Diagnostic Berlin, Germany) , and inotropic support at basal time (T0) , at 24 hours (T1) and 48 hours (T2).

All data are expressed as Mean Sd. ANOVA test was used to compare the changes of EAA , IL6 and Procalcitonin.

**Results:** 53 septic patients with renal failure and sepsis /septic shock were enrolled. Group A included 33 patients (62 %), Group B 14 patients (26 %) and group C 6 patients (11 %). At Table 26 are shown the changes of EAA. In Group A also IL6 decreased from to 357 ± 100 pg/mL to 143 ± 43 (p < 0,05) and Procalcitonin from 30 ± 5 to 8 ± ng/mL (p < 0,05). In Group B and C ,too, IL6 and Procalcitonin decreased significantly . 33 patients survived and 20 deceased. Survivors had at T2 lower EAA then non survivors (EAA 0,5 ± 0,2 vs .0,66 ± 0,13 . p < 0,01).

**Conclusions:** 1 The adsorbing membrane oXiris may decrease endotoxin in patients with EAA > 0,6. -2 This is accompanied by a reduction of Il6 and Pro calcitonin. 3- Endotoxin decrease to < 0,6 may increase survival. These data confirm, in part, a very recent study on liver failure treatment with a new extracorporeal liver device combining Septex filter with Oxiris.

**Reference**

1. Journal of Hepatology 2015 vol. 63 j 634¨C642

**Table 26 (Abstract P196).** ᅟ

	T0	T2	p value
Group A	0,7 ± 0,16	0,56 ± 0,16	0,001
Group B	0,5 ± 0,07	0,52 ± 0,18	ns
Group C	0,21 ± 0,05	0,37 ± 0,06	ns

## P197 An observational prospective study on the onset of augmented renal clearance: the first report

### S. Morimoto, H. Ishikura

#### Fukuoka University hospital, Fukuoka, Japan

**Introduction:** The phenomenon of augmented renal clearance (ARC) is increasingly reported in intensive care unit (ICU) patients. However, there have been few reports on ARC in Japan. The aim of this observational study was to describe the prevalence and the patient characteristics of ARC in a cohort of patients with normal serum creatinine level at a Japanese ICU.

**Methods:** We conducted a prospective cohort study from May to September, 2015 at the emergency ICU of a tertiary university hospital in Japan. The creatinine clearance (CLCR) rate of all patients was measured on admission. ARC was defined as a body surface adjusted CLCR rate of †130 mL/min/1.73 m2. We sought to study the incidence of ARC and to record the patient characteristics that were associated with ARC prospectively.

**Results:** Two hundred ninety-five patients were admitted to our ICU over the study period; 33 of whom were recruited into the present study. We identified 13 patients (39.4 %) with ARC. Patients with ARC were younger (46.5 vs. 70.7 years; p < 0.05), were less frequently male (23.1 % vs. 70 %; p < 0.05), had a lower APACHE II score (12.6 vs. 17.6; p < 0.05), and were less frequently diagnosed with sepsis (0 % vs. 40 %; p < 0.05) than patients without ARC.

**Conclusions:** ARC was a common finding in Japanese ICU patients with normal serum creatinine levels. Further multicenter studies are needed to gain a better understanding of the precise epidemiology of ARC in the Japanese population.

## P198 An ultrasound- guided algorithm for the management of oliguria in severe sepsis

### I. Hussain, N. Salahuddin, A. Nadeem, K. Ghorab, K. Maghrabi

#### King Faisal Specialist Hospital and Research Center, Riyadh, Saudi Arabia

**Introduction:** Acute Kidney Injury (AKI) is frequent in severe sepsis; 44 % in our ICU [1]. We developed a goal-directed, diagnostic ultrasonographic algorithm to assist in the evaluation in AKI. Our aims were to determine the effects on the incidence rate of AKI.

**Methods:** A prospective, observational study of patients with severe sepsis and acute oliguria (met the definition of AKI by reduced urine output of RIFLE criteria) was performed. Inferior vena cava (IVC) diameter, collapsibility, and renal perfusion with color Doppler were incorporated into an algorithm that was used by primary intensivists for volume assessment and management. The effects of the algorithm-directed interventions were assessed by serum creatinine and urine output (in ml/kg/hour) twenty-four hours later and by 28-day outcomes. The proposal was approved by the Research Ethics Committee (RAC no.2141039)

**Results:** Fifty-three patients were studied; mean APACHE II score 21 ± 7.6,mean SOFA score 9 ± 3.8. Twenty-four (45 %) patients were diabetic, 23(43 %) hypertensive, 17(32 %) cirrhotic, 34(64 %) patients were on vasopressors. Mean creatinine at time of evaluation was 147 ± 75.4, serum lactate 3.2 (1,17).Ultrasound evaluation revealed intravascular dehydration in 18 (34 %), euvolumia in 23(43 %) and fluid repletion in 9 (17 %). Based on ultrasound results, 21(39 %) patients were given loop diuretics, 10(19 %) started on continuous renal replacement therapy (CRRT), fluid boluses to 21(39 %). In 1 (2 %) patient CRRT was stopped. Serum creatinine increased by 1.5 times in 5 (9 %) patients, urine output was 0.9 ml/kg/hour (IQR 1.6). 68 % patients recovered from AKI, with 6 (12 %) either on chronic dialysis or with chronic kidney disease. At follow-up, 32 % (17 patients) had renal injury.

**Conclusions:** Incorporation of a standardized, diagnostic algorithm using bedside ultrasonographic assessment helped us to significantly reduce our rates of acute kidney injury.

**Reference**

1. Positive fluid balance is an independent risk factor for acute kidney injury in critically ill patients: results of a prospective, cross-sectional study. N Salahuddin, M Sammani, A Hamdan, M Joseph, Y AlNemary, R Alquaiz ,K Maghrabi.Critical Care 2015, 19(Suppl 1):P190

## P199 Ultrasound in acute kidney injury (aki). First findings of farius, an education-programme in structural ultrasonography

### S. K. Kloesel, C. Goldfuss, A. Stieglitz, A. S. Stieglitz, L. Krstevska, G. Albuszies

#### GPR Klinikum Ruesselsheim, Ruesselsheim (Hessen), Germany

**Introduction:** AKI is characterised by an abrupt loss of kidney function. Due to its worldwide increasing prevalence and mortality, the disease has become an important health care challenge to be treated in intensive care units (ICU)1,2]. AKI causes are numerous,~10 % of cases are postrenal obstruction with urineaccumulation. Ultrasound(US) is the procedure of choice diagnosing this cause of AKI and hence choosing the best therapy. A survey in UK revealed more than 40 % ICUs do not contact a nephrologist within 48 h in AKI. Moreover, not even 40 % of ICUs were provided with a US option for 24 h a day[3]. This is the reason, why we developed FARIUS (focused on acute renal injury with US) a programme for the education of students and physicians in diagnosing obstruction via US in an expert like manner.

**Methods:** FARIUS was approved by the ethic commission and developed as a 4 h programme for a training in renal US. To validate FARIUS, we compared US results of 22 trainees (15 students, 7 physicians) with those of 10 experts (2 ICU physician, 3 urologist, 1 nephrologist, 4 internal specialist). We present the results of two quality parameter, i.e.(i) defining the length(cm) of the right kidney of a female volunteer,(ii) diagnosing postrenal obstruction by evaluating images from US examinations. FARIUS participants voted if presented images deveal postrenal obstruction or not. Answers analyzed for correctness, specificity and sensitivity. Statistical analysis (Wilcoxon Test) were realized with INSTANT+.

**Results:** (i) FARIUS:n = 22;mean = 10,25;SD = 1,013;min = 7,5;max = 12,3;Expert:n = 10;mean = 10,15; SD = 0,463;min = 9,7;max = 11,3. Statistical analysis did NOT reveal a significant difference between groups (p = 0,823; p > 0,05). (ii) Evaluation of US images reveal 81,2 %(12,2 of 15) correct answers (mean:12,2; min = 8; max = 15; SDE = 1,84), sensitivity (98,2 %), specificity (73,2 %).

**Conclusions:** We could demonstrate, that absolvents for FARIUS are able to provide US examination to detect and evaluate postrenal obstruction at similar quality like experts. Identification of pathological results had a high sensitifity. However, the variance for the results indicates some amendment. Thereby the treatment of ICU patients with AKI could be more independent from external conditions offering an equal diagnostic quality. Economic interests and human resources could be positive affected also. Further investigations are in planning.

**Reference**

[1] Ronco C,et al. Nat Rev Nephrol, Apr;9(4):198-9,2013

[2] Li PK, et al. Transplantation, Mar 15;95(5):653-7,2013

[3] Jones SL, et al. Nephrol Dial Transplant, May;28(5):1186-90,2013

## P200 Effectiveness of renal angina index score predicting acute kidney injury on critically ill patients

### M. Aguilar Arzapalo, L. Barradas, V. Lopez, A. Escalante, G. Jimmy, M. Cetina

#### Hospital O’horan, Mérida, Mexico

**Introduction:** Until two thirds of critically ill patients develop Acute Kidney Injury (AKI) and it is associated with an increased risk of death. Renal Angina Index is a score that evaluates the risk of presenting AKI. This study was used to determine the effectiveness of the Renal Angina Index as a developing predictor of AKI in 3 days.

**Methods:** The study was based on a prospective cohort of critically ill patients in whom Renal Angina Index score was completed with a 72 hour follow up of serum creatinine levels, water balance and urinary output, establishing after patients that develop AKI. Afterwards a relationship was established between Renal Angina Index score and its predictive capacity of developing AKI, determining sensibility, specificity, positive predictive value and negative predictive value for the score.

**Results:** A final sample of 206 patients was obtained at the end of the study. The incidence of AKI in the studied population was 27.2 % (n = 56), and the average scores of Renal Angina Index were 20.52 in those who develop AKI and 4.35 in those who didn’t develop AKI. This score offers a 90.7 % sensibility, 95.4 % specificity with an area under de curve of 0.963 (0.934-0.991). A positive predictive value of 0.88 was obtained and a negative predictive value 0.97.

**Conclusions:** Renal Angina Index score is effective predicting AKI in critically ill adult patients.

**References**

1. Chawla L, Goldstein S, Kellum J, Ronco C. Renal Agina: concept and development pretest probability assessment in acute kidney injury. Critical Care. 2015; 19: 93.

2. Basu R, Zappitelli M, Brunner L, Wang Y, Wong H et al. Kidney International. 2013; 85, 659–667.

3. Basu R, Wang Y, Wong H, Chawla L, Wheeler D, Goldstein S, Incorporation of biomarkers with the renal angina index for prediction of severe AKI in critically ill children. Clin J Am Soc Nephrol. 2014; 9: 654–662

**Fig. 41 (Abstract P200).**
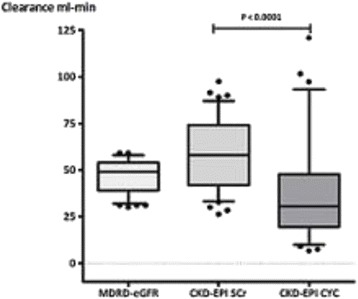
ROC Curve “Angina Index Score”

## P201 Time length below blood pressure thresholds and progression of acute kidney injury in critically ill patients with or without sepsis: a retrospective, exploratory cohort study

### J. Izawa^1^, T. Iwami^2^, S. Uchino^1^, M. Takinami^1^, T. Kitamura^3^, T. Kawamura^2^

#### ^1^Jikei University School of Medicine, Tokyo, Japan; ^2^Kyoto University, Kyoto, Japan; ^3^Osaka University, Osaka, Japan

**Introduction:** There is still uncertainty about whether interventions for blood pressure is effective to treat new-onset acute kidney injury (AKI) in critically ill patients. Moreover, little things are known about the significance of blood pressure in AKI without sepsis. The purposes of this study are to explore which parameter of blood pressure after the onset of AKI is associated with AKI stage progression, and to investigate whether the meaning of blood pressure in new-onset oliguric AKI is different between patients with or without sepsis.

**Methods:** This is a retrospective cohort study including adult patients whose stay in the ICU (Jikei University Hospital, Tokyo, Japan) for 24 hours or more after the new onset of oliguric AKI. Oliguric AKI was defined as urine output less than 0.5 mL/kg/h for 6 hours according to the Kidney Disease Improving Global Outcomes (KDIGO) definition. The primary outcome was AKI Stage 3 progression in the KDIGO criteria. In a 24-hour time frame, we analyzed mean arterial pressure (MAP) parameters (time-averaged MAP; lowest MAP; and time length below MAP 70 mmHg), considering stratification by sepsis.

**Results:** We analyzed 576 patients with oliguric AKI. Stage 3 progression occurred in 16.0 % of them. In 158 patients with sepsis on ICU admission, Stage 3 progression occurred in 29.6 % of them. When the patients were stratified by sepsis, time length below MAP 70 mmHg was significantly associated with Stage 3 progression in multivariable analyses: OR 1.09 (95 % CI: 1.02-1.16) each hour with sepsis, OR 1.07 (1.03-1.12) each hour without sepsis. In the multivariable analysis of all patients, categorized time length below MAP 70 mmHg showed increasing trends of ORs: 1.58 (0.73-3.41) for 0-6 hours, 1.71 (0.74-3.98) for 6-12 hours, 3.19 (1.24-8.24) for 12-18 hours, 6.42 (2.41-17.10) for 18-24 hours.

**Conclusions:** In a 24-hour time frame after the new-onset of oliguric AKI, time length below MAP 70 mmHg was associated with Stage 3 progression in oliguric AKI with or without sepsis. The results suggest that the recognition and treatment of AKI should be early regardless of sepsis. Urine output as a continuous monitor might be more useful for this purpose than serum creatinine. Further studies should be needed to investigate the meaning of interventions for blood pressure as treatment of AKI.

## P202 Anaemia does not affect renal recovery in acute kidney injury

### J. G. Powell-Tuck^1^, S. Crichton^2^, M. Raimundo^3^, L. Camporota^1^, D. Wyncoll^1^, M. Ostermann^1^

#### ^1^Guy’s & St Thomas’ NHS Foundation Trust, London, UK; ^2^King’s College, London, UK; ^3^Hospital de Santa Maria, Lisbon, Portugal

**Introduction:** In hospitalized patients anaemia increases the risk of developing acute kidney injury (AKI). Our aim was to determine whether anaemia also has an impact on the risk of progression from early AKI to more severe AKI in critically ill patients.

**Methods:** We retrospectively analysed the data of patients admitted to an ICU between 2007 – 2009 who had AKI stage I, as per AKI Network classification, and had undergone haemodynamic monitoring within 12 h of diagnosis. We collected baseline characteristics and differentiated between patients who progressed to AKI stage III and those who did not. Univariate and multivariate logistic regression analyses were used to identify risk factors for progression.

**Results:** 210 patients were included in the analysis. 85 patients (41 %) progressed to AKI III, 120 did not. Five patients with AKI I died before their renal function recovered or progressed. Patients with AKI I who progressed to AKI III were sicker on the day of AKI I with a higher SOFA score (9 vs 8; p < 0.001), higher arterial lactate (2 vs 1.6 mmol/L; p < 0.001), lower MAP (median 71 vs 74 mmHg; p = 0.01), lower cardiac index (median 2.6 vs 3.3 L/min/m^2^; p < 0.001) and increased need for respiratory and cardiovascular support. There was no difference in Hb concentration (96 g/L). Risk factors for progression from AKI I to AKI III are shown in Table 27.

**Conclusions:** In critically ill patients with AKI stage I, anaemia was not associated with an increased risk of progression to more severe AKI.

**Table 27 (Abstract P202).** Multivariable logistic regression models for progression to AKI stage III

Parameters on day of AKI	OR	95% CI	p-Value
Mechanical ventilation	22.12	2.40-205.52	0.006
CAD/CCF	3.17	1.55-6.49	0.002
Arterial lactate	1.57	1.15-2.13	0.004
SOFA score	1.21	1.04-1.39	0.01
CVP	1.07	1.01-1.14	0.02
Cardiac Index	0.69	0.50-0.96	0.03
MAP	0.97	0.92-1.02	0.18
Hb	0.82	0.65-1.03	0.09
SaO2	0.97	0.79-1.20	0.79

## P203 Estimated glomerular filtration rate based on serum creatinine: actual practice in Dutch ICU’s

### A. Hana, H. R. De Geus

#### ErasmusMC, Rotterdam, Netherlands

**Introduction:** Glomerular filtration rate (GFR) describes the flow rate of filtered fluid through the kidneys. Creatinine clearance (CrCl) is the volume of blood plasma that is cleared of serum creatinine (SCr) per time unit and is an important measure to approximate “true” GFR. CrCl can be estimated either by population based mathematical equations or it can be measured using serum and urine creatinine concentration after collection of urine over a certain period of time. Deterioration of CrCl/GFR identifies patients with acute kidney injury and is important for appropriate drug dosing. In our ICU practice the MDRD-eGFR equation is standard of care for a “reliable” estimation of GFR. Recently, the CKD-EPI-SCr equation was introduced as a more accurate measure especially in patients with higher GFRs. However, in critical ill patients there is no steady state of SCr, which is a prerequisite in order to estimate GFR irrespective of the mathematical formula used. We regularly treat patients with demonstrably highly impaired kidney function in spite of “normal” estimated GFR leading to toxic levels of renally cleared drugs. We investigated the actual practice of GFR approximation in Dutch ICUs.

**Methods:** Structured questionnaire via telephone.

**Results:** In June 2015 35 hospitals in the Netherlands (27 hospitals in southern Holland and 8 academic hospitals) were approached and the on-call intensive care doctor and clinical chemist were queried. At the moment of our query 15 hospitals used the CKD-EPI-SCr equation in order to estimate GFR, 20 hospitals used MDRD-equation with 5 planning to soon introduce CKD-EPI-SCr equation. In all intensive care units, ours included, eGFR on admission was used to define patient’s GFR and determine drug dosing. Intensive care doctors were conscious that eGFR might overestimate their patient’s “true” GFR. Protocols for drug level analysis (i.e. Vancomycine) exist. However, standardized measurements of patients CrCl were not performed in none of the ICU’s.

**Conclusions:** In current clinical practice mathematical equations are used to estimate GFR even in critically ill patients. Apparently, there is a shift from MDRD- toward CKD-EPI-SCr-equation. However, when applied in the critically ill both formulae provide completely incomparable values (data to be published). Therefore it is highly debatable if CKD-EPI-Scr eGFR will be a better estimate in comparison to MDRD-eGFR. Recent literature suggests that the only method that approximates Inulin-based-GFR during critical illness is a measured GFR, which we support to become clinical practice in all ICU’s.

## P204 Comparison of estimated glomerular filtration rate calculated by mdrd, ckd-epi-serum-creatinine and ckd-epi-cystatin-c in adult critically ill patients

### H. R. De Geus, A. Hana

#### ErasmusMC, Rotterdam, Netherlands

**Introduction:** The serum-creatinine based estimation of Glomerular Filtration Rate (eGFR) using the MDRD-equation is inaccurate in critically ill patients and tends to severely overestimate the “real time clearance capacity” especially in catabolic states. Recently, CKD-EPI-Serum-Creatinine and CKD-EPI-Cystatin-C equations were introduced as more precise alternatives for eGFR. Originally, all these equations have been validated in patients with stable chronic kidney disease. Most Dutch hospital laboratories, ours included, still report MDRD-eGFR in their electronic patient data system, however many intend to, or already have switched to CKD-EPI-Serum-Creatinine-eGFR. To date, (e)GFR is a key element in clinical decision making, even in the ICU setting. However, indiscriminate application of these mathematical equations may be questionable in the critically ill.

**Methods:** In order to calculate GFR applying MDRD, CKD-EPI-Serum-Creatinine and CKD-EPI-Cystatin-C formulae and assess their mutual comparability in an all-comer adult academic ICU cohort we performed a post-hoc analysis from the NGAL-study. MDRD-eGFR, CKD-EPI-Serum-Creatinine-eGFR and CKD-EPI-Cystatin-C-eGFR were calculated using admission-day data. Man-Whitney U test was used for comparison between groups.

**Results:** Out of 663 consecutive admissions, 47 cases were excluded due to missing data. Patient characteristics comprise median (95 % CI) age of 59 years (57-60), SOFA score 5 (5-5), APACHE II score 18 (17-19), male:female ratio 358:257. Analysis of eGFR by MDRD-, CKD-EPI-Serum-Creatinine en CKD-EPI-Cystatin-C resulted in significantly different outcomes. In comparison to the MDRD-equation, CKD-EPI-Serum-Creatinine-eGFR values are higher (P[<=]0.05), whereas CKD-EPI-Cystatin-C values are lower (P[<=]0.002). In direct comparison the CKD-EPI-Serum-Creatinine-eGFR is higher than the CKD-EPI-Cystatin-C-eGFR. This difference is significant for the entire cohort and persists throughout KDIGO stages 1-4 subgroups (p[<=]0.003). In KDIGO stage 5 small sample size limits interpretation of results.

**Conclusions:** In our cohort the use of the abovementioned mathematical equations results in completely different eGFR values. Particularly, the new CKD-EPI-Serum-Creatinine and CKD-EPI-Cystatin-C equations provide incomparable eGFR values. Although these formulae are supposed to be more precise, it remains questionable whether they are sufficiently reliable in the ICU-setting.

## P205 Early diagnosis of septic acute kidney injury in medical critical care patients with a urine cell cycle arrest marker: insulin like growth factor binding protein-7 (IGFBP-7)

### M. Aydogdu^1^, N. Boyaci^2^, S. Yuksel^3^, G. Gursel^1^, A. B. Cayci Sivri^3^

#### ^1^Gazi University Medical Faculty, ANKARA, Turkey; ^2^Gazi University School of Medicine, Critical Care Fellowship Programme, Ankara, Turkey; ^3^Gazi University School of Medicine Biochemistry Department, Ankara, Turkey

**Introduction:** An ideal biomarker for early diagnosis of septic acute kidney injury (AKI) should reflect renal damage at cellular level. The aim of this study is to assess and compare the role of a urinary cell cycle arrest marker, insulin like growth factor binding protein-7 (IGFBP-7) in early diagnosis of septic AKI in adult critical care patients. Besides, its role in mortality prediction was also evaluated.

**Methods:** Single center prospective cohort study. Patients without AKI, admitted to a medical intensive care unit (ICU) between January 2010 and March 2013, were included. According to their ICU followup characteristics they were grouped as sepsis-non AKI, sepsis-AKI and non-sepsis-non AKI [control]. IGFBP-7 was studied with Human ELISA Kit/96 Test/USCNK® from admission and daily collected serial urine samples.

**Results:** One hundred and eighteen patients formed the cohort; 52 in sepsis-non AKI, 43 in sepsis-AKI, 23 in control group. Admission urine IGFBP-7 predicted septic AKI development with 72 % sensitivity and 70 % specificity for a threshold level of 2.5 ng/ml with an area under the receiver operating characteristics curve (AUC) of 0.79 (95 % CI:0.70-0.88). No impact of sepsis was observed on urine IGFBP-7 levels in the absence of AKI. In septic AKI group Urine IGFBP-7 levels continuously increased upto the day of AKI development and high levels suspended for 10 days further. If it continued to be > = 4.7 ng/ml after 7 days of AKI than it predicted mortality with 83 % sensitivity and 82 % specificity (p = 0.009,AUC:0.894,95%CI: 0.742-1.046). But it was not one of the significant predictors of mortality in multivariate analysis.

**Conclusions:** Admission urine IGFBP-7 levels and following its course in ICUs can be used for early diagnosis of septic AKI development without being affected by sepsis itself. But, its role in predicting mortality needs further clarification.

**References**

1. Kashani K, Al-Khafaji A, Ardiles T et al. Discovery and validation of cell cycle arrest biomarkers in human acute kidney injury”, Critical Care 2013; 17: R25.

2. Bell M, Larsson A, Venge P, Bellomo R, Martensson J. Assesment of Cell Cycle Arrest Biomarkers to Predict Early and Delayed Acute Kidney Injury. Disease Markers 2015, Article ID 158658, 9 pages. http://dx.doi.org/10.1155/2015/15658

## P206 Urinary neutrophil gelatinase-associated lipocalin as early biomarker of severe acute kidney injury in intensive care

### J. Meza-Márquez, J. Nava-López, R. Carrillo-Esper

#### Fundación Clínica Medica Sur, Mexico City, Mexico

**Introduction:** Establish categories of risk for development of severe acute kidney injury based on the combination of levels uNGAL with various clinical variables.

**Methods:** A clinical, retrospective, observational, descriptive and analytical study in the ICU of the Medica Sur Clinic Foundation in understood period of January 1, 2013 to December 31, 2014.

**Results:** 82 patients included in the study, 37 (45.1 %) were male and 45 female (54.9 %) with a mean age of 57 ± 20 years. The main cause of admission was septic shock in 57.3 %. Creatinine levels and time uresis uNGAL significant differences with a probability less than 0.05. The cutoff for the diagnosis of acute kidney injury admission was 166.8 ng / ml, and the outcome of severe acute kidney injury, the income cutoff was 306.35 ng / ml.

**Conclusions:** There was no statistically significant difference between variables except for the creatinine levels and urinary lipocalin uresis time. Cutting uNGAL admission to the outcome of severe AKI was 306.35 ng / ml.

**References**

Cruz DN, et al. Intensive Care Med 2010;36:444-451.

Ronco C, et al. Lancet 2013;382:939-940.

Ronco C, et al. Critical Care 2013;17:117.

**Fig. 42 (Abstract P206).**
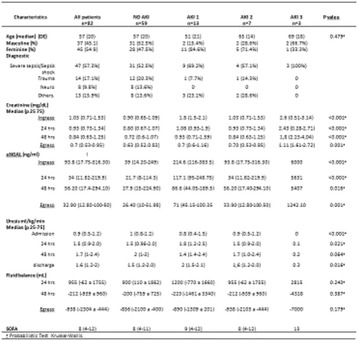
Baseline Characteristic of the study population

**Fig. 43 (Abstract P206).**
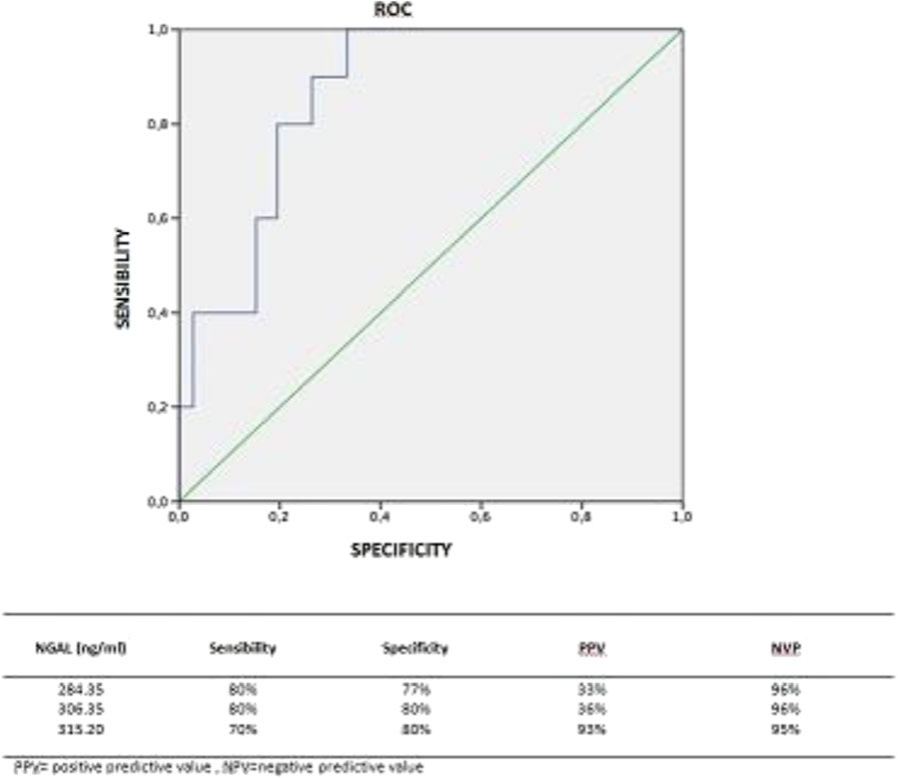
Baseline Characteristic of the study population

## P207 Shrunken pore syndrome is associated with a sharp rise in mortality in patients undergoing elective coronary artery bypass grafting

### A. Dardashti, A. Grubb

#### Lund University and Skane University Hospital, Lund, Sweden

**Introduction:** Shrunken Pore Syndrome(SPS) is suggested as a an eGFR based on cystatin C, which is lower than 61 % of their eGFR based on creatinine, i.e. when eGFRcystatin C <61 % of eGFR-creatinine.

**Methods:** The preop. plasma levels of cystatin C and creatinine were measured in 1638 patients undergoing CABG. eGFRcystatin-C and eGFRcreatinine were calculated using two pairs of estimating equations, CAPA-LMrev, and CKD-EPIcystatin C and CKD-EPIcreatinine, respectively. The patients were studied over a median follow-up time of 3.5 years (2.0-5.0 y) and the mortality determined.

**Results:** The Cox multivariate analysis demonstrated that the preop presence of SPS independently predicts poorer survival in addition to previously known risk factors. The five year survival for all patients was (mean ± 95 % CI) 90.0 ± 1.2 %,. Survival for patients with SPS defined by the equation pair CAPA-LMrev was 58.8 ± 9.0 %. Survival for patients with SPS by the equation pair CKD-EPIcystatin C-creatinine was 65.1 ± 0.6 %.

**Conclusions:** SPS is a strong, independent, predictor of mortality.

**Reference**

Grubb. Scand J Clin Lab Invest 2015;75:333-40

**Table 28 (Abstract P207).** Shrunken pore syndroms and risk for mortality after CABG

A - CKD-EPI, cut-off 0.6	B - CAPA-LMrev, cut-off 0.6	B - CAPA-LMrev, cut-off 0.7	
	p, HR, 95%CI	p, HR, 95%CI	p, HR, 95%CI
Left ventrikular EF < 30%	0.1102, 1.487, 0.914-2.419	0.0776, 1.552 0.952-2.530	0.1559, 1.426, 0.873-2.329
Shrunken Pore Syndrome	<0.0001 2.742 1.733-4.339	<0.0001 3.445 1.810-6.557	<0.0001 2.941 1.842-4.694

**Fig. 44 (Abstract P207).**
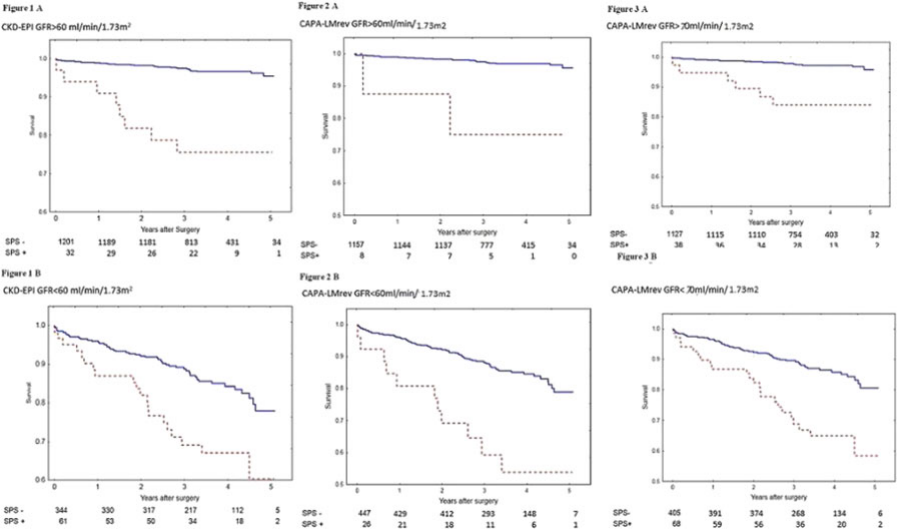
Risk of mortality after CABG by Kaplan Meier for Shrunken pore syndrom

## P208 The biomarker nephrocheck™ can discriminate the septic shock patients with an akin 1 or 2 acute renal failure who will not progress toward the akin 3 level

### J. Maizel, M. Wetzstein, D. Titeca, L. Kontar, F. Brazier, B. De Cagny, A. Riviere, T. Soupison, M. Joris, M. Slama

#### CHU Amiens, Amiens, France

**Introduction:** Patients hospitalized for septic shock have an increased risk to develop acute renal failure (ARF) and require a renal replacement therapy (RRT). The best delay to start RRT is a matter of debate. A new urine test, the Nephrocheck^TM^ has been validated. It corresponds to the urine concentration product of 2 tubular suffering markers (TIMP2 and IGFBP7) associated with a risk of developing an ARF within 12 hours. The aim of our study was to analyze the capacity of the urine TIMP2*IGFBP7 to predict the absence of evolution toward the AKIN 3 in patients hospitalized for a septic shock who present an ARF AKIN 1 or 2.

**Methods:** Every patient admitted for a septic shock in our medical ICU who presented an ARF AKIN 1 or 2 within the first 6 hours following the start of catecholamines was prospectively included. Exclusions criteria were pre-terminal chronic renal failure or chronic dialysis, anuria, patients AKIN 0 or AKIN 3. TIMP2*IGFBP7 urine assay was performed at baseline (H0) and H12. Clinical and biological parameters were collected at H0, H12 and H24. The performance of the Nephrocheck^TM^ to predict the absence of evolution toward the AKIN 3 level within the first 24 hours was tested for the baseline measurement and for the evolution of the Nephrocheck^TM^ between baseline and H12 (delta H0-H12).

**Results:** Fifty-seven patients were included, 20 (35 %) patients developed an AKIN 3 ARF (group AKIN = 3) and 37 patients did not reach this level (AKIN < 3). At H0, the urine output was lower n the AKIN < 3 (0.69 (0.47-0.99)) versus AKIN = 3 (0.33 (0.16-0.58)ml/h/kg, p = 0.007). There was no difference for the other variables at baseline including creatininemia. The TIMP2*IGFBP7 was different between the two groups at H0 (AKIN < 3 versus AKIN = 3, respectively 0.96 (0.68-1.34) versus 5.96 (2.33-10.37) p = 0.0002) and at H12 (0.46 (0.28-0.93) and 3.65 (0.95-9.98); p = 0.0008). The median delta H0-H12 was not different between the groups. A TIMP2*IGFBP7 at H0 ¡Ü1.85 predict the absence of progression toward AKIN3 with a sensibility of 80 % and a specificity of 73 %. Compared to the creatininemia at baseline (AUC 0.61, 0.47-0.74), the AUC of TIMP2*IGFBP7 (0.80, 0.67-0.89; p = 0.05) urine test was superior for assessing the risk of AKIN3. But there was no difference with the diuresis at baseline (AUC 0.72, 0.58-0.83; p = 0.33).

**Conclusions:** The Nephrocheck^TM^ and the urine output at admission predict the absence of progression from AKIN 1 or 2 toward the AKIN 3 level of septic shock patients.

## P209 A worldwide multicentre evaluation of acute kidney injury in septic and non-septic critically ill patients: the intensive care over nations (ICON) audit

### E. Peters^1^, H. Njimi^2^, P. Pickkers^1^, J. L. Vincent^2^

#### ^1^Radboudumc, Nijmegen , Netherlands; ^2^Erasme Hospital, Université Libre de Bruxelles, Brussels, Belgium

**Introduction:** Acute Kidney Injury (AKI) is a serious complication in hospitalized patients, particularly in the critically ill, in whom AKI is independently associated with poor prognosis and outcome. Sepsis is the most important cause of AKI and its pathogenesis is clearly distinct from non-septic AKI. Here, we compared the clinical course and outcome in septic and non-septic critically ill patients worldwide.

**Methods:** Intensive Care Units (ICUs) were invited to participate voluntarily in the Intensive Care Over Nations (ICON) audit. In participating centers, data was prospectively collected from all adult patients (>16 years) admitted to the ICU during a 10-day period. Patients admitted for less than 24 h for routine postoperative surveillance were excluded. Data was collected daily during the ICU stay up to 28 days, with a follow-up for outcome data until hospital discharge or death. AKI was defined according to the Risk, Injury, Failure, Loss and End-Stage (RIFLE) classification and was diagnosed during the first 48 h after ICU admission. Patients with pre-existent chronic renal failure (CRF) were analyzed as a separate subgroup.

**Results:** The study population comprised 9579 patients. At admission, 30 % of patients fulfilled sepsis criteria of which 67 % developed AKI, compared to 57 % in the non-septic population (p < 0.0001). AKI was more severe in septic patients and these patients were less likely to recover to a lower RILFE category compared to non-septic patients (improvement between day 3 and day 7 in 22 vs. 32 %, p < 0.0001). Dialysis incidence was higher in septic patients compared to non-septic patients (21 % vs. 5 %, p < 0.0001). Also in-hospital mortality rates were higher (33 % vs. 14 %, p < 0.0001), which increased to 51 % in septic and 35 % in non-septic AKI-F patients. Hospital mortality of septic AKI-F patients was similar compared to septic patients with pre-existent CRF (HR 1.6, 95%CI 1.3-2.0 compared to 1.8, 95%CI 1.5-2.1 in septic AKI-F patients). Septic patients with AKI-R or AKI-I that improved between day 3 and day 7 after admission showed a similar mortality compared to septic patients who did not develop AKI, while mortality remained elevated in AKI-F patients that improved between day 3 and 7.

**Conclusions:** AKI in sepsis is more severe, less likely to recover and associated with a higher mortality, compared to non-septic AKI.

**Reference**

1. Vincent JL et al. Lancet Respir Med. 2014 May;2(5):380-6.

## P210 Does enhanced recovery after surgery reduce the incidence of acute kidney injury in those undergoing major gynae-oncological surgery?

### M. Waraich , J. Doyle, T. Samuels, L. Forni

#### The Royal Surrey County Hospital, Guildford , UK

**Introduction:** Gynae-oncological surgery involves a patient population that is elderly, undergoing intra-abdominal surgery sometimes with tumour extension to the ureters; long anaesthetic and surgical times with extensive blood loss. All of these factors have been highlighted in the Kidney Disease: Improving Global Outcomes (KDIGO) as factors for developing acute kidney injury (AKI) [1]. The incidence of AKI in the gynaecological population is thought to be about 13 % [2]. Enhanced recovery after surgery (ERAS) pathways incorporate the use of goal directed fluid therapy with cardiac output monitoring perioperatively. Our study aim was to look at the incidence of AKI before and after the introduction of an ERAS pathway in our centre.

**Methods:** We retrospectively looked at patients undergoing major gynae-oncological surgery before and after the introduction of an ERAS pathway. There was no patient exclusion in our cohort. We used the KDIGO definition of AKI using the patient’s preoperative serum creatinine as baseline. Serum creatinine on admission to the critical care unit was taken as day 1. Data was collected for day 1, 3, 7 and 30. The main outcome of the study was the incidence of AKI pre and post ERAS introduction. Contingency analysis using Yates Chi-squared was performed.

**Results:** Our single centre cohort included 459 patients, of which 127 were in the group before the introduction of ERAS (pre ERAS) and 332 were post introduction group (post ERAS). The overall incidence of AKI was 10.24 % in the pre ERAS group and 9.04 % in the post ERAS group which was not statistically different. At day 1 the incidence of AKI was 3.94 % in the pre ERAS group and 1.52 % in the post ERAS group (p-value 0.22). At day 3 it was 8.2 % in the pre group and 6.48 % in the post group (p-value 0.67). Analysis showed there was no statistical difference between the incidence of AKI in the pre and post ERAS groups at day 1,3,7 or 30.

**Conclusions:** The incidence of AKI in our cohort of patients was lower than cited at about 10 %. There was no statistical difference in the incidence of AKI after the introduction of an ERAS pathway. Thus, we observed no reduction in the incidence of AKI with the introduction of an ERAS pathway.

**References**

1. Kidney Disease: Improving Global Outcomes. Acute Kidney Injury work group clinical practice guidelines. Kidney Intl. Suppl 2012;2:1-138.

2. Vaught AJ et al. Acute kidney injury in major gynaecological surgery: an observational study. BJOG September 2015;122(10):1340-1348

## P211 Identification of risk factors for the development of acute kidney injury after lower limb arthroplasty

### N. Desai, R. Baumber, P. Gunning, A. Sell

#### Royal National Orthopaedic Hospital, Middlesex, UK

**Introduction:** Acute kidney injury (AKI) is an independent contributor to morbidity and mortality [1]. In view of this we evaluated factors that may be associated with the development of AKI after hip and knee arthroplasty and studied the subsequent effect AKI had on length of stay (LOS) and mortality.

**Methods:** On a retrospective basis, a clinical database of information on all lower limb arthroplasties at an elective tertiary orthopaedic hospital was analysed for the time period between 1st January 2009 and 31st October 2015. AKI was defined by the Kidney Disease: Improving Global Outcomes (KDIGO) classification.

**Results:** Incidence of AKI between 2009 and 2015 was 2.99 % in 8033 patient episodes. All factors other than gender were noticeably associated with AKI. Rate of AKI was 2.7 % in elective cases compared to 10.5 % in urgent cases. Revision surgery was associated with an increased rate of AKI when compared to primary surgery. AKI increased with age and the rate of AKI in patients over the age of 80 was 9.9 %. Development of AKI progressively increased from 0.88 % in ASA 1 to 13.3 % in ASA 4 patients. Pre-operative anaemia and blood transfusion increased the risk of AKI from 2 % to 4.6 % and 2.2 % to 8.9 % respectively. All patients who developed AKI had post-operative anaemia. AKI affected 6.9 % of those patients who were admitted to the HDU while patients not admitted to HDU demonstrated a rate of AKI of 1.7 %. Patients with AKI had a mean LOS of 28.6 days compared to 9.4 days in those without AKI. Patients with no AKI had a mortality of 1.8 % at one year compared with 5.8 %, 6.5 % and 7.7 % in patients with Stage 1, Stage 2 and Stage 3 AKI respectively.

**Conclusions:** Our incidence of AKI is much lower than that reported in the literature and a retrospective study demonstrated the rate of AKI to be 14.8 % after elective orthopaedic surgery [2]. It is possible that this may be secondary to the routine post-operative admission of patients with risk factors to the HDU environment. Risk factors found to be associated with AKI were consistent with the results of previous studies [3]. Even Stage 1 AKI resulted in an increased mortality at one year. Interpretation of our findings is limited by confounding. Early recognition of risk factors will aid identification of patients at increased risk of AKI. Subsequent pre-optimisation and normalisation of reversible risk factors where possible as well as planning of appropriate post-operative care may reduce the burden of AKI.

**References**

1. Chertow GM et al. J Am Soc Nephrol 16:3365-70, 2005.

2. Kimmel L et al. Clin Kidney J 7:546-551, 2014.

3. Gross JL et al. BJA Education 15:213-8, 2015.

## P212 Incidences and associations of acute kidney injury after major trauma

### S. Lin^1^, H. Torrence^2^, M. O’Dwyer^2^, C. Kirwan^1^, J. Prowle^2^

#### ^1^Barts Health NHS Trust, London, UK; ^2^Queen Mary University of London, London, UK

**Introduction:** Acute Kidney Injury (AKI) has been identified as a common and clinically significant complication after major trauma requiring ICU admission [1].

**Methods:** We performed a retrospective, single centre observatory study of major trauma admissions to the Royal London Hospital Adult Critical Care Unit over a 30-month period to assess the incidence and associations of AKI in this population. AKI was defined by the related KDIGO definitions using creatinine criteria. Trauma-related AKI was defined as AKI by the KDIGO creatinine criteria occurring in the seven days after admission. In the absence of known baseline creatinine the admission value was used.

**Fig. 45 (Abstract P212).**
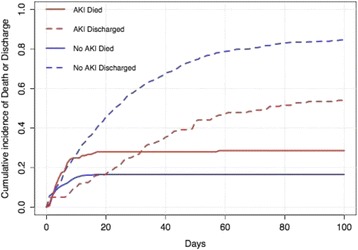
Cumulative incidence of death or hospital discharge in 858 trauma ICU patients

**Results:** 858 patients admitted to the ICU after major trauma were included in the study, 81 % were male. Median Age was 41 (IQR: 27-56), median New Injury Severity Score was 34 (22-50) and median ICU admission APACHE-2 was 12 (8-16). Overall, AKI incidence was 16 %. AKI was associated with greater hospital mortality (33 % vs. 16 % p < 0.001) and hospital length of stay (Fig. 45). In multivariable logistic regression, accounting for illness severity scoring AKI remained associated with increased in-hospital mortality (OR = 1.9, 1.2-3.2) however when broken down by AKI category only AKI-3 was significantly associated (OR = 3.4, 1.6-7.3).

**Conclusions:** Acute Kidney Injury is a common finding in critically ill patients after major trauma and is associated with greater illness severity, rates of death and length of stay.

**Reference**

Bagshaw et al 2008 Renal Failure 30:581

## P213 Acute kidney injury of major trauma patients

### T. Kim

#### Asan medical center, Seoul, South Korea

**Introduction:** Major trauma patients have more chance to reduce kidney function due to hemorrhagic shock or direct injury. This study aimed to evaluate the clinical characteristics and risk factors of acute kidney injury (AKI) in patients after major trauma.

**Methods:** We performed a retrospective cohort study in a single center. Among 386 patients who visited emergency department from January 2012 to December 2013, 322 patients with trauma were enrolled. Patients were divided into two groups, patients who developed AKI (n = 301) or not (n = 21), to compare the clinical data. We conducted multivariable logistic regression to independent predictors for AKI and mortality.

**Results:** The overall incidence of AKI was 6 % (n = 21). The mean age was 70.9 ¡¾ 12.5 years and 80.9 % were males. Also initial intervention was done embolization (n = 5, 23 %, P < 0.01), and operation (n = 7, 33 %, P < 0.45). 10 AKI patients were need to receive renal replace therapy. (47 %) Independent risk factors associated with traumatic AKI were injury severity score (ISS) [odds ratio (OR) = 1.084, P < 0.01], presence of shock (OR = 3.78, P = 0.018), and increasing of the serum creatine kinase (CK) (OR = 1.123, P < 0.01) level. Hospital mortality was higher in AKI group (23 %) than not (1 %).

**Conclusions:** The development of AKI was associated with severity of damage and organ perfusion. Furthermore, the AKI patients after trauma was refer to increase mortality.

## P214 Trajectory of serum creatinine after major surgery and the diagnosis of acute kidney injury

### M. E. O’Connor^1^, R. W. Hewson^1^, C. J. Kirwan^1^, R. M. Pearse^2^, J. Prowle^2^

#### ^1^Barts Health NHS Trust, London, UK; ^2^Queen Mary University of London, London, UK

**Introduction:** We have demonstrated that even mild and transient perioperative acute kidney injury is associated with significantly worse survival in the year after major surgery (see accompanying abstract). However, confounding effects of major illness on serum creatinine levels may cause severity of AKI to be underestimated and recovery from AKI over-estimated [1].

**Methods:** From a population of 1897 patients undergoing major surgery we examined of which 128 had AKI we examined baseline and hospital discharge creatinine-base estimated GFR (CKDEpi formula) and the trajectory of serum creatinine in patients who were hospitalized for > =5 days after surgery.

**Fig. 46 (Abstract P214).**
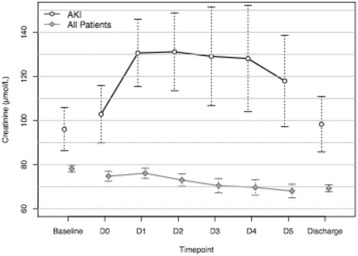
Trajectory of serum creatinine in patients with post-op LOS > 5d. Mean, SEM

**Results:** In 1836 patients who survived to hospital discharge median duration of hospitalization was 7 days (IQR: 4-11) and 11 days (6-25) in those patients who had post-operative AKI. In all hospital survivors mean eGFR rose significantly from baseline to discharge: 88.4 to 94.7 ml/min/1.73 m^2^ (p < 0.001). However in those with AKI discharge eGFR was similar to baseline 76.4 to 74.5 (p = 0.6). 1225 patients stayed > =5 days after surgery in those with AKI mean creatinine rose rapidly and eventually settled to near baseline however overall creatinine fell progressively in the five days after surgery and was ~10 % below baseline at discharge (Fig. 46). In a regression analysis a higher discharge eGFR significantly correlated with longer hospital length of stay (p < 0.001), however no such relationship existed for baseline eGFR (p = 0.79).

**Conclusions:** Baseline reduction in serum creatinine after major surgery may confound our ability to identify AKI and to assess its severity and recovery. Importantly, these effects may be more pronounced in patients with longer hospital admissions who are at higher risk of complications including AKI.

**Reference**

1) Prowle et al Clin J Am Soc Nephrol 9: 1015-1023

## P215 Epidemiology of acute kidney injury after cardiac surgery. A single center retrospective study

### S. Hanoura , A. Omar, H. Othamn, S. Sudarsanan , M. Allam, M. Maksoud, R. Singh, A. Al Khulaifi

#### Hamad medical corporation, Doha, Qatar

**Introduction:** Despite of the advances in the critical care diagnosis and treatment, the incidence of acute kidney injury (AKI) remains high. In cardiac surgery AKI is reported to be around 30 % with need for renal replacement therapy up to 1 %. [1] We aim to characterize the differences in premorbid status, etiology, intraoperative and postoperative influencing factors for AKI development and the patients’ secondary outcomes.

**Methods:** Single center retrospective observational sturdy, AKI was determined from Acute Kidney Injury Network as an acute (within 48 h) deterioration in renal functions when serum creatinine concentration entirely increase to value of greater than 0.3 mg/dL (26.4 μmol/L) from baseline or 1.5-fold increment from baseline) [2]. Patients divided into 2 groups, group I without AKI (544 patients) and group II with AKI (181 patients). The patients admission database were recorded as well as the secondary outcome measures.

**Results:** The mean age was 52.7 ± 11.6 years. We reported high AKI association in our settings (25 %). The AKI group match the other group with respect to age, gender, body mass index, the association of diabetes or hypertension. AKI group had significantly higher additive Euro Score 6 ± 4 vs 4 ± 2, lower ejection fraction 47 ± 11 vs 50 ± 9 ( P = 0.00 and 0.00 respectively. Lengths of ventilation, stays in ICU and in hospital were significantly higher in the AKI group. The AKI group with post-operative atrial fibrillation 29.8 % vs 10.5 % (p = 0.001). AKI group had significantly higher mortality 6 % vs group I 1.7 % (p = 0.026). Interestingly, AKI was more prominent in Asian group than Arab 92(50.8 %) vs 77(42.5 %) P = 0.04 The independent risk factors for AKI in our population were additive Euro Score, cardiopulmonary bypass time, post-operative low hemoglobin, post-operative white cell count and total blood loss.

**Conclusions:** Cardiac surgery induced AKI is highly predominant and prognostically substantial. Therapies targeting preoperative anemia, cardiopulmonary bypass time, and perioperative red blood cell transfusions, may display guard against this complication.

**References**

1. Rosner MH & Okusa MD. Acute kidney injury associated with cardiac surgery. Clin J Am Society Nephrol. 2006; 1(1), 19-32.

2. Mehta RL, Kellum JA, Shah SV, Molitoris BA, Ronco C, Warnock DG, and & Levin A. Acute Kidney Injury Network (AKIN): report of an initiative to improve outcomes in acute kidney injury. Crit Care. 2007;11(2):R31.

## P216 Post-operative acute kidney injury after major non-cardiac surgery and its association with death in the following year

### M. E. O’Connor^1^, R. W. Hewson^1^, C. J. Kirwan^1^, R. M. Pearse^2^, J. Prowle^2^

#### ^1^Barts Health NHS Trust, London, UK; ^2^Queen Mary University of London, London, UK

**Introduction:** Acute kidney injury is a common complication after major surgery that has been associated with adverse outcomes.

**Methods:** A retrospective study of major non-cardiac surgery in a teaching hospital over an 18 month examining the incidence of AKI with one-year follow-up. AKI was defined by KDIGO creatinine criteria. Major surgery was defined as inpatient procedures of >1 h duration. To assess the independent effect of AKI on survival, a multi-variable Cox proportional-hazard survival model was developed.

**Fig. 47 (Abstract P216).**
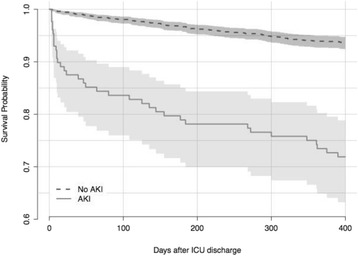
Unadjusted Kaplan-Meier plot for post-operative survival by AKI status

**Results:** 1897 patients were included, post-operative AKI occurred in 128 (6.8 %), of these 101 had KDIGO stage 1 (5.4 %), 19 stage 2 (1 %) and 8 stage 3 (0.4 %). Patients who developed AKI were significantly older, had a lower baseline eGFR, were in a higher ASA class and spent longer in hospital post-operatively compared to those without AKI. Hospital Mortality occurred in 17/128 (13.3 %) of patients that developed AKI post-operatively compared to only 16/1741 (0.9 %) of those that did not (p < 0.001). However by a year of surgery 34/94 (27 %) of patients who developed post-operative AKI had died compared to only 106/1741 (6 %) who had not had AKI (Fig. 47), p < 0.001. In the multi-variable model accenting for multiple confounders AKI was associated with a hazard ratio for death of 3.0 (1.91-4.72) in the year after surgery. Importantly AKI defined by only a 26 μmol/L creatinine rise and AKI with recovery remained associated with on-going ibcreased risk of death to one year.

**Conclusions:** In major surgery patients we have demonstrated an association between even mild acute kidney injury and increased risk of death in the next year.

## P217 Factors affecting acute renal failure in intensive care unit and effect of these factors on mortality

### O. Uzundere, D. Memis , M. Ýnal, A. Gultekin, N. Turan

#### Trakya Univ, Edirne , Turkey

**Introduction:** Early detecting acute renal failure in intensive care units is important by the aspect of determination of the disease’s severity and grade of the organ dysfuncion, discharging the patient from the intensive care units and decreasing mortality rate. The Acute Dialysis Quality Initiative workgroup proposed a classification system for acute kidney injury (AKI) identified by the RIFLE (Risk, Injury, Failure, Loss of kidney function, and End-stage kidney disease). The purpose of our study is to assess acute renal failure development in intensive care units patients, factors affecting it and the effect of the factors over mortality via using RIFLE score.

**Methods:** The age, height, weight, gender, diagnosis, comorbid diseases, admission reason to the intensive care and intensive care stay were recorded. Also in the first day, in the seventh day and in the 14.day after admission to the intensive care, the APACHE II score, SOFA score, RIFLE score, biochemical parameters (albumin, prealbumin, urea, creatinin, cholesterol, HCO3- level), triceps thickness and waist circumference measurement were all recorded. Patients was grouped into AKI and non-AKI. Patients in non-AKI group had no oliguria and obvious creatinine rise, while patients in AKI group had oliguria and obvious creatinine rise. The AKI group was assessed by RIFLE score due to hourly urine output, creatinin rise and seperated into three groups as R = Risk, I = Injury, F = Failure.

**Results:** Our study was carried out with 502 patients. 39,2 % of the patients was in the acute kidney ýnjury group while %60.8 was in non- acute kidney ýnjury group. acute kidney ýnjury group was consisting of % 21,3 (n = 107) R group, % 12,4 (n = 62) I group and % 5,6 (n = 28) F group. The renal failure development is related with high age, short body height, excessive weight, existence of chronic disease and long intensive care units hospitalization period. As a result of our study renal failure was also related with high urea, creatinine, HCO3- levels, low cholesterol, albumine and prealbumine levels. Renal failure was not related with triceps thickness measurement. Intensive care units hospitalization stay and mortality were higher in patients who develop renal failure.

**Conclusions:** we think that patients especially in intensive care units with high age, excessive weight, chronic diseases, high urea, creatinine, HCO3- levels, low cholesterol, albumine and prealbumine levels are prone to renal failure. Those patients should be carefully followed for renal failure.

## P218 Results of the live kidney transplantations according to national data of turkish organ and tissue information system

### M. A. Aydin^1^, H. Basar^2^, I. Sencan^1^, A. Kapuagasi^1^, M. Ozturk^1^, Z. Uzundurukan^1^, D. Gokmen^3^, A. Ozcan^4^, C. Kaymak^2^

#### ^1^General Directorate of Health Services, Ministry of Health, Ankara, Turkey; ^2^AnkaraNKARA Research and Training Hospital, Ankara, Turkey; ^3^Department of Biostatistics, Faculty of Medicine, Ankara University, Ankara, Turkey; ^4^Ankara Research and Training Hospital, Ankara, Turkey

**Introduction:** Although there are differences in criteria of live-kidney transplantation in organ transplantation programmes in the World, it is still performed widespread. Even though there are developments in tissue matching and immunesupressive protocols, graft loss is still an important problem after live-kidney transplantations because of acute and chronic allograft nephropathy. We aimed to assess the survival rates of patients and grafts after live-kidney transplantation.

**Methods:** The results of live-kidney transplantations between years 2011-2014 were reviewed. Patients’ age, gender and tissue antigen integration were determined. The chronic rejection and primary graft failure rates were recorded. Survival rates of the grafts and patients during 3,6,9,12,24,36 and 48 months were determined.

**Results:** The number of kidney transplantations was 11755 between 2011-2014. The source of organ in 80.8 % of the transplantations was live-donors. The mean age of the patients who had live-kidney transplantation was 40.8 ± 11.6(mean ± SD), and 65.1 %(6182) were male and 34.9 %(3314) were female. Chronic rejection and primary graft failure were determined in 2.4 %(224). Patient and graft follow-up periods were 26.20 ± 14.4(mean ± SD) and 24.8 ± 14.1(mean ± SD) months, respectively. Mean survival time of the patients was 49.42 ± 0.086 months, and survival rates for 3,6,9,12,24,36 and 48 months were 98.5 ± 0.001 %; 97.7 ± 0.02 %; 97.3 ± 0.002 %; 97.1 ± 0.002 %; 96.9 ± 0.002 % and 96.7 ± 0.002 %, respectively. Mean survival time of the graft was 47.87 ± 0.07 months in these patients and survival rates of the graft for 3,6,9,12,24,36 and 48 months were 99.2 ± 0.001 %; 99 ± 0.001 %; 98.7 ± 0.001 %; 98.5 ± 0.001 %; 97.6 ± 0.002 %; 96.7 ± 0.002 % and 96.3 ± 0.003 %, respectively.

**Conclusions:** In recent years, there is a significant increase in live-kidney transplantations in our country, due to inadequte obtaining of organ from cadaver. We observed a quite high patient and graft survival times and a low chronic rejection incidence in our live-kidney transplantation patients. Although there is a high life quality and better graft function in live-kidney transplantations compared to cadaver-kidney transplantations, cadaver-kidney transplantations should be increased.

## P219 Anaesthesia procedure and intensive therapy in patients with neck phlegmon

### V. A. Artemenko, A. Budnyuk

#### MC Into-Sana, Odessa, Ukraine

**Introduction:** Difficult intubation and extubations remains the most important problem in anaesthesia of patients with neck phlegmon [1,3]. Old guidelines for airway flow [2,3] does not fully satisfy the anesthesia procedures in patients with the neck phlegmon. We should create safe algorithm for intubation and extubation in that patients.

**Methods:** Uncontrolled prospective cohort clinical study was made in 75pts. with neck phlegmon. In main group (n = 38) intubation was performed using fibrobronhoskope or Flaplight laryngoscope with video-adapter. Tracheostomy was not performed. Sedation was by deksmedetomidine infusion. In control group (n = 37) intubation was made by conventional laryngoscope and early tracheostomy followed by surgery. Sedation by thiopental Na or propofol.

**Results:** The laryngoscope Flap-Flight upgraded with video-adapter was more efficient in case of difficult intubation. Early tracheostomy was associated with absolutely risk of mediastenitis (28,0 %) (95%CI: 0,14-0,47) in comparison with patients without tracheostomy. Dexmedetomidine was more effectiveness method for sedation which reduced reaction of cardiovascular system from 86 % to 9,5 % (χ =24,44; Ð < 0,01). In control group was 13,5 % of postextubation ARF in comparison with 0.3 % in main group. Postponed extubation was performed from 10 to 72 hours after decreasing inflammatory, larynges edema, and trizm.

Intubation algorithm: 1. Evaluate risk difficult intubation, trizm and stridor; 2. Perform laryngoscopy and classify by Kormack-Lehane: III-IV grade - use fibrobronchoscope; 3. In case of opening mouse to 1,5 cm, unable to use fibrobronchoscope - perform tracheostomy under LA. 4. In case cannot ventilate-cannot intubate surgery airway.

Exctubation algorithm:1. Postponed extubation (10-72 hrs) is recommended; 2. For sedation use dexmedetomidin infusion rate 1,5-3 mqg/kg/hrs.

**Conclusions:** The algorithm of anesthesiologist’s actions was created to provide the safe airway flow in intra- and postoperative period of patients with neck phlegmon, which reduced the incidence of serious intubations from 51,4 % to 7,9 %, failed intubations from 19 % to 0,3 %, mediastinitis from 27 % to 2,6 %, deaths from 32,4 % to 2,6 % and mortality due to traheostomy from 32 % to 6,3 %.

**References**

1. Green L.Can’t intubate,can’t ventilate! A survey of khowledge and skills/L.Green//EJA2009.–Vol.26–P.480-483.

2. Algorithm of difficult intubations trachea/Chuev P.N.–Kiev,2007.–p 52.

3. Burov N.E. Algorithm of safe airway flow//Clinical anaesthesiology-2005.–V.2-^1^4.–P2-15.

## P220 Nasal high flow oygen for acute respiratory failure: a systematic review

### R. Pugh, S. Bhandari

#### Glan Clwyd Hospital, Rhyl , UK

**Introduction:** Nasal high flow oxygen (NHFO) is an attractive therapy for acute hypoxemic respiratory failure (AHRF), with reported comfort, and theoretical wash-out of CO2 and generation of PEEP. However, its clinical efficacy is uncertain. We undertook a systematic review of current evidence to support its use in adults.

**Methods:** Medline and EMBASE were searched using the terms: nasal AND (high-flow OR high flow) AND (oxygen OR oxygen therapy. RCTs were included if comparison was made between NHFO and standard O2 therapy, or NHFO and CPAP or NIV, for AHRF in adults, and primary outcomes (intubation and mortality) reported on.

**Results:** We identified 468 potentially relevant studies. 4 RCTs met inclusion criteria. There were important differences in patient groups, interventions and outcome measurements, precluding meta-analysis. There were no significant differences in intubation rate or mortality between intervention groups, with the exception of Frat (2015), who demonstrated significantly reduced 90d mortality in NHFO compared with standard O2 and NIV groups, and lower intubation rate on post hoc analysis of patients with lowest PF ratio.

**Conclusions:** There is only limited evidence from RCTs at present to support use of NHFO rather than conventional therapy for acute hypoxemic respiratory failure in adults, and further study of patients at risk of requiring invasive ventilation is needed.

**References**

1. Jones RG Respir Care 2015

2. Bell N Emerg Med Austral 2015

3. Frat J-P NEJM 2015

4. Lemiale V Crit Care 2015

**Table 29 (Abstract P220).** Study characteristics, and intubation outcome

Study	Setting	Participants	NHFO	Standard O2	
Jones 2015	ED	Tachypnoea, hypoxia	1/165	3/138	p = 0.23
Bell 2015	ED	Tachypnoea, hypoxia	0/48	1/52	p = 0.33
Frat 2015	ICU	Respiratory failure	40/106	44/94	p = 0.19
Lemiale 2015	ICU	Immunocompromise/ respiratory failure	5/52	4/48	p = 0.82

## P221 Setting optimal flow rate during high flow nasal cannula support: preliminary results

### T. Mauri^1^, C. Turrini^2^, T. Langer^1^, P. Taccone^1^, C. A. Volta^2^, C. Marenghi^1^, L. Gattinoni^1^, A. Pesenti^1^

#### ^1^Fondazione IRCCS Ca’ Granda, Maggiore Policlinico Hospital, Milan, Italy; ^2^University of Ferrara, Sant’Anna Hospital, Ferrara, Italy

**Introduction:** High Flow Nasal Cannula (HFNC) is a non-invasive respiratory support that might impact major clinical outcomes of acute respiratory failure patients [1]. We present preliminary data from a clinical study that aims to describe physiological effects of HFNC at different flow rates.

**Methods:** We performed a prospective randomized cross-over study on 4 acute respiratory failure patients admitted to the ICU with PaO[sub]2[/sub]/FiO[sub]2[/sub] < =300 mmHg. FiO[sub]2[/sub] was set to obtain SpO[sub]2[/sub] of 90-95 % by facial mask and left unchanged during the study. All patients underwent 4 randomized steps (20 min): 1. Facial mask at 12 L/min; 2. HFNC at 30 L/min; 3. HFNC at 45 L/min; 4. HFNC at 60 L/min. During all phases, continuous recordings of Electrical Impedance Tomography (EIT) data calibrated by spirometry and esophageal pressure (Pes) tracings were obtained.

**Fig. 48 (Abstract P221).**
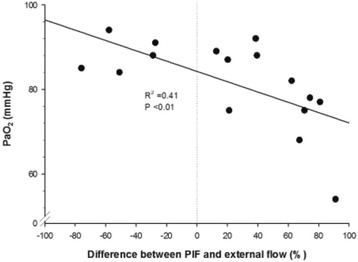
ᅟ

**Results:** At higher flow rates, PaO[sub]2[/sub]/FiO[sub]2[/sub] improved (mask: 162 ± 78 vs. HFNC 30: 187 ± 82 vs. HFNC 45: 199 ± 79 vs. HFNC 60: 198 ± 68 mmHg; p < 0.005) and Borg dyspnea score decreased (6.2 ± 3.0 vs. 5.0 ± 2.4 vs. 5.0 ± 2.4 vs. 4.2 ± 1.7; p < 0.05). Pes swings, a measure of patients’ inspiratory effort, decreased with HFNC (10.9 ± 5.5 vs. 7.9 ± 2.9 vs. 8.1 ± 4.1 vs. 7.4 ± 2.8 cmH[sub]2[/sub]O; p < 0.05). Peak inspiratory flow (PIF), as assessed by EIT [2], decreased at higher flow rates (60.6 ± 27.5 vs. 53.2 ± 22.5 vs. 53.9 ± 24.1 vs. 50.0 ± 21.4 L/min; p < 0.05), suggesting decreased respiratory drive. Interestingly, when patient’s PIF exceeded the flow delivered by each external source, oxygenation was lower, likely indicating higher discrepancy between externally set and alveolar FiO[sub]2[/sub]’s (Fig. 48).

**Conclusions:** HFNC improves oxygenation and reduces inspiratory drive and effort, thus decreasing the risk of respiratory decompensation. Optimal flow rate during HFNC might be titrated based on patient’s inspiratory flow.

**Reference**

1. Frat JP et al. N Engl J Med 2015 2. Bodestein M et al. BMC Pulm Med 2014

## P222 Dose to dose consistency across two different gas flow rates using cystic fibrosis and normal adult breathing profiles during nasal high flow oxygen therapy

### L. Sweeney, A. O’ Sullivan, P. Kelly, E. Mukeria, R. MacLoughlin

#### Aerogen, Galway, Ireland

**Introduction:** This study investigated the dose to dose consistency by aerosol delivery during nasal high flow therapy (NHF). We sought to investigate the efficiency of dosing across two different gas flow rates during simulated normal and cystic fibrosis (CF) breathing. Salbutamol was used as a tracer aerosol. The efficient and reliable delivery of aerosol to CF sufferers by a non-invasive, comfortable means of delivery could have considerable effects on patient care.

**Fig. 49 (Abstract P222).**
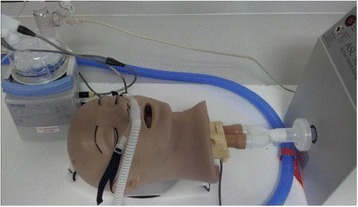
Tracheal Dose Test Setup

**Methods:** Two adult breathing patterns were evaluated using a breathing apparatus to simulate both a normal adult (15 BPM, Vt 500ML, I/E 1:1) [1] and a Cystic Fibrosis adult (22 BPM, Vt 525ML, I/E 1:4) [2] Adult high flow nasal cannula (Optiflow^TM^ Fisher and Paykel) were attached to a humidifier (MR850^TM^, Fisher and Paykel) set at 37 °C. 2.0 ml of Salbutamol (2 mg/ml) was nebulized using a vibrating mesh nebuliser (VMN) (Aerogen Solo, Aerogen, Ireland) with an average mass median aerodynamic diameter (MMAD) of 4.8 μm (measured using the Apparatus E/5 impactor). Tracheal Dose (dose delivered beyond the trachea) at each gas flow rate under test (10 and 30 LPM) was captured using an absolute filter (Respirgard 303, Baxter) distal to the airway model (n = 10), see Fig. 49. The mass of drug eluted from the filters was determined using UV spectroscopy (at 276 nm) and interpolation on a standard curve of Salbutamol concentrations (200 μg/mL to 3.125 μg/mL).

**Results:** The results of testing are presented in Table 30. Time to delivery of the 2.0 ml dose of Salbutamol was recorded at approximately 4.25 minutes for each run. Results are expressed as a percentage of the nominal dose placed in the nebuliser medication cup.

**Conclusions:** From the results of testing performed for both normal and CF simulated adults at gas flow rates of 10 and 30 LPM, the p-values for each respectively are >0.05 (0.891, 0.744, 0.188 and 0.589). This indicates that each set of data (n = 10) are normally distributed and therefore dosing is consistent. It may be concluded from this study that delivery of aerosol to adults at both high and low gas flow rates via NHF therapy is an efficient and highly reproducible means of administration.

**References**

1 Hart N et al. Am J Respir Crit Care Med 2002; 166:61-66.

2 Tiddens et al. Eur Respir J 2000; 15:735-742.

**Table 30 (Abstract P222).** Tracheal Dose in Both Normal and CF Adult. (n = 10)

Gas Flow Rate (LPM)	Normal	Normal	Normal	CF	CF	CF
	Mean Tracheal Dose (%)	SD	P-value	Mean Tracheal Dose (%)	SD	P-value
10	20.46	1.6940	0.891	13.84	0.3471	0.188
30	13.85	0.4200	0.744	9.38	0.2563	0.589

**Fig. 50 (Abstract P222).**
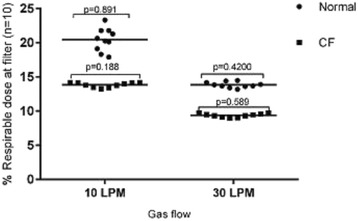
Illustration of CF and Normal adult data distributions (n = 10)

## P223 Final results of an evaluation of airway medix closed suction system compared to a standard closed suction system

### M. Pfeffer^1^, J. T. Thomas^2^, G. B. Bregman^1^, G. K. Karp^1^, E. K. Kishinevsky^1^, D. S. Stavi^1^, N. A. Adi^1^

#### ^1^Kaplan Medical Centre, Rehovot, Israel; ^2^West Virginia University, Morgantown, West Virginia, USA

**Introduction:** Endotracheal Tube (ETT) begins to be colonized with oral bacteria and respiratory pathogens that adhere to its inner surface. The use of Airway Medix Closed Suction System (AMCSS) could effectively reduce ETT resistance, bacterial burden, biofilm development and the Clinical Pulmonary Infection Score (CPIS). The goal of this study was to evaluate the efficiency of the AMCSS compared to a standard closed suction system.

**Methods:** 80 critically ill patients were randomized into two groups. 40 patients (Control) were suctioned using KIMVENT* Closed Suction System (Kimberly Clark, USA) and 40 patients (Study) were suctioned using AMCSS (AMCSS, Biovo Technologies, Israel). 15 patients were withdrawn from the trial. CPIS calculations were performed daily. Post extubation, resistance through the ETT was measured using a Mass flow meter and pressure transducer. The ETTs were then cut open longitudinally. Two 1 cm-long hemi-sections of the distal part of the ETT were dissected for qualitative and quantitative analysis of representative biofilm accumulations using Scanning Electron Microscopy (SEM). During the analysis, investigators were blind to treatment allocation.

**Results:** Mean resistance at flow rate of 60[l/min] was lower in the Study group compared to the Control group – 11.85 vs. 14.33 [cmH2O/l/s]. This difference was significant according to Mann-Whitney test. CPIS was found to be lower in the Study group compared to the Control group (4.9 vs. 5.9). The proportion of subjects with CPIS above 7 was lower in the Study arm (3.5 %) compared to the Control (25 %). This difference was significant according to Mann-Whitney test [Table 31]. Biofilm stage was lower in the Study group compared to the Control—2.26 vs. 2.72. In addition, patients that were treated with AMCSS had 2.933 times higher probability for a lower biofilm stage in comparison to Control group.

**Conclusions:** The results conclude that the use of AMCSS reduces the resistance, CPIS and biofilm formation and can be effective in limiting the biofilm development to the early stages.

**Table 31 (Abstract P223).** CPIS Results

Device	Mean CPIS	Number of patients with CPIS > 7
Control (KimVent)	5.9	9/36 (25%)
Study (AMCSS)	4.9	1/29 (3.5)

## P224 Different cuff materials and different leak tests - one size does not fit all

### T. Poropat, R. Knafelj

#### Rihard Knafelj, Ljubljana, Slovenia

**Introduction:** Once inflated endotracheal tube cuffs can form microfolds enabling microaspiratoins. We tested adequacy of methylen blue visual test.

**Methods:** 10 porcine tracheas were intubated with different cuff shape/materials. Barrel and tappered shaped cuffs made of polyvinyl chloride (PVC) and polyurethane (POLY) were tested. Following intubation cuffs were filled with air (25 cmH2O cuff pressure). 3 mL of methylene blue (1%water solution) with iodine contrast (Iomeron 300) was instiled in trachea. Same protocol was applied using 20 mL syringes instead of tracheas. After 1 and 24 h visual confirmation and CT 3D reconstruction was performed to evaluate distal tracheal leakage.

**Results:** No CT or visual leakage was detected in POLY. No visual leakage was confirmed in tubes with supraglotic suctioning, while some leakage was visible on CT, POLY performed better. PVC leaked visually and on CT within the first hour. All cuffs had superior seal in cylindrical model of trachea, POLY performing better.

**Conclusions:** POLY cuffs offered superior seal to PVC cuffs regardless of shape, adequate seal was maintained through 24 h. Methylene blue compared can not confirm micro leakage. Syringe is not satisfactory surrogate to actual trachea.

**Reference**

Optimal care and design of the tracheal cuff in the critically ill patient. Jailette et all Ann Int Care 2014;4:7

**Fig. 51 (Abstract P224).**
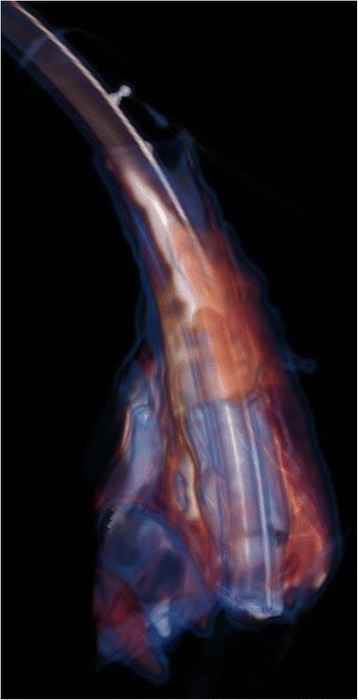
ᅟ

## P225 Observational study on the value of the cuff-leak test and the onset of upper airway obstruction after extubation

### E. Llopart, M. Batlle, C. De Haro, J. Mesquida, A. Artigas

#### Corporació Sanitària i Universitària Parc Taulí, Universitat Autònoma de Barcelona, CIBER Enfermedades Respiratorias, Sabadell (Barcelona), Spain

**Introduction:** The cuff-leak (CL) test has been postulated as a useful tool in order to assess the risk of upper airway obstruction (UAO) after extubation. However, current evidence is still limited, and systematic exclusion of patients receiving corticosteroids in prospective trials further limits our knowledge. Our objective was to evaluate the association of the value of the CL test and the appearance of UAO, including patients receiving corticosteroids, and to detect other associated risk factors.

**Methods:** Prospective observational study in a general mixed ICU (May-December 2014). Daily screening of patients considered ready to wean by their medical team was performed. Only those patients who succeeded a spontaneous breathing trial and were eligible for extubation, according to the local protocol, were included. Demographic and clinical data were collected. We recorded the CL value (%) at the end of the successful spontaneous breathing trial, prior to extubation. The appearance of UAO, the need for reintubation, and reinstitution of MV were recorded.

**Results:** Thirty-three patients were studied. The mean age was 65 ± 12 years old, 64 % were males, 10 patients (30 %) were receiving corticosteroids, and the total duration of MV at inclusion was 13 ± 7 days. After extubation, four patients (12 %) developed UAO, requiring reintubation in two cases. The CL test values were significantly lower in those patients who developed UAO (35 ± 22 % vs 61 ± 25 %; p = 0.05). The ROC curve analysis of the value of CL test in predicting UAO showed an AUC of 0.8 (95 % CI 0.61 to 0.98; p = 0.06). Other factors associated with the appearance of UAO were a difficult intubation (75 % vs 8 %, p = 0.001.), number of attempts in the initial intubation (2 vs 1, p = 0.001), and prehospital intubation (50 % vs 3 %, p <0.01). No differences in Cormack, MV days or diameter of the endotracheal tube were found. None of the patients receiving corticosteroids prior to extubation developed UAO, whereas among the remaining 23 patients, 4 (17 %) developed UAO (p =0.1).

**Conclusions:** In our sample, the appearance of UAO after extubation was associated with significantly lower CL test values. Further studies are required in order to evaluate the usefulness of the CL test and corticosteroid therapy in the prevention of UAO.

## P226 A device for emergency transtracheal lung ventilation

### D. Pavlovic^1^, L. Lewerentz^2^, A. Spassov^2^, R. Schneider^2^

#### ^1^Dalhousie University, Halifax, Canada; ^2^Ernst-Moritz-Arndt-Universität, Greifswald, Germany

**Introduction:** Here we present an improved theoretical version of a valve for transtracheal ventilation as a bi-directional manual respiratory pump where a combination of low flow during inspiration, by reducing gas supply to the valve, and increased flow during expiration, by increasing gas supply to the valve, permits more effective venturi effect and efficient expiration, with low total gas consumption.

**Fig. 52 (Abstract P226).**
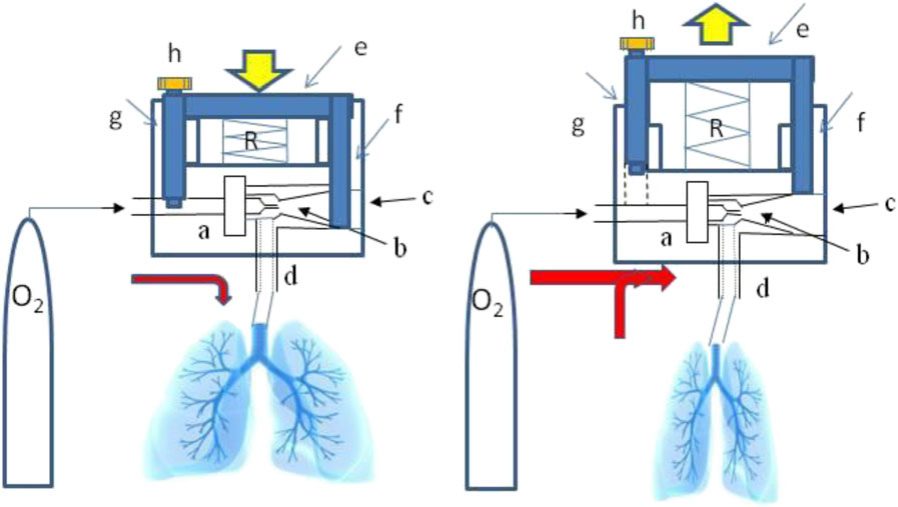
A device for Emergency Transtracheal Lung Ventilation

**Methods:** The theoretical performance of the valve was modeled mathematically and the model was tested in vitro with a standard valve but by variable flow rates (Fig. 52).

**Results:** It was shown that, by increasing flow during expiration, the valve would permit to shorten the expiratory time and achieve higher minute volumes (i.e. volumes of 7 L/min of gas or higher), as compared with the ventilation with the similar transtracheal cannula without variable flows (volumes achieved were about 4 L/min). Variable flow would provide shortening of the inspiratory time and efficient expiratory aid, and permit I:E ratios of 1:1, or even the inverse ratio ventilation.

**Conclusions:** Satisfactory lung ventilation can be assured with transtracheal ventilation with a bidirectional manual respiration valve with variable gas flow.

## P227 Long-term outcome and health-related quality of life in patients discharged from the intensive care unit with a tracheostomy and with or without prolonged mechanical ventilation

### S. De Smet, S. De Raedt, E. Derom, P Depuydt, S. Oeyen, D. Benoit, J. Decruyenaere

#### Ghent University Hospital, Gent, Belgium

**Introduction:** Long-term outcome and quality-of-life (QoL) in patients requiring prolonged mechanical ventilation (PMV) after failure to wean is scarcely documented. However, due to improvements of respiratory care in the ICU, an increasing amount of patients requires PMV. This study evaluates long-term survival and QoL in patients treated by tracheostomy for difficult weaning, with or without need for PMV at ICU discharge.

**Methods:** We retrospectively investigated post-ICU trajectories and survival in patients admitted at the medical ICU of a tertiary center between 1999 and 2013. Only patients requiring tracheostomy for respiratory failure of varying etiology and difficult weaning were included. Patients were discriminated between those who did or did not require ventilation at ICU discharge. Patient characteristics affecting survival rates were examined. QoL assessment was done in survivors using the Short Form Health Survey (SF-36) and the Severe Respiratory Insufficiency (SRI) questionnaire in 2014.

**Results:** A total of 114 patients was included, of whom 59 required mechanical ventilation at ICU discharge and 55 were weaned from ventilation at that point. One-year survival rates were 73 % and 69 %, respectively. In patients with a follow-up of at least five years, 5-years survival rates were 40 % (16/40) for ventilated and 42 % (14/33) for weaned patients. In the ventilated group, survival rates were significantly lower in the elderly (p = 0.001) and in patients with a high Charlson Comorbidity Index (p = 0.02) or chronic kidney disease (p < 0.001). QoL was assessed in 21 ventilated and 22 weaned patients after a median of 58 and 52 months. Response rates were 84 % and 81 %, respectively. Overall, QoL scores for physical functioning were low in both groups. QoL for physical functioning, assessed with SF-36, was lower in the ventilated (8/100) than in the weaned group (20/100). Scores for social functioning and mental health were less below norm and similar between both groups (62 and 52/100 vs. 62 and 48/100, respectively). A similar trend was observed with the SRI questionnaire.

**Conclusions:** Long-term survival in patients selected for PMV with tracheostomy after failure to wean was not significantly different from that of weaned patients with tracheostomy. This suggests that survival of these patients depends more on the underlying disease(s) than on the ventilation itself. Despite the physical QoL scores being low in both groups, mental QoL was still acceptable.

## P228 Ultrasound-guided percutaneous dilational tracheostomy versus bronchoscopy-guided percutaneous dilational tracheostomy in critically ill patients (trachus): a randomized clinical trial

### A. Gobatto, B. Besen, P. Tierno, L. Melro, P. Mendes, F. Cadamuro, M. Park, L. M. Malbouisson

#### Universidade de São Paulo, São Paulo, Brazil

**Introduction:** Percutaneous dilational tracheostomy (PDT) is routinely performed in the intensive care unit (ICU) with bronchoscopy guidance. Recently, ultrasound has emerged as a potentially useful tool to assist PDT as it helps to identify the most appropriate location for the tracheal puncture site, guide needle insertion into the trachea, and may significantly reduce procedure-related complications.

**Methods:** An open-label, parallel, noninferiority, randomized controlled trial was conducted comparing ultrasound-guided PDT with bronchoscopy-guided PDT in mechanically ventilated critically ill patients indicated for a tracheostomy. The primary outcome was procedure failure defined as a composite end-point of conversion to surgical tracheostomy; associated use of bronchoscopy in the case of ultrasound-guided PDT; associated use of ultrasound in case of bronchoscopy-guided PDT; or occurrence of a major complication.

**Results:** A total of 4965 patients were screened, of whom 118 underwent the procedure, 60 patients in the ultrasound group and 58 patients in the bronchoscopy group. Procedure failure occurred in one (1.67 %) patient in the ultrasound group and one (1.72 %) patient in the bronchoscopy group, with an absolute risk difference of 0.06 % between groups (90 % confidence interval, -5.57 to 5.85) in favor of the ultrasound group in the as treated analysis, not including the prespecified margin of 6 % for noninferiority. No other patient had any major complication in both groups. Procedure-related minor complications occurred in 20 (33.3 %) patients in the ultrasound group and 12 (20.7 %) patients in the bronchoscopy group, (P = 0.122). The median procedure length was 11 [7-19] vs. 13 [8-20] minutes (P = 0.468), respectively and clinical outcomes were also not different between groups.

**Conclusions:** Ultrasound-guided PDT is noninferior to bronchoscopy-guided PDT in mechanically ventilated critically ill patients.

## P229 Is it safe to discharge patients with tracheostomy from the ICU to the ward?

### B. C. Civantos, J. L. Lopez, A. Robles, J. Figueira, S. Yus, A. Garcia

#### Hospital La Paz, Madrid, Spain

**Introduction:** Many of the patients admitted to the ICU receive a tracheostomy and are discharged whit it to the general ward. Insufficient skills and experience of staff caring for tracheostomy patients may lead to suboptimal care and increased morbidity and mortality. Tracheostomy outreach teams and an appropriate training to the staff are the key to improve quality of care, decrease adverse events and make decannulation with safety in these patients.

**Methods:** We conducted a retrospective study in a medical ICU in a tertiary care hospital that doesn’t have a step-down unit. A two intensivists team followed up daily the patients discharged with tracheostomy from the ICU to the general ward until they were discharged from the hospital. Training sessions to the ward,s staff (doctors, nurses and nursing assistants) were made about tracheostomy cares every week. We analized clinical and epidemiologic variables, time to decannulation, complications related to the tracheostomy, decannulation failures, readmissions and mortality.

**Results:** From February 2012 to October 2015, a total of 2568 patients were admitted to our ICU; during this period, 100 patients were discharged to the regular ward with tracheostomy tube in place. Of these, 68 % had neurological damages (due to head injury, ischemic and hemorrhagic events), 14 % chronic obstructive pulmonary disease, 8 % severe miopathy, 6 % postanoxic encephalopathy, 2 % Guillain Barre and 2 % amyotrophic lateral sclerosis. 10 % of the patients with a high length of stay had complications because of the tracheostomy (tracheal stenosis, granuloma and malacia), and all of them were solved. There were 2 readmissions to the ICU due to cannula obstruction. Decannulation at the ward was possible in 36 % of the patients, none of them had to be recannulated and all survived. Global mortality was 23 %, but excluding the patients under palliative cares, the mortality decreased to 5 % and the causes of death were not directly related to the tracheostomy.

**Conclusions:** In hospitals without a step-down unit and a high healthcare demand the monitoring of the tracheostomized patients by a skilled team let to decannulate the patients at the ward with safety. It would allow discharge sooner this kind of patients from the ICU and therefore decrease the length of stay, the complications associated to the ICU admission and accordingly the costs. On the other hand, once at the ward, the team can detect early the complications, solve them and create a safety atmosphere for the patient, the family and the staff in charge.

## P230 The application of tracheostomy in children in ICU

### A. Oglinda^1^, G. Ciobanu^2^, C. Oglinda^1^, L. Schirca^1^, T. Sertinean^1^, V. Lupu^3^

#### ^1^Institute of Mother and Child, Chisinau mun., Moldova; ^2^Institute of Emergency Medicine, Chisinau mun., Moldova,^3^State University of Medicine and Pharmacy, Chisinau mun., Moldova

**Introduction:** The study arises from the need to implement some therapeutic conducts by applying tracheostomy in children who have been in prolonged artificial pulmonary ventilation over the last 8 years.

**Methods:** The patients included the study groups are 96 children from the pediatric ICU of the Institute of Mother and Child. Children submitted to study were divided into 2 groups. 1st group - 61 children (63.5 %) on prolonged artificial pulmonary ventilation without tracheotomy application and 2nd group - 35 children (36.4 %) to whom during prolonged artificial ventilation management the tracheotomy was applied.

**Results:** After a retrospective study, we have noted the following: children on prolonged artificial ventilation spent on avg. 24.7 ± 3.3 bed/days. The classification by disease of children requiring intubation with prolonged ventilation was the following: Multiple development abnormalities - 31 cs (32.2 %); Brain and neuromuscular disorders - 25 cs (26.0 %); Bronchopulmonary Chronic diseases - 16 cs (16.6 %); Genetic diseases - 12 cs (12.5 %); septic conditions - 10 cs (10.4 %); Development abnormalities of diaphragm - 2 (2.0 %). In the 2nd group, our study revealed the following complications: complication of subcutaneous emphysema - 6 cs (17.1 %), but pneumomediastinum - 2 cs (5.7 %), lobar emphysema - 1 cs (2.8 %), suppurations - 2 cs (5.7 %), situations that were resolved. All children received a special critical care under the Protocol of pneumonia prophylaxis associated with artificial ventilation: gentle suctioning of secretions with closed suction systems after cuffinflation. Placing on 30° and oral hygiene for 2-3 times/day being mandatory for each one. Also, a daily hydroelectrolitic rebalancing, calculation of individual energy need and symptomatic therapy was performed. Along with the improvement of general condition, appearance or improvement of the deglutition reflex, the oral nutrition with semi-solid preparations recommenced in 5 children (22.5 %) from the first day of tracheostomy application, in 7 cs the nutrition recommenced on 3-5 day together with partial parenteral nutrition until the full resumption of oral nutrition. It is worth noticing also the significant decrease in length of stay on artificial respiration in the 2ndgroup of children (22 ± 0.9 days) compared to the 1stgroup (37 ± 1.7 days). Despite the complex therapeutic effort, due to severity of basic disease in the 1 group 15 (24.5 %) children died and in the 2nd group 3 children (8.5 %) died.

**Conclusions:** 1.The application of tracheotomy in patients with endotracheal intubation and prolonged artificial ventilation reduces the infant mortality rate.

2. In the 2 group we have noticed the decrease of the duration of treatment, which leads to a reduction in the prime cost of the child treated in the intensive care unit.

## P231 The impact of passive humidifiers on aerosol drug delivery during mechanical ventilation

### P. Kelly, A. O’Sullivan, L. Sweeney, R. MacLoughlin

#### Aerogen, Galway, Ireland

**Introduction:** The purpose of this study was to determine the impact passive humidifiers (PH) on delivered aerosol dose.

The prevalence of PH usage during invasive mechanical ventilation where the upper airway is bypassed is on the increase due to their low cost and ease of use [1-2]. However PH are not without their limitations. These include blockages and aerosol therapy (AT) associated issues [3-4]. PH manufacturers have identified the restraint of use with AT as a major area for development. Bypass PH for use with AT have been developed by Smiths-Medical, Hudson RCI and Carefusion. Other companies have developed non-bypass PH that are compatible with AT, such as Pall and Intersurgical.

**Fig. 53 (Abstract P231).**
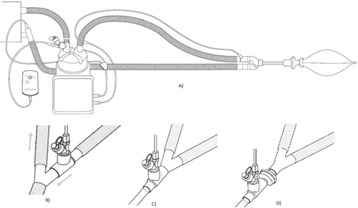
A)dry side of HH B)inspiratory limb C)between Wye & ETT D)between HMEF and ETT

**Methods:** The nebulizer positions are shown Fig. 53. A ventilator (SERVO-i®, Maquet) was operated with the conditions- Vt 500 mL, 15BPM, I:E 1:2 and PEEP 5 cm.H2O, with a heated pass-over humidifier (Fisher & Paykel) with heated-wire circuit attached to an 6.5-mm inner-diameter endotracheal tube (ETT)(Flexicare). The absolute filter (Respirgard 303, Baxter) was positioned elevated to the distal tip of the ETT. The HH conditions were 38 ± 1 °C. For Passive humidification conditions, the PH were pre-conditioned for 4 hours under heated and humidified conditions. A vibrating-mesh nebulizer (Aerogen Solo, Aerogen) was used. A nominal dose of 2.5 mL of Salbutamol (2 mg/mL) was used. The mass of drug eluted from the filters was determined using UV spectroscopy. 3 commercially available PH were used, Ultipor100 (Pall) and Filta-Therm (Intersurgical) compatible with AT, while the Airlife (Carefusion) was not approved for use with AT.

**Fig. 54 (Abstract P231).**
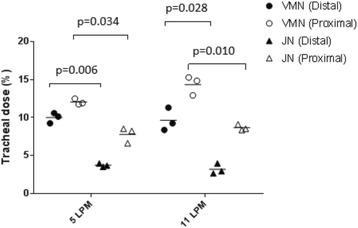
Comparison of the Aerosol delivery to lung filter (n = 3)

**Results:** The results of testing are presented in Fig. 54. Results are expressed as a percentage of the nominal dose recovered from the filter. The values used for Circuit A and B were reported in [5].

**Conclusions:** The largest aerosol dose delivered was at position B. Position A, C and D aerosol dose delivered were comparable. When the PH was placed between the Wye and the Nebulizer (position D), the delivered dose was unchanged when compared to position C (p-value between 0.298 - 0.488), suggesting that the inclusion of a PH has minimal effect on aerosol delivery.

**References**

1. Campbell, RS, et al.: Respir Care, 2000, Vol.45(3)

2. Wilkes, AR., et al.: Anaesthesia, 2000, Vol.55. 458-465

3. Turnbull, D., et al.: British Journal of Anaesthesia, 2005, Vol.94(5),675–82

4. Morgan-Hughes, NJ., et al.: British Journal of Anaesthesia, 2001, Vol.87(2)

5. Ari, A., et al.,: Respiratory care, 2010, Vol.55(7)

## P232 Evaluation of vibrating mesh and jet nebuliser performance at two different attachment setups in line with a humidifier nebuliser system

### A. O’Sullivan, P. Kelly, L. Sweeney, E. Mukeria, M. Wolny , R. MacLoughlin

#### Aerogen, Galway, Ireland

**Introduction:** This study investigated emitted and tracheal dose delivery for a vibrating mesh nebuliser (VMN) and jet nebuliser (JN) in line with a humidifier nebuliser system with two different nebuliser positions relative to the patient. Selection of nebuliser type can have a significant impact when treating a patient, previous studies have shown that the VMN delivers more aerosol than the conventional JN [1].

**Methods:** Aerosol delivery performance was evaluated by characterising the Emitted dose [drug that exits the mouthpiece or T-piece of a nebuliser] and Tracheal dose [drug delivered beyond the trachea]. A 2.0 mL dose of Salbutamol (1 mg/mL) was nebulised as a tracer aerosol using both a VMN (Aerogen Solo, Aerogen, Ireland), and a JN (Cirrus 2, Intersurgical). Both nebulisers were put in-line with the humidifier nebuliser (Aquamist, Intersurgical) system. Two nebuliser positions (distal and proximal to the patient) were assessed for their effect on both emitted and tracheal dose delivery at supplemental gas flow rates of 5 and 11 LPM (n = 3). For tracheal dose the nebuliser was connected to the head model via facemask. The head model was connected to a breathing simulator (BPM 15, Vt 500 mL, I:E 1:1,) and drug collected on an absolute filter (RespirGard II 303, Baxter). The drug was extracted and quantified using UV spectrophotometry. Finally, a paired t-test analysis was carried out to evaluate the different test setups.

**Results:** Based on the results presented, the VMN delivered the highest emitted dose (70.49 ± 1.86 %, distal at 5 LPM), (67.84 ± 0.98 %, distal at 11 LPM) and (101.03 ± 5.45 %, proximal at 5 LPM), (107.40 ± 5.51 %, proximal at 11 LPM) at both attachment setups and supplemental gas flow rates compared with the JN (48.43 ± 6.92 %, distal at 5 LPM) & (47.94 ± 3.48 %, distal at 11 LPM) and (56.86 ± 1.12 %, proximal at 5 LPM), (53.48 ± 5.22 %, proximal at 11 LPM).

The VMN also delivered the highest tracheal dose (10.00 ± 0.67 %, distal at 5 LPM), (9.66 ± 1.51 %, distal at 11 LPM) and (12.06 ± 0.39 %, proximal at 5 LPM), (14.36 ± 1.25 %, proximal at 11 LPM) at both attachment setups and supplemental gas flow rates compared with the JN (3.73 ± 0.22 %, distal at 5 LPM) & (3.19 ± 0.69 %, distal at 11 LPM) and (7.79 ± 1.03 %, proximal at 5 LPM), (8.68 ± 0.39 %, proximal at 11 LPM).

**Conclusions:** Results demonstrate that the VMN delivered significantly larger fractions of aerosol (P-values <0.05) for both emitted and tracheal dose at both positions in the humidifier nebuliser patient circuit.

**Reference**

(1) Ari A et al. Respir Care 55(7):845–851

**Fig. 55 (Abstract P232):**
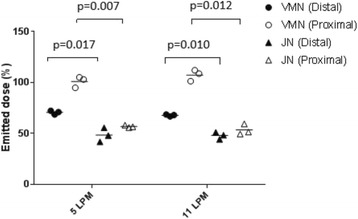
Emitted dose (%). (P-values included from paired t-test analysis

**Fig. 56 (Abstract P232):**
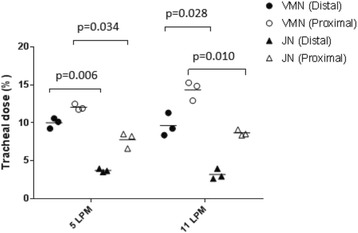
Tracheal dose (%). (P-values included from paired t-test analysis)

## P233 Psv-niv versus cpap in the treatment of acute cardiogenic pulmonary edema

### A. Pagano^1^, F. Numis^2^, G. Visone^3^, L. Saldamarco^3^, T. Russo^3^, G. Porta^2^, F. Paladino^3^

#### ^1^Cardarelli Hospital, Naples, Italy; ^2^San Paolo Hospital, Naples, Italy; ^3^Cardarelli Hospital, Naples, Italy

**Introductions:** Acute cardiogenic pulmonary edema (ACPE) is a common cause of acute respiratory failure. Non invasive continuous airway positive pressure (CPAP) has been validated as effective treatment in addition to pharmacological therapy. The aim of study is to compare non invasive pressure support ventilation (PSV-NIV) and CPAP in this setting of patients.

**Methods:** From 1 september 2015 to 15 november 2015, 24 patients were admitted to emergency department (ED) for ACPE. All patients were treated with standard medical therapy. In addition, 12 patients were treated with CPAP (group A) and 12 were treated with NIV-PSV (group B). Arterial hemogasanalysis was performed at admission (t0) and after 30 minutes from the beginning of ventilation treatment (t30′) to evaluate pH, pO2/FiO2, pCO2 and lactate cleareance (lactate t0-lactate t30′/lactate t0).

**Results:** We enrolled 24 patients (10 men and 14 women). The median (o average) age was 74 years. Baseline characteristics of patientes were: sistolic blood pressure 180 mmhg, diastolic blood pressure 102 mmhg, pO2/FiO2 181, Ph 7,21, pCO2 53 mmhg, lactate 3,4 meq/l. After 30 minutes of CPAP, we noticed in group A patients an improvement of 52 % in pO2/FiO2 ratio, an increase of 0,02 points in pH value, a reduction of 10 % in pCO2, a lactate cleareance of 28 %, in group B an improvement of 120 % in pO2/FiO2 ratio, an increase of 0,13 points in pH value, a reduction of 20%in pCO2, a lactate cleareance of 35 %. No patients required endotracheal intubation. Two patients in A group, after 30 minutes of CPAP, shifted in PSV-NIV because of increase of pCO2.

**Conclusions:** The preliminary results of our ongoing trial have shown a better outcome at t30′ in patients affected by ACPE treated with PSV-NIV compared to those treated with CPAP in lactate clearance, improvement of pO2/FiO2 ratio, pH and pCO2 value. No differences in side effects between two groups were shown.

**References**

1 Park. Randomized, prospective trial of oxygen, continuous positive airway pressure, and bilevel positive airway pressure by face mask in acute cardiogenic pulmonary edema. Crit Care Med 2004; 12 2407-2415

2 Scot. Two-Hour Lactate Clearance Predicts Negative Outcome in Patients with Cardiorespiratory Insufficiency. Critical Care Research and Practice

## P234 Noninvasive ventilation in patients with haematologic malignancy: a retrospective review

### C. Bell^1^, J. Liu^2^, J. Debacker^2^, C. Lee^2^, E. Tamberg^2^, V. Campbell^2^, S. Mehta^2^

#### ^1^National University of Ireland, Galway, Galway City, Ireland; ^2^Mount Sinai Hospital, Toronto, Canada

**Introduction:** Non-invasive ventilation (NIV) is commonly used as a first line therapy for immunocompromised patients with acute respiratory failure, although it may not be appropriate for every patient. Failure of NIV is an independent predictor of mortality and delayed endotracheal intubation may worsen prognosis. We report our center’s experience and outcomes for patients with active haematologic malignancy treated with NIV.

**Methods:** We conducted a retrospective chart review of consecutive patients with haematologic malignancy admitted to Mount Sinai Hospital ICU for acute respiratory failure between January 1, 2010 and May 31, 2015, and were initially treated with NIV (BiPAP or CPAP). We compared characteristics of patients who were successfully treated with NIV and avoided endotracheal intubation (ETI), and those who failed NIV.

**Results:** 79 patients (32 females, 56 ± 14 yrs, APACHE II 27.6 ± 4.6) with haematologic malignancy were treated with NIV for acute respiratory failure. 76 % of patients had acute leukaemia, 10 % had lymphoma, and 14 % had chronic leukaemia, multiple myeloma or myelodysplastic syndrome. Aetiology of respiratory failure was multifactorial in 39 % patients, with features of pneumonia in 77 % patients, severe sepsis or septic shock in 42 %, and pulmonary oedema in 30 %. 57 % of patients failed NIV and required ETI, 8 % had a do-not-intubate (DNI) order and died following NIV failure, and 35 % avoided ETI. Compared with patients who avoided ETI, those who failed NIV were more likely to have acute leukaemia (84 % vs 61 %, p = 0.019) and at baseline had higher PaCO2 (39 vs 30, p = 0.038), higher FIO2 (0.6 vs 0.4, p = 0.002), and more vasopressor use (31 % vs 11 %, p = 0.059). Only one patient had a diagnostic bronchoscopy while treated with NIV and 25 (55 %) had bronchoscopy within 1 day following ETI. Of the 44 patients who failed NIV and required ETI, (68 %) died in the ICU. Overall ICU mortality of this cohort was 41 % and 3-month mortality was 57 %.

**Conclusions:** Two thirds of patients with haematologic malignancy and acute respiratory failure required ETI following NIV failure and had a high subsequent mortality. Patients who fail NIV have higher PaCO2, higher FiO2 and more vasopressor use. Use of NIV may be associated with delayed diagnostic bronchoscopy.

## P235 Use of non-invasive ventilation in infectious diseases besides classical indications

### A. Silva-Pinto, A. Sarmento, L. Santos

#### Centro Hospitalar São João, Porto, Portugal

**Introduction:** Non-invasive ventilation (NIV) is used with well-defined scientific evidence in acutely decompensated chronic pulmonary disease and acute pulmonary edema. NIV has been also successful in immunosuppressed patients with pulmonary infiltrates, especially in hematological and pharmacological immunosuppressed patients. The use of NIV remains controversial in pneumonia, tuberculosis, flu, acute respiratory distress syndrome (ARDS)… The aim of this study is to analyse the use of NIV in an Infectious Diseases Intensive/Intermediate Care Unit (ID-ICU).

**Methods:** We performed a cross-sectional study of adult patients needing NIV, admitted to the ID-ICU between 2010 and 2014. We excluded patients who need NIV with chronic pulmonary disease, acute pulmonary edema or during weaning of mechanic ventilation. The patients were identified by our hospital database and subsequently the clinical records were consulted. The primary outcome was the result of NIV and secondary outcomes were death and its predictors. We used the most appropriate measure of central tendency and distribution and statistical test (significance level 0.05).

**Results:** We included 50 patients (34 male) with a median age of 52. The majority (27) were immunosuppressed: 21 patients had human immunodeficiency virus (HIV) infection with CD4 count less than 350/mm3, 3 had hematological neoplasia under chemotherapy, 2 were under immunosuppressant drugs, and 2 had solid neoplasia under chemotherapy. The median APACHE II score was 19 and SAPSII score was 43. Eight patients had a “do not intubate” status due to their comorbidities. The most frequent reason to perform NIV was bacterial pneumonia (44 %) followed by pneumocystosis (30 %), ARDS (16 %), flu (6 %) and tuberculosis (2 %). Median time of NIV was 3,5 days. We had a NIV success rate of 60 % (14 patients were intubated, 5 did not have intubation indication and 1 patient died from another cause). The mortality rate was 28 %. We found statistically significant association between the severity scores SAPSII and APACHE and the NIV result and death (higher scores were associated with failure of NIV and death). We also found that patients with hematological neoplasia under chemotherapy had higher rates of failure of NIV and death. HIV infection was associated with death but not with NIV failure.

**Conclusions:** The use of NIV is nowadays more frequent apart from its classical indications. We found that higher severity scores and hematological neoplasia under chemotherapy were associated with unsuccessful NIV. However NIV can be used in these patients as a comfort measure.

## P236 The impact of fragility on noninvasive mechanical ventilation application and results in the ICU

### Ý. Kara^1^, F. Yýldýrým^1^, A. Zerman^2^, Z. Güllü^2^, N. Boyacý^2^, B. Basarýk Aydogan^1^, Ü. Gaygýsýz^1^, K. Gönderen^1^, G. Arýk^3^, M. Turkoglu^2^, M. Aydogdu^1^, G. Aygencel^2^, Z. Ülger^3^, G. Gursel^1^

#### ^1^Gazi University School of Medicine Respiratory Medicine and Critical Care Department, Ankara, Turkey; ^2^Gazi University School of Medicine, Internal Medicine Critical Care Department, Ankara, Turkey; ^3^Gazi University School of Medicine, Geriatrics Department, Ankara, Turkey

**Introduction:** Many factors affecting noninvasive ventilation (NIV) success had been investigated up to now but there is no any data about the affect of fragility on NIV outcomes in critically ill patients. In this prospective and observational study, we aimed to investigate the effect of fragility on the application and results of NIV in the intensive care unit (ICU).

**Methods:** We included in the patients over 50 years old who were admitted to Medical ICUs of our hospital. For detection of patients fragility Clinical Fragility Score (CFS) and comprehensive geriatric assesment were used. Patients who have CFS > =5 were considered as fragile. The successful NIV group (NSG) is defined as achieving success in at least two of the followings: PaO2 > 60 mmHg, PaCO2 < 50 mmHg, pH = 7,35-7,45, improvement of respiratory effort, recovery of consciousness. The relationship between entubation and fragility was also assessed. Application problems were defined as; cooperation problems, hearing problems, delirium/agitation (Richmond agitation sedation scale > 1) , claustrophobia, alzheimer, problems related with chin, mouth tooth and leak (>35 L/min).

**Results:** We included in 85 patients who were underwent to NIV in two ICUs. Mean age of patients was 72,5 ± 10,6, 60 % of them were male and mean APACHE II score was 20,6 ± 5,7 (8-34). Mean CFS of the patients was 4,6 ± 1,6 and 42 % of them were fragile (CFS > =5). There were 63 (75 %) patients in NSG and 22 (25 %) in NIV failure group (NFG). APACHE II and SOFA scores of NFG were higher and NSG had more non-fragile patients (CFS < 5) (41 vs 22, p = 0.011). Fifty-three (62,4 %) patients had NIV application problems and mean CFS of this group was higher (4,9 ± 1,6 vs 4,0 ± 1,7, p = 0,028).

Using NIV previously at home (p = 0,025), APACHE II (p = 0,001), SOFA (P = 0,012) were detected as factors affecting the NIV success in univariate analysis. While fragile patients(CFS > =5) had significantly higher application problems(80 % vs 57 % p:0,038), entubation (31 % vs 8 %, p:0,007) and mortality rates (30 % vs 4 %, p:0,001), significantly lower NIV succes rates (61 % vs 85 %, p: 0,011). In logistic regression analyses fragility was only independent risk factor for NIV failure (OR:3.5, p:0,02).

**Conclusions:** These results showed that fragility is associated with NIV application problems and failure in the ICU and increases NIV failure risk more than 3 times.

## P237 Effects of metabolic alkalosis on noninvasive ventilation success and ICU outcome in patients with hypercapnic respiratory failure

### N. Boyacý, Z. Isýkdogan, Ö. Özdedeoglu, Z. Güllü, M. Badoglu, U. Gaygýsýz, M. Aydogdu, G. Gursel

#### Gazi University School of Medicine Respiratory Medicine and Critical Care Department, Ankara, Turkey

**Introduction:** Metabolic alkalosis (MA) represents up to half of all acid-base disturbances in hospitalized patients and it is important to know that if it is interact with the noninvasive ventilation (NIV) therapy in hypercapnic respiratory failure. Because of the frequent diuretic and steroid usage lots of hypercapnic patients with COPD and heart failure requiring NIV are also at risk for MA and it may cause hypoventilation. While many reasons of NIV failure have been studied and defined extensively MA as a reason of NIV failure has not been investigated in hypercapnic respiratory failure yet. The aim of this study is to assess if MA influence NIV success and intensive care unit (ICU) outcome in patients with hypercapnic respiratory failure and receiving NIV therapy.

**Methods:** Data of 206 patients with hypercapnic respiratory failure and treated with NIV screened retrospectively. Patients who received asetozolamid during the NIV therapy excluded from the study. We investigated frequency, risk factors for the development of MA and if MA effects PaCO2 reduction rate, NIV, ICU and hospital stay and mortality.

**Results:** Sixty one of 206 (30 %) patients developed MA at 3rd day and this group were older, more obese, had heart failure and used loop diuretics more often than the other group Lenght of NIV, ICU, hospital stay were longer in MA group than the non-MA group (p:0.024, 0.006, 0.001 respectively). Arterial blood gas values at the day of MA was pH: 7.45 PaO2: 71 mmHg, PaCO2: 58 mmHg HCO3:41 meq. Admission diagnosis of heart failure increased development of MA risk 3.4 times (OR:3,4 (CI 95 %:1,6-7) p:0,001). 190 mg of furosemid dose determined as cutt of point for the development of MA (AUC = 0,782 p < 0,001, sensitivity 68 %, specifity 80 %). Duration of NIV was significantly longer in patients with MA but it was independent risk factor for only hospital stay longer than 10 days (OR:3.4, CI95%:1,7-6,8), p < 0.0001).

**Conclusions:** MA may be seen as high as 30 % in patients with hypercapnic respiratory failure and requiring NIV and may cause to increase in risk of longer hospital stay in these patients .

## P238 Asynchrony index and breathing patterns of acute exacerbation copd patients assisted with noninvasive pressure support ventilation and neurally adjusted ventilatory assist

### N. Kongpolprom , C. Sittipunt

#### Chulalongkorn University, Bangkok, Thailand

**Introduction:** Noninvasive ventilation(NIV) is practically used for patients with acute exacerbation of COPD(AECOPD) with acute respiratory failure(ARF). Several new modes of NIV are currently provided for ventilatory assistance, however it is not known what the best one is. Theoretically, neurally adjusted ventilatory assist(NAVA) improves asynchrony and preserves natural breathing [1], which might benefit to dynamic hyperinflation.

**Methods:** A pilot crossover study comparing asynchrony index and breathing patterns between AECOPD patients assisted with NIV-PS and those assisted with NIV-NAVA was conducted. AECOPD patients with ARF using NIV-PS were recruited. The clinically stable patients were randomly ventilated with NIV-PS or NIV-NAVA for 30 minutes, and then switched to the other mode after a wash-out period. One patient could be enrolled more than 1 comparative sequences. Breathing patterns, 6 types of asynchrony and electrical diaphragmatic activity(EAdi) were continuously recorded during the 20 last-minute periods. Continuous data were reported as medians (IQR) or means (SD) and compared by using unpaired t-test or Mann-Whitney U test.

**Results:** Three AECOPD patients were allocated to seven comparative sequences. NIV-NAVA tended to decrease % asynchrony index: 12.88(3.33, 21.95)% in PS VS 2.62(0.85, 11.57)% in NAVA, p = 0.13. NIV-NAVA significantly reduced trigger-delay(Td) : Td in PS 9 (6.75, 20.75) ms VS Td in NAVA 3 (1,6.5) ms, p = 0.017,and improved ineffective triggering: 1.03(1.8) events/minute in PS VS 0(0) event/minute in NAVA, p = 0.026. Moreover, assistance with NIV-NAVA tended to deliver lower tidal volume(Vt) with higher Vt variability: mean Vt 8.91(2.7) ml/Kg with CV 28.42 during PS VS mean Vt 6.81(1.85) ml/Kg with CV 38.45 during NAVA. In contrast, the similarity of respiratory rate (RR) and EAdi variability was observed: RR 21.44(2.75) /minute with CV13.25 in PS VS 22.7(4.11) /minute with CV10.04 in NAVA and EAdi 22.17(14.42) μV. with CV 46.40 in PS VS 17.02(11.02) μV. with CV 45.87 in NAVA, respectively.

**Conclusions:** Compared with NIV-PS, NIV-NAVA attenuated asynchrony, especially ineffective triggering, in AECOPD patients. It also promoted physiologic breathing pattern, including matched neural triggering and low Vt with high variability.

**Reference**

1. Terzi N, Piquilloud L, Roze H, Mercat A, Lofaso F, Delisle S, et al. Clinical review: Update on neurally adjusted ventilatory assist- report of a round-table conference. Critical care 2012;16(3):225.

## P239 High frequency jet ventilation for severe acute hypoxemia

### A. Eden, Y. Kokhanovsky, S. Bursztein – De Myttenaere, R. Pizov

#### Carmel, Lady Davis Medical Center, Haifa , Israel

**Introduction:** High frequency ventilation has theoretical advantages for the management of ARDS, however, high frequency oscillatory ventilation (HFOV) did not improve outcome [1]. High frequency jet ventilation (HFJV), unlike HFOV allows for spontaneous ventilatory efforts. We report our experience with HFJV in patients with severe hypoxemia over a 10 year period.

**Methods:** HFJV (Monsoon, Acutronic Medical Systems, Hirzel, Switzerland) was superimposed upon low rate (6/min) low volume (100-200 ml) SIMV. Patients who required FiO[sub]2[/sub] > 0.5 and PEEP > 10 cmH[sub]2[/sub]O or those with bronchopleural fistula were candidates for HFJV. Patients were excluded if air trapping was evident on expiratory flow curve or the machine was unavailable (1 unit only). In all patients intratracheal mean airway pressure (MAWP) was measured continuously. Parameters were compared over time using ANOVA (p < 0.01 significant).

**Fig. 57 (Abstract P239).**
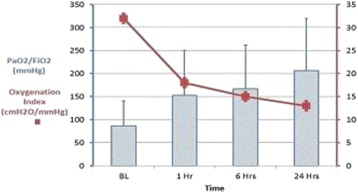
ᅟ

**Results:** 47 patients received HFJV (35 males, age 58 ± 18, BMI 26.4 ± 4.2, APACHE II 24.4 ± 8.8). Baseline (BL) Vt, PEEP and peak pressure were 7.4 ± 1.5 ml/kgpbw, 12 ± 3 and 32 ± 6 cmH[sub]2[/sub]O Respectively. At BL 8 were in prone position and 10 received NO. During HFJV 3 remained in prone position and 8 received NO. One patient was converted to ECMO and 2 patients were weaned from ECMO to HFJV (data not shown). Over the first 24 hours of HFJV oxygenation improved significantly at all time points (Fig. 57) while PaCO[sub]2[/sub] decreased from 52 ± 11 to 41 ± 10 (P < 0.001). pH increased (7.30 ± 0.1 to 7.40 ± 0.1, P < 0.001) while MAWP (22 ± 5 to 19 ± 5 cmH[sub]2[/sub]O P = 0.03) and lactate (1.9 ± 1.2 to 1.8 ± 1.0 mM/L, p = 0.6) did not change. During the first 24 hours oxygenation improved in 41 patients. HFJV was continued for a median of 5 (IQR 2,7) days. 30 of the patients survived to hospital discharge.

**Conclusions:** This report suggests that HFJV improves oxygenation without increasing airway pressures, reducing ventilation, or affecting the general condition as manifested by lactate levels. Further, prospective trials are required.

**Reference**

1. Ferguson ND et al. N Engl J Med 368:795-805,2013

## P240 HFOV revisited: a 7 year retrospective analysis of patients receiving HFOV who met oscillate trial entry criteria

### L. Neilans, N. MacIntyre

#### Duke University, Durham, USA

**Introduction:** High Frequency Oscillatory Ventilation (HFOV) offers attractive lung protective features by providing mean airway pressures but minimal tidal alveolar pressure/volume swings. Small trials have suggested benefit to HFOV but two recent large trials (OSCAR and OSCILLATE) suggested either no benefit or even harm [1,2]. Questions of generalizability have been raised. In OSCILLATE, enrolled subjects were often in shock and mandated unusually high mean airway pressures. This combination can be difficult to manage in centers with variable expertise in HFOV management. Because our institution has considerable HFOV experience, we retrospectively reviewed all of the patients who received HFOV with us over the last 7 years.

**Methods:** We evaluated 219 patients who received HFOV from 2007-2014 at Duke University. Forty-six ARDS patients met the OSCILLATE inclusion criteria and data was collected via retrospective chart review.

**Results:** Our results compared with OSCILLATE and OSCAR results are summarized in the Table 32.

**Conclusions:** Despite limitations to our study (retrospective, observational and with limited data availability), we think our lower HFOV mortality despite worse baseline oxygenation when compared to OSCILLATE and OSCAR is notable. This may reflect lower HFOV mean airway pressures in shock patients as well as having experienced personnel available 24 hours a day in our institution. We conclude there may still be a role for HFOV in severe ARDS if patients are properly selected and careful attention is paid to mean airway pressures.

**References**

1. Young D et al. N Engl J Med 2013;368:806-813

2. Ferguson ND, et al N Engl J Med 2013;368:795-805.

**Table 32 (Abstract P240).** ᅟ

Parameter	Current Review	OSCILLATE	OSCAR
Number of Subjects	46	275	398
Age	50 +/- 12	55 +/-16	55 +/-19
Gender (%Male sex)	61	61	64
Pre HFOV PaO2/FiO2	77 +/- 25	121 +/- 46	113 +/-37
HFOV mean Paw (cm H20) day 1	28 +/-2.1	31 +/-2.6	26.9 +/- 6.2
Apache II	24.1 +/- 5.9	29 +/- 8	21.8 +/- 6.0
Mortality (%)	39	47	41.7

## P241 Implementation of a goal-directed mechanical ventilation order set driven by respiratory therapists can improve compliance with best practices for mechanical ventilation

### M. Radosevich, B. Wanta, V. Weber, T. Meyer, N. Smischney, D. Brown, D. Diedrich

#### Mayo Clinic, Rochester, USA

**Introduction:** Multiple studies have shown that low tidal volume, lung protective ventilation strategies may reduce pulmonary complications in non-ARDS ICU patients. In busy ICUs, discrepancies may arise between ventilator management goals and actual practice. We developed a goal-directed mechanical ventilation order set that included physician-specified lung-protective ventilation and oxygenation goals to be implemented by our respiratory therapists (RTs). Our primary outcomes were to determine if a simple respiratory therapist-driven order set with predefined goals regarding oxygenation and ventilation could be implemented with good compliance and to determine compliance with the ARDSNet PEEP/FiO2 table. As secondary outcomes, we evaluated duration of mechanical ventilation, ICU length of stay, and both in-hospital and ICU mortality.

**Methods:** We evaluated 1302 patients undergoing invasive mechanical ventilation prior to and after institution of a standardized, goal-directed mechanical ventilation order set. Specific goals for oxygenation (SpO2, PaO2, PEEP/FiO2 table use) and ventilation (pH, PaCO2) were selected by providers and were subsequently implemented by our RTs as they saw fit.

**Results:** A total of 1693 unique episodes of invasive mechanical ventilation and 1302 individual patients were analyzed. Compliance with the new mechanical ventilation order set was found to be high with a mean of 86.9 % of ventilator settings compliant with orders. The difference in order compliance before and after implementation was statistically significant (mean 1.3 % vs. 86.9 %, p <0.0001). Compliance with the ARDSNet PEEP/FiO2 table after implementation of the order set was significantly greater (median of 82.9 % vs. 86 %, p = 0.02). There was no difference in duration of mechanical ventilation, ICU length of stay, in-hospital or ICU mortality.

**Conclusions:** A standardized mechanical ventilation order set with physician-specified oxygenation and ventilation goals to be implemented by RTs who routinely practice lung-protective ventilation can be implemented with good compliance and improve adherence to the ARDSNet PEEP/FiO2 table.

## P242 A reduction in tidal volumes for ventilated patients on ICU calculated from IBW. can it minimise mortality in comparison to traditional strategies?

### A. Fuller^1^, P. McLindon^2^, K. Sim^1^

#### ^1^St Helens and Knoowsley, Prescot, UK; ^2^Royal Infirmary , Edinburgh, UK

**Introductions:** Over 60 % of patients admitted to an Intensive Care Unit (ICU) will require Endotracheal Tube (ETT) intubation and mechanical ventilation[1]. The aim was to perform an audit of ventilation regimes in critically ill patients admitted to Whiston Hospital. Its main objectives were to evaluate the current management of ventilated patients on ICU at Whiston hospital in comparison to the current local standards of best practice, with the main emphasis surrounding IBW (Ideal Body Weight) documentation and reduction in initial tidal volume (TV) from traditional guidelines.

**Methods:** A retrospective study looking at a sample cohort of 182 patients admitted to the ICU at Whiston from January 2014 to the end of June 2014 who was managed with either Pressure Regulated Volume Control (PRVC) or Synchronised Intermittent Mandatory Ventilation (SIMV). Of those 182 candidates, 107 matched the ventilation inclusion criteria. The data sources used were under the ICU subcategory on the trust electronic data management system and ICU electronic charting system. A data collection proforma was completed for each patient.

**Results:** All patients initiated on PRVC or SIMV should have their anthropometric measurement of ulnar length or actual height recorded. It was found only 35.5 % of patients admitted had these recorded as opposed to the local guideline of 100 %. 50 % had documented IBW with 16.9 % of those not having a recorded optimal TV based on 6 ml/kg. Of the 45 patients who had documented IBW appropriate TV calculated, 62 % were commenced on TV greater than their calculated optimal TV. Every patient’s arterial oxygen saturations were to be maintained between 88-92 %. Only 10.3 % of patients met this standard, although 95.4 % of patients maintained saturations greater than 88 %.

**Conclusions:** To summarise it is clear that documentation relating to ventilation care is lacking despite a clear section outlined on the ICU admission proforma. A proposal to resolve this issue would be to use a laminated sheet including patient’s ulnar length/actual height, IBW and optimal TV.

**Reference**

Colledge N, Walker B, Ralston S. Davidson’s Principles & Practice of Medicine. Critical Illness; Management of Organ Failure. 21st Edition. Pages 192-198. Churchill Livingstone. Date Accessed 26/11/2015.

## P243 Predictive value of lung aeration scoring using lung ultrasound in weaning failure

### M. Shoaeir^1^, K. Noeam^1^, A. Mahrous^1^, R. Matsa^2^, A. Ali^1^

#### ^1^Alexandria Universitry General Hospital , Alexandria, Egypt; ^2^ University Hospital North Middlands, Stoke On Trent, UK

**Introduction:** Optimal time to wean from mechanical ventilation (MV) remains a challenge in most critically ill patients [1]. There have been studies looking at the factors that aid weaning from MV, however the results have been variable. Recent evidence suggests lung ultrasound (LUS) can predict successful weaning from MV [2]. This study assesses the ability of the lung aeration scoring [3] using LUS to predict successful weaning in critically ill patients.

**Methods:** This prospective, observational and non-interventional study was performed in a general ICU in a large university hospital over a period of 6 months. We included all patients who needed MV for > 48 hours (n = 50), and were eligible for Spontaneous Breathing Trial (SBT). LUS was performed during SBT and lung aeration scoring was calculated in 12 lung regions. Points were allocated according to the worst ultrasound pattern observed: N (normal lung pattern) = 0, B1 lines ( multiple well-defined B lines) =1, B2 lines (multiple coalescent B lines) = 2, c (consolidation or atelectasis) = 3.

Relevant data were collected from patients, including demographic and LUS aeration scoring, in a standardized case report form (Table 33). Patients were divided into two groups: Group F and Group S based on the failure and success in weaning, respectively. The LUS aeration scoring was applied to both groups to assess the predictability of weaning failure.

**Results:** Lung aeration scores were significantly higher in patients in Group F compared to Group S (p < 0.01). The sensitivity, specificity for failed extubation for a LUS score of > 18 is 88.89 and 86.96 respectively.

**Conclusions:** Lung aeration score using lung ultrasound can predict weaning failure in critically ill patients. Our study showed that a LUS score of >18 has a good prediction value. However larger randomised controlled trials are required to validate this pilot study.

**References**

1. Conti G et al.: Intensive Care Med 2004; 30:830–36.

2. Soummer A et al.: Critical Care Med 2012; 40:2064–72.

3. Bouhemad B et al.: Critical Care Med 2012; 38(1): 84-92.

**Table 33 (Abstract P243).** Patients’ characteristics

Patients’ characteristics	Group F (n = 27)	Group S (n = 23)	P value
Male: Female in %	51.9:48.1	60.9:39.1	0.52
Age	51.89 ± 14.58	47.52 ± 14.60	0.29
LUS score	20.47 ± 2.75	12.52 ± 4.61	<0.05

## P244 Conventional versus automated weaning from mechanical ventilation using SmartCare™

### C. Dridi, S. Koubaji, S. Kamoun, F. Haddad, A. Ben Souissi, B. Laribi, A. Riahi, M. S. Mebazaa

#### Mongi Slim University Hospital, La Marsa, Tunisia

**Introduction:** Clinicians are routinely faced with the challenges of managing the ventilatory care and weaning process during the clinical course of the illness. Almost 40 % of mechanical ventilation time is spent on weaning [1] and in the last decade, automatic ventilator control and ventilation modes were designed to help weaning from mechanical ventilation. In many studies automated weaning significantly decreased weaning time in critically ill patients when compared with physician controlled weaning process [2,3]. We conducted a prospective randomized trial comparing automated weaning with a standardized weaning protocol in a multidisciplinary ICU.

**Methods:** From April 2014 to Mars 2015, we enrolled critically ill adults requiring more than 48 hours of mechanical ventilation. Patients were randomized since admission considering availability of SmartCare equipped ventilators (8 Evita XL in our unit: 4 with SmartCare and 4 without). We included patients who tolerated at least 24 hours of pressure support with PEP equal or less than 5 cmH2O and were not ready to undergo a spontaneous breathing trial, they were assigned to be weaned either by SmartCare (SC Group) or by the physician controlled weaning according to local guidelines (PCW Group). Weaning duration, total duration of mechanical ventilation and the success of weaning were the primary endpoints.

**Results:** Forty-four patients were enrolled, 22 in each group. There was no difference between the two groups concerning age, sex, neurological or respiratory history, diagnosis or severity at study entry. Weaning duration and total mechanical ventilation were similar between the two groups and were respectively 6 +/- 7 days in PCW Group vs 5 +/- 4 days in SC Group (p = 0.63) and 11 [3-37] days vs 10 [3-23] days in SC Group. In the PCW Group, we noted only one extubation failure and there were no reintubation in SC Group.

**Conclusions:** Although automated weaning using SmartCare did not show significant benefit for patients concerning duration of mechanical ventilation and duration of weaning when compared to physician-controlled weaning according to local protocols; it has certainly offer a gain of time for the medical staff in an ICU where there is no respiratory physiotherapist neither critical care specialty nurses.

**References**

1. Esteban A. et al.: N Engl J Med. 1995; 332:345-50.

2. Karen E. et al.: Am J Respir Crit Care Med. 2013; 187:1203-11.

3. Burns KE. et al.: Cochrane Database Syst Rev. 2014; 9:CD008638.

## P245 Ultrasonographic evaluation protocol for weaning from mechanichal ventilation

### A. Pérez-Calatayud^1^, R. Carrillo-Esper^1^, A. Zepeda-Mendoza^1^, M. Diaz-Carrillo ^1^, E. Arch-Tirado^2^

#### ^1^Medica Sur, Mexico City, Mexico; ^2^Instituto Nacional de Rehabilitacion, Mexico City, Mexico

**Introduction:** Mechanical ventilation (MV) is a therapy for vital support used in a significant proportion of critically ill patients. The right time to succesfully discontinue this therapy is a challenge for the intensivist The ecocardiographic evaluation of the diastolic dysfunction, the diaphragm and the lung have become an invaluable tool for weaning from MV protocols, especially in pacients with dificult or prolong weaning from MV. We propose a mathematichal model of an ultrasound protocol for weaning from MV that integrates the three modalities

**Methods:** Based on current literature, we develop a score justified by a mathematical model based on inequations

**Results:** When the risk of failure in the weaning process rises, for these reason the weaning process should be suspended. When the risk of failure is low and the weaning process should be continued.

**Conclusions:** The use of math models for decision making is of great importance, it sets an objective parameter within the existing evaluations. We proposed the use of inequations, to set intervals of solution with the three points of care for ultrasound guided weaning from MV. With this the inequations propose generate an area of certainty within the proposed values an the solution intervals.

**References**

1. Lamia B, et al. Crit Care Med. 2009; 37:1696-1701.

2. Matamis D, et al. Intensive Care Med. 2013;39:801-810.

3. Soummer A, et al. Crit Care Med. 2012 ;40:2064-2072.

**Fig. 58 (Abstract P245).**
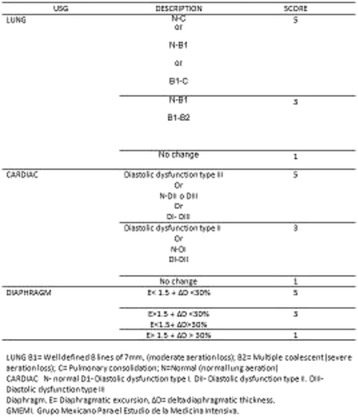
GMEMI SCORE

**Fig. 59 (Abstract P245).**
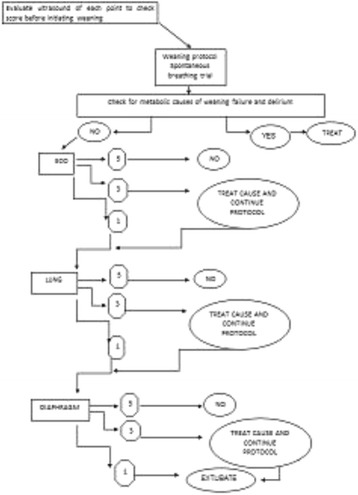
Algorithm based in GMEMI score

## P246 Diaphragm ultrasonography: a method for weaning patients from mechanical ventilation

### S. Carbognin^1^, L. Pelacani^2^, F. Zannoni^1^, A. Agnoli^2^, G. Gagliardi^2^

#### ^1^University of Padua, Padua, Italy; ^2^Sant Antony hospital, Padua, Italy

**Introduction:** Our aim was to assess diaphragm ultrasonography as a tool to predict the outcome of weaning.

**Methods:** We enrolled 5 intubated patients who were admitted to our Intensive Care Unit at the Saint Antony Hospital of Padua; a Nutrivent nasogastric tube was placed in each to measure the diaphragm contractility as the transdiaphragmatic pressure (ÄPdi) with the following formula: ÄPdi = gastric pressure-esophageal pressure. We performed a diaphragmatic sonography with the M-mode technique. We calculated the diaphragm contraction speed as the slope (Scdi) of the curve provided by the diaphragm contraction during the inspiratory phase of the spontaneous breathing trials. The correlation between the mean ÄPdi and the mean Scdi was evaluated using the Pearson’s method according with a linear regression analysis.

**Fig. 60 (Abstract P246).**
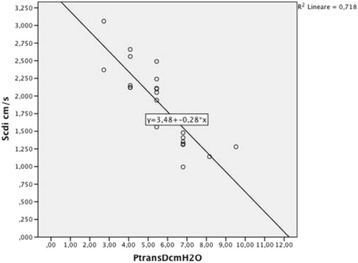
Relationship between ÄPdi and Scdi

**Results:** We found a significant correlation between the ÄPdi and the Scdi with a Pearson coefficient ñ = -0.851 and a linear determination index R = 0.718 as shown in Fig. 60.

**Conclusions:** the Scdi calculated during trials of spontaneous breathing represented a bedside, standardized and reproducible tool to predict the outcome of weaning.

**References**

1. Eskandar N et al. Critical care clinics 23(2): 263-74; 2007.

2. Powers SK et al. Critical care medicine 37(10 Suppl): S347; 2009.

3. Boles J-M et al. European Respiratory Journal 29(5): 1033-56; 2007.

4. Kim WY et al. Critical care medicine 39(12): 2627-30; 2011.

## P247 Dorsal diaphragmatic excursion tracks transpulmonary pressure in ventilated ARDS patients: a potential non-invasive indicator of lung recruitment?

### R. Cho^1^, A. Adams ^1^, S. Lunos^2^, S. Ambur^1^, R. Shapiro^1^, M. Prekker^1^

#### ^1^HCMC, Minneapolis, USA; ^2^University of Minnesota, Minneapolis, USA

**Introduction:** Optimizing positive end-expiratory pressure (PEEP) in acute respiratory distress syndrome (ARDS) patients to attain lung recruitment while avoiding over-distension is a clinical challenge. Methods of titrating PEEP to safely recruit the lungs have included achieving adequate SpO2, attaining best compliance, or increasing PEEP while monitoring esophageal manometry to establish a positive end-expiratory transpulmonary pressure (Ptp). Bedside ultrasonography is available and useful as a diagnostic tool in the care of critically ill patients. Preliminary work identified marked differences in excursions between the dorsal and ventral diaphragms during lung-protective tidal ventilation in ARDS patients. We hypothesize that lung recruitment in the supine patient may be detected non-invasively by increased dorsal diaphragmatic excursions, indicating that dependent lung regions may be recruiting with adequate PEEP.

**Methods:** In this proof-of-concept study, we enrolled 7 ARDS patients treated with invasive mechanical ventilation and neuromuscular blockade in the supine position. We measured Ptp using esophageal manometry and ventral/dorsal diaphragm excursions using anatomic M-mode ultrasonography as the applied PEEP was changed (set by the treating physician) as follows: +3,+6, -3, and -6 cmH2O. A standard lung history was established by recruitment maneuvers between each PEEP change, and the sequence of PEEP changes was randomized for each subject. We used linear mixed effects models to evaluate for an association between applied PEEP and Ptp and dorsal diaphragmatic excursion (DDE).

**Results:** Acceptable ultrasound images of diaphragm excursion were obtained in 6 of 7 patients; one patient had a large hepatic cyst precluding diaphragm ultrasound, data from this subject was excluded from the analysis. At enrollment, the mean(+/-SD) P/F ratio was 156+/-61 mmHg, mean baseline PEEP was 12.7+/-2.2 cmH2O, and the mean SpO2 was 94+/-2.2 %. Ventral diaphragmatic excursions were unchanged by PEEP. Increasing PEEP resulted in corresponding increases in Ptp and DDE (p = .0006, p = .005). The transition from a negative to positive Ptp with increasing PEEP occurred as DDE was observed to markedly increase.

**Conclusions:** This exploratory study suggests an association between Ptp and DDE as dorsal lung is recruited by increasing PEEP. If validated in a larger sample, ultrasound evaluation of dorsal diaphragmatic movement may be a non-invasive method for estimating lung recruitment in ARDS.

## P248 Pulse oximetry in the icu patient: is the perfusion index of any value?

### M. Thijssen, L. Janssen, N. Foudraine

#### Viecuri Medical Center, Venlo, Netherlands

**Introduction:** Pulse oximetry (SpO2) is intensively used as a surrogate for arterial oxygenation (SaO2) however, little is known about its accuracy in daily ICU practice. Disturbed perfusion may lead to a poor signal as indicated by a lower perfusion index value (PFI) and so compromise its accuracy. We studied SpO2 accuracy in its relationship to the PFI.

**Methods:** In a 5 month period, 281 patients were retrospectively studied resulting in 1281 concomitantly measured values of SpO2 (Philips M1191BL/M1194A), SaO2 and PFI. Inotropic use, pH, MAP, mechanical ventilation and body temperature were studied as independent variables. PFI values were categorized as low or poor with PFI < 1.0, intermediate or moderate with 1.0 < PFI < 2.5 and as reliable or high with PFI > 2.5. Data collection: All SaO2, SpO2 and PFI measurements during the first 3 days after admittance were collected unless the patient was invasively ventilated, then all measurements were collected until extubation. Statistical analysis: A linear correlation of the SpO2 and SaO2 values was assessed by Pearson’s method and the mean SpO2-SaO2 difference (Δ ) was assessed by using the Bland and Altman method.

**Fig. 61 (Abstract P248).**
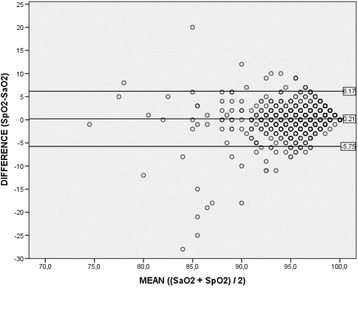
ᅟ

**Results:** Statistical analysis showed an overall correlation (r = 0.685; p < 0.01) between SaO2 and SpO2. The Bland and Altman analysis revealed that the limit of agreement within the general mean and ±2SD showed a Δ of ±6 % (Fig. 61). Of all values SpO2 overestimated the SaO2 value in 48.2 % of all cases and underestimated SaO2 in 31.4 %.

**Fig. 62 (Abstract P248).**
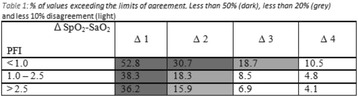
ᅟ

Over a wide range of PFI values (median 1.4, range 0.1-19.2) we found moderate agreement between SpO2 and SaO2 with slightly better agreement for higher PFI values (Fig. 62). Even with a PFI > 2.5, 15.9 % of all measurements showed a difference between SpO2 and SaO2 of more than ±2 %. The other independent variables showed only very weak associations (r < 0.2) with the Δ SpO2-SaO2, except for mechanical ventilation (ANOVA p = 0.002).

**Conclusions:** We found a clinical relevant lack of agreement between SpO2 and SaO2 measurements. In contrast to general expectations SpO2 exceeds SaO2 in 48.2 % of all measurements. PFI is of little value as differences may be great as even with a high (good) PFI value, 15.9 % of concurrent measurements of SpO2 and SaO2 differ > ±2 %. These findings may influence daily practice on how to adjust mechanical ventilation by SpO2 measurements.

## P249 Ventilation is a better assessment of respiratory status than EtCO2

### C. J. Voscopoulos^1^, J. Freeman^2^

#### ^1^University of Hawaii, John A. Burns School of Medicine, Honolulu, USA; ^2^Respiratory Motion Inc., Waltham, USA

**Introduction:** ICU patients require multiple monitoring inputs to optimize care & are subjected to many discomfort-causing interventions.. Direct measurement of respiratory effort in non-intubated patients is difficult, so clinicians rely on secondary indicators of respiratory sufficiency, such as pulse oximetry & capnography (EtCO2), which have significant limitations. Clinicians often resort to using respiratory rate (RR) measurements from the capnograph. We used a novel non-invasive respiratory volume monitor (RVM), which measures minute ventilation (MV), tidal volume (TV) & respiratory rate (RR), to assess the ability of EtCO2-generated RR to quantify respiratory status in non-intubated patients.

**Methods:** Continuous RVM & capnography data were collected from 50 subjects (age:46 ± 14 yrs) using an impedance-based RVM (ExSpiron) & capnograph (Capnostream 20) with a sampling oral/nasal cannula (Smart Capnoline Plus). Each subject performed 6 2.5-min breathing trials at various RRs. The correlations between EtCO2 measurements (low: <35 mmHg, normal: 35-45 mmHg, high: >35 mmHg), capnography-based RR (low: <6 b/min, adequate: > = 6 b/min), & RVM-based MV (low: <2 L/min, adequate: > = 2 L/min) were evaluated.

**Fig. 63 (Abstract P249).**
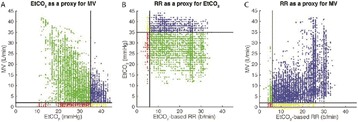
ᅟ

**Results:** Comparison of MV & EtCO2 measurements revealed that in only 24.6 % of the 9324 analyzed epochs did adequate MV coincide with normal EtCO2. 68.7 % of the time adequate MV coincided with low EtCO2, & 100 % of low MV corresponded to either normal or low EtCO2 (Fig. 63a). Correlation was poor between the capnograph’s RR & EtCO2 measurements: normal EtCO2 coincided with adequate RR just 24.9 % of the time & none of the low RR measurements were revealing of a high EtCO2 (Fig. 63b). When using RR as a proxy for MV it was also noted that low MV is observed at a wide range of RRs, with only 15.5 % of all low MV events captured by a low EtCO2-based RR (Fig. 63c).

**Conclusions:** This study confirmed that EtCO2 is an inadequate proxy for MV in non-intubated patients. EtCO2-based RR is a poor proxy for EtCO2 & an even worse proxy for MV.

## P250 Evaluation of the relationship between non-invasive minute ventilation and end-tidal CO2 in patients undergoing general vs spinal anesthesia

### C. J. Voscopoulos^1^, J. Freeman^2^, E. George^3^

#### ^1^University of Hawaii, John A. Burns School of Medicine, Honolulu, USA; ^2^Respiratory Motion Inc., Waltham, USA; ^3^Massachusetts General Hospital, Boston, USA

**Introduction:** Capnography (EtCO2) is unreliable for monitoring respiratory status in non-intubated patients. We used a noninvasive respiratory volume monitor (RVM), which provides accurate measurements of minute ventilation (MV), tidal volume & respiratory rate (RR) in non-intubated patients, to compare the relationship between EtCO2 & MV in intubated patients with general anesthesia (GA), non-intubated patients with spinal anesthesia (SA) & awake volunteers.

**Methods:** RVM data (ExSpiron, Respiratory Motion Inc., Waltham MA) were collected from 153 patients in 3 groups: Group 1, 54 patients, age:65.2 ± 12.1 yrs, BMI:31.2 ± 6.3 kg/m2, surgery with GA; Group 2, 60 patients, 68.9 ± 9.0 yrs, 30.5 ± 5.8 kg/m2, surgery with SA; Group 3, 39 volunteers, 48.5 ± 13.8 yrs, 27.9 ± 8.6 kg/m2, coached to breathe at varied RRs. Groups 1&2 EtCO2 data were collected via ventilator (Draeger Apollo), with an ET tube in Group 1 & sampling nasal cannula in Group 2. Group 3 EtCO2 data were collected via capnograph (Capnostream20, Covidien) with nasal cannula. Deming regression quantified the relationship between EtCO2&MV. EtCO2 sensitivity&mean EtCO2 were compared across cohorts by un-paired t-tests.

**Fig. 64 (Abstract P250).**
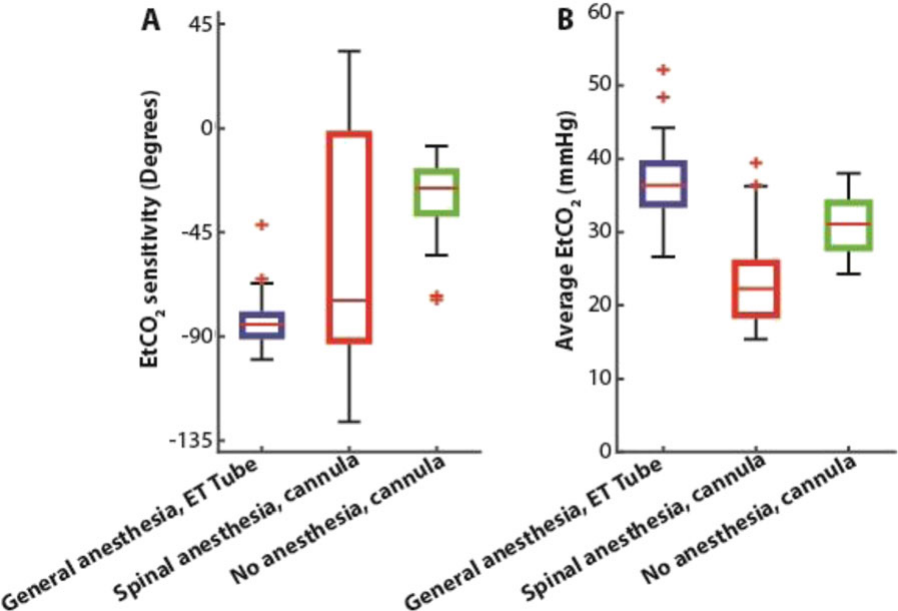
ᅟ

**Results:** The EtCO2 sensitivity was significantly higher in intubated patients vs volunteers (-83.5° ± 9.7°vs-30.1° ± 16.1°, p < 0.001, Fig. 64a). In non-intubated patients the sensitivity was not normally distributed & spanned the range of intubated patients & volunteers. Measured EtCO2 values were higher in Group1 than Groups 2&3 (37.2 ± 4.4 mmHg vs 23.0 ± 5.2 mmHg vs 31.0 ± 4.0 mmHg, respectively, p < 0.001, Fig. 64b). EtCO2 measurements were normally distributed in all groups.

**Conclusions:** Our results show that EtCO2 lacks adequate sensitivity to changes in MV&introduces measurement bias with the nasal cannula.

## P251 Respiratory volume monitoring provides early warning of respiratory depression and can be used to reduce false alarms in non-intubated patients

### C. J. Voscopoulos^1^, D. Eversole^2^, J. Freeman^2^, E. George^3^

#### ^1^University of Hawaii, John A. Burns School of Medicine, Honolulu, USA; ^2^Respiratory Motion Inc., Waltham, USA; ^3^Massachusetts General Hospital, Boston, USA

**Introduction:** Pulse oximetry (SpO2) is associated with significant false alarms, & gives late notice of respiratory depression. In this observational study, we assessed the ability of a respiratory volume monitor (RVM) that non-invasively measures minute ventilation (MV), tidal volume & respiratory rate to capture respiratory depression in advance of low SpO2 & with fewer false alarms.

**Methods:** RVM and SpO2 data were collected from 240 patients (age: 66.8¡À10.3 yrs, BMI:29.6¡À5.7 kg/m2) at 1-min intervals. ¡°Predicted¡ ± MV (MVPRED) was calculated for each patient, based on body surface area. ¡°Low SpO2¡ ± (alarm condition) was an SpO2 < 90 %; ¡°desaturation event¡ ± was ¡°Low SpO2¡ ± for ¡Ý2min; and ¡°false alarm¡ ± was ¡°Low SpO2¡ ± for <2 min. ¡°Un-Safe MV¡ ± was defined as MV < 40%MVPRED. MFANOVA was used to evaluate differences in the clinical populations, & unpaired one-sided t-tests were used to compare measurements across groups.

**Fig. 65 (Abstract P251).**
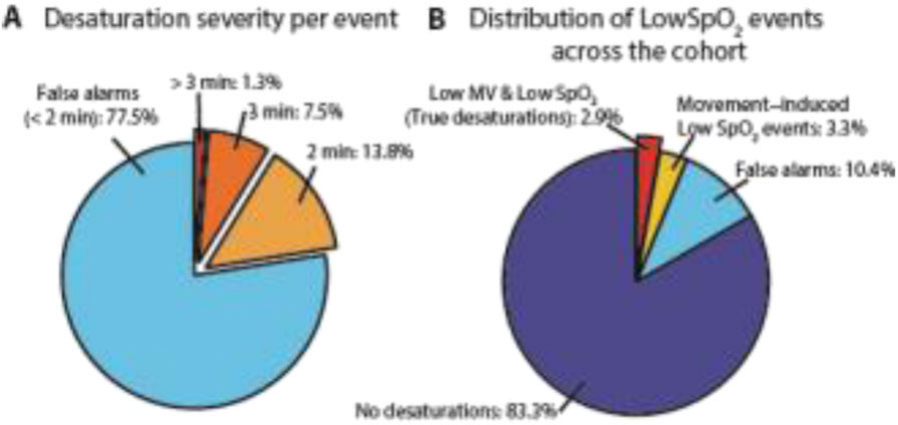
ᅟ

**Results:** Of 80 alarm conditions, 62 (78 %) were false alarms. The other 18 events (¡Ý 2 min) occurred in 15 patients (Fig. 65a). The RVM showed that 11/18 desaturation events were from patient motion & high MV & only 7 events were ¡°true desaturation¡ ± events, (Fig. 65b). Each true desaturation event was preceded by ¡°Un-Safe MV¡ ± by 16.7¡À4.6 min (mean¡ÀSEM) & the severity of ¡°Un-Safe MV¡ ± was strongly correlated with the time delay (r = 0.77, p < 0.05). While MFANOVA found no difference in the demographics of the populations with true desaturation events vs. false alarms (p > 0.2 for ht, wt, age, BMI, sex), the length of stay in the PACU for true desaturations group was significantly longer (176¡À9min vs. 134¡À18min, p < 0.05).

**Conclusions:** This study showed that >90 % of SpO2 alarms in the PACU were likely false. MV monitoring gives advanced warning of respiratory depression with fewer false alarms.

## P252 P/i index: a predictive edi-derived weaning index during nava

### S. Muttini^1^, R. Bigi^1^, G. Villani^2^, N. Patroniti^3^

#### ^1^AO Desio e Vimercate, Vimercate, Italy; ^2^AO Crema, Crema, Italy; ^3^University of Milan-Bicocca, Monza, Italy

**Introduction:** Edi-derived indices provide reliable predictors of successful separation from the ventilator only during the SBT [1-3]. Their performance is not better than the performance of RSBI [3].

We previously investigated in a physiological study the relationship between Edi peak and Edi area under the curve (P/I Index); we concluded that P/I Index can give important information on the balance between respiratory drive and respiratory demand, and may predict weanability at clinical support level [4].

The aims of the study are: 1) to validate P/I Index as weaning predictor at clinical ventilatory support and 2) to compare P/I Index sensitivity during NAVA in predict SBT success respect to: Edi peak, Neuro-ventilatory efficiency (NVE) and RSBI.

**Methods:** We prospectively enrolled 54 patients ventilated with NAVA meeting criteria for SBT.

For each patient, we identified 2 step: 1) enrollment, T[sub]0[/sub]; 2) end of SBT. We recorded RSBI, Edi peak, and two Edi-derived weaning indices: NVE , P/I Index.

SBT failure and success were defined according to clinical protocol in use.

**Results:** 34 patient completed SBT and were successfully extubated; 11 patients failed SBT ; 9 patients failed the first weaning step before the trial (subgroup A).

At T[sub]0[/sub] differences between success (S) and failure (F) group were statistically significant for P/I only.

At the end of SBT differences between S and F group were statistically significant for RSBI and P/I.

**Conclusions:** P/I Index predict ability to sustain SBT under ventilator assistance and during SBT. Subgroup A may represent a group of patients not adequately assisted, where classic indices and NVE are not able to predict the inability to start SBT. Further investigations are needed to understand whether P/I Index may provide information on adequacy of assistance during NAVA ventilation.

**References**

1) Liu, Crit Care 2012, 16:R143

2) Rozé, BJA 2013, 111(6):955-60

3) Dres, ICM 2012, 38:2017-25

4) Muttini, JCC 2015, 30(1): 7-12

**Fig. 66 (Abstract P252).**
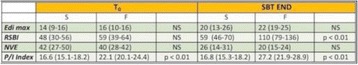
Results (median, Q1, Q3)

## P253 Adequacy of ventilation in patients receiving opioids in the post anesthesia care unit: minute ventilation versus respiratory rate

### G. Williams^1^, C.J. Voscopoulos^2^, J. Freeman^3^, E. George^4^

#### ^1^UT Health Science Center at Houston, Houston, TX, USA; ^2^University of Hawaii, John A. Burns School of Medicine, Honolulu, HI, USA; ^3^Respiratory Motion Inc., Waltham, MA, USA; ^4^Massachusetts General Hospital, Boston, MA, USA

**Introduction:** Opioid use decreases respiratory drive and can cause opioid-induced respiratory depression (OIRD), which is challenging to recognize in non-intubated patients from respiratory rate (RR) alone. Using a non-invasive respiratory volume monitor (RVM) that measures minute ventilation (MV), tidal volume (TV) and RR, we examined whether RR alone can identify LowMV episodes (used to define OIRD) in the post-anesthesia care unit (PACU).

**Methods:** After written informed consent, MV, TV and RR measurements were collected via RVM (ExSpiron, Respiratory Motion, Waltham MA) from 358 patients (age: 67.1, 19-91 yrs; BMI: 29.8,19-49 kg/m2) undergoing elective joint replacement surgery. 205 patients received PCA opioids in the PACU: 96 received hydromorphone (0.2 mg) and 109 received morphine (1 mg per dose). RVM measurements were calculated from 30-second segments in the 30 minutes before and after first dose. Predicted MV (MVPRED) was calculated from each patient’s BSA. LowMV was defined as MV < 40%MVPRED for >1 min. LowRR was defined as RR < 6breaths/min. Sensitivity and specificity analysis correlated LowRR with LowMV.

**Fig. 67 (Abstract P253).**
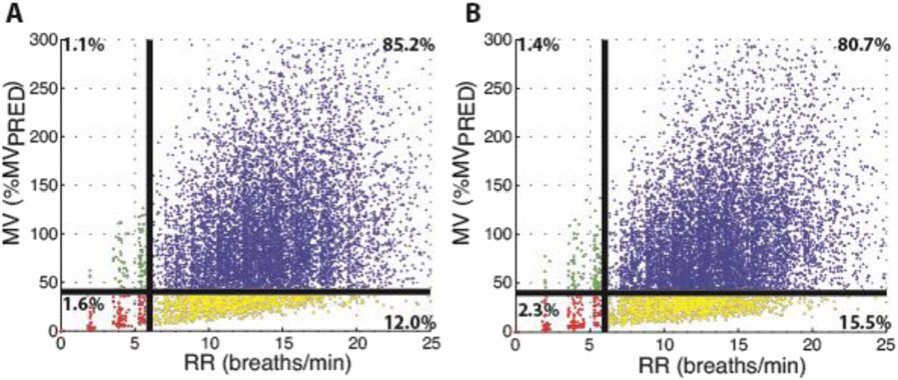
ᅟ

**Results:** For patients receiving opioids, LowMV occurred 13.7 % of the time before and 17.8 % of the time after a dose. Importantly, LowRR coincided with less than 12.5 % of all LowMV episodes. On average, patients experienced 1.5 ± 0.2 LowMV episodes before opioid (range: 0-9episodes; duration: 2.17 ± 0.13 min) and 1.8 ± 0.2 LowMV episodes (0-9episodes; 2.40 ± 0.12 min) after opioid dosage (p > 0.5). In both groups, a low RR alarm set to below 6 breaths/min would miss 88.3 % of LowMV episodes, yielding a sensitivity of 12.4 %, specificity 98.5 %, positive predictive value 60.5 %, and negative predictive value 85.8 %. Figure 67 shows a plot of paired MV and RR measurements.

**Conclusions:** LowRR measurements alone provide an insufficient assessment of respiratory status. Our data suggest that LowMV is primarily related to a decrease in TV and not RR.

## P254 Comparison of regional and global expiratory time constants measured by electrical impedance tomography (EIT)

### A. Waldmann^1^, S. Böhm^1^, W. Windisch^2^, S. Strassmann^2^, C. Karagiannidis^2^

#### ^1^Swisstom AG, Landquart, Switzerland; ^2^Kliniken der Stadt Köln, Pneumology and Critical Care Medicine, Witten / Herdecke University, Ostmerheimer Str. 200, 51109 Cologne, Germany

**Introduction:** Although regional compliance (C) and resistance (R) can vary considerably between different lung regions, traditional pulmonary function tests can measure global values, only. To overcome this drawback we recently proposed a novel EIT-based method to estimate regional expiratory time constants (τ = tau). In this abstract regional values were compared with global ones.

**Fig. 68 (Abstract P254).**
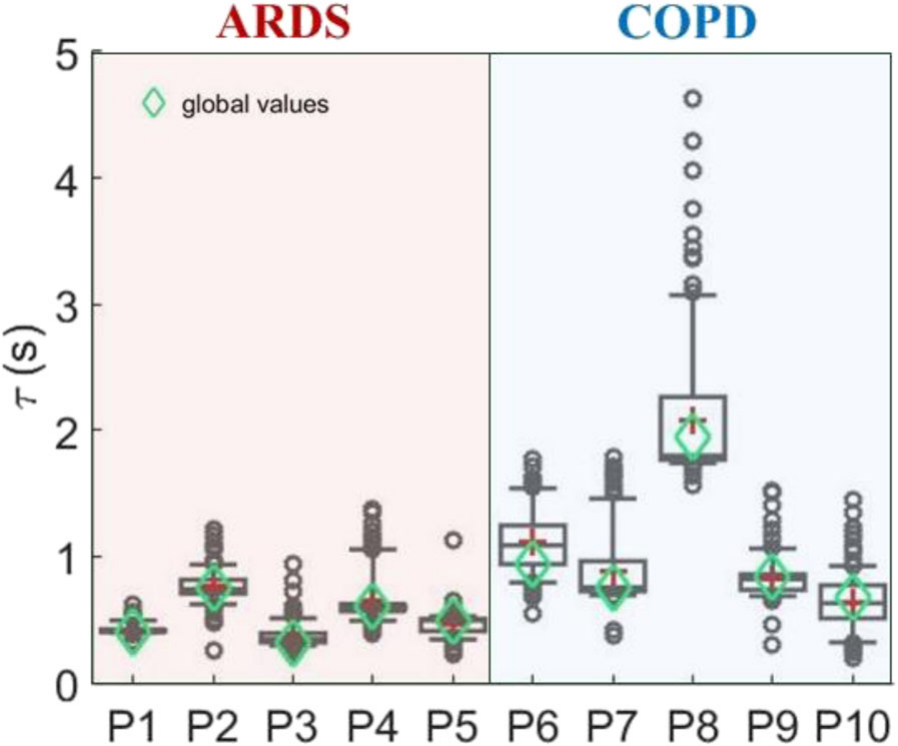
ᅟ

**Methods:** In ten intubated patients with hypoxemic and hypercapnia lung failure time constants (τ = R·C) were estimated from global and regional volume EIT signals obtained by Swisstom BB^2^ (Swisstom, Switzerland). A first order exponential decay model was fitted to each regional and global impedance curve using MATLAB (The MathWorks, USA). The spread and mean of regional τ values is depicted in Fig. 68 and compared with the one τ value derived from the global signal.

**Results:** ARDS lungs exhaled “faster” whereas COPD lungs were “slower”, the latter reflecting the predominance of airway obstruction. ARDS patients show only minor regional differences and global τ corresponded perfectly with the mean τ derived from different lung regionals. In contrast, COPD patients showed a large spread of their regional τ values. Nonetheless, the mean of the regional τ values corresponded well with global τ .

**Conclusions:** Mean expiratory time constant derived from regional EIT signals was similar to the one value calculated from the global EIT signal in both, patients with short (ARDS) and long expiration time requirements (COPD). As a sign of heterogeneous disease, the latter showed a wide spread of regional τ values.

## P255 Electrical impedance tomography: robustness of a new pixel wise regional expiratory time constant calculation

### A. Waldmann^1^, S. Böhm^1^, W. Windisch^2^, S. Strassmann^2^, C. Karagiannidis^2^

#### ^1^Swisstom AG, Landquart, Switzerland; ^2^Kliniken der Stadt Köln, Pneumology and Critical Care Medicine, Witten/ Herdecke University, Ostmerheimer Str.200, 51109, Cologne, Germany

**Introduction:** Passive exhalation shows a characteristic exponential course with asymptotic approximation of the end expiratory lung volume. It can be described as V(t) = Vinsp · e^(-t/(R · C)) where V is the lung volume at time t, Vinsp the volume at end inspiration, R the resistance and C the compliance of the lungs. The product of R · C is called τ (tau). Although R and C can vary considerably between different lung regions, traditional pulmonary function tests provide global values, only. Therefore, we recently proposed a novel method to obtain regional τ values using electrical impedance tomography (EIT). As this approach can be challenging in presence of small ventilation amplitudes, noisy signals and heterogeneity we determined in this study its robustness.

**Fig. 69 (Abstract P255).**
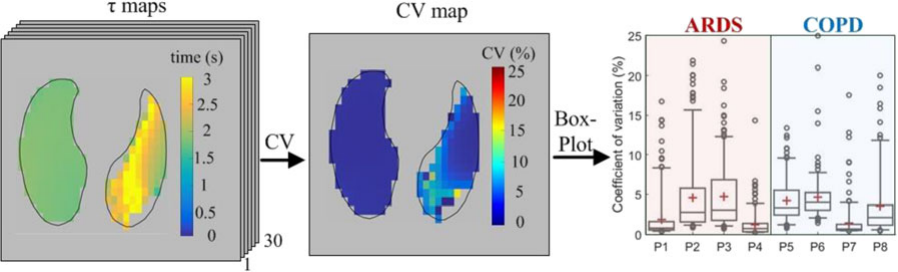
ᅟ

**Methods:** In 8 intubated patients, with hypoxemic or hypercapnic respiratory failure, we measured EIT signals at 50 images per second using Swisstom BB^2^ recording 30 consecutive breaths during steady state conditions. Regional breath by breath τ values (Fig. 69) were estimated by fitting an exponential model to each regional curve. Only τ values within the range of 0.05 and 3 s stemming from appropriately fitted curves (R^2^ > 0.6) were considered thereby excluding poorly ventilated and poorly fitted lung areas. To estimate the robustness of τ , we calculated the regional coefficient of variation (CV) for all breaths as standard deviation divided by mean.

**Results:** Mean and median CV values of each patient were below 5 %, however some pixels showed values of up to 25 % mainly at the lung boundaries or close to poorly ventilated areas.

**Conclusions:** The suggested approach for calculating regional expiratory τ values provided robust results with low coefficients of variation. However, certain lung regions at the lungs’ boundary showed high variability. Therefore, to increase reliability such pixels should be excluded from future calculations.

## P256 Validation of regional and global expiratory time constant measurement by electrical impedance tomography in ards and obstructive pulmonary diseases

### C. K. Karagiannidis^1^, A. W. Waldmann^2^, S. B. Böhm^2^, S. Strassmann^1^, W. W. Windisch^1^

#### ^1^ARDS and ECMO Center Köln-Merheim, Cologne, Germany; ^2^Swisstom, Chur, Switzerland

**Introduction:** Obstructive airway diseases such as asthma or COPD are increasing in numbers and frequently lead to severe exacerbations with the need of non-invasive or invasive mechanical ventilation. Severe COPD in particular is often characterized by inhomogeneous ventilation. In case of mechanical ventilation in obstructive pulmonary diseases or ARDS, modern ICU ventilators provide accurate measurements of pressure-volume curves on a global, but not on a regional level. Electrical impedance tomography (EIT) in contrast is an established non-invasive tool to measure regional ventilation. The aim of our study was to calculate an EIT-based (32x32 pixel, 50 frames per second) expiratory time constant τ (tau) for each image pixel and a global one for the entire lung and to validate it against a global volume signal measured at the airway opening.

**Methods:** In 12 intubated patients with hypoxemic and/or hypercapnic respiratory failure we performed EIT and pneumatic volume measurements (Swisstom BB2, Switzerland) on different PEEP level recording 100 consecutive breaths during steady state conditions with breathing frequencies between 14 and 16/min. Regional breath by breath τ values were obtained by fitting in a nonlinear least square manner an exponential function to the EIT curve of each pixel. The same algorithm was applied to the pneumatic volume curve and correlated with the mean τ of all regional EIT measurements.

**Results:** The mean τ calculated from the regional EIT signals showed a strong correlation with the global τ calculated from the pneumatic volume signal. In 2773 breaths Pearson r was 0.8752 (0.8662 to 0.8836) and r2 0.7660. Patients with severe COPD typically showed longer time constants than those with ARDS. The mean τ calculated from the global EIT signals showed a strong correlation with the global τ calculated from the pneumatic volume signal as well (Pearson r: 0.8978).

**Conclusions:** The mean τ calculated from the regional EIT signals shows a very strong correlation with the global pneumatic volume signal reflecting EIT as a valid method to calculate global and regional expiratory time constants. EIT therefore provides for the first time a regional breath by breath measurement of airflow obstruction and may help to guide mechanical ventilation especially in obstructive pulmonary diseases.

## P257 Transpulmonary pressure in a model with elastic recoiling lung and expanding chest wall

### P. Persson, S. Lundin , O. Stenqvist

#### Sahlgrenska Univ Hospital, Göteborg, Sweden

**Introduction:** We have shown in pigs and ARDS patients that lung elastance can be determined without esophageal pressure as the change in PEEP divided by the change in end-expiratory lung volume, deltaPEEP/deltaEELV [1,2]. The hypothesis is that this is an effect of contra-directional forces of the expanding rib cage and recoil of the lung that creates negative pleural pressure, which persists also at an increased PEEP/EELV. Esophageal pressure, used as a surrogate for pleural pressure (PPL) is unreliable for determining absolute end-expiratory esophageal pressure, because of extra-pleural effects on measurements. The aim of the present study was to evaluate the effect of an expanding rib cage on absolute pleural pressure changes during PEEP steps in a respiratory system model.

**Fig. 70 (Abstract P257).**
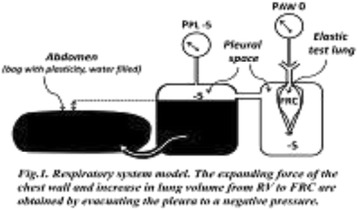
ᅟ

**Methods:** A model with a recoiling elastic lung and expansive chest wall complex was built (Fig. 70). Lung elastance could be varied between 46, 24, and 17 cmH2O/L. In volume control, PEEP was increased from 0 to 4, 8, and 12 cmH2O, while airway and pleural pressure was measured and deltaEELV obtained by spirometry.

**Fig. 71 (Abstract P257).**
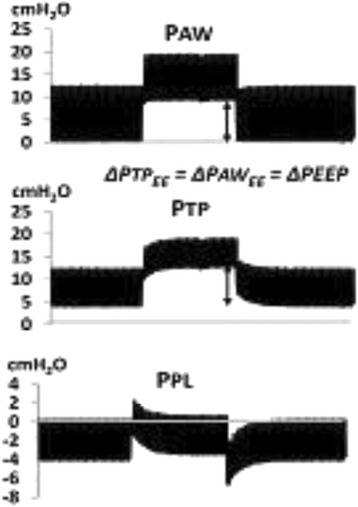
Airway, transpulmonary and pleural pressure during a PEEP change 0-8-0 cmH2O

**Results:** When PEEP was increased endexpiratory pleural pressure increased during the first expiration, but then declined back to baseline, while end-expiratory transpulmonary pressure (PTPEE) increased until it equaled the change in end-expiratory airway pressure (deltaPEEP = deltaPTPEE) (Fig. 71). There was an good correlation between transpulmonary pressure at all lung volume levels derived from PAW-PPL (pleural pressure) and transpulmonary pressure derived from lung elastance calculated as deltaPEEP/deltaEELV, r2 = 0.99, y = 1.01x.

**Conclusions:** As end-expiratory pleural pressure remains unchanged when increasing PEEP, end-expiratory transpulmonary pressure changes as much as PEEP and lung compliance can be determined as deltaEELV/deltaPEEP.

**References**

1. Stenqvist et al Acta Anestesiol Scand 2012;56:738-47

2. Lundin et al Acta Anesthesiol Scand 2015;59:185-96

## P258 Lactate in pleural and abdominal effusion

### G. Porta^1^, F. Numis^2^, C. S. Serra^3^, A. P. Pagano^4^, M. M. Masarone^5^, LR Rinaldi^1^, AA Amelia^1^, MF Fascione^1^, LA Adinolfi^1^, ER Ruggiero^2^

#### ^1^Second University of Naples, Naples, Italy; ^2^San Paolo Hospital, Naples, Italy; ^3^University of Sassari, Sassari, Italy; ^4^Cardarelli Hospital, Naples, Italy; ^5^University of Saerno, Salerno, Italy

**Introduction:** The aim of this study is to evaluate whether the increase in lactate in pleural and abdominal effusion can be used as a criterion for the differential diagnosis of the nature of the fluid (transudate or exudate).The presence of pleural or abdominal effusion is a frequent finding in patients in the ICU and Internal Medicine Department.It is possible to distinguish between transudate and exudate by Light’s criteria[1,2].Pursuing an acid-base assessment of the fluid,we have noticed an increase in the value of lactate,beyond the blood range,in the cases that were diagnosed like exudate to the calculation of Light criteria[4].The advantages of this test is that it is a simple test,executable by the physician and his result is available within few minutes.

**Methods:** We collected data of 30 patients who had clinical indication for thoracentesis or paracentesis.For each patient we performed arterial blood gas with lactate,total protein and serum LDH dosage,acid-base assessment with lactate,total protein and LDH dosage,cytology and bacterial culture on the fluid.Of every patient we calculated “liquid/serum” lactate ratio in order to measure the eventually present increase in pleural or abdominal effusion.The diagnosis of the liquid effusion nature (exudate or transudate) was performed by Light’s criteria.A statistical analysis was carried out by performing a ROC curve in order to find the best cut-off for liquid/serum lactate ratio predicting the presence of an exudate.

**Results:** Of the 30 patients (13 [43 %] males and 17 [57 %] females, mean age 65,53 ± 13,271 years) 15 had peritoneal effusion and 15 had pleural effusion,15 patients had a exudate and 15 a transudate by Light’s criteria.We performed a ROC curve to predict the presence of exudate by the liquid serum lactate ratio and we obtained an AuROC of 0.69 with the best cut-off value of 2,02 (Sensitivity 0,73 and Specificity 0,73). When we performed the same analysis only on pleural effusion patients we obtanined a an AuROC of 0,78 with the best cut-off value of 2,13 (Sensitivity 0,7, Specificity 1,0).

**Conclusions:** Liquid/Serum lactate ratio seems to be a promising tool to predict the presence of an exudate,in particular in pleural effusions. Further studies are needed to warrant these statements.

**References**

[1] Richard W. Light: N Engl J Med, Vol. 346, No. 25 2002

[2] N.A.Maskell et al.: Thorax;58(Suppl II):ii8–ii17 2003

[3] A.A.Nanji et al.: J Emerg Med. 1984;1(6):521-6.

[4] S.M.Smith et al.: Journal of Clinical Microb,Mar.1989,385-388

## P259 Outcome of patients admitted to the intensive care with pulmonary fibrosis

### F. Asota, K. O’Rourke, S. Ranjan, P. Morgan

#### East Surrey Hospital, Surrey and Sussex NHS Trust, Surrey, UK

**Introduction:** The objective of this study was to evaluate the outcomes of patients admitted to the intensive care with pulmonary fibrosis. Numerous studies have demonstrated a poor outcome with patients admitted to the intensive care unit (ICU) with pulmonary fibrosis [1-2].

**Methods:** A computerised hospital database was used to search for all patients admitted to the ICU at East Surrey Hospital with pulmonary fibrosis between 2003-2015. Data was analysed using Microsoft Excel 2007 and GraphPad software. The primary endpoint was survival at unit and hospital discharge.

**Results:** 8048 records were reviewed and 79 met the inclusion criteria. A control group of patients without a diagnosis of pulmonary fibrosis was used for comparison (7954). Results showed 54 % of patients with pulmonary fibrosis died before discharge from the unit compared to 18.8 % in the control group p = 0.0001 (Table 34). Similarly, 63.3 % of patients with pulmonary fibrosis died before hospital discharge compared to 26.3 % of the control group p = 0.0001 (Table 35).

**Conclusions:** The results from this study are consistent with previous findings. The risk of death prior to discharge from our intensive care unit is almost 30 % higher for patients with pulmonary fibrosis. From the data presented above, it may be prudent to consider that patients with pulmonary fibrosis presenting with no reversible causes of acute respiratory failure may be less likely to benefit from admission to the ICU.

**References**

1. Al-Hameed FM, Sharma S.Outcome of patients admitted to the ICU for acute exacerbation of idiopathic pulmonary fibrosis. Can Respir J 2004;11: 117-122

2. Bilvet S, Philit F, Sab JM.Outcome of patients with idiopathic pulmonary fibrosis admitted to ICU for respiratory failure. Chest 2001;120:209-212.

**Table 34 (Abstract P259).** Discharges from unit

Unit outcome	Died	Alive	Total
Pulmonary fibrosis	43	35	78
Control	1484	6446	7930
Total	1527	6841	8008

**Table 35 (Abstract P259).** Discharges from hospital

Died	Alive	Total	Pulmonary fibrosis
50	27	77	Control
2093	5695	7788	Total
2143	5722	7865	

## P260 Sedation and analgesia practice in extra-corporeal membrane oxygenation (ECMO)-treated patients with acute respiratory distress syndrome (ARDS): a retrospective study

### J. W. DeBacker^1^, E. Tamberg^1^, L. O’Neill^1^, L. Munshi^1^, L. Burry^1^, E. Fan^2^, S. Mehta^1^

#### ^1^Mount Sinai Hospital and University of Toronto, Toronto, Canada; ^2^University Health Network-Toronto General Hospital and Univeristy of Toronto, Toronto, Canada

**Introduction:** For patients with ARDS supported on ECMO, the optimal sedation and analgesia strategy is unknown. Our objective was to characterize pharmacological practice in a high-volume ECMO center and describe sedation depth, incidence of delirium and mobilization in this population.

**Methods:** We conducted a retrospective study to describe medication (sedative, analgesic, paralytic, antipsychotic) administration in 60 patients treated with VV-ECMO for ARDS at Toronto General Hospital. We recorded Sedation-Agitation Scale scores (SAS), medications administered, routes and doses, CAM-ICU scores, and mobilization while on ECMO.

**Results:** (INTERIM) 23 adults (19 males, 48 ± 11 years, APACHE II 31 ± 5) with severe ARDS (P/F 72 ± 19), mainly from pulmonary sepsis (91 %), were treated with VV-ECMO from Jan 2012-July 2014. Median duration of VV-ECMO was 9 days (IQR 5-11). At 48 hr post ECMO initiation all patients were deeply sedated (SAS[<=]3), with 96 % and 100 % receiving a sedative (midazolam 87 %, propofol 26 %) and opioid (fentanyl 91 %) infusion respectively. Within 24 hr prior to ECMO discontinuation, in patients who survived ECMO (78 %), 44 % of patients were still deeply sedated. At this time, 89 % were receiving sedatives (67 % infusion vs 22 % intermittent) and 83 % were receiving opioids (72 % infusion vs 11 % intermittent). At 48 hr post ECMO discontinuation 83 % of patients reached light levels of sedation (SAS > 3). At this time point only 61 % were still receiving sedatives (33 % infusion vs 29 % intermittent) and 50 % were receiving opioids (33 % infusion vs 17 % intermittent); 22 % were not receiving any sedative or opioid. While on ECMO 57 % of patients had cisatracurium infusions for a median duration of 16 hrs (IQR 10-76). 88 % had [>=]1 positive CAM-ICU score for delirium. Haloperidol and quetiapine were the primary antipsychotics used throughout ECMO (30 % and 57 % respectively). 57 % of patients underwent physical therapy within a median of 5 days of starting ECMO (IQR 3-7), yet the majority (62 %) achieved only passive range of motion in bed during ECMO.

**Conclusions:** Patients with ARDS were deeply sedated with midazolam and fentanyl infusions after VV-ECMO initiation. Patients transitioned from deep to light levels of sedation around the time of ECMO discontinuation with many receiving sedation and analgesia via intermittent bolus or not at all. Few patients were actively mobilized during ECMO treatment.

## P261 Characteristics and outcomes of patients deemed not eligible when referred for veno-venous extracorporeal membrane oxygenation (vv-ECMO)

### S. Poo^1^, K. Mahendran^2^, J. Fowles^2^, C. Gerrard^2^, A. Vuylsteke^2^

#### ^1^University of Cambridge, Cambridge, UK; ^2^Papworth Hospital, Cambridge, UK

**Introduction:** Veno-venous ECMO is a well-established life-saving measure in patients with severe acute respiratory failure refractory to conventional therapies. There are currently no accepted definite guidelines defining the suitability of patients nor studies outlining the outcomes of all patients referred. Criteria for acceptance and refusal are defined according to international standards and clinical judgement. We aimed to characterise the patient population based on whether they were refused or accepted into the ECMO service, and compare the outcomes in each category.

**Methods:** We reviewed all patients referred for veno-venous ECMO to one national centre over 44 months. Clinical parameters include gender, age, working diagnoses; referral centres, ventilator requirements, respiratory investigations and survival information. All referrals were categorized according to whether they were accepted or refused. The refused cohort was substratified based on whether they were ¡®too well¡¯ or ¡®futile¡¯. Patients were followed up to 180 days after referral.

**Results:** Of 451 referrals, 276 (61 %) were refused whilst 125 (28 %) were accepted into the service. The remaining 50 (11 %) were transferred to another national centre due to the lack of ECMO beds at the studied centre. The mean age and duration of mechanical ventilation before referral were significantly higher in the futile group (56.1 years, 5.1 days) compared to those who were accepted (42.9 years, 3.1 days) or ¡®too well¡¯ (46.5 years, 3.3 days) (p <0.05). The futile group had higher rates of multi-organ failure and severe immunosuppression. Gender, body mass index, lung injury scores and degree of hypoxaemia were similar in all 3 groups. Interestingly, 60 % of patients who were too well had higher lung injury scores (Murray score ¡Ý3) compared to 45 % and 41 % in those who were futile and accepted respectively. The survival of the ¡®futile¡¯ group was poor; whilst those who were too well and accepted had similar outcomes. At 180 days, 44 % of those who were too well had died, whilst 15 % of those who were futile survived.

**Conclusions:** We present the first report on the outcomes of patients who referred but not accepted for vv-ECMO in the context of a national centralised referral system. Identical survival at 30, 60 and 180 days between the accepted patients and the ones deemed too well warrant further investigations.

## P262 The SAVE SMR for veno-arterial ECMO

### R. Loveridge, C. Chaddock, S. Patel, V. Kakar, C. Willars, T. Hurst, C. Park, T. Best, A. Vercueil, G. Auzinger

#### King’s College Hospital NHS Foundation Trust, London, UK

**Introduction:** The international Extracorporeal Life Support Organisation (ELSO) publishes ECMO outcome scores. In 2015, Schmidt et al validated the SAVE score which uses 12 pre-ECMO variables derived from the ELSO dataset to assist in predicting survival after veno-arterial ECMO and to allow comparison between centres - we have evaluated our outcomes against this.

**Methods:** All patients receiving ECLS had data prospectively collated and where possible, a SAVE score was calculated - actual outcomes were compared to provide quality assurance information and a standardised mortality ratio which we describe as our ‘SAVE SMR’.

**Results:** There have been 57 ECLS runs in 53 patients and 33 patients who received peripheral VA ECMO – all of these cases were moribund by conventional intensive care standards, and included 17 true eCPR cases. Overall, 21 weaned (64 %) and 17 survived (52 %) – 3 who were weaned but died, were refused transfer for destination VAD/transplant assessment.

Within the VA ECMO group who were not eCPR at initiation, 13/17 (76 %) weaned and 11 survived (65 %). The SAVE-score was calculated for 13 cases, with 4 patients excluded as they were post planned procedures (cardiac surgery or emergency VT ablation where 3/4 (75 %) survived).

The mean age was 37 years (15-64), mean SOFA score in the 6 hours prior to ECMO cannulation being 17 (12-24) and the mean SAVE Score was – ve 13 (-2 to- 20).

11/13 (85 %) patients were in the most severe category (V) as compared to 6.7 % & 20.4 % in the derivation and validation cohorts in the study. The predicted survival from our SAVE scores was 20.8 %, whilst our observed survival to discharge was 61.5 %.

The standardized mortality ratio (SMR) for our service was 0.49.

**Conclusions:** King’s College Hospital ECLS service, which recently won an international award from ELSO, performs excellently.

We recommend ensuring that centres undertake benchmarking for governance purposes.

We recommend supporting centres that do not impose restrictive criteria in regard of the initiation of ECLS – moribund patients (INTERMACS classification 0 or 1) can do well with mechanical circulatory support.

Destination therapy & commissioning remains a problem within the UK.

**Reference**

Schmidt M et al. European Heart Journal 36: 2246–2256, 2015

## P263 A simplified score to predict early (48 h) mortality in patients being considered for VA-ECMO

### A. Borgman, A. G. Proudfoot, E. Grins, K. E. Emiley, J. Schuitema, S. J. Fitch, G. Marco, J. Sturgill, M. G. Dickinson, M. Strueber, A. Khaghani, P. Wilton, SM Jovinge

#### Meijer Heart & Vascular Institute, Grand Rapids, USA

**Introductions:** Patient selection for veno-arterial extracorporeal membrane oxygenation (VA-ECMO) in refractory cardiogenic shock, in particular the early recognition of futility, remains a significant clinical challenge. Previous case series and the SAVE score [1] have focussed on variables that predict in-hospital mortality. The early identification of those patients who are likely to deteriorate and die within 48 hours despite VA-ECMO is an attractive paradigm to guide clinical decision making and improve appropriate resource utilization.

**Methods:** Data were retrospectively collected from the records of consecutive patients supported with VA-ECMO at Spectrum Health between January 2010 and October 2015. Based on a review of the current literature, 8 candidate predictors of 48 h-mortality were expertly selected (Image 1); the ‘worst’ value within the 24 hours prior to ECMO cannulation was considered for modeling. Ridge regression [2] was used to develop a risk prediction model and cross validation applied to select the optimal tuning parameter. Model discrimination was assessed using the area under the receiver operating curve (AUC) and calibration was assessed using several common indices.

**Fig. 72 (Abstract P263).**
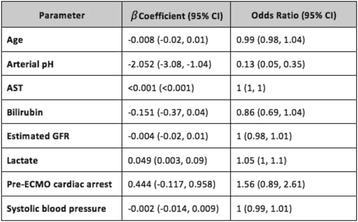
Model estimates with bootstrap confidence intervals

**Results:** 141 patients were included. 48 h-mortality was 16 % (22 patients) with an in-hospital mortality of 43 %. The model demonstrated good discriminatory power AUC = 0.79 (Bootstrap 95 % CI = 0.68 to 0.88) and good calibration. Arterial pH shows the strongest association with mortality in our model (Fig. 72).

**Conclusions:** In a small retrospective cohort study, an 8-parameter score of pre-ECMO variables predicted mortality with an AUC of 0.789. Whilst validation and optimisation in a larger cohort is required, the use of pre-ECMO variables corresponds with medical decision-making in real-time, enhancing clinical utility in the acute-setting.

**References**

1. Schmidt M et al. European Heart Journal 2015; 36;33:2246-56

2. Pavlou M et al. BMJ. 2015 Aug 11;351:h3868

## P264 Lung function six months post extra corporeal membrane oxygenation (ECMO) for severe acute respiratory failure in adult survivors

### C. Sampson, S. Harris-Fox

#### Glenfield Hospital, UHL, Leicester, UK

**Introduction:** Our aim was to assess spirometry values 6 months post ECMO. ECMO is used in severe acute respiratory failure refractory to conventional management. Support entails gas exchange through an extracorporeal oxygenator allowing lung rest as the underlying pathology resolves whilst preventing further damage from ventilator associated lung injury. Knowledge and experience in ECMO is growing however the significant costs and potential complications associated with the technique mean it is imperative to evaluate the longer term outcomes.

**Methods:** Glenfield hospital is one of five centres in the UK commissioned by NHSEngland to provide respiratory ECMO support. All adult ECMO survivors are invited to a follow up clinic 6mths post ECMO decannulation and undertake spirometry by rolling seal. FEV1 (Forced Expiratory Volume in 1 sec), FVC (Forced Vital Capacity) and PEF (Peak Expiratory Flowrate) are measured and compared to normal values indexed to patient’s age, gender and Body Mass Index.

**Results:** Between January 2011 and June 2015 100 patients attended follow-up clinic. Average age 42.4 years (SD 12.9) with 48 % males. Average number of days on ECMO was 8.5 (SD 6.3).

Six patients were unable/unwilling to undertake spirometry leaving 94 patients’ data for analysis. Mean FEV1, FVC and PEF, presented as a % of that predicted (standard deviation) were 84.5 % (SD 21.4 %), 87.9 % (SD 21.5) and 97.6 % (SD 27.9) respectively. Normal values are > =80 % predicted. 19 patients (20 %) had a FEV1 < 70 % predicted, of which 7 patients (39 %) had a diagnosis of asthma. 19 patients (20 %) had FVC values <70 % predicted, indicating a moderate or severe restrictive defect. Patients with FVC or FEV1 < 70 % had a significantly longer time on ECMO than patients with values > 70 % (11.3 vs 6.2 days, p < 0.001).

**Conclusions:** 20 % of our ECMO survivors demonstrated a moderate/severe obstructive, restrictive or mixed defect. These patients had a significantly longer ECMO run than those with normal values. This is comparable to a smaller study in ECMO patients and 2 studies assessing spirometry in conventionally treated ARDS patients at 6 months (1-3).

**References**

1. Linden et al. Act Anaes Scand. 2009;53:489-95

2. Neff et al Chest. 2003;123:845-53

3. Masclans et al. Chest.2011;1:1340-6

## P265 Bicarbonate dialysis removes carbon dioxide in hypoventilated rodents.

### M. E. Cove^1^, L. H. Vu^2^, A. Sen^3^, W. J. Federspiel^4^, J. A. Kellum^4^

#### ^1^National University Hospital, Singapore, Singapore; ^2^National University Singapore, Singapore, Singapore; ^3^Mayo Clinic, Phoenix, AZ, USA,^4^University of Pittsburgh , Pittsburgh, PA, USA

**Introduction:** Low tidal volume ventilation improves outcomes in the acute respiratory distress syndrome, but may impair carbon dioxide (CO[sub]2[/sub]) clearance. Extracorporeal carbon dioxide removal (ECCO[sub]2[/sub]R) can control CO[sub]2[/sub], but existing devices are expensive and require considerable expertise to operate. Since CO[sub]2[/sub] is transported predominantly as bicarbonate, using dialysis to remove bicarbonate offers several advantages, but this approach has been limited by bicarbonate replacement problems. However, contemporary understanding of acid-base balance treats bicarbonate as an anion whose concentration is dependent on the strong ion difference (SID). We hypothesised that a novel dialysate designed to remove bicarbonate, but maintain SID, would lower CO[sub]2[/sub] with no change in pH. We tested this hypothesis in a rodent dialysis model, comparing our dialysate to a conventional solution (Prismasol™).

**Methods:** 14 Sprague-Dawley rats (400-600 g) were anaesthetised with ketamine, xylazine and butorphanol, then intubated and placed on mechanical ventilation. A femoral and jugular vein were cannulated to provide dialysis access. Ventilation rate was adjusted to create hypercapnia and dialysis commenced. Dialysis was provided using a M10 filter (Baxter, France), and the dialysate was constructed such that no bicarbonate is present, but SID was maintained. Blood gases were tested hourly (iStat, Abbott). Control dialysis experiments were performed with Primal™ (Baxter, France). Data is reported as mean and standard deviation and differences compared using Students t-test.

**Results:** Using a blood flow and dialysate flow rate of 1 ml/min, mean pre-filter partial pressure of CO[sub]2[/sub] (PCO[sub]2[/sub]) was 78 mmHg (±14) whereas mean post-filter PCO[sub]2[/sub] was 70 mmHg (±14) using Prismasol and 12 mmHg (±5) using our bicarbonate free dialysate (p < 0.001). Removal of bicarbonate did not significantly affect pH across the dialysis filter (0.003, p = 0.49). This translates to the removal of 0.041 ml/min (±0.0019) of CO[sub]2[/sub]. When scaled up to an adult dialysis filter of 0.9 m^2^, with a blood flow of 200 ml/min, it’s the equivalent of removing 175 ml/min (±8.25) of CO[sub]2[/sub].

**Conclusions:** A bicarbonate free dialysis solution designed to maintain the SID, lowers PCO[sub]2[/sub] in post-filter blood, when compared to conventional dialysate, while maintaining pH. We estimate we could remove 175 ml/min of CO[sub]2[/sub] in adults, existing low-flow ECCO[sub]2[/sub]R devices only remove 90 ml/min.

## P266 Procalcitonin as predictor of primary graft dysfunction and mortality in post-lung transplantation

### C. Mazo Torre^1^, J. Riera^1^, S. Ramirez^1^, B. Borgatta^1^, L. Lagunes^1^, J. Rello^2^

#### ^1^Vall D’Hebron University Hospital, Barcelona, Spain, ^2^Universitat Autonoma de Barcelona, Barcelona, Spain

**Introductions:** The purpose of this study was to evaluate the association of procalcitonin, interleukin-6, interleukin-8 and interleukin-10 plasma levels during the first 72 hours after lung transplantation (LT) with early oxygenation, severity of primary graft dysfunction (PGD), ICU mortality and graft function one year after LT

**Methods:** Prospective observational study in a 32-bed mixed ICU. Procalcitonin, Interleukin-6, 8, 10 plasma levels were measured at 24 h, 48 h, 72 h after LT from 100 LT-recipients included prospectively. Patients were followed until one year after LT. Time and cause of death were recorded.

**Results:** Procalcitonin at 24 h were associated with PaO2/FIO2 over the first 72 h and the severity of PGD at 72 h after LT (procalcitonin at 24 h of patients with grade 0-2 PGD were different from those patients with grade 3 PGD) (p < 0.01). PCT levels at 24 h was higher from the 9 patients who died, (p = 0.02), AUC = 0.74 predict ICU-mortality. Procalcitonin and interleukin-10 at 24 h, 48 h, 72 h were correlated with the FEV1 one year after LT. Interleukin-10 at 48 h, also were correlated with the FVC one year after LT

**Conclusions:** Higher levels of procalcitonin at 24 h following LT are associated with grade 3 PGD at 72 h and ICU mortality. Interleukin-10 and Procalcitonin levels during 72 h posttransplantation were associated with patients with worse graft function after one year.

**Table 36 (Abstract P266).** Characteristics of the population and global outcomes

Age, median (IQR) years	55 (48–60)
Male, no.	59
Bilateral lung transplant, no.	51
Unilateral of lung transplant, no.	49
APACHE II score, median (IQR)	19 (16-23)
Respiratory infection, no.	39
Rejection, no.	29
T0 Grade 3 PGD, no.	50
T24 Grade 3 PGD, no.	33
T48 Grade 3 PGD, no.	25
T72 Grade 3 PGD, no.	22
ICU mortality, no.	9

## P267 New molecular biomarkers of acute respiratory distress syndrome in abdominal sepsis

### A. K. Kuzovlev^1^, V. Moroz^1^, A. Goloubev^1^, S. Polovnikov^2^, S. Nenchuk^2^

#### ^1^V.A. Negovsky Research Institute of General Reanimatology, Moscow, Russia; ^2^NN Burdenko Main Military Hospital, Moscow, Russia

**Introductions:** Acute respiratory distress syndrome (ARDS) is a common complication of nosocomial pneumonia (NP) in patients with abdominal sepsis. The aim of this study was to investigate into new molecular biomarkers of ARDS in abdominal sepsis.

**Methods:** The observational study in ICU ventilated septic patients with peritonitis (76 %) and pancreonecrosis (24 %) was performed in 2010 and 2015. ARDS was diagnosed and staged according to the V.A. Negovsky Research Institute criteria and the Berlin definition. Plasma surfactant protein A and D (SPD, SPA) were measured on day 0, 3, 5 by the immunoenzyme essay (BioVendor, USA). Patients were treated according to the international guidelines. Data were statistically analyzed by STATISTICA 7.0, ANOVA and presented as median and 25-75th percentiles (ng/ml); p < 0.05 was considered statistically significant. Areas under the receiver operating curves were calculated.

**Results:** One hundred patients (out of 783 screened) were enrolled in the study according to the inclusion/exclusion criteria. Patients were assigned into groups: NP + ARDS (n = 55, 45 ± 5.1 years old, M/F 50/5, mortality 29,0 %) and NP (n = 45, 42 ± 5.8 years old, M/F 41/4, mortality 26,6 %). Groups were comparable in APACHE II and SOFA scores on the baseline. In the NP + ARDS group SPD was higher at all points than in the NP group. Plasma SPD on day 0 > 111.2 ng/ml yielded a sensitivity of 68.2 % and specificity of 92.3 % (AUC 0.85; 95 % CI 0.684-0.945; p < 0.0001) for diagnosing ARDS in NP. A complex ROC analysis (SPD + P/F ratio <280 + EVLWI >8.3 ml/kg) yielded a better diagnostic accuracy of SPD: cutoff >93.7 ng/ml, sensitivity 81.0 %, specificity 100.0 % (AUC 0.96; 95 % CI 0.817-0.998; p < 0.0001). SPA on day 0 is not informative for ARDS diagnosis (SPA > 36.2 ng/ml, sensitivity of 51.2 %, specificity of 92.3 %, AUC 0.61; 95 % CI 0.474-0.745; p < 0.1168). Plasma SPA was significantly lower in survived versus died patients with ARDS. Plasma SPA on day 0 > =18,6 ng/ml yielded a sensitivity of 88,9 % and specificity of 72 > 7 % for prediction of mortality in ARDS patients on day 5-6 of ICU stay (AUC 0.86; 95 % CI 0.685-0.962; p < 0,0001).

**Conclusions:** Thus, in abdominal sepsis patients with NP and ARDS new molecular biomarkers can be used for ARDS diagnosis (SPD) and prognostification of outcomes (SPA).

## P268 Tight junction’s proteins claudin -5 and regulation by tnf in experimental murine lung injury model of ali/ards

### V. Karavana^1^, C. Glynos^1^, A. Asimakos^1^, K. Pappas^1^, C. Vrettou ^1^, M. Magkou ^1^, E. Ischaki^1^, G. Stathopoulos^2^, S. Zakynthinos^1^

#### ^1^George P. Livanos and Marianthi Simou Laboratories, Athens, Greece; ^2^University of Patras, Rio, Achaia, Greece

**Introduction:** Acute Lung Injury and acute respiratory distress syndrome (ALI/ARDS) is characterized by tight junction’s (TJ’s) barrier disruption and changes in the composition and integrity of Claudins (CLDN) which are components of TJ’s. CLDN5 is highly expressed and augment permeability in lung parenchyma, although the regulation by inflammatory stimuli remains elusive. This study aims to investigate CLDN5 expression and regulation by TNF, in LPS-induced ALI/ARDS in mice.

**Methods:** Lung injury was induced by intratracheal LPS administration in adult male C57BL/6, and TNF-/- mice. Mice underwent bronchoalveolar lavage (BAL) and sacrificed at 6 h and 24 h after LPS administration. Histological score of ALI was assessed in lung tissues. CLDN5 cellular localization was evaluated by immunohistochemistry, while its expression levels were verified by Western blotting.

**Results:** In mice lung tissue, following LPS administration, CLDN5 immunoreactivity was distributed along the alveolar epithelium and vascular endothelium. CLDN5 protein expression levels was increased by 3 fold along with ALI characteristics (augmented in BALF cellularity, protein content and ALI score). In TNF-/- mice, CLDN5 expression is statistically reduced as early as 6 h after LPS administration with consequently increased in BALF protein content. In addition increased CLDN5 expression was observed upon 24 h LPS administration. TNF biphasic action on CLDN5 expression was dependent on ERK1/2 expression levels.

**Conclusions:** In LPS induced ALI/ARDS in mice, CLDN5 was increased associated with ALI severity. Moreover, TNF regulates CLDN5 expression, and TJs permeability dependent in part on ERK1/2 pathway. Our findings emerge the role of Claudins and especially CLDN5 to elucidate the pathophysiology of this form of injury.

## P269 Cell counts in endobronchial aspirate to assess airway inflammation in ARDS patients: a pilot study

### S. Spadaro^1^, I. Kozhevnikova^1^, F. Dalla Corte^1^, S. Grasso^2^, P. Casolari^1^, G. Caramori^1^, C. Volta^1^

#### ^1^University of Ferrara, Ferrara, Italy; ^2^University of Bari, Bari, Italy

**Introductions:** Adult respiratory distress syndrome (ARDS) is characterized by a breakdown of capillary integrity, leading to the leakage of protein-rich fluid and the accumulation of inflammatory cells within the airspaces. Bronchoalveolar lavage (BAL) is the commonly used method to obtain lower respiratory tracts samples and to define the role of cells in the inflammatory response in ARDS. However, there is the need to assess the feasibility of using relatively non-invasive endotracheal aspirate (EASP) techniques for cytological studies of the lower respiratory tract in ARDS patients.

**Methods:** EASP samples were taken from 25 ICU mechanically ventilated ARDS patients, at <36 h, <6d, and >6d from admission. Total cell counts/ml were determined in a Neubauer chamber and differential cell counts were performed on a cytospin preparation stained with Diff-Quick.

**Results:** The total number of cells in all ARDS EASP samples was 89 ± 25x104/ml; median cell viability 89 [80–95]%. A correlation was found between neutrophil counts in blood and in the EASP specimens (ρ =0.42 p = 0.04).

Total cell counting tended to be lower within 36 h samples, but not statistically different from sampling at other times. In subjects with bacterial infection and ARDS, high neutrophil percentage was found up to 6d (95[82–98]%); the percentage slightly decreased at >6d (85[74–97]%).

Macrophage percentage had a trend for being higher in ARDS patients with a positive bacterial culture compared to those with a negative one (15[5–27]vs5[3–12];p = 0.066).

**Fig. 73 (Abstract P269).**
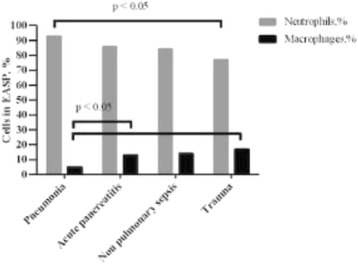
ᅟ

Macrophage and neutrophil percentage according to ARDS etiology are reported in Fig. 73.

**Conclusions:** EASP analysis found an increase in neutrophils in bacterial infection vs non-infective ARDS patients. In addition, different risk factors for ARDS were associated with different percentage of macrophages and neutrophils in EASP. Following our preliminary findings, further studies are required to assess if monitoring EASP cellularity over time could be useful to evaluate prognosis in ARDS.

## P270 Epidemiological and clinical profile of patients with acute respiratory distress syndrome in the surgical intensive care unit surgical, hospital JRA, Antananarivo

### T. Andrianjafiarinoa, T. Randriamandrato, T. Rajaonera

#### CHU HJRA, Antananarivo, Madagascar

**Introduction:** Acute respiratory distress syndrome (ARDS) is a very common situation for patients hospitalized in intensive care. It is associated with a high morbidity and mortality rates and the management is always a great challenge for intensive care. The objective of this study is to describe the epidemiological and clinical profile of patients with ARDS in the surgical ICU of the University Hospital Joseph Ravoahangy Andrianavalona JRA Antananarivo Madagascar

**Methods:** This study of twelve months was led from January to December 2014 in the surgical ICU of JRA Hospital Antananarivo. It included all patients with clinical and radiological signs of ARDS. Age, gender, clinical and radiological signs, treatment and outcome of the patients were considered.

**Results:** Twenty-six patients were identified, that means 1.74 % of patients hospitalized in intensive care during the study period. The average age was 56 years with a sex ratio M / F 1.16. Of these patients, 62 % had a history of ethyl and smoking, 8 % had asthma and 30 % had no particular experience. In 69 % of cases, ARDS was present at intake and is a reason for hospitalization. In ARDS, 31 % of patients had hemodynamic shock with failure and 15 % of them were associated with liver failure. All patients were put on antibiotics treatment and 85 % of them were on mechanical ventilation. The mortality rate was high 81.61 % in a context of multiple visceral failure.

**Conclusions:** In this study, ARDS affects mainly young patients with respiratory history. The management is very heavy especially in a country like ours because of the lack of materials and particularly monitoring. The mortality rate from ARDS remains high. Currently the development of the prone technique and body extra support may improve the ARDS mortality rate.

**References**

Younsuck Koh. Update in acute respiratory distress syndrome. Koh Journal of Intensive Care 2014,2:2

Eric Kipnis.SDRA Aspects pratiques > Journees Lilloises d Anesthesie Reanimation 2014

## P271 Effect of high PEEP after recruitment maneuver on right ventricular function in ARDS. Is it good for the lung and for the heart?

### S. El-Dash^1^, ELV Costa^2^, MR Tucci^2^, F Leleu^1^, L Kontar^1^, B. De Cagny^1^, F. Brazier^1^, D. Titeca^1^, G. Bacari-Risal^1^, J. Maizel^1^, M. Amato^2^, M. Slama^1^

#### ^1^Réanimation médicale, Centre Hospitalier Universitaire, Amiens, France; ^2^ Cardio-Pulmonary Department, Pulmonary Division, Heart Institute (Incor), University of São Paulo, Brazil

**Introduction:** Protective ventilatory strategies for ARDS with lower tidal volumes and higher PEEPs may benefit the lung. But high PEEP may induce right ventricular (RV) dysfunction which may severely impair hemodynamic. We hypothesized that opening the lung with recruitment maneuver, followed by maintaining high PEEP may be good for the lung and for the heart.

**Methods:** We included ARDS patients (P/F < 200) under mechanical ventilation sedated and paralyzed. An open-lung protocol consisting of a 4-step recruitment maneuver (from 25 to 40 cm of PEEP) and a decremental PEEP titration (to find the best PEEP named open-lung PEEP) was performed after ruling out hypovolemia. We recorded mean arterial pressure (MAP), cardiac output (CO), right-to-left ventricle diameter ratio (RV/LV) using echocardiography, tricuspid annular plane systolic excursion (TAPSE), aortic velocity time integral (VTI), systolic lateral RV annulus velocity (S) during the recruitment maneuver at 40 cmH2O and 1 hour and 24 hours after this maneuver at the chosen best PEEP (open-lung PEEP).

**Results:** 10 patients with ARDS (age 55 ± 13, SAPS 70 ± 15) were included. The median PEEP increased from 9 (6.0 – 13.5) cmH2O at baseline to 21.0 cmH2O (16.0 – 23.0) (p < 0.01) at openlung PEEP. PO2/FiO2 increased from 70.6 (40.6 – 89.6) to 209 (141 – 229), p < 0.01. RV/LV increased from 0.70 [0.58 – 0.75] to 0.80 [0.73 – 0.83], p = 0.02, during PEEP of 40cmH2O and returned to baseline values (0.75 [0.63 – 0.89], p = 0.10) at open-lung PEEP. The TAPSE decreased from 18 [17 – 21]mm to 16 [15 – 17]mm (p = 0.02) at a peep of 40 cmH2O, returned to baseline 18 [17 – 19]mm at open-lung and remained stable 24 hours later. S presented the same pattern. MAP dropped from 89 [73– 95]mmHg at baseline to 70[60– 81]mmHg (p = 0,01 vs baseline) during the last step of recruitment, was 70 [69–77]mmHg at open-lung PEEP, and was 79[67– 84]mmHg 24 hours later. CO decreased from 5.9 l.min-1 (IQR 4.6 – 7.3) to 4.4 l.min-1 (IQR 3.5 – 6.4) during recruitment but returned to baseline value at openlung PEEP (5.6 l.min-1 (IQR 4.4 – 7.1)) and remained stable at 24 hours. Ejection fraction was unchanged at open-lung PEEP and at 24 hours, compared with baseline value.

**Conclusions:** In our ARDS patients, recruitment maneuver permitted to open the lung with an increase of P/F and apply high PEEP of 21 cmH20 to maintain the lung open. The right ventricular function was not impaired, confirming that what is good for the lung may be not deleterious for the heart.

## P272 Effect of recruitment maneuver on left ventricular systolic strain

### P. Mercado, J. Maizel, L. Kontar, D. Titeca, F. Brazier, A. Riviere, M. Joris, T. Soupison, B. De Cagny, S. El Dash, M. Slama

#### CHU Amiens, Amiens, France

**Introduction:** In ARDS patients under mechanical ventilation recruitment maneuver may open the lung and improve blood oxygenation. During this maneuver cardiac dysfunction may occur. We decided to analyze using speckle tracking, the effect of recruitment maneuver on the left ventricular longitudinal strain (LVLS).

**Methods:** We included ARDS patients under mechanical ventilation sedated and paralyzed with P/F < 200. An open-lung protocol consisting of a 4-step recruitment maneuver (from 25 to 40 cm of PEEP) and a decremental PEEP titration (to find the best PEEP) was performed after ruling out hypovolemia and severe acidosis. We recorded clinical parameters (mean arterial pressure (MAP), heart rate (HR)) as well as ejection fraction (EF) using 2D echocardiography, mitral annular plane systolic excursion (MAPSE) with M-Mode echocardiography, aortic velocity time integral (using pulsed Doppler)(VTI), systolic lateral LV annulus velocity using spectral tissue Doppler imaging (S) and left ventricular longitudinal stress (LVLS) using speckle tracking at baseline, during the recruitment maneuver at 40 cmH2O and 2 minutes after this maneuver at the chosen best PEEP.

**Results:** 9 patients with ARDS (age 55 ± 13y/o) with SAPS 70 ± 15, P/F 130 ± 19, lung compliance 26 ± 8 ml/mmHg, PEEP 9 ± 3 cm H2O, respiratory rate 25 ± 5/mn, MAP 78 ± 12 mmHg, HR 85 ± 12/mn, EF 60 ± 18 %,VTI 22 ± 6 cm, MAPSE 1,3 ± 0,5 cm, S 8,4 ± 4,5 cm/s, were included. During recruitment at a PEEP of 40 cmH2O, HR 90 ± 14/mn and P/F176 ± 85 increased; MAP 62 ± 22 mmHg, MAPSE 1,3 ± 0,5 cm; S 8,4 ± 4,6 cm/s; EF 49 ± 22 %, VTI17 ± 8 cm all decreased. At the best PEEP of 12 ± 5cmH2O, P/F dramatically increase to 242 ± 80 and all hemodynamical and echocardiographic and Doppler parameters returned to the baseline values. LVLS was at -15,6 ± 5,6 % at baseline and dramatically increased to -8,1 ± 5,6 at a PEEP of 40cmH2O and returned to the baseline value of -13,8 ± 5 % immediately after the recruitment maneuver. This left longitudinal strain impairment during the maneuver was more severe at the apex (-19 ± 8 % vs -7 ± 8 %).

**Conclusions:** Recruitment maneuver induces transient LV systolic dysfunction particularly at the apex of the LV. This dysfunction maybe due to a transient myocardial ischemia and disappears immediately after this maneuver.

## P273 Inhaled nitric oxide – is switching supplier cost effective?

### Remmington, A. Fischer, S. Squire, M. Boichat

#### Royal Brompton Hospital, London, UK

**Introduction:** Updating equipment in Critical Care is important to improve practice but changing to a new service involves a significant amount of effort from initial planning to final launch. This study was designed to evaluate the stages of implementation, quantify the time spent and the cost of updating our nitric oxide delivery service. Nitric Oxide is one of the most expensive therapies currently used in our Hospital accounting for over 700,000 Euros (€) per annum. Estimated cost savings (€350,000) have to be weighed against clinical risk and the cost of implementing a new device.

**Methods:** Updating the nitric oxide delivery system was relevant to Adult (AICU) and Paediatric Intensive Care Units (PICU), Catheter Laboratories and Theatres. The implementation plan was studied to establish the number of hours and cost of each stage using meeting minutes, email correspondence, training records and working time equivalent calculations.

**Results:** 1. Testing equipment and trial of the new device on AICU and PICU: carried out by Clinical Engineering. Six months was spent testing and modifying the equipment so it was safe to use in a clinical setting and a further six months to find a suitable patient. Time spent 35 hours and cost = €5320.

2. Approval to switch: approved at a Clinical Practice Committee meeting and with Divisional Directors. Time spent = 7 hours and cost = €408.

3. Develop a costing model: data from our electronic prescribing system was used to establish the number of patients, hours of treatment and the volume of gas used in the financial year of 2014/15. This information was essential to calculate the number of cylinder refills, number of consumables required, and the cost of the rental for the machines and cylinders. These data were presented to Finance to highlight potential cost savings for switching delivery systems. Time spent = 36 hours and cost = €2,100.

4. Contract: a service level agreement for the new service was agreed between Pharmacy, Clinical Engineering, Procurement and the company. An agreement was made on number of machines and cylinders to be held on site, timescale for urgent deliveries of back-up machines and cylinders and annual service requirements. This was signed by the Director of Operations and the Director of Pharmacy. Time spent = 14 hours and cost = €532.

5. Training of staff: Training sessions to nurses and doctors of all grades for AICU amounted to 20 hours of time spent at a cost of €582. Training for PICU was 30 hours and cost €620. Training for Cath Labs and Theatres was 3.5 hours and cost €100.

**Conclusions:** This evaluation has shown that switching to a new nitric oxide delivery system was cost effective (€9662) but the time (145.5 hours), effort and clinical risk involved was huge. A carefully considered implementation plan was essential for a safe and successful switchover.

## P274 Epidemiological study of severe acute pancreatitis in Japan ; comparison of the etiology and the patient outcomes on 1159 patients.

### H. Honzawa^1^, H. Yasuda^1^, T. Adati^1^, S. Suzaki^1^, M. Horibe^2^, M. Sasaki^2^, M. Sanui^2^

#### ^1^Musashino Red Cross Hospital, Tokyo, Japan; ^2^JSEPTIC Clinical Trial Group, Tokyo, Japan

**Introduction:** Acute pancreatitis is known to have a variety of causes, but the specific death rate and severity for each cause remains unknown. Epidemiological studies of acute pancreatitis have been reported in China and Europe, but these have not included statistical analysis of factors affecting the outcomes of patients; thus, there is a need for examination.

**Methods:** The present study was a retrospective study. We studied cases of severe acute pancreatitis in 20 Japanese facilities from January 2009 to December 2013. The target cases were divided into four groups: gallstone, alcoholic, idiopathic, and other cases. We examined the relationship between patient outcomes by cause and various types of factors using statistical techniques.

**Results:** The total cases of severe acute pancreatitis (n = 1159) were divided into gallstone cases (n = 456), alcoholic cases (n = 456), idiopathic cases (n = 240), and other cases (n = 222). According to the comparison regarding the background of patients, young men were prone to be more in the alcoholic group. A comparison of patient outcomes revealed significant statistical differences for rates of administration of early enteral nutrition (gallstone 22.8 %, alcoholic 30.9 %, idiopathic 26.2 %, other 20.2 %, p = 0.014), mechanical ventilation management (31.5 %, 27.6 %, 37.9 %, 29.2 %, p = 0.043), and mortality (14.1 %, 8.1 %, 16.6 %, 17.5 %, p = 0.001) among the four groups.

We performed a multivariate analysis for death at the time of discharge from hospital and development of infection. The odds ratio for death at the time of discharge from hospital for patients who received early enteral nutrition was 0.48 (95 % CI 0.28 to 0.81). These results showed the different relationship among the groups. In the group of cause-specific cases, a similar trend was observed only in patients with gallstones.

The odds ratio for the development of infection for patients in the alcoholic group who received early enteral nutrition was 0.26 (95 % CI 0.10 to 0.63). In the other group, we couldn ft indicate the same tendency statistically.

**Conclusions:** The results of the present study suggest that the factors contributing to an improvement in outcome may differ according to the cause of severe acute pancreatitis. There might be a need to examine tailor-made treatments for severe acute pancreatitis according to cause.

## P275 Extracorporeal liver support therapy. Experience in an intensive care unit

### R. Marinho, J. Daniel, H. Miranda, A. Marinho

#### Centro Hospitalar do Porto, Porto, Portugal

**Introduction:** Severe liver failure is common and carries a high mortality risk in patients with both acute and acute-on-chronic liver failure. Extracorporeal liver support therapy (ECLST) could be one of the treatment option. But despite proven biochemical efficacy, there are little data regarding clinical end points. We show our experience for more than six years in our ICU.

**Methods:** Single-center retrospective observational study that enrolled patients with hepatic failure treated with ECLST, between November of 2006 and December of 2012. Data on demographics, clinical characteristics, SOFA and MELD were evaluated. Excel and GraphPad Prism were used for statistical analysis.

**Results:** 15 patients were enrolled, that underwent in total 22 sessions. 8 (53 %) were men, with an average age of 46 ± 14, mortality rate 53 %. 2 patients (13 %) were admitted due to acute liver failure (ACF) and the other 13 (87 %) due to acute-on-chronic liver failure (ACLF). The length of stay (LOS) was 12.40 ± 11.54 in the ICU and 35.13 ± 31.04 days in the hospital. SOFA and MELD score at admission were respectively 9.47 ± 3.66 and 30.13 ± 8.55; before the session 9.77 ± 4.29 and 31.2 ± 9.1; after the session 9.14 ± 3.91 (p = 0.14) and 30.91 ± 5.81 (p = 0.514). After ECLST there was a decrease in total bilirubin (24.99 ± 5.61 mg/dL to 15.15 ± 4.38 mg/dL, p < 0.0001), creatinine (1.01 ± 1.044 mg/dL to 0.63 ± 0.732 mg/dL, p < 0.001), BUN (49.59 ± 40.95 mg/dL to 25.14 ± 20.89 mg/dL, p <0.0001) and platelets (69.000 ± 50.800/mm3 to 54.000 ± 34.800/mm3, p <0.005). The global mortality rate was 60 %. 33 % of the patients (5) were submitted to a liver transplante, with a mortality rate of 0 %, wether the mortality rate in the patients without liver transplant was 90 %.

**Conclusions:** ACF and ACFL have high mortality rate without liver transplant, although extracorporeal liver support therapy improved total bilirubin, creatinine and BUN. Nevertheless, the value of extracorporeal liver support remains to be corroborated by further clinical studies that include the optimal timing, mode, intensity and duration of this treatment.

## P276 Accuracy of mortality prediction models in acute versus acute-on-chronic liver failure in the intensive care setting

### K. Milinis^1^, M. Cooper^2^, G. R. Williams^2^, E. McCarron^2^, S. Simants^2^, I. Patanwala^2^, I. Welters^1^

#### ^1^University of Liverpool, Liverpool, UK; ^2^Royal Liverpool University Hospital, Liverpool, UK

**Introduction:** Acute-on-chronic liver failure (ACLF) is a distinct syndrome from acute liver failure (ALF) characterised by acute decompensation, organ failure and high short-term mortality (50 %) [1]. Several novel measures (Consortium Liver Failure-SOFA (CLIF-SOFA) and Royal Free Hospital (RFH) scores) have been specifically developed to predict mortality in ACLF, however their validity has not been assessed in ALF [1]. The aim of this study is to compare the performance of mortality prediction models in ALF and ACLF in the intensive care unit (ICU) setting.

**Methods:** Retrospective data were collected on patients admitted with liver failure to ICU between 2008 and 2014. Demographic, clinical and biochemical parameters were recorded on admission to ICU. The main outcome was death in ICU. The scores were calculated for CLIF-Organ failure (CLIF-C OF, a shortened version of CLIF-SOFA), CLIF-ACLF (includes age and white cell count), RFH, Model for End-Stage Liver Disease (MELD) and Acute Physiology and Chronic Health Evaluation (APACHE II). Accuracy and calibration of the scoring systems were assessed using area under the receiver operator curve (AUROC) and goodness-of-fit χ respectively.

**Results:** 220 patients were included in the study (ACLF = 118, ALF = 102). The main aetiology of cirrhosis in ACLF group was alcohol (91.5 %). The leading causes of ALF were paracetamol overdose (48 %), alcoholic hepatitis (31.4 %) and viral hepatitis (11.8 %). ACLF patients were significantly more likely to have longer stay (median 6 vs. 3 days, p < 0.005) and higher risk of dying (54.2 % vs. 33.7 %, χ <0.05). In general, the scoring systems performed better in ALF (AUROC values: CLIF-OF 0.77; CLIF-ACLF 0.79; RFH 0.80; MELD 0.80; APACHEII 0.76) compared to ACLF (AUROC values: CLIF-OF 0.68; CLIF-ACLF 0.68; RFH 0.72; APACHEII 0.65; MELD 0.71). Calibration of the models was acceptable across the both groups, except for APACHEII in ALF patients (χ =14.2, p < 0.05).

**Conclusions:** Mortality and morbidity is higher in ACLF compared to ALF. Prediction models developed in ACLF populations perform even better in ALF. This could be possibly explained by relatively few comorbidities in ALF. In our patient cohort, the highest accuracy was achieved using RHF and MELD scores rather than CLIF-SOFA based measures.

**Reference**

[1] R. Jalan et al: J. Hepatol., vol. 61, no. 5, pp. 1038–1047, 2014.

## P277 Risk of coronary artery disease in patients with chronic liver disease: a population based cohort study

### Y. Su

#### Dalin Tzu Chi Hospital, Buddhist Tzu Chi Medical Foundation, Chiayi County, Taiwan

**Introduction:** It is known that the risk of hemorrhage in patients with chronic liver disease is higher compared with general population. However, the evidences between chronic liver disease and coronary artery disease are inconclusive. We investigated the diagnosis of coronary artery disease in patients with chronic liver disease in Taiwan to evaluate if there is a higher risk compared to the general population.

**Methods:** We utilized a sampled National Health Insurance claims database containing one million beneficiaries. We followed all adult beneficiaries older than 18 years from January 1, 2007 till December 31, 2010 to see if they were diagnosed with coronary artery disease. We further identified patients with liver disease and compared their risk of coronary artery disease with the general population.

**Results:** We identified 28,511 patients with chronic liver disease and 728,285 patients without. After controlling for age, gender, urbanization level, socioeconomic status, diabetes, hypertension, hyperlipidemia, malignancies, heart failure, atrial fibrillation, smoking, obesity, peripheral artery disease and Charlson Comorbidity Index score, the adjusted hazard ratio was 0.74 (95 % confidence interval, 0.65¡X0.84) in patients with chronic liver disease. In subgroup analyses of cirrhotic patients with severe complications, the hazard ratio of coronary artery disease is much lower compared with general population, but the result is not statistically significant. (hazard ratio, 0.30; 95 % confidence interval, 0.04¡X2.14)

**Conclusions:** Chronic liver disease may be associated with decreased risk of coronary artery disease. However, no severity-effects associations can be observed.

## P278 20 years of liver transplantation in Santiago de Compostela (Spain). Experience review

### J. Fernández Villanueva, R. Fernández Garda, A. López Lago, E. Rodríguez Ruíz, R. Hernández Vaquero, S. Tomé Martínez de Rituerto, E. Varo Pérez

#### Hospital Clínico Universitario de Santiago de Compostela, Santiago de Compostela, Spain

**Introduction:** 809 Liver Trasplantations (LT) were made at Hospital Clínico Universitario de Santiago de Compostela (Spain) in the period 1994-2014, accounting 4.25 % of all LT performed in Spain (19005 cases, according to the Spanish Liver Transplant Registry- RETH). 20 years of experience review and comparison of Acumulated Surveillance Rates of our experience respect to the Spanish data provided by RETH.

**Methods:** Retrospective and descriptive study of 809 cases of LT performed from 1994 to 2014 at Hospital Clínico Universitario de Santiago de Compostela (Spain) based in our local LT Registration and in the National Spanish Registration- RETH.

**Results:** 809 LT cases, 12 of which were Hepatorenal Transplantations, 3 required a Re-Transplantation. Media of LT: 36 LT/year. Gender: 79.35 % were Male and 20.64 % Female with a Mean Age of 51 years old. Blood Group Predominancy: A positive (49 %) followed by the group 0 positive (39 %). The most frequent indication of TL was: Alcohol-related Cirrhosis (43 %), followed by Idiopathic (43.01 %) and Fulminant Hepatic Failure (6.18 %). The most common indication for Re-Transplantation was Hepatic Artery Thrombosis (34 %) followed by Primary Allograft Dysfunction (28.57 %). Mortality at 20 years: 34.86 %, the most frequent cause of Death was: Recurrence of Underlying Disease (28 % with a 80 % rate in Hepatitis C Virus Cirrhosis) followed by Bacterial/Fungal infections (20 %). The Acumulated Surveillance Rate at 20 years is 47 % in our series, higher in comparison with the overall data provided by RETH (37 % using Kaplan-Meier Curve p <0.01) and in the last five years (2008- 2013) is 77 %, higher compared with RETH data for the same period of time (70 % p <0.05). The Acumulated Surveillance Rate at 20 years based on LT Indication: 70 % in Fulminant Hepatic Failure group, 50 % in the Alcoholic Cirrhosis group and 35 % in Hepatitis C Virus Cirrhosis. In Re-transplantation group, Acumulated Surveillance Rate at five years (2008-2013) is just 23 %.

**Conclusions:** Alcohol-related Liver Disease, Hepatitis C Virus and Hepatocellular Carcinoma are the most frequent Indications of LT in the current Transplantation Program. Acumulated Sirveillance Rates are similar to the ones found in other Transplantation Programs. The main Cause of Death is Recurrence of the Underlying Disease, followed by Infectious and Cardiovascular Diseases. High Mortality Rates in Re-transplantation Group leads to a more careful selection of cases.

## P279 Diarrhea is a risk factor for liver injury and may lead to intestinal failure associated liver disease in critical illness

### N. Lefel, F. Schaap, D. Bergmans, S. Olde Damink, M. Van de Poll

#### MUMC, Maastricht, Netherlands

**Introduction:** We hypothesize that diarrhea can lead to intestinal failure associated liver disease (IFALD) in critical illness. Risk factors for liver injury in the ICU include toxicity of drugs and TPN, but etiology often remains unknown. Currently, there is emerging interest in the relation between intestinal failure and liver injury. The occurrence of IFALD is mostly attributed to the use of TPN in patients with intestinal failure, but recent data suggest that interruption of the enterohepatic cycle also may be an important cause. Diarrhea induces nutrient malabsorption but may also impair reabsorption of bile acids and disturbance of the gut-liver axis. Along this line of reasoning diarrhea may be a hitherto unidentified risk factor for liver injury through the development of IFALD.

**Methods:** We retrospectively analyzed patients admitted to our ICU from September 2014 until February 2015. Markers of liver injury and other clinical and biochemical parameters were recorded on the day diarrhea (>250 ml/day) developed (median at the 2d day of ICU admission) and 2 days thereafter. In patients without diarrhea data were recorded at the day of ICU admission and 2 days later. Patients with liver injury (defined as an elevation of serum y-glutamyltransferase or alkaline phosphatase levels above reference values) on admission or at the moment diarrhea occurred were excluded. 79 patients remained for analysis (19 with liver injury). Uni- and multivariate analyses were performed to identify risk factors for liver injury.

**Results:** In patients who developed liver injury the incidence of diarrhea (58 %) was higher than in control patients (22 %) p = 0.003, X2-test. Multivariate logistic regression analysis showed that diarrhea is an independent risk factor for the development of liver injury. (OR 4.1 (95 % CI 1.2-14.5), p = 0.028)

**Conclusions:** Diarrhea is an independent risk factor for the development of liver injury in the ICU. This supports the hypothesis that disturbance of the liver-gut axis due to diarrhea and bile salt malabsorption in ICU patients should be regarded as a manifestation of intestinal failure, potentially leading to intestinal failure associated liver disease. Future research should be aimed at unraveling mechanisms of IFALD in ICU patients and at interventions limiting malabsorption or its consequences for intestinal-hepatic bile salt signaling.

## P280 Bowel care on the intensive care unit: constipation guideline compliance and complications

### K. Tizard, C. Lister, L. Poole

#### Royal Liverpool University Hospital, Liverpool, UK

**Introduction:** Constipation in critically ill patients is a common problem and has been associated with a number of significant complications including prolonged mechanical ventilation, increased length of stay, compromise of intenstinal protective barrier and difficulty establishing enteral feeding [1].

Our intensive care unit (ICU) bowel care guideline specifies initiation of treatment for constipation, defined as a patient not opening their bowels for three consecutive days.

**Methods:** We retrospectively audited consecutive ICU patients over a two-months to establish the incidence of constipation as well as adherence to bowel care guideline. Patients were excluded if they had stayed on the ICU for less than three days, had undergone recent bowel surgery or were on an alternative bowel care protocol (e.g. encephalopathy, spinal or brain injury patients).

Data collected included bowel care prescriptions, rectal examinations, episodes of bowel opening and stool type.

**Results:** Of the 37 patients included, 43 % suffered a period of constipation during their ICU stay. Treatment for patients who had not had their bowels opened for three consecutive days was initiated in only 44 % of patients. Treatment strategies for constipation varied and were partially or fully compliant with guidelines in only 37.5 % of constipated patients. Despite similar APACHE II scores and ages across the two groups, the incidence of a patient having at least one episode of failure to feed via the enteral route occurred in a higher proportion of constipated patients (69 %) compared with non-constipated patients (35 %).

**Conclusions:** The incidence of constipation on our unit is decreasing, however it remains a common problem, which seems to impact on establishing enteral feeding. As it can cause potentially serious complications in critical care patients, compliance with the guideline should be improved. We have changed policy to include starting prophylactic laxatives [2], unless contraindicated, in all new admissions with earlier escalation of treatment. Continued education and promotion of the new guideline is ongoing in our unit.

**References**

1. Mostafa SM, Bhandari S, Richie G, et al. Constipation and its implications in the critically ill patients. Br J Anaesth 2003; 91:815-819.

2. Masri Y, Abubaker J, Ahmed R. Prophylactic use of laxative for constipation in critically ill patients. Ann Thorac Med. 2010 Oct-Dec; 5: 228–231.

## P281 Malnutrition assessed by phase angle determines outcomes in low risk cardiac surgery patients

### D. Ringaitiene ^1^, D. Gineityte^2^, V. Vicka^2^, I. Norkiene^1^, J. Sipylaite^1^

#### ^1^Vilnius University Hospital Santariskiu Clinics, Vilnius , Lithuania; ^2^Vilnius University, Faculty of Medicine, Vilnius, Lithuania

**Introduction:** Phase angle (PA), obtained from bioelectrical impedance analysis (BIA), is a non-invasive method, for measuring altered electrical properties of biological tissues. It has been recognised as an objective prognostic marker of disease severity and frailty. The aim of this study is to determine, whether phase angle is a marker of malnutrition and postoperative morbidity in low operative risk patients undergoing cardiac surgery.

**Methods:** A prospective study was conducted in a tertiary hospital. The nutrition state of cardiac surgery patients was evaluated using BIA the day before scheduled surgery. After applying selection criteria, 342 low operative risk patients were selected and classified into two groups in accordance to PA value: a low PA group and normal PA group. The correlation between low phase angle and low fat free mass index (FFMI), a marker of malnutrition, was assessed. Associations between low phase angle and adverse postoperative outcomes, defined by STS postoperative risk evaluation model, were analysed. The impact of low PA on length of stay in ICU and hospital was evaluated.

**Results:** Low PA was detected in 61 (17,8 %) patients in the selected group, which consisted of low operative risk patients with median Euroscore II value of 1,46 [IQR:0,97-2,03] and was associated with FFMI with Pearson’s R of 0,515 (p < 0,001). Low PA was associated with higher rates (13 (21,3 %) vs. 30 (10,7 %) p = 0,023) and risk of postoperative morbidity in univariate regression analysis (OR = 2,27,Cl95% = 1,10-4,66, p = 0,026). Furthermore, low PA persisted as an independent factor in multivariate regression analysis (OR = 2,50, CI95% 1,18-5,29, p = 0,016) adjusted for preoperative risk factors of postoperative morbidity. Evaluation of hospitalization length revealed tendency of a prolonged hospitalization (>14 days) rate (31 (50,8 %) vs. 105 (37,8 %), p = 0,063) in the group with low PA.

**Conclusions:** A low preoperative phase angle is an indicator of malnutrition and determines adverse outcomes after cardiac surgery. Further research is needed to evaluate clinical applications of the phase angle, such as a more accurate identification of malnourished cardiac surgery patients.

## P282 Preoperative fasting times in an irish hospital

### A. O’Loughlin, V. Maraj, J. Dowling

#### Mercy University Hospital, Cork, Ireland

**Introduction:** Perioperative fasting reduces the risk of aspiration of gastric contents. This risk is weighed against the risk of intraoperative hypotension and insulin resistance postoperatively. Guidelines have varied from the “nil-by-mouth-from-midnight” regime to the more liberal “2-4-6” regime, as per the Association of Anaesthetists of Great Britain and Ireland (AAGBI) and ESA guidelines, whereby unlimited clear fluids are allowed up to 2 hours preoperatively.

**Methods:** Adult patients were recruited prior to theatre on their day of surgery. Fasting times were calculated using a verbal questionnaire and noting the anaesthetic start time.

**Results:** All patients (100 %) exceeded the ESA fasting guidelines for solids, with some fasting for greater than 24 hours before an elective procedure. Fasting times for liquids were shorter, but on average were in excess of 2 hours.

**Conclusions:** Patients, irrespective of whether they arrive on the day of surgery or are inpatients fast for similar times, all longer than the ESA guidelines. Patients and their families, as well as hospital staff should be aware of current guidelines. Current protocols should be adjusted to improve compliance.

**References**

-ESA Guideline on Pre-operative fasting, European Journal of Anaesthesiology 2011

-Audit on preoperative fasting of elective surgical patients in an African academic medical center. World J Surg 2014

## P283 Costs and final outcome of early x delayed feeding in a private Brazil ICU

### M. B. Velasco , D. M. Dalcomune, E. B. Dias, S. L. Fernandes

#### Hospital Meridional S.A., Cariacica, Brazil

**Introduction:** ICU is a local where there is a great concern with cost-benefit ratio. Early enteral nutritional is recommended in cases of critically patients and started within 48 h of initial treatment, after hemodynamic stabilization. Early Feeding (EF) improves nutritional outcomes, reduces ICU and hospital death, while Delayed Feeding (DF) reduces feeding complications and ICU length stay. Patients survival improves total costs of care and reduces meaningfully in Early Feeding. Our study objectives is if the EF would have influence on costs, length stay, and final outcome in ICU and hospital when compared with the DF.

**Methods:** This study was in a 50 particular hospital ICU beds in ES-Brazil. 151 critically patients with nutritional therapy indication were enrolled. This retrospective study (nov13-nov14) we grouped as EF, until 48 h admission, and DF, after 48 h. We compared SAPS3, lenght stay, direct costs (materials, medicines, gas and hospital fees), and outcome. Medical records were extracted from the MVsystem and Epimed Software. Differences between the groups was studied using the Student-t for independent samples and the Ztest for two proportions was used to assess the outcome. Significance level was 5 % and 95 % confidence interval.

**Results:** We evaluated 151 patients, 56.1 % male, age 59.9yo ± 19, SAPS3 44.1 ± 13.7. EF ICU and hospital length stay: 7.3d ± 11.2 and 21.0d ± 40.5; EF ICU and hospital daily cost: US$4,758 ± 22,006 and US$863 ± 620; EF ICU and hospital discharge: 75.2 % and 64.8 %; EF ICU and hospital death: 24.8 % and 35.2 %. DF ICU and hospital length stay: 8.1d ± 6.3 and 17.9d ± 11.7; DF ICU and hospital daily cost: US$2,024 ± 3,363 and US$893 ± 500; DF ICU and hospital discharge: 45.7 % and 54.3 %; DF ICU and hospital death: 54.3 % and 45.7 %. SAPS3, lenght stay, costs and hospital death had no statistical difference. EF received more discharge from the ICU and hospital (p < 0.001). EF showed less ICU deaths (p < 0.001).

**Conclusions:** The cost and length stay (ICU and hospital) showed no statistical difference. There were improvement in the ICU mortality and length stay in ICU and hospital. Although the ICU mortality showed difference, the hospital death had no considerable variations.

**References**

1). Huang HH et al; Association between illness severity and timing of initial enteral feeding in critically ill patients: a retrospective observational study. Nutr J. 2012 May 3;11:30

2). Doig GS et al; Early enteral nutrition in critical illness: a full economic analysis using US costs. Clinico econ Outcomes Res. 2013 Aug 23;5:429-36

## P284 Can ventilator derived energy expenditure measurements replace indirect calorimetry?

### T. Oshima, S. Graf, C. Heidegger, L. Genton, V. Karsegard, Y. Dupertuis, C. Pichard

#### Geneva Universtiy Hospital, Geneva, Switzerland

**Introduction:** A recent paper suggests that energy expenditure (EE) can be calculated from ventilator derived CO2 measurements EEVCO2 and a fixed respiratory quotient (RQfixed) [1]. However, this method can introduce clinically significant errors when applied to individual patient measurements.

**Methods:** 261 unselected mechanically ventilated patients with EE measured by indirect calorimetry were analyzed. EE was compared with calculated EEVCO2 using measured VCO2 and RQfixed = 0.85. The clinically acceptable accuracy level for EEVCO2 was set at ±5 %. Equations for the calculation of EE and EEVCO2 are shown on Table 37. Since 5 % variation of EEVCO2 corresponded to ±0.05 of RQfixed, we compared the EEVCO2 and EE among patients with measured RQ lower, matching, or higher than this range. Statistical analysis was conducted by paired t-test of EEVCO2 and EE, and bias was determined from the mean difference (kcal/d). Actual 5 % accuracy rates of EEVCO2 for each group were also calculated. All analyses were conducted on SPSS statistics version 22 (IBM, USA).

**Results:** Overall bias for EEVCO2 was +24 (kcal/d), but only 40 % presented 5 % accuracy compared to EE. 46 % of the patients had matching level RQ, but 5 % accuracy rate was 57 %. The level of bias was clinically unacceptable for patients with lower or higher RQ [Table 38].

**Conclusions:** Energy expenditure calculated from ventilator derived measurements is acceptable for only 40 % of the patient population, and large bias can be overlooked if results are not verified by indirect calorimetry. EE derived from ventilator measurements should not be considered equal to EE measured by indirect calorimetry.

**Reference**

1. Stapel et al.: Critical Care 2015; 19: 370

**Table 37 (Abstract P284).** Equations for calculating energy expenditure (EE) and EEVCO2

EE = 1.44 × [3.941 × VO2(ml/min) + 1.11 × VCO2(ml/min)]
Respiratory quotient (RQ) = VCO2/VO2
EEVCO2 = 1.44 × [3.941 × VCO2/RQfixed + 1.11 × VCO2]

**Table 38 (Abstract P284).** EEVCO2 bias vs. measured EE (Matching RQ : RQ fixed ±0.05 (0.80 ~ 0.90))

N (%)	EEVCO2 Bias (kcal/day)	p value	Overall
261 (100)	+24	.029	Lower RQ
78 (30)	-166	.000	Matching RQ
119 (46)	+51	.000	Higher RQ
64 (24)	+203	.000	

## P285 Revisiting the refeeding syndrome: results of a systematic review

### N. Friedli^1^, Z. Stanga^2^, B. Mueller^1^, P. Schuetz^1^

#### ^1^Medical University Department, Kantonsspital Aarau, Aarau, Switzerland; ^2^Department of Endocrinology, Diabetes and Clinical NutritionUniversity Hospital Bern, Bern, Switzerland

**Introduction:** Although described more than 70 years ago, the refeeding syndrome remains understudied with lack of a standardized definition and treatment recommendations. The aim of this systematic review was to provide evidence in regard to a standardized definition, incidence rate and time course of occurrence, association with adverse clinical outcomes, risk factors and therapeutic strategies to prevent or treat this condition.

**Methods:** We searched MEDLINE and EMBASE for interventional and observational clinical trials focusing on refeeding syndrome, excluding case reports and reviews. We extracted data based on a predefined case report form including bias assessment.

**Results:** Out of 2206 potential abstracts, 44 records with a total of 6269 patients were included in the final analysis including 2 interventional studies and 16 studies including anorexic patients. Definitions for refeeding syndrome were highly heterogenous with most studies relying on electrolyte disturbances only and others also including clinical symptoms. Incidence rates varied between 0 % and 80 % depending on the definition and patient population. Occurrence was mostly within the first 72 hours of start of therapy. Most of the risk factors are in accordance with the NICE guidelines, but older age and enteral feeding were additionally described. Associations of refeeding syndrome and adverse outcomes remain unclear, as does the effect of preventive measures and treatment algorithms.

**Conclusions:** Although there is consensus in regard to risk factors and timing of the occurrence of refeeding syndrome, there is wide variation in definition, reported incidence rates and management recommendations (preventive measures and treatment). Further research is warranted to fill this gap.

## P286 Compliance with the new protocol for parenteral nutrition in our ICU

### L. Vandersteen, B. Stessel, S. Evers, A. Van Assche, L. Jamaer, J. Dubois

#### Jessa Ziekenhuis, Hasselt, Belgium

**Introduction**

After participating in the EPaNIC trial, we changed the protocol for the administration of parenteral nutrition (PN) in our ICU [1]. The administration of PN supplementing enteral nutrition (EN) in case of insufficient caloric intake, was postponed from day 3 after ICU admission to day 8, compared to the “late-PN-group” in the EPaNIC trial. One of the exceptions is patients with a BMI less than 18, where starting PN depends on the individual decision of the attending intensive care physician. The protocol for the administration of EN and glycaemic control remained unchanged. Micro-nutrients intravenously are prescribed for all patients as from day 3 after ICU admission in case of inadequate caloric intake. Two years after implementing the new protocol, we conducted a survey investigating the compliance with the new protocol.

**Methods**

We conducted a retrospective analysis of all patients admitted to a 6-bed medical unit of our ICU from 01/04/2013 to 30/11/2013. From 220 admitted patients, 123 were considered critically ill since they received tight glycaemic control. The other patients were excluded from the analysis since they stayed for less than 24 hrs in the ICU, mostly for monitoring purposes.

**Results**

Only 6 out of 123 patients received PN (4.88 %). In 4 cases PN was started at day 8 after ICU admission in accordance to the new protocol. PN was started earlier in 2 patients suffering from an haematological-oncological disease complicated with neutropenic sepsis and a BMI less than 18 and already receiving PN on the ward. Since a caloric intake less than ¾ of the caloric target at day 3 after ICU-admission was considered a cut-off for starting PN in the old protocol, 88.62 % of the patients would have met the criteria for receiving PN according to that protocol.

**Conclusions**

There is a good compliance to the revised protocol where PN is only started at day 8 after ICU admission in our medical ICU unit, besides some predefined exceptions.

This significantly reduces the number of patients receiving PN compared to the old protocol.

**Reference**

1. Casaer MP, Mesotten D, Hermans G, Wouters PJ, Schetz M, Meyfroidt G, et al. Early versus late parenteral nutrition in critically ill adults. N Engl J Med.2011; 365(6): 506-517.

## P287 Nutrition may be another treatment in the intensive care unit where less is more?

### R. Marinho^1^, H. Castro^1^, J. Moura^2^, J. Valente^3^, P. Martins^4^, P. Casteloes^5^, C. Magalhaes^6^, S. Cabral^7^, M. Santos^8^, B. Oliveira^8^, A. Salgueiro^4^, A. Marinho^1^

#### ^1^Centro Hospitalar do Porto, Porto, Portugal; ^2^Unidade Local de Saude do Alto Minho, Viana do Castelo, Portugal; ^3^Unidade de Saude Local de Castelo Branco, Castelo Branco, Portugal; ^4^Centro Hospitalar e Universitario de Coimbra, Coimbra, Portugal; ^5^Centro Hospitalar de Vila Nova de Gaia, Vila Nova de Gaia, Portugal; ^6^Centro Hospitalar do Algarve, Faro, Portugal; ^7^Instituto Portugues de Oncologia do Porto, Porto, Portugal; ^8^Faculdade de Ciencias da Nutricao e Alimentacao da Universidade do Porto, Porto, Portugal

**Introduction**

Several studies have shown that exclusive enteral nutrition is mostly ineffective in providing adequate energy intake in critically ill patients. For this reason, ESPEN reviewed its guidelines in 2009 in an attempt to improve this practice by recommending that all patients, who are receiving less than their targeted enteral feeding after 2 days should be considered for parenteral nutrition.

The aim of this study is to evaluate whether Portuguese ICUs changed their nutritional strategy according to ESPENs guidelines.

**Methods**

Observational prospective study, conducted in eleven Portuguese ICUs of nine general hospitals. Patients with 18 years of age or older were eligible if they were ventilated and had a length of stay (LOS) in ICU greater than 7 days. Demographic data, along with the energetic intake in the first 7 to 10 days and type of nutritional support used were collected from the selected patients.

**Results**

130 patients were enrolled, 63.8 % were male, the median age - 64 ± 16 (19Ð91), median BMI Ð 27.9 ± 5.9 (18.8Ð49), ICU LOS Ð 15.4 ± 6,1 days, mortality rate of 26.9 % (35). 70 % of patients were admitted for medical reasons, 31.5 % had normal weight, and the remaining patients were either overweight or obese. Energy intake in the first 10 days was 12.4 ± 6.6 Kcal/Kg/day (0Ð34.1); excluding the first 3 days it rose to 15.6 ± 7.4Kcal/Kg/day (0Ð34.1). Nutritional support was evaluated over a period of 1,223 days in which 80 % of the days patients received nutritional support, 66 % by enteral route and 14 % by parenteral route.

**Conclusions**

Energy needs in a critically ill patient can vary between 20-25 Kcal/kg/day. In our study a large proportion of Portuguese ICUs patients receive inadequate nutrition. Recent randomized clinical trials support the recommendation that “less nutrition is better”. However, it is not uncommon for critically ill patients to be starved and this is deleterious and should be avoided.

## P288 Should we provide more protein to critically ill patients?

### R. Marinho^1^, M. Santos^2^, E. Lafuente^3^, H. Castro^1^, S. Cabral^4^, J. Moura^5^, P. Martins^6^, B. Oliveira^2^, A. Salgueiro^6^, S. Duarte^7^, S. Castro^8^, M. Melo^9^, P. Casteloes^10^, A. Marinho^1^

#### ^1^Centro Hospitalar do Porto, Porto, Portugal; ^2^Faculdade de Ciencias da Nutrição e Alimentação da Universidade do Porto, Porto, Portugal; ^3^Centro Hospitalar Tamega e Sousa, Penafiel, Portugal; ^4^Instituto Português de Oncologia do Porto, Porto, Portugal; ^5^Unidade Local de Saude do Alto Minho, Viana do Castelo, Portugal; ^6^Centro Hospitalar e Universitário de Coimbra, Coimbra, Portugal; ^7^Unidade de Saude Local de Castelo Branco, Castelo Branco, Portugal; ^8^Centro Hospitalar do Algarve, Faro, Portugal; ^9^Centro Hospitalar do Baixo Vouga, Aveiro, Portugal; ^10^Centro Hospitalar de Vila Nova de Gaia, Vila Nova de Gaia, Portugal

**Introduction**

Appropriate nutrition delivery in the ICU is a topical issue. Most studies have focused on energy supply by enteral or parenteral nutrition. A few studies now also focused on protein supply. Studies also agree on the importance of adequate protein supply, 1.2-2.0 g/kg/day, for the outcome.

The aim of this study is to evaluate the amount of protein supply given to critically ill patients in Portuguese ICUs

**Methods**

Observational prospective study, conducted in eleven Portuguese ICUs of nine general hospitals. Patients with 18 years of age or older were eligible if they were ventilated and had a length of stay (LOS) in ICU greater than 7 days. Demographic data, along with the energetic intake in the first 7 to 10 days and type of nutritional support used were collected.

**Results**

130 patients were enrolled, 63.8 % were male, the median age - 64 ± 16 (19Ð91), median BMI Ð 27.9 ± 5,9 (18.8Ð49), ICU LOS Ð 15.4 ± 6,1 days, mortality rate of 26.9 % (35). 70 % of patients were admitted for medical reasons, 31.5 % had normal weight, and the remaining patients were either overweight or obese.

The energy supply in the first 10 days was 12,4 ± 6,6 Kcal/Kg/day (0Ð34,1); Carbohydrates are the main source of calories - 1,5 ± 0,93 g/kg/day (0Ð4,2), lipids - 0,4 ± 0,3 g/kg/day (0Ð1.37), protein/amino acid administration were - 0,32 ± 0,27 g/kg/day (0Ð1.11). Regarding additional supplements besides artificial nutrition, the impact on overall energetic intake of propofol was 1 ± 0.6 Kcal /Kg/day (0-9.7), while the amount for glucose solution was 1.3 ± 1 Kcal /Kg/day (0Ð8).

**Conclusions**

We may safely conclude that less calories is better in the ICU but scientific recognition of the importance of protein is growing, and although optimal protein dosing studies are not available, expert opinion supports administering in excess of 1.2 g/kg/day. At present, in most of the Portuguese ICUs, the majority of critically ill patients receive less than half of the recommended protein intake.

## P289 Protein provision in an adult intensive care unit

### S. Gray

#### Nottingham University Hospital NHS Trust, Nottingham, UK

**Introduction**

An audit was carried out to determine the amount of protein, in grams per kilogram body weight per day (g/kgBW/d), being provided to adult, Level 3, critically ill patients. International evidence based guidelines recommend 1.2 g/kgBW/d [1]. Protein is an integral component of nutritional therapy in critically ill patients which aims to minimise muscle wasting [2].

**Methods**

A prospective Audit was carried out in 2015 in a 21 bedded, Level 3, Adult Intensive Care Unit within a University Teaching Hospital and Major Trauma Centre. Inclusion criteria included all patients who were enterally fed. Exclusion criteria included parenteral nutrition, less than 4 days of enteral nutrition and incomplete data. Protein provision was measured against 1.2 g/kgBW/d. Propofol was not recorded which was a limitation of the Audit. The Audit was not subject to ethical approval according to Trust standards.

**Results**

Of 308 admissions, 111 patients were audited, 27 excluded and 84 patients included within data analysis (n = 84). Mean protein provision was 0.87 g/kgBW/d. Mean protein percentage delivery was 78 %. Protein deficits became more pronounced when measuring against ‘higher’ disease specific recommendations. Feeding protocols were adhered to in 6 % of patients. At initial assessment the nutritional products met protein in 55 % of patients. On average, patients experienced 20 hours of enteral nutrition stoppages.

**Conclusions**

Patients received a mean of 0.87 g/kgBW/d of protein compared to the minimum of 1.2 g recommended by international guidelines. There were wide variations of protein delivery between patient groups. Reasons for not meeting protein provision were multi-factorial. Dietitians are expertly skilled within the MDT to improve nutritional care within the ICU.

**References**

1. McClave SA, Martindale RG, Vanek VW, McCarthy M, Roberts P, Taylor B, et al. Guidelines for the Provision and Assessment of Nutrition Support Therapy in the Adult Critically Ill Patient: Society of Critical Care Medicine (SCCM) and American Society for Parenteral and Enteral Nutrition (A.S.P.E.N.) *JPEN J Parenter Enteral Nutr*. 2009; 33(3):277–316.

2. Weijs PJ. Fundamental determinants of protein requirements in the ICU. Current Opinion in Clinical Nutrition and Metabolic Care. 2014; 17:183–189.

**Fig. 74 (Abstract P289).**
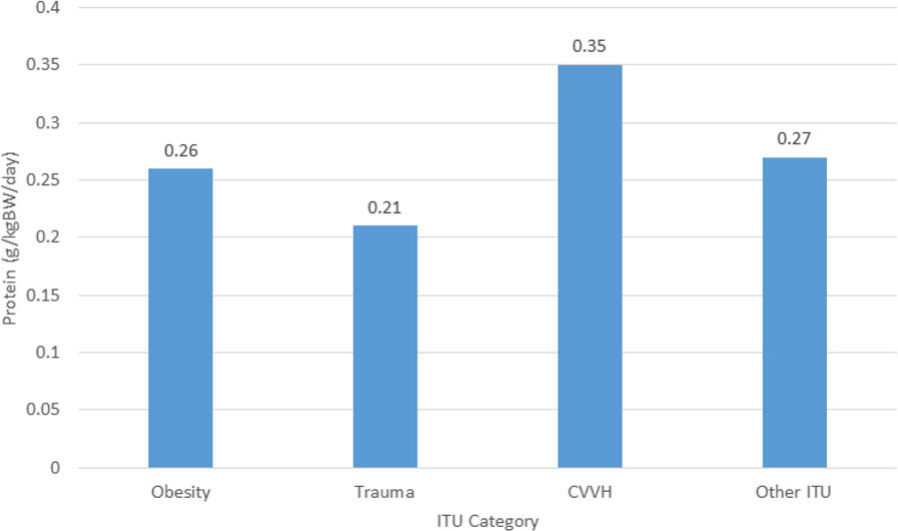
Mean protein deficit in g/kgBW/d compared to 1.2g

## P290 Prevalence and clinical outcomes of vitamin d deficiency in the medical critically ill patients in Songklanagarind hospital

### K. Maipang, R. Bhurayanontachai

#### Prince of Songkla University, Songkla, Thailand

**Introduction**

Vitamin D deficiency is commonly found in hospitalized patients, and associated to the multiple adverse clinical events, including increased in morbidity and mortality. Although, this condition is also frequently found in the critically ill patients, the correlation to clinical outcomes remains unclear. This report will demonstrate the prevalence of vitamin D deficiency in medical and the associated clinical outcomes among the medical critically ill patients in Thailand.

**Methods**

Prospective observational study was conducted during 6 months in the medical ICU in Songklanakarind hospital. Demographic data and clinical outcomes, including 28-days mortality, ventilator days, ICU and hospital length of stay, were collected. Serum 25-hydroxy-vitamin D (25(OH)D was measured within 24 hours after ICU admission. The 25(OH)D level less than 20 ng/ml is generally defined as a deficiency state. The correlation of 25(OH)D status on admission and clinical outcomes were then analyzed with chi-square test or student T-test. The p-value less than 0.05 defined as clinically significant.

**Results**

Of the 116 critically ill patients, the prevalence of 25(OH)D deficiency was 64.65 %. Patients with 25(OH)D deficiency were statistically significantly younger, had female gender, had higher blood glucose levels, higher rate of respiratory arrest 24 h prior to ICU admission and higher requirement of mechanical ventilator support. The 25(OH)D deficiency was not significantly correlated to the 28-day all-cause mortality. However, there was a trend toward increased in the vasopressor used, higher rate of ICU infection and longer hospital length of stay, among the deficiency group.

**Conclusions**

Our study demonstrated that the prevalence of vitamin D deficiency in Thai critically ill population was high (64.65 %). We also found the significantly higher rate of respiratory arrest and mechanical ventilator dependent in the deficiency group. However, the vitamin D deficiency in Thai critically illness was not significantly associated to the ICU morbidity and mortality.

**Table 39 (Abstract P290).** Clinical characteristics between 25(OH)-Vitamin D status

Variable	Sufficient ( > = 20 ng/ml) n = 41	Deficient ( <20 ng/ml) n = 75	p- value
Age (years), mean ± SD	61.44 ± 15.79	58.76 ± 21.10	0.018
Male gender (n,%)	31 (75.6%)	35 (46.6%)	0.003
Body mass index, mean ± SD	21.95 ± 4.29	22.04 ± 3.61	0.535
APACHE II, mean ± SD	16.73 ± 7.45	18.52 ± 8.68	0.124
Respiratory arrest 24 h prior to ICU (n,%)	19 (46.3%)	49 (65.3%)	0.047
Ventilator dependent (n,%)	20 (48.8%)	51 (68.0%)	0.042
Serum 25(OH)D (ng/ml), mean ± SD	34.97 ± 11.63	10.90 ± 4.90	<0.001

**Table 40 (Abstract P290).** Clinical outcomes between 25(OH)-Vitamin D status

Sufficient ( > = 20 ng/ml) n = 41	Deficient ( <20 ng/ml) n = 75	p-value	MICU mortality (%)
19.5%	21.3%	0.817	Total MICU length of stay (day+/-SD)
7.07 ± 7.62	6.97 ± 6.94	0.861	Hospital mortality (%)
21.9%	29.3%	0.368	28-day mortality (%)
17.0%	20.0%	0.701	Total Hospital length of stay (day+/-SD)
19.85 ± 20.43	26.78 ± 26.90	0.087	Vasopressor therapy (%)
63.4%	80.0%	0.051	Duration of ventilator (day+/-SD)
14.19 ± 15.27	11.37 ± 13.57	0.181	Rate of ICU infection (%)
19.5%	25.3%	0.478	

## P291 Vitamin d deficiency strongly predicts adverse medical outcome across different medical inpatient populations: results from a prospective study

### L. G. Grädel, P. Schütz

#### Kantonsspital Aarau, Aarau, Switzerland

**Introduction**

Vitamin D deficiency has been associated with several adverse outcomes mainly in the outpatient setting. The objective of this study was to examine the prevalence of vitamin D deficiency and its association with risk of adverse clinical outcomes in a large prospective cohort of medical inpatients.

**Methods**

We collected clinical data on measured 25(OH)D levels in adult medical patients upon hospital admission and followed them for 30 days. Regression analyses adjusted for age, gender, comorbidities and main medical diagnosis were performed to study the effect of vitamin D deficiency on several hospital outcomes.

**Results**

Of 4257 included patients, 1,510 (35.47 %) had 25(OH)D levels of 25-50 nmol/l (Vitamin D insufficiency) and 797 (18.72 %) had levels <25 nmol/l (severe deficiency). Vitamin D insufficiency and severe deficiency were associated (OR/HR, 95%CI) with an increased risk of 30-day mortality (OR 1.70, 1.22 to 2.36 and 2.70, 1.22 to 2.36) and increased length of stay (HR 0.88, 0.81 to 0.97 and 0.72, 0.65 to 0.81). Severe deficiency was associated with risk of falls (OR 1.77, 1.18 to 2.63), impaired Barthel index (OR 1.80, 1.42 to 2.28) and impairment in of quality of life. Most associations remained robust after multivariate adjustment and in subgroups based on gender, age, comorbidities and main diagnoses (P for interaction >0.05).

**Conclusions**

In this comprehensive and large medical inpatient cohort, Vitamin D deficiency was highly prevalent and strongly associated with adverse clinical outcome. Interventional research is needed to proof the effect of vitamin D supplementation on these outcomes.

## P292 Omega-3 fatty acids in patients undergoing cardiac surgery: a systematic review and meta-analysis

### P. Langlois ^1^, W. Manzanares^2^

#### ^1^Université de Sherbrooke, Sherbrooke, Canada; ^2^University Hospital, Montevideo, Uruguay

**Introduction**

Over the last few years, supplementation with omega-3 polyunsaturated fatty acids (n-3 PUFA) has emerged as a therapeutic option for patients undergoing cardiac surgery due to their immunomodulatory properties and anti-arrhythmic action. Nonetheless, there is a paucity of data supporting the effectiveness of n-3 PUFA in the treatment of cardiac surgery patients. So far, several randomized controlled trials (RCTs) have assessed the effect of perioperative n-3 PUFA preventing postoperative atrial fibrillation (POAF), although their efficacy still remains controversial. Therefore, we conducted an updated systematic review and meta-analysis evaluating the effects of perioperative oral/enteral n-3 PUFA and intravenous (IV) fish oil lipid emulsions on relevant clinical outcomes for cardiac surgery patients.

**Methods**

We included RCTs enrolling adult patients undergoing cardiac surgery, which evaluated oral/enteral and parenteral n-3 PUFA compared to a placebo and reported clinically important outcomes. According to eligibility criteria, original studies were abstracted in duplicate. Intensive care unit (ICU) length of stay (LOS) was the primary outcome; secondary outcomes were incidence of POAF, duration of mechanical ventilation (MV) and hospital LOS. Hypothesis-generating subgroup analysis was performed to identify potentially more beneficial treatment strategies.

**Results**

**Fig. 75 (Abstract P292).**
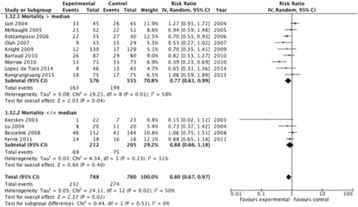
Atrial Fibrillation: subgroup analysis

A total of 11 RCTs (n = 2846 patients) met inclusion criteria. When the data from 4 trials were aggregated, n-3 PUFA had no effect on ICU LOS (WMD -4.43, 95 % CI -13.43, 4.48, P = 0.34, heterogeneity I2 = 43 %). However, n-3 PUFA were associated with a trend in the reduction of POAF (RR 0.89, 95 % CI 0.76, 1.05, P = 0.17; I2 = 34 %, P = 0.13). In addition, in those oral/enteral based trials, n-3 PUFA showed a tendency towards a reduction in POAF (RR 0.87, 95 % CI 0.74, 1.03, P = 0.11; I2 = 47 %, P = 0.07; Fig. 75). The test for subgroup differences on overall POAF showed a trend (P = 0.16). There was no effect of n-3 PUFA on MV days and hospital LOS.

**Conclusions**

In patients undergoing cardiac surgery, supplementation with omega-3 fatty acids does not improve any clinical outcome in the postoperative period.

## P293 Can 5-hydroxytriptophan prevent post-traumatic stress disorder in critically ill patients?

### R. Tincu^1^, C. Cobilinschi^1^, D. Tomescu^2^, Z. Ghiorghiu^1^, R. Macovei^1^

#### ^1^Bucharest Clinical Emergency Hospital, Bucharest, Romania; ^2^Fundeni Clinical Institute, Bucharest, Romania

**Introduction**

Long-term hospitalized patients in Intensive Care Units (ICUs) are at high risk of developing major depressive syndrome or Post-traumatic Stress Disorder (PSD). There are correlations between the degree of depression and survival in these patients. The incidence of depression and PSD is three times higher in patients in intensive care compared with the general population. 5-hydroxytryptophan, a precursor of serotonin, can be used for the prevention of depression in these patients.

**Methods**

We conducted a randomized controlled trial on 30 patients, hospitalized in our ICU for non-surgery causes. We dosed serotonin levels on admission day, at 7 and at 14 days. Patients with psychiatric disorders and malignancies were excluded as those who were in treatment with drugs that interfere with the metabolism of serotonin. Selected patients were randomly assigned either to receive placebo treatment (first group, N = 30) or to received 300 mg of 5-hydroxytryptophan (5-HTP) (second group, N = 30). Psychiatric evaluation was performed using the Clinician-Administered Posttraumatic Stress Disorder Scale.

**Results**

At baseline, no significant differences were recorded regarding serotonin plasma levels (243 ± 47.75 and 252.6 ± 38.59 μ g/l, P > 0.05) or age (55 ± 12.38 and 54.66 ± 12.11, P > 0.05), in both groups. After the first determination we observed a decrease in serotonin levels in the placebo group (184.46 ± 37.57) and in 5-HTP group (229.26 ± 39.35) (p < 0.001, T-value is 3.18). Delirium was present in 3 patients in the placebo group and 1 patient in 5-HTP group. After 14 days, serotonin levels were lower in the placebo group when compared to treatment group (P < 0.001), and delirium was absent in the latter. Mean end points for Clinician-Administered Posttraumatic Stress Disorder Scale were higher in placebo group when compared to treatment group (P = 0.006), at the same time.

**Conclusions**

Several studies suggest that depression can increase mortality in critically ill patients. The use of antidepressants in these patients may be contraindicated, therefore 5-HTP can become an alternative therapy in depression of patients in ICU.

## P294 Parenteral selenium in the critically ill: an updated systematic review and meta-analysis

### W. Manzanares^1^, P. Langlois^2^, M. Lemieux^3^, G. Elke^4^, F. Bloos^5^, K. Reinhart^5^, D. Heyland^3^

#### ^1^University Hospital, Montevideo, Uruguay; ^2^Université de Sherbrooke, Sherbrooke, Canada; ^3^Queen’s University, Kingston, Canada; ^4^University Medical Center Schleswig-Holstein, Kiel, Germany; ^5^University of Jena, Jena, Germany

**Introduction**

Selenium is an essential trace element with antioxidant and immunomodulatory effects. Several randomized controlled trials (RCT) and meta-analyses have demonstrated that parenteral selenium may be able to improve clinical outcomes in intensive care unit (ICU) patients and new trials have been published since our last update. Thus, we updated our data with this systematic review on parenteral selenium as single strategy or in combination with other antioxidant micronutrients in the critically ill.

**Methods**

We included RCTs that evaluated clinical outcomes associated with intravenous selenium as single or combined strategy in parenterally or enterally fed patients. Overall mortality was the primary outcome; secondary outcomes were infections, ICU length of stay (LOS), hospital LOS, and ventilator days. Subgroup analyses were done to elucidate the effects of selenium on mortality and infections (Table 41).

**Results**

21 trials met our inclusion criteria (n = 4129). When the results of the 20 trials reporting mortality were aggregated, no statistically significant reduction in mortality was found (RR 0.99, 95 %, CI 0.99, 1.08, P = 0.75, I2 = 0 %). In addition, there was no significant effect of parenteral selenium on infections (P = 0.15). There was no effect of IV selenium therapy on ICU LOS, hospital LOS, and ventilator days. Result of subgroup analysis is found in Table 41.

**Conclusions**

Parenteral selenium as monotherapy or in combination with other antioxidant micronutrients has no effect on overall mortality, infections, as well as on ICU LOS, hospital LOS, and ventilator days in critically ill patients.

**Table 41 (Abstract P294).** Subgroup analysis

Subgroup analysis	Mortality	Infections
Monotherapy	RR 0.91 (95% CI 0.80, 1.04; P = 0.18)	RR 0.95 (95% CI 0.85, 1.06; P = 0.36)
Combined therapy	RR 1.08 (95% CI 0.93, 1.25; P = 0.33)	RR 0.90 (95% CI 0.78, 1.05; P = 0.18)
High dose	RR 0.98 (95% CI 0.87, 1.11; P = 0.78)	RR 0.97 (95% CI 0.89, 1.05; P = 0.46)
Daily dose = 500 mcg	RR 0.88 (95% CI 0.57, 1.34; P = 0.54)	RR 0.87 (95% CI 0.64, 1.19; P = 0.39)
Low dose	RR 0.94 (95% CI 0.67, 1.33; P = 0.75)	RR 0.87 (95% CI 0.72, 1.05; P = 0.15)
Loading dose	RR 0.91 (95% CI 0.76, 1.09; P = 0.30)	RR 0.99 (95% CI 0.90, 1.09; P = 0.84)
No loading dose	RR 1.01 (95% CI 0.90, 1.14; P = 0.83)	RR 0.87 (95% CI 0.77, 0.99; P = 0.03)

## P295 Probiotics in the critically ill: an updated systematic review and meta-analysis

### P. Langlois ^1^, M. Lemieux^2^, I. Aramendi^3^, D. Heyland^2^, W. Manzanares^3^

#### ^1^Université de Sherbrooke, Sherbrooke, Canada; ^2^Queen’s University, Kingston, Canada; ^3^University Hospital, Montevideo, Uruguay

**Introduction**

Critical illness is characterized by changes in the microbiology of the intestinal tract, leading to a loss of commensal flora and an overgrowth of pathogenic bacteria. Probiotics are living non-pathogenic bacteria colonizing intestine and providing benefits to the host. In 2012, we demonstrated that probiotics may be able to reduce infections and may influence intensive care unit (ICU) mortality1. Over the last three years different randomized controlled trials (RCTs) have been published. Therefore, the aim of this systematic review is to update our previous data on probiotics in the critically ill.

**Methods**

We included RCTs enrolling critically ill adults, which evaluated probiotics compared to a placebo and reported clinically important outcomes. According to eligibility criteria, original studies were abstracted in duplicate by two reviewers. Overall infections was the primary outcome; secondary outcomes were overall mortality, ventilator-associated pneumonia (VAP), diarrhea, ICU length of stay (LOS), and hospital LOS.

**Results**

**Fig. 76 (Abstract P295).**
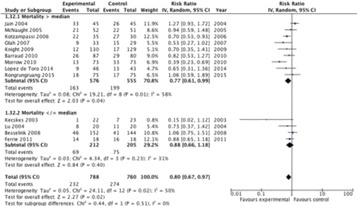
Effects of probiotics on infections

A total of 29 RCTs (n = 2737) met inclusion criteria, including 6 new trials since our last update. When the results of trials reporting infections were aggregated, probiotics were associated with a significant reduction in infections (RR 0.82, 95 % CI 0.69, 0.97, P = 0.02; heterogeneity I2 = 41 %), and particularly among patients with higher risk of death (RR 0.75, 95 % CI 0.61, 0.99, P = 0.04; I2 = 58 %; Fig. 76). Moreover, probiotics were associated with a significant reduction in the incidence of VAP (RR 0.74, 95 % CI 0.58, 0.96, P = 0.02, I2 = 29 %). Finally, probiotics had no effect on mortality, diarrhea, and hospital LOS

**Conclusions**

Probiotics are associated with a significant reduction in overall infections, as well as a reduction in the incidence of VAP.

**Reference**

1. Petrof EO, et al. Probiotics in the critically ill: a systematic review of the trial evidence. Crit Care Med. 2012; 40: 3290-3292.

## P296 Diabetes with hyperglycemic crisis episodes may be associated with higher risk of pancreatic cancer: a population-based cohort study

### Y. Su

#### Dalin Tzu Chi Hospital, Buddhist Tzu Chi Medical Foundation, Chiayi County, Taiwan

**Introduction**

The relationship between diabetes and pancreatic cancer has been discussed. However, the effects of diabetes with poor control on pancreatic cancer have never been evaluated. We address the strength of association for relationship between diabetes and pancreatic cancer.

**Methods**

The data from 1,000,000 National Health Insurance beneficiaries were utilized. The study cohort consisted of 42,581 diabetic patients and 672,750 unexposed subjects. Among patients with diabetes, 1082 have been admitted with hyperglycemic crisis episodes. All adult beneficiaries were followed from 1 January 2005 to 31 December 2012 to evaluate if pancreatic cancer was diagnosed. Cox regression models were applied to compare the hazards adjusted for potential confounders.

**Results**

After controlling for age, gender, urbanization level, socioeconomic status, liver cirrhosis, hypertension, coronary artery disease, hyperlipidemia, malignancy, smoking, chronic obstructive pulmonary disease, obesity, history of alcohol intoxication, chronic renal insufficiency, biliary tract disease, chronic pancreatitis and Charlson Comorbidity Index score, the adjusted hazard ratio of pancreatic cancer was 2.48 (95 % confidence interval, 1.84¡X3.34) in patients with diabetes. In patients with history of hyperglycemic crisis episodes, the hazard ratio of pancreatic cancer was significant higher. (hazard ratio, 3.60; 95 % confidence interval, 1.15¡X11.25)

**Conclusions**

The cohort study provided evidence for the relationship between diabetes and pancreatic cancer. Moreover, diabetes with hyperglycemic crisis episodes is associated with higher risk of pancreatic cancer.

## P297 Incidence of hypoglycemia in an intensive care unit depending on insulin protocol

### R. Marinho, N. Babo, A. Marinho

#### Centro Hospitalar do Porto, Porto, Portugal

**Introduction**

Hypoglycemia is associated with increased mortality in critically ill patients. The aim of this study was to assess glucose control in an intensive care unit and the incidence of hypoglycemia depending on insulin protocol.

**Methods**

Single-center prospective observational study. All patients admitted to 2 ICUs between January and April of 2014 for more than five days, were enrolled. Data on demographics and clinical characteristics of patients were collected at baseline. All blood glucose measurements during the first 5 days were recorded. Three insulin protocols were used, two using subcutaneous actrapid insulin depending if the patient is diabetic (DP) or not (NDP), and an intensive protocol (IP) using IV insulin depending solely on the glycemic value using a target value of 180 mg/dL. A glucose value of 70 mg/dL or less was considered moderate hypoglycemia and 40 mg/dL or less as severe hypoglycemia. SPSS was used for statistical analysis.

**Results**

106 patients were included, 66 (66,26 %) were men, with an average age of 62,53 ± 15,61 years, mortality rate was 20,75 %. 50 (47,17 %) patients were non-surgical. 34 were diabetic (32,08 %). On average 6.1 glucose measurements were performed per patient/day. The average value of blood glucose levels over the first five days was 168,18 +/- 46,93 mg/dL (diabetic - 182,65 +/- 46,91 mg/dL; nondiabetic - 161,18 +/- 46,93 mg/dL). Depending on protocol the average value of blood glucose was 190,76 +/- 52,20 mg/deal for DP, 158,12 +/- 39,65 mg/dl for the NDP and 175,12 +/- 47,03 mg/dl (IP). The glycemic variability was 103,45 +/- 71,06 mg/dl for DP, 64,41 +/- 55,14 mg/dl for NDP and 113,50 +/- 67,76 mg/dl for IP. 17 patients (16,04 %) had at least 1 episode of moderate hypoglycemia and 7 patients (6,6 %) had severe hypoglycemia. In diabetic patients 19/886 (2,14 %) determinations were under 70 mg/dL and 4/886 (0,45 %) were under 40 mg/dL, as for non-diabetic 15/1720 (0,87 %) were under 70 mg/dL and 5/1720 (0,29 %) were under 40 mg/dL. Depending on insulin protocol, the incidence of moderate hypoglycemia was 2,88 % in DP, 0,98 % in NDP and 0,96 % in IP; and in severe hypoglycemia was 0,89 % in DP, 0,25 % in NDP and 0,21 % in IP.

**Conclusions**

As usually the incidence of hypoglycemia was higher in the diabetic population. With the new target for glycemic control in intensive therapy, the incidence of hypoglycemia is now very low in this group. In contrast the protocol for NDP had a better glycemic control than the Intensive Protocol.

## P298 Severity of the diseases is two-dimensionally correlated to blood glucose, including blood glucose variability, especially in moderately to severely ill patients with glucose intolerance.

### M. Hoshino^1^, Y. Haraguchi^2^, S. Kajiwara^1^, T. Mitsuhashi^3^, T. Tsubata^2^, M. Aida^1^

#### ^1^Aida Hospital, Fukushima, Japan; ^2^Keiyo Hospital, Tokyo, Japan; ^3^Shisei Hospital, Saitama, Japan

**Introduction**

Elucidation of the relationship between severity of the diseases and blood glucose(BG), including BG variability, is considered to be significant for determining BG targets. The purpose of this study is to analyze the relationship because that analysis has not been fully performed.

**Methods**

One hundred and forty nine patients with glucose intolerance were analyzed during 1 week after ICU admission. Studied items were, 1) maximum value of SOFA score(SOFAmax), 2)mean of BG(BGm) and 3)standard deviation of BG(BGsd). BG was measured basically every 6 hours and insulin was used when BG was above 140 mg/dL. Correlation between SOFAmax and BG(BGm, BGsd) was analyzed according to the SOFAmax levels.

**Results**

1)Mortality of the patients with SOFAmax of 0-3(n = 60), 4(n = 26), 5(n = 21), 6(n = 13), 7-8(n = 12), 9-10(n = 8), 11-19(n = 9) was 0 %, 8 %, 14 %, 23 %, 25 %, 50 % and 78 %, respectively. 2)BGsd was linearly correlated to BGm(correlation coefficient r = 0.79). 3)SOFAmax was two-dimensionally correlated to both BGm and BGsd. Range of BGm and BGsd with SOFAmax less than 4, calculated from the two-dimensional, or quadratic, equation, was 121-178 and 24-44 mg/dL, respectively. 4)Two-dimensional correlation coefficient(R) between SOFAmax and BGm according to SOFAmax above 0(n = 149), 2(n = 121), 4(n = 89), 6(n = 42), 8(n = 21) and 10(n = 11) was 0.38, 0.41, 0.37, 0.49, 0.66 and 0.54, respectively. 5)R between SOFAmax and BGsd according to SOFAmax above 0, 2, 4, 6, 8 and 10 was 0.43, 0.46, 0.45, 0.54, 0.62 and 0.70, respectively.

**Conclusions**

1)BG control to hyperglycemia is one of the measures which bring the reduction of BG variability. 2)BG targets were considered to be obtained by the analysis of two-dimensional relationship between severity of the diseases and BG. 3)There is a possibility that strict BG control is more effective to severer(moderate to severe) patients, considering the results indicating higher correlation coefficient (R) in severer patients. 4)Glucose administration for increasing BG and BG variability might bring beneficial effect not only for hypoglycemic patients but also for a part of normoglycemic patients, judging from BG targets listed above. 5)Low BG variability in a part of severe patients may be related to lower variability of physiologic activity. 6)Analysis of the relationship between severity of the diseases and BG based on the fine BG control, including by the use of artificial pancreas, was considered to link to better BG control and outcomes.

## P299 A study of glycemic control by subcutaneous glargine injection transition from continuous regular insulin infusion in critically ill patients

### T. Rattanapraphat, R. Bhurayanontachai, C. Kongkamol, B. Khwannimit

#### Prince of Songkla University, Hat Yai City, Songkhla province, Thailand

**Introduction**

Vary Insulin protocols were applied to control blood glucose(BG) in critical patients, mostly based on short acting insulin, which consumed lots of manpower and resources. By previous study yielded 66.7 % success rate of glycemic control with 80 % dose conversion from short acting insulin dose with mean of differences of 26.5 mg/dL. The aim of this study was to determine whether a single subcutaneous glargine injection with 100 % dose transition is not inferior to the continuous regular insulin (RI) infusion for BG control in critically ill patients (inferior margin acquired from 85 % of the mean of differences from previous study =22.525 mg/dL).

**Methods**

All eligible participants admitted Critical care unit which required constant rate of continuous RI infusion per standard MICU insulin infusion protocol for 24 consecutive hours were switched to receive single dose of glargine insulin subcutaneously by 100 % of their total daily insulin requirement calculated by cumulative dose of RI requirement in the previous 24 hours. The continuous insulin infusion was discontinued 2 hours after the transition. BG were monitored every 2 hours for the following 24 hours or until the study termination or discontinuation

**Results**

Of 20 cases included, 12 cases achieved a good glycemic control. Among success subgroup, there were no BG level higher than mean glucose during RI more than 22.525 mg/dl. Only a mild hypoglycemia event was observed with spontaneously resolved. Otherwise among 8 participants in failure subgroup, half of them developed hyperglycemia at 8th hour. This research also found that median RI dose was different as 1.8 mg/dL among success cases and 2.5 mg/dL among failure cases.

**Conclusions**

A single subcutaneous Glargine injection with 100 % dose transition was not inferior to the continuous RI infusion for BG control in critically ill patients with 60 % success rate. Higher pre-treatment RI dose was related to failure and success outcome.

**Fig. 77 (Abstract P299).**
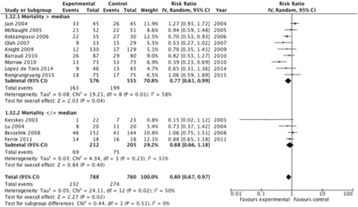
ᅟ

## P300 Glycemic control in Portuguese intensive care unit

### R. Marinho^1^, M. Santos^2^, H. Castro^1^, E. Lafuente^3^, A. Salgueiro^4^, S. Cabral^5^, P. Martins^4^, J. Moura^6^, B. Oliveira^2^, M. Melo^7^, B. Xavier^7^, J. Valente^8^, C. Magalhaes^9^, P. Casteloes^10^, A. Marinho^1^

#### ^1^Centro Hospitalar do Porto, Porto, Portugal; ^2^Faculdade de Ciencias da Nutrição e Alimentação da Universidade do Porto, Porto, Portugal; ^3^Centro Hospitalar Tamega e Sousa, Penafiel, Portugal; ^4^Centro Hospitalar e Universitário de Coimbra, Coimbra, Portugal; ^5^Instituto Português de Oncologia do Porto, Porto, Portugal; ^6^Unidade Local de Saude do Alto Minho, Viana do Castelo, Portugal; ^7^Centro Hospitalar Baixo Vouga, Aveiro, Portugal; ^8^Unidade de Saude Local de Castelo Branco, Castelo Branco, Portugal; ^9^Centro Hospitalar do Algarve, Faro, Portugal; ^10^Centro Hospitalar de Vila Nova de Gaia, Vila Nova de Gaia, Portugal

**Introduction**

Target levels of glycemia in critically ill patients have fluctuated since 2001, as evidence initially indicated that tight glycemic control (80-110 mg/dl) leaded to the lowest morbidity and mortality, however subsequent studies have demonstrated minimal clinical benefit combined with greater morbidity and mortality, so nowadays the target glucose level approaches 150-180 mg/DL. The aim of this study was to assess glucose control in critically ill patients in Portuguese ICUs and evaluate the incidence of hyperglycemia and hypoglycemia.

**Methods**

Prospective observational study conducted in eleven ICUs in nine Portuguese hospitals for six months. Included participants were patients who were expected to have a length of stay (LOS) in the ICU of 7 days or more. Data on demographics and clinical characteristics of patients were collected at baseline. All patients were assigned a target glucose of 180 mg/dL or less. All blood glucose measurements during the first 7 days were recorded. A glucose value of <40 mg/dL was considered as severe hypoglycemia.

**Results**

155 patients were included, where 60.6 % were men, with an average age of 62. ± 17, ICU LOS was 15.2 ± 6.4 days and mortality rate was 25.2 %. 70 % of patients were non-surgical. 49 were diabetic. 1085 days of hospitalization were evaluated and 6563 glucose measurements were performed, with an average of 6,0 +/- 2,5 per patient/day. The average value of blood glucose levels over the first seven days was 155,8 +/- 40,6 mg/DL (diabetic (D) - 175,2 +/- 82,8 mg/DL; nondiabetic (ND) -147,1 +/- 82,8 mg/dL). 71/6563 determinations were below 70 mg/dL and 11/6563 were below 70 mg/DL and 11/6563 were below 40 mg/DL (per patient D - 2,0 %; ND - 0.9 %). 2302/6563 (26 %) were above 180 mg/dL (D - 63.5 %; ND - 22.9 %).

**Conclusions**

Using a target glucose level of 180 mg/DL, uncontrolled hyperglycemia was avoided over 72 %, with fewer hypoglycemic episodes. As diabetics had more hyperglycemic and hypoglycemic events, may be diabetic patients should have different targets.

## P301 Impact of hyperglycemia duration on the day of operation on short-term outcome of cardiac surgery patients

### D. Moisidou, F. Ampatzidou, C. Koutsogiannidis, M. Moschopoulou, G. Drossos

#### Department of Cardiothoracic Surgery, George Papanikolaou General Hospital , Thessaloniki, Greece

**Introduction**

Aim of this study was to investigate whether hyperglycemia duration (hours/24 hour cycle) intraoperative and on first ICU day, has any impact on postoperative outcome after cardiac surgery

**Methods**

This long-range observational study, carried in our department, from January 2013 until June 2014 and consisted of data from 615 cardiac surgery patients. Based on glucose levels on the operation day (24 hours), patients were divided in 5 groups depending on the hours of hyperglycemia. We investigated the possible relationship between duration of hyperglycemia and the following postoperative complications: Deep sternal wound infection, acute kidney injury, need of intravenous drip infusion of a diuretic solution and renal replacement therapy requirement in ICU. The study was conducted by an independent investigator and the results were processed by statistician with the statistical program SPSS 20.

**Results**

Results are showed in Table 42.

**Conclusions**

Hyperglycemia has statistical significant relationship with AKI, need of IV diuretics and RRT requirement. In our study, hyperglycemia has no impact on postoperative sternal wound infections.

**Table 42 (Abstract P301).** ᅟ

Hyp/mia hours	Pts	AKI	DIURETIC	RRT	DSWI
0	55	12.7%	16.4%	0	1
1-6	116	12.6 %	8.6%	0	5
7-12	162	13.6%	16%	2	8
13-18	197	18.3%	24.9%	4	8
19-24	85	23.5%	40%	7	4
	616	(p = 0.03)	(p = 0.00)	(p = 0.01)	(p = 1)

## P302 Lactate levels in diabetic ketoacidosis patients at ICU admissions

### G. Taskin^1^, M. Çakir^1^, AK Güler^1^, A. Taskin^2^, N. Öcal^1^, S. Özer^1^, L. Yamanel^1^

#### ^1^Gülhane Military Medical Academy, Ankara, Turkey; ^2^Ankara Mevki Military Hospital, Ankara, Turkey

**Introduction**

Diabetic ketoacidosis (DKA) is a life-threatening acute complication of diabetes that presents with high-anion-gap metabolic acidosis, hyperglycemia and ketonemia (1). The aim of this study is to determine lactate levels and its clinical outcomes in DKA patients admitted to the medical Intensive Care Unit (ICU).

**Methods**

A total of thirty-one (31) patients who admitted to our medical ICU of tertiary care university hospital with DKA in last thirty (30) months was included in the study retrospectively. Hyperlactatemia was defined as serum lactate >2.5 mmol/L.

**Results**

The mean age of patients with DKA was 50.4 ± 24.1 and 45.2 % (n = 14) of them were men and 54.8 % (n = 17) were women. Twelve (38.7 %) of patients had type 1 diabetes and 19 (61.3 %) had type 2. The mean serum lactate was 2.93 ± 1.81 mmol/L (0.89–9.20). Of 31 patients, 14 (45.2 %) had elevated serum lactate levels (>2.5 mmol/L), and 6 (19.4 %) had high lactate levels (>4 mmol/L). No correlation was found between serum lactate levels and mortality, length of ICU and hospital stay. Besides, lactate levels were not correlated with Acute Physiology and Chronic Health Evaluation II (APACHE II) score, blood pressure, serum glucose and HbA1C levels.

**Conclusions**

Elevated lactate level is commonly found in DKA patients (2). However, this is not associated with worse clinical outcomes and disease severity (3). Thus, elevated lactate levels determined in DKA patients should not to be appreciated by clinicians to define a high-risk population.

**References**

1. Kitabchi AE, Umpierrez GE, Murphy MB, et al. Hyperglycemic crises in adult patients with diabetes: a consensus statement from the American Diabetes Association. Diabetes Care. 2006; 29(12):2739–2748.

2. Fulop M, Hoberman H, Rascoff J, et al. Lactic acidosis in diabetic patients. Arch Intern Med. 1976; 136(9):987-990.

3. Cox K, Cocchi MN, Salciccioli JD, et al. Prevalence and significance of lactic acidosis in diabetic ketoacidosis. J Crit Care. 2012 April ; 27(2): 132–137.

## P303 Intensive care implications of merging heart attack centre units in London

### J. M. Wong, C. Fitton, S. Anwar, S. Stacey

#### St Bartholomew’s Hospital, London, UK

**Introduction**

The London Chest Hospital and The Heart Hospital merged with St Bartholomew’s Hospital to become the Bart’s Heart Centre, receiving all Heart Attack Centre (HAC) Activations in the North Central and North East London areas. This created the largest Cardiac Intensive Care Unit in Europe with 42 beds currently (and expanding to 58 next year.) We are reporting the changes that have occurred following the merger of two HAC services in adjacent regions. Data was collected from the Intensive Care National Audit and Research Centre (ICNARC) database as well as the Myocardial Ischaemia National Audit Programme (MINAP).

**Methods**

The ICNARC and MINAP databases were interrogated to identify numbers for:

- HAC activation
- pPCI (primary percutaneous coronary intervention)
- Admissions to Level 3 Intensive Care.

We compared data from the five months following the merger (May – September 2015) with the same months in 2014. High dependency and coronary care admissions were excluded. The in-unit mortality and length of stay were examined. Results are expressed as median and interquartile range (IQR) for non-normal distribution data. Ethics approval was sought but deemed not necessary.

**Results**

Between May and September 2014 there were 628 HAC activation patients admitted to the legacy sites of the London Chest Hospital and Heart Hospital. Following the merger there was an increase of 246 patients (39 %). Of the HAC activations at the legacy sites from May-September 2014, 282 (45 %) patients underwent primary percutaneous coronary intervention, pPCI, compared to 254 (29 %) patients in the five months post-merger. ICNARC data was not collected at the Heart Hospital and therefore data is only available from one legacy site. The ITU admission rate at the London Chest was observed as 4.9 % compared to 6.4 % in the new Centre. The length of stay on Intensive Care increased following the merger from a median 3.9 days (IQR 2.1-8.0) to a median 2.9 days (IQR 1.6-7.0). The in-unit mortality decreased in May-September 2015 to 41.1 % from 45.2 % the previous year.

**Conclusions**

To our knowledge this is a unique situation where three specialised London hospitals have merged to produce one large cardiac centre. The rise in patient numbers yet fall in pPCI rate is interesting and further analysis is needed to understand this trend.

## P304 Special characteristics of in-hospital cardiac arrests

### M. Aggou, B. Fyntanidou, S. Patsatzakis, E. Oloktsidou, K. Lolakos, E. Papapostolou, V. Grosomanidis

#### Aristotle Medical School, Thessaloniki, Greece

**Introduction**

In-hospital cardiac arrests (CAs) are not a sudden event but in most of the cases the result of a slow and progressive deterioration of the patient. The aim of this study was the systematic analysis of CA incidence, primary causes and initial rhythm with regard to its location in the hospital in order to identify potential obstacles and to undertake preventive measures to enhance outcome.

**Methods**

177 in-hospital CAs, in which resuscitation was attempted, were included in the study and data were recorded according to the Utstein Style template for in-hospital CA. Age and gender of patients, primary causes, initial rhythm and location of CA were recorded and reviewed.

**Results**

Results are depicted on Tables 1 & 2.

**Conclusions**

The vast majority of CAs (42.3 %) occurred in areas supervised by the cardiology department. Together with the “cardio surgical” CAs, cardiology related CAs percentage arises to 58.2 %. Number of CAs in the ED and in the RIU was also significant. Mean age of CAs patients was similar in all locations, whereas there were significant differences as far as primary causes and initial rhythm analysis are concerned. These interesting results merit further study, larger number of patients and detailed analysis of all related parameters in order to conduct safe conclusions and to be able to undertake preventive measures.

**Fig. 78 (Abstract P304).**
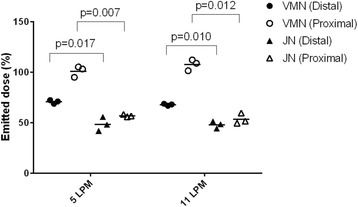
Demographic data, causes and initial rhythm of CAs

**Fig. 79 (Abstract P304).**
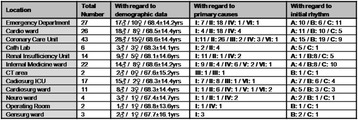
Location of CAs and correlation with all recorded parameters

## P305 Clinical evaluation of ICU-admitted patients who were resuscitated in the general medicine ward

### S. Suda , T. Ikeda, S. Ono, T. Ueno, Y. Izutani

#### Tokyo Medical University Hachiosi Medical Center, Tokyo , Japan

**Introduction**

Patients with nosocomial cardiopulmonary arrest (CPA) have aggravated clinical signs before CPA occurs. The early recognition of these signs and quick treatment may reduce the death rate in these patients. We examined the background factors and various biomarkers of the patients who were resuscitated in the general medicine ward.

**Methods**

Eighteen patients who were admitted to the ICU after resuscitation in the general medicine ward were studied. Patients were classified as gfair prognosis h (FP-group: 12 persons) if the patient survived more than 28 days or gpoor prognosis h (PP-group: 6 persons) if the patient died within 28 days. Blood samples were drawn for measuring blood chemistry and sepsis-related biomarkers (procalcitonin, presepsin, endotoxin activity assay) immediately after ICU admission. Statistical analysis was performed with the Mann-Whitney U-test and Chi square test or Fisher’s test. A value of p < 0.05 indicated statistical significance.

**Results**

The underlying diseases were as follows: sepsis (5), acute respiratory distress syndrome (3), lethal arrhythmia (2), ileus (1), suffocation (1), amyotrophic lateral sclerosis (1), sudden unexpected death in epilepsy (1), unknown (4). The SOFA scores of the FP-group and PP-group were 10}3 (10) and 14}3 (14), respectively; the difference between the groups was statistically significant (p < 0.05). APACHE2 scores of the FP-group and PP-group were 34}7 (35) and 40}8 (44), respectively; however, there was no significant difference between the groups. Procalcitonin levels of the FP-group and PP-group were 9}13 (6) and 22}26 (15), respectively; again there was no significant difference between the groups. IL-6 levels of the FP-group and PP-group were 1096}2851 (98.8) and 19,475}24,603 (11,305), respectively; the difference between the groups was statistically significant (p < 0.05). The EAA level of the FF-group was 0.41}0.16 (0.46), whereas the PP-group was 0.54}0.26 (0.49); however, there was no significant difference in EAA levels between the groups.

**Conclusions**

In our institute, the 28-day survival rate of the patients who were resuscitated in the general medicine ward was 66.7 %. The factors which affect patient outcome were SOFA score and IL-6. However, we could not evaluate the duration of CPA; therefore, it will be necessary to examine this further in the future.

## P306 Serious game evaluation of a one-hour training basic life support session for secondary school students: new tools for future bystanders

### S. Gaudry^1^, V. Desailly^1^, P. Pasquier^2^, PB Brun^3^, AT Tesnieres^2^, JD Ricard^1^, D. Dreyfuss^1^, A. Mignon^2^

#### ^1^Hôpital Louis Mourier, Colombes, France; ^2^Laboratoire ILUMENS, PARIS, France; ^3^Institut de Hauts de Seine, Nanterre, France

**Introduction**

Teaching basic life support (BLS) to secondary school students has been proved feasible, but not much evaluated. We conducted a study to evaluate the efficacy of a one-hour BLS training course for secondary school students.

**Methods**

From September 2014 to June 2015, six secondary schools were included in the study. Two of them received a one-hour BLS session conducted by an intensivist assisted by 3 medical students. Sessions contained: a theoretical lecture and a practical simulation-based training using low-fidelity manikins (MiniAnnePlus®, Laerdal). Between 2 and 3 months later, both trained and non-trained students were assessed using a serious game reproducing real-life cardiac arrest situation (3D real-time simulation software, Stayingalive®, ILUMENS-Dassault system). Primary outcome was the time spent to complete the serious game (from onset of cardiac arrest to shock delivery and recovery). Secondary outcomes included knowledge of emergency call, hand placement during chest compressions and electrodes placement of automated external defibrillator.

**Results**

Among 199 students assessed on the serious game, 72 had received the one-hour BLS session. Children’s ages did not differ between the 2 groups (12 [11-12] vs 12 [11-12] years old, p = 0.7).

**Fig. 80 (Abstract P306).**
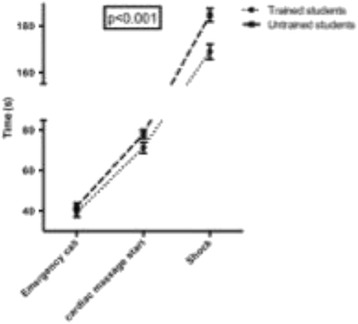
Game completion time

The total game completion time was 169 (+/-28) seconds in the trained group versus 185 (+/-32) seconds in the non-trained group (p = 0.0006) (Fig. 80).

Knowledge of emergency call did not differ between the two groups (92 % in trained group vs 86 % in non-trained group, p = 0.2). Hands were more often properly placed during chest compression in the trained-group (92 % vs 73 %, p = 0.002). Electrodes of automated external defibrillator were more often properly placed in the trained-group (44 % vs 10 %, p = 0.0001).

**Conclusions**

A one-hour BLS training session with low-fidelity manikin improved significantly young students’ performance during a 3D-real life simulation session.

## P307 Public and clinical staff perceptions and knowledge of CPR compared to local and national data

### J. C White^1^, A. Molokhia^1^, A. Dean^2^, A. Stilwell^1^, G. Friedlaender^3^

#### ^1^University Hospital Lewisham, London, UK; ^2^Queen Elizabeth Hospital , London, UK; ^3^Minet Green Health Practice, London, UK

**Introduction**

People have unrealistic expectations of the outcomes of inpatient adult cardiopulmonary resuscitation (CPR). We conducted a survey looking at clinical staff and the general public perceptions and knowledge of CPR compared to local and national data.

**Methods**

276 public and staff were surveyed at 2 hospitals and a GP surgery in South East London.

**Results**

75.2 % of the public knew what CPR was and 29.5 % felt they had enough CPR knowledge. Knowledge did not appear to be influenced by attending university.

**Fig. 81 (Abstract P307).**
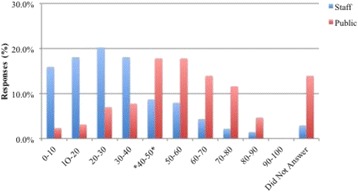
shows the predicted survival rates of initial CPR (staff v public)

Compared to the national figure of 45.6 % for initial CPR survival, the public predicted the correct survival range more accurately (see Fig. 81) [1]. Staff results were more comparable to their local hospital data. Hospital A’s initial survival rate was 33 %: 15 % of staff compared to 6.6 % of public chose the correct survival range. In hospital B, every doctor underestimated the initial CPR survival figure (55 %). Nurses were closer to the figure with the most popular response range being 30-40 % compared to 10-20 % for doctors. GPs were most pessimistic at predicting general CPR survival rates and nurses were most optimistic.

87 % of staff wanted CPR if they were healthy, this dropped to 29 % if they were to have a chronic illness that affected them daily. Public figures were 62 % and 48.8 % respectively. People of African origin were less likely to want CPR if they had a chronic illness, compared to Caucasians. Those who went to university were more likely to want to discuss CPR with a doctor. Less than 3 % surveyed had a do not attempt resuscitation (DNAR) in place.

**Conclusions**

Staff are not better at predicting initial CPR survival rates. The public felt that they need more knowledge about CPR; the most popular learning modality was online learning. Staff opinions on whether they would want CPR are strongly influenced by the presence of a chronic disease causing a daily impact.

**Reference**

1. NCAA: Key statistics from the National Cardiac Arrest Audit 2014/2015.

## P308 Dispatcher-assisted telephone cardiopulmonary resuscitation using a French-language compression-ventilation pediatric protocol

### M. Peters^1^, S. Stipulante^1^, A. Delfosse^2^, AF Donneau^3^, A. Ghuysen ^1^

#### ^1^Department of Public Health , University of Liège, Belgium; ^2^Department of Emergency Medicine, University Hospital of Liège, Belgium; ^3^Department of Medical Biostatistics, University of Liège, Belgium

**Introduction**

Pediatric out-of-hospital cardiac arrest (OHCA) is a devastating event with low survival rates, partly due to lack of bystander cardiopulmonary resuscitation (CPR) (1). While telephone dispatcher-assisted CPR instructions (T-CPR) improve the frequency and quality of bystander CPR for OHCA in adults (2), this support remains undeveloped in pediatry. We aim to assess the effectiveness of a new pediatric T-CPR protocol in both previously trained and untrained bystanders, and secondarily, the utility of the ventilations.

**Methods**

Adults with no CPR experience were recruited in a public movie center and among bachelor nursing students in Liège district. Volunteers were randomly assigned either to «T-CPR» or to «no T-CPR» and were submitted to a 6 minutes infant OHCA scenario. CPR performance data were extracted from video and infant manikin using Laerdal Simpad® SkillReporter.

**Results**

161 volunteers were recruited and 115 met the inclusion criteria. They were distributed in 4 groups : trained unguided group (U-NG, n = 27), untrained guided group (U-G, n = 32), trained unguided group (T-NG, n = 26), trained guided group (T-G, n = 30).

T-CPR increased significantly CPR attempts (74.1 % of U-NG group performed chest compressions, compared to 100 % of other groups; p = 0,0007) and overall CPR performance, but median time to first chest compression was longer in guided groups (p < 0,0001).

The proportion of volunteers who managed to deliver ventilation during CPR was higher (p < 0,0001) in guided groups (81,2 % for U-G group and 83,3 % for T-G group) than in unguided groups (0 % for U-NG group and 46,1 % for T-NG group). While the tidal volume delivered was too high for each group, the fraction of time required to give ventilations was significant higher for guided groups.

**Conclusions**

T-CPR instructions have the potential to increase the number and the performance of bystander CPR in trained or untrained volunteers (3). Mouth to mouth ventilations was responsible for major interruptions in chest compressions. Moreover, we identified an overall tendency to hyperventilation.

**References**

1. Atkins DL et al. Improving outcomes from out-of-hospital cardiac arrest in young children and adolescents. Pediatr Cardiol. 2012; 33(3): 474–483.

2. Stipulante S et al. Implementation of the ALERT algorithm, a new dispatcher-assisted telephone cardiopulmonary resuscitation protocol, in non-Advanced Medical Priority Dispatch System (AMPDS) Emergency Medical Services centres. Resuscitation. 2014;85(2): 177–181.

3. Goto Y et al. Impact of Dispatcher‐Assisted Bystander Cardiopulmonary Resuscitation on Neurological Outcomes in Children With Out‐of‐Hospital Cardiac Arrests: A Prospective, Nationwide, Population‐Based Cohort StudyJ Am Heart Assoc. 2014; 30(3): e000499.

## P309 Dantrolene versus amiodarone for resuscitation – an experimental study

### C. Feldmann, D. Freitag, W. Dersch, M. Irqsusi, D. Eschbach, T. Steinfeldt, H. Wulf, T. Wiesmann

#### University Hospital, Philipps University Marburg, Marburg, Germany

**Introduction**

Dantrolene is a therapeutic drug for patients with malignant hyperthermia. Interestingly, dantrolene has antiarrhythmic characteristics and might be an alternative for patients with VTs (ventricular tachycardia) or VF (ventricular fibrillation). However, experimental resuscitation studies comparing dantrolene and amiodarone are still missing. Aim of this study was to evaluate ROSC (return of spontaneous circulation) rates for dantrolene versus amiodarone in a pig model of sustained cardiac arrest (8 min) under VF.

**Methods**

Ventricular fibrillation was induced in anesthetized pigs (dantrolene, n = 14 and amiodarone, n = 14 or saline, n = 10). After 8 min of untreated VF, chest compressions and ventilation were started and one of the drugs (amiodarone 5 mg kg -1, dantrolene 2.5 mg kg -1 or saline (sham)) was applied in a blinded fashion. After 4 min of CPR without epinephrine or other drugs, defibrillation with 200 J was performed. Standardized resuscitation including mechanical cpr and defibrillation according to protocol was applied in all animals. ROSC rates and cardiovascular as well as blood gas analysis and cerebral oximetry and perfusion measurements were calculated during the experiments.

**Results**

Initial ROSC rates were 10 of 14 animals in the dantrolene group vs. 5 of 14 animals in the amiodarone group (3 of 10 for saline group). However, persistence of ROSC until 120 min follow-up was shown in 6 animals of the dantrolene group, 4 animals in the amiodarone group and 2 animals of the saline (sham) group. However, results were not statistically significant between groups. Hemodynamic data and cerebral perfusion as well as cerebral oximetry were comparable between survivor animals.

**Conclusions**

Pharmacological effect of dantrolene is comparable to amiodarone regarding ROSC in our experimental model. Hemodynamic parameter did not show any relevant significance between both groups.

Future studies should evaluate dantrolene for CPR in a bundle approach including epinephrine and post-ROSC therapy protocol.

**References**

Zamiri N, Massé S, Ramadeen A, Kusha M, Hu X. Dantrolene improves survival after ventricular fibrillation by mitigating impaired calcium handling in animal models. Circulation. 2014;129(8): 875-885.

Roden DM, Knollmann BC. Dantrolene. from better bacon to a treatment for ventricular fibrillation. Circulation. 2014;129:834–836.

## P310 Long term survival and functional neurological outcome in comatose survivors undergoing therapeutic hypothermia

### N. Kongpolprom, J. Cholkraisuwat

#### Chulalongkorn University, Bangkok, Thailand

**Introduction**

Therapeutic hypothermia(TH) improves short term physical neurological outcome and survival in post-arrest patients. However, there are limited data of long term survival and functional neurological outcome in the survivors.

**Methods**

We reviewed database of post arrest patients undergoing TH who survived to discharge with consciousness (Glasgow-Pittsburgh Cerebral Performance: CPC 1-3) in our hospital from 2006 to 2014. All hospital survivors were evaluated life or death status and death date on January 31st , 2015 by checking death certificates from national registry system. We contacted the survivors or their relatives by phone or mail and scheduled a follow-up visit to evaluate their ability. The patients who were unable to visit were assessed the functional disability by phone interview or using recorded follow-up data. The functional neurological outcome was scored on a disability rating scale.[1] This scale consisted of 1) arousability awareness and responsivity, 2) cognitive ability for self-care activities, 3) dependence on others and 4) psychosocial adaptability. The level of disability was defined as the followings: 0 – none; 1 – mild; 2 to 3.5– partial; 4 to 6 – moderate; 7 to 11 – moderately severe; 12 to 16 – severe; 17 to 21 – extremely severe; 22 to 24 – vegetative state and 25 to 29 – extreme vegetative state. In addition, survival rates at 6 months, 1 and 2 years after discharge were analyzed.

**Results**

Of 51 patients treated with TH, 27 patients survived to hospital discharge. Seventeen of the hospital survivors were conscious: 6, 3 and 8 patients with CPC at discharge 1, 2 and 3, respectively. Five of them passed away later. Approximately 78.6 %, 76.9 % and 75 % of awake patients survived at 6 months, 1 and 2 years after discharge, respectively. The majority (3/5) of dead cases died within 6 months with severe functional disability (score 24-26). The patients’ disability scores were shown in table. One-third of awake patients with CPC 2 and 3 who still survived at 6 months after discharge finally recovered to normal physical and cognitive function, while most of patients with CPC1 returned to normal function or minimal disability.

**Conclusions**

Long term survival rate of conscious survivors with TH was high. Most of hospital survivors alive longer than 6 months after discharge had good functional capability.

**Reference**

1.Rappaport M, Hall KM, Hopkins K, Belleza T, Cope DN. Disability rating scale for severe head trauma patients: coma to community. Arch Phys Med Rehabil. 1982; 63:118–123.

**Table 43 (Abstract P310).** Disability Scale

No.	CPC at discharge	Status	Arousability Awareness and Responsivity	Cognitive Ability	Dependence	Psychosocial Adaptability	Total score
1	1	Death	0	3	2	2	7
2	1	Survive	0	0	1	0	1
3	1	Survive	0	1	1	1	3
4	1	Survive	0	0	0	0	0
5	1	Survive	0	0	0	0	0
6	1	Survive	0	0	0	0	0
7	2	Survive	1	3	2	2	8
8	2	Death	10	9	4	3	26
9	2	Survive	0	0	0	0	0
10	3	Death	4	9	4	3	20
11	3	Death	8	9	4	3	24
12	3	Death	9	9	4	3	25
13	3	Survive	0	3	2	3	8
14	3	Survive	0	0	0	0	0
15	3	Survive	2	2	1	3	8

## P311 Impact of kidney disease on mortality and neurological outcome in out-of-hospital cardiac arrest: a prospective observational study

### S. Beitland, E. Nakstad, H. Stær-Jensen, T. Drægni, G. Andersen, D. Jacobsen, C. Brunborg, B. Waldum-Grevbo, K. Sunde

#### Oslo University Hospital, Oslo, Norway

**Introduction**

Kidney disease after out-of-hospital cardiac arrest (OHCA) is previously incompletely described. We examined the occurrence of chronic kidney disease (CKD) and acute kidney injury (AKI) in OHCA patients, and the impact of AKI on six-months mortality and neurological outcome in patients with our without renal replacement therapy (RRT).

**Methods**

Prospective cohort study at Oslo University Hospital, Oslo, Norway, between September 8, 2010, and January 13, 2014. Resuscitated comatose OHCA patients admitted to an intensive care unit were included. Patients were treated according to a standardized treatment protocol including therapeutic hypothermia to 33 °C for 24 hours. CKD and AKI were classified according to the Kidney Disease Improving Global Outcomes (KDIGO) guidelines. Patients with previously known CKD were excluded from further detailed analyses. Main outcomes were six-months mortality and good neurological outcome defined as Cerebral Performance Category 1-2.

**Results**

Among 245 included patients (84 % males, mean age 61 years), 4 % had previously known CKD and 46 % developed AKI. The AKI patients consisted of 58 (52 %) AKI stage 1, 30 (27 %) AKI stage 2, and 24 (21 %) AKI stage 3. Overall six-months outcome revealed that 46 % died and 50 % had good neurological outcome. Compared with no kidney disease, AKI patients had significantly increased mortality (odds ratio (OR) 3.17, 95 % confidence interval (CI) 1.95-5.43, p < 0.01) and worsened neurological outcome (OR 0.29, 95 % CI 0.17-0.50, p < 0.01). AKI patients with and without RRT had comparable six-month mortality (50 versus (vs.) 61 %, p = 0.40) and good neurological outcome (44 vs. 35 %, p = 0.42), also after excluding patients where RRT was withheld due to futility.

**Conclusions**

Kidney disease occurred in about half of patients successfully resuscitated from OHCA. AKI was associated with unfavorable six-month mortality and neurological outcome; however, RRT in these patients was not.

## P312 ICU dependency of patients admitted after primary percutaneous coronary intervention (PPCI) following out of the hospital cardiac arrest

### K. Hoyland, D. Pandit

#### William Harvey Hospital, Ashford, UK

**Introduction**

Available clinical evidences indicate early PCI improves outcome for non STEMI out of hospital cardiac arrest (OHCA) patients with suspected cardiac cause [1,2]. Adoption of this approach will increase ICU admission at the regional PPCI centers. In this observational cohort study we analysed the ICU service dependency of the PPCI patients following OHCA.

**Methods**

All adult patients admitted to our intensive care unit between April 2014 and March 2015 were analysed. Patients admitted from the PPCI pathway following OHCA were compared with all other unplanned admissions during the study period. Data regarding outcome and dependency was collected from the ICNARC (Intensive Care National Audit Research Council, UK) database. Paired t-test & Chi square test were performed to estimate statistical significance levels.

**Results**

The number of OHCA-PPCI and unplanned admissions during the study period were 51 and 630 respectively. Median length of ICU stay were significantly high for the PPCI patients (4.25 vs 3.70 days, P = 0.02). Furthermore, PPCI patients had higher ICNARC severity scores (29.3 vs 20; p < 0.0001). PPCI patients had significantly higher levels of dependency, evidenced by higher levels of organ support (3.2 vs 2 organs; p < 0.0001), greater level 3 care (4.1 vs 2.6 days; p = 0.012), more days ventilated (4 vs 2.4 days; p = 0.005), and higher requirements for advanced CVS support (1.6 vs 0.6 days; p < 0.0001). There were no significant differences between dialysis requirement or hospital length of stay. Overall ICU mortality rates were higher in the PPCI group (46.9 % vs 30.7 %, p = 0.02). Despite representing only 8 % of the total number of patients admitted during the study period, PPCI patients accounted for 12 % of total ICU level 3 bed days, 13 % of ventilated days and 19 % of advanced CVS days.

**Conclusions**

Overall, patients undergoing PPCI post OHCA had significantly greater levels of dependence than other patients admitted to the ICU. The high level of dependency need to be considered when contemplating expansion of PCI services for non STEMI, OHCA patients with suspected cardiac causes.

**References**

1. Noc M et al. Invasive coronary treatment strategies for out-of-hospital cardiac arrest: a consensus statement from the EAPCI /SFL groups. Euro Intervention. 10: 31–7, 2014.

2. Anthony C et al. Cardiac catheterization is associated with superior outcomes for survivors of out of hospital cardiac arrest: Review and meta-analysis. Resuscitation. 85: 1533–40, 2014.

## P313 Prognostic indicators and outcome prediction model for patients with return of spontaneous circulation from cardiopulmonary arrest: comprehensive registry of in-hospital intensive care on OHCA survival (critical) study in Osaka, Japan

### K. Hayakawa

#### Kansai Medical University Takii Hospital, Moriguchi, Japan

**Introduction**

We established a multi-center, prospective cohort that included Utstein data in prehospital and treatment contents and data after hospital arrival. The purpose of this study was to determine the most important indicators of prognosis in patients with return of spontaneous circulation (ROSC) following out-of-hospital cardiopulmonary arrest (OHCA) and to develop a best outcome prediction model.

**Methods**

All consecutive patients who were suffering from OHCA and who were then transported to institutions participating in this registry were prospectively recorded in July 2012 to December 2013. Criteria for inclusion of this study were a witnessed cardiac arrest, age greater than 17 years, presumed cardiac origin of the arrest, and successful ROSC. Multivariate logistic regression (MLR) analysis was used to develop the best prediction model. The dependent variables were favorable outcome (cerebral-performance category: CPC 1-2) and poor outcome (CPC 3-5) at 1 month after the event. The explanatory variables were used concerning patient characteristics and resuscitation.

**Results**

Subjects comprised 147 patients in VF and 177 patients with pulseless electrical activity (PEA)/asystole. The percentage of favorable outcomes was 42.2 % (62/147) in VF and 11.3 % (20/177) in PEA/asystole. The most important prognostic indicators of favorable outcome founded by MLR were age (p < 0.01), time from collapse to ROSC (TROSC) (p < 0.01), base deficit (p = 0.06), presence of by-stander cardiopulmonary resuscitation (p = 0.09) for VF and age (p = 0.05), TROSC (p < 0.01), and base deficit (p < 0.05) for PEA/asystole. Areas under the receiver-operating characteristic curve were 0.866 for VF and 0.896 for PEA/asystole respectively.

**Conclusions**

A model based on four selected indicators showed a high predictive value for favorable outcome in OHCA patients with ROSC.

## P314 Cerebral oxygen saturation during resuscitation in a porcine model of cardiac arrest

### E. Oloktsidou, K. Kotzampassi, B. Fyntanidou, S. Patsatzakis, L. Loukipoudi, E. Doumaki, V. Grosomanidis

#### Aristotle Medical School, Thessaloniki, Greece

**Introduction**

Near infrared spectrometry (NIRS) could be a useful non-invasive monitoring of cerebral perfusion during cardiopulmonary resuscitation (CPR). The aim of our study was to investigate and evaluate NIRS during CPR in a porcine model of cardiac arrest (CA).

**Methods**

24 pigs under general anesthesia and mechanical ventilation were included in this study. In all study animals ventricular fibrillation was induced with application of electrical current via a pacing wire. After 7 min of CA, CPR was initiated with the use of LUCAS mechanical device and after 5 more min, initial CA-rhythm was analyzed and advanced life support interventions were provided according to the 2015 ERC guidelines. Regional cerebral oxygenation (rSO2) was recorded before and after resuscitation in 1 min intervals by INVOS OXIMETER via a Somasensor electrode placed on pigs heads. Pigs were divided into two groups depending on return of spontaneous circulation or not, G-A: no ROSC (n = 15) & G-B: ROSC (n = 9). G-A & G-B were compared at 6 Phases: P0 (baseline), P1 (7 min after CA-no flow), P2 (5 min after CPR initiation) and thereafter every 5 min (P3-P5). For the statistical analysis repeated measures ANOVA was used (SPSS21).

**Results**

Duration of CPR was 40 min for G-A and until ROSC for G-B. Directly after CA, a rSO2 decrease was recorded, which remained for the whole resuscitation attempt (Table). There weren’t any statistical differences between the study groups. After ROSC, rSO2 remained decreased and increased gradually over time.

**Conclusions**

According to our study results, rSO2 alterations are useful in identifying CA immediately but do not provide any other useful information during CPR.

**Fig. 82 (Abstract P314).**
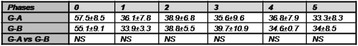
rSO2 alterations over time

## P315 Presumption of cardiopulmonary resuscitation for sustaining cerebral oxidation using regional cerebral saturation of oxygen: observational cohort study (press study)

### H. Yasuda

#### Japanese Red Cross Musashino Hospital, Tokyo, Japan

**Introduction**

While a new 2015 AHA guideline of cardiopulmonary resuscitation recommends cardiac compression should be performed on the sternal bone at a level of bust point, there is individual difference in the heart position. The ratio of real-time low-oxygenized cerebral blood circulation represented by rSO2 (regional cerebral oxygen saturation) can be non-invasively and quantitatively measured using a near infrared-red spectroscopy (NIRS) sensor placed at the forehead in which higher absorption of near infrared-red ray occurs in low-oxygenized blood. Therefore, rSO2 potentially enables us to monitor the effective cardiopulmonary resuscitation. The purpose of this study was to validate rSO2 monitoring on effectiveness of chest compression by investigation of relationship between arterial blood pressure and rSO2 in patients with out-of-hospital cardiac arrest (OHCA).

**Methods**

This study was a prospective observational study conducted at a tertiary emergency medical care center in Japan. Systolic arterial pressure (SAP) and mean arterial pressure (MAP) through invasive arterial pressure monitoring, and rSO2 were measured at every 3 minutes during the cardiopulmonary resuscitation of OHCA patients, and the relationships between absolute values in the measurements were compared using the two types of coefficient of correlation, within and between patients in order to perform the statistical analysis considering repeated measurement taken from a single patient.

**Results**

There were 38 patients out of total 169 patients with OHCA who fulfilled the inclusion criteria of the study and 16 patients were finally included. Total measurement points of these patients were 29 points. Median value of the patient age was 82 years old (IQR 71-87). Male patient number was 14 (88 %), and 81 % patients had witnesses at the time of cardiopulmonary arrest. Two types of coefficient of correlation between arterial pressure and rSO2 showed that the within patients f coefficient of correlation between MAP and rSO2 was 0.20 (95 % CI:-1.8-0.53), but the coefficient of correlation between patients was 0.82 (95 % CI:0.14-0.72).

**Conclusions**

There was correlation between rSO2 and MAP during sternal chest compression in patients with OHCA indicating that rSO2 may be useful to monitor cerebral blood flow during the chest compression. Our results suggest that sternal chest compression under rSO2 monitoring improves outcome of the patients with OHCA.

## P316 EEG reactivity in patients after cardiac arrest: a close look at stimuli

### MM Admiraal^1^, M. Van Assen^1^, MJ Van Putten^2^, M. Tjepkema-Cloostermans^2^, AF Van Rootselaar^1^, J. Horn^1^

#### ^1^Academic Medical Center, Amsterdam, Netherlands; ^2^Medisch Spectrum Twente, Enschede, Netherlands

**Introduction**

Testing EEG reactivity is a standard procedure during EEG registration in unconscious ICU patients. It is suggested to use EEG reactivity as a marker for poor outcome after cardiac arrest [1]. However, there is no clearly defined protocol how to test reactivity. In this study we investigated which stimulus type most effectively evokes a cortical response in cardiac arrest patients.

**Methods**

Prospective cohort study in patients monitored with continuous EEG after cardiac arrest in two Dutch ICUs. Twice a day a very strict stimulus protocol including five stimulus types (in this order: clapping, yelling patients name, passive eye opening, nasal tickle and sternal rub) was executed. Each stimulus was applied 3 times for 5 s with an interval of 30s. Each stimulus response was individually scored reactive, non-reactive, doubtful or unscorable (e.g. too much noise) by three independent, blinded raters (JH, MvP, MT-C). Reactivity was defined as a change in the amplitude or frequency in the EEG upon stimulation, excluding muscle artifacts. Iso-electric EEGs were excluded beforehand. Stimulus responses on which two out of three raters agreed were included in the analysis.

**Results**

The stimulation protocol was executed 72 times in 29 patients, resulting in 1050 independently rated stimulus responses. 42 stimuli were excluded because of disagreement between raters. The intraclass correlation coefficient was 0.38. The response to 91 stimuli in 19 patients was found reactive. The stimulus inducing EEG reactivity most often was clapping, 29 %. Passive eye opening least often induced EEG reactivity, 16 %.

**Conclusions**

A sudden loud noise stimulus such as clapping seems to most effectively evoke a cortical response in patients after cardiac arrest. We suggest integrating this stimulus in the standard set of stimuli for testing EEG reactivity. More studies are needed to determine the relevance for prognostication.

**Reference**

1. Sandroni C, et al. Prognostication in comatose survivors of cardiac arrest: An advisory statement from the European Resuscitation Council and the European Society of Intensive Care Medicine. Intensive Care Med. 2014; 40(12):1816–1831.

## P317 Prognostic value of neuron-specific enolase after cardiac arrest

### F. Ragusa^1^, A. Marudi ^2^, S. Baroni^2^, A. Gaspari^1^, E. Bertellini^2^

#### ^1^University Modena, Modena, Italy; ^2^Nuovo Ospedale Civile Sant’Agostino Estense, Modena, Italy

**Introduction**

The serum concentration of neuron-specific enolase (NSE) has been established as a highly specific predictor of poor outcome after cardiac arrest but no clear cut-off has been identified.

**Methods**

We prospectively included all out-of-hospital cardiac arrest patients admitted in our intensive care unit (ICU) from 2011 to 2014. They were submitted to target temperature management (34 °C) for 24 hours. Outcome was assessed according to the Cerebral Performance Category (CPC) at ICU discharge. CPC 1 to 3 was considered as good outcome and CPC 4 to 5 as poor outcome. Recent guideline suggest to sample NSE at 72 hours after cardiac arrest.

**Results**

132 patients were admitted from 2011 to 2014. 54 patients presented good outcome while 78 patients presented poor outcome according to CPC. The NSE serum concentration was sampled at the 3rd days after cardiac arrest.

In the first group NSE value was 22.3 mcg/l. In the second group NSE value was 74,5 mcg/l.

**Fig. 83 (Abstract P317).**
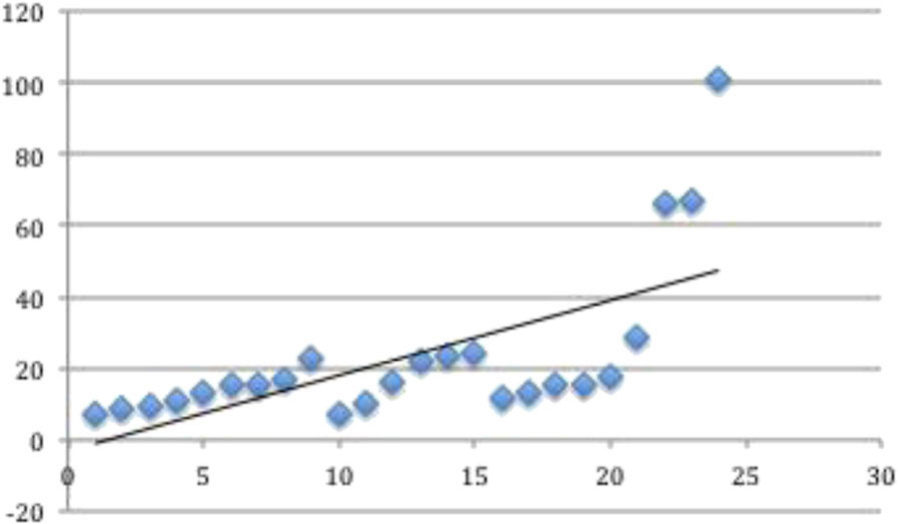
NSE distribution in good outcome patients

Nevertheless 8 patients presented poor outcome and NSE serum concentration < 40 mcg/l and 3 patients presented good outcome and NSE > 60 mcg/l (Fig. 83).

**Fig. 84 (Abstract P317).**
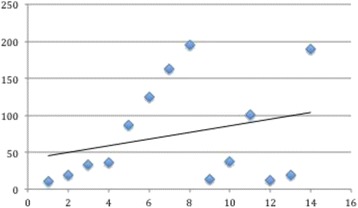
NSE distribution in poor outcome patients

We observed great variability of NSE value in patients with poor outcome (Fig. 84).

**Conclusions**

NSE could be an important biomarker of poor outcome after cardiac arrest. Further investigation is necessary to clarify timing for sample and cut off.

**Reference**

Jerry P. Nolan, et al. European Resuscitation Council and European Society of Intensive Care Medicine Guidelines for Post-resuscitation Care 2015. Section 5 of the European Resuscitation Council Guidelines for Resuscitation 2015. Resuscitation. 2015; 95 L 202–222

## P318 Correlation between electroencephalographic findings and serum neuron specific enolase with outcome of post cardiac arrest patients

### A. Taha, T. Abdullah, S. Abdel Monem

#### Faculty of Medicine Alexandria University, Alexandria, Egypt

**Introduction**

Anoxic–ischemic encephalopathy after cardiac arrest is a common cause of coma requiring intensive care of survivors. Certain malignant electroencephalographic (EEG) patterns have been shown to correlate with poor prognosis. Neuron specific enolase (NSE) released after cardiac arrest is regarded as a severity indicator of postanoxic neuronal injury. We investigated the EEG findings in post-cardiac arrest patients and correlated these findings with NSE levels and outcome scales.

**Methods**

34 Egyptian patients after resuscitation from cardiac arrest were subjected to, EEG, NSE measurement, and Glasgow outcome scale (GOS).

EEG data EEG monitoring was done within the first day of admission for about 30 minutes and repeated on the seventh days after cardiac arrest. EEG classification on days 1 and 7 post-arrest according to Young et al. (1)

I. Delta/theta > 50 % of recording
II. Triphasic waves
III. Burst-suppression pattern: with or without epileptiform activity
IV. Alpha/theta/spindle pattern coma (no reactivity).
V. Suppression (generalized) : <20 microvolts.

NSE will be sampled at both day 1 and 3 after cardiac arrest. Serum concentrations of NSE will be measured using ELISA technique.

**Results**

There was statistically significant negative correlation between EEG coma scale on day 1 and 7 with GOS ( p > 0.001). There was statistically significant negative correlation between NSE on day 1 and 3 with GOS (p > 0.001) (Table 44).

There was statistically significant positive correlation between EEG scale on day 1 and 7 with neuron specific enolase on day 1 and 3 (Table 45).

**Conclusions**

In post-cardiopulmonary arrest patients, there is significant correlation between EEG coma scale and Glasgow outcome scale, as certain malignant EEG patterns are correlated with high serum levels of NSE and they are good predictors of poor outcome of these patients.

**Reference**

1. Young GB, McLachlan RS, Kreeft JH, Demelo JD. An electroencephalographic classification for coma. Can J Neurol Sci. 1997; 24(4): 320–325.

**Table 44 (Abstract P318).** ᅟ

	GOS (r)	GOS (p)
EEG SCALE 1ST DAY	-0.670	<0.001
EEG SCALE 7TH DAY	-0.722	<0.001
NSE 1ST DAY	-0.624	<0.001
NSE 3RD DAY	-0.673	<0.001

**Table 45 (Abstract P318).** ᅟ

NSE 1ST DAY (P)	NSE 3RD DAY (P)	EEG SCALE 1ST DAY
0.002	<0.001	EEG SCALE 7TH DAY
<0.001	<0.001	

## P319 Introduction of a targeted temperature management strategy following cardiac arrest in a district general hospital intensive care unit

### S. Alcorn, S. McNeill, S. Russell

#### Victoria Hospital, Kirkcaldy, UK

**Introduction**

We report the experience of a 10-bed intensive care unit (ICU) introducing a targeted temperature management (TTM) strategy following cardiac arrest based on the TTM trial investigation protocol [1], including pre- and post-intervention data. Our unit averages 4 cardiac arrest cases a month; at this low frequency, and given recent updates to TTM evidence, we were concerned that our temperature management practice could be inconsistent or outdated.

**Methods**

Cases were identified retrospectively from the Scottish national ICU audit database. All patients with a diagnosis of cardiac or respiratory arrest were selected for full case note review. Baseline survey from May-November 2014 yielded 21 cases of which 3 sets of notes were unobtainable. Data collected included documented target temperature (TT), use of active cooling, hourly temperature data over the first 24 hours in ICU, and eventual outcome.

A new TTM strategy, based on the TTM study protocol, was then introduced in February 2015. This was presented at the local ICU meeting and copies made available at each bed space for staff to refer to. Subsequently, all cases from March-September 2015 were identified by the same method and the same data set collected, totalling 20 cases of which 1 set of notes was unobtainable.

**Results**

Baseline data indicated no consistent TTM strategy with documented TT ranging from 35 to 37 °C, with no target documented in 50 %. Only 28 % were actively cooled, while 50 % were pyrexial (defined as >37.5 °C) and all but one patient (94 %) exceeded the TTM target of 36 °C by at least 0.5 °C. Prior to intervention, the average time spent above 37.5 °C was 5.8 hours and the average time above 36.5 °C was 11.8 hours.

Following the intervention, TTs ranged from 36-36.5 °C with an increased proportion of patients (12, 63 %) having the desired TT of 36 °C. A decreased proportion, 26 % regrettably still had no documented TT, however significantly more patients (13, 68 %) were actively cooled. Only 4 patients (21 %) were pyrexial, while 12 still exceeded 36.5 °C (63 %). Encouragingly, after the introduction of the protocol, the average time spent above 37.5 °C was 1.15 hours (80 % reduction) and the average time above 36.5 °C was 4.2 hours (64 % reduction).

**Conclusions**

Following changes to TTM in recent years, our promising initial data show that introduction of a TTM strategy in a small unit with infrequent cases is achievable, and compliance with targets can be significantly improved with a simple intervention protocol.

**Reference**

1. Nielsen N, et al. Targeted Temperature Management at 33 °C versus 36 °C after Cardiac Arrest. N Eng J Med. 2013; 369: 2197-2206.

## P320 The evolution of cerebral oxygen saturation in post-cardiac arrest patients treated with therapeutic hypothermia

### W. Eertmans, C. Genbrugge, I. Meex, J. Dens, F. Jans, C. De Deyne

#### Ziekenhuis Oost-Limburg, Genk, Belgium

**Introduction**

Regardless of the recent advances in cardiopulmonary resuscitation and post-resuscitation care, most patients who die during their hospital stay after out-of-hospital cardiac arrest (OHCA) decease due to post-anoxic neurological injury. Near infrared spectroscopy (NIRS) provides information on regional brain oxygenation by measuring the regional cerebral oxygen saturation (SctO2) and offers the possibility to determine changes in the balance between oxygen delivery and uptake in the frontal lobe of the brain. The aim of the study was to monitor SctO2 for 48 hours in OHCA patients during targeted temperature management (TTM).

**Methods**

A prospective, observational study was performed during therapeutic hypothermia (TH; 24 hours at 33 °C, followed by rewarming at 0.3 °C/h over 12 hours) in 107 successfully resuscitated OHCA patients. SctO2 was continuously monitored with NIRS for 48 hours using the FORE-SIGHT^TM^ (CAS Medical systems, Branford, CT, USA). The Cerebral Performance Category (CPC) scale was used to define patient’s outcome at 180 days after OHCA, where CPC 1-2 and CPC 3-5 represented a good and poor outcome, respectively. Data are reported as mean ± SD. A student t-test was performed as appropriate and a mixed model was created to analyze continuous SctO2 data over time.

**Results**

While 50 patients (47 %) survived with a good neurological outcome, 57 patients (53 %) corresponded to a CPC score of 5. The mean SctO2 at ICU admission was 64 % ± 7 for survivors compared to 66 % ± 6 for non-survivors (p = 0.184). After initiation of TH, an important decrease was observed in both groups with a lowest SctO2 of 62 % at hour 3 for survivors and 61 % at hour 5 for non-survivors (p = 0.432). This was followed by a progressive increase towards baseline values. With respect to non-survivors who regained baseline SctO2-values at 22 hours (66 % ± 6), survivors already reached baseline values within 12 hours (65 % ± 6). All SctO2-values were fitted in a linear mixed model and a significant time effect was observed during the course of SctO2 after adjustment for age and gender (p < 0.001). We found that SctO2-values of non-survivors followed a linear course over time, whereas SctO2-values of survivors could be fitted in a logarithmic curve.

**Conclusions**

TTM following OHCA is accompanied by fluctuations in the balance between oxygen delivery and supply to the brain as reflected by changes in SctO2. Moreover, the course of SctO2 over time is significantly different as SctO2-values of survivors follow a logarithmic course compared to a linear one for non-survivors.

## P321 Prognostic factors and neurological outcomes of therapeutic hypothermia in comatose survivors from cardiac arrest : 8-year single center experience

### J. Cholkraisuwat, N. Kongpolprom

#### Chulalongkorn University, Bangkok, Thailand

**Introduction**

Therapeutic hypothermia(TH) improves neurological outcome and survival in comatose patients with ROSC after cardiac arrest [1]. Our institute has developed hypothermia protocol and used it as a standard practice for 8 years. We aimed to evaluate clinical outcomes of patients undergoing TH and analyze factors associated with these outcomes.

**Methods**

We retrospectively reviewed baseline characteristics, cooling practice and clinical outcomes of post-arrest patients treated with TH in our institute from 2006 to 2014. Neurological status measured by the Cerebral Performance Categories Scale (CPC) and survival rate were evaluated. Prognostic factors for good neurological recovery and survival were analyzed. Continuous data were reported as medians and percentage. Chi-square and Mann- Whitney U test were used to compare data of patients with favorable outcomes and those with unfavorable outcomes.

**Results**

TH was applied in 51 comatose survivors, including 40 survivors (78.4 %) from out-of-hospital arrest. The primary cardiac rhythm for the majority (40/51, 78.4 %) was non-shockable rhythm. The median age of patients was 59 years. Overall, 52.9 %(27/51) of patients survived. The median hospital LOS was 13 days. Nine of 27 survivors at discharge had good recovery (CPC1 = 6 and CPC2 = 3) Eight patients had severe disability(CPC3) and 10 patients were in a vegetative state (CPC4). Free of vasopressor was the only prognostic factor for good neurological recovery (p = 0.012) and survival (p < 0.001), while the shorter time from collapse to ROSC was associated with favorable outcome (p = 0.044). Additionally, duration of less than 6 hours from ROSC to achieved temperature and protocol completion were associated with survival (p = 0.038 and p = 0.027, respectively). Cardiac rhythm, location of arrest, target temperature attainment, rebound hyperthermia and age were not associated with any clinical outcome.

**Conclusions**

Regardless of cardiac rhythm and arrest location, approximately 53 % of post arrest patients with TH survived and 33 % of survivors had favorable neurological outcome. The factors associated with the good recovery were free of vasopressor and time from collapse to ROSC, while factors associated with survival were free of vasopressor after successful resuscitation, time to target temperature of less than 6 hours and protocol completion.

**Reference**

1. Alzaga A, Cerden M, Varon J. Therapeutic hypothermia. Resuscitation. 2006; 70(3): 369-380.

## P322 Adherence to targeted temperature management after out of hospital cardiac arrest

### B. Avard^1^, R. Burns^2^

#### ^1^The Canberra Hospital, Hughes, ACT, Australia; ^2^ANU Medical School, Canberra, Australia

**Introduction**

Targeted temperature management after out of hospital cardiac arrest remains a recommended therapeutic approach by the Australian Resuscitation Council. As one of the top five reasons for admission to our major tertiary Australian Intensive Care Unit, we aimed to audit our current practice with regards to time to target temperature, time at target temperature and rewarming practices.

**Methods**

We conducted a retrospective review of 54 patients admitted to our Intensive Care Unit after return of spontaneous circulation (ROSC) from out of hospital cardiac arrest, collecting data from our electronic medical records system ‘Metavision’ and analysed using SPSS v22.0. Ethics approval was obtained.

**Results**

Only 16 % of patients achieved target temperature within 4 hours, with 25 % still not achieving target within 16 hours of ROSC and 44 % were maintained at the target for 12-24 hours. The target was chosen as 32-24 degrees in 73 % of patients. Rewarming occurred at a a rate of <0.5 degrees per hour in less than 50 % of the patients. Despite these findings there was no correlation between neurological outcome and adherence or not to the current evidence based recommendations.

**Conclusions**

This data shows that our unit has been unable to consistently apply targeted temperature management in “real world practice” according to evidence based guidelines, however this has not resulted in adverse outcomes for our patient population.

**References**

Nielsen N, et al. Targeted temperature management at 33 °C versus 36 °C after cardiac arrest. N Engl J Med. 2013; 369(23): 2197-2206.

Wise MP, et al. Targeted temperature management after out-of-hospital cardiac arrest: certainties and uncertainties. Crit Care. 2014, 18(4): 459.

Nunnally ME, et al. Targeted temperature management in critical care: a report and recommendations from five professional societies. Crit Care Med. 2011; 39(5): 1113-1125.

Nolan JP, et al. Therapeutic Hypothermia After Cardiac Arrest. Circulation. 2003; 108: 118-121.

**Fig. 85 (Abstract P322).**
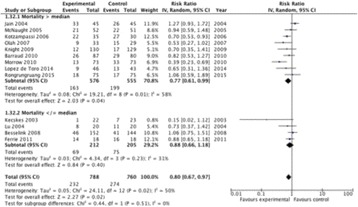
ᅟ

## P323 Implementation of a therapeutic hypothermia protocol for comatose survivors of out-of-hospital cardiac arrest

### A. Patarchi, T. Spina

#### “Spirito Santo” Hospital, Pescara, Italy

**Introduction**

Mild therapeutic hypothermia (MTH) improves neurological outcome and survival after out-of-hospital cardiac arrest (OOH-CA) from TV/FV [1]. However, this technique is not universally accepted, partially due to technical difficulties related with the implementation of practice protocols [2]. We present our experience over one year since the introduction of an institutional MTH protocol, testing its feasibility.

**Methods**

We cooled comatose patients (unable to respond to verbal commands) survived to OOH-CA from TV/FV since the implementation of a MTH protocol between May 2014 and May 2015 at Pescara Spirito Santo Hospital, a second-level hospital serving 300000 people. MTH was induced after ROSC by intravenous 70 ml/Kg Ringer’s Lactate over 30′ and maintained, after ICU admission, by a temperature-regulated surface device (Arctic Sun®, Medival, Padova, Italy; target temperature 33 °C, 0.5 °C/hours). Rewarming started 24 hours from induction (target 36.5 °C, 0.5 °C/hours); device pads were left on site for 24 hours after normothermia was achieved. Neurological sequelae were investigated before ICU discharging (clinical evaluation, CT scan and EEG) and 3 months after, by telephonic interview.

**Results**

6 patients met protocol inclusion criteria (5 males, mean age 51.7). Mean GCS value at ROSC was 4/15. MTH was started in ED (4 patients) or in ambulance (2). 4 patients underwent emergency coronarography. Mean core temperature at ICU admission was 35.3 °C. Mean time to target temperature was 320′, bradycardia was common in this phase (6/6), as were hypokalemia (4/6) and metabolic acidosis (4/6). Mean active rewarming time was 375′. Mild hyperkalemia occurred in 2 cases, while a case of hyperthermia (38 °C) needed pads repositioning. All patients were discharged alive, 4 of them free from apparent neurological sequelae (1 agitation, 1 confusion and amnesia). Mean ICU stay was 7.2 days. At 3 months, all patients were at home and free from neurological alterations.

**Conclusions**

The study points out feasibility of a MTH protocol implementation in this particular hospital setting. We overcame difficulty to rapidly induce and safely maintain MTH mixing two cooling techniques: times of induction and rewarming were acceptable, maintenance phase was characterized by temperature stability and lack of life threatening events, and clinical outcome was encouraging.

**References**

1. Bae KS, et al. The effect of mild therapeutic hypothermia on good neurological recovery after out-of-hospital cardiac arrest according to location of return of spontaneous circulation: a nationwide observational study. Circulation 2015 ; 89: 129-136.

2. Toma A, et al. Perceived barriers to therapeutic hypothermia for patients resuscitated from cardiac arrest: a qualitative study of emergency department and critical care workers. Crit Care Med 2010; 38(2): 504-509.

## P324 Factors associated with ventilator weaning after targeted temperature management for cardiac arrest patients in japan

### H. Tanaka, N. Otani, S. Ode, S. Ishimatsu

#### St Lukes International Hospital, Akashi-Chou Chuo-Ku, Japan

**Introduction**

Out of hospital cardiac arrest (OHCA) patients treated with targeted temperature management (TTM) may have substantial difficulty in ventilator weaning due to multiple organ failure, including post-TTM neurologic injury. Ability to predict the clinical course of TTM patients regarding duration of ventilation is important for clinical decision-making for both safer extubation and planning early tracheostomy. However, predictive factors of ventilator weaning after TTM remain unclear. Hypothesizing that weaning difficulty is associated with admission resuscitation conditions, we explore whether failure to wean may also be predicted at admission. The purpose of this study is to examine which factors predict ventilator weaning difficulty.

**Methods**

We performed a retrospective cohort study of OHCA patients brought to the emergency room at St. Luke fs International Hospital in Tokyo, Japan, who underwent TTM between January 2006 and July 2015. Primary outcome was days to weaning from admission to the intensive care unit. Using the electronic medical record, we collected patient characteristics, resuscitation conditions, and examination data at admission. After characterizing weaning success using descriptive statistics, the relationship between ventilator weaning during hospitalization and resuscitation conditions were assessed with Cox regression.

**Results**

Of 115 OHCA patients who completed TTM, median time to weaning from ventilation was 6 days (4-8: IQR). 98 patients (85 %) were weaned within 2 weeks after admission. Earlier weaning was significantly associated with age (HR, 0.96; 95 % CI, 0.94 to 0.97) and motor response on admission (HR, 1.56; 95 % CI, 1.01 to 2.41). Though not significantly associated with weaning, ventricular fibrillation and asystole showed a trend towards early and later weaning, respectively, compared to Pulseless Electrical Activity.

Conclusions

A large majority of TTM patients can be weaned from ventilation within 2 weeks. Older age and absence of motor response at admission are related with prolonged mechanical ventilation. Early tracheostomy planning should be considered for patients after achievement of TTM who are older and does not show motor response in ER.

**Reference**

1. Okada K, Ohde S, Otani N, Ishimatsu S. Prediction protocol for neurological outcome for survivors of out-of-hospital cardiac arrest treated with targeted temperature management. Resuscitation. 2012; 83(6): 734-739.

## P325 Differential activation of c-fos in paraventricular nuclei of the hypothalamus and thalamus of the rat following myocardial infarction

### J. Cho, J. B. Moon, C. W. Park, T. G. Ohk, M. C. Shin, M. H. Won

#### Kangwon National University, Chuncheonsi, South Korea

**Introduction**

c-Fos is well used to detect a pathogenesis in CNS disorders. We examined changes in c-Fos immunoreactivity in the paraventricular nucleus of the hypothalamus (PVNT) and paraventricular nucleus of the thalamus-(PVNT) after myocardial infarction (MI) in rats.

**Methods**

Infarction in the left ventricle was examine by Massoni’s trichrome staining. Neuronal degeneration (damage/death) was examined for 56 days after MI using cresyl violet (CV) and Fluoro-Jade B (F-J B) histofluorescence staining. Changes in C-Fos immunoreactivity were examined by immunohistochemistry for c-Fos.

**Results**

The average infarct size of the left ventricle circumference was about 44 % after MI. Neuronal damage/death was not detected in both PVNH and PVNT after MI. c-Fos immunoreactive (+) cells were hardly found in both nuclei of the sham-group. However, c-Fos + cells were increased in both nuclei after MI and peaked in the PVNH and PVNT 3 days and 14 days, respectively, after MI. At 56 days after MI, c-Fos + cells were barely found in both nuclei.

**Conclusions**

These results show that MI dramatically induced c-Fos immunoreactivity in the PVNT and PVNT and suggest that the increase of c-Fos expression may be associated with brain stress associated with MI.

## P326 Monitoring of cTroponin I in patients with acute ischemic stroke - predictor of inhospital mortality

### S. Dakova, Z. Ramsheva, K. Ramshev

#### Military Medical Academy , Sofia, Bulgaria

**Introduction**

Cerebrovascular diseases are leading cause of morbidity, mortality and disability [1]. Elevated troponin levels after AIS are common and are associated with increased risk of death and cardiac complications [2]. The aim of these study was to investigate if elevated levels of serum cTnI in acute Ischemic stroke patients are able to predict higher inhospital mortality.

**Methods**

We retrospectively investigated 218 patient between 11.2012 and 11. 2014. They were admitted in ICU and diagnosed as (AIS) according to the TOAST classification. They were 8.51 % of total 2561 patients hospitalized in the ICU in the same period. Brain CT or MRI were used on admission. The severity of neurological deficit were scoring based on NIHSS. The functional condition of patients was assessed on discharge by modified Rankin scale (mRS). Troponin I was monitored on the 1st, 3rd and 5th day. Inclusion criteria were age over 18,AIS less than 24 hours, CT/MRT on admission and Troponin I on 1st, 3rd and 5th day. Exclusion criteria were SAH or ICH renal failure, Acute coronary syndrome, Acute PE.

**Results**

Patients were divided into two groups-discharged alive and died in hospital. The average age of patients was 75,27 ± 12,6 (SD) years. Men’s age was 72,64 ± 13.50(SD)years, and women’s 75,27 ± 12,67 (SD) years. In hospital mortality among patients were 43.6 % (95/218). The patients discharged alive were 56.4 %(123/218).The cTnI was investigated on the 1st, 3rd and 5th day. According to the laboratory test results established upper limit of cTnI is 0.06 mg/l. We found higher levels on admission in 47.2 % (103/218), on the 3rd day in 54,6 % (119/218) and on the 5th day in (93/218). Among all patients with high cTnI in hospital mortality was 70.9 %(73/103)on admission, on the 3rd day 76.5 % (91/119), and 82.8 % (77/93). We used nonparametric method for comparing the medians of two variables. Statistically significant differences in the levels of cTnI were found in group died in hospital. About the first and fifth days the cut off points established by ROC analysis were 0,075 mg/l, while about the third was 0,085 mg/l (Area = 0.771,CI = 0.704-0.837). The sensitivity of the method was 74.16 % and specificity 76.42 % for the first measurement, 86.52 % and 78.05 % for the third and for the fifth day 77.53 % and 88.62 %.

**Conclusions**

The study showed increased levels of cTnI in patients with AIS died inhospital. Those results gave us the grounds to offer routine monitoring of cTnI for early stratification of high risk patients.

**References**

1. WHO, The 10 Causes of Death. 310.Geneva, 2011

2. Kerr GD, et al. Elevated troponin after stroke: a systematic review. Cerb Dis. 2009; 28(3): 220–226.

## P327 Hyperthermic preconditioning severely accelerates neuronal damage in the gerbil ischemic hippocampal dentate gyrus via decreasing sods expressions

### J. Cho, J. B. Moon, C. W. Park, T. G. Ohk, M. C. Shin

#### Kangwon National University, Chuncheonsi, South Korea

**Introduction**

It is well known that neurons in the dentate gyrus (DG) of the hippocampus are resistant to short period of ischemia. Hyperthermia is a proven risk factor for cerebral ischemia and can produce more extensive brain damage and related with mortality rates. The aim of this study was to examine the effect of hyperthermic conditioning (H) on neuronal death, gliosis and expressions of SODs as anti-oxidative enzymes in the gerbil DG following 5 min-transient cerebral ischemia.

**Methods**

The animals were randomly assigned to 4 groups: 1) (N + sham)-group was given sham-operation with normothermia (N); 2) (N + ischemia)-group was given 5 min-transient ischemia with N; 3) (H + sham)-group was given sham-operation with H; 4) (H + ischemia)-group was given 5 min-transient cerebral ischemia with H. H (39 ¡¾ 0.5¨¬C) was induced by subjecting the animals to a heating pad for 30 min before and during the operation.

**Results**

In the (N + ischemia)-groups, a significant neuronal death was observed in the polymorphic layer (PL) from 1 day after ischemia-reperfusion. In the (H + ischemia)-groups, neuronal death was also observed in the PL from 1 day post-ischemia; the degree of the neuronal death was severer than that in the (N + ischemia)-groups. In addition, we examined the gliosis of astrocytes and microglia using anti-glial fibrillary acidic protein (GFAP) and anti- ionized calcium-binding adapter molecule 1 (Iba-1). GFAP+ and Iba-1+ glial cells were much more activated in the (H + ischemia)-groups than those in the (N + ischemia)-groups. On the other hand, immunoreactivities and levels of SOD1 rather than SOD2 were significantly lower in the (H + ischemia)-groups than those in the (N + ischemia)-groups.

**Conclusions**

In brief, on the basis of our findings, we suggest that cerebral ischemic insult with hyperthermic conditioning brings up severer neuronal damage and gliosis in the polymorphic layer through reducing SOD1 expression rather than SDOD2 expression in the DG.

## P328 Failure in neuroprotection of remote limb ischemic post conditioning in the hippocampus of a gerbil model of transient cerebral ischemia

### J. Cho, J. B. Moon, C. W. Park, T. G. Ohk, M. C. Shin

#### Kangwon National University, Chuncheonsi, South Korea

**Introduction**

Remote ischemic post conditioning (RIPoC) has been proven to provide potent protection of the heart and brain against ischemia-reperfusion injury. However, despite the evidence of cerebral protection with RIPoC is compelling, RIPoC-mediated neuroprotection against transient cerebral ischemic damage insult is still mired in controversy. In this study, we examined the effect of RIPoC induced by sublethal transient hind limb ischemia neuronal death in the hippocampus following 5 min of transient cerebral ischemia in gerbils.

**Methods**

Animals were randomly assigned to sham-, ischemia-, sham plus (+) RIPoC- and ischemia + RIPoC-groups. RIPoC was induced by three cycles of 5 min and 10 min occlusion-reperfusion of both femoral arteries at predetermined points in time ( 0, 1, 3, 6, 12 and 24 h after transient cerebral ischemia). CV staining, F-J B histofluorescence staining and NeuN immunohistochemistry were carried out to examine neuroprotection in the RIPoC-mediated hippocampus 5 days after ischemia-reperfusion.

**Results**

In the ischemia-group, we found a significant loss of pyramidal neurons in the stratum pyramidale (SP) of the hippocampal CA1 region at 5 days post-ischemia compared with the sham-group. In all of the ischemia + RIPoC-groups, the loss of pyramidal neurons in the CA1 region at 5 days post-ischemia was not different from that in the ischemia-group.

**Conclusions**

Our present findings indicate that RIPoC does not prevent hippocampal CA1 pyramidal neurons from neuronal death induced by transient cerebral ischemia.

## P329 Brain death and admission diagnosis in neurologic intensive care unit, a correlation?

### A. Marudi ^1^, S. Baroni^1^, A. Gaspari^2^, E. Bertellini^1^

#### ^1^Nuovo Ospedale Civile Sant’Agostino Estense, Modena, Italy; ^2^University Modena, MODENA, Italy

**Introduction**

Aim of the study is to evaluate which acute brain injury (ABI) was correlated with brain death (BD) in our neurologic intensive care unit (NICU).

**Methods**

All dead patients for ABI in the NICU of the Modena Hospital were included from January 1st 2009 to November 30th 2015. Data were collected from the transplant register DONOR ACTION of National Transplant Service.

**Results**

**Fig. 86 (Abstract P329).**
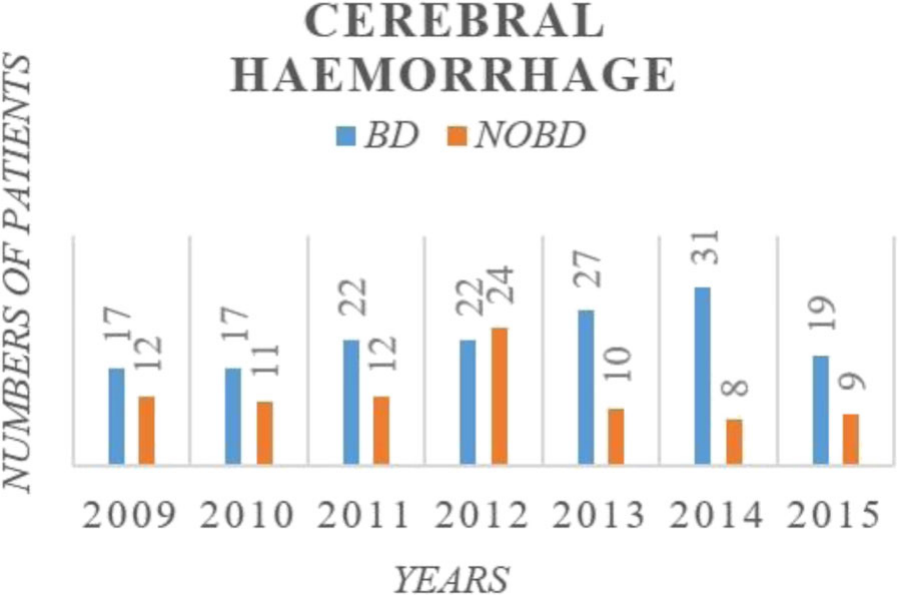
Brain death in cerebral hemorrhage

**Fig. 87 (Abstract P329).**
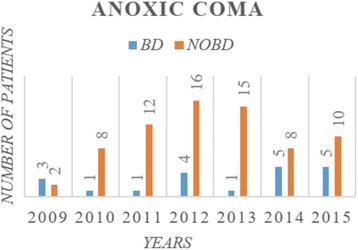
Brain death in anoxic coma

465 dead patients with ABI were included in the study. Age, sex, admission GCS and diagnosis, cause of death were recorded. The mean age was 68.8 ± 14.9 standard deviation (SD) with a progressive increase during the period observed from 60.3 ± 14.4 (2009) to 69.1 ± 14.4 (2015) in the BD group (p < 0.05). Males were 236 (50.8 %). The mean GCS at admission was 4.4 ± 2.5. Among all patients, 245(52.7 %) have met criteria for brain death according with Italian law. Admission diagnoses were cerebral haemorrhage for 241 patients (51.8 %), anoxic coma for 91(19.6 %), brain trauma for 71(15.3 %), ischemic stroke for 46 (9.9 %), brain cancers for 9 (1.9 %) and other reasons for 7 (1.5 %). 155 (63,3 %) patients with brain haemorrhage developed brain death (p < 0.01) (Fig. 86), while only 20 (21,9 %) anoxic brain injury patients experienced brain death (p = 0.052) (Fig. 87).

**Conclusions**

The incidence of brain death was higher for patients admitted in the NICU with cerebral haemorrhage than for patients with anoxic brain injury.

## P330 Brain magnetic resonance imaging findings in patients with septic shock

### G. Orhun^1^, E. Senturk^1^, P. E. Ozcan^1^, S. Sencer^2^, C. Ulusoy^3^, E. Tuzun^3^, F. Esen^1^

#### ^1^Istanbul University, Medical Faculty of Istanbul, Anesthesiology and Intensive Care, Istanbul, Turkey; ^2^Istanbul University, Medical Faculty of Istanbul, Department of Neuroradiology, Istanbul, Turkey; ^3^Istanbul University, Institute of Experimental Medicine, Neuroscience, Istanbul, Turkey

**Introduction**

The imaging of the brain of septic shock patients showing alterations in their neurological status are unremarkable most of the time. The current study provided findings from magnetic resonance imaging of the brain in septic shock.

**Methods**

Twenty patients with septic shock and brain dysfunction symptoms (acute alterations in mental status, delirium, coma, seizures and focal neurological deficits) [median age 54 years (26 to 65), APACHE II: 21 (15 to 29), SOFA: 8(1 to 15)] underwent brain magnetic resonance imaging (MRI) including gradient echo T1-weighted, fluid-attenuated inversion recovery (FLAIR), T2-weighted and diffusion isotropic images, and mapping of apparent diffusion coefficient. MRI findings were classified based on lesion types and localizations. Blood was withdrawn simultaneously for biomarker analysis. Neurological recovery of all patients were evaluated using Glasgow Outcome Scale (GOS) during discharge.

**Results**

None of the patients’ brain imaging was normal at the time of the diagnosis of neurological alterations including mostly delirium. Twelve patients showed white matter hyperintensity (leukoencephalopathy), three patients showed ischemic lesions and posterior reversible encephalopathy syndrome findings were evident in three patients. Unexpected findings of cerebral atrophy not relating to age were seen in seven patients. The lesions were correlated with disease severity and GOS. No unexpected events were encountered during transport and MRI scan.

**Conclusions**

Our study indicates the importance of brain imaging in severe sepsis and septic shock. MRI can be an important imaging method in these patients, where lesions were associated with disease severity and poor outcome.

## P331 Benefits of L-carnitine in valproic acid induced encephalopathy

### R. Tincu^1^, C. Cobilinschi^1^, D. Tomescu^2^, Z. Ghiorghiu^1^, R. Macovei^1^

#### ^1^Bucharest Clinical Emergency Hospital, Bucharest, Romania; ^2^Fundeni Clinical Institute, Bucharest, Romania

**Introduction**

Valproic acid (VPA) is widely used as antiepileptic drug, which is known to induce hepatotoxicity and hyperammonemia encephalopathy. These toxic side effects are produced by a decrease in the carnitine blood-levels, of which the valproic acid is directly responsible. The aim of this report is to demonstrate the benefits of L-carnitine supplementation in patients with acute valproic acid intoxication. Patients who are chronically treated with VPA show low levels of carnitine by depletion of its deposits, therefore L-carnitine addition proves to be appropriate.

**Methods**

This was a randomized controlled trial conducted in the Intensive Care-Toxicology Unit of the Clinical Emergency Hospital in Bucharest, that included all patients admitted for acute VPA poisoning (VPA >150 μg/l), in 2014. The patients were randomly allocated in 2 groups to receive standard therapy or 1800 mg of L-carnitine/ day together with standard therapy for 3 days. Plasma levels of valproic acid and ammonemia were determined every six hours. Data was statistically analyzed and the results were considered statistically significant at p < 0.05.

**Results**

A total of 62 patients (34 in the standard group and 28 in the L-carnitine group) accomplished the inclusion criteria. L-Carnitine supplementation resulted in significant reductions in ammonemia (47.9 ± 6 vs. 61.9 ± 11.39 μ mol/L), determined after 24 hours, levels compared with baseline (P < 0.001), whereas this parameter remained still high in standard group. The trend was similar for plasma VPA levels (p < 0.05). Studied parameters were not influenced by gender, but were correlated with age (p = 0.01).

**Conclusions**

The use of L-carnitine in the treatment of VPA poisoning accelerates the elimination of VPA and facilitates the decrease in ammoniac plasma levels, therefore reducing systemic toxicity and encephalopathy risk. Further controlled, extented trials are required to better elucidate the therapeutic and prophylactic roles of L-carnitine in the management of VPA toxicity.

## P332 Automatic analysis of EEG reactivity in comatose patients

### M. Van Assen^1^, M. M. Admiraal^1^, M. J. Van Putten^2^, M. Tjepkema-Cloostermans^2^, A. F. Van Rootselaar^1^, J. Horn^1^

#### ^1^Academic Medical Center, Amsterdam, Netherlands; ^2^Medisch Spectrum Twente, Enschede, Netherlands

**Introduction**

EEG reactivity has been reported as a predictor of outcome in comatose patients with post-anoxic encephalopathy [1,2]. However, visual analysis of EEG reactivity has a high inter-rater variability and is time consuming [3,4]. Therefore, analysis of EEG reactivity may benefit from a quantitative approach enabling automatic interpretation.

**Methods**

Prospective cohort study in comatose ICU patients with continuous EEG background patterns in two Dutch ICUs. Reactivity to auditory, visual and sensory stimuli was tested following a strict protocol. Visual analysis was regarded as the gold standard. Reactivity was defined as a change in amplitude or frequency in the EEG after stimulation, excluding muscle activity. Stimulus responses were scored by three independent experts (JH, MvP, MT-C), responses on which all three experts agreed were used. Data was separated in an independent training and validation set. Spectral characteristics of the EEG before and after each stimulus were compared. These were determined by both parametric and non-parametric methods. Epochs of 2, 5 and 10 seconds before and after the start of the stimulus were analyzed and averaged over 1, 3, 5 and 7 channel.

**Results**

In the training set and validation set, 190 and 163 stimuli of 13 and 7 patients, respectively, were included. The prevalence of a reactive response is 8.3 % in the training set and 3.1 % in the validation set. The non-parametric power spectral density with a difference of 30 %, in 5 second epochs, averaged over 5 channels was found to be the optimal method for automatic analysis. This resulted in a sensitivity of 92.9 % (95%CI 87.6-96.2) and a specificity of 94.8 % (95%CI 90.0-97.7) in the training set. In the validation set a sensitivity of 40.0 % (95%CI 32.5-48.0) and a specificity of 78.5 % (95%CI 71.2-84.4) were found.

**Conclusions**

Automatic analysis of EEG reactivity based on the differences in spectral characteristics detects reactivity. With further research we will optimize this method to increase objectivity of EEG reactivity testing.

**References**

1. Rossetti, A. O. et al. Prognostication after cardiac arrest and hypothermia: a prospective study. Annals of Neurology. 2010; 67(3):301-307.

2. Rossetti, AO, et al. Prognostic value of continuous EEG monitoring during therapeutic hypothermia after cardiac arrest. Critical Care. 2010; 14(5): R173.

3. Westhall, E, et al. Interrater variability of EEG interpretation in comatose cardiac arrest patients. Clinical Neurophysiology. 2015; 126(12): 2397-2404.

4. Hermans, MC, et al.(in press) Clinical Neurophysiology, 2015

## P333 Usefulness of common ICU severity scoring systems in predicting outcome after spontaneous intracerebral hemorrhage

### M. Fallenius^1^, M. B. Skrifvars^1^, M. Reinikainen^2^, S. Bendel^3^, R. Raj^1^

#### ^1^Helsinki University Hospital, Helsinki, Finland; ^2^North Karelia Central Hospital, Joensuu, Finland; ^3^Kuopio University Hospital, Kuopio, Finland

**Introduction**

Accurate prognostic models, including common intensive care severity scores such as APACHE II (acute physiology and chronic health evaluation II), SAPS II (simplified acute physiology score) and SOFA (sequential organ failure assessment) are of high importance for quality assurance and research in intensive care [1-3]. The aim of our study was to investigate the usefulness of these in predicting mid-term mortality after spontaneous intracerebral hemorrhage (ICH), and whether the scores are of any extra value compared to a basic model comprising of only age and level of consciousness.

**Methods**

We included adult patients with spontaneous ICH, treated in Finnish intensive care units (ICU) in 2003-2012 from a nationwide ICU database. Logistic regression was used to customize APACHE II, SAPS II and SOFA for six-month mortality prediction. We created a reference model based on age and Glasgow Coma Scale (GCS) score for comparison. We used a temporal split-sample technique for internal validation. We tested model performance by assessing discrimination (area under the curve [AUC]) and calibration (Hosmer-Lemeshow Ĉ and GiViTI calibration tests).

**Results**

Totally 3,218 patients were included (1,238 in the development and 1,980 in the validation cohort). Both APACHE II and SAPS II showed good discrimination (AUC 0.84, AUC 0.85, respectively) but did not outperform the reference model (AUC 0.85; compared to APACHE II, p = 0.133; compared to SAPS II, p = 0.141). SOFA, however, displayed significantly poorer performance compared to the other models (AUC of 0.76). All models showed poor calibration (p < 0.05). Age was found to be a particularly strong predictor of death in patients with GCS 9-12, as the mortality for patients aged <40 was only 8 %, yet being as high as 43 % for patients aged >79.

**Conclusions**

Both APACHE II and SAPS II-based models showed good discrimination, but poor calibration for predicting six-month mortality in patients with spontaneous ICH treated in the ICU. The performance of a simple prognostic model composed of only age and GCS, was comparable to the more complex scoring systems and based on our results, could replace these in this patient group.

**References**

1. Knaus WA et al. Chest 100:1619–1636, 1991

2. Le Gall JR et al. JAMA 270:2957-2963, 1993

3. Vincent JL et al. Intensive Care Medicine 22:707-710, 1996

## P334 Evalution of patients with suspected subarachnoid haemorrhage and negative ct imaging

### M. Abu-Habsa, C. Hymers, A. Borowska, H. Sivadhas, S. Sahiba, S. Perkins

#### King’s College Hospital, London, UK

**Introduction**

The commonest symptom of non-traumatic subarachnoid haemorrhage (SAH) is sudden onset severe headache. The Emergency Physician is faced with a significant diagnostic challenge by this condition as headache represents 1-2 % of ED attendances while SAH only accounts for 1-3 % of these headaches [1,2]. Conventional practice remains lumbar puncture (LP) sample analysis in patients with a negative CT scan [1]. Recent series in Europe and the United States have demonstrated wide variation in practice as whether an LP is undertaken with no clear guidance on LP omission [2]. We propose a clinical decision tool integrating recent evidence on this patient group.

**Methods**

Our search strategy included Medline, Embase, Google Scholar, international guidelines and reference searches as well as the authors’ collection on the subject. The following search strings were applied: [Subarachnoid haemorrhage exp OR subarachnoid hemorrhage exp OR SAH.mp OR berry aneurysm.mp OR perimesencephalic OR arteriovenous malformation.mp] AND [Lumbar puncture.mp OR cerebrospinal fluid exp OR spinal tap.mp OR spinal OR LP.mp OR CSF.mp] AND [Computed Tomography exp OR CT.mp]. Studies using < 4 detector technology CT-scanners were excluded.

**Results**

Multiple risk factors have been identified for SAH including: Loss of consciousness, family history, hypertension, polycystic kidneys, excessive alcohol intake and smoking. The aetiology of SAH in the majority (>85 %) of patients is an aneurysm in the “Circle of Willis” [2]. CT sensitivity within eligible studies ranged from 91-100 % [2], the largest study was of a prospective design and included 3132 patients and reported 100 % sensitivity if CT is performed within 6 hours of symptom onset [1]. Recent work in the UK including 2248 patients who underwent an LP concluded that an LP to be of low diagnostic yield with the investigation of choice in patients equivocal LP findings or recurrent symptoms was CT Angiography [2].

**Conclusions**

The majority of patients presenting with acute severe headache can have a SAH excluded with a negative CT scan if seen with 6 hours of onset and the images are reported by a Radiologist. A number of patients with delayed presentation or risk factors for SAH benefit from CT Angiography unless contra-indicated. Our findings will be presented as an evidence based clinical decision tool.

**References**

1. Perry, J.J., I.G. Stiell, M.L. Sivilotti, et al., Sensitivity of computed tomography performed within six hours of onset of headache for diagnosis of subarachnoid haemorrhage: prospective cohort study. BMJ, 2011. 343: p. d4277.

2. Refs 2-40.

## P335 Timing of endovascular and surgical treatment for aneurysmal subarachnoid haemorrhage: early but not so fast.

### J. Rubio^1^, J. A. Rubio^2^, R. Sierra^1^

#### ^1^Hospital Universitario Puerta del Mar, Cadiz, Spain; ^2^Hospital Infanta Cristina, Badajoz, Spain

**Introduction**

Aneurismal subarachnoid haemorrhage (aSAH) is a significant cause of morbidity and mortality throughout the world. The primary goal of the treatment is the occlusion of the ruptured aneurysm preventing a rebleeding. To reduce the rate of this adverse event current guidelines recommend that surgical clipping or endovascular coiling should be performed as early as feasible. However, this strategy may be associated with several disadvantages and timing of procedure remains controversial. The aim of this study was to evaluate the effect of timing of invasive treatment for aSAH on clinical outcomes.

**Methods**

We performed a prospective, observational cohort study at a tertiary university 750-bed hospital including patients with severe aSAH admitted to the Intensive (ICU) or Intermediate Post-operative Care Unit (IMCU) after trying early endovascular coiling or surgical clipping over a 1-year period (November 2014 to October 2015). We analysed demographic, comorbidities, APACHE II score, Fisher and Hunt-Hess grade, aneurysm characteristic, type of treatment, time interval between ICU or IMCU admission and invasive procedure, ICU, IMCU and hospital length of stay, and outcomes variables. The study outcomes were mortality, readmissions and re-operations.

**Results**

A total of 31 patients were included aged 56.5 ± 9.8 years old, with 22 (71 %) females and APACHE II score on admission 15.9 ± 8.4. Endovascular coiling was performed in 27 (87 %) and surgical clipping 4 (13 %) patients. Twenty-three (74.2 %) patients were admitted to the ICU and eight (25.8 %) to the IMCU. Aneurysms were mainly on anterior location (71 %), with saccular morphology (64.5 %) and medium size (7.8 ± 3.4 mm). Fisher and Hunt-Hess grade 4 occurred most frequently. A total of 7 (22.6 %) patients (5 coiling, 2 clipping) died during ICU stay. The mean time from ICU admission to procedure performed was shorter in this group than in the survivor group (0.8 days [95 % CI 0.2 to 1.5]) versus 2.2 days (95 % [CI 1.4 to 2.9]); P = 0.03). There was no correlation between time from admission to procedure and ICU, IMCU or hospital length of stay. Only one patient was readmitted to the ICU and in three was necessary repeat a new invasive procedure.

**Conclusions**

In our cohort of aSAH patients treated with endovascular and surgical methods and requiring intensive care we found a high mortality rate mainly related to early procedure. Furthermore, we speculate that these findings could have a significant impact on organizational strategies and cost saving of healthcare organizations in some countries.

## P336 Red blood cell transfusion in aneurysmal subarachnoid hemorrhage – the Sahara cohort study

### S. English^1^, M. Chasse^2^, A. Turgeon^2^, F. Lauzier^2^, D. Griesdale^3^, A. Garland^4^, D. Fergusson^5^, R. Zarychanski^3^, A. Tinmouth^5^, C. Van Walraven^5^, K. Montroy^5^, J. Ziegler^4^, R. Dupont Chouinard^2^, R. Carignan^2^, A. Dhaliwal^3^, C. Lum^5^, J. Sinclair^5^, G. Pagliarello^5^, L. McIntyre^5^

#### ^1^The Ottawa Hospital, Ottawa, Canada; ^2^Ulaval, Quebec City, Canada; ^3^UBC, Vancouver, Canada; ^4^UManitoba, Winnipeg, Canada; ^5^OHRI, Ottawa, Canada

**Introduction**

Restrictive red blood cell transfusion (RBCTx) strategies are advocated in most critical care populations. Whether this is standard practice and applies to an aneurysmal subarachnoid hemorrhage (aSAH) patient population is unclear. We aimed to describe transfusion practices in an aSAH population in Canadian hospitals, including hemoglobin (Hb) triggers and predictors of RBCTx in preparation for a transfusion trial.

**Methods**

This is a multi-centre retrospective cohort study conducted at four Canadian academic tertiary care centres. Population: All adult aSAH patients admitted to the study hospitals from January 1, 2012 to December 31, 2013. Patients were identified from hospital administrative discharge records and existing local SAH databases. The diagnosis was confirmed by reported presence of blood in the subarachnoid space (on either imaging or lumbar puncture) due to a ruptured cerebral aneurysm (as demonstrated on angiography, neuro-imaging or autopsy). Data Collection: Trained abstractors collected demographic data, aSAH characteristics, administration of RBC transfusion, daily hemoglobin concentrations, other major aSAH co-interventions, and outcomes using a pre-tested case report form in reference to a standardized operations manual.

**Results**

From 886 screened patients, a total of 527 (59.5 %) with aSAH were included in our study. Mean (±SD) age was 57 ± 13 years; 357 (67.7 %) were female. Median presenting Fisher Grade was 4 (IQR 3-4). Aneurysms were secured in 90.1 % of patients (52.2 % by endovascular coiling, and 39.7 % by surgical clipping). Mean nadir hemoglobin was 98 ± 20 g/L and occurred on median post-admission day (PAD) 4 (IQR 2-11). RBCTx occurred in 100 patients (19.0 %). RBCTx rates varied across centres (12.1-27.4 %, p = 0.02). A median 1 unit of RBCs (IQR 1-2) was administered per transfusion episode and patients received a median total of 2 units (IQR 1-4). Median pre-transfusion hemoglobin for first transfusion was 79 g/L (IQR 74-93) and did not vary substantially across centres (78-82 g/L, p = 0.37). Of patients with nadir Hb < 80 g/L, 65.8 % received an RBCTx compared to 7.0 % with Hb nadir > =100 g/L (p < 0.0001). Univariate predictors of RBCTx were increasing age, anterior circulation aneurysm, lower presenting GCS, advanced Fisher Grade, neurosurgical clipping and lower Hb.

**Conclusions**

In our retrospective study of aSAH patients, we observed that RBCTx was uncommon (19.2 % of patients), and mostly reserved for patients with significant anemia (Hb <80 g/L).

## P337 Aneurysmal subarachnoid hemorrhage and anemia: a canadian multi-centre retrospective cohort study

### S. English^1^, M. Chasse^2^, A. Turgeon^2^, F. Lauzier^2^, D. Griesdale^3^, A. Garland^4^, D. Fergusson^5^, R. Zarychanski^4^, A. Tinmouth^5^, C. Van Walraven^5^, K. Montroy^5^, J. Ziegler^4^, R. Dupont Chouinard^2^, R. Carignan^2^, A. Dhaliwal^3^, C. Lum^5^, J. Sinclair^5^, G. Pagliarello^5^, L. McIntyre^5^

#### ^1^The Ottawa Hospital, Ottawa, Canada; ^2^Ulaval, Quebec City, Canada; ^3^UBC, Vancouver, Canada; ^4^UManitoba, Winnipeg, Canada; ^5^Ottawa Hospital Research Institute, Ottawa, Canada

**Introduction**

Little is published about anemia in aneurysmal subarachnoid hemorrhage (aSAH). We aimed to describe the disease burden of an aSAH population in Canadian hospitals, and the prevalence and incidence of moderate anemia (hemoglobin <100 g/L) in preparation for a transfusion trial.

**Methods**

This is a multi-centre retrospective cohort study conducted at four Canadian academic tertiary care centres. Population: All adult aSAH patients admitted to the study hospitals from January 1, 2012 to December 31, 2013. Patients were identified from hospital administrative discharge records and existing local SAH databases. The diagnosis was confirmed by presence of blood in the subarachnoid space (on either imaging or lumbar puncture) due to a ruptured cerebral aneurysm (as demonstrated on any of angiography, neuro-imaging or autopsy). Data Collection: Trained abstractors collected demographic data, aSAH characteristics, daily hemoglobin concentrations and nadir hemoglobin, other major aSAH co-interventions, and outcomes using a pre-tested case report form in reference to a standardized operations manual.

**Results**

A total of 527 patients (59.5 %) with aSAH met study eligibility criteria. Mean (±SD) age was 57 ± 13 years; 357 (67.7 %) were female. The most common prior medical history was presence of hypertension (44.2 %) and active tobacco smokers (35.1 %). Mean aneurysm size was 7 ± 4 mm with 62.0 % occurring in the anterior circulation. Median presenting Glasgow Coma Scale score and Fisher Grade was 14 (IQR 11-15) and 4 (IQR 3-4) respectively. Aneurysms were secured in 90.1 % of patients (52.2 % by endovascular coiling, and 39.7 % by surgical clipping). Moderate anemia was prevalent in 29 patients (5.5 %) at admission and occurred in an additional 259 (52.0 %) during hospitalization. Nadir hemoglobin occurred on median post-admission day (PAD) 4 (IQR 2-11). Mean admission and nadir hemoglobin were 130 ± 17 g/L and 98 ± 20 g/L, respectively. Within the first 21 days of admission, moderate anemia occurred in 273 (51.8 %) of patients by median PAD 2 (1-4). RBC transfusion occurred in 100 (19.0 %) of patients. In-hospital mortality was 17.7 %; 35.3 % of the survivors were discharged from hospital with moderate to severe disability.

**Conclusions**

Moderate anemia (hemoglobin <100 g/L) is common in patients admitted with aneurysmal subarachnoid hemorrhage. Less than 20 % of aSAH patients receive a RBC transfusion. Acute aSAH is associated with significant mortality, and morbidity in survivors.

## P338 Does the neutrophil-to-lymphocyte (NLR) ratio predict symptomatic vasospasm or delayed cerebral ischemia (DCI) after aneurysmal subarachnoid haemorrhage (SAH)?

### T. Groza, N. Moreau, D. Castanares-Zapatero, P. Hantson

#### Cliniques St Luc, Université catholique de Louvain, Brussels, Belgium

**Introduction**

NLR has been investigated as a biomarker of poor outcome in various conditions. The prognostic value of NLR and of C-reactive protein (CRP) was investigated in patients with SAH. Our hypothesis was that the profile of NLR from admission to the 5th hospital day could predict the development of symptomatic vasospasm or DCI.

**Methods**

We retrospectively reviewed data from consecutive SAH patients (Jan 1, 2004 - Jan 1, 2015) and recorded demographics, neurological examination, radiological and transcranial Doppler findings, length of stay (LOS) in the ICU, and mortality. NLR values were calculated from admission to the 5th hospital day. Medians were compared between groups using Mann Whitney U test. Multivariate logistic regression analysis using stepwise backward method was generated to predict the occurrence of symptomatic vasospasm and DCI. Receiver operating characteristic (ROC) curves were generated to assess the accuracy of NLR to predict complications.

**Results**

The cohort included 123 patients (71 % F, median age 53). Severity was high, with 40.7 and 50.4 % of the patients having respectively a Fisher grade 3 or 4 SAH. The mortality rate was 23.6 %. The median ICU LOS was 12 days (IQR:15-40). Asymptomatic vasospasm was noticed in 9.8 % of the patients, and symptomatic vasospasm in 36 %. DCI was demonstrated in 62 %. Early infection (within the first five days) was noticed in 15.4 %. The median NLR at ICU admission was 9.2 (IQR:5-12.7) for the entire cohort and NLR decreased over time with a median NLR at 6.4 on day 5 (IQR:4.6-9.6). The median NLR was different in patients with or without symptomatic vasospasm (7.8 vs 6.7,p = 0.045), but only on day 3. CRP values were different on day 1 and 4 in patients with early infection but showed no difference between patients with or without DCI. Univariate analysis found that younger age and Fisher grade were associated with DCI. Neither NLR nor CRP could predict the onset of DCI. Lower NLR at day 5 was associated with symptomatic vasospasm (OR:0.89[95%CI:0.79-0.98]) in univariate analysis. Following multivariate analysis, a younger age (OR:0.96[95%CI:0.93-0.99]) and a lower NLR at day 5 (OR:0.89[95%CI: 0.80-0.99]) were identified as risk factors for symptomatic vasospasm.

**Conclusions**

NLR from day 0 to 5 did not vary with the presence of vasospasm or DCI. NLR at day 3 was slightly lower in patients with vasospasm. A lower NLR at day 5 was associated with an increased risk of symptomatic cerebral vasospasm.

## P339 ICU-acquired infections in aneurysmal subarachnoid hemorrhage patients: impact on ICU and hospital length of stay

### M. Carbonara ^1^, F. Ortolano^1^, T. Zoerle^1^, S. Magnoni^1^, S. Pifferi^1^, V. Conte^1^, N. Stocchetti^2^

#### ^1^Fondazione IRCCS Ca’ Granda Ospedale Maggiore Policlinico, Milan, Italy; ^2^Milan University, Milan, Italy

**Introduction**

Patients with aneurismal subarachnoid hemorrhage (aSAH) have increased risk to develop nosocomial infections, associated with poor outcome.

Aim of our study was to describe infections incidence and features in aSAH patients and evaluate the impact on ICU and hospital length of stay (LOS).

**Methods**

We retrospectively analyzed data from a observational trial on aSAH. All patients with aSAH admitted to our ICU were included. Demographic, clinical, radiological and microbiological data were recorded. Infections were diagnosed according to current guidelines. Patients who died within 72 hours from admission were excluded from analysis. Statistical analysis was performed using Prism. Significance was set at a p value < = 0.05.

**Results**

159 patients were included: median age was 59 years old, 30 % was male. Aneurysm was located in anterior circulation in 139 patients and secured through coiling in 76 %.

74 patients (47 %) experienced 98 septic episodes: incidence was low (31 %) in less severe (World Federation of Neurosurgeons grade (WFNS) 1 to 3) and higher in more severe aSAH (WFNS 4-5 78 %, p < 0.001). Diagnosis was made at 4 ± 1 days from admission. VAP (79 %) and urinary tract infections (10 %) were the most prevalent infection. 1 patients had septic shock. Most common pathogens were Haemophilus influenzae, Escherichia coli and Pseudomonas aeruginosa, accounting for 37 % of infections, while multidrug resistant bacteria were less than 5 %.

**Fig. 88 (Abstract P339).**
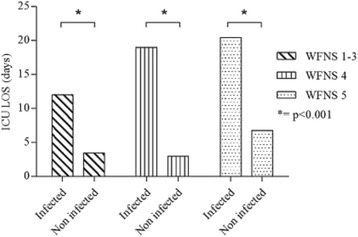
ᅟ

ICU and hospital LOS were significantly higher in septic compared to non-septic patients, independently of WFNS category, as shown in Fig. 88.

**Conclusions**

ICU-acquired infections are common especially in severe aSAH patients, predominantly VAP caused by Gram negative bacteria. Infections cause longer ICU and hospital LOS.

## P340 Cerebral metabolic effects of normobaric hyperoxia during the acute phase of aneurysmal subarachnoid hemorrhage

### L. Carteron^1^, T. Suys^1^, C. Patet^1^, H. Quintard^2^, M. Oddo^1^

#### ^1^CHUV, Lausanne, Switzerland; ^2^CHU de Nice, Nice, France

**Introduction**

Mechanical ventilation is often necessary in patients with poor-grade subarachnoid hemorrhage (SAH) and supra-normal oxygen levels are frequently achieved in the acute phase both in prehospital setting or in the intensive care unit. However the exact cerebral physiologic effects of normobaric hyperoxia have not been investigated in this setting in humans. Cerebral microdialysis (CMD) is a unique technique that allows the chemistry of the extracellular brain interstitial fluid to be monitored continuously at patient bedside.

**Methods**

Retrospective analysis of a prospective database of comatose patients after SAH monitored with CMD as part of standard care. The relationship of hyperoxia (defined as a supra-normal PaO2 > 150 mmHg for at least 1 hour) with brain extracellular biomarkers (including CMD lactate, lactate/pyruvate ratio [LPR] and glutamate) was examined during the acute phase (first 48 hours) following SAH. Comparisons between groups were explored using non-parametric Mann-Whitney U test. Significance was set at p < 0.05. Approval for the study was obtained from our local Ethical Committee.

**Results**

A total of 460 matched PaO2/CMD samples from 28 consecutive mechanically ventilated poor-grade SAH patients (median age 58 [10th-90th percentiles 42-68] years, median WFNS 5 [3-5]) were analyzed. Compared to non-hyperoxic samples (n = 366), those with hyperoxia (n = 94) were associated with higher CMD levels of lactate (3.5 [2-7.5] vs. 4.7 [2.3-9.1] mmol/L), LPR (30 [19-54] vs. 36 [23-53]) and glutamate (12.1 [2.2-61.2] vs. 27.2 [1.9-68.6] μmol/L) (all p < 0.01), while main cerebral (PbtO2, ICP, CPP) and systemic (PaCO2, MAP) variables were within normal ranges and did not differ between the two conditions.

**Conclusions**

Our findings in comatose patients with SAH monitored with intra-cerebral microdialysis suggest that hyperoxia was associated with increased secondary cerebral damage. Our study indicates that the use of supra-normal oxygen levels (PaO2 > 150 mmHg) may be harmful in the acute ICU phase following SAH.

**Fig. 89 (Abstract P340).**
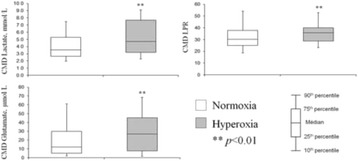
Box-plots illustrating CMD concentrations in normoxic and hyperoxic conditions

## P341 Postoperative care for elective craniotomy: where is best done?

### J. A. Rubio^1^, J. Rubio^2^, R. Sierra^2^

#### ^1^Hospital Infanta Cristina, Badajoz, Spain; ^2^Hospital Universitario Puerta del Mar, Cadiz, Spain

**Introduction**

Admission to intensive care unit (ICU) is a routine practice following elective craniotomy for brain surgery. The rationale for this practice is the high risk of surgery on this vital organ and that ICU allows the early detection of serious complications. However, several studies have shown that many of these patients require minimal ICU interventions [1]. In addition, ICU resources are scarce and costly. Intermediate care units provide more monitoring than general wards and less than ICU with evidence to support the benefits on cost and patient outcomes [2]. The aim of this study was to compare outcomes of patients underwent elective craniotomy who were admitted to the intensive or intermediate post-operative care unit (IMCU) settings postoperatively.

**Methods**

We conducted a prospective observational study during a year period (November 2014 to October 2015) with patients who had undergone elective craniotomy for brain surgery admitted postoperatively to ICU or IMCU of a tertiary 750-bed hospital. Main variables analyzed included demographic, comorbidities, ASA, APACHE II score, duration of anesthesia and surgery, surgical procedure and diagnosis, ICU and IMCU length of stay. Study outcomes were mortality, readmissions to ICU and re-operation rates.

**Results**

One hundred and fourteen patients were included in the study (mean age 53.8 ± 14.2 years, 61 (63.5 %) males). Sixty-five (57 %) patients were ASA II and 29 (24.4 %) class III. Mean APACHE II score was 10.1 ± 8.3. Duration of anesthesia and surgery were 241 ± 99.6 min and 208 ± 87.6 min, respectively. Eighty-two patients were admitted to ICU and 32 to IMCU. Main diagnoses were supratentorial meningioma (n = 27) and glioma (n = 20), infratentorial tumor (n = 21) and transsphenoidal resection of pituitary tumours (n = 21). Duration of stay was 24.3 (95 % CI [7 to 24.9]) hours in ICU and 16.2 (95 CI [13.8 to 18.5]) hours in IMCU group, respectively (P < 0.001). Two patients admitted to the ICU died. Four patients from the ICU and two from the IMCU group were readmitted. Five patients in ICU and two in IMCU patients were re-operated.

**Conclusions**

In our setting, patients undergoing elective craniotomy for brain surgery received proper postoperative care in both units. Length of stay was shorter in the IMCU than in the ICU group. Mortality, readmissions and re-operations were similar. Cost could be reduced when postoperative care for this surgery is provided in intermediate care unit.

**References**

1. Bui JQ et al.: J Neurosurg 2011; 115: 1236-1241.

2. Vincent JL et al.: Crit Care 2015; 19 (1):89.

## P342 5-year follow-up of patients after transplantation of organs from donors from neurocritical care

### V. Spatenkova^1^, E. Pokorna^2^, P. Suchomel^1^

#### ^1^Regional Hospital, Liberec, Czech Republic; ^2^Institute of Experimental Medicine, Prague, Czech Republic

**Introduction**

Every realized donor counts, because they can all save or improve somebody’s life. The aim of this study was to analyse patients after receiving organs from donors from our neurocritical care.

**Methods**

We performed a 5-year prospective follow-up study on patients from the Transplant Center after transplantation of organs donated by 14 stroke patients with a diagnosis of brain death in our adult neurocritical care unit (subarachnoid haemorrhage 7, intracerebral haemorrhage 4, ischemic stroke 3). Overall 29 organs (27 kidneys, 2 livers) were retrieved and transplanted to 29 patients with mean age 55.3 ± 9.76 years.

**Results**

Of the 27 patients who underwent a kidney transplant, 21 (78 %) patients lived 5 years, of those 17 (63 %) patients with functional graft. One patient (4 %) had primary afunctional graft and in three patients (11 %) rejection occurred (3, 15 and 41 months). Six patients (22 %) died after their kidney transplants, 1 patient with functional graft, 1 patient had primary afunctional graft and 4 (15 %) patients had rejection (1, 12, 44 and 56 months). The two patients with liver transplants lived 5 years with functional graft.

**Conclusions**

14 neurocritical care organ donors helped patients live 5 years with functional graft,17 patients after kidney transplants and 2 patients after liver transplants.

## P343 Evaluation of levetiracetam pharmacokinetics after severe traumatic brain injury in neurocritical care patients at a level one trauma center

### N. Ebert, J. Jancik, H. Rhodes

#### Hennepin County Medical Center, Minneapolis, USA

**Introduction**

Traumatic brain injury (TBI) may affect the pharmacokinetics of anti-epileptic drugs (AEDs) leading to the potential for ineffective post-traumatic seizure (PTS) prophylaxis. Levetiracetam (LEV) is increasingly utilized for the prophylaxis of PTS. Recent studies suggest that neurocritical care patients may require higher LEV doses to achieve therapeutic trough levels of 12-20mcg/mL [1,2]. The aim of this study is to describe the pharmacokinetics of LEV in the early (less than or equal to 7 days after injury) post-TBI period and evaluate the incidence of seizures.

**Methods**

This retrospective chart review included adult patients with a diagnosis of severe TBI who had at least one LEV level drawn during an intensive care unit (ICU) admission from January 2010 to October 2015. Patients were evaluated for the following outcomes: LEV trough levels (drawn at steady state), LEV dosing regimen, % of patients reaching LEV trough goal, LEV duration of therapy, diagnosis of seizure during hospital admission, hospital and ICU length of stay (LOS), and in-hospital mortality.

**Results**

Evaluation of 26 patients resulted in a median LEV trough of 9.7mcg/mL (IQR 6.75-18.8) with only 19.2 % of patients within trough goal (57.6 % below goal, 23.1 % above goal). A subset of 15 patients had two consecutive LEV levels, which allowed for pharmacokinetic evaluation, resulting in median maximum concentration (Cmax) = 32.2mcg/mL, minimum concentration (Cmin) = 8.1mcg/mL, volume of distribution = 28.5 L, and half-life = 7.4 hours. The most common maintenance dose was 1 g twice daily (57.6 % of patients), followed by 1.5 g twice daily (23.1 %). Median duration of LEV therapy was 7 days. Seizures occurred in 7 patients during admission (26.9 %), with the majority of seizures occurring in the first 24 hours of admission. Median ICU and hospital LOS were 11 and 15 days, respectively, with an in-hospital mortality rate of 11.5 %.

**Conclusions**

The majority of LEV trough levels were subtherapeutic during the early post-TBI period despite more aggressive initial dosing compared to previous studies. Linear regression suggests that the optimal dose of LEV for patients with normal renal function is 1.5 g every 8 hours. Further research is necessary to determine the impact of augmented renal clearance on LEV levels in patients with severe TBI.

**References**

1. Dewolfe JL, et al. Frontiers in Neurology 4:1-8, 2013

2. Spencer DD et al. Pharmacotherapy 31:934-941, 2011

## P344 Model based time series cluster analysis to determine unique patient states in traumatic brain injury

### T. Bylinski^1^, C. Hawthorne^2^, M. Shaw^3^, I. Piper^3^, J. Kinsella^4^

#### ^1^University of Glasgow, Glasgow, UK; ^2^Institute of Neurological Sciences, NHSGGC, Glasgow, UK; ^3^Department of Clinical Physics and Bioengineering, NHSGGC, Glasgow, UK; ^4^Academic Unit of Anaesthesia, Pain and Critical Care Medicine, University of Glasgow, Glasgow, UK

**Introduction**

The BrainIT database contains the physiological and outcome data of 261 TBI patients [1]. We aimed to identify distinct patient groups distinguishable by the trajectory of their physiology over time using cluster analysis, a form of data mining [2]. We hypothesised that the resulting groups would have a good or poor outcome, measured by the extended Glasgow outcome score (eGOS). This would indicate physiological trajectories associated with a good or poor prognosis.

**Methods**

The MREC for Scotland approved the use of BrainIT data for scientific purposes in 2002, and need for informed consent was waived. We first cleaned the database, which left 155 patients. These patients were then clustered based on 24-hour trajectories of CPP, ICP, HR, SaO2, and mean BP. To do this, we applied the general linear mixed model, a form of cluster analysis [2], to the data to create three algorithms: Algorithm 1 clustered patients on minute-by-minute physiological data, Algorithm 2 used the same data with six outlying patients removed, and Algorithm 3 used hour-by-hour data, with the same six outlying patients removed as in Algorithm 2. Each algorithm’s resulting clusters were paired to admissions data such as first GCS and outcome data as measured by eGOS.

**Results**

Algorithm 1 identified six outliers with distinct and extreme physiological trajectories and eGOS. (n = 149, eGOS 5; n = 3, eGOS 7; n = 2, eGOS 1; n = 1, eGOS 1). The patients in Algorithm 2 did not separate into clusters (n = 149, eGOS = 5). Algorithm 3 revealed three clusters (n = 65, eGOS 5; n = 58, eGOS 5; n = 26, eGOS 6) with similar physiological trajectories and outcomes. However, as well as having a slightly better outcome, the cluster with an eGOS of 6 was older than the other two clusters and had a higher CPP and mean BP.

**Conclusions**

Clustering TBI patients based on physiological trajectories is possible. We identified outliers with distinct eGOS based on minute-by-minute physiology. The model is not able to identify groups of patients with distinct outcomes based on hour-by-hour clustering with outliers removed.

**References**

1. Brain IT Group. Brain Monitoring with Information Technology. Available from: http://www.brain-it.eu/about. [accessed 16/04/2015]

2. Jain AK, Murty MN, Flynn PJ. Data Clustering: A Review. ACM Comput Surv. 1999;31(3).

## P345 Brain compartment monitoring capabilities from ICP to BI (bioimpedance) during HS (hypertonic saline) administration. State of art simulation outcome depending on brain swelling type

### A. K. Kink ^1^, I. R. Rätsep^2^

#### ^1^Smartimplant Ltd., Tallinn , Estonia; ^2^NEMC, Tallinn, Estonia

**Introduction**

Changes of pressure inside cranium occur only when compartment inside has reached the compliance limit and changes below that point are not monitored. Second weakness of ICP is that local changes may have gone wrong long before ICP has reached the alarming value. Use of electrical BI is real-time and has high sensitivity to minor changes of volume shifts related to metabolism or HS infusion (1).

**Methods**

We used four electrode BI catheter connected to multi-frequency bioimpedance measurement device (Smartimplant Ltd.) (2). BI measurement provided continuously from frequencies 100 Hz to 100 Khz. All data have been recorded simultaneously with ICP and arterial pressure (AP) values.

**Results**

**Fig. 90 (Abstract P345).**
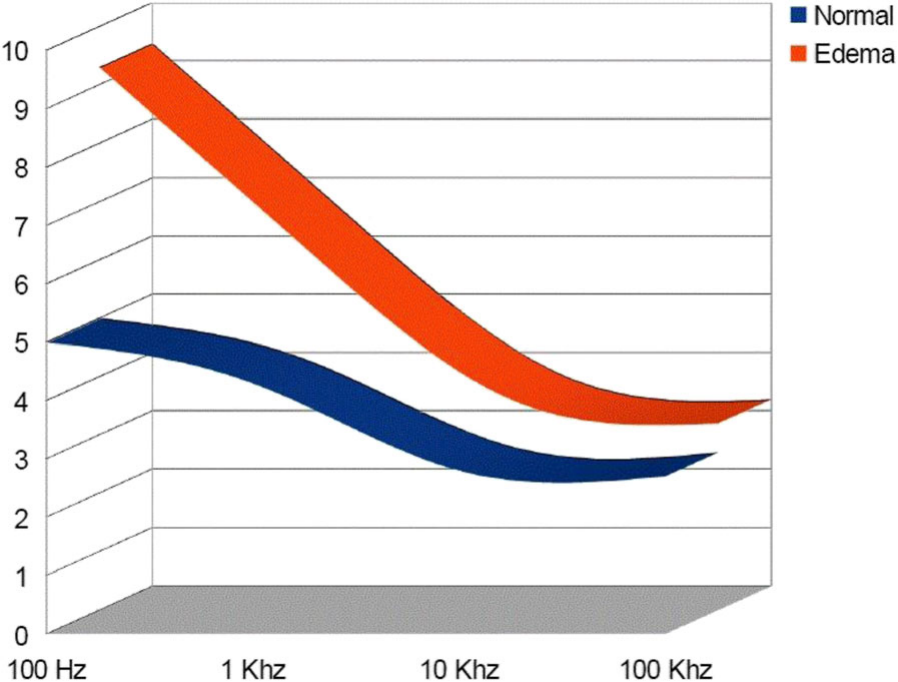
Brain tissue resistance “Z” changes in Ohms during cytotoxic edema

In ischemic tissue extravascular volume will decrease and we have cytotoxic edema. In case of microvascular and trauma damage we will have vasogenic edema and extracellular space will expand (Fig. 90). By administrating HS we can monitor changes of intracellular and extracellular volumes.

**Conclusions**

BI is fast and rapid indicator to any volume changes in tissue level and is useful method to measure brain tissue status in icu as indicator of HS administration resultativity and rebound effect.

**References**

1. Dowrick T, In vivo bioimpedance measurement of healthy and ischaemic rat brain: implications for stroke imaging using electrical impedance tomography. Physiol. Meas. 36 (2015) 1273–1282

2. Method and apparatus for determining conditions of a biological tissue US7970461B2

## P346 Transfusion of red blood cells in patients with traumatic brain injury admitted to Canadian trauma health centers: a multicenter cohort study

### A. Boutin^1^, L. Moore^1^, M. Chasse^1^, R. Zarychanski^2^, F. Lauzier^1^, S. English^3^, L. McIntyre^3^, J. Lacroix^4^, D. Griesdale^5^, P. Lessard-Bonaventure^1^, A. F. Turgeon^1^

#### ^1^Universite Laval, Quebec, Canada; ^2^University of Manitoba, Winnipeg, Canada; ^3^Ottawa Hospital Research Institute, Ottawa, Canada; ^4^Universite de Montreal, Montreal, Canada; ^5^University of British Columbia, Vancouver, Canada

**Introduction**

We aimed to evaluate RBC transfusion frequency, their determinants and associated clinical outcomes.

**Methods**

We conducted a retrospective multicenter cohort study using data from the National Trauma Registry of Canada. All patients admitted with a moderate or severe traumatic brain injury to one of the 114 participating centers from 2005 to 2013 were eligible. The registry contains information on age, gender, trauma severity, discharge status and discharge disposition. Data from the Discharge Abstract Database containing information on blood products, comorbidities, interventions and complications were linked to NTR observations. We conducted multivariate robust Poisson regression with a random effect at the center level. Missing data were handled through multiple imputation techniques.

**Results**

Over the course of their hospital stay, 1991 patients (28.19 %; 95%CI 27.16 to 29.25)) received RBC transfusions among 7062 patients suffering from traumatic brain injury, with frequencies varying from 0 to 43 % between centers. Female gender, age 55-65 (in reference to patients aged < 55), anemia, coagulopathy, sepsis, bleeding, hypovolemic shock, presence of multiple other comorbidities, face, thoracic and abdominal, spine, lower extremities and skin injuries, as well as invasive interventions were associated with higher frequency of RBC transfusions. Trauma severity and invasive interventions explained 76 % of the observed variation. Mortality, complications and discharge elsewhere than home were increased in patients who received RBC transfusions. ICU length of stay and hospital length of stay were also longer in patients who received RBC transfusions. When stratified, patients who were anemic on admission showed neither benefit nor disadvantage on any outcome relative to RBC transfusions. Similar results were obtained for stratum of patients who were diagnosed with sepsis on admission.

**Conclusions**

RBC transfusion is common in patients with traumatic brain injury and is potentially associated with unfavourable outcomes. Important variation between centers was observed, and highlights the need for stronger evidence over optimal transfusion practices in this population. Considering the potential impact of RBC on brain oxygenation in this population, rigorous trials evaluating Hb triggers for transfusion are needed.

## P347 Hemoglobin thresholds and red blood cell transfusions in adult patients with moderate or severe traumatic brain injury: a retrospective cohort study

### A. Boutin^1^, L. Moore^1^, R. Green^2^, P. Lessard-Bonaventure^1^, M. Erdogan^2^, M. Butler^2^, F. Lauzier^1^, M. Chasse^1^, S. English^3^, L. McIntyre^3^, R. Zarychanski^4^, J. Lacroix^5^, D. Griesdale^6^, P. Desjardins^1^, D. A. Fergusson^3^, A. F. Turgeon^1^

#### ^1^Universite Laval, Quebec, Canada,^2^Dalhousie University, Halifax, Canada,^3^Ottawa Hospital Research Institute, Ottawa, Canada,^4^University of Manitoba, Winnipeg, Canada,^5^Universite de Montreal, Montreal, Canada,^6^University of British Columbia, Vancouver, Canada

**Introduction**

In general intensive care units (ICU), the non-inferiority of restrictive transfusion strategies has been observed. Due to the risk of secondary cerebral lesions in traumatic brain injury, concerns have been raised regarding the impact of low hemoglobin and hypoxia in this population. Data remain scarce and clinical equipoise persists.

**Methods**

We conducted a cohort study of patients with moderate/severe traumatic brain injury admitted to the ICU of a level I trauma center (Halifax). Data related to pre-transfusion hemoglobin levels and red blood cells were collected. The association between hemoglobin level, red blood cell transfusion and mortality was evaluated using a robust Poisson model. We also constructed proportional hazard models with time-dependant variables (hemoglobin and transfusion) to evaluate survival. Neurological and non-neurological trauma complications as well as length of stay (LOS) in ICU or hospital were considered as secondary outcomes.

**Results**

We included 215 patients (78 % male; mean age: 45 years). A third of patients were transfused over their ICU stay (n = 66)). The median pre-transfusion hemoglobin levels in patients who were transfused were 81 g/L (IQR 67 to 100); and 110 g/L (IQR 93 to 123) in patients who did not receive red blood cells. Worse outcomes were significantly more frequent in patients who were transfused in adjusted models (mortality RR: 2.25 [95%CI 1.41 to 3.59]; neurological complications RR: 3.45 [1.43 to 8.32]; trauma complications RR: 1.64 [1.29 to 2.07]; ICU LOS geometrical ratio: 1.99 [1.45 to 2.73]; hospital LOS geometrical mean ratio: 2.38 [1.51 to 3.75]). A non-statistically significant trend toward higher risk ratios for unfavorable outcomes following transfusions in strata of patients with higher hemoglobin levels was observed. No significant modification of transfusion effect by age, presence of comorbidities, or traumatic brain injury severity was observed.

**Conclusions**

In our cohort, red blood cell transfusions were associated with worse outcomes in patients with traumatic brain injury, an association that tend to increase with hemoglobin levels. However, residual confounding from comorbidities and traumatic brain injury severity is possible.

## P348 Characteristics of patients with gunshot wounds to the head - an observational Brazilian study

### B. Goncalves, B. Vidal, C. Valdez, A. C. Rodrigues, L. Miguez, G. Moralez

#### Hospital Estadual Getulio Vargas, Rio de Janeiro, Brazil

**Introduction**

Penetrating gunshot wounds to the head (PGWH) are associated with high mortality and morbidity. Getulio Vargas Hospital is a public trauma center in Rio de Janeiro with a high volume of neurocritical patients and an unusual number of civil patients admitted with PGWH.

The aim of this study was to describe patients with PGWH admitted in Intensive Care Unit (ICU), investigating clinical characteristics, complications and management that could be related to a better outcome in this setting.

**Methods**

We retrospectively assessed the hospital records of every patient with PGWH admitted to the ICU from October 1st, 2014 to September 30th, 2015. Exploratory analysis of clinical data, image results, treatment, complication and outcomes was made. The primary end point was the modified Rankin scale at hospital discharge.

**Results**

In the period of the study, 1789 patients were admitted to the ICU and 13 PGWH patients were included. There were 10 male patients (77 %) and 3 female (23 %), with a mean age of 30 years (range 14-64). There were no self-inflicted lesions (all PGWH were results of aggression). Glasgow Coma Scale at admission was 8 or less in 10 patients. On admission, 7 patients were anisocoric, 8 presenting shock, 5 had associated body lesions from another gunshot wound (such as limbs or in the thorax). Mean SAPS 3 was 67 (range 35-94) and mean Apache II, 26 (range 8-37). The CT scan findings were: midline shift in 8 patients, 7 single lobe haemorrhages (such as frontal or parietal lobe), and 6 patients with bleeding in more than one lobe. Six patients had subarachnoid and 3 had intraventricular hemorrhage. Early surgical procedure was made to 10 patients (mainly decompressive craniectomy). The mean hospital length of stay was 21 days (range 2 to 136 days). Six patients had wound infection, and three, infection in other sites. The mortality rate for the entire group was 54 % (7 out 13). 4 patients progressed to brain death. Of the six patients discharged from the hospital, four had good outcome (defined as modified Rankin scores of 0-3), and two, bad outcome (modified Rankin of 4).

**Conclusions**

In agreement with previous reports, our results showed that the surviving group was mostly of hemodynamically stable patients, included all with GCS above 8, and had lower SAPS3 and APACHE 2 scores. Patients with multiple gunshot wounds, even on non-vital organs, had worse outcome.

## P349 Base excess as predictor for ICU admission and the injury severity in blunt trauma patients

### T. Hong

#### Yonsei University College of Medicine, Seoul, South Korea

**Introduction**

According to the advanced trauma life support; vital signs, mental status, base excess (BE), and serum lactate are valuable parameters to evaluate the hemodynamic status of the trauma patients. Only few studies have examined the role of admission BE in blunt trauma patients. To evaluate the value of admission BE in blunt trauma patients with respect to intensive care unit (ICU) admission rate and length of hospital stay.

**Methods**

From January 2014 to October 2014, 293 patients visited emergency room of our hospital due to the traumatic injury. In triage, Vital signs, Glasgow coma scale (GCS), BE, and SL were checked as initial parameters. Also study database included admission BE, length of hospital stay, mechanism of injury, injury severity score (ISS), and ICU admission. Admission BE drawn within 20 minute of arrival emergency room. The patients with intracranial hemorrhage, penetrating injury, age under 16 years old, and death on arrival were excluded. The patients were grouped by the Injury severity score: group I (<8), II (9 - 15), III (16 - 25), and IV (>26).

**Results**

A total of 293 patients were enrolled in our study. Among them 96 patients were admitted to ICU. Most common cause of ICU admission was driver traffic accident (n = 25, 26 %). Admission BE associated with significantly ICU admission (p < 0.000). The area under the curve of the receiver operating characteristic curve of admission BE for predicting ICU admission was 0.63. Also admission BE (odds ratio [OR], 0.904; 95 % confidence interval [CI], 0.823-0.992; P = 0.033) and ISS (OR, 1.146; 95 % CI, 1.100-1.194; P < 0.000) were determined to be predictors of ICU admission. BE seemed to show a weak correlation for the ISS (R Spearman = -0.30). Admission BE was significantly lower in the group IV than the remaining groups.

**Conclusions**

Admission BE in triage may be predicted injury severity and ICU admission in blunt trauma patients.

## P350 Enhancement of usual emergency department care with proadrenomedullin to improve outcome prediction - Results from the multi-national, prospective, observational TRIAGE study

### A. Kutz^1^, P. Hausfater^2^, D. Amin^3^, T. Struja^1^, S. Haubitz^1^, A. Huber^4^, B. Mueller^1^, P. Schuetz^1^

#### ^1^Kantonsspital Aarau, Aarau, Switzerland; ^2^Emergency Department, Groupe Hospitalier Pitié-Salpêtrière, Paris, France; ^3^Morton Plant Hospital, Clearwater, USA; ^4^Department of Laboratory Medicine, Kantonsspital Aarau, Aarau, Switzerland

**Introduction**

Previous studies have found Proadrenomedullin (ProADM), an inflammatory blood marker, to provide additional prognostic information for risk stratification. We aimed to translate ProADM cut-off levels into an easy-to-use emergency department (ED) triage algorithm to improve current risk assessments and clinical outcome prediction of medical patients.

**Methods**

In this large multi-national, prospective, observational study from Switzerland, France and the United States [1], we combined two ProADM cut-off values with a five-level ED risk assessment tool (Manchester Triage System, MTS) to further risk-stratify medical ED patients [2]. The performance of this algorithm was tested to predict adverse clinical outcome.

**Results**

Using data from 7132 consecutive ED patients (median age 62 years, 53.3 % male), the risk for 30 day mortality showed a stepwise increase from low (0.64 % [95 % CI 0.37-0.92]) in patients with a ProADM value of <0.75 nmol/L, to intermediate (4.38 % [95 % CI 3.58-5.18]) with a ProADM value >0.75 nmol/L and <1.5 nmol/L, to high (15.5 % [95 % CI 13.5-17.5]) with a ProADM value of >1.5 nmol/L (ANOVA, p < 0.0001). Combining initial ED triage (MTS) and ProADM reveals an observed risk for 30 day mortality of 0.5 % (14/2780 patients) in case of low priority initial triage and a ProADM value of <0.75 nmol/L until 23.1 % (96/416) in case of high priority initial triage and a ProADM value of >1.5 nmol/L. In low priority patients the overall 30 day mortality was 2.95 % (164/5550). Adding ProADM values of <0.75 nmol/L the relative risk reduction was -83 %, whereas adding ProADM values of >1.5 nmol/L increased the risk for mortality four times. In high priority patients (11.56 % overall 30 day mortality) relative risk reduction was -86 % adding ProADM values of <0.75 nmol/L, and doubled in patients with ProADM values of >1.5 nmol/L.

**Conclusions**

The proposed ProADM based triage algorithm allows a more accurate prediction for adverse clinical outcome in medical ED patients at high risk. To proof safety and efficacy of this new ED triage algorithm, an intervention study involving a rapid ProADM point-of-care measurement is mandatory.

**References**

1. Schuetz, P., et al., Crit Care, 2015. 19(1): p. 377.

2. Steiner, D., et al., J Emerg Med, 2015. (in press)

## P351 Developing an innovative emergency medicine point-of-care simulation programme

### T. Brown, J. Collinson, C. Pritchett, T. Slade

#### Royal Cornwall Hospital Trust, Truro, UK

**Introduction**

Simulation training within Emergency Medicine is growing with various peer-reviewed journals promoting simulation as a valuable educational tool [1,2]. High-fidelity mannequins are commonly used but considerable barriers remain, particularly a lack of human interaction. We suggest that a live patient during a point-of-care simulation teaching series could both create more realistic scenarios, and develop non-technical skills between the multi-disciplinary team. In addition, studies have shown that curriculum mapping optimises teaching in medical education and results in improved student satisfaction [3]. Thus, we set out to develop an innovative Emergency Medicine point-of-care simulation programme using live patients with an associated curriculum map.

**Methods**

We developed and implemented a six-month point-of-care simulation programme within the Royal Cornwall Hospital Emergency Department covering critical illness, paediatric, and major trauma presentations. A live clinically trained actor, rather than a high-fidelity mannequin, acted as the patient in suitable weekly simulations. A curriculum map was designed for each session detailing specific curriculum topics covered. Feedback was collected from 12 Emergency Medicine doctors using visual analogue scale questionnaires.

**Results**

Using a curriculum map, doctors’ knowledge of specific curriculum learning increased from 42 % to 88 %, an increase of 46 percentage points. 100 % stated that a curriculum map was useful/very useful. 100 % found interacting with a live patient a positive learning experience. 92 % stated it improved realism and the learning experience compared to a high-fidelity mannequin. 100 % felt a live patient was superior for non-technical skills.

**Conclusions**

Our research suggests that interaction with a live, simulated patient creates a realistic emergency medicine simulation providing the opportunity to develop non-technical skills within the multi-disciplinary team. We appreciate that particular scenarios may not be suitable for this approach. A comprehensive curriculum map in this context is valued by trainees, and improves trainee familiarity with their curriculum. Further work will involve evaluating any clinical/patient outcome improvements from participating in this simulation programme.

**References**

1. Ten Eyck RP. Pediatr Emerg Care 27(4): 333-341, 2011

2. Gjerraa K, Møller TP, Østergaard D. Acta Anaesthesiol Scand 58(7): 775-787, 2014

3. Wong R, Roberts J. BMC Med Educ 7: 42, 2007

## P352 The InSim program: an in situ simulation program for junior trainees in intensive care

### M. Le Guen, S. Hellings, R. Ramsaran

#### Manchester Royal Infirmary, Manchester, UK

**Introduction**

In situ simulation is increasingly recognised as an effective means of delivering education to clinicians within their own work environment [1,2]. We have developed the InSIM program, a novel in situ simulation program aimed at junior trainees within our ICU. The program focuses on developing both the clinical and non-clinical skills of trainees, improving multi-disciplinary teamwork and identifying areas for logistical improvement within our unit.

**Methods**

The simulation team developed three simulation scenarios: Alarming Ventilator; Massive Haemorrhage; and Tracheostomy Emergency. Each scenario has learning points addressing both clinical and non- technical skills and involves a structured debrief following the scenario. The initial program objective was to deliver a total of 15 simulations, including junior medical and nursing staff within each scenario.

**Results**

A total of eight out of 15 planned sessions were delivered. Feedback on the scenarios indicate that participants ‘Strongly Agree’ (72 %) or ‘Agree’ (18 %) that the scenarios were useful and improved their understanding of the clinical subject (Strongly Agree 60 %, Agree 40 %). They also ‘Strongly Agree’ (50 %) or ‘Agree’ (50 %) that discussion of the non-clinical aspects of the scenarios was useful. When asked to identify one thing they learnt from each scenario, multiple respondents identified non-technical aspects such as the importance of communication within the team, situational awareness and closed loop feedback.

A number of important logistical issues were identified through the simulation program. These included a lack of knowledge on the contents of the difficult airway trolley and a lack of familiarity on the correct procedure for activating the hospital’s Massive Haemorrhage Protocol.

**Conclusions**

We have been able to develop and deliver an in situ simulation program to our junior trainees within our ICU. The program has been well received and has increased junior trainees’ understanding and confidence in dealing with a number of emergent scenarios. It has also helped to develop and increase their awareness of key non-technical skills in these situations. Finally, it has identified a number of logistical issues within our intensive care unit, which are being addressed through quality improvement projects.

**References**

1. Walker ST, Sevdalis N, McKay A, et al Unannounced in situ simulations: integrating training and clinical practice. BMJ Qual Saf. 2013 June;22:453-8.

2. Rosen MA, Hunt EA, Pronovost PJ, et al In situ simulation in continuing education for the health care professions: a systematic review. J Contin Educ Health Prof. 2012 Fall; 32:243-54.

## P353 Impact of excessive and inappropriate troponin testing in the emergency setting how good are we

### A. Alsheikhly

#### Hamad Medical Corporation, Doha, Qatar

**Introduction**

The cardiac Troponin test has a key role in the diagnosis, prognosis and risk stratification of acute coronary syndrome (ACS). Over utilization and inappropriate requests for it have created a heavy workload for laboratory staff, increased costs to the health care system, and unnecessarily increased the length of stay and costs for patients. The aim of this study was to make and standardize all requests of Troponin more accurate and scientific without causing any burden to the patients, doctors or laboratory section.

**Methods**

A total number of 1073 requested serum Troponin-T Tests in the Emergency department)ED) of Hamad General hospital were reviewed retrospectively during the period from start of October/2014 to end of December /2014, then a data analysis was done including sex, race, age, a proper history taking and physical examination in addition to electrocardiographic (ECG) records and any necessary aid can be helpful before making any decision for Troponin request, then our results were compared to other studies and international results.

**Results**

Out of 1073 involved cases, there were only 208 patients with elevated serum Troponin-T levels, representing about 19.38 %, those whom were proved to have acute coronary syndrome were only 83, representing 7.7 % from the whole studied cases, (having a cardiac cause for their chest pain). Although patients who describe chest pain to the emergency physician represent an immediate challenge, but still we conclude that there is over utilization of Troponin test which increases workload and costs.

**Conclusions**

The majority of chest pain complaints are not due to (ACS). Non-cardiac chest pain is the second most common reason presentation to the ED and accounts for approximately 2 to 5 percent of all visits worldwide. Unnecessary and inappropriate requests for serum troponin should be reduced. The reduction in these requests eases the workload, cuts test and labor costs. Reducing unnecessary testing can lead to shortening stay times also beneficial for patients.

**References**

1. Ammann P, Maggiorini M. et al: Troponin as a risk factor for mortality in critically ill patients without acute coronary syndromes. J Am Coll Cardio 2003, 41:2004-2009.

2. Ammann P. Fehr T. et al: Elevation of troponin I in sepsis and septic shock. Intensive Care Med 2001, 27:965-969.

3. Pope JH, Selker HP. Acute Coronary Syndrome in the Emergency Department: Diagnostic Characteristics, Test, and Challenges: Cardiol Clin 2005, 23:432-435.

## P354 The development of time tracking monitor at emergency department

### T. Abe

#### University of Tsukuba, Tsukuba Medical Center Hospital, Tsukuba, Japan

**Introduction**

Examination time at emergency department (ED) could have influence for patient Ls prognosis. Door to balloon time is one of the most famous quality indicators related to patient Ls outcome. However, there have not been a lot of time quality indicators at ED except door to needle time and door to antibiotics time. Other door to examination or intervention time could be a quality indicator at ED. It will be used evaluation, improvement, and maintenance of ED. Our aim was to develop a real time monitor to register and manage patient Ls flow and time. We will make new time quality indicators at ED with this monitor. It will lead to hospital automation.

**Methods**

We developed the monitor system to track patient Ls flow and to show it in real time on a big display at ED in such a way of logistics system, which is connected with electronic health record (EHR). It can show time components such as door to intervention time. We named it Time Tracking Monitor (TTM) at ED.

**Results**

Our TTM system had three big appeals. First, it showed time data per examination or per intervention in real time. It helped to share patient Ls flow within medical staff. It would prevent to mistake. Second, this time data was being compared with a world standard time period based quality indicators such as door to balloon time in real time. It was also alerted if that time ran past the time limit. Moreover, we can analyze the relationship between diseases and time data because TTM has capability as database. Data analysis is used quality comparison among patients L individuals, within a hospital setting, among hospitals.

**Conclusions**

It has been still hard to manage patient Ls flow because there were variety of patients and diseases at ED although we used many automatic data accumulation with EHR. However, this monitor could bring medical staff to their attention of examination time and its importance.

## P355 Role of focussed echocardiography in emergency assessment of syncope

### L. Kanapeckaite, M. Abu-Habsa , R. Bahl

#### King’s College Hospital, London, UK

**Introduction**

Syncope remains a common Emergency Department (ED) presentation [1]. Almost 50 % of syncope cases are likely caused by a reflex response with excellent prognosis [1,2]. Unfortunately, up to 33 % of patients with syncope will be discharged with no clear diagnosis and are known to have an elevated risk of morbidity and mortality from cardiac causes [1-3]. We set out to develop a focused-echocardiography based algorithm to risk stratify this patient group.

**Methods**

Literature searches were carried out by the authors using Medline 1946-2015, Google Scholar, Cochrane Collection and international guidelines as well as reference searches. Our search converged on the role of comprehensive echocardiography in evaluation of syncope in parallel to utility of focused echocardiography in detecting structural cardiac disease.

**Results**

In patients with syncope, echocardiography is an imaging modality with high sensitivity and specificity for clinically significant structural disease [3]. Current guidelines only recommend formal echocardiographic evaluation in patients with clinically suspected structural cardiac disease [3]. Clinical assessment tools including composite risk scores have repeatedly been demonstrated to be poor at identifying structural heart disease [3]. Syncopal events in such context are associated with significant increase in mortality [1-3]. Studies have demonstrated that emergency physicians can attain a high level of proficiency in echocardiography allowing diagnostic evaluation of complex systolic and diastolic dysfunction and in operators with > 250 examinations, accurate identification of structural disease [3]. Focused-echocardiography can be safely integrated into the clinical assessment process in the presence of an experienced operator and yields findings superior to clinical assessment alone [3].

**Conclusions**

Clinical evaluation, laboratory investigations, and electrocardiography continue to be core investigations for patients with syncope. In the cohort of patients with no clear diagnosis, focussed-echocardiography by an appropriately trained operator offers additional safety-net for patients who are discharged without a clear diagnosis. Our paper assimilates these findings into a novel focused-echocardiography based algorithm for ED evaluation of syncope.

**References**

1. da Silva RM, Syncope: epidemiology, etiology, and prognosis. Front Physiol. 2014;5(December):8–11.

2. Soteriades E, Evans JC, Larson MG, et al. Incidence and Prognosis of Syncope. N Engl J Med. 2002;347(12):878–85.

3. Ref 3-48.

## P356 Insertion of an open-ended 14-gauge catheter through the chest wall causes a significant pneumothorax in a self-ventilating swine model

### MQ Russell^1^, KJ Real^2^, M Abu-Habsa ^3^, RM Lyon^1^, NP Oveland^4^

#### ^1^Kent, Surrey & Sussex Air Ambulance Trust, Kent, UK; ^2^Prometheus Delta-Tech, Herefordshire, UK; ^3^King’s College Hospital, London, UK; ^4^Stavanger University Hospital , Stavanger, Norway

**Introduction**

This real-time ultrasound (US) guided study aimed to determine the rate of pneumothorax progression after 14-gauge (G) cannula insertion in a self-ventilating swine model. Standard treatment of suspected tension pneumothorax includes emergent insertion of a large bore catheter into the pleural cavity [1]. Widely accepted practice includes a catheter is left in-situ, open-ended and not capped based on a quoted dictum that air will only significantly be entrained into the pleural space via a wound > ¨ø the diameter of the trachea [2].

**Methods**

Three swine were consecutively anaesthetised using total intravenous anaesthesia and remained self-ventilating without endotracheal intubation at all times. A single 14G cannula (BD Venflon) was inserted sequentially into the second intercostal space of each hemithorax under US guidance. Real-time US was used to identify the development of pneumothorax. Lung-point shift was used to track pneumothorax expansion within pre-mapped numeric chest wall segments. In the event of respiratory distress, air was aspirated through the cannula.

**Results**

Insertion of an uncapped 14G cannula resulted in a significant pneumothorax developing in <2 minutes. Each insertion (n = 3) resulted in the lung-point moving laterally by >10 cm in <2 minutes in all cases. At 2 minutes, swine were observed to develop a significant tachypnoea (respiratory rate >50/min) and showed signs of respiratory distress. Upon aspiration of 800 ml-1200ml of air, normal physiology was restored. During aspiration, the lung-point was observed to shift medially and apically over time, confirming lung re-inflation. Upon replacement of caps and monitoring over 5 mins, no subsequent pneumothorax or physiological distress were observed, suggesting that the expanding pneumothorax resulted from air entrained via the open ended catheter.

**Conclusions**

In this self-ventilating swine model, insertion of an uncapped, open-ended 14G catheter into the pleural space resulted in the rapid development of a large pneumothorax. If these data are transferable to humans, questions must be raised about leaving an open-ended catheter in the pleural space of a spontaneously breathing patient and this warrants further study.

**References**

1. Committee on Trauma, ATLS Manual 2012, 9th Edition, American College of Surgeons

2. Greaves and Porter, 1999, Pre-Hospital Medicine, Arnold Publishers

## P357 Ez-io® intraosseous access teaching in the workplace using a mobile ‘tea trolley’ training method

### J. Penketh, M. Mcdonald, F. Kelly

#### Royal United Hospital, Bath, UK

**Introduction**

EZ-IO® ‘tea trolley’ training aimed to provide emergency intraosseous (IO) access teaching for time pressured hospital practitioners. Recent guidelines advocate the use of emergency IO access when intravenous access is difficult [1], as battery powered devices like the EZ-IO® allow IO access within 49 seconds [2]. Despite introductory training few use IO devices on a regular basis. There is increasing recognition that lifesaving technical skills used in emergencies but required infrequently, need regular practise to prevent ‘psychomotor skill fatigue’ [3,4].

**Methods**

We adopted a novel technique for technical skill training, used initially for difficult airway training [4], to train anaesthetic and ICU staff for EZ-IO® access. A theatre trolley is converted into a mobile training trolley with EZ-IO® training devices, tea and cakes. The trolley is operated by two anaesthetists and visits theatres during normal working hours: one anaesthetist takes over the care of a stable patient in theatre while the second delivers a 15 minute training session in the anaesthetic room to the listed anaesthetist and nurse/operating department practitioner (ODP). Training includes EZ-IO® device locations, all practical aspects of their use, and is followed by tea, cake and a questionnaire.

**Results**

The EZ-IO® tea trolley was used to train a mixture of 36 consultants, juniors, nurses and ODPs. 97 % reported an increase in their confidence using the EZ-IO® system and 100 % found the ‘tea trolley’ a useful teaching method (mean Linkert 4.9, 5 = most useful). Following training 100 % of participants were correctly able to identify the locations of EZ-IO® devices for their clinical areas and identify the correct anatomical sites for IO insertion.

**Conclusions**

We have utilised the ‘tea trolley’ to improve EZ-IO® awareness and skills, and to provide mobile training sessions requiring minimal manpower and time commitments from participants who ordinarily have little time to practice. It is a transferable training approach which could be applied to many resuscitation skills and utilised in many departments.

**References**

1. RCUK 2015 Resuscitation Guidelines, UK, London,2015

2. Reiter D et al. The quality of cardiopulmonary resuscitation using supraglottic airways and intraosseous devices: A simulation trial. Resuscitation 84:93-7,2013

3. Campbell R et al. Student attainment of proficiency in a clinical skill: the assessment of individual learning curves. PLoS One 9:e88856,2014

4. O’Farrell G et al. ‘Tea trolley’ difficult airway training. Anaesthesia 70:104, 2014

## P358 Black widow envenomation in Saudi Arabia: a prospective observational case series

### M. Alfafi^1^, S. Alsolamy^2^, W. Almutairi^2^, B. Alotaibi^3^

#### ^1^Aseer Central Hospital, Abha, Saudi Arabia; ^2^King Saud bin Abdulaziz University for Health Sciences and King Abdullah International Medical Research Center, Riyadh, Saudi Arabia; ^3^King Fahad Medical City, Riyadh, Saudi Arabia

**Introduction**

Latrodectus is a genus of spider, which is the widely known black widow. It is characterised by neurotoxic venom. It has a worldwide distribution. The purpose of this study is to describe patients’ presentation, clinical manifestations and management course of patients with envenomations by black widow spiders in southern region of Saudi Arabia.

**Methods**

A Prospective observational case series study was performed at a tertiary hospital in southern region of Saudi Arabia over one year period .All adult patients with history of envenomations by black widow spiders included. We recorded the symptoms, physical signs, laboratory workup, management course and disposition of patients who came with envenomations by black widow spiders.

**Results**

A total of 8 subjects participated in the study. All of the patients were males with an age range of 25–58 years (median: 47 years). In all eight cases the black widow spider identification was made via a description of the spider. The time of onset of pain ranged from 30 minutes after the sting to 4 hours (median: 1 hour).Of the eight patients, three were asymptomatic , three had leg pain and two patients had abdominal pain. One patient had nausea and vomiting (12 %); the same patient had ptosis and sweating with neck rigidity and back pain (12 %).Upon examination, most of the symptomatic patients had redness over the sting location with no signs of compartment or swelling. Patients with abdominal pain had a rigid abdomen and guarding that mimicked an acute abdomen. blood test includes white blood count, kidney function test, coagulation profile, and creatine kinase. The results were normal in seven patients; only one patient developed acute kidney injury although his kidney function returned to normal upon discharge. In the treatment, all symptomatic patients received analgesia depending on the physician’s discretion. Three patients received calcium gluconate intravenously and reported no improvement in their symptoms. In addition, all patients received tetanus toxoid vaccine. Four of the patients required admission (50 %), but they were all discharged with a mean hospital of stay of 3.75 days.

**Conclusions**

Envenomation by black widow is a complex syndrome that manifests itself in various ways and results in various outcomes. The syndrome is generally self-limited and antivenom treatment is rarely necessary. Patients with significant pain that does not improve should be admitted for observation and possible treatment with antivenom.

## P359 Mechanical ventilation in patients with overdose not yet intubated on icu admission

### A. E Van den Berg^1^, Y. Schriel^2^, L. Dawson^2^, I. A. Meynaar^1^

#### ^1^HagaZiekenhuis, Den Haag, Netherlands; ^2^Reinier de Graaf Gasthuis, Delft, Netherlands

**Introduction**

Patients with an overdose or intoxication are admitted to the ICU for hemodynamic or respiratory monitoring. It was our impression that many patients who are monitored do not need interventions. The objective of this study was to estimate the incidence of mechanical ventilation within 24 hours after ICU admission in intoxicated patients who were not already ventilated on admission to the ICU.

**Methods**

The study was done in ICUs of the Reinier de Graaf Hospital in Delft and the HagaHospital in the Hague, both in the Netherlands. The study is a retrospective cohort study of all consecutive patients admitted to either ICU between 2010 and 2014 with a diagnosis of intoxication or overdose who were not mechanically ventilated or intubated on admission. The need for ethical approval was waived by the local ethical committee. All patient data are collected regularly in the databases of both units for administrative and quality improvement purposes. The study endpoint was mechanical ventilation within the first 24 hours after ICU admission.

**Results**

During the study period a total of 403 patients were admitted with an overdose or intoxication. Of these 403 patients, 91 patients were excluded since they were intubated and ventilated on admission. Characteristics of the study population of 312 patients are presented in the Table.

**Conclusions**

Our results show that only a small minority of patients (18/312 = 5.8 %) admitted with intoxication or overdose that are not ventilated on ICU admission develop the need for intubation and mechanical ventilation in the next 24 hours. A quarter of patients admitted with a GCS < 8 was subsequently intubated and ventilated in the first 24 hrs, whereas only 2.6 % of patients admitted with a GCS > =8 needed tracheal intubation and ventilation in the first 24 hrs after ICU admission. Our study shows that the GCS on admission is a significant predictor for the need of mechanical ventilation.

**Fig. 91 (Abstract P359).**
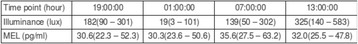
ᅟ

## P360 Central nervous system depressants poisoning and ventilator associated pneumonia: an underrated risk factor in toxicological intensive care unit

### H. Talaie

#### Toxicological Research Center, Department of Clinical Toxicology, Loghman-Hakim Hospital, Shahid Beheshti University of Medical Sciences, Tehran, Iran

**Introduction**

The objective of the present survey was to identify the ventilator-associated pneumonia (VAP) risk and prognostic factors among poisoned patients, who admitted to Toxicological ICU (TICU), especially in CNS depressants due to its prevalence and importance. VAP is a main cause of nosocomial infection in intensive care units (ICUs) which causes high mortality and morbidity.

**Methods**

A case- control study was conducted at Loghman Hakim Hospital between March 2013 and March 2014. Among 300 poisoned patients with mechanical ventilator > =48 hours, 150 patients who developed microbiologically-confirmed VAP were considered as VAP group and 150 without VAP were defined as control group. The data were collected by age, sex, type of poisoning, GCS, APACHE II score, length of hospital stay, previous antibiotic use, microbial culture of the trachea, body temperature, leukocyte count, and patients’ outcome. Based on the type of poisoning, patients were divided in 3 groups that included opioid (opium, heroin and methadone), CNS depressants (Antidepressant, Benzodiazepine and anti-convulsive) and other such as pesticide, methanol, 3, 4-methylenedioxy-methamphetamine (MDMA) and multi drugs. All data were expressed as mean (SD) for continuous variable and frequency for categorical variables. Logistic regression was used to determine the relationship between risk factors and VAP.

**Results**

Mean age of the patients was 33.9 ± 14.3 years. The probable VAP incidence and mortality were 22 % and 18.6 %, respectively. CNS depressant versus opioid (odds ratio, 3.74; p < .027), APACHE II (odds ratio, 1.28; p < .000) and Hospital length of stay (odds ratio, 2.15; p < .000) were the independent risk factor for VAP. While, APACHE II score (odds ratio, 1.12; p < .044) and Hospital length of stay (odds ratio, 2.15; p < .000) were the independent predictors of VAP mortality among these patients. The most common microorganisms in VAP cases were methicillin-resistant staphylococcus aureus (MRSA), and Acinetobacter sp in order to 56.7 % and 12.7.

**Conclusions**

CNS depressant was an important risk factor for VAP among poisoned patients. Hypoventilation due to CNS depression can lead to VAP. APACHE II and Hospital length of stay were shown as independent predictors of VAP and mortality among these patients. It seems that patients’ short turn over and hospitalization would be resulted in patients’ outcome.

## P361 Acute barium intoxication treated with hemodiafiltration

### D. Silva, S. Fernandes, J. Gouveia, J. Santos Silva

#### Santa Maria University Hospital, Lisboa, Portugal

**Introduction**

Soluble Barium salts poisoning is a rare and potentially fatal cause of severe hypokalemic paralysis. There is no agreement on adequate management, particularly on the use of renal support. We present a case of barium intoxication successfully treated with hemodiafiltration, resulting in rapid clinical improvement and undetectable barium levels.

**Methods**

Case report

**Results**

A 21-year-old male with a history of depression was admitted to our hospital emergency department two hours after the voluntary ingestion of a non-quantified amount of barium chloride, which the patient had procured and purchased online as a means for a suicide attempt. The clinical picture consisted of vomiting, diarrhea, and progressive generalized muscle weakness requiring tracheal intubation and mechanical ventilation, with subsequent ICU admission. Severe hypokalemia was documented (minimum K+ 1.2 mmol/L), complicated by frequent ventricular premature beats with prolonged QT interval (corrected value was 640 ms). Magnesium sulfate and a K+ infusion were administered, but serum K+ remained low. Serum K+ was then 2.2 mmol/L and barium levels were 0.04 mmol/L (toxic range). Continuous veno-venous hemodiafiltration (CVVHDF) was started. This resulted in a rapid decrease in barium levels and normalization of serum K+. Clinical improvement ensued with the correction of hypokalemia and restored muscular function, allowing spontaneous ventilation and extubation 6 hours later. Barium was persistently undetectable from 12 hours after the beginning of CVVHDF (<0.006 mmol/L). The patient was later transferred to the Psychiatry department, with an uneventful hospital stay.

**Conclusions**

There is very limited published data on the use of renal replacement techniques in acute barium intoxication, which is associated with a quicker barium half-life reduction and clinical improvement. A transcellular K+ shift and the barium concentration itself impact muscle weakness, implicating a possible direct effect of barium on either skeletal muscle or neuromuscular transmission. This patient presented with symptoms typical of severe barium intoxication, non-responsive to K+ supplementation. Clinicians in the ICU should remain aware of the potential ingestion of unusual chemicals such as barium chloride in the appropriate clinical context, as the risk of availability through internet purchases may continue to rise in the future. With this unique case we add on to the scanty existing evidence in the literature and strongly advocate the use of dyalisis, targeting both barium removal and the correction of hypokalemia.

**Consent to publish**

Written informed consent was obtained from the patient for publication of this abstract.

## P362 Major trauma presenting to the emergency department. the spectrum of cycling injuries in Ireland

### J. Foley, A. Kaskovagheorgescu, D. Evoy, J. Cronin, J. Ryan

#### St. Vincent’s University Hospital, Dublin 4, Ireland

**Introduction**

The Central Statistics Office Ireland (CSO) reported following the 2011 census that there was a 9.6 % increase in the number of people cycling to work compared with 2006. This leads to a higher prevalence of injuries and hospital attendances. The Road Safety Authority (RSA) conclude from their most recent research that there were 639 cycling associated collisions in 2012. We hypothesise that both the true number of cycling injuries and the major trauma that results from cycling collisions is being under-reported and this is placing a significant demand on Emergency Departments (EDs) and Intensive Care Units (ICUs) in Ireland.

**Methods**

This is a retrospective review of patients with a cycling-related injury presenting to Saint Vincent¡¯s University Hospital (SVUH) from 1st of January to 31st of December 2014. Subjects were identified by interrogating the Maxims Clinical© patient information system in use at the ED. Triage records were searched for the keywords; “bike”, “cycling”, “cyclist”, “pushbike”, “bicycle”. SVUH participates in the Trauma Audit and Research Network (TARN) and data was extracted from this database, e.g. the injury severity score (ISS) for patients who fulfilled the criteria for TARN.

**Results**

The total number of cycling associated injuries that attended our ED in 2014 was 534, accounting for just over 1.0 % of attendances in 2014. 380 (71.2 %) occurred in males. The mean age of the patients was 36.6. 79 cyclists (14.8 %) presented following a road traffic collision involving at least one vehicle. There was a wide spectrum of injuries with upper limb injuries sustained in 43 % (n = 243), head injuries in 16 % (n = 89) and facial injuries in 11 % (n = 84). 11 (2.1 %) patients were included in TARN with ISS varying from 4 to 24. An ISS ¡Ý15 is defined as major trauma and in our cohort, 5 patients fulfilled this criteria including 2 mortalities. These injuries included pneumothoraces, haemothoraces, spinal fractures, intracranial haemorrhage and skull fractures. All of these patients required intensive care review with 3 patients being admitted to the ICU.

**Conclusions**

Cycling injuries result in significant ISS with 5 patients in our cohort sustaining major trauma. There is significant demand placed on EDs by these injuries regarding trauma resuscitation procedures, use of specialised radiology and intensive care involvement. Cycling injuries are being under-reported in Ireland and studies such as this may help to guide injury prevention strategies in the future.

**Reference**

Census 2011. Central Statistics Office. December 2012

## P363 Burns from French military operations: a 14-year retrospective observational analysis.

### M. Huck, C. Hoffmann, J. Renner, P. Laitselart, N. Donat, A. Cirodde, J. V. Schaal, Y. Masson, A. Nau, T. Leclerc

#### Percy Military Teaching Hospital, Clamart, France

**Introduction**

Burns in combat usually represent 5-20 % of injuries [1]. The purpose of the study was to describe epidemiological characteristics and the management of burns during French military operations.

**Methods**

A retrospective observational study was performed of French casualties with burns, transported to Percy Burn Center (R4 Medical Treatment Facility) between January 1, 2001, and December 31, 2014.

**Results**

46 patients were included. 76 % of burns analyzed were non-battle injuries, mainly due to mishaps occurring with the burning of waste and the use of fuel. Combat burns (n = 11/46) were all explosions related (improvised explosive devices). The median total body surface area burned was 15 % [9;27]. Major anatomical regions affected were upper limbs (91 %), head/neck (78 %) and hands (70 %). Battle-related burns resulted in higher median Injured Severity Score, more patients under mechanical ventilation, more blood transfusions and surgeries, respectively 16 [6;23] vs 4 [1;8], 64 % vs 17 %, 8 [0;17] vs 0 and 3 [0;4] vs 0 (p <0.05). The combination of burn injury and multiple trauma occurred in 20 % (n = 9/46). The case fatality rate was 2 % (n = 1/46).

**Conclusions**

Majority of burns are non-battle injuries, small in size and accessible to prevention [2]. For battle-related burn injuries, the anatomical topography can be explained by the personal protective equipment and the higher severity score due to the associated trauma and mechanism. The initial management of burns with multiple trauma remains attention to the priorities of circulation, airway and breathing. The treatment of either the burn or the associated injuries may be compromised by their combined presence, and a team approach is essential to their optimal management [3]. Only patients with major burns that required hospitalizations have been included. So, this study is the visible tip of the iceberg. All military care providers should be familiar with the assessment and treatment of burns in military settings.

**References**

1. Cancio LC et al. J Burn Care Rehabil 2005;26:151-161.

2. Kauvar DS et al. J Burn Care Res 2009;30:700-704.

3. Dougherty W et al. Surg Clin North Am 1996;76:923-58.

## P364 A comparison of mortality scores in burns patients on the intensive care unit.

### O. Howarth, K. Davenport, P. Jeanrenaud, S. Raftery

#### Whiston Hospital, Prescot, UK

**Introduction**

Burn injuries are amongst the most severe physical and psychological insults a patient can experience and morbidity is extensive with variable mortality [1]. Studies have repeatedly confirmed factors associated with high mortality, which include increasing age, extent of burn and presence or absence of inhalational injury [2]. Predicting mortality from burns is useful in identifying those that may benefit from treatment or those in whom initiation of treatment is futile and not in the best interests of the patient. The objective of this surveillance study was to evaluate and compare the predictive value of Baux [3], Modified Baux (m-Baux) [4], and the Intensive Care National Audit and Research Centre (ICNARC) scores for overall outcome in our cohort of burns patients admitted to the ICU over the last 5 years.

**Methods**

A single centre, retrospective surveillance study was carried out on all patients with a total body surface area burn (TBSA) > =15 % admitted to the ICU between February 2011 and February 2015.

The Baux, m-Baux, and ICNARC scoring systems were compared with data analysis performed using logistic regression models. The fitness of each model was assessed using the Hosmer-Lemeshow, the Cox-Snell, and the Nagelkerke R2 statistics.

**Results**

45 patients were admitted to the ICU with burn injuries > =15 % TBSA, none were excluded from this study. 17 patients died resulting in a mortality rate of 37.7 %. On all three measurements, the Baux score had the highest R2 value (0.21, 0.25, 0.33).

We found that the odds ratio for survival changes by 0.96 (95 % c.i. 0.92 to 0.98) for each increase in Baux score by one unit.

**Conclusions**

Our data suggests that the Baux score is most useful for predicting overall mortality in patients with Burns versus the m-Baux and ICNARC scoring systems but all tests utilised have good discrimination and calibration for mortality prediction.

**References**

1. De Roche R et al. Epidemiological data and costs of burn injuries in workers in Switzerland: an argument for immediate treatment in burn centres. Burns 1994;20:58-60.

2. Clark CJ et al. Mortality probability in victims of fire trauma: revised equation to include inhalation injury. Br Med J 1986;292:1303-1305.

3. Baux S. Contribution a l’Etude du traitement local des brulures thermigues etendues. Paris:These;1961.

4. Osler T et al. Simplified estimates of the probability of death after burn injuries: extending and updating the Baux score. J Trauma 2010;68:690-697.

## P365 Clasification of pain and its treatment and an intensive care rehabiliation clinic

### P. MacTavish, H. Devine, J. McPeake, M. Daniel, J. Kinsella, T. Quasim

#### Glasgow Royal Infirmary, Glasgow, UK

**Introduction**

Treatment in an Intensive Care Unit (ICU) often necessitates uncomfortable and painful procedures for patients throughout their admission. There is growing evidence to suggest that chronic pain is becoming increasingly recognised as a long term problem for patients following an ICU admission [1]. Intensive Care Syndrome: Promoting Independence and Return to Employment (InS:PIRE) is a five week rehabilitation programme for patients and their caregivers after ICU discharge at Glasgow Royal Infirmary. This study investigated the incidence and location of chronic pain in patients discharged from ICU and classified the analgesics prescribed according to the World Health Organization analgesic

**Methods**

The InS:PIRE programme involved individual sessions for patients and their caregivers with a physiotherapist and a pharmacist along with interventions from medical, nursing, psychology and community services. The physiotherapist documented the incidence and pain location during the assessment. The pharmacist recorded all analgesic medications prescribed prior to admission and at their clinic visit. The patient’s analgesic medication was classified according to the WHO pain ladder from zero to three, zero being no pain medication and three being treatment with a strong opioid. Data collected was part of an evaluation of a quality improvement initiative, therefore ethics approval was waived.

**Results**

Data was collected from 47 of the 48 patients who attended the rehabilitation clinic (median age was 52 (IQR, 44-57) median ICU LOS was 15 (IQR 9-25), median APACHE II was 23 (IQR 18-27) and 32 of the patients were men (67 %)). Prior to admission to ICU 43 % of patients were taking analgesics and this increased to 81 % at the time of their clinic visit. The number of patients at step two and above on the WHO pain ladder also increased from 34 % to 56 %.

**Conclusions**

Of the patients seen at the InS:PIRE clinic two-thirds stated that they had new pain since their ICU admission. Despite the increase in the number and strength of analgesics prescribed, almost a quarter of patients still complained of pain at their clinic visit. These results confirm that pain continues to be a significant problem in this patient group. Raising awareness in primary care of the incidence of chronic pain and improving its management is essential to the recovery process following an ICU admission.

**References**

1. Griffiths J et al. Critical Care 17:R100, 2013

2. World Health Organization. Cancer pain relief, with a guide to opioid availability. 2nd edition. Geneva: WHO, 1996

## P366 Pain management adequacy in critical care areas ,the process and the barriers perceived by critical care nurses

### S. Alrabiee^1^, A. Alrashid ^2^, S. Alsolamy^3^

#### ^1^King Abdulaziz Medical City, National Guard Hospital, Riyadh, Saudi Arabia; ^2^Department of Management, College of Business Administration, King Saud University. Saudi Arabia , Riyadh, Saudi Arabia; ^3^King Saud bin Abdulaziz University for Health Sciences and King Abdullah International Medical Research Center, Riyadh, Saudi Arabia

**Introduction**

Despite the development and availability of effective analgesic procedures, pain is still underestimated and poorly managed, especially in the critical areas. Nurses’ knowledge about pain plays a significant role in effective clinical decision making for pain management. The objective of the study is describing the level of knowledge and practice of nurses in pain management as well as highlighting the possible barriers that nurses face in assessing and managing acute pain in the critical areas ICU and ER.

**Methods**

This study a cross-sectional study. A semi-structured questionnaire was distributed to the nurses in the critical care areas in tertiary care hospital. These nurses have direct contact with patient care in clinical settings and must provide pain assessment and management to their patients. Measurement investigated like the demographic data, work ranking, level of education and years of experience. As well their actual level of knowledge and practice and the perceived barriers about pain assessment and management during their clinical work.

**Results**

A total of 600 nurses participates in the study with a response rate of 70 %, 14.3 % male, 85.7 % female. 81 % of the nurse participants reported that they are documenting pain on a routinely bases as they document more than 75 % of the assessments while 2 % of them document rarely . 62 % of nurse participants always agree with patient statements about their pain. 83 % of the nurse participants indicate that they have adequate knowledge and 17 % are none. 75 % only of nurse participants have read the guidelines about pain management. Out of the 14 reasons the majority of nurses identifies working load (70 %) as a barrier against adequate pain management while (65 %) identify the use of sedation & language issues by (2 %).

**Conclusions**

Although the critical care nurses who participated in this study have good exposure to different critical specialties, but still the inadequate pain management is significant. The level of knowledge and the perceived barriers are playing a major role in the adequacy of pain management practiced in the hospital and those needs to improve.

**References**

1. Batiha, A. (2014). Pain management barriers in critical care units: A qualitative study. , 3(1), 1-5.

2. Kizza, I. B. (2012). Nurses’ knowledge and practices related to pain assessment in critically ill patients at Mulago Hospital, Uganda. 63-70.

## P367 Pain assessment in critically ill adult patients: validation of the Turkish version of the critical-care pain observation tool

### O. Gundogan^1^, C. Bor^1^, E. Akýn Korhan^2^, K. Demirag ^1^, M. Uyar^1^

#### ^1^Ege University Hospital, Izmir , Turkey; ^2^Katip Celebi University, Health Sciences Faculty, Izmir, Turkey

**Introduction**

Critical-Care Pain Observation Tool (CPOT) and The Behavioral Pain Scale (BPS) are behavioral pain assessments for unconscious intensive care unit (ICU) patients. The aim is to determine the validation and reliability of the CPOT in Turkish in mechanically ventilated adult ICU patients.

**Methods**

This prospective observational cohort study included 50 mechanically ventilated mixed ICU patients who were unable to report pain. The study was conducted in a tertiary ICU in a university hospital in Turkey.

After obtaining permission from the author of the original study (1), CPOT was translated into Turkish and language validity was performed according to the reports obtained from 10 senior intensivists. Pain was assessed before and during non-painful and painful routine care procedures (touching with wet towel and suctioning respectively) using the CPOT and the BPS performed by a resident and an intensivist concomitantly. Tests reliability, interrater reliability, and validity of the CPOT and the BPS were evaluated.

**Results**

Thirty three of the patients (66 %) was male. The mean age was 57.4 and the mean APACHE II score was 18.7. Glasgow Coma Score was equal or less than 8 in 66 % of all patients. A total of 100 assessments performed by a resident and an intensivist were recorded from 50 patients using the CPOT and the BPS. Scores of the CPOT and the BPS during the painful procedures were both significantly higher than those during the nonpainful procedures. The agreement between CPOT and BPS during painful and nonpainful stimuli was ranged as; sensitivity 66.7 % - 90.3 %; specificity 89.7 % - 97.9 %; kappa value 0.712 - 0.892. The agreement between resident and intensivist during painful and non-painful stimuli was ranged from 97 % to 100 % and the kappa value was between 0.904 - 1.0.

**Conclusions**

The Turkish version of the CPOT showed good correlation with the BPS. Inter-rater reliability between resident and intensivist was good. The study showed that the Turkish version of BPS and CPOT are reliable and valid tools to assess pain in intubated and unconscious mechanically ventilated critically ill patients for use in daily clinical practice.

**Reference**

1. Gelinas C et al.: Am J Crit Care 2006; 15: 420-427

## P368 An audit of pain and sedation assessments in the intensive care unit: recommendations for clinical practice

### F. Frame, C. Ashton, L. Bergstrom Niska

#### Milton Keynes University Hospital NHS Foundation Trust, Milton Keynes, UK

**Introduction**

Assessing and managing pain and sedation levels in patients who are mechanically ventilated in the intensive care unit is often challenging but essential in order to provide holistic patient-centred critical care [1,2].

The aim of this audit was to evaluate the assessment of pain and sedation in the intensive care unit at Milton Keynes University Hospital NHS Foundation Trust, by examining current clinical practice and highlighting areas for improvement.

**Methods**

Guidelines were utilised to formulate audit criteria and standards. A proforma was developed for use in 50 adult patient cases who were mechanically ventilated in the intensive care unit over a 3 month period between August and October 2015. All data were coded and entered into a spreadsheet for analysis. Initial recommendations for change have been made.

**Results**

90 % of patient cases had a pain assessment documented within 6 hours of admission, scored utilising the VAS. 73 % of initial pain assessments were scored by nursing staff, with self-reporting occurring in the remaining 27 %. When pain scores were subsequently evaluated over a following 24 hour period, the mean number of scores documented was n = 5.

When sedation scores were evaluated utilising the RASS over the same 24 hour period, the mean number of scores documented was n = 6 (ranging from -5 to +2). Of note, 60 % of patients did not have a formal sedation hold, with 66 % having no reason for this documented. In addition, only 46 % of patients had a goal RASS documented on the daily ward round, and of these 39 % met their target RASS.

**Conclusions**

This audit has raised awareness of the need for improvement in the assessment of pain and sedation in the intensive care unit. As a result, a new clinical guideline has been developed and an educational intervention will be designed to integrate this into daily clinical practice. A second audit cycle will take place in 2016.

**References**

1. Gelinas, C Chanques, G & Puntillo, K 2014, ‘In pursuit of pain: recent advances and future directions in pain assessment in the ICU’, Intensive Care Medicine, 40, 1009-1014

2. Jackson, DL Proudfoot, CW Cann, KF & Walsh, T 2010, ‘A systematic review of the impact of sedation practice in the ICU on resource use, costs and patient safety’, Critical Care, 14, R59

## P369 Impact of pharmaceutical care on treatment of pain and agitation in medical intensive care unit

### P. Dilokpattanamongkol^1^, T. Suansanae^1^, C. Suthisisang^1^, S. Morakul^2^, C. Karnjanarachata^2^, V. Tangsujaritvijit^2^

#### ^1^Faculty of Pharmacy, Mahidol University, Bangkok, Thailand; ^2^Faculty of Medicine, Ramathibodi Hospital, Mahidol University, Bangkok, Thailand

**Introduction**

As underlying diseases and causes of agitation are varied in different patients, a selection of sedative agents should be made individually. Currently, there is a lack of guideline for the sedative use in the medical intensive care unit (ICU), Ramathibodi Hospital. A prescription of sedative agents performed by critical care pharmacists during patient care rounds, based on appropriate guidelines and patient data would shorten the ICU length of stay (LOS). This study was set out to investigate the impact of pharmacist interventions on ICU LOS, hospital LOS, ventilator days and mortality in Medical Intensive Care patients at Ramathibodi Hospital.

**Methods**

A before-after study at the medical ICU (total of 8 beds) of the university hospital was conducted on mechanical ventilated patients receiving sedative agents. Baseline characteristics, active problems and APACHE II were used as the criteria to match the patients between two groups, the retrospective group (no pharmacist interventions) and the prospective group (pharmacist interventions). Medical chart reviews were performed and data were collected over a 2-year period in the retrospective group. In the prospective group, pharmacists made interventions with the team to select sedative agents to individual patients based on underlying disease, active problems, renal and hepatic function, and causes of patient agitation.

**Results**

There were 156 mechanically ventilated patients prescribed sedative agents, 66 and 90 patients in the prospective and retrospective group, respectively. The median duration of ICU LOS was reduced from 10.0 days in the retrospective group to 6.5 days in the prospective group (p = 0.002). The median hospital stay and ventilators days were significantly different between two groups. The median hospital stay was reduced from 30.50 days in the retrospective group to 17.50 days in the prospective group(p = 0.000). The ventilator days were 14 days and 8.5 days in retrospective and prospective group, respectively (p = 0.008). Mortality remained unchanged, 53.03 % in the prospective group compared to 46.67 % in the retrospective group (p = 0.432).

**Conclusions**

The pharmacist participations in critical care team resulted in a significant reduction in the duration of ICU stay, hospital LOS and ventilator days, but not mortality in mechanically ventilated patients receiving sedation.

**References**

1. Barr J, et al. Clinical practice guidelines for the management of pain, agitation, and delirium in adult patients in the intensive care unit. Crit Care Med 2013;41:263-306.

2. Marshall J, et al. Impact of a clinical pharmacist-enforced intensive care unit sedation protocol on duration of mechanical ventilation and hospital stay. Crit Care Med. 2008 Feb;36(2):427-433.

## P370 Agitation in trauma ICU, prevention and outcome

### S. Mahmood, H. Al Thani, A. Almenyar

#### Specialist Anesthesia, Doha, Qatar

**Introduction**

Agitation is a syndrome characterized by the acute onset of central nervous system dysfunction identified by the several features including a change or fluctuation in baseline mental status, and either disorganized thinking or an altered level of consciousness. Agitation occurs frequently in critically ill patients and intensive care unit (ICU).

**Methods**

A retrospective study with waiver of consent was conducted in one of the trauma intensive care unit (ICU) for one year. ICU patients were enrolled in the study and classified based on their agitation status. The depth of sedation along with the Ramsey Sedation Scale (RS), Glasgow Coma Scale (GCS), type of injury and observation of vital signs were clinically evaluated.

**Results**

One hundred and two patients (n = 102) were enrolled during the period of the study. Among those patients, forty six patients (n = 46) were agitated. Comparing the two groups of patient, agitated patients had higher incidence of infection (p < 0.02) compared to the non-agitated patients. In addition, there was a significant difference type of sedation used (p < 0.001), ventilators free days (p < 0.001) and length of stay.

**Conclusions**

Agitation in ICU patients is associated with several adverse outcomes including prolonged stay, nosocomial infections, and unplanned extubations.

**References**

1. Bay EJ, Algase DL. Fear and anxiety: a simultaneous concept analysis. Nursing Diagnosis.

2. Bergbom-Engberg I, Haljamae H. Assessment of patients’ experience of discomforts during respirator therapy.

3. Berniker E, McNabb DE. Dialectic inquiry: A structured qualitative research method. The Qualitative Report. 2006;11(4):643–664.

4. Broyles LM, Colbert AM, Tate JA, Swigart VA, Happ MB. Clinicians’ evaluation and management of mental health, substance abuse, and chronic pain conditions in the intensive care unit.

## P371 Correlation between percentages of ventilated patients developed vap and use of sedative agents in icu patients.

### A. Vakalos , V. Avramidis

#### Xanthi General Hospital, Xanthi, Greece

**Introduction**

The aim of our observation retrospective study was to test the hypothesis that a correlation exists between percentages of ventilated patients developed VAP (% VP) and the use of main sedative and neuromuscular blockage agents indexes in our both medical and surgical ICU served in community hospital.

**Methods**

From January 2008 to December 2012 admitted to our ICU 384 patients. From our database we looked for percentage of ventilated patients developed VAP as well as the use of the following agents as items per ventilation day per ventilated patient: Midazolame (amp 50 mg), Propofol (vial 2 % 50 ml), Sisatracurium (amp 5 ml/10mg) per year from 2008 to 2012. Using linear correlation method, we looked for linear slope, correlation coefficient (r), and coefficient of determination (r2), and by linear regression method using ANOVA test we looked for p value, according percentage of ventilated patients developed VAP and use of Midazolam, Propofol and Sisatracurium.

**Results**

Table 46

**Conclusions**

According to our data, there was no statistically significant correlation detected between percentages of ventilated patients developed VAP and use of Midazolam, Propofol and Cisatracurium. On the other hand, not all but some patients had increased demand for more sedative agents. Our data suggest that the percentages of ventilated patients developed VAP is independent from the use of main sedative and neuromuscular blockage agents in all ICU ventilated patients.

**Table 46 (Abstract P371).** Correlation between % VP and use of sedative and neuromuscular blockage agents

	Slope	St. Error	r	r2	L. CI	U.CI	p value
Midazolam	0.072	0.088	0.425	0.161	-0.210	0.354	0.4750
Propofol	0.282	0.222	0.591	0.349	-0.424	0.989	0.2934
Cisatracurium	0.012	0.007	0.694	0.481	-0.011	0.036	0.1904

## P372 Improving recording of sedation events in the Emergency Department: The implementation of the SIVA International Taskforce adverse event reporting tool for procedural sedation

### R. Sharvill^1^, J. Penketh^1^

#### ^1^Royal United Hospital, Bath, UK

**Introduction**

Sedation is routine in UK emergency departments (EDs); to maintain and improve our quality of care, and compare local practice with national and international standards, a standardised and sensitive tool is needed to detect and report adverse events (AE). The World SIVA International Sedation Taskforce tool [1] incorporates physiological thresholds, clinical interventions, and overall outcome to grade AE as minimal to sentinel. We incorporated this tool into our sedation documentation, and report its effect on the detection and documentation of AEs.

**Methods**

All notes from patients undergoing sedation in our ED between 1/1/14 and 30/4/14 were retrospectively reviewed for documentation of AE, as well as evidence of adverse events where not formally recorded. After implementation of a new sedation proforma incorporating the SIVA tool [1], the data collection process was repeated retrospectively between 1/10/14 and 30/1/15.

**Results**

See Table 47.

**Conclusions**

The SIVA tool highlights AEs within our ED have gone unreported. Its incorporation improved awareness and documentation of AE, but further education is needed to improve the accuracy of reporting. Utilising this tool should facilitate more sensitive and standardised data collection, allowing regular review and improvement of practice, as well as comparison with national and international standards.

**Reference**

1. Mason KP et al:. BJA 2012; 108(1): 13-20.

**Table 47 (Abstract P372).** AE documentation before and after intervention

	Before implementation of SIVA tool (n = 43)			After implementation of SIVA tool (n = 67)		
	AE formally recorded by sedating clinician	Evidence of AE in notes	Unrecorded AE (as % of total AE)	AE formally recorded by sedating clinician	Evidence of AE in notes	Unrecorded AE (as % of total AE)
Minimal	0	0	0	4	6	2 (33%)
Minor	0	7	7 (100%)	2	14	12 (86%)
Moderate	0	1	1 (100%)	0	0	0

## P373 Impact of sedative drug use on the length of mechanical ventilation

### S. E. Morton^1^, Y. S. Chiew^1^, C. Pretty^1^, J. G. Chase^1^, G. M. Shaw^2^

#### ^1^University of Canterbury, Christchurch, New Zealand; ^2^Christchurch Hospital, Christchurch, New Zealand

**Introduction**

Sedation of invasive mechanically ventilated (MV) Intensive Care Unit (ICU) patients is common. However, recent studies found increased sedation delivery can adversely affect MV patient outcomes. A retrospective audit of length of MV (LoMV) and sedation delivery in the Christchurch Hospital ICU over 3 calendar years (2012-2014) was carried out.

**Methods**

Data on sedative drug (Dexmedetomidine, Fentanyl and Propofol) usage and LoMV were collected from hospital records. Total sedative drug use and LoMV were assessed monthly and compared between years using a Wilcoxon Ranksum and Kruskal-Wallis test. Zero-lag cross correlations were used to assess the relationship between month-to-month trends in LoMV and sedation use. A Monte-Carlo simulation was performed to assess the impact of potential outlier months on the results (4 of 36 months were removed without replacement and the analysis repeated 10,000 times). Standardised Mortality Rate (SMR) was calculated each year to account for changes in cohort.

**Results**

**Fig. 92 (Abstract P373).**
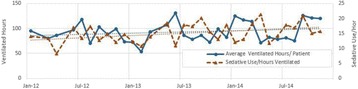
ᅟ

In 2012, the median [Interquartile range (IQR)] of LoMV hours was 92 [IQR: 77-101] while 2013 was 86 [IQR: 75-103] and 2014 was 107 [IQR: 80-121]. The median LoMV per patient for 2013 and 2014 were significantly higher than 2012 (p < 0.05) (92 and 107 hours vs 86 hours). Median [IQR] for total sedative drug usage was 13 [IQR: 12-16] mg/hour LoMV in 2012, 15 [IQR: 13-18] in 2013 and 16 [IQR: 14-18] 2014. Usage in 2013 and 2014 were significantly higher than 2012 (p < 0.05). The zero-lag cross-correlation between LoMV and sedation usage per month over the 3 years was 0.76 indicating that these rising trends are well mirrored, as additionally shown in Fig. 92. Monte Carlo simulation shows that the trend of increasing sedation usage associated with increasing LoMV is robust, with 98 % of the simulation indicating an increasing trend.

Over the study period, the SMR was 0.71 (2012), 0.58 (2013) and 0.61 (2014) for MV patients suggesting that increased LoMV was not due to a significantly more ill MV cohort.

**Conclusions**

Sedative drug use per ventilated hour is strongly associated with LoMV and the increase of these metrics over the last 3 years is significant and strongly associated.

## P374 Co-administration of nitric oxide and sevoflurane using anaconda

### R. Knafelj^1^, P. Kordis^2^

#### ^1^Rihard Knafelj, Ljubljana, Slovenia; ^2^Clinical Center Ljubljana, Ljubljana, Slovenia

**Introduction**

Sedation in ICU using sevoflurane via Anaconda enables desirable sedation level with numerous advantages over propofol/midazolam based sedation. In patient with severe ARDS inhaled nitric oxide (NO) improves oxygenation and reduces right heart afterload. Both therapies are simultaneously used in our ICU with ARDS patients and patients with right heart failure being ventilated for various reasons. Since sevoflurane can interact with certain gas monitoring (eg. Quark rmr indirect calorimetry) we examined potential gas readings interaction with NO.

**Methods**

NO circuit (Carefusion) and Anaconda were connected to ventilator tubing and gas concentrations was measured at different circuit outlets. Ventilator (NPB 840) was set to V-AC, 450 mL and 16 BPM. Two sevoflurane concentrations (5 mL/h and 8 mL/h) and three FiO2 (air, 50 % and 100 % oxygen) were tested against constant NO flow (20 ppm).

**Results**

At constant V-AC ventilation and constant NO flow, inspiratory concentration NO readings showed minimal (NS) variations during different FiO2 or sevoflurane concentration administered. Similarly, administering constant NO and 2 different sevoflurane concentration did not affect sevoflurane concentration readings.

**Conclusions**

Co-administration of NO and sevoflurane via Anaconda does not affect gas analysis and can be safely used as a sedation and ARDS salvage therapy.

**References**

1. Mehra S, Garg A, Dalvi PB, Taneja S, Nindra P, Ray S: Inhaled NO as a salvage therapy in ARDS

2. Ferrando C, Moreno J, Soro M, Belda J. Efficacy and safety of sedation with sevofurane administered by the device AnaConDa in ARDS.

3. Acute Respiratory Distress Syndrome The Berlin Definition. JAMA. 2012;307(23):2526-2533

**Fig. 93 (Abstract P374).**
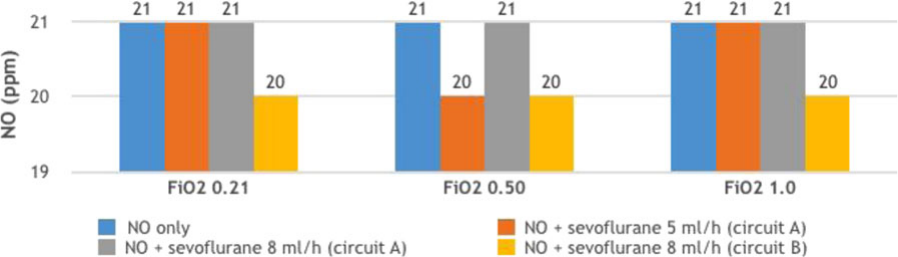
NO concentration

**Fig. 94 (Abstract P374).**
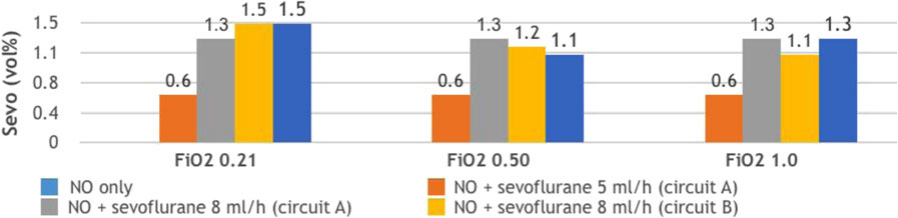
Sevoflurane concentration

## P375 A retrospective study of the use of Dexmedetomidine in an oncological critical care setting

### S. Patel, V. Grover

#### The Royal Marsden Hospital, London, UK

**Introduction**

Dexmedetomidine (DEX) may spare other sedative use, reduce delirium incidence, facilitate earlier extubation, and thereby prevent the need for a tracheostomy as well as reduce hospital expenses. There is however a paucity of data in patients with cancer. We sought to evaluate the clinical and cost-effectiveness of the drug in this setting.

**Methods**

A retrospective review of electronic records between February 2014 and March 2015 was conducted. Sedative use, ventilator weaning, extubation rates and tracheostomy requirement were assessed. In addition, a cost-analysis per patient was determined.

**Results**

Twenty-nine patients (19 male) received DEX in the study period (age range 19-82 years). The median length of stay was 19.6 days (interquartile range (IQR) 13.0-26.3) as a result of prolonged mechanical ventilation (median 15.8 days (IQR 8.0-23.0). Nineteen patients survived to ICU discharge. Eighteen patients (61.1 %) required a tracheostomy, with a median duration of 18 days (IQR 10.5-19.77). DEX was used to facilitate sedation reduction in those patients who were difficult to wean, manage agitation and delirium and to expedite extubation/decannulation. It was utilised for a median of 79 hours (IQR 144-47) with a median dose of 0.91 micrograms/kg/hr (IQR 0.57-1.13). It was deemed to have improved the defined outcomes for 12 patients (41 %). Bradycardia and/or hypotension was observed in 17 patients within 4-hr of DEX treatment, almost half required support. The mean cost of DEX treatment was £630 per patient. This approximates to £1500 per patient with clinical benefit. This cost was offset by the reduction of sedation (11 patients) in those patients with difficult to manage agitation with other agents, as well as ventilator weaning, earlier extubation and avoidance of tracheostomy (10 patients).

**Conclusions**

This cohort of patients belonged to a group with high mortality and high length of stay as a result of prolonged ventilation, associated with difficult to manage sedation and/or delirium. Further studies should evaluate the optimal duration of treatment, to facilitate de-escalation in those patients not obtaining benefit.

**References**

1. Curtis J et al. Propofol-Based Versus Dexmedetomidine-Based Sedation in Cardiac Surgery Patients. J Cardiothorac Vasc Anesth. 2013; 27(6): 1289-9

2. Thoma BN et al. Clinical and economic impact of substituting dexmedetomidine for propofol due to a US drug shortage: examination of coronary artery bypass graft patients at an urban medical centre. Pharmacoeconomics. 2014; 32(2): 149-57

## P376 Dexmedetomidine and posttraumatic stress disorder incidence in alcohol withdrawal icu patients

### I. Kuchyn, K. Bielka

#### Bogomolets National Medical University, Kiev, Ukraine

**Introduction**

Alcohol withdrawal patients have the increased risk of PTSD [1], especially when treated at intensive care unit (ICU) with high BZD doses. We hypothesized that dexmedetomidine addition to BZD for AWS patients’ sedation in the ICU will decrease PTSD incidence.

**Methods**

Prospective cohort study was conducted in the mixed ICU during July 2014 - July 2015. AWS patients were assigned in 2 groups: group D (dexmedetomidine) and group C (Control). In group D dexmedetomidine infusion at dose 0.2-1.4 μ g/kg/h prescribed for sedation and AWS symptoms control. BZD (diazepam 10 mg) and antipsychotics (haloperidol 5 mg) were prescribed in both groups with similar symptom-triggered local protocol. Posttraumatic stress disorder was diagnosed according to the Diagnostic and Statistical Manual of Mental Disorders, 4th Edition criteria by a psychiatrist using a clinical interview within 1, 3 and 6 months after hospital discharge.

**Results**

98 patients were included in the study. 8 % of the patients in the Gr. D and 26 % of pateints in gr.C had a posttraumatic stress disorder diagnosis 1 month after hospital discharge (p = 0.03). At 3-month follow-up 17 % of patients in gr.D and 36 % in gr.C were diagnosed PTSD (p = 0,04), after 6 months – 15 % and 30 %, respectively (0,03).

**Conclusions**

Dexmedetomidine addition to basic AWS therapy with benzodiazepines and antipsychotics, may decrease risk of PTSD for alcohol withdrawal patients in ICU.

**Reference**

1. Parker AM, Sricharoenchai T, Raparla S, Schneck KW, Bienvenu OJ, Needham DM. Posttraumatic stress disorder in critical illness survivors: a metaanalysis. Crit Care Med. 2015 May;43(5):1121-9.

**Table 48 (Abstract P376).** PTSD incidence in study groups

Assessment period / Group	Group D, n = 48	Group C, n = 50	Odds ratio [95% confidence interval]	p value
1 month, n (%)	4/48 (8)	13/50 (26)	4 [1, 06-17]	0,031
3 month, n (%)	8/48 (17)	18/50 (36)	2,8 [1-8]	0,04
6 month, n (%)	7/48 (15)	15/50 (30)	3 [1,03-10]	0,03

## P377 Hemodynamic effects of dexmedetomidine in a porcine model of septic shock

### Z. Aidoni, V. Grosomanidis, K. Kotzampassi, G. Stavrou, B. Fyntanidou, S. Patsatzakis, C. Skourtis

#### Aristotle Medical School, Thessaloniki, Greece

**Introduction**

Dexmedetomidine is a selective a2-adrenoreceptor agonist and due to its unique actions on central nervous system it has been widely used as a sedative agent in the Intensive Care Unit and in the operating theatre. The aim of our study was to investigate if dexmedetomidine can be used effectively and safely as a sedative agent in a porcine model of septic shock.

**Methods**

32 pigs under general anesthesia and mechanical ventilation were included in this study and were randomly assigned into one of the 4study groups (G-A, G-B, G-C, G-D-each group consisted of 8 animals). All pigs received continuous infusion of propofol and rocuronium. In G-A, no other intervention was performed, in G-B dexmedetomidine was administered in a dose of 0.8mcg/kg/hr, in G-C sepsis was induced by intravenous administration of LPS and in G-D both dexmedetomidine infusion and sepsis induction were performed in a similar manner like in G-B and G-C respectively. Study parameters included heart rate-HR, mean systemic-mSAP and pulmonary artery blood pressure-mPAP, central venous pressure-CVP and pulmonary artery occlusion pressure-PAOP. Parameters were recorded at baseline (phase-P0) and every 30 min for 3 hrs (P1-6). For the statistical analysis, repeated measures ANOVA was used (SPSS21).

**Results**

HR, mSAP and mPAP values are depicted on the following Table. CVP and PAOP did not show any significant alterations.

**Conclusions**

According to our study results, dexmedetomidine has positive effects both on HR and on sepsis induced pulmonary hypertension. The decrease of mSAP was similar in all study animals, both in the septic and the non-septic ones.

**Fig. 95 (Abstract P377).**
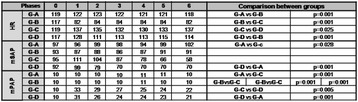
HR, mSAP, mPAP alterations over time in groups & comparison between groups

## P378 Ketamine for analgosedation in severe hypoxic respiratory failure

### S. D. Lee^1^, K. Williams^1^, I. D. Weltes^2^

#### ^1^Royal Liverpool Intensive Care Unit, Liverpool, UK; ^2^University of Liverpool, Liverpool, UK

**Introduction**

Evidence from experimental studies suggests that ketamine may be beneficial for analgosedation in acute respiratory failure due to bronchospasmolytic and immunomodulatory effects. However, limited data exist whether analgosedation with ketamine is safe to use and whether it decreases the requirements for respiratory support.

**Methods**

Over a 6-year period we retrospectively identified patients who received ketamine for hypoxic respiratory failure after admission to critical care. Ketamine dose, mean arterial pressure, heart rate, blood biochemistry, ventilator settings, and sedation score were recorded over the first 5 days of ketamine treatment. Complications (delirium, arrhythmias, malabsorption of feed) were documented. Chest X-ray findings were documented on the day ketamine was commenced.

**Results**

27 patients (11 males, 16 females) were included. Mean age was 54.2 ± 13.4 years. The mean APACHE II score was 14.9 ± 4.1. 37.0 % died while on Intensive Care. The underlying diagnosis of respiratory failure included COPD in 14 and severe asthma in 13 patients. The mean length of stay in critical care was 10.9 ± 8.7 days. All patients were intubated on admission and 26 patients (96.3 %) were receiving controlled ventilation. By Day 5, 17 patients were still ventilated, with 10 patients on controlled ventilation, 8 were weaned to ASB, CPAP, or SIMV. Ketamine was given for 3.9 ± 1.9 days. On day 1 of ketamine treatment the Peak Inspiratory Pressure-max was 30.0 ± 3.4 mmHg and PEEP-max was 8.0 ± 3.7 mmHg. 25 patients received intravenous aminophylline (92.6 %), 20 salbutamol (74.1 %) and 22 MgSO4 (81.5 %). The total dose on days 1 to 5 was 73.9 ± 64.9, 130.7 ± 76.7, 127.1 ± 56.4, 101.0 ± 73.8 and 124.7 ± 84.2 mg. pCO2-max decreased from 15.5 ± 6.1 kPa to 8.8 ± 3.5 kPa, lactate-max decreased from 3.5 ± 2.6 mmol/L to 1.6 ± 1.0 mmol/L, whereas pH-min increased from 7.06 ± 0.15 to 7.34 ± 0.11. Abdominal distension was observed in 5 patients and 13 failed to absorb enteral feeds. Delirium requiring treatment with haloperidol was seen in 5 patients. 14 patients (51.9 %) had X-ray changes consistent with infection and 19 patients (70.4 %) had positive microbiology results within the first 7 days of ketamine administration. 2 patients (7.4 %) developed new onset AF and 22 patients (81.5 %) had episodes of tachycardia.

**Conclusions**

Ketamine is safe for analgosedation in severe hypoxic respiratory failure caused by COPD or asthma. Chest infection was a common trigger. Common complications of critical care (constipation, delayed gastric transport, abdominal distension, delirium, atrial fibrillation) were not more frequently observed than reported for critically ill patients.

## P379 Madness from the moon? lunar cycle and the incidence of delirium on the intensive care unit

### S. Berhane, C. Arrowsmith, C. Peters, S. Robert

#### Homerton University Hospital, London, UK

**Introduction**

Many Health professionals believe that the lunar cycle influences the incidence of psychiatric presentation to hospitals [1,2]. Research has shown that the circa-lunar cycle can influence the objective quality of sleep [3]. However more robust research largely involving the effect of the full moon on emergency psychiatric presentations has failed to find any correlation [4]. The aim of this novel study was to examine whether the incidence of delirium in intensive care patients was influenced by the lunar cycle.

**Methods**

The study was conducted over six months in a general intensive care unit. Data was collected on the day of the full moon and new moon of each month and 24 hours before and after). Delirium was assessed using the confusion assessment method – intensive care (CAM-ICU). Patients who were too sedated to be assessed by CAM- ICU were excluded.

**Results**

Data on 92 patients was collected. There was no significant difference in these baseline characteristics and interventions known to increase the risk of delirium. Sedative drugs used in the two groups were similar apart from the fact that patients in the full moon group received more benzodiazepines (0 % vs. 10 %) and more haloperidol (0 % vs. 12 %).

There was no statistical difference between the incidence of delirium during the new moon (CAM-ICU positive 20 %) and the full moon groups (CAM-ICU positive 14 %).

**Conclusions**

Our study found no association between the incidence of delirium and the full moon in intensive care patients. We hypothesise that the reason for the persistence of the belief that the full moon is associated with increased psychiatric morbidity is due to confirmation bias. This may explain why patients received more sedative drugs during the full moon when compared to the new moon.

**References**

1. Wilson, J.E., Tobacyk, J.J. Lunar phases and crisis center telephone calls. J. Soc. Psychol, vol. 130, 47–51. 1990.

2. C. L. Raison, H. M. Klein, and M. Steckler. “The moon and madness reconsidered,” Journal of Affective Disorders, vol. 53, 99–106, 1999

3. Csilla Zita Turanyi, Katalin Zsuzsanna Ronai, Rezso Zoller. Association between Lunar phase and sleep characteristics. Sleep medicine, Vol. 15, 1411–1416, 2014.

4. Varinder S. Parmar, Ewa Talikowska-Szymczak, Emily Downs et al. Effects of Full-Moon Definition on Psychiatric Emergency Department Presentations. ISRN Emergency medicine. 1-6. 2014.

## P380 Impaired dynamic cerebral autoregulation after coronary artery bypass grafting and association with postoperative delirium

### J. Caldas^1^, R. B. Panerai ^2^, T. G. Robinson ^2^, L. Camara^1^, G. Ferreira^2^, E. Borg-Seng-Shu ^1^, M. De Lima Oliveira ^1^, N. C. Mian^1^, L. Santos ^1^, R. Nogueira^1^, S. P. Zeferino^1^, M. Jacobsen Teixeira^1^, F. Galas^1^, L. A. Hajjar^1^

#### ^1^University of Sao Paulo, Sao Paulo, Brazil; ^2^University of Leicester, Leicester, UK

**Introduction**

Cardiac surgery is associated with a high incidence of neurological complications, including stroke, delirium and cognitive impairment. Involved mechanisms include embolism and reduced brain flow during bypass. There are no data evaluating the impact of cardiac surgery in cerebral autoregulation and its potential relation to neurological outcomes. The aim of this prospective observational single-centre study was to evaluate the impact of cardiac surgery on dynamic cerebral autoregulation (dCA) and assess its potential association with postoperative delirium.

**Methods**

Adult with a EuroSCORE > 6 or left ventricular ejection fraction < 40 % undergoing coronary artery bypass graft (CABG) surgery with bypass were included. Cerebral blood flow velocity (CBFV, transcranial Doppler, middle cerebral artery), end-tidal PaCO2 (EtCO2, infrared capnograph), and blood pressure (BP, Finapres or intra-arterial) were continuously recorded supine during 5 minutes at rest pre-operatively (T1),after 24 h (T2) and 7 days after surgery (T3). Diagnosis of delirium was performed using the confusion assessment method for ICU (CAM-ICU). Autoregulation index (ARI) was estimated from CBFV response to a step change in BP derived by transfer function analysis using standard template curves. Changes in ARI at T1, T2 and T3 were assessed with repeated measures ANOVA.

**Results**

**Fig. 96 (Abstract P380).**
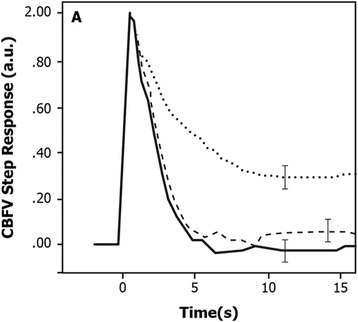
CBFV Step Response in T1(continuous line), T2(dotted line) and T3(dashed line)

**Fig. 97 (Abstract P380).**
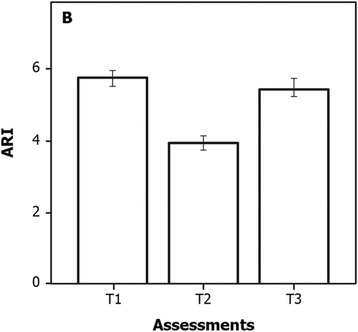
Mean values of the autoregulation index (ARI), assessed in T1, T2 and T3

Thirty-three (23 male) patients, mean age 64.5 (SD10.9) years had data with acceptable quality.CBFV step responses at T2 were markedly different from T1 and T3 (Fig. 96) with corresponding values of ARI (Fig. 97) significantly reduced at T2 (3.9 ± 1.4), compared to T1(5.7 ± 1.4) and T3 (5.4 ± 1.6) (p < 0.001). We defined impaired dCA < 4. Of a total of 33 patients, 19 (57 %) had impaired CA at T2 and 7 patients (21 %) at T3.Delirium was diagnosed in 8 patients; of these, 6 (31 %) had impaired dCA, compared to 2 (14 %) with normal dCA (Fisher p = 0.31). At T3, 71 % of patients with impaired dCA had delirium compared to 8 % with normal dCA (Fisher p = 0.0175).

**Conclusions**

Dynamic cerebral autoregulation is impaired after CABG surgery and possibly associated with occurrence of postoperative delirium. Further work is needed to elucidate the underlying mechanisms of dCA and to establish the adequate therapy.

## P381 Risk factors predicting prolonged intensive care unit length of stay after major elective surgery.

### P. Killeen, M. McPhail, W. Bernal, J. Maggs, J. Wendon, T. Hughes

#### King’s College Hospital, London, UK

**Introduction:** This study investigated risk factors for prolonged intensive care unit (ICU) length of stay (LOS) in an elective surgical patient cohort. LOS is an important outcome as a marker of ICU resource consumption, and there is increasing pressure to use beds more efficiently. If we can estimate the common LOS for common ICU patient groups, such as elective procedure patients, and identify risk factors for prolonged LOS in these groups, then beds can be booked in a more prospective way. This could help improve the flow efficiency of these patients through intensive care beds.

**Methods:** Retrospective cohort study of 1288 elective surgical patients who stayed on an ICU at King’s College Hospital between the period 1/4/2014 to 31/3/2015. Data was collected for patients including pre-operative and immediate (within 6 hours) post-operative physiology and comorbidities. A Charlson comorbidity index (CCI) was calculated for each patient using an ICD-10 specific algorithm. The main outcome, prolonged LOS, was defined as an LOS greater than or equal to the 75th percentile. Multivariate logistic regression was used to identify variables significantly associated with prolonged length of stay.

**Results:** With multivariate logistic regression, patients with prolonged LOS were characterised by renal disease (OR 5.564 CI 95 % 1.166-26.546 p = 0.031), high post-operative sodium (OR 1.342 CI 95 % 1.115-1.615 p = 0.002), high pre-operative c-reactive protein (OR 1.036 CI 95 % 1.003-1.070 p = 0.033) and low post-operative c-reactive protein (OR 0.963 CI 95 % 0.929-0.998 p = 0.041), with a c-index of 0.756 (CI 95 % 0.655-0.856 p < 0.0004). Analysing the univariate relationship between each factor and LOS additionally identified the presence of anaemia, malignancy, low albumin and high CCI as significantly associated with a prolonged stay, but these were not validated as significant at the multivariate stage. Age, chronic pulmonary disease and heart failure were not found to be significantly associated with an increased LOS in our cohort.

**Conclusions:** Prolonged LOS is significantly associated with several measurable patient characteristics. Further work would be required to more accurately characterise risk factors, but validation of similar risk factors could facilitate prospective bed booking in a dedicated post anaesthetic care unit, with those patients who have risk factors for prolonged LOS instead being admitted directly to the general ICU post-operatively.

## P382 Systemic inflammatory response syndrome criteria and hospital mortality prediction in a brazilian cohort of critically ill patients

### L. U. Taniguchi, E. M. Siqueira, J. M. Vieira Jr, L. C. Azevedo

#### Research and Education Institute, Sao Paulo, Brazil

**Introduction:** Introduction: Criteria for Systemic Inflammatory Response Syndrome (SIRS) are frequently used to identify patients at higher risk of death. However, the utility of SIRS has recently been questioned. The objective of this study was to evaluate if SIRS criteria might predict hospital mortality in a Brazilian cohort of critically ill patients.

**Methods:** Methods: This is a retrospective cohort study conducted at a private tertiary hospital (Hospital Sirio-Libanes) at São Paulo, Brazil. We extracted relevant information from the adult intensive care unit database (sistema Epimed™). A comparison was performed between SAPS 3 score model and SIRS model both as a dichotomous variable (> = 2 criteria - “SIRS positive” versus 0-1 criterion - “SIRS negative”) and as an ordinal variable from 0 to 4 (according to the number of SIRS criteria met) for predicting hospital mortality. Models discriminations were compared using area under the ROC curves.

**Results:** Results: Between January to December 2012, we studied 932 patients (60.4 % were classified as “SIRS positive”). Patients “SIRS positive” were more severily ill compared to patients “SIRS negative” (SAPS 3 of 45 [34 – 56] versus 39 [31 – 50] respectively; p < 0.001) and had higher hospital mortality rates (16.9 % vs 8.1 % respectively; p < 0.001). However, area under ROC curve for SAPS 3 was higher (0.81 [95 % CI 0.78 – 0.85]) than for SIRS criteria both as a dichotomous variable (0.60 [95 % IC 0.55 – 0.65]) as an ordinal variable (0.62 [95 % IC 0.57 – 0.68]; p < 0.001 for comparisons between SAPS 3 and SIRS criteria).

**Conclusions:** Conclusion: The utility of SIRS criteria to recognize severity of illness and predict mortality in our cohort could not be observed.

## P383 Evaluating the efficacy of a risk predictor panel in identifying patients at elevated risk of morbidity following emergency admission

### A. N. Ahmad, M. Abu-Habsa, R. Bahl, E. Helme, S. Hadfield, R. Loveridge

#### King’s College Hospital, London, UK

**Introduction:** This study aims to evaluate the efficacy of pre-defined clinical and biochemical criteria in predicting deterioration following hospital admission via the Emergency Department (ED), and to evaluate their utility as triggers for involvement of the iMobile Critical Care Outreach service within the hospital.

**Methods:** 113 patients were randomly selected from King’s College Hospital emergency admissions. Notes were screened for the presence of: a National Early Warning Score (NEWS) > =5 or respiratory rate > =24 at transfer from the ED to the ward, sepsis with hypotension at any point, new acute kidney injury (AKI), lactate > =4 or metabolic acidosis on blood gas analysis and need for high flow oxygen at transfer from the ED to the ward. Primary outcome measures were mortality, Intensive Care Unit (ICU) admission, and length of stay. Associations between proposed criteria and patient outcomes were statistically evaluated.

**Results:** Results calculated based on 14-day outcome data suggest that likelihood of ICU admission is significantly increased by NEWS > =5 (chi-squared = 5.029, p = 0.025), new AKI (chi-squared = 9.099, p = 0.003) and need for high flow oxygen at transfer (chi-squared = 16.634, p < 0.0001). Patient mortality was significantly increased by NEWS > =5 (chi-squared = 6.391, p = 0.011), respiratory rate > =24 (chi-squared = 5.417, p = 0.020), need for high flow oxygen at transfer (chi-squared = 19.394, p < 0.00001), detection of metabolic acidosis on blood gas analysis (chi-squared = 4.764, p = 0.030) and new AKI (chi-squared = 5.056, p = 0.025). Additionally, new AKI significantly increases length of stay (+5.785 days, p = 0.003). Patients will be followed up for a period of 3 months and current preliminary results will be reassessed with 3-month outcome data in this ongoing study.

**Conclusions:** NEWS > =5, new AKI, elevated respiratory rate, metabolic acidosis and a high inspired oxygen fraction requirement are significant predictors of inpatient stay morbidity and could potentially be used as triggers for early Critical Care input. A study based on a larger patient sample is underway.

## P384 A retrospective comparison of outcomes for elective surgical patients admitted post-operatively to the critical care unit or general ward

### J. Shak, C. Senver, R. Howard-Griffin

#### Ipswich Hospital NHS Trust, England, UK, Ipswich, UK

**Introduction:** In 2014, admissions following elective surgery occupied 26 % of admissions to the Critical Care Unit (CCU) at Ipswich Hospital. A retrospective study showed 75 % of these admissions did not result in Level 2 or 3 intervention.

Depending on CCU bed pressures, patients booked for elective post-operative admission are assessed in the recovery room for suitability to return to a general ward. The purpose of this study is to compare the outcomes of all patients who were identified as high risk, thus being booked for post-operative CCU admission, thereby identifying those who do benefit from CCU admission and those safe for care on the ward.

**Methods:** A retrospective analysis of all CCU bed requests was undertaken between November 2013 - April 2014. Patient demographics, comorbidities, nature of surgery and post-operative progress were recorded. A comparison was made between those admitted to CCU and those returning to a general ward.

**Results:** Of 149 CCU bed requests made, 100 cases were admitted to CCU, 49 cases returned to the ward following anaesthetist or intensivist assessment. There was no significant difference in age between the two groups (mean 72.9 vs 75.6 years, p = 0.16). There was no significant difference in comorbid state, quantified using the Charlson Comorbidity Index (mean score 2.55 vs 2.31, p = 0.24). Of all surgical specialities, colorectal patients were most likely to be admitted to CCU.

Overall length of stay was significantly longer in those admitted to CCU compared to ward, (mean 11 vs 5 days, p = 0.00006). There were also more post-operative complications; 64 events in 42 patients vs 12 events in 11 patients. 6 of 100 patients admitted to CCU required subsequent readmission. 1 of 49 patients returned to the ward was admitted to CCU day 1 post-operatively. Thus, only 26 of 149 CCU bed requests resulted in admission with Level 2 or 3 interventions.

**Conclusions:** This study suggests that patient demographics and comorbid state have little influence on the eventual post-operative care pathway in this patient cohort. Rather, an objective assessment in the recovery room effectively identified patients with a shorter length of stay and fewer post-operative complications.

We recommend routine assessment of high risk patients in the recovery room to determine suitability for admission to CCU. We have introduced a proforma outlining peri-operative risk factors to allow consistent assessment of suitability for ward-based care.

## P385 Effect of obesity on mortality in surgical critically ill patients

### P. Wacharasint^1^, P. Fuengfoo^1^, N. Sukcharoen^1^, R. Rangsin^2^

#### ^1^Phramongkutklao Hospital, Bangkok, Thailand; ^2^Phramongkutklao College of Medicine, Bangkok, Thailand

**Introduction:** Obesity is one of the major risk factor for a number of chronic diseases including diabetes, cardiovascular diseases and cancer. While obesity itself is a chronic inflammatory condition, previous studies showed that the obese patient with severe infection was found associated with significantly lower mortality compared to the septic patients with normal BMI [1]. In surgical critically ill patients whether obesity confers a “protective” effect is not clearly defined. We hypothesized that surgical critically ill patients, which their disease pathologies differ from septic medical patients, may also have “protective” effect from obesity.

**Methods:** We conducted a retrospective cohort analysis using the THAI-SICU study databases, which recruited 4652 Thai patients who admitted to the surgical intensive care units of nine university-based hospitals in Thailand [2]. All 4579 patients whose body mass index (BMI) and mortality at 28 days were available, were included in this analysis. The patients were categorized into 4 groups based on their BMI i.e., underweight (BMI <18.5), normal BMI (BMI 18.5-24.9), overweight (BMI 25-29.9), and obesity (BMI >30). Primary outcome was 28-day mortality, and secondary outcome was the incidence of systemic inflammatory response syndrome (SIRS).

**Results:** Of 4579 patients, 768 (16.8 %), 2624 (57.3 %), 858 (18.7 %), and 329 (7.2 %) patients were classified as underweight, normal BMI, overweight, and obese, respectively. Obese patients had the lowest APACHE II score (8, interquartile range (IQR) 5-12), followed by overweight (10, IQR 7-15), normal BMI (10, IQR 7-16), and underweight (12, IQR 8-16) (p < 0.001). Among these four patient groups, obese patients had the lowest 28-day mortality (6.7 %), followed by overweight (9.6 %), normal BMI (14.7 %), and underweight patient groups (18.5 %) (p < 0.001). Obese patients also had the lowest incidence of SIRS (29.5 %), followed by overweight (34.5 %), normal BMI (35.6 %), and underweight patients (41.1 %) (p = 0.001). In multivariate analysis, we found that every one unit increasing of BMI associated with lower risk of hospital mortality, adjusted odds ratio = 0.96 (95%CI: 0.94-0.98, p < 0.0001) after adjusted for age, gender, and APACHE II severity score.

**Conclusions:** We demonstrated that, compared to surgical critically ill patients with lower BMI, surgical patients with higher BMI had significantly lower risk of mortality.

**References**

1. Wacharasint et al. Crit Care 2013;17:R122.

2. Chittawatanarat K et al. J Med Assoc Thai 2014;97(Suppl1):S45-54.

## P386 The national early warning score (news) reliably improves adverse clinical outcome prediction in community-acquired pneumonia - results from a 6 year follow-up

### D. Sbiti-Rohr, P. Schuetz

#### Kantonsspital Aarau, Aarau, Switzerland

**Introduction:** Clinical scores such as pneumonia severity index (PSI) and CURB-65 are recommended for risk assessment in community-acquired pneumonia (CAP). We investigated the prognostic potential of the National Early Warning Score (NEWS) in a large and well defined cohort of CAP patients with a 6 years follow-up.

**Methods:** We prospectively followed 925 CAP patients from a previous Swiss multicentre trial. Our primary outcome was all-cause mortality within 6 years of follow-up. Secondary outcomes were adverse clinical outcome defined as intensive care unit (ICU) admission, complications (empyema) and unplanned hospital readmission all within 30 days. We used logistic regression models and area under the receiver operating characteristics curve (AUC) to compare NEWS with PSI and CURB-65.

**Results:** Six-year overall mortality was 45.1 % (n = 417) with a step-wise increase with higher NEWS categories. For 30-days and 6-year mortality prediction, NEWS showed only moderate discrimination (AUC 0.65 and 0.60) inferior compared to PSI and CURB-65. For prediction of ICU admission, NEWS showed high discrimination (AUC 0.73) and improved the prognostic accuracy of PSI (AUC from 0.66 to 0.74, p = 0.001) and CURB-65 (AUC from 0.64 to 0.73, p = 0.015). NEWS was also superior to PSI and CURB-65 for prediction of complications, but did not well predict rehospitalisation.

**Conclusions:** NEWS provides additional prognostic information in regard to risk of ICU admission and complications thereby improves traditional clinical risk scores in the management of CAP patients in the emergency department setting.

## P387 Clinical usefulness of the charlson¡¯s weighted index of comorbidities _as prognostic factor in patients with prolonged acute mechanical ventilation

### H. Na, S. Song, S. Lee, E. Jeong, K. Lee

#### Pusan National University Hospital, Busan, South Korea

**Introduction:** Concurrent comorbidities increase the risk of severe infections and greatly influence the patient outcome. Our study evaluated the clinical utility of Charlson¡¯s weighted index of comorbities (WIC) as a prognostic factor in patients with prolonged acute mechanical ventilation (PAMV, ventilator care ¡Ã 96 hrs).

**Methods:** We retrospectively enrolled 311 PAMV patients who were admitted to the mechanical intensive care unit (ICU) of Pusan National University Hospital between 2008 and 2013. Survivors were defined as patients who survived for 28 days after their ICU admission.

**Results:** The median age of the subjects was 67 years (range: 18-93), and 71.7 % of them were male. The median lengths of their stay in the ICU and the hospital were 15 (range: 4-129) and 26 (range: 5-430) days, respectively. The ICU and hospital mortality rates were 43.7 and 44.4 %, respectively, and the 28-day mortality rate after the ICU admission was 34.7 %. The median WIC was 2 (range: 0-11), and there was a significant difference in the WICs of the non-survivors and survivors (p < 0.05). The linear regression analysis revealed a significant positive relationship between the WIC and the Acute Physiology and Chronic Health Evaluation II and Sequential Organ Failure Assessment scores on the admission day (p < 0.05). The receiver operating characteristics curve indicated a WIC of 3 as the cut-off value [area under the curve, 0.593; 95 % confidence interval (CI), 0.525-0.661; and p < 0.05], and the subjects with a WIC greater than 3 had a higher mortality rate (44.5 % vs. 28.6 %, p < 0.05). The univariate logistic regression analysis showed that a WIC ¡Ã 3 was independently associated with 28-day mortality in our population (odds ratio, 2.0; 95 % CI, 1.240-3.226; and p < 0.05).

**Conclusions:** In our study, WIC was the useful prognostic indicator for 28-day mortality in patients with PAMV.

## P388 Comparison of mortality prediction scoring systems in patients with cirrhosis admitted to general intensive care unit

### M. Cooper^1^, K. Milinis^2^, G. Williams^2^, E. McCarron^1^, S. Simants^1^, I. Patanwala^1^, I. D. Welters^2^

#### ^1^Royal Liverpool University Hospital, Liverpool, UK; ^2^University of Liverpool, Liverpool, UK

**Introduction:** Mortality of patients with cirrhosis admitted to the intensive care unit (ICU) has been reported to be as high as 60 %. Recently, a number of scoring systems have been developed in order to predict mortality in tertiary liver units, including CLIF-SOFA-based scoring systems and Royal Free Hospital (RFH) tool, however, none of them has been validated in patients admitted to general ICU [1]. The aim of the study is to validate the new measures and compare with established prediction models.

**Methods:** Data on patients with confirmed cirrhosis admitted to a general ICU at a major university hospital between 2008 and 2014 were retrospectively collected from medical records. Scores were calculated for CLIF-Consortium Organ Failure score (CLIF-C OF, a shortened version of CLIF-SOFA), CLIF-C ACLF (includes age and white cell count), RFH, Model for End-Stage Liver Disease (MELD) and Acute Physiology and Chronic Health Evaluation (APACHE II). The main outcome was mortality in ICU. Predictive accuracy and calibration was assessed using area under the receiver operator curve (AUROC) and goodness-of-fit χ statistic.

**Results:** 118 patients were identified (mean age 50.6 years, 66.9 % male). ICU mortality was 54.2 %. Alcoholic liver disease was the main aetiological factor present in 91.5 % of the patients. 56 % of patients were admitted to ICU with gastrointestinal bleeding. All measures were well calibrated (χ >0.2, p > 0.05), particularly CLIF-OF (χ =1.3, p = 0.93). Accuracy of the novel models was generally not superior to the established scores (AUROC values: CLIF-OF 0.68; CLIF-ACLF 0.68; RFH 0.72 vs. APACHEII 0.65; MELD 0.71). Bilirubin, creatinine and platelets were found to be independent predictors of mortality in a multivariate logistic model. The accuracy of creatinine alone was higher than any of the prediction models (AUROC 0.85, χ =8.4, p = 0.35).

**Conclusions:** RHF and MELD scores have the best predictive accuracy. However, complex and specialised measures do not offer advantage in predicting mortality over MELD and routinely available APACHEII score in cirrhotic patients admitted to general ICU. Creatinine alone is a robust prognostic indicator of mortality. Simplified scores encompassing few variables may offer similar accuracy, but higher utility.

**Reference**

[1] E. Theocharidou et al.: Am. J. Gastroenterol., vol. 109, no. 4, pp. 554–562, 2014.

## P389 Impact of admission source and time of admission on outcome of pediatric intensive care patients: retrospective 15 years study

### E. Zoumpelouli, EA Volakli , V. Chrysohoidou, S. Georgiou, K. Charisopoulou, E. Kotzapanagiotou, V. Panagiotidou, K. Manavidou, Z. Stathi, M. Sdougka

#### PICU, Hippokration General Hospital, Thessaloniki, Greece

**Introduction:** Admission source and time of admission could play a role on the outcome of Pediatric Intensive Care Unit (PICU) patients (pts). The aim of the present study is to investigate admission characteristics in a Greek multidisciplinary 8 bed PICU and their relationship with mortality at discharge.

**Methods:** Retrospective 15 years study (1/6/1999-31/12/2014). Data collected: Demographics, reason for admission, emergency admissions, readmissions, co-morbidities, source of admission (internal pediatric, internal surgical, other in city hospital, remote peripheral hospital), season of admission (spring, summer, autumn, winter), time of admission (in or out-of-hours), length of stay (LOS) and the outcome. Statistics: t-test for independent samples, chi-square test, p < 0.05.

**Results:** 1977 pts, 57.5 % male, aged 55.51 ± 50.28 mo (mean ± SD, median 40) were recorded. The main reasons for admission were respiratory failure (26 %), postoperative care (15.7 %), seisures (11.5 %), head trauma (11.2 %), coma (8 %), polytrauma (6.5 %), sepsis (4.7 %), cardiovascular (4.5 %), accidents (3.9 %), MODS (3.7 %), miscellaneous (2.9 %) and metabolic (1.5 %). LOS was 11.35 ± 26.64 days (median 5) and mortality at discharge 14.36 %. Age (p = 0,742), infancy (p = 0.204), readmissions (p = 0.793), co-morbidities (p = 0.969), season of admission (p = 0.560) and time of admission (p = 0.353) did not play a role on the mortality. On the contrary, mortality was significantly related to sex (male 12.1 % vs. female 17.6 %, p = 0.003), emergency admissions (16.7 % vs. 3.1 %, p = 0.000), source of admission (p = 0.000), and reason for admission (p = 0.003). Internal pediatric pts had the highest mortality (25.7 %), followed by other in city hospital (11.5 %), remote peripheral hospital (12.2 %) and internal surgical (8.6 %). Moreover, outcome was worse for MODS (mortality 65.3 %), cardiovascular (43.2 %), sepsis (27.5 %), coma (24.2 %) and metabolic (21.4 %) pts. Non survivors stayed significantly longer (16.38 ± 36.52 vs. 9.97 ± 13.92 days, p = 0.000).

**Conclusions:** Our results are in accordance with data that attribute higher mortality in internal pts, which is partially expected in tertiary care hospitals like ours. Season and time of admission did not have a significant impact on the outcome, indicating a high level of care even under suboptimal conditions where resources are restricted, as happened during holidays and out-of-hours admissions.

## P390 Heart rate variability and outcomes prediction in critical illness

### N. Salahuddin, B. AlGhamdi, Q. Marashly, K. Zaza, M. Sharshir, M. Khurshid, Z. Ali, M. Malgapo, M. Jamil, A. Shafquat, M. Shoukri, M. Hijazi

#### King Faisal Specialist Hospital & Research Centre, Riyadh, Saudi Arabia

**Introduction:** Heart Rate Variability (HRV) is an indicator of the dynamic equilibrium between the sympathetic and parasympathetic divisions of the autonomic nervous system. We hypothesized that baseline HRV variables and changes during resuscitation, may predict outcomes from critical illness.

**Methods:** A prospective, observational study was performed on inpatients that required a Rapid Response Team (RRT) consultation. 24-hour holter monitoring and serial measurements of physiological and biochemical data were made. Heart rate variability was measured as time domains measured over 24 hours (SDNN, ASDNN, rMSSD, pNN50%, SDANN, mean NN) and frequency domains measured hourly (Very Low Frequency VLF, Low Frequency LF, High Frequency HF, Low/High ratio). The Research Ethics Committee approved the study protocol (RAC No. 2151069).

**Results:** Fifty-three patients were enrolled, mean APACHE II score was 23.5 ± 6.3, age 52 ± 24.3 years. Day 1 SOFA score was 8.9 (range 1, 23). Forty patients (75.5 %) required ICU admission; ICU mortality rate was 27.5 %.

HRV was significantly higher in RRT consultations who stabilized and did not require ICU admission; time domains; ASDNN [33(IQR21) vs 18(IQR21), p = 0.024], rMSDD [23(IQR19) vs 15(IQR18), p = 0.036] and frequency domains; meanVLF [16.6(IQR7.3) vs 9.3(IQR10), p = 0.018], meanLF [12.4(IQR11) vs 5.4(IQR7), p = 0.009], meanHF [9.3(IQR12) vs 4.8(IQR7), p = 0.011].

Baseline HRV was significantly higher in survivors; ASDNN [31.5(IQR24) vs 12(IQR9),p = 0.002],rMSDD [25(IQR19) vs 11.5(IQR10), p = 0.012], pNN50% [6(IQR9.5) vs 0.75(IQR2.5),p = 0.002], meanNN[732.5(IQR291) vs 570(IQR87),p = 0.006], mean VLF [12.1(IQR11.8) vs 5.3(IQR4), p = 0.002], meanLF [8.5(IQR10.2) vs 3.4(IQR4.6), p = 0.009], meanHF [7.5(IQR6) vs 3.3(IQR3.9), p = 0.005].

Survivors also demonstrated a significantly larger increase in HRV over 24 hours of resuscitation; deltaVLF [3(IQR8.1) vs -0.6(IQR8), p = 0.015], deltaLF [3.2(IQR5.9) vs -0.3(IQR7.6), p = 0.017].

**Conclusions:** HRV analysis appears to be a powerful identifier of outcomes in critical illness. Baseline values and changes over the first 24 hours of resuscitation accurately predicted both the need for ICU admission and survival.

## P391 The incidence and outcome of hyperlactatemia in the post anaesthesia care unit

### T. Abe, S. Uchino, M. Takinami

#### The Jikei University School of Medicine, Tokyo, Japan

**Introduction:** Critically ill patients often develop hyperlactatemia, and elevated serum lactate levels are associated with increased hospital mortality. However, the epidemiology of hyperlactatemia in patients admitted to the post anaesthesia care unit (PACU) has never been studied previously.

**Methods:** This retrospective observational study was conducted in the PACU of a university hospital in Tokyo, Japan from 1 January 2010 to 31 December 2014. Adult PACU patients who were monitored with the arterial catheter after elective surgery were included, except for patients with no lactate measured within 3 hours after PACU admission. We measured serum lactate levels in arterial blood samples using a point-of-care device. Study patients were divided to three groups according to maximum lactate levels in the first 24 hours of the PACU stay; normal lactate (<2 mmol/L), moderate hyperlactatemia (2-3.9 mmol/L) and severe hyperlactatemia (> = 4 mmol/L). The primary outcome was the composite outcome consisted of in-hospital mortality, re-admission to the PACU or admission to the ICU, and emergency reoperation during the same hospital stay.

**Results:** A total of 5362 patients were included in the study. Among these patients, 3421 (63.8 %) patients had normal lactate, 1642 (30.6 %) had moderate and 299 (5.6 %) had severe hyperlactatemia. The composite outcome occurred in 333 (6.2 %) of all patients. The incidence rate of the composite outcome increased as serum lactate levels elevated (normal: 4.7 %, moderate: 7.9 %, severe: 14.1 %, normal vs. moderate: [i]p[/i] <0.001, moderate vs. severe: [i]p[/i] <0.001). In-hospital mortality, re-admission to the PACU or admission to the ICU, and emergency reoperation occurred in 71 (1.3 %), 190 (3.5 %), and 168 (3.1 %) for all patients, respectively. Each primary outcome tended to increase with elevated serum lactate levels. Multivariable logistic regression analysis revealed that moderate and severe hyperlactatemia were risk factors for the composite outcome, odds ratio: 1.56 (1.20 - 2.01) for moderate hyperlactatemia, 1.57 (1.01 - 2.43) for severe hyperlactatemia referred to normal lactate.

**Conclusions:** The incidence of hyperlactatemia in the PACU was 36.2 % after elective surgery. Although hyperlactatemia was independently associated with poor outcome, severe hyperlactatemia was only similarly associated with poor outcome compared with moderate hyperlactatemia. Further studies, especially for causes of hyperlactatemia in the PACU, will be clarified.

## P392 Correlation between arterial blood gas disturbances and arterial lactate levels during hospitalization and outcome in critically septic patients

### N. R. Rangel Neto, S. Oliveira, F. Q. Reis, F. A. Rocha

#### Albert Schweitzer State Hospital, Rio de Janeiro, Brazil

**Introduction:** Severe sepsis is a life-threatening multisystem process that can result in significant morbidity or mortality. In most patients, severe sepsis and shock septic is preceded by a period of physiological deterioration; but evidence suggests that the early signs of this are frequently missed. An arterial blood gas (ABG) analysis is indispensable tool to assess and monitor critically ill patients in the ICU or other critical care settings.

**Methods:** In this cross-sectional study, the medical data of all critically septic patients admitted to the Albert Schweitzer Hospital were reviewed between January 2013 and September 2015. Data including age, gender, SAPS III score upon admission, SOFA score, arrival and 24 hour ABG and arterial lactate levels, as well as length of stay and in-hospital mortality rate were collected. A logistic regression model was used to determine the association between acid-base disturbance and lactate levels with in-hospital mortality after adjustment for potential confounding factors.

**Results:** Of the 2974 patients, 54 % were male; mean age of 62 years old, mean SAPS III score of 63 points, SOFA score of 9 and 37,9 % of them died in the hospital. Univariate analysis showed a significantly higher risk of mortality in patients who developed metabolic acidosis plus hyperlactatemia (OR = 2,98, p-value 0.01). Multiple logistic regression analysis demonstrated that mixed metabolic acidosis plus respiratory acidosis (OR = 3.19, 95 % CI = 1.48-9.32, p-value = 0.001) and hyperlactatemia (OR = 1.78, 95 % CI = 0.49-4.56, p-value 0.02), patients with acidoses had very higher mortality (pH less then 7,2 44,1 % died and pH less then 7,05 95,5 % died; p 0.01 and p < 0,001 respectively), patients with hyperlactatemia had higher mortality (arterial lactate over 2,5x regular levels 23,2,% died and arterial lactate over 5x regular levels 63,4,% died; p 0.05 and p 0,01 respectively) were two significant predictors of mortality, regardless of other confounding variables.

**Conclusions:** The results show that in critically septic patients initial metabolic acidosis and hyperlactatemia are significant predictors of mortality, reinforcing the significance of early action to restore tissue perfusion and minimizing damage.

**Reference**

Lactate, pH, and blood gas analysis in critically ill patients. Waldau T ET AL Acta Anaesthesiol Scand Suppl. 1995 ;107:267-71.

## P393 External validation of saps 3 and mpm iii scores in 48,816 patients from 72 brazilian icus

### G. Moralez^1^, K. Ebecken^1^, L. S. Rabello^1^, M. F. Lima^2^, R. Hatum^3^, F. V. De Marco^4^, A. Alves^5^, J. E. Pinto^6^, M. Godoy^7^, P. E. Brasil^8^, F. A. Bozza^8^, J. I. Salluh^8^, M. Soares ^8^

#### ^1^PPG Internal Medicine, Federal University of Rio de Janeiro, Rio de Janeiro, Brazil; ^2^Hospital Esperanca Recife, Recife, Brazil; ^3^Hospital Total Cor, Rio de Janeiro, Brazil; ^4^Hospital viValle, São José dos Campos, Brazil; ^5^Hospital Rios DOr, Rio de Janeiro, Brazil; ^6^Hospital Norte DOr, Rio de Janeiro, Brazil; ^7^Hospital Esperanca Olinda, Olinda, Brazil; ^8^DOr Institute for Research and Education - IDOR, Rio De Janeiro , Brazil

**Introduction:** To validate the SAPS 3 and MPM-III scores in a large contemporary cohort of patients admitted to Brazilian ICUs.

**Methods:** Retrospective multicenter cohort study in 48,816 adult patients consecutively admitted to 72 ICUs in 51 hospitals during 2013. We excluded patients who underwent cardiac surgeries or invasive cardiac procedures, readmissions, and those admitted for acute coronary syndromes and burns. We retrieved clinical and outcome data from an electronic ICU quality registry (Epimed Monitor System), including the estimates for the MPM-III and SAPS 3 scores. For the SAPS 3 score, we estimated predicted mortality using both standard (SE) and Latin-American (LA) customized equations. We evaluated models’ discrimination using the area under the receiver operating characteristic curve (AUROC). We applied the calibration belt to evaluate the agreement between observed and expected mortality rates (calibration). We also calculated standardized mortality ratios (SMR) for each model.

**Results:** Median age was 65 (48-79) years. Main reasons for ICU admission were postoperative care (26 %), sepsis (22 %), cardiovascular (11 %) and neurological (11 %) complications. Mechanical ventilation was used in 16 % of admitted patients, vasopressors in 13 %, and dialysis in 3 %. ICU and hospital mortality rates were 11 % and 17 %. Mean SAPS 3 score was 44 ± 15 points. Predicted mortality rates were 16 ± 19 % (SAPS 3 SE), 22 ± 23 % (SAPS LA) and 14 ± 14 % (MPM-III). SMRs were 1.00 (95 % CI, 0.98-.102) for the SAPS 3 SE, 0.76 (0.74-0.77) for the SAPS 3 LA and 1.15 (1.13-1.18) for the MPM-III. Discrimination was better for SAPS 3 models (AUROC = 0.85) than for MPM-III (AUROC = 0.80) (p < 0.001). In the calibration belt analyses, the SAPS 3 LA overestimated mortality throughout all risk classes while the MPM-III underestimated it uniformly. The SAPS 3 SE did not show relevant deviations from ideal calibration.

**Conclusions:** In a contemporary large cohort, the SAPS 3 SE was accurate in predicting outcomes for Brazilian patients.

**References**

Funded by IDOR, CNPq and FAPERJ. Endorsed by BRICNet.

## P394 The frailty penalty: pre-admission functional status confounds mortality prediction models in critically ill patients

### J. Krinsley, G. Kang

#### Stamford Hospital, Stamford, USA

**Introduction:** While previous studies have assessed the relationship of preadmission functional status (FS) to mortality in critically ill patients, none has assessed the impact of FS on the results of mortality prediction models (MPM). We hypothesized that FS would confound predictions of severity adjusted mortality.

**Methods:** This is a retrospective review of prospectively collected data, and includes all 10,149 patients admitted to the 16 bed intensive care unit of our university-affiliated hospital between 10/1/05 and 9/30/15. We abstracted information from the ICU’s database, including diagnoses, APACHE II scores (APII), APACHE IV predictions of mortality (APIVM) and hospital discharge status. We prospectively characterized patients as FS1 (fully independent, n = 8,146), FS2 (partly dependent, living at home, n = 1,026) and FS3 (not living at home, n = 977). We stratified by severity of illness, compared observed:expected mortality ratios (O:E) and created a multivariable model to assess the independent effect of FS on mortality.

**Results:** FS1 were younger than FS2 and FS3, were more likely to be admitted to the ICU after surgery and had lower APII, APIVM and mortality than did FS2 or FS3 (p < 0.0001 all comparisons).

The impact of FS on confounding predictions of mortality was inversely related to severity of illness:

O:E mortality ratios, based on hospital discharge status and APIVM and stratified by severity of illness for FS1, FS2, FS3:

APIVM <10 % - 0.33, 0.72, 1.02

APIVM 10-25 % - 0.49, 1.03, 0.88

APIVM 25-50 % - 0.60, 0.76, 0.98

APIVM >50 % - 0.87, 0.93, 0.94

Multivariable analysis demonstrated that FS2 and FS3 were independently associated with increased risk of mortality when compared to FS1: OR (95 % CI) 1.93 (1.57-2.37) and 2.29 (1.87-2.79), respectively, (p < 0.0001 for both comparisons).

**Conclusions:** Preadmission functional status is independently associated with risk of mortality in critically ill patients and confounds the results of mortality prediction models, especially in patients with less severe illness. Future mortality prediction models should incorporate functional status in their design.

## P395 ‘sooner rather than later’: how delayed discharge from critical care leads to increased out of hours discharges and subsequent increase in in-hospital mortality.

### J. Perry^1^, H. Hines^2^

#### ^1^Darent Valley Hospital, Dartford, UK; ^2^William Harvey Hospital, Ashford, UK

**Introduction:** Studies have identified an increase in mortality and critical care readmissions in those patients discharged from critical care out of hours (22:00-06:59) [1].

**Methods:** Wardwatcher© provided mortality and readmission data of patients discharged from two district hospital ITU’s in a year. Discharges were split into night, weekday and weekend. Chi squared was used to calculate the significance of mortality data. Rate of delayed discharge (>240 minutes) and reason for delay were recorded.

**Results:** In hospital 1, 15 % were night discharges. 65 % weekday and 20 % weekend. Night discharge mortality was 9 %, 8 % weekday and 7 % weekends. 84 % of weekend discharges were delayed, 77 % at night and 70 % during the week. In hospital 2 6.7 % were night discharges. 74 % weekdays and 19 % weekends. Night discharge mortality was 18 %, 9 % on weekdays and 8 % at weekends. 68 % of daytime discharges were delayed, 53 % at weekends and 62 % at night. The combined mortality data (Table 49) has a p value of 0.089, using Chi-squared. In all groups >50 % of discharges were delayed over four hours. Across both sites, >80 % of delays were due to a lack of ward beds. Readmission rates were <5 %.

**Conclusions:** An increase in mortality for night discharges was noted. Hospital 1 had a higher rate of night discharges and a higher rate of delayed discharges. Hospital 2 has a lower rate of both. Possibly demonstrating that prompt discharge of patients leads to fewer night discharges. With the increase in mortality in those discharged at night, it highlights the importance of avoiding delayed discharges. Although a causal relationship between delayed discharge, night discharge and mortality cannot be proved; this data adds to the evidence. Demonstrating a higher mortality associated with night discharges and that delaying discharges contributes to a higher rate of night discharges. More research is required to explore this.

**Reference**

1. Edie et al. Critical care 19:564, 2015

**Table 49 (Abstract P395).** Combined mortality data

Discharge time	Total number of patients	In-hospital mortality
Night	123	13%
Weekday	849	8%
Weekend	233	8%

## P396 Identifying poor outcome patient groups in a resource-constrained critical care unit

### K. M. Wilkinson^1^, C. Tordoff^2^, B. Sloan^2^, M. C. Bellamy^2^

#### ^1^St James’s University Hospital, Leeds, UK; ^2^St James’s University Hospital, Leeds, UK

**Introduction:** Our objective was to identify factors associated with high mortality on admission to a mixed ICU in a resource-constrained environment, and to examine the impact of high-risk admissions. Currently we admit around 1200 patients per year to our mixed general university hospital ICU. Our predicted mortality is higher than the national average (ICNARC).

**Methods:** This was a retrospective review of 6570 consecutive admissions from 2009-14. We analysed our existing ICNARC dataset, together with referring specialty, lead time bias and CPR prior to admission. Logistic regression was used to describe predicted hospital mortality using age, Apache II score, referring specialty and pre-admission CPR. The subset admitted post-CPR were further analysed. These models were then fit-tested using ROC curves. Cost of ICU stay was estimated using mean duration of stay and our current mean ICU cost.

**Results:** Mean age was 61 (range 16-95), median APACHE II score was 20 (IQR 15-26). Median time between hospital and ICU admission was 2 days (IQR 2-5), ICU LOS 2.1 days (1-4.9), post-ICU LOS 8 days (1-19).

**Fig. 98 (Abstract P396).**
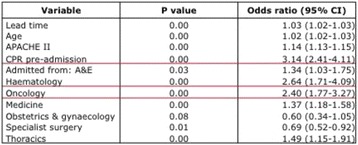
Predicting survival in all admissions; logistic regression model

The logistic regression model used is shown in Fig. 98. Factors strongly associated with mortality were referral from haematology (OR 2.64), oncology (OR 2.40) or admission after CPR (OR 3.14). Area under the ROC was 0.8.

**Fig. 99 (Abstract P396).**
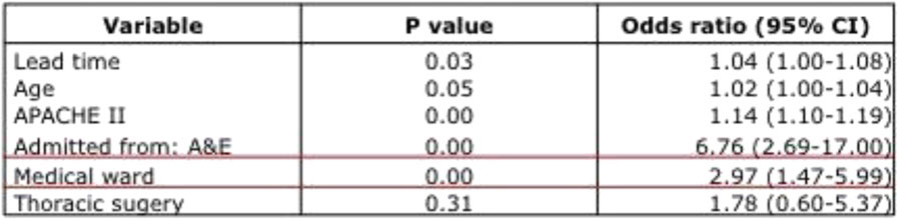
Predicting survival after CPR; logistic regression model

366 patients were admitted after CPR. Overall ICU mortality in this group was 50 %. Logistic regression (Fig. 99) demonstrated a strong association between mortality and admission from a medical ward (OR 2.97) or A&E (OR 6.76). Area under the ROC was 0.81. Estimated ICU costs per survival to hospital discharge were £75,091 if admitted with an APACHE II predicted mortality >90 %, £48,000 for admission post-CPR, and £56,000 if referred from haematology.

**Conclusions:** Referral from haematology, oncology, and post-CPR are independently associated with poor outcome and a high cost per survivor. These results suggest a need to review our admissions policy in view of the severe resource constraint and bed shortages currently experienced.

## P397 Effects of icu weekend admission and discharge on mortality.

### E. Moreira, F. Verga, M. Barbato, G. Burghi

#### Hospital Maciel, Montevideo, Uruguay

**Introduction:** Mortality is one of the most used outcome indicator among critical care patients. It depends on several variables like comorbidities, reason for admission and severity of the disease. Less attention has been directed to how the timing of admission and discharge from Intensive Care Unit (ICU) may influence hospital mortality.

The aim of this study was to determine the impact on ICU and hospital mortality of the moment of admission to and discharge from the Intensive Care Unit.

**Methods:** To this end we developed a prospective observational study, which included patients admitted between April and November 2014 in the ICU of the Maciel Hospital of Montevideo-Uruguay. We consider weekdays between monday to thursday, and weekend friday to sunday. Nocturnal admission was defined between 8:00 PM to 7:59 AM.

**Results:** We analyzed 325 patients with a median age of 55 (36-71) years and SAPS II of 43 (29-58) value points. No differences in mortality in ICU were found when comparing the time of admission (35 % on weekends vs 31 % on weekdays, p = ns) nor the time of entry (nocturnal 35 % vs diurnal 31 % , p = ns). The discharge day was associated with higher hospital mortality (57 % in the weekend vs. 14 % on weekdays, p = 0.000).Factors associated with hospital mortality by uni variate analisys were: age less than 50 years (43 % vs 17 %; p = 0.000); SAPS II higher than 35 points (36 % vs 21; p = 0.017), weekend discharge from ICU (57 % vs 14 %, p = 0.000), COPD (46 % vs 26 %, p = 0,031), cardiovascular comorbidity (46 % vs 25 %, p=,005) , and tracheostomised patients (42 % vs 25 %, p = 0,019).

Factors independently associated with hospital mortality after discharge from the ICU were patients older than 50 years (OR 2.4; 95 % CI, 1.1 to 5.4) and discharge during the weekend (OR 7.7 95 % CI, 3.8 to 15.6).

**Conclusions:** This study shows that the moment of discharge was independently associated with hospital mortality.

## P398 Organizational factors, outcomes and resource use in 9,946 cancer patients admitted to 70 ICUs

### M Soares ^1^, U. V. Silva^2^, L. C. Azevedo^3^, A. P. Torelly^4^, J. M. Kahn^5^, D. C. Angus^5^, M. F. Knibel^6^, P. E. Brasil^1^, F. A. Bozza^1^, J. I. Salluh^1^

#### ^1^DOr Institute for Research and Education - IDOR, Rio De Janeiro , Brazil; ^2^Hospital de Cancer de Barretos, Barretos, Brazil; ^3^Hospital Sírio Libanês, Sao Paulo, Brazil; ^4^Sta. Casa de Porto Alegre, Porto Alegre, Brazil; ^5^University of Pittsburgh Medical Center, Pittsburgh, USA; ^6^Hospital Sao Lucas, Rio de Janeiro, Brazil

**Introduction:** To investigate the impact of organizational factors on the outcomes and resource use in cancer patients admitted to a large sample of Brazilian ICUs.

**Methods:** Retrospective cohort study of 9,946 cancer patients (solid = 8,956; hematological = 990) (surgical = 4,929; medical = 5,017) admitted to 70 ICUs (51 located in general hospitals and 19 in cancer centers) during 2013. We retrieved clinical and outcome data from an electronic ICU quality registry (Epimed Monitor System). We surveyed ICUs regarding hospital and ICU structure, organization, staffing patterns and process of care. We used multilevel logistic regression analysis to identify characteristics associated with hospital mortality. We evaluated efficiency in resource use using standardized mortality rates (SMR) and resource use (SRU) based on the SAPS 3 score.

**Results:** ICUs were mostly medical or medical-surgical (n = 60, 86 %) and located in private hospitals (n = 66, 94 %). Median number of cancer patients per center was 110 (IQR 58-154), corresponding to 16 % (9 %-25%) of all ICU admissions. Median SAPS 3 score was 47 (36-59) points; mechanical ventilation (MV) was used in 21 % of the patients. ICU and hospital mortality rates were 15.9 % and 25.4 %, respectively. Adjusting for relevant patients’ characteristics (age, MV use, admission diagnosis, cancer type, performance status, comorbidities, neutropenia), the presence of clinical pharmacist in the ICU (OR = 0.67 (95 % CI, 0.49-0.90), the number of protocols (OR = 0.92 (0.87-0.98)) and daily meetings between oncologists and ICU team (OR = 0.69 (0.52-0.91)) were associated with lower mortality. Conversely, mortality was higher in ICU holding training programs in critical care (OR = 1.38 (1.05-1.81)). The number of protocols (OR = 1.52 (1.11-2.07)) and meetings between oncologists and ICU team (OR = 4.70 (1.15-19.22)) were also independently associated with a more efficient resource use (both low SMR and SRU). Admission to ICUs in cancer centers compared to general hospitals and annual case-volume had no impact on mortality or resource use.

**Conclusions:** Organizational aspects, namely the implementation of protocols and presence of clinical pharmacists in the ICU, and close collaboration between oncologists and ICU team are targets to improve mortality and resource use in critically ill cancer patients.

**References**

Funded by IDOR, CNPq and FAPERJ. Endorsed by BRICNet.

## P399 Evaluation of oncological critically ill patients, severity score and outcome compared to not oncological in a particular hospital cti.

### M. B. Velasco , D. M. Dalcomune

#### Hospital Meridional S.A., Cariacica, Brazil

**Introduction:** Cancer has grown steadily in ICU. The cancer patients are gradually improving the treatment of the underlying disease, both the earlier diagnosis, as most effective treatment, this lead to a better prognosis. And the prospect is that these numbers reach higher proportions over the years. But when you have to decide how much admission or not in a ICU? Based on this question we decided to retrospectively (nearly two years) evaluate the results of hospitalization of patient with or without cancer, and compare their major clinical outcomes. This study aimed to improve the knowledge of the main clinical outcomes of cancer patients in the ICU of a large private hospital. Can we break the paradigm that cancer patients may have a non-significantly different evolution of a patient without cancer admitted to the ICU?

**Methods:** We evaluated 2341 patients admitted in ICU during the period from Jan 2014 to Oct 2015. It was an observational study which was assessed retrospectively, the data of all adults patients admitted in ICU (excluding terminals). We separated in two groups: (1) With some oncologic disease (2) Without oncologic disease. After the division of the groups they were analyzed in comparison to each other in age, sex, SAPS3 severity score, ICU and hospital length stay, final outcome in the ICU and hospital. Data were collected from Epimed System and the MV system, tabulated in Excel and studied statistically.

**Results:** The group (1) with 2077 patients (56 % male) had a mean age of 60.0 ± 18.9; SAPS3 41.1 ± 13.3; ICU stay 6.8d ± 10.3; hospitalization 19.2d ± 35.6 while group (2) with 264 (58 % male) had a mean age of 62.4y ± 15.9; SAPS3 42.2 ± 12.9; ICU stay 6.9d ± 9.6; hospitalization 20.4d ± 27.4. None of these values showed significance with the Student-t.

Mortality for the group (1) in the ICU and hospital was 7.5 % and 10.3 % respectively an the group (2) was 10.6 % and 18.6 %. The expected mortality by SAPS3 to group (1) was 12.3 % ± 15.4 and (2) 13.4 % ± 15.2. The Z test was evaluated for two proportions and there was no difference in mortality in the ICU for both groups, there were differences for in-hospital mortality, was more significant for cancer patients.

**Conclusions:** Oncology patients have the same outcome as in the ICU, so the fact of the presence of the tumor can not be an obstacle in the hospital in ICU.

**Reference**

1: Parakh S, Piggin A, Neeman T, Mitchell I, Crispin P, Davis A. Outcomes of haematology/oncology patients admitted to intensive care unit at The Canberra Hospital. Intern Med J. 2014 Nov;44(11):1087-94;

## P400 Outcomes of patients admitted to a large uk critical care department with palliative oncological diagnoses

### R. Marshall^1^, T. Gilpin^1^, A. Tridente^2^, A. Raithatha^1^

#### ^1^Sheffield Teaching Hospitals, Sheffield, UK; ^2^Whiston Hospital, St Helens & Knowsley, UK

**Introduction:** Palliative oncology patients are admitted to critical care not uncommonly, outcomes are variable and often difficult to predict. We sought to observe Intensive care unit (ICU) and hospital outcomes of palliative oncology patients, in relation to demographic, disease and organ failure criteria.

**Methods:** Retrospective review of the electronic database and notes of patients admitted to ICU between January 2012 and October 2014 was undertaken. Patients without a formal palliative diagnosis or with a diagnosis of haematological malignancy, were excluded. Demographics, functional status, current cancer treatment, interventions received, and palliative care team involvement were amongst parameters collected. SAPS II, APACHE II scores and ICU outcome were recorded. STATA 14.1 was used for Logistic regression analyses.

**Results:** 31 patients were identified, median (IQR) age was 68 (62-76) years, 14 (45.2 %) were male, median (IQR) SAPS II score was 56 (53-71). Of 28 patients, where information was available, 18 (64.3 %) had metastatic disease; 16 (61.5 %) were undergoing palliative chemotherapy and/or radiotherapy, 5 (19.2 %) palliative surgical intervention. Median (IQR) LOS was 3.25 (1.3-5.1) days and 6 (19.4 %) patients had died at CC discharge. Hospital mortality was 29.6 %. Median survival was 7 days in hospital patients and 143 days in patients discharged home. At logistic regression analysis, adjusted for age and gender, the only factor associated with mortality at ICU discharge was SAPS II score (OR 0.9, 95%CI 0.8-1, p = 0.046), while length of stay (LOS), type of palliative treatment received prior to ICU admission, metastatic spread, neutropenia, smoking and diabetic status and highest recorded lactate in the 24 hours prior to ICU admission seemed to not be associated with ICU outcome. The lack of statistical significance for these correlations may be related to the small sample size.

**Conclusions:** A high SAPS II score may confer accurate prognostic information in the context of critically unwell palliative cancer patients. Overall ICU mortality was lower than those documented by previous works (47-70 %), [1][2][3] this may be related to strict triage by both the ICU and oncology teams. Median survival of palliative oncology patients admitted to critical care remains poor.

**References**

[1] Soares M et al. Crit Care 2004; 8:194-203

[2] Nelson J et al. CCM 2001; 29(2): 277-282

[3] Kress J et al. CCM 1999; 160: 1957-1961

## P401 Predictors of mortality in febrile neutropenic patients with haematological malignancies admitted to an intensive care unit of a cancer center

### D. Mota^1^, B. Loureiro^2^, J. Dias^2^, O. Afonso^2^, F. Coelho^2^, A. Martins^2^, F. Faria^2^

#### ^1^Centro Hospitalar e Universitário de Coimbra, Coimbra, Portugal; ^2^Instituto Português de Oncologia Francisco Gil - Porto, Porto, Portugal

**Introduction:** Hemato-oncological patients with neutropenic sepsis or septic shock are a growing concern in oncological Intensive Care Units (ICU). Acute Physiology and Chronic Health Evaluation II (APACHE II) and Simplified Acute Physiology Score II (SAPS II) are used to predict prognosis in critical patients based in clinical and analytic criteria obtained in the first 24 hours after admission. Through this study we aim to analyse which factors predict mortality in a cohort of febrile neutropenic (FN) patients with haematological malignancies.

**Methods:** We carried out a retrospective study which evaluated all hemato-oncological patients with FN admitted to an ICU in a comprehensive cancer center between January, 2011 and December, 2014. Baseline clinical and demographic information was described using descriptive statistics. Factors predicting in-ICU mortality were identified using univariate logistic regression. Variables with a p value <0.20 were included in a multivariate analysis.

**Results:** A total of 54 patients were included for analysis. The median age at admission was 48 years and 51.9 % were male (N = 28). The baseline cause of immunosuppression was Alogeneic Stem Cell Transplant (N = 20) and recent Chemotherapy (N = 34). The SAPS II and APACHE II scores had a median of 64 and 28.5, respectively. Multiple organ dysfunction was observed in 79.63 % of cases (N = 43). The single more frequent dysfunctions were respiratory, in 94.44 % (N = 51), renal, in 62.96 % (N = 34), and cardiovascular dysfunction, in 57.40 % of patients (N = 31). Renal replacement therapy was required in 11 cases. Invasive ventilation was performed in 81.48 % of patients (N = 44), with median duration of 168 hours. The median ICU length of stay was 8 days. In-ICU mortality was 31.48 % (N = 17). In univariate analysis, the APACHE II (OR 1.279, p value <0.01), SAPS II (OR 1.082, p value <0.01), renal dysfunction (OR 3.403, p value <0.05) and invasive ventilation (IV) (OR 8.7, p value <0.20) were associated with in-ICU mortality. In a multivariate analysis, APACHE II remained statistically significant (OR 1.546, p value 0.018, AUC 0.84) as IV in an exact logistic analysis.

**Conclusions:** A high APACHE II score and IV seems to predict an elevated mortality risk in ICU patients with FN and haematological malignancies. SAPS II appears to be a less important predictor of mortality in this setting. However, it should be noted that the scores shouldn’t be used to ascertain prognosis in individual cases.

## P402 Patients with hematologic malignancies requiring invasive mechanical ventilation: characteristics and predictors of mortality

### H. Al-Dorzi, H. Al Orainni , F. AlEid, H. Tlaygeh, A. Itani, A. Hejazi, Y. Arabi

#### King Saud bin Abdulaziz University for Health Sciences , Riyadh, Saudi Arabia

**Introduction:** Acute respiratory failure may complicate the course of patients with hematological malignancies and has historically been associated with poor prognosis. This study evaluated the characteristics, outcomes and predictors of mortality of patients with hematologic malignancies who required intubation.

**Methods:** This was a retrospective study of all patients with hematological malignancies who were admitted to the intensive care unit (ICU) of King Abdulaziz Medical City-Riyadh from 01/01/2008 to 31/12/2013 and required intubation. We noted their baseline clinical characteristics, treatments received in the ICU and different outcomes. Multivariate logistic regression analysis was performed to evaluate predictors of hospital mortality. Variables entered in the model were gender, APACHE II score, PO2:FiO2 on admission, admission serum lactate and INR, septic shock, noninvasive ventilation before intubation, renal replacement therapy and chemotherapy administration in the ICU.

**Results:** In the 6-year period, 190 patients with hematological malignancies were admitted to the ICU and 123 (64.7 %) required intubation for acute respiratory failure. These patients had mean age = 56.9 ± 19.6 years and APACHE II score = 27.9 ± 7.9 and were predominantly males (63.4 %). Lymphomas (43.9 %) were the most common hematologic malignancy followed by acute leukemias (39.0 %). Only 4 patients had bone marrow transplantation. Septic shock was present in most (62.6 %) patients on ICU admission. Noninvasive ventilation was tried in 23 (18.7 %) but failed. Chemotherapy was provided in 19 (15.4 %) patients during ICU stay. New renal replacement therapy for acute kidney injury was used in 18 (14.6 %) patients. Hospital mortality was 69.1 % (64.6 % for acute leukemias and 70.4 % for lymphomas, p = 0.68). Most deaths (82.4 %) occurred in the ICU. On multivariate analysis, male gender (odds ratio, 4.81; 95 % confidence interval, 1.69-13.72), septic shock (odds ratio, 5.67; 95 % confidence interval, 1.92-16.75) and new renal replacement therapy (odds ratio, 13.59; 95 % confidence interval, 1.14-161.93) were independent mortality predictors. Neither noninvasive ventilation failure nor chemotherapy during ICU stay was associated with mortality.

**Conclusions:** Patients with hematologic malignancies requiring intubation had high mortality (69 %). Male gender, septic shock and renal replacement therapy were independent predictors of mortality.

## P403 Patient-important outcomes in randomized controlled trials in critically ill patients: a systematic review

### S. Gaudry^1^, J. Messika^1^, J. D. Ricard^1^, S. Guillo^2^, B. Pasquet^2^, E. Dubief^1^, D. Dreyfuss^1^, F. Tubach^2^

#### ^1^Hôpital Louis Mourier, Colombes, France; ^2^Hôpital Bichat, Paris, France

**Introduction:** Intensivists’ clinical decision-making should pursue two main goals for patients: to decrease mortality and improve patient-centered outcomes such as quality of life and functional status in survivors.We sought to investigate how published randomized controlled trials (RCTs) explore these patient-important outcomes in critically ill patients.

**Methods:** Literature searches were conducted in Medline (via Pubmed®) to identifiy eligible articles indexed from January 2013 to December 2013.Articles were eligible if they reported an RCT involving critically ill adult patients and were written in English. Data extraction was conducted by 2 independent senior intensivists on a standardized, pre-tested extract form. We assessed patients and study characteristics. All outcomes (primary and secondary) were collected, described and classified using 6 categories of outcomes including patient-important outcomes. Patient-important outcomes were represented by 2 entities: mortality at any time and patient-centered outcomes assessed after ICU discharge (such as quality of life and functional status).

**Results:** Of the 716 articles retrieved, 152 RCTs met the inclusion criteria and were assessed. These totaled 142,252 patients (age: 61 [54-66] years, male sex: 62 %). Most common topics were mechanical ventilation (31 %), sepsis (20 %) and nutrition (14 %).

Among the 152 primary outcomes 31 (20 %) were patient-important outcomes (mainly mortality, 25/31) but only 6 (4 %) were patient-centered outcomes assessed after ICU discharge (functional disability = 4; quality of life = 2). Among the 801 secondary outcomes, 169 (21 %) were patient-important outcomes (mainly mortality, 117/169) but only 52 (6 %) were patient-centered outcomes assessed after ICU discharge (quality of life = 22, functional disability = 19; neurological/cognitive performance = 7; handicap = 2; post-traumatic stress = 1; patient satisfaction = 1).

Ninety-six (63 %) RCTs reported at least one patient-important outcome (either primary or secondary outcomes) but only 17 (11 %) reported at least one patient-centered outcome assessed after ICU discharge. In a multivariate analysis, sepsis as topic (OR, 7.6; 95 % CI, 2.2-25.5) and RCTs with Jadad score > 3 (OR, 4.8; 95 % CI, 2.1-10.7) were more likely to assess at least one patient-important outcome.

**Conclusions:** A small percentage of outcomes used in RCTs in critically ill patients published in 2013 were patient-important outcomes and a large majority of them was mortality outcome.

## P404 Alopecia in survivors of critical illness: a qualitative study

### C . Battle, K. James, P. Temblett

#### Morriston Hospital, Swansea, UK

**Introduction:** Alopecia in adult survivors of critical illness has not been previously well researched. During acute critical illness, alopecia is of minimal concern to an ICU team, as survival is the primary aim. In the recovery phase of illness however alopecia can prove distressing. (1) The aim of this study was to investigate the incidence and nature of patient-reported alopecia in a cohort of critical illness survivors.

**Methods:** This was a single centre, qualitative survey study completed in the ICU Follow-up Clinic of a teaching hospital in Wales. All patients who had been discharged home for at least 12 weeks, following an ICU stay of three or more days and attending the Follow-Up Clinic, were invited to complete the survey. The survey was adapted from one used in a previous study and included questions regarding patient demographics and alopecia.(2) Statistical analysis included numbers (%) and medians (IQR). Comparisons between patients with and without alopecia were made using Fisher’s Exact test and Mann Whitney U test. Due to the anonymity of the surveys the Wales REC 6 confirmed ethical approval was not needed for this study.

**Results:** The survey was completed by 75 patients attending the clinic between July and December 2014. Median age was 65 years and 35 % of patients were male. Alopecia was reported in 13 (17 %) patients and the hair loss was gradual in 12 (92 %) of the 13 patients. The severity of hair loss in this cohort ranged between less than 10 % to over 50 %, with the location of hair loss varying between the patients. In all but one of the patients hair loss occurred gradually at between one and six weeks post-ICU discharge and lasted for between one and seven months. On analysis, no significant differences were found between the patients with and without alopecia in the demographic variables.

**Conclusions:** Limited research exists examining the incidence and nature of patient-reported alopecia in adult survivors of critical illness. The type, amount and location of the hair loss varied between patients in this study. The main limitation of this study is the survey design resulting in a lack of information regarding potential predictors of alopecia. The incidence reported in this study however suggests that further research is warranted.

**References**

1) Bernstein, G.M., Crollick, J.S., Hassett, J.M. Post-febrile telogen effluvium in critically ill patients. Crit Care Med 1988;16:98–99.

2) Pettignano R, Heard ML, Labuz MD et al. Hair loss after extracorporeal membrane oxygenation. Pediatr Crit Care Med 2003;4:363-366.

## P405 The impact of mental health on icu admission

### L. Davies, C. Battle, C. Lynch

#### Abertawe Bro Morgannwg University Health Board, Swansea, UK

**Introduction:** South Wales is an area of relative socio-economic deprivation, with a myriad of well-recognised associated mental health problems. Patients with mental health disorders are commonly admitted to an ICU as a direct consequence of their illness, such as a suicide attempt, or due to indirect consequences, such as an intravenous drug overdose in drug addicts. As a result of these findings we set out to ascertain the effect of pre-existing mental health issues including addiction on ICU admission.

**Methods:** A prospective study design was used in which data was collected on all admissions to the general ICU of a large teaching hospital in South Wales, over a two month period. All adult patients were screened for the following questions; Was the admission a direct consequence of a mental health disorder or recreational/ illicit psychoactive substance use? Is there a co-existing mental health diagnosis present prior to admission? ICD-10 codes were used to define mental health disorders. Outcomes studied included in-hospital mortality, ventilator days and length of ICU stay. Data analysis included Fisher Exact and Mann Whitney U tests. Statistical significance was set at p < 0.05. Ethical approval was waived for this service evaluation.

**Results:** A total of 192 patients were screened with 51 (27 %) patients admitted with a mental health disorder (15 patients as a direct consequence and 36 patients with an existing mental health problem). The patients with a mental health disorder were significantly younger than the controls (p < 0.05) however there was no difference in APACHE II scores. There were no significant differences in mortality, mechanical ventilation days or ICU length of stay between the two groups. There was a significant difference in the mortality rate between the patients admitted as a direct consequence of a mental health disorder and the control group (p < 0.05)

**Conclusions:** Patients with mental health problems appear to have worse outcomes than those without. The mortality of those admitted to ICU as a direct consequence of their mental health problems is significantly higher than controls. The reasons for this are unclear and likely multi-factorial. A future multi-centre prospective study is planned in Wales to investigate the national impact of mental health disorders on ICU resources.

## P406 Cognitive impairment 5 years after ICU discharge

### S. Pereira, S. Cavaco, J. Fernandes, I. Moreira, E. Almeida, F. Seabra Pereira, M. Malheiro, F. Cardoso, I. Aragão, T. Cardoso

#### Centro Hospitalar Porto, Porto, Portugal

**Introduction:** Survivors of critical illness often have an incapacitating form of cognitive, psychological and functional impairment but the potential reversibility of these clinical conditions 5 years after intensive care unit (ICU) discharge remains inadequately understood. Our aim is to evaluate long-term cognitive impairment, psychological morbidity and quality of life 5 years after ICU discharge.

**Methods:** Prospective cohort study of survivors, admitted to a mixed ICU between January and September 2010. Clinical evaluation was performed at 6 months and 5 years after hospital discharge and it included the Dementia Rating Scale-2 (DRS-2), Hospital Anxiety and Depression Scale (HADS), Post-traumatic stress syndrome 14-questions inventory (PTSS-14), Medical Outcomes Study 36-Item Short Form (SF-36) and the Euro Quality of Life 5 Dimensions (EQ-5-D) in both two moments.

**Results:** During the study period, 26 patients were evaluated in the outpatient clinic 6 months after ICU discharge, with a mean age of 63 ± 16 years; of these, 18 were re-evaluated 5 years after ICU discharge. Twelve (46 %) patients presented cognitive impairment (DRS-2 < 10 percentile) in the first evaluation and 3 (17 %) at 5 years (p = 0.063). Anxiety and depression at 6 months was present in 5 (19 %) and 4 (15 %), at 5 years only 2 patients reported anxiety and none had depression. Evaluation with SF-36 did not show significant differences in the physical function [53 (39-75) vs 64 (43-94), p = 0.619], physical pain [78 (39-90) vs 58 (35-100), p = 0.810], general health [59 (39-68) vs 62 (42-71), p = 0.845], vitality [63 (38-80) vs 56 (22-78), p = 0.276], social function [50 (38-63) vs 100 (25-100), p = 0.179], emotional performance [100 (0-100) vs 100 (67-100), p = 0.091] and mental health [66 (54-86) vs 67 (29-79), p = 0.098]; differences were found in physical performance [100 (0-100) vs 89 (41-100), p = 0.010] and in health status change for worse: 4 (15 %) vs. 6 (33 %) patients (p = 0.018). Evaluation with EQ-5-D showed that 27 % vs. 50 % (p = 0.031) had some problems in self-care, 46 % vs. 58 % (p = 0.549) in usual activities, 62 % vs. 62 % (p = 1.000) reported pain/discomfort, 58 % vs. 65 % (p = 0.727) some problems in motility and 50 % vs. 50 % (p = 1.000) anxiety/depression; visual analogue scale was 80 (50-80) at 6 months vs. 60 (50-82)points at 5 years (p = 0.599).

**Conclusions:** In our cohort, patients did not show an increase in cognitive deterioration, nor in anxiety or depression. There was a deterioration in physical function and a higher proportion of patients reported a health change for worse during the previous year, but that did not translate in worse quality of life.

## P407 Apache ii versus apache iv for octagenerians in medical icu

### M. Fister, R. Knafelj

#### Rihard Knafelj, Ljubljana, Slovenia

**Introduction:** ICU illness severity scores are used in the ICUs for outcome prediction and guidance to human and financial resources use. Applicability of severity illness scores to octagenerians in medical ICU remains unclear.

**Methods:** Retrospective analysis of 244 consecutive octogenarians admitted to our MICU in 2014 was performed calculating APACHE II and IV. Predicted mortality was compared to actual mortality. Actual and predicted length of stay were compared.

**Results:** Out of 1075 admitted patients, there were 244 octagenerians (23 %) with 28 % ICU mortality. ICU mortality in younger patients was 19 % (p < .005). Both APACHE II and IV predicted 50 % mortality thus overestimating it by nearly 45 %. Actual LOS (3 days) was statistically shorter then predicted (6 days), p < 0.005.

**Conclusions:** APACHE II and APACHE IV correlate poorly with observed mortality when applied to octagenerians in MICU. There is discrepancy between predicted and actual LOS.

**Reference**

Vincent JL, Noreno R. Scoring system in critically ill. Critical Care 2010,14:207.

**Fig. 100 (Abstract P407).**
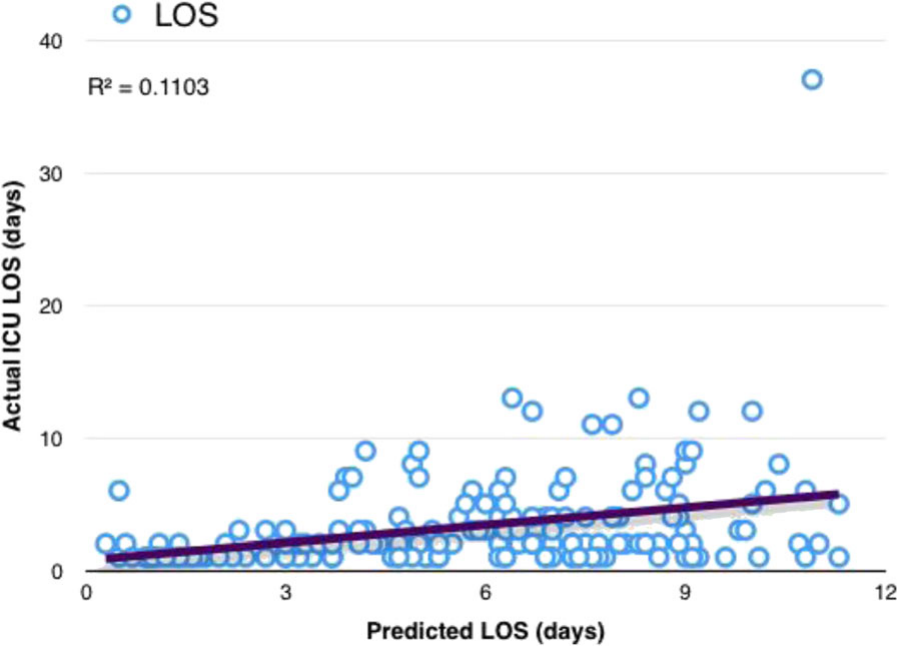
Predicted vs Actual LOS

**Fig. 101 (Abstract P407).**
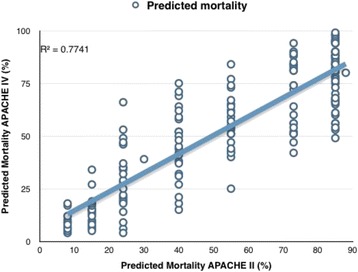
Mortality rates

## P408 Outcomes of octagenarians in an indian icu

### P. Muraray Govind, N. Brahmananda Reddy, R. Pratheema, E. D. Arul, J. Devachandran

#### Apollo Speciality Hospital - OMR, Chennai, India

**Introduction:** Advanced age is associated with increased illness severity leading to an increased mortality when critical care is required. Although the average life expectancy in India is 68 years, and only 0.9 % of the population is above 80 years of age, ICUs in major cities like ours admit a substantial proportion of octagenarians. Since the ICU outcomes of octagenarians in the Indian setting is not known, we conducted this retrospective review.

**Methods:** This retrospective review was conducted in a 19 bedded multidisciplinary ICU (mixed medical, surgical and coronary care) in the state of Tamilnadu in south India. Data was extracted from our ICU patient database for a one year period from Sept 2014 to Aug 2015. There were 470 admissions during this period, of which 53 patients were octagenarians making it 11.3 % of the total admissions. The demographics and outcomes of octagenarians were compared with the rest of the ICU admissions with full available APACHE 4 during this period.

**Results:** The average age of octagenarians admitted to our ICU was 84 years. Unlike the rest of the admissions, there were more females (55 %) amongst the octagenarians. Their average APACHE 4 score was 57.6 with a predicted mortality of 13.9 %. This correlated well with the actual mortality of 11.3 %. There was no statistically significant difference between the length of ICU stay or mortality between the octagenarians and the rest of the admissions. The number of cardiac patients between the two groups was also comparable.

**Conclusions:** 11.3 % of all our ICU admissions were octagenarians. Compared to the western population, octagenarians admitted to our ICU seem to have a lower predicted and actual mortality indicating less illness severeity. This could be due to a combination of patient factors, healthcare factors, healthcare delivery location and cultural factors. Published western data may not be helpful for discussing the outcomes with the patient and the family or decision making, before or after ICU admission.

**References**

1. Roch A et al. Long-term outcome in medical patients aged 80 or over following admission to an intensive care unit. . Critical Care 2011, 15:R36.

2. Ford PNR et al. Determinants of outcome in critically ill octogenarians after surgery: an observational study. British Journal of Anaesthesia 2007. 99 (6): 824–9.

## P409 Mortality and outcomes in elderly patients 80 years of age or older admitted to the icu

### M. B. Velasco, D. M. Dalcomune

#### Hospital Meridional S.A., Cariacica, Brazil

**Introduction:** The brazilian population is ageing and this is affecting trends in the percentage of hospital and intensive care unit (ICU) admissions attributable to patients aged 80 years or older, the very elderly. In 2050 the population elderly will be a quarter of the total population. Advanced age is associated with increased severity of acute critical illnesses and admission to ICU. They have a high risk of death.

**Methods:** We evaluated age-specific 2329 subjects > = 15y old and retrospectively admitted to the ICU from Jan 2014 to Oct 2015. We analyzed the clinical data of the subjects and divided into two groups: less than 80 (L80) and greater than or equal to 80 years (G80). We evaluated the major clinical outcomes and compared the two groups.

**Results:** We had 56.2 % male. The average length of stay in ICU (L80 = 6.5d ± 10.2; G80 = 8.2 ± 10.5) and hospital (L80 = 18.7 ± 35.9; G80 = 23.0 ± 28.7) showed no significant difference between the two groups. Mean SAPS 3 was 41.2 ± 13.3, with differences between the L80 group (38.8 ± 12.5) and G80 (53.0 ± 10.8) as well as the probability of death L80 = 9.8 % ± 13.6 and G80 = 24.8 % ± 17.3, respectively. As might be expected mortality, both in ICU or hospital were higher in the older group with significance according to the Z test for two proportions.

**Conclusions:** Age did not affect the length stay in ICU and hospital. The elderly subjects (> = 80y) admitted to the ICU had a higher mortality rate compared with subjects of other ages. These findings raise questions about the use of critical care at the end of life for the very elderly.

**References**

1. Sim YS et al. Mortality and outcomes in very elderly patients 90 years of age or older admitted to the ICU. Respir Care. 2015 Mar;60(3):347-55

2. MB Velasco et al. Study of the costs of an ICU according to age groups relating to SAPS 3 gravity, stay and outcomes. Crit Care. 2015; 19 (Suppl 1): P528

3. Santana-Cabrera L et al. Influence of age in the duration of the stay and mortality of patients who remain in an Intensive Care Unit for a prolonged time. Rev Clin Esp (Barc). 2014 Mar;214(2):74-8.

## P410 Octagenerians in medical icu - adding days to life or life to days?

### R. Knafelj, M. Fister

#### Rihard Knafelj, Ljubljana, Slovenia

**Introduction:** Compared to younger patients, octagenerians experience higer shor- and long-term mortality rates, and their admission rates over the last decade is increasing. The purpose of this study was to evaluate ICU and hospital mortality and mortality associated with ICU readmission.

**Methods:** Single center retrospective study in tertiary center academic hospital. Charts of 244 octagenerians consecutively admitted to medical ICU were analyzed. Pre -ICU performance was assesed, ICU mortality (APACHE IV), rate of remissions and hospital mortality was analyzed.

**Results:** In 2014, 1075 patients were admitted to our MICU, 244 aged 80 or more, oldest being 98. ICU mortality among octagenerians was 28 % and was significantly higher compared to the younger patients (19 % mortality; p <0.0005). None of the patients aged 80 or more diagnosed with sepsis survived ICU. In-hospital mortality of octagenerians was 43 %. 10 patients (4 %) were readmitted to ICU, none of whom survived. Pre-admission performance status was not predictive considering ICU or hospital mortality.

**Conclusions:** Octagenerians have higher ICU mortality rates compared to younger patients and their in-hospital mortality doubles. Readmission to ICU in analyzed period was associated with 100 % mortality. Pre ICU performance status was independent risk factor for mortality. Careful evaluation is warranted before readmitting elderly patient to MICU. We encourage making clear therapeutic plan after ICU discharge with clear recommendations considering potential ICU readmission, re-introducing invasive or non-invasive mechanical ventilation, vasopressor or commencing renal replacement therapy and antibiotic treatment.

**Reference**

Orsini J, Blaak C, Shamian B, Fonesca X, Salem A, Chen YL. Assessing the utility of ICU admission for octogenarians. Aging Clinical and Experimental Research:2015;5:1-7.

**Table 50 (Abstract P410).** Mortality rates

	No of patients	ICU deaths n, %	Hospital deaths n, %	Readmission n, %	Readmission deaths
Male	101	29 (29%)	42 (42%)	4 (4%)	4 (100%!
Female	143	40 (28%)	64 (46%)	6 (6%)	6 (100%)
All	244	69 (29%)	106 (43%)	10 (5%)	10 (100%)

## P411 The very elderly admitted to intensive care unit: outcomes and economic evaluation

### N. Chin-Yee^1^, G. D’Egidio^1^, K. Thavorn^1^, D. Heyland^2^, K. Kyeremanteng^1^

#### ^1^The Ottawa Hospital, Ottawa, Canada; ^2^Kingston General Hospital, Kingston, Canada

**Introduction:** Many elderly patients are admitted to intensive care units (ICUs) despite known poor outcomes and frequent patient preference to avoid unnecessary prolongation of life. The REALISTIC-80 (Realities, Expectations and Attitudes to LIfe Support Technologies in Intensive Care for Octogenarians) trial studied the outcomes of very elderly patients admitted to ICUs in Canada. Not only was mortality high, but long-term physical and functional recovery was also poor, raising questions about the appropriateness of ICU admission in this group. These findings also carry significant economic implications. Using data from REALISTIC-80 trial, we examined the cost of ICU admission for the very elderly, factors affecting this cost, and the potential savings of reducing admissions that are either undesired by the patient or medically futile.

**Methods:** This multicentre, prospective, observational cohort study included patients > =80 years old admitted to 22 Canadian ICUs from 2009-2013. Overall cost of care per patient was determined by length of stay and direct-variable costs for ICU admission. Using both univariate and logistic regression models, we investigated potentially predictive factors influencing cost, including patient outcome (death vs. survival), frailty, residence in a nursing home, as well as the presence of advance directives. An exploratory analysis was performed to illustrate the potential cost savings of reducing the number of ICU admissions in this population.

**Results:** In total, 3,064 patients > =80 years old were admitted to ICU, of which 1,917 were eligible, and 610 were enrolled in the study. The average age was 84 years; median length of stay was 5 days in ICU and 21 days in hospital. Mortality was 14% in ICU, 26% in hospital and 41% at 12 months. For non-survivors, the median time from admission to death was 12 days. Of enrolled patients, 240 (39%) remained in ICU for 7 days or more; 99 (41%) of these died in hospital. At 1 year, 28% of patients had meaningful recovery measured by the Short Form-36, a survey of general health status that includes both physical and mental health component scales. The results of the cost analysis are pending and will be presented at the conference.

**Conclusions:** In this study, we will demonstrate the significant economic costs of critical care in very elderly patients admitted to ICU—a population for whom this level of care is often unwanted or futile. More importantly, we expect to illustrate the potential cost savings of reducing these admissions, which will be crucial for informing clinical and policymaking decisions.

## P412 The very elderly in intensive care: relationship between acuity of illness and long-term mortality

### A. G. Murchison^1^, K. Swalwell^2^, J. Mandeville^3^, D. Stott^3^

#### ^1^Milton Keynes Hospital, Milton Keynes, UK; ^2^Imperial College, London, UK; ^3^Buckinghamshire Healthcare NHS Trust, Aylesbury, UK

**Introduction:** The purpose of this study was to determine the relationship between acuity of illness on ICU and two-year mortality for patients aged 85 or older. Few data exist about this association.

**Methods:** Patients aged 85 or older who were admitted to ICU at Stoke Mandeville and Wycombe Hospitals between Oct 2012 and Oct 2013 were identified. 24 month mortality was evaluated according to category of admission, organ support during ICU stay and APACHE III scores.

**Results:** 78 patients were included with a median age of 87 (range 85-96). 22 patients (28%) died during their ICU stay, and a further 12 (15%) died on the ward before discharge, giving a total of 34 (44%) hospital deaths. 6- and 24- month mortality were 54% and 60% respectively.

**Fig. 102 (Abstract P412).**
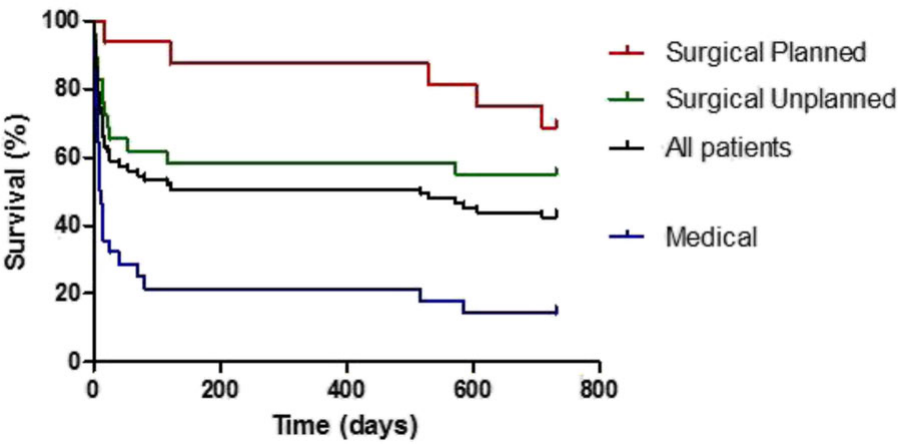
Survival curves by admission group over 24 months

**Fig. 103 (Abstract P412).**
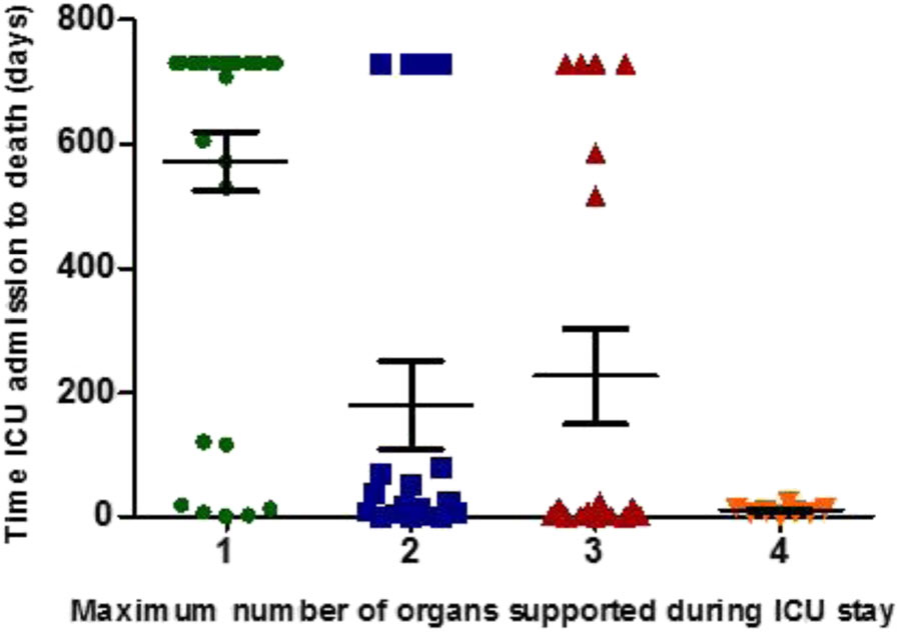
Association between number of organs supported and mortality (means with SEM)

Medical patients had the highest acuity scores and highest mortality at all end-points (Fig. 102). High APACHE III scores (Fig. 103) and increasing numbers of organs supported correlated with shorter time from ICU admission to death (p < 0.0001 in both cases). Of those who required support of 4-5 organs (8 patients), none survived their hospital admission.

**Conclusions:** Almost half of patients aged over 85 admitted to ICU died before discharge from hospital, but a large proportion of patients who survived 6 months were still alive at two years. High APACHE III scores and organ support were associated with higher mortality. These results suggest that intensive care is of benefit to selected patients aged over 85, and that acuity of illness is a useful indicator of long-term survival.

## P413 Acquired weakness in an oncological intensive care unit

### I. Guerreiro

#### IPO -Porto, Porto, Portugal

**Introduction:** Intensive Care Unit–acquired weakness (ICU-AW) is a frequent complication of critically ill patients with an approximately incidence of 25%-50%. The diagnosis is clinical and consists in assessing the strength of various muscles groups in the upper and lower extremities. This problem represents one of the greatest burdens patients face after surviving ICU care. Objectives: To assess the incidence and identify clinical factors associated with ICU-AW in an Oncology Center.

**Methods:** Retrospectively collected data on patients admitted to the ICU between January 2013 and December 2014 were reviewed. Patients admitted to the ICU, aged > = 18 years, mechanically ventilated for > = 48 hours and with the final diagnosis of ICU-acquired weakness were selected. Predictive factors of ICU-AW were identified using multivariate logistic regression analysis.

**Results:** From the 177 patients included, 20,3% developed ICU-AW. The mean age was 57,3 years and 58,3% were female. The median time of mechanical ventilation was 343,5 hours. 55,6% of patients had solid tumors and 44,4% had hematological malignancies. Glucocorticoids and neuromuscular blocking agents were administered in 88,9% and 38,9% of patients, respectively. Septic shock developed in 72,2% of patients and was found to be a predictive factor for developing ICU-AW (OR 4,15, pvalue 0,018). 66,7% of patients started or continued physical rehabilitation during the hospital length of stay approximately 12 days after admission in the ICU. 44,4% of patients with ICU-AW died 37 days after admission in the ICU. 8 patients (22,2%) with ICU-AW were re-evaluated by physical and rehabilitation medicine 144 days after hospital discharge. Significant improvement was noted in their physical status while in the program of physical therapy.

**Conclusions:** ICU-AW is a relatively frequent problem and septic shock was found to be a predictive factor for the development of this entity. Early mobilization is an important intervention to decrease the weakness and physical deconditioning in the critically ill patients.

**References**

-Kress J,Hall J.ICU-AW and recovery from Critical Illness.NEJM 2014,370:1626-35

-Lipshutz A,Gropper M.Acquired Neuromuscular Weakness and Early Mobilization in the ICU. Anesthesiology 2013,V118;No 1

-Wieske L et al.Impact of ICU-AW on post-ICU physical functioning:a follow-up study.Critical Care 2015,19:196

## P414 Musculoskeletal problems in intensive care unit (ICU) patients post-discharge

### H. Devine, P. MacTavish, J. McPeake, T. Quasim, J. Kinsella, M. Daniel

#### Glasgow Royal Infirmary, Glasgow, UK

**Introduction:** The aim of this study was to examine the incidence of musculoskeletal problems (i.e. pain, weakness, decreased joint range of movement) in critical care patients post discharge. Post intensive care syndrome (PICS) is now a widely used term to describe the collection of problems patients develop due to their stay in intensive care. ICU survivors have been found to have a high risk of developing not only psychological problems but physical problems such as Intensive Care Unit Acquired Weakness (ICUAW) and chronic pain [1, 2].

**Methods:** Discharged patients from ICU attended a 5 week multidisciplinary rehabilitation programme as part of a quality improvement initiative within Glasgow Royal Infirmary ICU. Participants completed a one-one musculoskeletal assessment with an ICU physiotherapist. Ethics approval was waived as the programme was part of a quality improvement initiative.

**Results:** Data was collected from 47 of the 48 patients who attended the programme (median age was 52 (IQR, 44-57), 67% of the patients were men, median ICU length of stay (LOS) was 15 days (IQR 9-25) and median APACHE II was 23 (IQR 18-27). 66% of participants (n = 47) reported a new incidence of pain since discharge from ICU, 28% reporting lower limb (LL) pain and 25% reporting shoulder pain. Bilateral symptoms were reported in 84% of those who complained of lower limb pain in contrast to 25% of those with shoulder pain. In relation to muscle weakness, 74% of participants presented with LL weakness compared with 51% in the upper limb (UL). UL joint range of movement was reduced in 40% of participants and a 19% reduction for the LL. 23% of all participants reported numbness in UL/LL or both.

**Conclusions:** Musculoskeletal problems especially shoulder pain and bilateral LL pain and weakness remain a significant problem for survivors of critical illness. This may have implications regarding falls risks, exercise capacity and reduce the likelihood of patients returning to work. Shoulder pain was found to be one of the most common complaints of pain supporting other research [1] with contributing factors such as the position of ventilator tubing, dialysis lines or central lines hypothesised. Collecting this data has helped raise awareness of these problems and may strengthen the case for more equipment for active mobilisation in ICU and herald a need for increased understanding in downstream wards on ICUAW.

**References**

1. Battle et al, Critical Care; 17:R101, 2013.

2. Griffiths et al, Critical Care; 17:R100, 2013.

## P415 Premorbid obesity, but not nutrition, prevents critical illness-induced muscle wasting and weakness

### C. Goossens, M. B. Marques, S. Derde, S. Vander Perre, T. Dufour, S. E. Thiessen, F. Güiza, T. Janssens, G. Hermans, I. Vanhorebeek, K. De Bock, G. Van den Berghe, L. Langouche

#### KU Leuven, Leuven, Belgium

**Introduction:** The ‘obesity paradox’ of critical illness refers to better survival with a higher BMI. We hypothesized that fat mobilized from excess adipose tissue during critical illness provides energy more efficiently than exogenous macronutrients and could prevent lean tissue wasting.

**Methods:** In a centrally-catheterized mouse model of cecal ligation and puncture (CLP)-induced prolonged septic critical illness, body weight and composition, and muscle wasting were assessed in lean and premorbidly obese mice, each with fasting and parenteral feeding [healthy mice: lean n = 8, obese n = 9; fed CLP mice: lean n = 7, obese n = 10; fasted CLP mice: lean n = 9, obese n = 9]. Muscle weakness was assessed in a second mice experiment examining ex vivo muscle force [healthy mice: lean n = 17, obese n = 15; fed CLP mice: lean n = 15, obese n = 15]. Mice were generated by providing 12-week old male C57BL/6J mice with ad libitum 10% fat chow or 45% fat chow for 12 weeks prior to the septic insult. Also, in matched lean (BMI < =25 kg/m2) and overweight/obese (BMI >25 kg/m2) prolonged critically ill patients and healthy controls, we compared markers of muscle wasting (m. vastus lateralis biopsies (n = 102) and m. rectus abdominis biopsies (n = 86)) as well as muscle weakness, quantified by Medical Research Council sum scores (n = 278).

**Results:** Five days of sepsis reduced body weight similarly in lean and obese mice, with more fat loss in the obese (p < =0.03). Lean CLP mice, but not the obese, showed reduced muscle mass (p < =0.04), muscle protein content (p < =0.06), myofiber size (p < 0.01), and muscle and hepatic triglyceride content (p < =0.06), irrespective of administered feeding. Obese CLP mice maintained normal maximal muscle force, whereas in lean CLP mice, maximal muscle force decreased (p < 0.01) and recovered less from fatigue (p < 0.01). These differences between lean and obese CLP mice coincided with signs of more effective hepatic fatty acid and glycerol metabolism, and ketogenesis in the obese. Also overweight/obese critically ill patients showed preserved myofiber size, while myofiber size reduced in lean patients (p = 0.02 in m. vastus lateralis biopsies, p = 0.01 in m. rectus abdominis biopsies). Furthermore, fewer overweight/obese patients suffered from muscle weakness, assessed 8 days post-ICU admission (p < 0.01).

**Conclusions:** In conclusion, during critical illness premorbid obesity, but not nutrition, facilitated utilization of stored lipids and attenuated muscle wasting and weakness.

## P416 Physical outcome measures for critical care patients following intensive care unit (icu) discharge

### H. Devine, P. MacTavish, T. Quasim, J. Kinsella, M. Daniel, J. McPeake

#### Glasgow Royal Infirmary, Glasgow, UK

**Introduction:** The aim of this study was to evaluate the most suitable physical outcome measures to be used with critical care patients following discharge. ICU survivors experience physical problems such as reduced exercise capacity and intensive care acquired weakness. NICE guideline ‘Rehabilitation after critical illness’ (1) recommends the use of outcome measures however does not provide any specific guidance. A recent Cochrane review noted wide variability in measures used following ICU discharge (2).

**Methods:** Discharged ICU patients attended a five week multidisciplinary programme. Patients’ physical function was assessed during the programme, at 6 months and 12 months post discharge. Three outcome measures were included in the initial two cohorts. The Six Minute Walk Test (6MWT) and the Incremental Shuttle Walk test (ISWT) were chosen as they have been used within the critical care follow up setting (2). The Chester Step Test (CST) is widely thought to be a good indicator of ability to return to work (one of the programmes primary aims). Ethics approval was waived as the programme was part of a quality improvement initiative.

**Results:** Data was collected for the initial patients attending the programme (n = 13), median age was 52 (IQR = 38-72), median ICU LOS was 19 days (IQR = 4-91), median APACHE II was 23 (IQR = 19-41) and 11 were men. One patient was so physically debilitated that the CST or ISWT could not be completed however a score was achieved using the 6MWT. Another patient almost failed to achieve level 1 of the ISWT. Subsequent patients for this project (total n = 47) have all therefore been tested using the 6MWT. Good inter-rater and intra-rater reliability and validity have been reported for the 6MWT (3).

**Conclusions:** Exercise capacity measurement is not achievable for some patients with either the ISWT or the CST due to the severity of their physical debilitation. Anxiety, post-traumatic stress disorder and depression are common psychological problems post discharge (4), therefore using a test with a bleep is not appropriate. Therefore, the 6MWT is the most appropriate physical outcome measure to be used with critical care patients post discharge.

**References**

1. NICE, Guideline 83, 2009

2. Connolly et al, The Cochrane Library, Issue 6, 2015

3. Weisman et al, Clin Chest Med, 22: 679-701, 2001

4. Jones et al, Clin Inten Care, Volume 9, Issue 5:199-205, 1998

## P417 Improving active mobilisation in a general intensive care unit

### B. Miles , S. Madden, H. Devine

#### Glasgow Royal Infirmary, Glasgow , UK

**Introduction:** We aimed to improve the active mobilisation of Intensive Care (ICU) patients through a quality improvement (QI) project. Only 50% of ICU patients return to work within one year of discharge [1]: there are ongoing physical, psychological and cognitive problems after ICU discharge [1, 2]. Active mobilisation shortens hospital stay, increases return to independent function [3] and reduces delirium [3]. UK ICU standards, introduced in 2013, direct 45 minutes/day of active mobilisation in suitable patients.

**Methods:** Our ICU selected a multidisciplinary (MD) team in January 2014 to lead the mobilisation QI project and a commitment was made to the weekly collection and presentation of data. We used the ADEPT (aim, data, evidence, process, team) format. Our initial aim was 20 minutes of active mobilisation daily in 95% of suitable ICU patients. Patients had to be able to obey commands, to have achieved a degree of cardiovascular stability and must have no musculoskeletal injuries precluding mobilisation. Vasopressor use and invasive ventilation per se were not barriers to active mobilisation. Agreed forms of active mobilisation were active limb exercises (a booklet of exercises was developed), bed edge sit (dangle), sitting out of bed in a chair, standing and walking. Baseline data was collected and serial Plan, Do, Study, Act (PDSA) cycles were carried out.

**Results:** Baseline data (March – April 2014) showed 34% daily mobilisation in the target group. Performance improved to a median of 95% by November 2014 and has been maintained from January to November 2015 at 94%. Our daily mobilisation aim was increased to 30 minutes in June 2015 and to 45 minutes in October 2015. We saw a reduction of 1.2 days in our ventilator length of stay September 2014 as better reliability in active mobilisation was achieved.

**Conclusions:** The results of this QI project show that a MD approach to mobilisation can achieve results. The weekly data collection and discussion proved essential in advancing success. We had no extra resources or new funding to help us increase mobilisation time and we used our data and successive PDSA cycles to achieve success.

**References**

1. Van der Schaaf M et al. Journal of Rehabilitation Medicine 41:1041-1048, 2009

2. Van der Schaaf M et al. Journal of Rehabilitation Medicine 41: 360-366, 2009

3. Schweickert WD et al. The Lancet 373: 1874–82, 2009

4. Core Standards for Intensive Care Units, Edition 1, Faculty of Intensive Care Medicine 2013

## P418 Mobilization in patients on vasoactive drugs use – a pilot study.

### M. Weiler, P. Marques, C. Rodrigues, M. Boeira, K. Brenner, C. Leães, A. Machado, R. Townsend, J. Andrade

#### Ernesto Dornelles Hospital, Porto Alegre, Brazil

**Introduction:** Recent studies show that ICU survivors who needed mechanical ventilation often presents with neuromuscular weakness and functional impairment. ICU Acquired Weakness may develop even in critical patients who are immobilized for only a few days despite receiving supportive care and physiotherapy. Such weakness contributes for poor outcome. Early mobilization may enhance functional recovery, reduce days in mechanical ventilation, shorten ICU and hospital length of stay, decrease the incidence of delirium and improve survival. A great part of ICU patients are on vasoactive drug therapy, but there are few data in literature about the safety of mobilization in this group. Therefore our study aimed to prove that early mobilization even of patients using low dose of vasoactive drugs is safe and feasible.

**Methods:** Whe assigned patients who were using or not low dose of vasoactive drugs to receive 2 daily sessions of physiotherapy during their ICU length of stay and analyzed their tolerance. The exercises consisted in 3 levels of mobilization: level 1 were passive exercises, level 2 were active exercises and level 3 were orthostatic position and walking. Hearth rate, respiratory rate, peripheral oxygen saturation and MAP were evaluated in 3 moments: before the physiotherapy session, immediately after and 30 minutes after.

**Results:** 154 patients were followed from November 3, 2015 to November 20, 2015. 50.6% of patients were men. Mean age was 71.1 in vasoactive group and 73.4 in control group. Mean SAPS 3 score was 73.6 in vasoactive group and 62.7 in control group (p < 0.05). The types of vasoactive drugs were 53.5% norepinephrine, 45.1% nitroprusside and 1.4% nitroglycerine. We removed from bed and putted on the seating position 34.7% of patients in the vasoactive group and 57.3% in the other group. There weren’t complications statistically significant. During the exercises there were changes on MAP, but with no major event associated, though increase in dose of vasoactive drug (p > 0.05). The only statistically significant difference between the two groups was increase in MAP in the vasoactive group immediately after mobilization (from mean MAP of 84.02 to 87.0). The mean dosage in mcg/kg/min of norepinephrine was 0.0117, nitroprusside 0.098 and nitroglycerin 0.278.

**Conclusions:** Our study showed that mobilization of patients on low dose of vasoactive drugs therapy may be possible and safe, with little complications and no major changes in MAP. These data are very important to encourage early mobilization of patients in ICU and to ameliorate their outcomes.

## P419 Pharmacy intervention at an intensive care rehabilitation clinic

### P. MacTavish, J. McPeake, H. Devine, J. Kinsella, M. Daniel, R. Kishore, C. Fenlon, T. Quasim

#### Glasgow Royal Infirmary, Glasgow, UK

**Introduction:** During an intensive care stay, patients often have their chronic medications withheld for a variety of reasons and new drugs commenced [1]. As patients are often under the care of a number of different medical teams during their admission there is potential for these changes to be inadvertently continued [2]. Intensive Care Syndrome: Promoting Independence and Return to Employment (InS:PIRE) is a five week rehabilitation programme for patients and their caregivers after ICU (Intensive Care Unit) discharge at Glasgow Royal Infirmary. Within this programme a medication review by the critical care pharmacist provided an opportunity to identify and resolve any pharmaceutical care issues and also an opportunity to educate patients and their caregivers about changes to their medication.

**Methods:** During the medication review we identified ongoing pharmaceutical care issues which were communicated to the patient’s primary care physician (GP) by letter or a telephone call. The patients were also encouraged to discuss any issues raised with their GP. The significance of the interventions was classified from those not likely to be of clinical benefit to the patient, to those which prevented serious therapeutic failure.

**Results:** Data was collected from 47 of the 48 patients who attended the clinic (median age was 52 (IQR, 44-57) median ICU LOS was 15 (IQR 9-25), median APACHE II was 23 (IQR 18-27) and 32 of the patients were men (67%). The pharmacist made 69 recommendations; including 20 relating to drugs which had been withheld and not restarted, dose adjustments were suggested on 13 occasions and new drug recommendations were made for 10 patients. Duration of treatment for new medications started during hospital admission was clarified on 12 occasions. Lastly adverse drug effects were reported on 4 occasions and the incorrect drug was prescribed on 2 occasions. Of the interventions made 58% were considered to be of moderate to high impact.

**Conclusions:** The pharmacist identified pharmaceutical care issues with 18.6% of the prescribed medications. Just over half of the patients reported that they were not made aware of any alterations to their prescribed medication on discharge. Therefore a pharmacy intervention is an essential part of an intensive care rehabilitation programme to address any medication related problems, provide education and to ensure patients gain optimal benefit from their medication.

**References**

1. Campbell AJ et al. Anaesthesia 61:1087−1092, 2006

2. Eijsbroek H et al.: J Critical Care 28: 46-50, 2013

## P420 Interactive gaming is feasible and potentially increases icu patients’ motivation to be engaged in rehabilitation programs

### T. Fiks, A. Ruijter, M. Te Raa, P. Spronk

#### Gelre Ziekenhuizen, Apeldoorn, Netherlands

**Introduction:** Light sedation has gained attention as part of standard daily care in the intensive care unit (ICU). Particularly early mobilization is associated with shorter time on the ventilator, shorter ICU length of stay and better survival. Interactive gaming may be a challenging way of engaging the patient in his own rehabilitation program. Few data are available for the use of these interactive games in the ICU envirnoment as part of daily routine physiotherapy, although one study showed that it was safe [1].

**Methods:** We developed a trolley with a Wii device that can be easily used when the patient is mobilized in a chair. In addition, we used the Mile™ device with a bedcycle and hypothesized that this would be associated with increased motivation to use the bedcycle by our patients.

**Results:** Patients liked to use the Wii device, particularly because a choice in games made it more interesting to use. Tennis, bowling and boxing were most frequently used. The use of the Wii was programmed in the daily mobilization schedule together with a physiotherapist, or just with the attending ICU nurse.

The mile™ bed-cycle device was used routinely since October 2015 with interesting results. Patients could choose the environment where they wanted to cycle around, e.g. “slot Zeist”, in the woods or using a water-cycle in the colourful village of Giethoorn. In parallel to the observation with the Wii device, patients liked the variety in scenes best. A study is in progress evaluating safety, as well as effects on motivation of patients using these interactive games in the ICU.

**Conclusions:** The use of interactive video games as part of routine rehabilitation programs in the ICU environment is feasible and appears safe in this short observation period. Interactive game therapy may complement existing rehabilitation techniques and increase motivation for ICU patients to be engaged in their own rehabilitation program.

**Reference**

Feasibility and observed safety of interactive video games for physical rehabilitation in the intensive care unit: a case series. Kho ME, Damluji A, Zanni JM, Needham DM. J Crit Care. 2012 Apr;27(2):219.e1-6

## P421 Simulation-based design of a robust stopping rule to ensure patient safety

### Y. S. Chiew^1^, P. Docherty^1^, J. Dickson^1^, E. Moltchanova^1^, C. Scarrot^1^, C. Pretty^1^, G. M. Shaw^2^, J. G. Chase^1^

#### ^1^University of Canterbury, Christchurch, New Zealand; ^2^Christchurch Hospital, Christchurch, New Zealand

**Introduction:** Stopping rules instigate early clinical trial termination when interim analysis shows statistically significant effects of treatment as part of a randomised controlled trial (RCT). This study aims to develop a stopping rule based on observing a powered and significant difference in mortality across RCT arms in a study.

**Methods:** The stopping rule was developed using a Monte-Carlo simulation approach with predetermined control group mortality rate (MC) and intervention group mortality rate (MI). This stopping rule is determined as the bias in mortality required to achieve a statistical significance of p < 0.05 with 80% power.

**Fig. 104 (Abstract P421).**
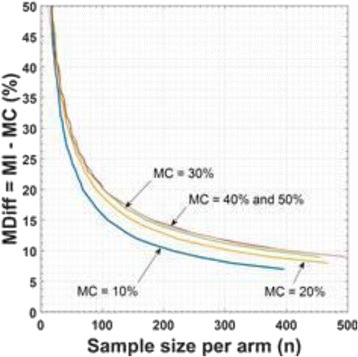
ᅟ

**Results:** The stopping rule determined is as shown in the Fig. 104. The y-axis is the mortality difference (MDiff) between MC and MI. Each line denotes a stopping rule for a different MC. For example, if MC is observed at 20% at sample size per arm of 100, the MDiff required to terminate the trials is 19%. Thus, the MI threshold for triggering the stopping rule is 39%. In practice, this means that if interim analysis of 100 patients per trial arm includes 20 deaths in the control group, the intervention group must observe 39 deaths or more to stop the trial (MI > = 39%).

For MC = 30% at sample size per arm of 430 patients, the MDiff from the Figure is approximately 9%. This mortality difference is similar to the result found in the ARDSnet trial when comparing lower and traditional tidal volume ventilation [1]. The ARDSnet trial found a mortality difference of 31.0% versus 39.8% was significant when ~430 patients per arm were recruited to the trial, and the early termination of the trial was triggered. This result further validates this stopping rule design method presented in this study.

**Conclusions:** This stopping rule design method can be adapted for any two-arm RCT that monitors probabilistic and binary outcomes. This approach in designing a RCT stopping rule can be adjusted to the participating cohort using local data and based on the desired study characteristics, and fulfills ethical needs for a statistically justifiable stopping rule.

**Reference**

1. The ARDS Network. NEJM, 2000. 342(18): p. 1301-1308.

## P422 Are daily blood tests on the intensive care unit necessary?

### T. Hall, W. C. Ngu, J. M. Jack, P. Morgan

#### Surrey and Sussex NHS Trust, Redhill, UK

**Introduction:** Daily blood tests are a long running feature of ICU care, and are regarded as an essential diagnostic tool by medical staff, however the risk of iatrogenic anaemia is a very real one and there are significant costs involved.^1^

**Methods:** Existing guidelines are intended to limit excessive testing in stable patients. We audited adherence to these and also recorded trends in haematocrit and haemoglobin to identify cases of iatrogenic anaemia on the unit. In order to establish trends and to exclude stable post surgical patients, we limited eligible patients to those who were on the unit for 3 days or more. On six randomly assigned days over a 2 month period, all eligible patients on the unit had a proforma filled retrospectively for all days up until their admission to the unit, or up to seven days previously, whichever sooner. Presence of arterial or central access was also noted, as these facilitate obtaining samples.

**Results:** We obtained 101 patient days worth of data across 20 patients who were considered eligible. Our results demonstrated that 54% of tests done were required but more importantly, 46% of tests carried out were deemed clinically unnecessary by a panel of three ICU physicians, and 48% were ordered despite the unit guidelines stating otherwise. The inpatient cost of carrying out the unnecessary blood tests added up to £842 and the amount of blood wasted totalled 1052 ml. Based on these figures, the average volume of unnecessary blood taken from a patient per week is 73 ml. Only 2 patients selected had no central or arterial line for part of their inspected days, and this had no effect as they had tests taken daily anyway.

**Conclusions:** Stopping short of suggesting an opt in approach to lab tests as other units have done^2^, we are recommending a period of education for existing nursing and medical staff, and also new trainees on the Intensive Care Unit. We are also recommending removal or alteration of the “Critical Care” test set that is currently available from our electronic requesting system in the hope that it will encourage more critical thinking about which tests are appropriate for the patient they are caring for.

**References**

1. Goddard K, Austin SJ. Appropriate regulation of routine laboratory testing can reduce the costs of associated with patient stay in the intensive care unit. Critical Care 2011;15:P133

2. R Gray* and F Baldwin. Targeting blood tests in the ICU may lead to a significant cost reduction. Critical Care 2014, 18:P15 doi:10.1186/cc13205

## P423 Measuring urine output in ward patients : is it helpful?

### B. Avard^1^, A. Pavli^1^, X. Gee^2^

#### ^1^The Canberra Hospital, Hughes, ACT, Australia; ^2^ANU Medical School, Canberra, Australia

**Introduction:** The significance of tracking urine output as a marker for patient deterioration on general wards remains unclear. We aimed to explore the utility of measurement of urine output in patients including those who deteriorated and required Intensive Care support and a cohort who did not, from both medical and surgical wards in a large metropolitan hospital in Australia. Of note this hospital had a program to recognise and respond to deteriorating patients well embedded, which included a modified early warning score and track & trigger response.

**Methods:** A retrospective cross sectional analysis was performed on 440 patients admitted to our hospital over a six month time period. We excluded patients with premorbid renal insufficiency or requiring dialysis prior to admission as these patients would have skewed results. We collected data on urine output, modified early warning scores, when patients were admitted to ICU the timing of deterioration being recognised and responded to, patient length of stay and mortality.All data was subsequently analysed using SPSS software. Ethics approval was obtained for this research.

**Results:** Only 9% of the ward patients had urine output measures performed, irrespective of whether they deteriorated to the point of ICU admission or remained on the ward. Despite this, those patients who had a low urine output recorded on the ward and who were subsequently admitted to ICU continued to have a lower mean output in the first 24 hours of their ICU stay. A statistically significant association was found between reduction in urine output and increased hospital mortality with a mortality rate of 7.9% in those with reduced output compared with 1.3% with normal output for those patients who were not subsequently admitted to ICU, with an even more pronounced correlation in those who deteriorated to the point of requiring ICU admission. 25% of those patients who received a medical review in response to deterioration may not have elicited a review if the urine output had not been monitored and included in the activation criteria.

**Conclusions:** This research suggests that urine output remains a vital component of our recognition of deteriorating patients and our focus should be on improving routine measurement of this parameter rather than removing this from modified early warning scores.More reliable measurement may allow further investigation as to whether earlier intervention based on this parameter may improve patient outcome.

**Reference**

Acute Med 2012 v11(2);p66; Kidney Int 2001 v80 p765; Anaesth 2005 v60,p547; ICM 2007 v33(4)p667; Annals ATS 2014v11(9)p1454

## P424 The incidence of pressure ulcers in an adult mixed intensive care unit in turkey

### C . Bor^1^, E. Akin Korhan^2^, K. Demirag^1^, M. Uyar^1^

#### ^1^Ege University School of Medicine, Izmir, Turkey; ^2^katip Celebi University, Izmir, Turkey

**Introduction:** Pressure Ulcers (PU) are common and serious complications in critically ill patients. This study aims to evaluate the incidence and risk factors that are associated with PU development in an adult mixed intensive care unit (ICU) in Turkey.

**Methods:** Ethics approval was obtained prior to start of the study. A prospective cohort study was performed from December 2013 to July 2014. Patients were screened daily until discharge or death, over a consecutive 28-day period. We collected data on demographic and baseline variables, co-existing diseases, serum albumin level, repositioning frequency, presence of mechanical ventilation, Acute Physiology and Chronic Health Evaluation II (APACHE II) score, Sequential Organ Failure Assessment (SOFA) score, Glasgow Coma Score (GCS) and vasoactive medications.

**Results:** A total of 194 patients were examined in the study, 30 (15,4 %) of them developed a PU. The factors associated with PU development were: serum albumin level (p =0,0004), generalized edema (p = 0,004), mechanical ventilation use (p = 0,0007), sedation (p = 0,01) and GCS (p = 0.00001). Age, gender, body mass index, APACHE II and SOFA scores, and repositioning were not different between patients with and without PU.

**Conclusions:** Pressure ulcers are common complications in adult mixed intensive care unit, with potentially severe consequences. The incidence of pressure ulcers were related with state of consciousness, sedation, mechanical ventilatory support and presence of generalized edema.

**References**

1. Oliveira Costa AC, Sabino Pinho CP, Almeida Dos Santos AD, Santos do Nascimento AC. Pressure Ulcer: Incidandc and Demographic, Clinical and Nutrition Factors Associated in Intensive Care Unit Patients. Nutr Hosp. 2015:1;32(5):2242-52. doi: 10.3305/nh.2015.32.5.9646

2. Mehta C, George JV, Mehta Y, Wangmo N. Pressure ulcer and patient characteristics--A point prevalence study in a tertiary hospital of India based on the European Pressure Ulcer Advisory Panel minimum data set. J Tissue Viability. 2015 Aug;24(3):123-30. doi: 10.1016/j.jtv.2015.04.001. Epub 2015 Apr 25

## P425 Intensivist/patient ratios in closed ICUs in Alexandria, Egypt; an overview

### M. Shirazy, A. Fayed

#### Alexandria University Faculty of medicine, Alexandria, Egypt

**Introduction:** There has been an increase in the number and size of closed ICUs in Alexandria, Egypt that has not been met by a comparable increase in the number of intensivists. Consequently, leading to work overload and negative impact on both patients and intensivists.

**Methods:** Using an in-person questionnaire of Consultants and In-house physicians in 27 closed ICUs in Alexandria, Egypt, obtaining information about their demographic data, patient workload, other hospital and medical duties and perception of the workplace and teaching environment.

**Results:** 254 Physicians responded. The average daily (SD) census of patients was 7.96 ± 6.24 patients, and the average (SD) maximum service size was 10.46 ± 6.75 patients. The median of consultant/patient ratio was (1:8), which classified 8 ICUs into Unfavourable ratio ICUs (>1:8), and 19 ICUs into Favourable ratio ICUs (<1:8). The median of In-house physician/patient ratio was (1:4.5), which classified 16 ICUs into Unfavourable ratio units (>1:4.5), and 11 ICUs Favourable ratio units (<1:4.5). Respondents from Unfavourable ratio ICUs perceived significantly more time constraints, teaching difficulties, burnout, and negative impact on patients.

**Conclusions:** Intensivists working in Unfavourable ratio ICUs frequently perceived being overburdened in comparison with those working in low ratio units. Also high physician/patient ratios may have a negative impact on teaching, patient care, and workforce stability.

**References**

1. Ward NS, Read R, Afessa B, Kahn JM. Perceived effects of attending physician workload in academic medical intensive care units: a national survey of training program directors. Crit Care Med 2012; 40(2):400-5.

2. Ward NS, Afessa B, Kalispell R, Tisherman S, Ries M, Howell M, et al.Intensivist/patient ratios in closed ICUs: a statement from the Society of Critical Care Medicine Taskforce on ICU Staffing. Crit Care Med 2013; 41(2):638-45.21.

3. Halpern NA, Pastores SM, Oropello JM, Kvetan V. Critical care medicine in the United States: addressing the intensivist shortage and image of the specialty. Crit Care Med. 2013; 41(12):2754-61.

## P426 Eicu (electronic intensive care unit): impact on ALOS (average length of stay) in a developing country like India

### S. Gupta^1^, A. Kaushal^1^, S. Dewan^2^, A. Varma^1^

#### ^1^Fortis Escorts Heart Institute, New Delhi, India; ^2^FMRI, Gurgaon, India

**Introduction:** Reduction in ALOS is an important efficiency indicator in any ICU [1]. We hypothesized whether eICU can create an impact on quality of service and financial implication thereof.

**Methods:** This retrospective study was done at a tier 2 city hospital with eICU support comprising of 36 ICU beds to assess the impact of eICU on ALOS for a period of 6months before and after inception. The study period was October 2014 to April 2015 and April to October 2015 respectively. The eICU had complete access 24*7 to patient’s real-time vitals, imaging, lab values, audiovisual and alerts. eICU support was pertaining to patient management, clinical protocol implementation, bundles compliance and did not involve any change in discharge policies or administrative policies. Patient base line demographics, including risk factors, severity score and all-cause mortality at 30 days were recorded. Descriptive analysis was done. Between groups comparison was performed by applying student’s T test, significance was assumed at a value of p < 0.05. Financial implication was derived based on average cost per ICU bed per day.

**Results:** Total of 2053 and 2432 patients were admitted with ICU days being 6460 and 6193 pre and post eICU respectively. Baseline demographics and patient profile in two groups did not show statistically significant difference. Mean APACHE II was 16.48 ± 6.81 [SMR (standardized mortality ratio) 0.71 ± 0.14] vs 16.82 ± 7.12 (SMR 0.49 ± 0.16). ALOS was 3.14 ± 2.39 vs 2.55 ± 2.45 days pre and post eICU respectively indicating a reduction of ~ 19% which is significant (p < 0.01). Nosocomial infection incidence per thousand device days post eICU came down from 16.4 to 6.31 (decrease of ~ 62%). Financial impact from reduced ALOS was INR 28.7 million which is significant compared to the eICU cost borne by the unit of INR 2.4 million.

**Conclusions:** eICU has been judged as a cost centre by most administrators but if we look at the above case study then it’s clear that in developing country like India not only it’s cost effective but also made a significant decrease in ALOS along with mortality and nosocomial infection. This leads to a direct impact of financials on patients and the hospital. By mobilizing shorter turnovers we can help in treating more patients without escalating cost by building new beds which will never have adequate manpower and resources.

**Reference**

1. D. McCarthy et al.: Committed to Safety: Ten Case Studies on Reducing Harm to Patients, The Common Wealth Fund, April 2006

## P427 Predicting deterioration in general ward using early deterioration indicator

### E. Ghosh, L. Yang, L. Eshelman, B. Lord, E. Carlson

#### Philips Research North America, Cambridge, USA

**Introduction:** Deterioration of patients in hospitals is typically preceded by changes in vital signs. Deterioration detection based on changes of a single vital sign can result in missed deterioration events and high false alarm rates. Multi- parameter systems such as the modified and national early warning system (MEWS and NEWS) use look up tables to score data from multiple vital signs and specify actions based on the score. These systems offer improved performance compared to single-parameter systems, but were created through physician consensus and may not be making optimal use of the available data. Here we explore use of modern data mining techniques to derive a high performance multi- parameter deterioration detection algorithm, the Early Deterioration Indicator (EDI).

**Methods:** EDI was created by producing risk curves for every input feature. Every feature value is mapped to a risk score which are added together to get the final score. Input features for EDI were: heart rate, systolic blood pressure, respiration rate, oxygen saturation, temperature and age. To derive and validate EDI, we collected data from 14282 general ward stays. 11864 stays were used to develop the algorithm. We tested the algorithm on the other 2418 stays. EDI, MEWS and NEWS were calculated in the middle of general ward stay for non- deteriorating stays. For patients who deteriorated, middle values in the last 6 hours before deterioration were used to calculate the scores. We computed sensitivity, specificity values and the area under the curve (AUC) for the receiver operator characteristic curve for the 3 scores and compared their performance.

**Results:** We tested the ability of EDI, MEWS and NEWS to predict deterioration which was defined as patient death or transfer to a higher level of care such as ICU. The training dataset comprised of 882 deteriorations (7.4% prevalence). In the training data, the AUC values were- EDI: 0.7793, NEWS: 0.6764, MEWS: 0.6404. The test dataset comprised of 184 deteriorations (7.6% prevalence). AUC values were- EDI: 0.7655, NEWS: 0.6569, MEWS: 0.6487.

**Conclusions:** EDI was developed using a data-driven approach to discover patterns in vital sign values that are predictive of imminent patient deterioration. It is based on continuous-value risk curves that are sensitive to subtle variations in vital signs, and may be automated for real- time risk assessment. EDI significantly improves performance over commonly deployed systems (MEWS and NEWS) while making use of the same input parameters, offering the possibility of a drop-in solution to reduce alarm fatigue.

## P428 High impact enhanced critical care outreach - the imobile service: making a difference

### E. Helme, R. Broderick, S. Hadfield, R. Loveridge

#### King’s College Hospital NHS Foundation Trust, London, UK

**Introduction:** King’s College Hospital is one of the largest acute and tertiary referral hospitals in the UK and plays a pivotal role in King’s Health Partners, an AHSC.

In 2013, the hospital set up a multi-disciplinary 24/7 enhanced critical care outreach service to support patients, relatives and staff providing: Rapid response & stabilisation; critical care follow-up; outreach education; and delivering virtual critical care beds when critical care flows exceed capacity.

**Methods:** Process and hospital outcome data was collated by our business intelligence unit and the National Cardiac Arrest Audit (NCAA). Trends in hospital outcomes & cardiac arrests were analysed using linear regression and contingency tables.

**Results:** The service saw 6046 patients in its first year which represented 12.2 % of the hospital admissions. The mean number of reviews per patient ranged from 3.25 for discharges to 13.5 for patients undergoing critical care interventions making 24,000 clinical episodes annually. The ‘dose’ was 82/1000 admissions, with only 8.8 % of calls inappropriate, and this exceeds the minimum dose recommended by the Institute for Healthcare Improvement.

The median time to review after activation was 14 minutes.

Hospital admissions and acuity rose during the study period by 11.2 % as measured by nationally set metrics and there was a reduction in the actual cardiac arrest rate as against the forecast cardiac arrest rate based on local historical NCAA data (P < 0.021).

There was a significant difference in the trends for expected vs. observed deaths pre and post the introduction of the iMobile service (p < 0.043).

**Conclusions:** High impact, high dose, critical care outreach services can deliver the changes in outcomes that are difficult to evaluate in healthcare socio-cultural interventions.

**Fig. 105 (Abstract P428).**
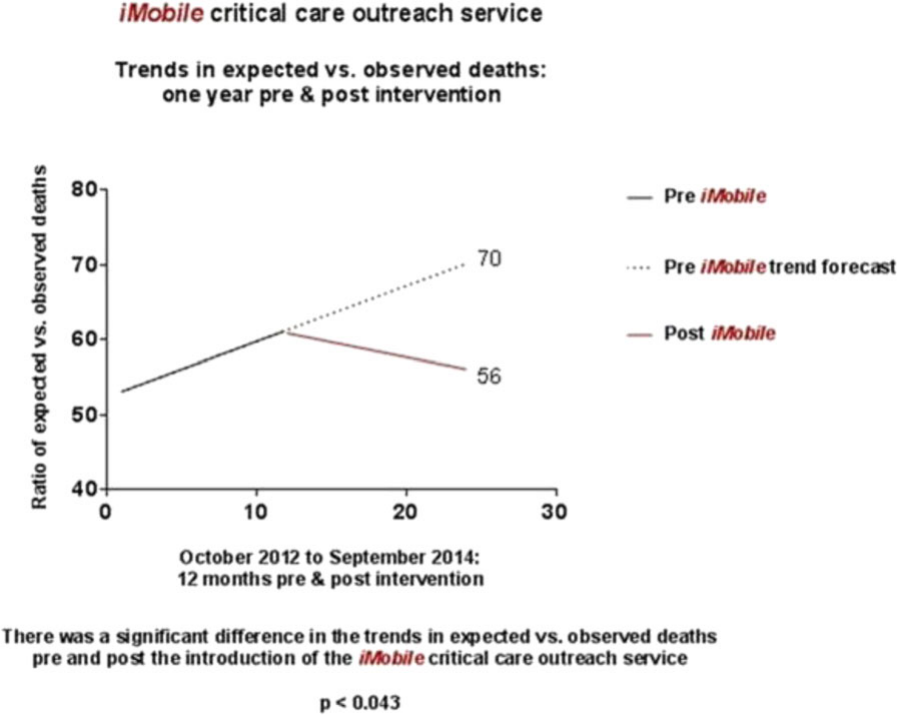
Trends in hospital outcomes

## P429 Impact of bed availability and cognitive load on intensive care unit (ICU) bed allocation: a vignette-based trial

### J. Ramos^1^, D. Forte^2^

#### ^1^Hospital Sao Rafael, Salvador, Brazil; ^2^Hospital das Clinicas, Sao Paulo, Brazil

**Introduction:** ICU bed scarcity is associated with reduced probability of ICU admission, but it is not clear if decisions to admit are subjected to heuristics and biases and to the impact of greater cognitive load.

**Methods:** Physicians were electronically invited to respond an online questionnaire. The study was a factorial 2x2 trial and each respondent was randomized to increased or no increased cognitive load and to different versions of the vignettes. Cognitive load was simulated by distracting videos, sounds and time-limit for response. Clinical vignettes were based on real patients for whom ICU admission was requested. Archetypical vignettes for ICU admission or refusal were defined based on experts opinion. Six groups of vignettes were constructed: 1)single archetypical for admission; 2)two simultaneous archetypical for admission; 3)single archetypical for refusal; 4)two simultaneous archetypical for refusal; 5)single non-archetypical vignette. Each of these groups were randomly presented in an ICU scarcity (last bed available) or non-scarcity setting. Group 6 vignettes were randomly presented in two versions: 6.1 consisted in a “multiple choice” vignette in which the respondent was presented with the decision to admit an archetypical case for ICU admission or an elective surgery patient; 6.2 consisted in a “status quo” vignette, in which the last ICU bed was already reserved for an elective surgery patient and the respondent was presented with two simultaneous ICU requests for archetypical cases for ICU admission.

**Results:** 159 physicians logged in and were randomized and 106 (67%) had complete data. Completion rate was lower for the cognitive load group (56% vs 79%, p < 0.05), but there were no differences on demographic characteristics. Cognitive load had no impact on the proportion of cases admitted or refused in each group. Group 3 vignettes were more often refused admission in ICU scarcity setting (68% vs 45%, p < 0.05). Archetypical vignettes for admission were more often refused in group 6.1 (multiple-choice) than group 6.2 (status quo): 20.4% vs 1.8%, p < 0.05.

**Conclusions:** There was no impact of cognitive load on the responses, but the results may have been hampered by the differential dropout rate. ICU scarcity was associated with increased refusal of patients for whom ICU admission was deemed inappropriate, but not with refusal of patients for whom ICU admission was deemed appropriate. Multiple-choice bias had more impact than status quo bias in the inappropriate refusal of ICU admission.

**Reference**

Sinuff et al.: Crit Care Med 2004; 32:1588-97

## P430 Characteristics of critically ill patients admitted through the emergency department

### F. Yang, P. Hou

#### Brigham and Women’s Hospital, Boston, USA

**Introduction:** Patients admitted through the emergency department (ED) to intensive care units (ICU) represent a unique population with varying etiologies of illness, for which existing descriptive analysis of severity and early prognosis of disease is lacking. Our study compares several ICU illness severity scores (Acute Physiology and Chronic Health Evaluation (APACHE)II, Simplified Acute Physiology Score (SAPS)II, Sequential Organ Failure Assessment (SOFA) score) within this cohort of patients during their early ED and ICU course to describe illness progression.

**Methods:** Retrospective review of consecutive patients admitted from the ED to the ICU in an academic referral center between January and February of 2014. Clinical and demographic data were collected to calculate APACHE II, SAPSII, SOFA scores on initial ED arrival (with exception of SAPSII), ED discharge, and after first 24 hours in the ICU. t-test used to evaluate differences between scores at each time interval, with p < 0.05 representing significance.

**Results:** We enrolled 82 patients, who averaged 62 years of age, 53% were female. 43% were admitted to medical, 39% to surgical, 17% to neurologic ICUs. Mortality rate was 13%. SOFA, APACHE II, SAPS II results summarized in Table 51. Comparison of scores by t-test showed significant increase in illness severity between ED arrival and end of first 24 hours in ICU (Table 52).

**Conclusions:** Illness scores (SOFA, APACHEII, and SAPSII) significantly increased at 24 hours after ICU admission compared to ED arrival. While a preliminary investigation, our data provides insight on expected progression of illness for ICU-bound patients admitted from the ED. Further work is needed in elucidating prognostic indicators and patient-care factors.

**Table 51 (Abstract P430).** ᅟ

Illness severity score	Mean value (±SD) Initial ED	Mean value (±SD) on ED discharge to ICU	Mean value (±SD) at 24 hrs after ICU admission
SOFA	2.29 (±2.62)	2.55 (±2.67)	3.52 (±3.07)
APACHE II	12.49 (±7.50)	13.65 (±8.03)	15.88 (±8.63)
SAPS II	N/A	30.92 (±13.78)	35.87 (±17.44)

**Table 52 (Abstract P430).** p-values from comparison of illness severity scores over early hospital course

Initial vs. ED discharge	ED discharge vs. ICU 24 hr	Initial vs. ICU 24 hrs	SOFA
0.54	0.031	0.006	APACHE II
0.30	0.075	0.007	SAPS II
N/A	0.045	N/A	

## P431 Admission to critical care: the quantification of functional reserve

### J. Dudziak^1^, J. Feeney^1^, K. Wilkinson^1^, K. Bauchmuller^2^, K. Shuker^2^, M. Faulds^2^, A. Raithatha^2^, D. Bryden^2^, L. England^1^, N. Bolton^1^, A. Tridente^1^

#### ^1^St Helens and Knowsley, Liverpool, UK; ^2^STH, Sheffield, UK

**Introduction:** Critical Care (CC) services are in increasing demand but published guidance for triaging admissions may no longer reflect current practice [1-2]. Exercise tolerance and clinical frailty assessment may have a role in assessing patients (pts) referred to CC [3]. We aimed to establish the impact of frailty and other factors on this decision making process.

**Methods:** Referrals to CC were prospectively enrolled in a review cohort. Data included patient demographics, a measure of acute physiological derangement (early warning score, EWS), prior hospital length of stay (LOS), exercise tolerance (ET), functional and dependence status, Canadian Clinical Frailty Scale (CCFS) and comorbidities. Logistic regression analysis was used to assess factors influencing admission, using STATA 14.1. Results are expressed as median (interquartile range) and odds ratios (OR) for admission with 95% confidence intervals (CI).

**Results:** Data was collected on 617 pts referred to CC between November 2013 and October 2014, of whom 344 (55.8%) were made out of hours, 279 (45.2%) were admitted. Median age was 65 (50-74) years, 311 (50.4%) were male and the median LOS prior to referral was 2 (0-2) days. The median CCFS, MET grading and EWS were 4 (3-6), 4 (0-6) and 4 (2-6) respectively. The majority of referrals came from the medical specialties (246 pts, 39.9%), directly from the emergency department (190 pts, 30.8%) and the surgical specialties (129, 20.9%). The most common comorbidities were cardiovascular (238 pts, 38.6%) diseases, respiratory (141 pts, 22.9%) and diabetes (108 pts, 17.5%). The most common referral reason was sepsis (140 pts, 22.7%), with post-operative monitoring accounting for 61 referrals (10%). At age and gender adjusted logistic regression analysis the EWS (OR per point 1.12, CI 1-1.25, p = 0.04), the CCFS (OR per point 0.79, CI 0.69-0.91, p = 0.001), ET > 30 yards (OR 2.5, CI 1.6-3.98, p < 0.001), self-caring status (OR 2.5, CI 1.6-3.97, p < 0.001) and housebound status (OR 0.38, CI 0.24-0.58, p < 0.001) influenced admission. The number of CC beds available, the LOS prior to referral and the MET grading did not.

**Conclusions:** Frailty, level of dependence and exercise tolerance appear to be major factors in decision making regarding admission to CC, even after adjusting for age. These factors may support decision making by allowing objective quantification of functional reserve.

**References**

1. ACCCM/SCCM task force. CCM;27:633-638.1999

2. ATS. AJRCCM;156:1282-1301.1997

3. LeMaguet P et al. ICM. 2014 May ; 40(5):674-82

## P432 Admission to critical care: the importance of frailty

### K. Bauchmuller^1.^, K Shuker^1.^, A Tridente^2.^, M Faulds^3.^, A Matheson^1^, J. Gaynor^1.^, D Bryden^1.^, S South Yorkshire Hospitals Research Collaboration^1^

#### ^1^Sheffield Teaching Hospitals, Sheffield, UK; ^2^Whiston Hospital, Prescot, UK,^3^Freeman Hospital, Newcastle upon Tyne, UK

**Introduction:** Demand for Critical Care (CC) is increasing but admission may be inappropriate if the patient is unlikely to survive. Consequently, published guidance may no longer reflect current practice [1-2]. Clinical frailty evaluation scores have recently been shown to be associated with increased mortality in patients already admitted to CC [3]. We aimed to establish the impact of frailty and other factors on the decision whether to admit a deteriorating hospital patient to CC.

**Methods:** Unplanned patient referrals were prospectively enrolled in a review cohort. Data included patient demographics, acute physiological parameters, prior hospital length of stay (LOS), Canadian Clinical Frailty Scale (CCFS) and comorbidities. Logistic regression analysis was used to assess factors influencing admission, using STATA 14.1. Results are expressed as median (interquartile range) and odds ratios (OR) with confidence intervals (CI).

**Results:** Between June and October 2015 data was collected on 220 patients referred to CC, of whom 87 (39.5%) were admitted. Median age was 66.5 (50-77) years, 134 (60.9%) were male and the median LOS prior to referral was 2 (0-7) days. The median SOFA score, CCFS and Charlson age-adjusted comorbidity index (CACI) was 5 (4-7), 4 (3-5) and 4 (2-6), respectively. At unadjusted logistic regression analysis age (p = 0.86), gender (p = 0.72), LOS (p = 0.34) and SOFA score (p = 0.42) did not impact on likelihood of admission to CC. Conversely, each unit increase in the CACI (OR 0.78, 95% CI 0.66-0.91, p = 0.002) and in the CCFS (OR 0.69, 95% CI 0.56-0.85, p < 0.001) caused a decrease of approximately 22% and 31% in the odds of admission, respectively. On age and gender adjusted multivariate logistic regression analysis CCFS retained its ability to predict admission to critical care (OR 0.65 per unit increase, 95% CI 0.49-0.85, p = 0.002), whilst CACI did not (OR 0.85, 95% CI 0.70-1.03, p = 0.09).

**Conclusions:** Frailty appears to be a major factor in decision making regarding admission to CC, even after adjusting for age, gender and other known comorbidities. Quantification of frailty using the CCFS may be a valuable tool in risk stratification and resource allocation, and merits further investigation in this context.

**References**

1. ACCCM/SCCM task force. CCM;27:633-638.1999

2. American Thoracic Society. AJRCCM;156:1282-1301.1997

3. LeMaguet P et al. Intensive Care Med. 2014 May ; 40(5):674-82.

## P433 Development of an instrument to aid triage decisions for intensive care unit admission

### J. Ramos^1^, B. Peroni^2^, R. Daglius-Dias^2^, L. Miranda^3^, C. Cohen^2^, C. Carvalho^2^, I . Velasco^2^, D. Forte^2^

#### ^1^Hospital Sao Rafael, Salvador, Brazil; ^2^Hospital das Clinicas, Sao Paulo, Brazil; ^3^Hospital Nove de Julho, Sao Paulo, Brazil

**Introduction:** Intensive care unit (ICU) admission triage is performed routinely in ICUs of developed and developing countries and is often based solely on clinical judgement, which could mask unethical prejudice or bias. Therefore, development of an objective triage score and of guidelines applying to individual patients has been discussed in the literature. To help triage decisions for ICU admission, a critical care team was assembled to come up with an instrument to aid triage decisions, based on the Society of Critical Care Medicine’s (SCCM) guidelines. We sought to evaluate the validity and reliability of this instrument.

**Methods:** An algorithm based on responses to an ICU request form was developed to classify patients in each of four SCCM’s priority strata. Nine physicians with experience in critical care or emergency medicine evaluated forty clinical vignettes for reliability analyzes. The correlation of algorithm-based priorities with surrogate gold standards, defined by a panel of specialists, was evaluated in a validity analysis. Validity was further evaluated by applying this algorithm to a retrospective sample of 603 patients with requests for ICU admission, for association with outcomes.

**Results:** Overall agreement was substantial, with a weighted kappa of 0.61 (95% CI 0.57-0.65). Algorithm-based priorities were also correlated with the priorities ascribed by specialists, with a weighted kappa of 0.67 (95% CI 0.60-0.74) and with subjective assessments of appropriateness of ICU admission. Algorithm-based priorities were also associated with ICU admission and hospital mortality (priorities 1,2, 3 and 4 had hospital mortality rates of 35.5%, 27.6%, 46.1% and 66.1%, respectively, P < 0.05). However, in an unadjusted analysis, there was no differential mortality benefit of ICU admission in each priority stratum.

**Conclusions:** This ICU admission triage tool demonstrated good reliability, validity and predictive characteristics. However, it may not be suitable to standard practice, yet, since it was not properly evaluated to demonstrate a difference in benefit of ICU admission, justifying the admission of one priority stratum over the others. Further prospective studies with additional collected variables should be implemented to address its limitations.

**References**

SCCM; Crit Care Med 1999; 27(3): 633-8

Sprung et al: Int Care Med 2013; 39 (11) 1916-24

## P434 Using selective serotonin re-uptake inhibitors and serotonin-norepinephrine re-uptake inhibitors in critical care: a systematic review of the evidence for benefit or harm

### J. M. Kelly^1^, A. Neill^2^, G. Rubenfeld^2^, N. Masson^3^, A. Min^4^

#### ^1^University Hospital Coventry and Warwickshire, Coventry, UK; ^2^Sunnybrook Health Sciences Centre, Toronto, Canada; ^3^NHS Scotland, Glasgow, UK; ^4^Royal College of Surgeons of Ireland, Dublin, Ireland

**Introduction:** SSRI/SNRIs are among the most commonly prescribed drugs in patients admitted to critical care. We reviewed the literature for evidence of benefit/harm from chronic use, continued use or withdrawal - to guide clinicians considering whether to continue, stop, or restart these medications on critical care.

**Methods:** Data Sources

4298 studies through November 2014 were identified using prespecified search terms in Medline, Embase and the Cochrane Central Register of Controlled Trials.

Study Selection

Abstracts were screened by predefined criteria, then 289 full-text articles assessed for eligibility. After applying prespecified exclusion criteria, only 14 studies were included in the systematic review (total 20048 patients - 14709 from one large study).

Data Extraction

Studies were analysed according to published quality criteria – cohort studies were awarded a Newcastle-Ottawa Score.

Data Synthesis

The low number of studies reviewed and their heterogeneity did not allow meta-analysis, so a narrative review was presented.

**Results:** We found few papers presenting useful evidence – five cohort studies, one case series and seven case reports. We found no evidence for benefit from use of SSRI/SNRIs in critical care. We found some evidence of increased morbidity in patients using SSRI/SNRIs on admission to critical care. However due to inadequate administration reporting it was often unclear if they were actually continued, and therefore whether harm was due to chronic effects, ongoing use, or drug withdrawal.

**Conclusions:** Overall there were inadequate data to draw firm conclusions. We feel there is sufficient uncertainty regarding the safety and utility of SSRI/SNRIs in critical care to warrant a randomized interventional study into continuing/withholding them on critical care admission.

**References**

1. Critical Care Medicine 2009, 37 (12 SUPPL.): A166

2. Chest. 2014 Apr;145(4):745-52.

3. Gastrointest Endosc. 2011 Jul;74(1):22-34.e1.

4. Heart Lung Circ. 2012 Apr;21(4):206-14

5. Chest. 2001 Feb;119(2):547-53.

## P435 Measuring adaptive coping of hospitalized patients with a severe medical condition:the sickness insight in coping questionnaire (sicq)

### E. Boezeman^1^, J. Hofhuis ^2^, A. Hovingh^2^, R. De Vries^3^, P. Spronk^2^

#### ^1^University Leiden, Leiden, Netherlands; ^2^Gelre Hospitals, Apeldoorn , Netherlands; ^3^VU University Amsterdam, Amsterdam, Netherlands

**Introduction:** The literature lacks a brief, specific, and validated instrument for measuring and monitoring adaptive coping of hospitalized patients with a severe medical condition. Hence, we introduce the Sickness Insight in Coping Questionnaire (SICQ) and examined its validity and patient-proxy agreement.

**Methods:** Study 1 (n = 103 hospitalized patients) addressed the internal consistency, initial factor structure, and construct validity, of the SICQ. Coping subscales of the BRIEF COPE, Illness Cognition Questionnaire, and Utrecht Coping List, were used as comparator measures in testing the construct validity of the SICQ-subscales (fighting spirit, toughness, redefinition, positivism, non-acceptance). Study 2 (n = 100 ICU-patients and close family members of ICU-patients as proxies) addressed the structural validity of the SICQ with confirmatory factor analyses, and examined patient-close proxy agreement with correlation and Bland-Altman Plot analyses.

**Results:** Study 1 showed that the SICQ has adequate internal consistency (0.64 [<=] α [<=] 0.79), a clear initial factor structure, and adequate convergent (0.24 [<=] r [<=] 0.50) and divergent (r [<=] 0.12) construct validity. Study 2 showed that the SICQ has good structural validity, and moderate (r = 0.37; non-acceptance) to strong (r > 0.50; fighting spirit, toughness, redefinition, and positivism) patient-close proxy agreement.

**Conclusions:** Overall, the SICQ has good psychometric properties. ICUs can use the SICQ to gain insight in adaptive coping style of patients through ratings of close family members.

## P436 Results of a national survey regarding intensive care medicine training

### G. Cabral-Campello, I. Aragão, T. Cardoso

#### Hospital de Santo António, Oporto Hospital Center, Porto, Portugal

**Introduction:** In Portugal training in intensive care is done after the achievement of a primary specialty. This is about to change with a proposal to create a primary intensive care medicine (ICM) specialty. A national survey was done to determine intensive care practitioners’ opinion on how to train in ICM.

**Methods:** Questionnaires were sent to all intensive care practitioners. Models of intensive care training were described as: primary specialty with direct entry following primary medical qualification; sub-specialty of another discipline; supra-specialty permitting multidisciplinary access from a range of base specialties.

**Results:** The team sent 391 questionnaires and obtained 240 answers (61%). Responders had 46 ± 10 years, 52% are male and worked in intensive care for 10(4-19) years, 86% worked in full time, of those working in shared schedule (n = 33) only 16 dedicated < 50 % of their time to ICM. Primary specialty of the responders was: Internal Medicine (67%), Anaesthesiology (24%) and other (9%). Most of them (73%) had previous experience in their primary specialty before initiating Intensive Care practice, during 2(1-5) years. When questioned about the ideal model 45% (n = 106) choose a “primary speciality” and of those: 69% thinks that training should be 5 years; 90% consider important a common core with Internal Medicine (92%), Anaesthesiology (92%), Emergency Medicine (89%), Cardiology (83%) and others (7,4%); for 81% the remaining training should be entirely in ICM and of these 95 % think that training in a dedicated ICU should be considered, mainly in neuro-ICU (93%) and RED-ICU (61%). For those who support ICM as a supra-specialty (51%,n = 121) or sub-specialty (28%,n = 65) the main reasons pointed were: previous specialty facilitates acquisition of ICM knowledge (97%), increases clinical autonomy (97%), provides a higher capacity in clinical judgment and medical decisions (90%) and allows more firm bioethics knowledge (80%); it is an advantage having a multidisciplinary team (95%). Regarding the training program: 77% knows the CoBatriCE project; 89% knows the PACT and 95% finds it usefull. Technical competencies found to be desirable in the ICM training were: Echocardiogram (97%), Bronchoscopy (97%), Fast Eco (97%) and others (30%). Undergraduate teaching was reported as desirable by 83% (n = 193).

**Conclusions:** Good response rate, less than half of the responders think that ICM should be a primary specialty, the vast majority thinks that an intensive care team should be composed by consultants from different specialties.

## P437 Work engagement among healthcare professionals in the intensive care unit

### M. Van Mol^1^, M. Nijkamp^2^, E . Kompanje^1^

#### ^1^Erasmus MC, Rotterdam, Netherlands; ^2^Faculty of Psychology and Educational Sciences, Heerlen, Netherlands

**Introduction:** Work-related stress with the accompanying emotions provoked specifically in the Intensive Care Unit (ICU) is well documented over the previous years. The high-stakes, high stress environment that ICU health care professionals (HCP) practice in, are demanding intellectually, physically, and emotionally. Work engagement has been operationalized as a positive work-related state of mind and characterized by vigor, dedication, and absorption. The aim of the study is to explore the influence of personal resources, e.g. empathic ability on work engagement among the ICU HCP.

**Methods:** The design of the study was a cross-sectional survey study among ICU HCP (i.e. intensivists and nurses) of Erasmus MC, a university hospital in the Netherlands. A digital link to the questionnaire was sent in October 2015 to a given e-mail address of 163 nurses in a mixed ICU, 45 nurses in a thoracic ICU, 54 nurses in a cardiac ICU and 53 intensivists. Two reminders were sent. Work engagement was measured with the Utrecht Work Engagement Scale, which includes 17 items five point Likert items about how a person feels at work. Additionally, 14 items based on the Jefferson Scale of Physician Empathy measured emphatic ability.

**Results:** The overall response rate was 66%, with a male-female ratio of .47. The mean age of the respondents was 43.4 years, mean working hours/weeks rated 33.6, and 54% finished A-levels in education. ICU HCP scored the same on vigor, higher on dedication (p < .05), and lower on absorption (p < .05) compared to the average Dutch employee. They reported no more stress related symptoms such as mental distance and sleeping disorders. Although only 2.8% of the ICU HCP assessed workload as high, compared to 3.6%, the emotional burden scored higher (3.1 and 1.8 in respective, p < .05). General HCP acted as benchmark in empathic ability. ICU HCP showed an equal score on the cognitive component (3.9 compared to 4.0) and a lower score on the emotional component (3.0 compared to 3.8, p < .05). Mean cognitive empathy is positively correlated with vigor (r = .21) although both cognitive and emotional empathy did not correlate with work engagement.

**Conclusions:** Workload at the ICU may be high, but seemed not too hard for the respondents. It is assumable that workload, with the emotional high burden, is acknowledged as an integral part of ICU work. ICU HCP understand the patients and relatives, but remain at a certain distance emotionally. The results of this study suggested no influence on work engagement by empathic ability.

## P438 Empowering the intensive care practitioners. is it a burnout ameliorating intervention?

### P. Ostrowski^1^, A. Omar ^2^

#### ^1^University of Toronto at Scarborough, Scarborough, ON, Canada; ^2^Hamad medical corporation, Doha, Qatar

**Introduction:** Burnout is a rapid resurgent worldwide problem; intensive care unit (ICU) practitioners are particularly at risk. Leader-empowering behavior could reduce the encountered job tension and enhancement of work effectiveness [1]. We aim to assess the role of empowerment on ICU practitioners’ burnout.

**Methods:** Mixed qualitative and quantitative methodology was used in a cross-sectional descriptive survey. The case study focused on Qatari ICU where anonymous questionnaire survey was presented to the staff members including physicians, nurses and respiratory therapists who work as full time. We used two main instruments, Maslasch Burnout scale (MBI-HSS), and Empowerment scale (ES). High burnout is considered when MBI-HSS is scores of 27 and more [2].

**Results:** Two-hundred healthcare practitioners were enrolled in mixed medical and surgical ICUs. Fifty-one ICU practitioners showed high prevalence association (25.5%). Physicians, nurses, and respiratory therapists were equally presented at burnout association (p = 0.19). Age was associated with high burnout (p = 0.000), while gender was not. Empowerment was significantly associated with burnout by two-tailed test (p = 0.006). Positive empowerment had a negative effect on burnout variance (3.8%) when speculating practitioners’ burnout.

**Conclusions:** Empowerment components could reduce burnout within ICU. Particular attention should be given to the high burnout in ICU settings.

**References**

1. Laschinger HKS, Wong C, McMahon L & Kaufmann C. Leader behavior impact on staff nurse empowerment, job tension, and work effectiveness’. J Nurs Adm.1999;29 (5), 28-39.

2. Maslach C, Jackson SE & Leiter MP. Maslach Burnout Inventory Manual. Palo Alto, California. 1996; 3rd Ed

## P439 Icu patients suffer from circadian rhythm desynchronisation

### K. Kiss ^1^, B. Köves^2^, V. Csernus^3^, Z. Molnár^1^

#### ^1^University of Szeged, Szeged , Hungary; ^2^Jahn Ferenc Hospital, Budapest, Hungary; ^3^University of Pécs, Pécs, Hungary

**Introduction:** Circadian rhythm has a profound role in harmonising the function of organs and cells of the human body, while disruption of this rhythm (i.e. chronodisruption) is accompanied with a wide spectrum of pathological conditions. This rhythm in healthy subjects is mainly orchestrated by the daily cycles of light and darkness, as shown by the elevation of melatonin (MEL) levels peaking at dawn [1]. The aim of our prospective clinical study was to examine the rhythm of MEL release in ICU patients suffering from continuous light pollution.

**Methods:** Mechanically ventilated patients over 18 years, without brain injury were enrolled into the study in January 2015. Illuminance was measured by a lux meter over the patients’ body and serum MEL levels were determined from blood samples. Measurements took place after admission starting with the closest time point of the regular sampling period of 01:00, 07:00, 13:00 and 19:00 hours with a precision of ±10 min. for 4 days. Blood samples were kept in total darkness until clotting before being centrifuged 8 min. at 3000 rpm, then serum samples were placed into -80 °C until MEL measurement was performed.

**Fig. 106 (Abstract P439).**
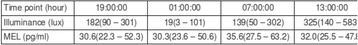
Data are presented as median (IQR)

**Results:** 15 patients were enrolled on a multidisciplinary ICU, with age of 63±9 years, male/female ratio was 9/6. Out of the 16 measurements/patient, 240 MEL samples were analysed. MEL and illuminance levels are summarised in Fig. 106. Although illuminance was the lowest at 01:00, but levels were still a lot higher than physiologically required. Furthermore, at 01:00 a MEL peak should occur [1], which was also missing. A repeated correlation analysis between MEL serum concentration and illuminance levels showed a weak within-subject correlation (r = -0.097, P = 0.152), and a still non-significant between-subject correlation (r = 0.438, P = 0.103).

**Conclusions:** Our results indicate that circadian rhythm is desynchronized in ICU patients, as indicated by a) the missing MEL peak during the night; b) by the poor correlation between illumination and MEL levels c) the absence of the normal 24h rhythm of MEL release. Future trials are hence warranted to test whether providing physiological light/dark conditions could resynchronise circadian rhythm and if it has any effect on patient outcomes.

**Reference**

1. Hardeland et al. Progress in Neurobiology (93) 350-384.(2011)

## P440 Noise reduction in the ICU: feasible ?

### Y. Hoydonckx, S. Vanwing, B. Stessel, A. Van Assche, L. Jamaer, J. Dubois

#### Jessa Ziekenhuis, Hasselt , Belgium

**Introduction:** Noise is an underestimated problem in the ICU that can attribute to sleep disturbance and delirium with an adverse effect on morbidity and mortality in critically ill patients.

WHO recommendations of average sound levels below 35 dBA are not achievable in an ICU, where 50% of the time average sound levels exceeding 52 dBA are reported [1].

Since noise is also in our ICU considered a problem, we investigated sound levels and its causes and tried to implement a bundle of measures for noise reduction.

**Methods:** We placed a sound level meter (Amptec 10EaZy RT) at two places in the ICU: one bedside in a two-bed room and one at the nursing station. Recordings were performed at each location continuously for a 24 hrs period.

Simultaneously, structured observations were performed to identify the causes of elevated sound levels.

After identifying the mean causes of noise and implementing a bundle of measures for noise reduction, the recordings and observations were repeated.

**Results:** Average sound levels were comparable both bedside and at the nursing station before and after the noise reduction measures (Bedside: 52.8 dBA vs 52.9 dBA at night and 54.6 dBA vs 53.7 dBA at daytime – Nursing station: 52.6 dBA vs 52.2 at night and 53.9 dBA vs 53.2 dBA at daytime).

There was also no difference in the incidence of sound level peaks (Bedside: 14 vs 16 - Nursing station: 11 vs 10).

However, the maximum sound level peak bedside was significantly reduced (101.1 dBA vs 91.6 dBA).

Further, observations showed that operational sounds and not structural sounds are the main cause for noise in the ICU. In particular, the ICU-team (nurses, doctors, …) seemed to be the main contributing factor.

**Conclusions:** Reduction of noise in the ICU is complex and multimodal. Reaching WHO levels seems not feasible in a ICU setting since measures for noise reduction can temper peaks but not the average sound level.

Since the ICU-team is the main contributing factor, behavioural guidelines play an important role in noise reduction.

**Reference**

1. Darbyshire et al. Critical Care 17:R187, 2013.

## P441 Accidental removal of invasive devices in the critical patient into the bed-washing. does the presence of professional nurse modify his incidence?

### V. Medo, R. Galvez, J. P. Miranda

#### Hospital Clinico Universidad de Chile, Santiago, Chile

**Introduction:** In the humanized care in the critical patient, the bed-washing is an internalized activity with impact over the risk involved for the accidental removal of invasive devices, and our intensive care unit it is not exempt from this.

It was modified the bed-washing protocol, in order to measure the impact of direct supervision and professionalized nursing intervention during the bad-washing in relation with the incidence of accidental removal of invasive devices.

**Methods:** The incidence of accidental removal of invasive devices (orotracheal tube, arterial catheter, central venous access, surgical drainage and indwelling urinary catheter) were reviewed of the intensive care unit (ICU) of the Hospital Clínico Universidad de Chile doing the difference between the totals events of those into the bed-washing in our patients. It was coordinated a continuous medical education to nursing team (emergency and intensive nurses, technicians of nurses and auxiliaries) modifying the bed-washing protocol, including the direct supervision and nursing intervention in critical patients with invasive devices. It was defined two periods for comparison (2003-2005 and 2012-2014). The incidents rates were analyzed for the study period by Kwallis nonparametric test. Significance level of p < 0.05.

**Results:** The incidents rates of accidental removal of invasive devices were reviewed of ICU through the database nursing department of our hospital. The period of diagnostic described 76 events of accidental removal of invasive devices; 13 into the bad-washing (rate: 1.18 adjusted events per year). In the evaluation period, 60 events are reported and 7 during the bed-washing (rate: 0.64 adjusted events per year). The rates showed significant differences in the totals events (p = 0.02). To compare rates of bed-washing events, a significant difference was observed in relation to the change of protocol (p = 0.04).

**Conclusions:** A decrease was evident in the overall incidence of the accidental removal of invasive devices in liaison to continuous medical education (p = 0.02). The same is observed on the incidence during the bed-washing, between the period of diagnostic and evaluation of the intervention (p = 0.04).

The presence of nursing into the bed-washing decreases significantly the rates of the accidental removal of invasive devices. We believe these data validate the implementation of our bed-washing protocol.

## P442 Deprivation of liberty safeguards (dols): audit of compliance in a of a 16-bed specialist cancer critical care unit.

### C. Stone, T. Wigmore

#### Royal Marsden Hospital , London, UK

**Introduction:** The aim of this audit is to investigate the compliance of a critical care unit (CCU) with the application of Deprivation of liberty safeguards (DoLS) authorisations. DoLS form part of the UK’s Mental Capacity Act and are intended to safeguard the rights of patients who lack mental capacity, and whose freedom to leave a healthcare environment is potentially limited.[1] They require written application to the local authority in order to gain permission for the patients on going care. However, because of the fact that this situation potentially applies to some patients in the CCU, an unintended consequence of the legislation is that it is increasingly being applied to critically ill patients. This can result in a significant workload for both the critical care team and the local council.

**Methods:** A search was made on the Phillips ICIP electronic record over a 10-month period (19th Dec 2014 - 8th Sept 2015) for the number of patients who had a mental health assessment form completed, declaring them as lacking capacity. A detailed search of their notes on the electronic patient record (EPR) was made in order to look retrospectively at the number of DoLS applications made in comparison to the number required.

**Results:** Out of a total of 994 admissions, 34 patients lacked capacity at some point during their stay. These were divided into emergency medical (56%), elective surgical (35%) and emergency surgical admissions (9%) respectively. Of 31 patients who were retrospectively deemed eligible for a DoLS, 7 (23%) had an application submitted to the local council. The reasons why authorisations were necessary were divided in to tracheostomy related (45%), chemical/physical restraint for delirium (39%) and other best interest decision (16%). On average a DoLS authorisation was necessary for 4.5 days. The local authority denied no applications. The average time between identifying a lack of capacity and completion of DoLS application was 5.2 days.

**Conclusions:** A significant number of patients admitted to CCU who lack mental capacity may be at risk of having their liberty deprived. A compliance rate of 23% may be a reflection of the way DoLS can now be applied to CCUs. Local interventions, such as routine pre-consent and streamlining of the DoLS application process are being implemented with the intention of reducing work load and improving the rate of compliance with the law.

**Reference**

1. M. Crews et al. Journal of Intensive Care Society. Volume 15: 4. 2014.

## P443 Use of a modified cristal score to predict futility of critical care in the elderly

### Y. Arunan, A. Wheeler, K. Bauchmuller, D. Bryden

#### Sheffield Teaching Hospitals, Sheffield, UK

**Introduction:** The CriSTAL checklist^1^ is a tool to identify the elderly, dying patient in hospital, aiming to help avoid aggressive, futile treatments and initiate appropriate plans for end of life care. We aimed to assess whether a version, modified for our department and institution, would help identify those elderly patients who may benefit from critical care.

**Methods:** Unplanned critical care admissions in patients aged over 70 years between January and March 2014 were retrospectively analysed. Validated predictors of short and medium term death chosen from the CriSTAL checklist were assigned a score of 1(present) or 0(absent) with a maximum of 15:

Chronic kidney disease (CKD [>=]3), Previous myocardial infarction, Abnormal ECG at presentation, Congestive cardiac failure, Chronic pulmonary disease, Cerebrovascular disease, Dementia/cognitive impairment, Long term institutionalised mental health, Chronic liver disease, Solid tumours, Hospital stay >5days before critical care review, Previous hospitalisation in past year, Previous ICU admissions, Marked weight loss in last 12 months and Frailty (Rockwood scale [>=]4)^2^.

The sum score for survivors and non-survivors was compared. Statistical analysis was performed via Mann-Whitney U test(SPSS) and values expressed as median(range).

**Results:** Of 125 patients with a median age of 79 (70-93), 42 (34%) were female, 83 (66%) male and 56 (45%) received level 3 and 69 (55%) level 2 care.

Median modified CriSTAL scores were higher in non-survivors (3.0) compared with survivors (2.0) with a p-value of 0.001.

This difference was maintained in the subgroups of level 3 (survivors 2.5, non-survivors 3.0; p-value 0.024) and level 2 patients (survivors 2.0, non-survivors 3.5; p-value 0.041).

**Conclusions:** Absolute CriSTAL score values, as assessed with our simplified tool, were low across all groups. The signal for increased mortality with higher scores may suggest use in informing treatment stratifications in elderly critically ill patients. Identification of the most appropriate individual parameters and their relative contributions towards an overall score merits further evaluation.

**References**

1. Cardona-Morrell M et al. BMJ Supportive and Palliative Care 2015;0:1-13.

2. Rockwood K et al. CMAJ 2005;173(5):489-495.

## P444 Improvement of Referral Rate to Palliative Care for Patients with Poor Prognosis in Neurosurgical Intensive Care Unit

### Y. Wong, C. Poi, C. Gu

#### Tan Tock Seng hospital, singapore, Singapore

**Introduction:** There is increasing evidence that a well-structured palliative care initiative in the form of an integrative or consultative or a combination of both into standard ICU care can provide benefits for patients and families.

Recent systemic reviews suggest that proactive palliative care in the ICU decrease hospital and ICU length of stay.

**Methods:** We embarked on a first of a kind ICU-Palliative Collaboration and Project in Singapore. The aim was to improve referral rate to palliative care for patients wih poor prognosis in Neurosurgical Intensive Care (NICU).

Poor Prognosis was defined as patients with at least one of the following referral criteria:

1. Hypoxic Ischaemic Encephalopathy
2. Severe Head Injury with poor neurological prognosis
3. Extensive Intracerebral/Subarachnoid Haemorrhage
4. Low presenting Glasgow Coma Scale (GCS) of less than 6

A multi-disciplinary team, consisting of intensivists, neurosurgeons, medical social workers,nurses and palliative care medicine physicians formed the core team to lead this project in NICU.

Interventions include

1. A structured screening tool that is easily available to the nurses
2. A referral criteria that is agreed upon by all involved in care of patient
3. Communication workshop conducted for all nurses and physicians in NICU to broach end of life care issues with family
4. Weekly palliative rounds in NICU to discuss current cases and to debrief previous patients referred.

**Results:** 1. The number of pallaitive care referrals, increased from a baseline of 30% to 85%.

2. Patients relatives when interviewed, had a higher level of satisfaction with overall medical care in hospital.

3. There was a 1.3 day reduction of ICU stay for patients referred to palliative care, with its attendant cost savings.

**Conclusions:** Palliative care in the ICU is an important part of management of patients with poor prognosis. It improves family emotional outcomes abd reduces ICU length of stay

**Reference**

IPAL-ICU consensus Group. Crit Care Med 2010

## P445 Factors associated with limitation of life supporting care (lsc) in a medico-surgical intermediate care unit, and outcome of patients with lsc limitation: a monocentric, six-month study.

### P. Molmy, N. Van Grunderbeeck, O. Nigeon, M. Lemyze, D. Thevenin, J. Mallat

#### Centre Hospitalier de Lens, Lens, France

**Introduction:** Limitations of Life Supporting Care (LSC) and Withdrawal of LSC are scarcely studied in Intermediate Care Units (IdtCUs). We report prevalence of LSC limited patients in a medico-surgical Intermediate Care Unit over a six month period, with description, outcomes, and patterns of LSC Limitation and Withdrawal for such patients.

**Methods:** Monocentric, retrospective observational study in the IdtCU of a 500-bed general hospital. Data were extracted from patients’ files and included age, gender, admission diagnoses, Knaus index, Charlson comorbidity score, severity scores (SAPS2 and SOFA), LSC treatments, IdtCU and hospital mortality / length of stay, LSC withdrawal.Factors associated with LSC limitation of LSC were determined through univariate and multivariate analysis.

**Results:** Out of 405 patients, 79 (19,5%) patients were admitted with LSC limitation in the IdtCU. They presented with higher chronic and acute severity scores than unlimited patients. The most frequent admission diagnosis of LSC limited patients was acute respiratory failure (51,8% of patients). Non Invasive Ventilation (NIV) use was frequent within this population (39%). Hospital mortality for LSC limited patients was high (53%) and associated with growing age -OR = 1,09 (1.02 - 1.15), preexisting organ failures or comorbidities -OR 7,34 (1.94 - 27.81)- elevated SOFA score -OR 1,36 (1.03 - 1.8)- and hypoxemic respiratory failure –OR 24,8 (3.52 – 175.3). Withdrawal of LSC was proceeded in 19, 5% of cases, with frequent terminal sedation with or without NIV retrieval (43, 8%).

**Conclusions:** Patients with limitations of LSC are frequently admitted in IdtCU. Hospital mortality is high and associated with age, acute and preexisting organ failures, and hypoxemic respiratory failure. Life support withdrawal includes NIV terminal weaning and palliative sedation. LSC limited hypercapnic patients appear to be safely treated with NIV. The very high mortality of most severe patients raises questions on appropriateness of care and use of acute care resources.

**References**

Nava S, Sturani C, Hartl S, magni G, Ciontu M, Corrado A (2007) End-of-life decision making in respiratory intermediate care units: a European survey. Eur Respir J 30: 156-164.

Nasraway SA, Cohen IL, Dennis RC, et al. Guidelines on admission and discharge for adult intermediate care units. American College of Critical Care Medicine of the Society of Critical Care Medicine. Crit Care Med. 1998 Mar;26(3):607-10.

## P446 Palliative care consultation and intensive care unit admission request: a cohort study

### J. Ramos^1^, M. Correa^2^, R. T. Carvalho^2^, D. Forte^2^

#### ^1^Hospital Sao Rafael, Salvador, Brazil; ^2^Hospital das Clinicas, Sao Paulo, Brazil

**Introduction:** It has been suggested that clinical deterioration during hospitalization could be seen as a trigger for palliative care (PC) consultation. The co-occurrence of urgent intensive care unit (ICU) admission request and PC consultation request was analyzed in hospitalized patients.

**Methods:** All urgent ICU admission and palliative care consultation requests at an academic tertiary hospital from May, 2014 to May, 2015 were evaluated. Patients were analyzed in four different groups: 1) ICU request without palliative care consultation (ICUnoPAL), 2) ICU request and palliative care consultation (ICUandPAL), comprising those patients with 3) palliative care consultation before ICU admission request (PALbefICU) and those with 4) palliative care consultation after ICU admission request (PALaftICU).

**Results:** There were 2476 ICU admission requests and 949 palliative care consultation requests. Of the formers, 179 (7%) were ICU and PAL. These patients, when compared to ICUnoPAL, were older (65 vs 54 years, p < 0.05), with more severe disease, as measured by MPMII0 score (0,40 vs 0,26, p < 0.05), less likely to be admitted to the ICU (49% vs 64%, p < 0.05) and had higher adjusted hospital mortality (83% vs 37%; OR [95%CI]: 7,2 [4,6-11,4]). There were 29 (16%) patients PALbefICU and 150 (84%) PALaftICU. When comparing these two groups, there were no differences in MPMII0 score, organ dysfunctions or use of artificial life support at the moment of ICU request. PALbefICU patients were hospitalized for fewer days before PC consultation request (8 vs 29 days, p < 0.05), were more likely to have no diagnosis at the moment of consultation (10% vs 0%, p < 0.05) and presented less frequently with palliative performance scale (PPS) lower than 30 (56% vs 85%, p < 0.05). More patients had PC consultations discontinued in the PALbefICU group (41% vs 20%, p < 0.05) and the main reason for discontinuation was the absence of indication for exclusive palliative care (31% vs 6%, p < 0.05). However, PALbefICU patients were less likely to be admitted in the ICU (31% vs 53%, p < 0.05) and hospital mortality was similar in both groups (88% vs 82%, p < 0.05), both adjusted for severity of illness.

**Conclusions:** Co-occurrence of ICU admission request and PC consultation in the same hospitalization was frequent and PC consultation before ICU request was associated with a lower chance of ICU admission. These patients had extremely high hospital mortality, despite differences in timing of consultation, diagnosis, PPS and definition of life support limitation. This data seems to support the notion that clinical deterioration during hospitalization may be seen as an opportunity to discuss end of life goals.

## P447 Nursing and medicine together in postsurgical intensive care unit: situations of prognostic conflict at the end of life. our critical care nurses suffer with our medical activism?

### A. Fernandez^1^, C. McBride^2^

#### ^1^Ntra Sra de Candelaria University Hospital, Santa Cruz de Tenerife, Spain; ^2^University of Texas at Austin, San Antonio, USA

**Introduction:** End-of-life (EOL) decision making in acute care is complex, involving difficult decision, such as whether to initiate or discontinue life support, place a feeding tube or a tracheostomy, or initiate cardiopulmonary resuscitation (CPR) in the event of a cardiac arrest.

**Methods:** our critical care nurses suffer when they think they are performing procedures that are harmful or of low efficacy and when their advocacy is ineffective. Prolonged, unrecognized suffering can be detrimental and can lead to disengagement and silence. Habitual silence or silencing in the face of perceived wrongs can result in permanent, deleterous changes in ethical values (1).

In recent studies researchers suggested that false optimism is associated with medical activism or a strong need for control over death, which is prevalent in the western world.. or ignorance or some emotional anesthesia present in us.

**Results:** Narratives enable those involved in the ICU to negotiate the meaning of critical care for the patient. Narrative functions as an interpretive procedure that allows diverse persons- we- nurses-patient, and patient’s families- to make sense of critical care.... and supplies a theory and instrument for negotiating meaning throughout the process that respects the realities and limitations of such care.

**Conclusions:** the effective practice of medicine requires “narrative competence.. the ability to acknowledge, absorb, interpret, and act on the stories and plights of others”.

The nurses that working with us every day are often caught in the middle-an ethically untenable position- as they attempt to comply with our medical directives and simultaneously protect and advocate for their patient.

**Reference**

1. Practice of Expert Critical Care Nurses in situations of Prognostic Conflict at the End of Life. Catherine McBride Robichaux and Angela P. Clark. Am J Crit Care 2006;15:480-491.

## P448 End of life who may decide

### E. Koonthalloor^1^, C. Walsh^1^

#### ^1^Beaumont Hospital, Dublin, Ireland

**Introduction:** to evaluate end of life practise in 20 bedded ’icu with neuro intensive care.

**Methods:** a prospective observational study of end of life decisions made by medical professionals and whether the patient, family, or legal representative were involved in the end of life decisions.

**Results:** 240 patients were admitted in ICU for a 3 month period of which 42 patients died in ICU (17.5%) 2 patients died in the ward following withdrawal of care.29(65.9%) of whom had died followed withdrawal or withholding of care.11(18% of the dead)had spontaneous cardiac arrest or `Brain Dead.all patients who had limitation of therapy had family consensus,of which 2 were autonomous decision of the patients.

**Conclusions:** the reasons for withdrawal of care was either unresponsive to maximal therapy or irreversible neurologic status,The optimal use of resources and the good practise of optimal care in ICU remains a challenge .Consnsus with the family remains a challenge despite good communication and well educated clientele.There is still scope for improvement in these areas.

**Reference**

Dying with dignity in icu New Eng journal june 2014

McDonagh JR, Elliott TB, Engelberg RA, et al. Family satisfaction with family conferences about end-of-life care in the intensive care unit: increased proportion of family speech is associated with increased satisfaction. Crit Care Med 2004;32:1484-1488

## P449 Correctly diagnosing death

### A. Webber, M. Ashe, K. Smith, P. Jeanrenaud

#### St Helens and Knowsley teaching hospitals, Liverpool, UK

**Introduction:** Around 50% of deaths in the UK occur in hospital, giving a total of around 250 000 in 2013 [1]. The responsibility of confirmation and documentation of these deaths rests across all specialties of hospital doctor. The Academy of Medical Royal Colleges (AMRC) released a consensus guideline in 2008 aiming to standardise this process [2]. Guidance is available for the diagnosis of death following cardiorespiratory arrest and death following cessation of brainstem function. The diagnosis should be transparent, consistent and instil public confidence in the process. This audit took a retrospective look at current practice and adherence to these guidelines.

**Methods:** A retrospective case notes examination was carried out between February and August 2015. 98 deaths were observed on Whiston ICU during this period. Doctors carrying out the reviews were not involved in the process of diagnosing death in order not to bias the study findings. Data was collected from entries made in the patient’s case notes and compared with recommended standards set out within AMRC guidelines. These include absence of heart and breath sounds, central pulse and corneal reflex. The patient should have been observed for 5 minutes and the time and date entered in the patient record, along with the name/grade of doctor.

**Results:** Of the 98 verifications of death examined, the % compliance was as follows:

Heart sounds 89.7, central pulse 82.6, respiratory effort 92.8, observed 5 minutes 9.1 (absent in 35 cases), pain response 72.1, pupillary response 89.7, corneal reflex 6.1, time correct 74.4, date correct 80.6, signed 97.9, printed name 97.9, grade 81.6. In the 63 cases where duration of observation was documented, mean time of was 2.1 minutes. There was only 1 instance of practice falling in line with current guidelines.

**Conclusions:** Despite AMRC consensus guidelines being available for best practice in diagnosis of death, there remains a great deal of variation in practice for this common procedure. Only 1% of the case notes examined met the guidelines, with the majority not observing the patient for long enough or documenting a corneal reflex. Furthermore, 20% of cases were found to have documented the time/date of death incorrectly. We propose that these wide variations in practice can easily be addressed with the introduction of a standardised proforma to be completed and signed by the clinician at the time of verification.

**References**

1. www.ons.gov.uk

2. AMRC: a code of practice for the diagnosis and confirmation of death 2008

## P450 Skin procurement performed by intensive care physicians: yes, we can

### A. Marudi , S. Baroni, F. Ragusa, E. Bertellini

#### Nuovo Ospedale Civile Sant’Agostino Estense , Modena , Italy

**Introduction:** Human deceased skin donor allografts represent a suitable and much used temporizing option for skin cover following any kind of skin injury. The main advantages for its use include dermoprotection while disadvantages of its use include the limited abundance of donors and availability of surgical retrival team. This paper will explore the role of skin procurement performed by intensive care physicians.

**Methods:** Data from skin procurement performed in Modena hospital were analyzed from 2006 to 2014. After attending Burn Care Unit in Cesena and a short training with a plastic surgeon who usually perfermed this kind of procedure, two intensive care physicians were able to perform skin retrival since 2011. Electric dermatome was used to remove skin. After the retrival skin grafts are sent to bank tissue. The activities of skin and tissues banks currently involve their recovery, processing, storage and distribution.

**Fig. 107 (Abstract P450).**
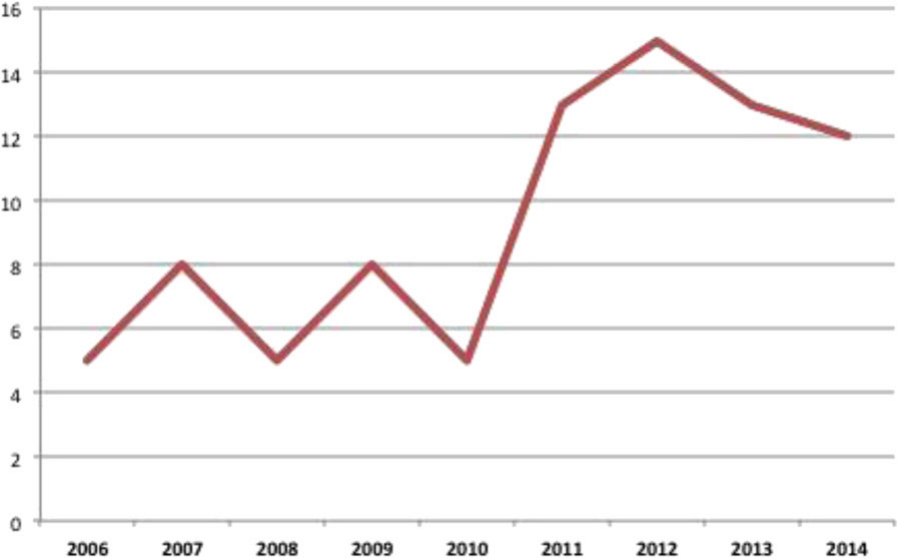
Skin procurement

**Results:** Human skin allografts were obtained from multi organ and tissue donors after both brain and cardiac death. During these nine years, 84 deceased donor skin allograft donations were performed. A significant improvement of numbers of skin procurement was observed from 2011 after the introduction of intensive care physicians as surgical team (Fig. 107). Quality and quantity of human skin allografts procured by intensivists were comparable to those procured by plastic surgeons.

**Conclusions:** The skin performs a variety of important functions for the human body. Deceased donor skin allografts is a feasible option for saving life of patient with extensive skin injury. Intensivists trained in human allografts procurement can implement donor pool.

**References**

• C. W. M. Horner • E. Crighton • P. Dziewulski Challenges in the provision of skin in the UK: the use of human deceased donor skin in burn care relating to mass incidents in the UK. Cell Tissue Bank (2013) 14:579–588 DOI 10.1007/s10561-013-9374-0

• Jorge Leon-Villapalos • Mohamed Eldardiri • Peter Dziewulski The use of human deceased donor skin allograft in burn care. Cell Tissue Bank (2010) 11:99–104 DOI 10.1007/s10561-009-9152-1

## P451 Death analysis in pediatric intensive care patients

### E. A. Volakli , E. Chochliourou, M. Dimitriadou, A. Violaki, P. Mantzafleri, E. Samkinidou, O. Vrani, A. Arbouti, T. Varsami, M. Sdougka

#### PICU, Hippokration General Hospital, Thessaloniki , Greece

**Introduction:** Mode of death in Pediatric Intensive Care Unit (PICU) could differ in different population and institutional setting. The aim of the present study is to investigate the mode of death in a single center.

**Methods:** Retrospective death analysis from 1/1/2012 to 31/12/2013 in a multidisciplinary PICU of Northern Greece. All deaths happened in pts stayed for > 4hours were recorded. Data collected: Demographics, reason for and severity score at admission, treatment characteristics, and laboratory data. Appropriateness of antibiotics was defined as pathogen susceptibility to at least one drug, given at least 24 hours before death. The mode of death was classified as death due to MODS, brain death (BD), and limitation of care (DL). P < 0.05.

**Results:** Twenty seven deaths (13 male/14 female), mean (±SD) age 53.12 ± 50.27 mo, were recorded in 231 admissions (11.68%). Eighteen pts died from MODS (66.66%), six from BD (22.22%) and three due to DL (11.11%). Pts died from MODS were significantly younger. All pts died due to DL were readmissions that suffered from co-morbidities. Severity score and lactate levels admission, and LOS didn’t differ significantly according to the mode of death. Although not significant, at the time surrounding death or after death, 10 positive blood cultures were revealed in 9 pts (8 in MODS); 4 acinetobacter b., 2 enterobacter c., 1 pseudomonas aer., 1 klebsiella p., 1 enterococcus f., 1 candida non al. Appropriate antibiotics were prescribed in 3 of them (33.33%).

**Conclusions:** We found that MODS were the main mode of death, following by BD and DL. Pts dying of MODS were more likely to be younger and suffer from co-morbidities. As the majority of pts died from MODS were under inadequate treatment, and the results arrived quite late, we could speculate that timely processing and handling of blood culture results, and better antibiotic coverage could lead to better infection control and less progress to MODS and death.

**References**

1. Lee JK et al. Pediatrics 2010; 126:e859-864

2. Ten Berge J et al. BMC Pediatr 2006; 6-22

## P452 The potential impact of euthanasia on organ donation: analysis of data from belgium

### J. A. Bollen^1^, T. C. Van Smaalen^2^, W. C. De Jongh^2^, M. M. Ten Hoopen^1^, D. Ysebaert^3^, L. W. Van Heurn^4^, W. N. Van Mook^2^

#### ^1^Maastricht University, Maastricht, Netherlands; ^2^Maastricht University Medical Center, Maastricht, Netherlands; ^3^University Hospital Antwerp, Edegem, Belgium; ^4^Academic Medical Center, Amsterdam, Netherlands

**Introduction:** Organ donation after euthanasia has been performed at least 30 times in Belgium and the Netherlands, often at an ICU. Thousands undergo euthanasia each year, but it is unclear what the potential of this procedure can be for the donor pool.

**Methods:** It was not possible to obtain detailed euthanasia data from Dutch authorities. All Belgian euthanasia reports of 2013 were analyzed. The primary outcome was the number of patients which could donate at least 1 organ. Subgroup analysis was performed to evaluate the number of potential donors per organ.

Exclusion was based on the contraindications for donation within the Eurotransplant region. Cancer was an exclusion criterion as the majority of malignancies are a contra-indication for organ donation. Patients with “Multiple pathologies” were excluded, because they would be unlikely to be accepted as an organ donor. Patients with “Other pathologies” were excluded, because many mentioned contra-indications for donation or details about the pathology were lacking. For subgroup analysis, patients with renal disease were excluded as potential kidney donors and patients with lung disease as potential lung donors.

Within the Eurotransplant region, 75 years is the maximum age for donation of kidneys, lungs and pancreas islet isolation in Maastricht category 3 deceased donors. For potential liver donors, a maximum age of 60 years was used and for whole pancreas donors 50 years.

**Results:** Of 1811 obtained Belgian reports, a total of 186 potential donors with at least 1 suitable organ for donation remained. 6 patients with a history of kidney disease were excluded, leading to 360 potential kidney donations. For lung donation 31 patients were excluded leaving a potential of 155 lung donors. 113 patients between 60-79 years old were excluded, leaving 73 potential liver donors. For whole pancreas donation, the age group of 50-79 years was excluded, resulting in 30 potential pancreas donors.

**Conclusions:** In this first analysis of Belgian data on euthanasia in order to explore the potential of organ donation after euthanasia, 10,3% of all euthanasia cases are potentially suitable patients for donation. Whether they are willing to donate is unclear and should be studied. If only a small percentage of these patients would consider donation, this could mean an increase in donation in Belgium and the Netherlands.

## P453 Communication within an intensive care setting

### K. Sim, A. Fuller

#### St Helens and Knoowsley, Prescot, UK

**Introduction:** The ability to communicate well with patients and their relatives is a fundamental clinical skill in intensive care medicine and central to good medical practice. Communication at the bedside, even when the patient is unconscious or sedated, may often be recalled by critical care survivors and can impact upon long-term psychological outcomes[1].The Intensive Care Society released guidelines for the provision of intensive care services and one area which was highlighted was the patient and relative perspective, the importance of effective communication with relatives and patients and valuable time spent talking to the patient[2].The aim of this audit was to assess the communication skills on the daily ward rounds between the team leader, normally the on call Consultant, and the patient at the bedside.

**Methods:** An observational study comprising of 32 daily ward rounds on the 14 bedded Critical Care Unit (CCU) at Whiston hospital between October and November 2015. An average of 11 patients per ward round were recorded including non-ventilated and ventilated patients. A data collection tool was used which assessed whether the Consultant leading the ward round introduced themselves, explained what their role was and introduced the rest of the team.

**Results:** Out of the 348 patients who were on the CCU within this time period, the majority of these patients were not ventilated. Saying that it was found that only 53% of Consultants introduced themselves, 52% explained their role and 45% introduced the rest of the team. There seemed to be a significant difference in the communication between Consultants and ventilated patients, showing that only 16% introduced themselves, 14% explained their role and 11% introduced the rest of the team.

**Conclusions:** The communication between patients and doctors within the CCU calls for vast improvement. Given the evidence as mentioned, it is imperative that strategies are put in place to help improve the results which were found. This would include a simple adjunct next to the bedside to help remind the Consultant about the importance of effective communication. It is worthwhile re-auditing once this implementation has been put in place to see whether communication has improved.

**References**

1. Gauntlett R, Laws D. Continuing Education in Anaesthesia, Critical Care & Pain. Communication skills in critical care. Volume 8 Number 4 2008. Page 122.

2. Young K. Guidelines for the Provision of Intensive Care Services. The Patient and Relative Perspective. Section 3.1.5. Edition 1 2015. Page 63-6

## P454 Development and implementation of a longitudinal communication curriculum for critical care medicine fellows

### A. Roze des Ordons, P. Couillard, C. Doig

#### University of Calgary, Calgary, Canada

**Introduction:** Communication with patients and families in critical care medicine (CCM) can be complex and challenging. Physicians benefit from dedicated communication teaching, although the ideal model has not been established. Our purpose was to develop and assess the impact of a communication skills curriculum for CCM fellows.

**Methods:** Surveys including multiple choice and free-text questions were sent to all CCM fellows, staff physicians, nurses and social workers at our institution. The results informed the design of a longitudinal communication skills curriculum for CCM fellows. The effectiveness of the curriculum is being assessed through trends in clinician feedback.

**Results:** The survey response rate was 7/7 fellows, 15/29 staff physicians, 56/404 nurses, and 1/5 social workers. More than 50% of non-fellow respondents identified that fellows ranked below expectations in counseling about the emotional impact of emergency situations; fellows reported they had the least amount of training in this area and were least comfortable addressing patient and family emotional issues. Staff physicians were least comfortable teaching and assessing fellows’ capacity to address emotion, and had received the least amount of training in this area themselves. All non-trainee groups described fellows’ focus on giving information over building rapport; fellows indicated their challenges may be related to the absence of a prior relationship with patients and families, and their own discomfort in talking about death and dying. Despite challenges in the relational aspects of communication, topics of greatest interest to fellows included organ donation, adverse events, conflict, and family meetings. Preferred learning methods included simulation and feedback in clinical practice. This data guided the development of a communication skills curriculum involving 5 formal sessions over a year, and structured feedback during CCM rotations. Each formal session consisted of a didactic presentation and simulated practice. One formal session was dedicated to basic principles of communication, which were incorporated into each of the topic-based sessions. A form to guide multidisciplinary preceptors in providing feedback to fellows in clinical practice was also developed and implemented. Preliminary data indicate that fellows value the curriculum and feedback; more objective curricular evaluation is ongoing.

**Conclusions:** Kern’s model has been valuable in developing a blended communication skills curriculum tailored to CCM fellows’ needs. The curriculum has been received favorably.

## P455 Staff-family conflict in a multi-ethnic intensive care unit

### R. V. Van Keer^1^, R. D. Deschepper^1^, A. F. Francke^2^, L. H. Huyghens^3^, J. B. Bilsen^1^

#### ^1^Vrije Universiteit Brussel, Brussel, Belgium; ^2^EMGO+/VU University medical center, Utrecht, Netherlands; ^3^Universitair Ziekenhuis Brussel, Brussel, Belgium

**Introduction:** Conflicts between healthcare professionals and patients’ relatives are rather common in the intensive care unit (ICU). As a result of societies’ increased ethnic diversity these conflicts more often involve actors with a different ethno-cultural background. However few is known about the specific nature of staff-family conflicts in a multi-ethnic ICU environment. In this study, we give an overview of some characteristics of conflicts between family members from ethnic-minority groups and staff members and compare them to characteristics of staff-family conflict in general.

**Methods:** Ethnographic fieldwork was done in 1 ICU of a multi-ethnic urban hospital in Belgium. During 6 months, data were collected through negotiated interactive observation, in-depth-interviews with staff, from patients’ medical records, and by making notes in a logbook. Data were analyzed via grounded theory and compared to the literature on staff-family conflict in general.

**Results:** It is known that, in general, physicians identify a situation less often as conflict-loaded than relatives, suggesting the presence of hidden conflicts during critical care [1-2]. However, in our studied multi-ethnic ICU conflicts were found to be explicitly recognized by both relatives as well as healthcare professionals, and to be very visible and auditable on the ward. Moreover where in general relative-staff conflict tend to be centered around crisis moments (end-of-life decision making, patients’ death) [3], we found that in a multi-ethnic ICU conflicts tend to be present during various care phases (curative phase, end-of-life decision making, non-curative phase, patients’ death) and concern a broad spectrum of care aspects ( e.g. bedside care activities, seeking a second opinion), easily assaulting the core of actors’ identity. Consequently, end-of-life decisions, often a difficult assignment in ICUs, might be even more problematical in a multi-ethnic context than in general, as tensions in the pre-end of life decision making phase might worsen conflicts in the end-of-life decision making phase.

**Conclusions:** In ICUs, staff-family conflicts tend to be more severe and overtly present in a multi-ethnic context than in general. Therefore we urge for the development of specific and effective staff-family conflict prevention strategies in a multi-ethnic ICU.

**References**

1. Schuster RA et al. Crit Care Med 42:328-35, 2014.

2. Long AC et al. Crit Care Med 42:461-62, 2014.

3. Visser M et al. Crit Care doi: 10.1186/s13054-014-0604-z, 2014.

## P456 Does the source of admission to critical care affect family satisfaction?

### B. Nyamaizi^1^, C. Dalrymple^2^, A. Molokhia^1^, A. Dobru^1^

#### ^1^University Hospital Lewisham, London, UK; ^2^Queen Elizabeth Hospital, London, UK

**Introduction:** The management of critically ill patients is often challenging for clinicians with most being sedated or too unwell to be actively involved in aspects of their care.

The quality of care received by patients can also vary between hospital locations, which in turn influences family perceptions of care provision. Family members are surrogates for patients and monitoring their experiences positively influences patient outcomes [1].

The aim of this study was to identify whether the source of admission to critical care had any impact family satisfaction.

**Methods:** This prospective study was conducted over a period of eight weeks on our critical care unit. Up to four relatives per patient prior to discharge from the unit, or after initiation of end of life care, were requested to fill in a modified (Family satisfaction Survey (FSS)-ICU questionnaire. The qualitative responses obtained using a Likert scale were transformed into numerical data for analysis.

**Results:** 34 completed surveys were analysed with majority of the respondents being female (65%).

46% of the patients were admitted from the emergency resuscitation department and whilst admissions from the inpatient wards accounted for 34%.

Of the patients admitted from the emergency department the level of satisfaction was excellent (56%) or very good (54%) in all aspects of the patient care, as opposed to 36% of patients from the ward

**Conclusions:** The results show that relatives are more likely to be satisfied when admitted from the emergency department than the from an inpatient ward. There is a correlation between inclusion in the decision-making process and overall satisfaction. The timely manner in which patients are admitted from the emergency department (RESUS) as well as the levels of staffing on initial management may account for the results seen.

**Reference**

1. Wall et al. Refinement, scoring, and validation of the Family Satisfaction in the Intensive Care Unit (FS-ICU) survey. Crit Care Med 2007; 35:271-279

## P457 A simple alternative to the family satisfaction survey (fs-icu)

### E. Marrinan, A. Ankuli, A. Molokhia

#### Lewisham & Greenwich NHS Trust, London, UK

**Introduction:** We sought to evaluate whether the succinct Critical Care Family Needs Inventory (CCFNI) [1], a validated survey, may serve as a suitable alternative to the FS-ICU. Undertaking relative experience surveys annually was outlined as a quality indicator by the Scottish Intensive Care Society Quality Improvement Group in 2012. Over the past 5 years we have completed the FS-ICU annually. This is a lengthy survey covering aspects of care & decision making. The FSS has been previously validated in large US/Canadian studies [2]. More recently the Family Reported Experiences Evaluation (FREE) validated the FS-ICU in the UK, employing the survey across 20 different ICUs with approximately 7000 forms completed.

**Methods:** The audit was conducted across both hospitals in the trust. Over 4 weeks we distributed the CCFNI to relatives of patients on ITU/HDU once decision to step down to a ward was made. We included a section for open comments at the end of the survey.

**Results:** Over 4 weeks a total of 44 surveys were completed. Selected results are detailed below with comparison to results from similar questions in the FS-ICU survey.

Quality of care: CCFNI: 77% of relatives felt that the best possible care was being given to the patient almost all the time. FS-ICU: 72% felt the care received from doctors was excellent & 71% felt the care received from nurses was excellent.

Communication: CCFNI: 73% felt that explanations about the patient’s condition were given in terms that they could understand almost all of the time & the remaining 27% felt explanations were understandable most of the time. FS-ICU: 69% felt staff provided understandable explanations excellently, 31% described this information as very good.

Empathy: CCFNI: 84% stated that staff members showed an interest in how relatives were feeling almost all of the time and most of the time. FS-ICU: 72% stated that ICU staff were excellent in showing interest & consideration to relative needs, and 28% said staff were very good.

**Conclusions:** Results suggest that relatives are satisfied with the care that their relatives received at the trust. The results appear to correlate well with those from the FS-ICU in several domains such as care, communication and empathy. We therefore propose that the CCFNI can be used as an alternative annual survey to measure relatives experience on ICU.

**References**

1. Wall et al. Crit Care Med 35:271–279, 2007

2. Azoulay et al. Am J Respir Crit Care Med 163:135-9, 2001

## P458 A study to explore the experiences of patient and family volunteers in a critical care environment: a phenomenological analysis

### J. McPeake^1^, R. Struthers^2^, R. Crawford^2^, H. Devine^2^, P. Mactavish^2^, T. Quasim^1^

#### ^1^University of Glasgow, Glasgow, UK; ^2^NHS Greater Glasgow and Clyde, Glasgow, UK

**Introduction:** ICU survivors suffer persistent physical, psychological and social problems in the months and years after discharge from critical care (1). Caregivers of these patients also suffer similar problems (2). As a result, an innovative, peer supported rehabilitation programme- Intensive Care Syndrome: Promoting Independence and Return to Employment (InS:PIRE) was created in Glasgow Royal Infirmary. This 5 week multi disciplinary programme, which is co facilitated by patient and family volunteers further along the recovery trajectory, aims to empower patients and caregivers to take control of their health and wellbeing. The objective of this study was to explore the experiences of the volunteers who participated in InS:PIRE. It also sought to identify the support required by volunteers from healthcare professionals involved in the project.

**Methods:** Six in depth semi structured interviews were undertaken with volunteers (both patients and family members) involved in the InS:PIRE clinic by an assistant psychologist. A predetermined topic guide was utilised to guide interviews. Interviews were audio recorded and transcribed verbatim. Interpretative Phenomenological Analysis was used to analyse the transcripts (3). Peer Review was undertaken to ensure credibility of the findings.

**Results:** Findings: Six key themes were identified from these interviews: the social impact of volunteering, shared experiences; supporting others; personal boundaries; support needs and personal gain. The importance of peer support and having a shared understanding of participants needs were key themes for the volunteers. Volunteers described the need for further support in areas such as: confidentiality; listening skills and understanding boundaries.

**Conclusions:** The use of peer volunteers in this ICU rehabilitation service has been successful within this local context. Further, larger scale research studies, which explore further the impact of volunteering for ICU survivors are required.

**References**

1. Quasim, T et al (2015) Employment, social dependency and return to work after intensive care. Journal of the Intensive Care Society; 16(1):31-36.

2. Smith, JA. et al (2009) Interpretative phenomenological analysis. Sage Publications. London.

3. Haines, KJ. et al (2015) Psychosocial outcomes in informal caregivers of the critically ill: A systematic review. Critical Care Medicine; 43(5):1112-1120.

## P459 Prevalence and risk factors of anxiety and depression in relatives of burn patients.

### P. Morelli^1^, M. Degiovanangelo^1^, F. Lemos^1^, V. MArtinez^2^, F. Verga^1^, J. Cabrera^1^, G. Burghi^2^

#### ^1^National Burn Unit, Montevideo, Uruguay; ^2^Hospital Maciel, Montevideo, Uruguay

**Introduction:** Intensive care unit is one of the most stressful places in a Hospital. Relatives are exposed to many factors that may cause symptoms of anxiety and depression.

The aim of this study was to determine the prevalence of anxiety and depression symptoms 72 hour and 90 days after ICU adimssion, and identify factors associated with this symptoms.

**Methods:** Relatives of Patients admitted to the National Burn Center in Montevideo between february and october 2015 were invited to participate in this study.

72 hours and ninety days after ICU admission, family members completed a survey that included the Hospital Anxiety and Depression Scale.

**Results:** 95 and 45 relatives reponded the survey at 72 hours and 90 days after ICU admission repectively.

The prevalence of Anxiety symptoms were 60% and 28% 72 hours and 90 days after ICU admission respectively.

Symptoms of depression were present in 47% of relatives at the third day and 18% at 90 days after ICU admission.

72 hous after ICU admission, a longer time lived with the patient was associated with anxiety 18 (3-26) years vs 2 (0-23) years (p = 0,02), and with depression 18 (7-26) years vs 4(0-23) (p = 0,02). At this moment, a higher total burn area 27% (18-37) vs 19% (10-27) (p = 0,02) was associated with depression.

Factors associated with anxiety 90 days after ICU admission were: felt that treatment information was incomplete (80% vs 22%,p = 0,02), and not being able to return to work (80% vs 22%p = 0,02).

The only factor associated with depression symptoms 90 days afetr ICU admission was not being able to return to work (38% vs 11%, p = 0,04).

**Conclusions:** Prevalence of Anxiety and Depression among relatives was very important after 72 hours of ICU admission, and remained elevated 3 months later. This study shows factors associated with the apearence of anxiety and depression.

## P460 Guidance of visiting children at an adult intensive care unit (icu)

### A. Rutten, S. Van Ieperen, S. De Geer, M. Van Vugt, E. Der Kinderen

#### St Elisabeth Ziekenhuis, Tilburg, Netherlands

**Introduction:** St Elisabeth Hospital has a 30 bed ICU for critically ill adult patients. We facilitate family centred care, but in earlier years children were not allowed to visit family members in our ICU. The objective of our project was to facilitate paediatric visitation and to provide support related to the distress and needs of children with a relative in our ICU.

**Fig. 108 (Abstract P460).**
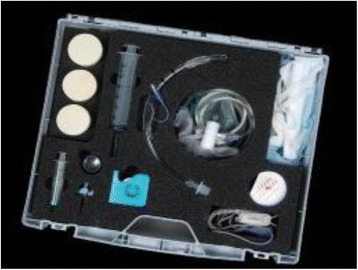
Our instruction box

**Fig. 109 (Abstract P460).**
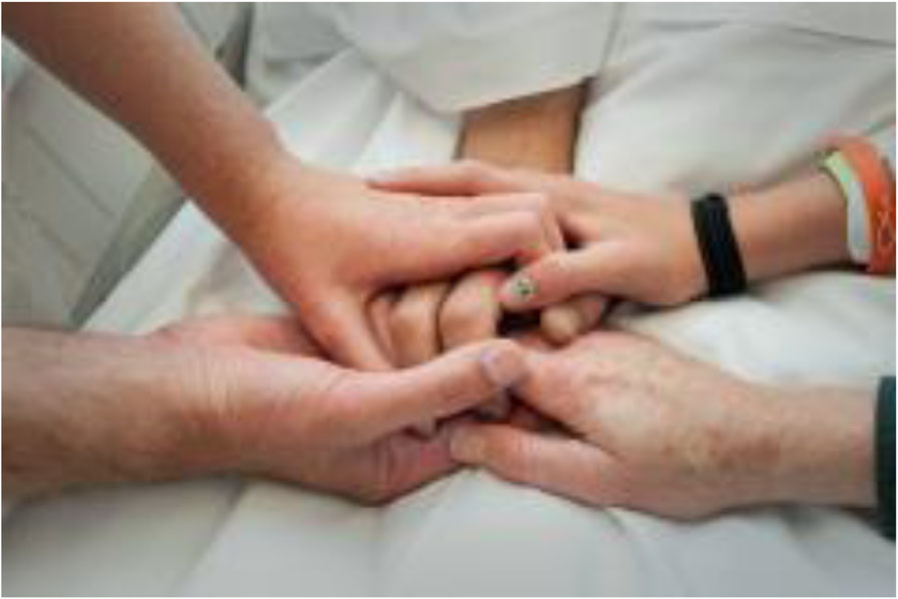
Last moments: hands of the family, including patient’s 14 and 16 year old siblings

**Methods:** Practical aids were developed for our caregivers. An information leaflet was made with practical information for parents. This was subdivided in developmental stages. Furthermore we created a book in which a child visits his father at our ICU. Pictures show what children can expect, which helps prepare the child for visiting. An instruction box is included with ICU materials (Fig. 108). These materials are used to give children a more tactile experience of the ICU. Guidance materials for when a patient may die were developed included. For example: there are little boxes that can be decorated by the children to put in a memory or a token of grief. Pedagogical staff are available to support parents, children and staff. If there are more profound problems there will be referral to our children’s psychologist. Our hospital photographers can be called upon to commemorate the last moments (Fig. 109). We instructed our nurses and doctors on how to use these materials and how to guide children. We held a survey among our staff to inquire if this met their needs.

**Results:** There’s an increasing awareness to the needs of children among our staff. Children and parents are welcomed and guided by our staff. Our survey amongst our staff showed a great appreciation for the materials we developed. When relatives of children die, children can be included in the farewell visit.

**Conclusions:** When a child is confronted with a sick or dying parent, loss and sorrow are inevitable. We, as care givers, can be more than bystanders and can help parents guide their children in difficult times.

## P461 Visiting policies in Italian pediatric ICUs: an update

### A. Giannini^1^, G Miccinesi^2^, T Marchesi^1^, E Prandi^1^

#### ^1^Fondazione IRCCS Ca’ Granda - Ospedale maggiore Policlinico, Milan, Italy; ^2^Istituto per lo Studio e la Prevenzione Oncologica, Florence, Italy

**Introduction:** In 2007 we found a clear tendency in Italian PICUs to apply restrictive visiting policies [1]. However, we also pointed out that a formal process of revision of the ward’s visiting policies was underway in roughly half of the units. Therefore, we carried out a new national survey to evaluate this issue in Italian PICUs.

**Methods:** An email questionnaire was sent to the heads of all 30 Italian PICUs asking about their visiting policies.

**Results:** The response rate was 100%. Median daily visiting time was 7 hours. Twenty per cent of surveyed PICUs (6/30) had unrestricted policies where one parent is allowed to be present both day and night, while 59% of units (17/30) do not allow constant presence of a parent even during the day.

Children were not permitted to visit in 64% (19/30) of PICUs. In the case of a dying patient, 53% of PICUs (16/30) extended visiting hours, 77% (23/30) increased the number of slots, and 77% (23/30) allowed more visitors.

A gowning procedure was compulsory for visitors in 70% (21/30) of PICUs. No waiting room for family members and visitors was provided by 13% of units (4/30). Thirteen PICUs out of thirty (43%) recently revised the ward’s visiting policies.

**Conclusions:** Although in Italian PICUs persists a tendency to apply restrictive visiting policies, these findings suggests that a revision of current policies is underway and that over the last eight years there has been perceptible change in favour of the liberalization of visiting in the Italian pediatric critical care setting. Despite the many objections considered valid in the past, there is conclusively no scientific basis for limiting family presence in ICU [2].

Our survey could contribute towards liberalizing visiting policies in Italian PICUs and promoting more attentive care for the patient and his/her family.

**Acknowledgments**

The study was supported by Associazione per il Bambino Nefropatico (Milan, Italy).

**References**

1. Giannini A et al. Pediatric Critical Care Med 2011;12:e46–e50

2. Giannini A et al. Intensive Care Med 2014;40:730–733

