# Erratum to: 36th International Symposium on Intensive Care and Emergency Medicine: Brussels, Belgium. 15-18 March 2016

**DOI:** 10.1186/s13054-016-1358-6

**Published:** 2016-10-24

**Authors:** Author et al, R. M. Bateman, M. D. Sharpe, J. E. Jagger, C. G. Ellis, J. Solé-Violán, M. López-Rodríguez, E. Herrera-Ramos, J. Ruíz-Hernández, L. Borderías, J. Horcajada, N. González-Quevedo, O. Rajas, M. Briones, F. Rodríguez de Castro, C. Rodríguez Gallego, F. Esen, G. Orhun, P. Ergin Ozcan, E. Senturk, C. Ugur Yilmaz, N. Orhan, N. Arican, M. Kaya, M. Kucukerden, M. Giris, U. Akcan, S. Bilgic Gazioglu, E. Tuzun, R. Riff, O. Naamani, A. Douvdevani, R. Takegawa, H. Yoshida, T. Hirose, N. Yamamoto, H. Hagiya, M. Ojima, Y. Akeda, O. Tasaki, K. Tomono, T. Shimazu, S. Ono, T. Kubo, S. Suda, T. Ueno, T. Ikeda, T. Hirose, H. Ogura, H. Takahashi, M. Ojima, J. Kang, Y. Nakamura, T. Kojima, T. Shimazu, T. Ikeda, S. Suda, Y. Izutani, T. Ueno, S. Ono, T. Taniguchi, M. O, C. Dinter, J. Lotz, B. Eilers, C. Wissmann, R. Lott, M. M. Meili, P. S. Schuetz, H. Hawa, M. Sharshir, M. Aburageila, N. Salahuddin, V. Chantziara, S. Georgiou, A. Tsimogianni, P. Alexandropoulos, A. Vassi, F. Lagiou, M. Valta, G. Micha, E. Chinou, G. Michaloudis, A. Kodaira, T. Ikeda, S. Ono, T. Ueno, S. Suda, Y. Izutani, H. Imaizumi, M. V. De la Torre-Prados, A. Garcia-De la Torre, A. Enguix-Armada, A. Puerto-Morlan, V. Perez-Valero, A. Garcia-Alcantara, N. Bolton, J. Dudziak, S. Bonney, A. Tridente, P. Nee, G. Nicolaes, M. Wiewel, M. Schultz, K. Wildhagen, J. Horn, R. Schrijver, T. Van der Poll, C. Reutelingsperger, S. Pillai, G. Davies, G. Mills, R. Aubrey, K. Morris, P. Williams, P. Evans, E. G. Gayat, J. Struck, A. Cariou, N. Deye, B. Guidet, S. Jabert, J. Launay, M. Legrand, M. Léone, M. Resche-Rigon, E. Vicaut, A. Vieillard-Baron, A. Mebazaa, R. Arnold, M. Capan, A. Linder, P. Akesson, M. Popescu, D. Tomescu, C. L. Sprung, R. Calderon Morales, G. Munteanu, E. Orenbuch-Harroch, P. Levin, H. Kasdan, A. Reiter, T. Volker, Y. Himmel, Y. Cohen, J. Meissonnier, L. Girard, F. Rebeaud, I. Herrmann, B. Delwarde, E. Peronnet, E. Cerrato, F. Venet, A. Lepape, T. Rimmelé, G. Monneret, J. Textoris, N. Beloborodova, V. Moroz, A. Osipov, A. Bedova, Y. Sarshor, A. Pautova, A. Sergeev, E. Chernevskaya, J. Odermatt, R. Bolliger, L. Hersberger, M. Ottiger, M. Christ-Crain, B. Mueller, P. Schuetz, N. K. Sharma, A. K. Tashima, M. K. Brunialti, F. R. Machado, M. Assuncao, O. Rigato, R. Salomao, S. C. Cajander, G. Rasmussen, E. Tina, B. Söderquist, J. Källman, K. Strålin, A. L. Lange, J. S. Sundén-Cullberg, A. M. Magnuson, O. H. Hultgren, G. Davies, S. Pillai, G. Mills, R. Aubrey, K. Morris, P. Williams, P. Evans, S. Pillai, G. Davies, G. Mills, R. Aubrey, K. Morris, P. Williams, P. Evans, S. Pillai, G. Davies, G. Mills, R. Aubrey, K. Morris, P. Williams, P. Evans, P. Van der Geest, M. Mohseni, J. Linssen, R. De Jonge, S. Duran, J. Groeneveld, R. Miller, B. K. Lopansri, L. C. McHugh, A. Seldon, J. P. Burke, J. Johnston, R. Reece-Anthony, A. Bond, A. Molokhia, C. Mcgrath, E. Nsutebu, P. Bank Pedersen, D. Pilsgaard Henriksen, S. Mikkelsen, A. Touborg Lassen, R. Tincu, C. Cobilinschi, D. Tomescu, Z. Ghiorghiu, R. Macovei, M. A. Wiewel, M. B. Harmon, L. A. Van Vught, B. P. Scicluna, A. J. Hoogendijk, J. Horn, A. H. Zwinderman, O. L. Cremer, M. J. Bonten, M. J. Schultz, T. Van der Poll, N. P. Juffermans, W. J. Wiersinga, G. Eren, Y. Tekdos, M. Dogan, O. Acicbe, E. Kaya, O. Hergunsel, S. Alsolamy, G. Ghamdi, L. Alswaidan, S. Alharbi, F. Alenezi, Y. Arabi, J. Heaton, A. Boyce, L. Nolan, J. Johnston, A. Dukoff-Gordon, A. Dean, A. Molokhia, T. Mann Ben Yehudah, C. Fleischmann, D. Thomas-Rueddel, C. Haas, U. Dennler, K. Reinhart, O. Suntornlohanakul, B. Khwannimit, F. Breckenridge, A. Puxty, P. Szturz, P. Folwarzcny, J. Svancara, R. Kula, P. Sevcik, L. Caneva, A. Casazza, E. Bellazzi, S. Marra, L. Pagani, M. Vetere, R. Vanzino, D. Ciprandi, R. Preda, R. Boschi, L. Carnevale, V. Lopez, M. Aguilar Arzapalo, L. Barradas, A. Escalante, J. Gongora, M. Cetina, B Adamik, D Jakubczyk, A Kübler, A. Radford, T. Lee, J. Singer, J. Boyd, D. Fineberg, M. Williams, J. Russell, E. Scarlatescu, D. Tomescu, G. Droc, S. Arama, M. Müller, M. Straat, S. S. Zeerleder, N. P. Juffermans, C. F. Fuchs, C. S. Scheer, S. W. Wauschkuhn, M. V. Vollmer, K. M. Meissner, S. K. Kuhn, K. H. Hahnenkamp, S. R. Rehberg, M. G. Gründling, N. Yamamoto, M. Ojima, S. Hamaguchi, T. Hirose, Y. Akeda, R. Takegawa, O. Tasaki, T. Shimazu, K. Tomono, E. Gómez-Sánchez, M. Heredia-Rodríguez, E. Álvarez-Fuente, M. Lorenzo-López, E. Gómez-Pesquera, M. Aragón-Camino, P. Liu-Zhu, A. Sánchez-López, A. Hernández-Lozano, M. T. Peláez-Jareño, E. Tamayo, D. O. Thomas-Rüddel, C. Fleischmann, C. Haas, U. Dennler, K. Reinhart, V. Adora, A. Kar, A. Chakraborty, S. Roy, A. Bandyopadhyay, M. Das, T. Mann Ben Yehudah, G. BenYehudah, M. Salim, N. Kumar, L. Arabi, T. Burger, P. Lephart, E. Toth-martin, C. Valencia, N. Hammami, S. Blot, J. L. Vincent, M. L. Lambert, J. Brunke, T. Riemann, I. Roschke, R. Tincu, C. Cobilinschi, D. Tomescu, Z. Ghiorghiu, R. Macovei, S. Nimitvilai, K. Jintanapramote, S. Jarupongprapa, D. Adukauskiene, D. Valanciene, G. Bose, V. Lostarakos, B. Carr, S. Khedher, A. Maaoui, A. Ezzamouri, M. Salem, J. Chen, D. R. Cranendonk, L. A. Van Vught, M. A. Wiewel, O. L. Cremer, J. Horn, M. J. Bonten, M. J. Schultz, T. Van der Poll, W. J. Wiersinga, M. Day, G. Penrice, K. Roy, P. Robertson, G. Godbole, B. Jones, M. Booth, L. Donaldson, Y. Kawano, H. Ishikura, H. Al-Dorzi, M. Almutairi, B. Alhamadi, A. Crizaldo Toledo, R. Khan, B. Al Raiy, Y. Arabi, H. Talaie, J. A. Van Oers, A. Harts, E. Nieuwkoop, P. Vos, Y. Boussarsar, F. Boutouta, S. Kamoun, I. Mezghani, S. Koubaji, A. Ben Souissi, A. Riahi, M. S. Mebazaa, E. Giamarellos-Bourboulis, N. Tziolos, C. Routsi, C. Katsenos, I. Tsangaris, I. Pneumatikos, G. Vlachogiannis, V. Theodorou, A. Prekates, E. Antypa, V. Koulouras, N. Kapravelos, C. Gogos, E. Antoniadou, K. Mandragos, A. Armaganidis, A. R. Robles Caballero, B. Civantos, J. C. Figueira, J. López, A. Silva-Pinto, F. Ceia, A. Sarmento, L. Santos, G. Almekhlafi, Y. Sakr, H. Al-Dorzi, R. Khan, S. Baharoon, A. Aldawood, A. Matroud, J. Alchin, S. Al Johani, H. Balkhy, Y. Arabi, S. Alsolamy, S. Y. Yousif, B. O. Alotabi, A. S. Alsaawi, J. Ang, M. D. Curran, D. Enoch, V. Navapurkar, A. Morris, R. Sharvill, J. Astin, M. Heredia-Rodríguez, E. Gómez-Sánchez, M. T. Peláez-Jareño, E. Gómez-Pesquera, M. Lorenzo-López, P. Liu-Zhu, M. Aragón-Camino, A. Hernández-Lozano, A. Sánchez-López, E. Álvarez-Fuente, E. Tamayo, J. Patel, C. Kruger, J. O’Neal, H. Rhodes, J. Jancik, B. François, P. F. Laterre, P. Eggimann, A. Torres, M. Sánchez, P. F. Dequin, G. L. Bassi, J. Chastre, H. S. Jafri, M. Ben Romdhane, Z. Douira, S. Kamoun, M. Bousselmi, A. Ben Souissi, Y. Boussarsar, A. Riahi, M. S. Mebazaa, A. Vakalos, V. Avramidis, T. H. Craven, G. Wojcik, K. Kefala, J. McCoubrey, J. Reilly, R. Paterson, D. Inverarity, I. Laurenson, T. S. Walsh, S. Mongodi, B. Bouhemad, A. Orlando, A. Stella, G. Via, G. Iotti, A. Braschi, F. Mojoli, M. Haliloglu, B. Bilgili, U. Kasapoglu, I. Sayan, M. Süzer Aslan, A. Yalcin, I. Cinel, A. Vakalos, V. Avramidis, H. E. Ellis, K. Bauchmuller, D. Miller, A. Temple, J. Chastre, B. François, A. Torres, C. E. Luyt, M. Sánchez, M. Singer, H. S. Jafri, Y. Nassar, M. S. Ayad, A. Trifi, S. Abdellatif, F. Daly, R. Nasri, S. Ben Lakhal, B. Bilgili, M. Haliloglu, F. Gul, I. Cinel, A. Kuzovlev, A. Shabanov, S. Polovnikov, V. Moroz, N. Kadrichu, T. Dang, K. Corkery, P. Challoner, G. Li Bassi, E. Aguilera, C. Chiurazzi, C. Travierso, A. Motos, L. Fernandez, R. Amaro, T. Senussi, F. Idone, J. Bobi, M. Rigol, A. Torres, C. J. Hodiamont, N. P. Juffermans, J. M. Janssen, C. S. Bouman, R. A. Mathôt, M. D. De Jong, R. M. Van Hest, L. Payne, G. L. Fraser, B. Tudor, M. Lahner, G. Roth, C. Krenn, H. Talaie, P. Jault, J. Gabard, T. Leclerc, S. Jennes, Y. Que, A. Rousseau, F. Ravat, H. Al-Dorzi, A. Eissa, S. Al-Harbi, T. Aldabbagh, R. Khan, Y. Arabi, A. Trifi, S. Abdellatif., F. Daly, R. Nasri, S. Ben Lakhal, F. Paramba, N. Purayil, V. Naushad, O. Mohammad, V. Negi, P. Chandra, A. Kleinsasser, M. R. Witrz, J. F. Buchner-Doeven, A. M. Tuip-de Boer, J. C. Goslings, N. P. Juffermans, M. Van Hezel, M. Straat, A Boing, R Van Bruggen, N Juffermans, D. Markopoulou, K. Venetsanou, V. Kaldis, D. Koutete, D. Chroni, I. Alamanos, L. Koch, J. Jancik, H. Rhodes, E. Walter, K. Maekawa, M. Hayakawa, S. Kushimoto, A. Shiraishi, H. Kato, J. Sasaki, H. Ogura, T. Matauoka, T. Uejima, N. Morimura, H. Ishikura, A. Hagiwara, M. Takeda, O. Tarabrin, S. Shcherbakow, D. Gavrychenko, G. Mazurenko, V. Ivanova, O. Chystikov, C. Plourde, J. Lessard, J. Chauny, R. Daoust, S. Shcherbakow, O. Tarabrin, D. Gavrychenko, G. Mazurenko, O. Chystikov, A. Vakalos, V. Avramidis, L. Kropman, L. In het Panhuis, J. Konings, D. Huskens, E. Schurgers, M. Roest, B. De Laat, M. Lance, M. Durila, P. Lukas, M. Astraverkhava, J. Jonas, I. Budnik, B. Shenkman, H. Hayami, Y. Koide, T. Goto, R. Iqbal, Y. Alhamdi, N. Venugopal, S. Abrams, C. Downey, C. H. Toh, I. D. Welters, V. B. Bombay, J. M. Chauny, R. D. Daoust, J. L. Lessard, M. M. Marquis, J. P. Paquet, K. Siemens, D. Sangaran, B. J. Hunt, A. Durward, A. Nyman, I. A. Murdoch, S. M. Tibby, F. Ampatzidou, D. Moisidou, E. Dalampini, M. Nastou, E. Vasilarou, V. Kalaizi, H. Chatzikostenoglou, G. Drossos, S. Spadaro, A. Fogagnolo, T. Fiore, A. Schiavi, V. Fontana, F. Taccone, C. Volta, E. Chochliourou, E. Volakli, A. Violaki, E. Samkinidou, G. Evlavis, V. Panagiotidou, M. Sdougka, R. Mothukuri, C. Battle, K. Guy, G. Mills, P. Evans, J. Wijesuriya, S. Keogh, A. Docherty, R. O’Donnell, S. Brunskill, M. Trivella, C. Doree, L. Holst, M. Parker, M. Gregersen, J. Almeida, T. Walsh, S. Stanworth, S. Moravcova, J. Mansell, A. Rogers, R. A. Smith, C. Hamilton-Davies, A. Omar, M. Allam, O. Bilala, A. Kindawi, H. Ewila, F. Ampatzidou, D. Moisidou, M. Nastou, E. Dalampini, A. Malamas, E. Vasilarou, G. Drossos, G. Ferreira, J. Caldas, J. Fukushima, E. A. Osawa, E. Arita, L. Camara, S. Zeferino, J. Jardim, F. Gaioto, L. Dallan, F. B. Jatene, R. Kalil Filho, F. Galas, L. A. Hajjar, C. Mitaka, T. Ohnuma, T. Murayama, F. Kunimoto, M. Nagashima, T. Takei, M. Tomita, A. Omar, K. Mahmoud, S. Hanoura, S. Sudarsanan, P. Sivadasan, H. Othamn, Y. Shouman, R. Singh, A. Al Khulaifi, I. Mandel, S. Mikheev, I. Suhodolo, V. Kiselev, Y. Svirko, Y. Podoksenov, S. A. Jenkins, R. Griffin, M. S. Tovar Doncel, A. Lima, C. Aldecoa, C. Ince, A. Taha, A. Shafie, M. Mostafa, N. Syed, H. Hon, F. Righetti, E. Colombaroli, G. Castellano, F. Righetti, E. Colombaroli, M. Hravnak, L. C. Chen, A. D. Dubrawski, G. C. Clermont, M. R. Pinsky, S. Gonzalez, D. Macias, J. Acosta, P. Jimenez, A. Loza, A. Lesmes, F. Lucena, C. Leon, M. S. Tovar Doncel, C. Ince, C. Aldecoa, A. Lima, M. Bastide, J. Richecoeur, E. Frenoy, C. Lemaire, B. Sauneuf, F. Tamion, S. Nseir, D. Du Cheyron, H. Dupont, J. Maizel, M. Shaban, R. Kolko, N. Salahuddin, M. Sharshir, M. AbuRageila, A. AlHussain, P. Mercado, J. Maizel, L. Kontar, D. Titeca, F. Brazier, A. Riviere, M. Joris, T. Soupison, B. De Cagny, M. Slama, J. Wagner, A. Körner, M. Kubik, S. Kluge, D. Reuter, B. Saugel, E. Colombaroli, F. Righetti, G. Castellano, T. Tran, D. De Bels, A. Cudia, M. Strachinaru, P. Ghottignies, J. Devriendt, C. Pierrakos, Ó. Martínez González, R. Blancas, J. Luján, D. Ballesteros, C. Martínez Díaz, A. Núñez, C. Martín Parra, B. López Matamala, M. Alonso Fernández, M. Chana, W. Huber, M. Eckmann, F. Elkmann, A. Gruber, I. Klein, R. M. Schmid, T. Lahmer, P. W. Moller, S. Sondergaard, S. M. Jakob, J. Takala, D. Berger, D. Bastoni, H. Aya, L. Toscani, L. Pigozzi, A. Rhodes, M. Cecconi, C. Ostrowska, H. Aya, A. Abbas, J. Mellinghoff, C. Ryan, D. Dawson, A. Rhodes, M. Cecconi, M. Cronhjort, O. Wall, E. Nyberg, R. Zeng, C. Svensen, J. Mårtensson, E. Joelsson-Alm, M. Aguilar Arzapalo, L. Barradas, V. Lopez, M. Cetina, N. Parenti, C. Palazzi, L. A. Amidei, F. B. Borrelli, S. C. Campanale, F. T. Tagliazucchi, G. S. Sedoni, D. L. Lucchesi, E. C. Carella, A. L Luciani, M. Mackovic, N. Maric, M. Bakula, H. Aya, A. Rhodes, R. M. Grounds, N. Fletcher, M. Cecconi, B. Avard, P. Zhang, M. Mezidi, J. Charbit, M. Ould-Chikh, P. Deras, C. Maury, O. Martinez, X. Capdevila, P. Hou, W. Z. Linde-Zwirble, I. D. Douglas, N. S. Shapiro, A. Ben Souissi, I. Mezghani, Y. Ben Aicha, S. Kamoun, B. Laribi, B. Jeribi, A. Riahi, M. S. Mebazaa, C. Pereira, R. Marinho, R. Antunes, A. Marinho, M. Crivits, M. Raes, J. Decruyenaere, E. Hoste, V. Bagin, V. Rudnov, A. Savitsky, M. Astafyeva, I. Korobko, V. Vein, T. Kampmeier, P. Arnemann, M. Hessler, A. Wald, K. Bockbreder, A. Morelli, H. Van Aken, S. Rehberg, C. Ertmer, P. Arnemann, M. Hessler, T. Kampmeier, S. Rehberg, H. Van Aken, C. Ince, C. Ertmer, S. Reddy, M. Bailey, R. Beasley, R. Bellomo, D. Mackle, A. Psirides, P. Young, S. Reddy, M. Bailey, R. Beasley, R. Bellomo, D. Mackle, P. Young, H. Venkatesh, S. Ramachandran, A. Basu, H. Nair, S. Egan, J. Bates, S. Oliveira, N. R. Rangel Neto, F. Q. Reis, C. P. Lee, X. L. Lin, C. Choong, K. M. Eu, W. Y. Sim, K. S. Tee, J. Pau, J. Abisheganaden, K. Maas, H. De Geus, E. Lafuente, R. Marinho, J. Moura, R. Antunes, A. Marinho, T. E. Doris, D. Monkhouse, T. Shipley, S. Kardasz, I Gonzalez, S. Stads, A. J. Groeneveld, I. Elsayed, N. Ward, A. Tridente, A. Raithatha, A. Steuber, C. Pelletier, S. Schroeder, E. Michael, T. Slowinski, D. Kindgen-Milles, S. Ghabina, F. Turani, A. Belli, S. Busatti, G. Barettin, F. Candidi, F. Gargano, R. Barchetta, M. Falco, O. Demirkiran, M. Kosuk, S. Bozbay, V. Weber, J. Hartmann, S. Harm, I. Linsberger, T. Eichhorn, G. Valicek, G. Miestinger, C. Hoermann, S. Faenza, D. Ricci, E. Mancini, C. Gemelli, A. Cuoghi, S. Magnani, M. Atti, T. Laddomada, A. Doronzio, B. Balicco, M. C. Gruda, P. O’Sullivan, V. P. Dan, T. Guliashvili, A. Scheirer, T. D. Golobish, V. J. Capponi, P. P. Chan, K. Kogelmann, M. Drüner, D. Jarczak, F. Turani, A. B. Belli, S. M. Martni, V. C. Cotticelli, F. Mounajergi, R. Barchetta, S. Morimoto, H. Ishikura, I. Hussain, N. Salahuddin, A. Nadeem, K. Ghorab, K. Maghrabi, S. K. Kloesel, C. Goldfuss, A. Stieglitz, A. S. Stieglitz, L. Krstevska, G. Albuszies, M. Aguilar Arzapalo, L. Barradas, V. Lopez, A. Escalante, G. Jimmy, M. Cetina, J. Izawa, T. Iwami, S. Uchino, M. Takinami, T. Kitamura, T. Kawamura, J. G. Powell-Tuck, S. Crichton, M. Raimundo, L. Camporota, D. Wyncoll, M. Ostermann, A. Hana, H. R. De Geus, H. R. De Geus, A. Hana, M. Aydogdu, N. Boyaci, S. Yuksel, G. Gursel, A. B. Cayci Sivri, J. Meza-Márquez, J. Nava-López, R. Carrillo-Esper, A. Dardashti, A. Grubb, J. Maizel, M. Wetzstein, D. Titeca, L. Kontar, F. Brazier, B. De Cagny, A. Riviere, T. Soupison, M. Joris, M. Slama, E. Peters, H. Njimi, P. Pickkers, J. L. Vincent, M. Waraich, J. Doyle, T. Samuels, L. Forni, N. Desai, R. Baumber, P. Gunning, A. Sell, S. Lin, H. Torrence, M. O’Dwyer, C. Kirwan, J. Prowle, T. Kim, M. E. O’Connor, R. W. Hewson, C. J. Kirwan, R. M. Pearse, J. Prowle, S. Hanoura, A. Omar, H. Othamn, S. Sudarsanan, M. Allam, M. Maksoud, R. Singh, A. Al Khulaifi, M. E. O’Connor, R. W. Hewson, C. J. Kirwan, R. M. Pearse, J. Prowle, O. Uzundere, D. Memis, M. Ýnal, A. Gultekin, N. Turan, M. A. Aydin, H. Basar, I. Sencan, A. Kapuagasi, M. Ozturk, Z. Uzundurukan, D. Gokmen, A. Ozcan, C. Kaymak, V. A. Artemenko, A. Budnyuk, R. Pugh, S. Bhandari, T. Mauri, C. Turrini, T. Langer, P. Taccone, C. A. Volta, C. Marenghi, L. Gattinoni, A. Pesenti, L. Sweeney, A. O’Sullivan, P. Kelly, E. Mukeria, R. MacLoughlin, M. Pfeffer, J. T. Thomas, G. B. Bregman, G. K. Karp, E. K. Kishinevsky, D. S. Stavi, N. A. Adi, T. Poropat, R. Knafelj, E. Llopart, M. Batlle, C. De Haro, J. Mesquida, A. Artigas, D. Pavlovic, L. Lewerentz, A. Spassov, R. Schneider, S. De Smet, S. De Raedt, E. Derom, P Depuydt, S. Oeyen, D. Benoit, J. Decruyenaere, A. Gobatto, B. Besen, P. Tierno, L. Melro, P. Mendes, F. Cadamuro, M. Park, L. M. Malbouisson, B. C. Civantos, J. L. Lopez, A. Robles, J. Figueira, S. Yus, A. Garcia, A. Oglinda, G. Ciobanu, C. Oglinda, L. Schirca, T. Sertinean, V. Lupu, P. Kelly, A. O’Sullivan, L. Sweeney, R. MacLoughlin, A. O’Sullivan, P. Kelly, L. Sweeney, E. Mukeria, M. Wolny, R. MacLoughlin, A. Pagano, F. Numis, G. Visone, L. Saldamarco, T. Russo, G. Porta, F. Paladino, C. Bell, J. Liu, J. Debacker, C. Lee, E. Tamberg, V. Campbell, S. Mehta, A. Silva-Pinto, A. Sarmento, L. Santos, Ý. Kara, F. Yýldýrým, A. Zerman, Z. Güllü, N. Boyacý, B. Basarýk Aydogan, Ü. Gaygýsýz, K. Gönderen, G. Arýk, M. Turkoglu, M. Aydogdu, G. Aygencel, Z. Ülger, G. Gursel, N. Boyacý, Z. Isýkdogan, Ö. Özdedeoglu, Z. Güllü, M. Badoglu, U. Gaygýsýz, M. Aydogdu, G. Gursel, N. Kongpolprom, C. Sittipunt, A. Eden, Y. Kokhanovsky, S. Bursztein – De Myttenaere, R. Pizov, L. Neilans, N. MacIntyre, M. Radosevich, B. Wanta, V. Weber, T. Meyer, N. Smischney, D. Brown, D. Diedrich, A. Fuller, P. McLindon, K. Sim, M. Shoaeir, K. Noeam, A. Mahrous, R. Matsa, A. Ali, C. Dridi, S. Koubaji, S. Kamoun, F. Haddad, A. Ben Souissi, B. Laribi, A. Riahi, M. S. Mebazaa, A. Pérez-Calatayud, R. Carrillo-Esper, A. Zepeda-Mendoza, M. Diaz-Carrillo, E. Arch-Tirado, S. Carbognin, L. Pelacani, F. Zannoni, A. Agnoli, G. Gagliardi, R. Cho, A. Adams, S. Lunos, S. Ambur, R. Shapiro, M. Prekker, M. Thijssen, L. Janssen, N. Foudraine, C. J. Voscopoulos, J. Freeman, C. J. Voscopoulos, J. Freeman, E. George, C. J. Voscopoulos, D. Eversole, J. Freeman, E. George, S. Muttini, R. Bigi, G. Villani, N. Patroniti, G. Williams, C. J. Voscopoulos, J. Freeman, E George, A. Waldmann, S. Böhm, W. Windisch, S. Strassmann, C. Karagiannidis, A. Waldmann, S. Böhm, W. Windisch, S. Strassmann, C. Karagiannidis, C. K. Karagiannidis, A. W. Waldmann, S. B. Böhm, S. Strassmann, W. W. Windisch, P. Persson, S. Lundin, O. Stenqvist, G. Porta, F. Numis, C. S. Serra, A. P. Pagano, M. M. Masarone, L. R. Rinaldi, A. A. Amelia, M. F. Fascione, L. A. Adinolfi, E. R. Ruggiero, F. Asota, K. O’Rourke, S. Ranjan, P. Morgan, J. W. DeBacker, E. Tamberg, L. O’Neill, L. Munshi, L. Burry, E. Fan, S. Mehta, S. Poo, K. Mahendran, J. Fowles, C. Gerrard, A. Vuylsteke, R. Loveridge, C. Chaddock, S. Patel, V. Kakar, C. Willars, T. Hurst, C. Park, T. Best, A. Vercueil, G. Auzinger, A. Borgman, A. G. Proudfoot, E. Grins, K. E. Emiley, J. Schuitema, S. J. Fitch, G. Marco, J. Sturgill, M. G. Dickinson, M. Strueber, A. Khaghani, P. Wilton, S. M. Jovinge, C. Sampson, S. Harris-Fox, M. E. Cove, L. H. Vu, A. Sen, W. J. Federspiel, J. A. Kellum, C. Mazo Torre, J. Riera, S. Ramirez, B. Borgatta, L. Lagunes, J. Rello, A. K. Kuzovlev, V. Moroz, A. Goloubev, S. Polovnikov, S. Nenchuk, V. Karavana, C. Glynos, A. Asimakos, K. Pappas, C. Vrettou, M. Magkou, E. Ischaki, G. Stathopoulos, S. Zakynthinos, S. Spadaro, I. Kozhevnikova, F. Dalla Corte, S. Grasso, P. Casolari, G. Caramori, C. Volta, T. Andrianjafiarinoa, T. Randriamandrato, T. Rajaonera, S. El-Dash, E. L. V. Costa, M. R. Tucci, F Leleu, L Kontar, B. De Cagny, F. Brazier, D. Titeca, G. Bacari-Risal, J. Maizel, M. Amato, M. Slama, P. Mercado, J. Maizel, L. Kontar, D. Titeca, F. Brazier, A. Riviere, M. Joris, T. Soupison, B. De Cagny, S. El Dash, M. Slama, Remmington, A. Fischer, S. Squire, M. Boichat, H. Honzawa, H. Yasuda, T. Adati, S. Suzaki, M. Horibe, M. Sasaki, M. Sanui, R. Marinho, J. Daniel, H. Miranda, A. Marinho, K. Milinis, M. Cooper, G. R. Williams, E. McCarron, S. Simants, I. Patanwala, I. Welters, Y. Su, J. Fernández Villanueva, R. Fernández Garda, A. López Lago, E. Rodríguez Ruíz, R. Hernández Vaquero, S. Tomé Martínez de Rituerto, E. Varo Pérez, N. Lefel, F. Schaap, D. Bergmans, S. Olde Damink, M. Van de Poll, K. Tizard, C. Lister, L. Poole, D. Ringaitiene, D. Gineityte, V. Vicka, I. Norkiene, J. Sipylaite, A. O’Loughlin, V. Maraj, J. Dowling, M. B. Velasco, D. M. Dalcomune, E. B. Dias, S. L. Fernandes, T. Oshima, S. Graf, C. Heidegger, L. Genton, V. Karsegard, Y. Dupertuis, C. Pichard, N. Friedli, Z. Stanga, B. Mueller, P. Schuetz, L. Vandersteen, B. Stessel, S. Evers, A. Van Assche, L. Jamaer, J. Dubois, R. Marinho, H. Castro, J. Moura, J. Valente, P. Martins, P. Casteloes, C. Magalhaes, S. Cabral, M. Santos, B. Oliveira, A. Salgueiro, A. Marinho, R. Marinho, M. Santos, E. Lafuente, H. Castro, S. Cabral, J. Moura, P. Martins, B. Oliveira, A. Salgueiro, S. Duarte, S. Castro, M. Melo, P. Casteloes, A. Marinho, S. Gray, K. Maipang, R. Bhurayanontachai, L. G. Grädel, P. Schütz, P. Langlois, W. Manzanares, R. Tincu, C. Cobilinschi, D. Tomescu, Z. Ghiorghiu, R. Macovei, W. Manzanares, P. Langlois, M. Lemieux, G. Elke, F. Bloos, K. Reinhart, D. Heyland, P. Langlois, M. Lemieux, I. Aramendi, D. Heyland, W. Manzanares, Y. Su, R. Marinho, N. Babo, A. Marinho, M. Hoshino, Y. Haraguchi, S. Kajiwara, T. Mitsuhashi, T. Tsubata, M. Aida, T. Rattanapraphat, R. Bhurayanontachai, C. Kongkamol, B. Khwannimit, R. Marinho, M. Santos, H. Castro, E. Lafuente, A. Salgueiro, S. Cabral, P. Martins, J. Moura, B. Oliveira, M. Melo, B. Xavier, J. Valente, C. Magalhaes, P. Casteloes, A. Marinho, D. Moisidou, F. Ampatzidou, C. Koutsogiannidis, M. Moschopoulou, G. Drossos, G. Taskin, M. Çakir, AK Güler, A. Taskin, N. Öcal, S. Özer, L. Yamanel, J. M. Wong, C. Fitton, S. Anwar, S. Stacey, M. Aggou, B. Fyntanidou, S. Patsatzakis, E. Oloktsidou, K. Lolakos, E. Papapostolou, V. Grosomanidis, S. Suda, T. Ikeda, S. Ono, T. Ueno, Y. Izutani, S. Gaudry, V. Desailly, P. Pasquier, PB Brun, AT Tesnieres, JD Ricard, D. Dreyfuss, A. Mignon, J. C White, A. Molokhia, A. Dean, A. Stilwell, G. Friedlaender, M. Peters, S. Stipulante, A. Delfosse, AF Donneau, A. Ghuysen, C. Feldmann, D. Freitag, W. Dersch, M. Irqsusi, D. Eschbach, T. Steinfeldt, H. Wulf, T. Wiesmann, N. Kongpolprom, J. Cholkraisuwat, S. Beitland, E. Nakstad, H. Stær-Jensen, T. Drægni, G. Andersen, D. Jacobsen, C. Brunborg, B. Waldum-Grevbo, K. Sunde, K. Hoyland, D. Pandit, K. Hayakawa, E. Oloktsidou, K. Kotzampassi, B. Fyntanidou, S. Patsatzakis, L. Loukipoudi, E. Doumaki, V. Grosomanidis, H. Yasuda, M. M. Admiraal, M. Van Assen, M. J. Van Putten, M. Tjepkema-Cloostermans, A. F. Van Rootselaar, J. Horn, F. Ragusa, A. Marudi, S. Baroni, A. Gaspari, E. Bertellini, A. Taha, T. Abdullah, S. Abdel Monem, S. Alcorn, S. McNeill, S. Russell, W. Eertmans, C. Genbrugge, I. Meex, J. Dens, F. Jans, C. De Deyne, J. Cholkraisuwat, N. Kongpolprom, B Avard, R Burns, A. Patarchi, T. Spina, H. Tanaka, N. Otani, S. Ode, S. Ishimatsu, J. Cho, J. B. Moon, C. W. Park, T. G. Ohk, M. C. Shin, M. H. Won, S. Dakova, Z. Ramsheva, K. Ramshev, J. Cho, J. B. Moon, C. W. Park, T. G. Ohk, M. C. Shin, J. Cho, J. B. Moon, C. W. Park, T. G. Ohk, M. C. Shin, A Marudi, S Baroni, A Gaspari, E Bertellini, G. Orhun, E. Senturk, P. E. Ozcan, S. Sencer, C. Ulusoy, E. Tuzun, F. Esen, R. Tincu, C. Cobilinschi, D. Tomescu, Z. Ghiorghiu, R. Macovei, M. Van Assen, M. M. Admiraal, M. J. Van Putten, M. Tjepkema-Cloostermans, A. F. Van Rootselaar, J. Horn, M. Fallenius, M. B. Skrifvars, M. Reinikainen, S. Bendel, R. Raj, M. Abu-Habsa, C. Hymers, A. Borowska, H. Sivadhas, S. Sahiba, S. Perkins, J. Rubio, J. A. Rubio, R. Sierra, S. English, M. Chasse, A. Turgeon, F. Lauzier, D. Griesdale, A. Garland, D. Fergusson, R. Zarychanski, A. Tinmouth, C. Van Walraven, K. Montroy, J. Ziegler, R. Dupont Chouinard, R. Carignan, A. Dhaliwal, C. Lum, J. Sinclair, G. Pagliarello, L. McIntyre, S. English, M. Chasse, A. Turgeon, F. Lauzier, D. Griesdale, A. Garland, D. Fergusson, R. Zarychanski, A. Tinmouth, C. Van Walraven, K. Montroy, J. Ziegler, R. Dupont Chouinard, R. Carignan, A. Dhaliwal, C. Lum, J. Sinclair, G. Pagliarello, L. McIntyre, T. Groza, N. Moreau, D. Castanares-Zapatero, P. Hantson, M. Carbonara, F. Ortolano, T. Zoerle, S. Magnoni, S. Pifferi, V. Conte, N. Stocchetti, L. Carteron, T. Suys, C. Patet, H. Quintard, M. Oddo, J. A. Rubio, J. Rubio, R. Sierra, V. Spatenkova, E. Pokorna, P. Suchomel, N. Ebert, J. Jancik, H. Rhodes, T. Bylinski, C. Hawthorne, M. Shaw, I. Piper, J. Kinsella, A. K. Kink, I. R. Rätsep, A. Boutin, L. Moore, M. Chasse, R. Zarychanski, F. Lauzier, S. English, L. McIntyre, J. Lacroix, D. Griesdale, P. Lessard-Bonaventure, A. F. Turgeon, A. Boutin, L. Moore, R. Green, P. Lessard-Bonaventure, M. Erdogan, M. Butler, F. Lauzier, M. Chasse, S. English, L. McIntyre, R. Zarychanski, J. Lacroix, D. Griesdale, P. Desjardins, D. A. Fergusson, A. F. Turgeon, B. Goncalves, B. Vidal, C. Valdez, A. C. Rodrigues, L. Miguez, G. Moralez, T. Hong, A. Kutz, P. Hausfater, D. Amin, T. Struja, S. Haubitz, A. Huber, B. Mueller, P. Schuetz, T. Brown, J. Collinson, C. Pritchett, T. Slade, M. Le Guen, S. Hellings, R. Ramsaran, A. Alsheikhly, T. Abe, L. Kanapeckaite, M. Abu-Habsa, R. Bahl, M. Q. Russell, K. J. Real, M. Abu-Habsa, R. M. Lyon, N. P. Oveland, J. Penketh, M. Mcdonald, F. Kelly, M. Alfafi, S. Alsolamy, W. Almutairi, B. Alotaibi, A. E Van den Berg, Y. Schriel, L. Dawson, I. A. Meynaar, H. Talaie, D. Silva, S. Fernandes, J. Gouveia, J. Santos Silva, J. Foley, A. Kaskovagheorgescu, D. Evoy, J. Cronin, J. Ryan, M. Huck, C. Hoffmann, J. Renner, P. Laitselart, N. Donat, A. Cirodde, J. V. Schaal, Y. Masson, A. Nau, T. Leclerc, O. Howarth, K. Davenport, P. Jeanrenaud, S. Raftery, P. MacTavish, H. Devine, J. McPeake, M. Daniel, J. Kinsella, T. Quasim, S. Alrabiee, A. Alrashid, S. Alsolamy, O. Gundogan, C. Bor, E. Akýn Korhan, K. Demirag, M. Uyar, F. Frame, C. Ashton, L. Bergstrom Niska, P. Dilokpattanamongkol, T. Suansanae, C. Suthisisang, S. Morakul, C. Karnjanarachata, V. Tangsujaritvijit, S. Mahmood, H. Al Thani, A. Almenyar, A. Vakalos, V. Avramidis, R. Sharvill, J. Penketh, S. E. Morton, Y. S. Chiew, C. Pretty, J. G. Chase, G. M. Shaw, R. Knafelj, P. Kordis, S. Patel, V. Grover, I. Kuchyn, K. Bielka, Z. Aidoni, V. Grosomanidis, K. Kotzampassi, G. Stavrou, B. Fyntanidou, S. Patsatzakis, C. Skourtis, S. D. Lee, K. Williams, I. D. Weltes, S. Berhane, C. Arrowsmith, C. Peters, S. Robert, J. Caldas, R. B. Panerai, T. G. Robinson, L. Camara, G. Ferreira, E. Borg-Seng-Shu, M. De Lima Oliveira, N. C. Mian, L. Santos, R. Nogueira, S. P. Zeferino, M. Jacobsen Teixeira, F. Galas, L. A. Hajjar, P. Killeen, M. McPhail, W. Bernal, J. Maggs, J. Wendon, T. Hughes, L. U. Taniguchi, E. M. Siqueira, J. M. Vieira Jr, L. C. Azevedo, A. N. Ahmad, M. Abu-Habsa, R. Bahl, E. Helme, S. Hadfield, R. Loveridge, J. Shak, C. Senver, R. Howard-Griffin, P. Wacharasint, P. Fuengfoo, N. Sukcharoen, R. Rangsin, D. Sbiti-Rohr, P. Schuetz, H. Na, S. Song, S. Lee, E. Jeong, K. Lee, M. Cooper, K. Milinis, G. Williams, E. McCarron, S. Simants, I. Patanwala, I. D. Welters, E. Zoumpelouli, E. A Volakli, V. Chrysohoidou, S. Georgiou, K. Charisopoulou, E. Kotzapanagiotou, V. Panagiotidou, K. Manavidou, Z. Stathi, M. Sdougka, N. Salahuddin, B. AlGhamdi, Q. Marashly, K. Zaza, M. Sharshir, M. Khurshid, Z. Ali, M. Malgapo, M. Jamil, A. Shafquat, M. Shoukri, M. Hijazi, T. Abe, S. Uchino, M. Takinami, N. R. Rangel Neto, S. Oliveira, F. Q. Reis, F. A. Rocha, G. Moralez, K. Ebecken, L. S. Rabello, M. F. Lima, R. Hatum, F. V. De Marco, A. Alves, J. E. Pinto, M. Godoy, P. E. Brasil, F. A. Bozza, J. I. Salluh, M. Soares, J. Krinsley, G. Kang, J. Perry, H. Hines, K. M. Wilkinson, C. Tordoff, B. Sloan, M. C. Bellamy, E. Moreira, F. Verga, M. Barbato, G. Burghi, M Soares, U. V. Silva, L. C. Azevedo, A. P. Torelly, J. M. Kahn, D. C. Angus, M. F. Knibel, P. E. Brasil, F. A. Bozza, J. I. Salluh, M. B. Velasco, D. M. Dalcomune, R. Marshall, T. Gilpin, A. Tridente, A. Raithatha, D. Mota, B. Loureiro, J. Dias, O. Afonso, F. Coelho, A. Martins, F. Faria, H. Al-Dorzi, H. Al Orainni, F. AlEid, H. Tlaygeh, A. Itani, A. Hejazi, Y. Arabi, S. Gaudry, J. Messika, J. D. Ricard, S. Guillo, B. Pasquet, E. Dubief, D. Dreyfuss, F. Tubach, C. Battle, K. James, P. Temblett, L. Davies, C. Battle, C. Lynch, S. Pereira, S. Cavaco, J. Fernandes, I. Moreira, E. Almeida, F. Seabra Pereira, M. Malheiro, F. Cardoso, I. Aragão, T. Cardoso, M. Fister, R. Knafelj, P. Muraray Govind, N. Brahmananda Reddy, R. Pratheema, E. D. Arul, J. Devachandran, M. B. Velasco, D. M. Dalcomune, R. Knafelj, M. Fister, N. Chin-Yee, G. D’Egidio, K. Thavorn, D. Heyland, K. Kyeremanteng, A. G. Murchison, K. Swalwell, J. Mandeville, D. Stott, I. Guerreiro, H. Devine, P. MacTavish, J. McPeake, T. Quasim, J. Kinsella, M. Daniel, C. Goossens, M. B. Marques, S. Derde, S. Vander Perre, T. Dufour, S. E. Thiessen, F. Güiza, T. Janssens, G. Hermans, I. Vanhorebeek, K. De Bock, G. Van den Berghe, L. Langouche, H. Devine, P. MacTavish, T. Quasim, J. Kinsella, M. Daniel, J. McPeake, B. Miles, S. Madden, H. Devine, M. Weiler, P. Marques, C. Rodrigues, M. Boeira, K. Brenner, C. Leães, A. Machado, R. Townsend, J. Andrade, P. MacTavish, J. McPeake, H. Devine, J. Kinsella, M. Daniel, R. Kishore, C. Fenlon, T. Quasim, T. Fiks, A. Ruijter, M. Te Raa, P. Spronk, Y. S. Chiew, P. Docherty, J. Dickson, E. Moltchanova, C. Scarrot, C. Pretty, G. M. Shaw, J. G. Chase, T. Hall, W. C. Ngu, J. M. Jack, P. Morgan, B. Avard, A. Pavli, X. Gee, C. Bor, E. Akin Korhan, K. Demirag, M. Uyar, M. Shirazy, A. Fayed, S. Gupta, A. Kaushal, S. Dewan, A. Varma, E. Ghosh, L. Yang, L. Eshelman, B. Lord, E. Carlson, E. Helme, R. Broderick, S. Hadfield, R. Loveridge, J. Ramos, D. Forte, F. Yang, P. Hou, J. Dudziak, J. Feeney, K. Wilkinson, K. Bauchmuller, K. Shuker, M. Faulds, A. Raithatha, D. Bryden, L. England, N. Bolton, A. Tridente, K. Bauchmuller, K Shuker, A Tridente, M Faulds, A Matheson, J. Gaynor, D Bryden, S South Yorkshire Hospitals Research Collaboration ᅟ, J. Ramos, B. Peroni, R. Daglius-Dias, L. Miranda, C. Cohen, C. Carvalho, I. Velasco, D. Forte, J. M. Kelly, A. Neill, G. Rubenfeld, N. Masson, A. Min, E. Boezeman, J. Hofhuis, A. Hovingh, R. De Vries, P. Spronk, G. Cabral-Campello, I. Aragão, T. Cardoso, M. Van Mol, M. Nijkamp, E. Kompanje, P. Ostrowski, A. Omar, K. Kiss, B. Köves, V. Csernus, Z. Molnár, Y. Hoydonckx, S. Vanwing, B. Stessel, A. Van Assche, L. Jamaer, J. Dubois, V. Medo, R. Galvez, J. P. Miranda, C. Stone, T. Wigmore, Y. Arunan, A. Wheeler, K. Bauchmuller, D. Bryden, Y. Wong, C. Poi, C. Gu, P. Molmy, N. Van Grunderbeeck, O. Nigeon, M. Lemyze, D. Thevenin, J. Mallat, J. Ramos, M. Correa, R. T. Carvalho, D. Forte, A. Fernandez, C. McBride, E. Koonthalloor, C. Walsh, A. Webber, M. Ashe, K. Smith, P. Jeanrenaud, A. Marudi, S. Baroni, F. Ragusa, E. Bertellini, E. A. Volakli, E. Chochliourou, M. Dimitriadou, A. Violaki, P. Mantzafleri, E. Samkinidou, O. Vrani, A. Arbouti, T. Varsami, M. Sdougka, J. A. Bollen, T. C. Van Smaalen, W. C. De Jongh, M. M. Ten Hoopen, D. Ysebaert, L. W. Van Heurn, W. N. Van Mook, K. Sim, A. Fuller, A. Roze des Ordons, P. Couillard, C. Doig, R. V. Van Keer, R. D. Deschepper, A. F. Francke, L. H. Huyghens, J. B. Bilsen, B. Nyamaizi, C. Dalrymple, A. Molokhia, A. Dobru, E. Marrinan, A. Ankuli, A. Molokhia, J. McPeake, R. Struthers, R. Crawford, H. Devine, P. Mactavish, T. Quasim, P. Morelli, M. Degiovanangelo, F. Lemos, V. MArtinez, F. Verga, J. Cabrera, G. Burghi, A. Rutten, S. Van Ieperen, S. De Geer, M. Van Vugt, E. Der Kinderen, A. Giannini, G Miccinesi, T Marchesi, E Prandi

**Affiliations:** 1 University of Western Ontario, London, Canada; 2 Hospital Dr Negrín, Las Palmas de GC, Spain; 3 Hospital San Jorge, Huesca, Spain; 4 Hospital Universitari del Mar, Barcelona, Spain; 5 Hospital Universitario de la Princesa, Madrid, Spain; 6 Hospital Clínico y Universitario de Valencia, Valencia, Spain; 7 Hospital Universitari Son Espases, Palma de Mallorca, Spain; 8 Medical Faculty of Istanbul, Istanbul University, Anesthesiology and Intensive Care, Istanbul, Turkey; 9 Medical Faculty of Istanbul, Physiology, Istanbul University, Istanbul, Turkey; 10 Institute of Experimental Medicine, Istanbul University, Neuroscience, Istanbul, Turkey; 11 Medical Faculty of Istanbul, Forensic Medicine, Istanbul University, Istanbul, Turkey; 12 Institute of Experimental Medicine, Immunology, Istanbul University, Istanbul, Turkey; 13 Ben-Gurion University of the Negev, Beer-Sheva, Israel; 14 Soroka Medical Center, Beer-Sheva, Israel; 15 Osaka University Graduate School of Medicine, Suita, Japan; 16 Nagasaki University Graduate School of Biomedical Sciences, Nagasaki, Japan; 17 Tokyo Medical University Hachioji Medical Center, Hachioji, Tokyo Japan; 18 National Defense Medical College, Tokorozawa, Saitama Japan; 19 Osaka University Graduate School of Medicine, Suita, Japan; 20 Hachiouji medical center, Tokyo medical university, Tokyo, Japan; 21 Kanazawa University, Kanazawa, Japan; 22 Kanazawa University Hospital, Kanazawa, Japan; 23 ThermoFisher, Hennigsdorf, Germany; 24 Institut für Klinische Chemie und Laboratoriumsmedizin, Mainz, Germany; 25 MVZ Labor Limbach Gruppe, Berlin, Germany; 26 Kantonsspital Aarau, Aarau, Switzerland; 27 King Faisal Specialist Hospital & Research Center, Riyadh, Saudi Arabia; 28 Saint Savvas Hospital, Athens, Greece; 29 Tokyo Medical University, Tokyo, Japan; 30 Tokyo Medical University, Hachioji Medical Center, Tokyo, Japan; 31 Hospital Virgen de la Victoria, Málaga, Spain; 32 St Helens and Knowsley NHS Trust, Merseyside, UK; 33 Maastricht University, Maastricht, Netherlands; 34 Center for Experimental and Molecular medicine, Amsterdam, Netherlands; 35 Academic Medical Center, Amsterdam, Netherlands; 36 NISCHR Haemostasis Biomarker Research Unit, Swansea, UK; 37 Hôpital Lariboisière, Paris, France; 38 Sphingotec, Berlin, Germany; 39 Hôpital Cochin, Paris, France; 40 Hôpital Saint-Antoine, Paris, France; 41 CHRU de Montpellier, Montpellier, France; 42 Hôpital Saint-Louis, Paris, France; 43 AP-HM, Marseille, France; 44 Hôpital Ambroise Paré, Paris, France; 45 Christiana Care Health System, Newark, USA; 46 Skane University Hospital, Lund University, Lund, Sweden; 47 Carol Davila University of Medicine and Pharmacy, Bucharest, Romania; 48 Hadassah-Hebrew University Medical Center, Jerusalem, Israel; 49 LeukoDx, Jerusalem, Israel; 50 Abionic SA, Lausanne, Switzerland; 51 Swiss Federal Laboratories (Empa), St. Gallen, Switzerland; 52 Pathophysiology of injury induced immunosuppression (PI3) Lab, Lyon 1 University / Hospices Civils de Lyon / bioMérieux, Lyon, France; 53 Negovsky V.A. Research Institute of General Reanimatology, Moscow, Russia; 54 Kantonsspital Aarau, Aarau, Switzerland; 55 University Hospital Basel, Basel, Switzerland; 56 UNIFESP, Sao Paulo, Brazil; 57 Albert Einstein Hospital, Sao Paulo, Brazil; 58 Sírio Libanês Hospital, Sao Paulo, Brazil; 59 Faculty of Medicine and Health Örebro University, Örebro, Sweden; 60 Department of Infectious Diseases, Karolinska University Hospital, Stockholm, Sweden; 61 Faculty of Medicine and Health, Örebro, Sweden; 62 Center for Infectious Medicine, Karolinska Institutet, Karolinska University Hospital, Stockholm, Sweden; 63 NISCHR Haemostasis Biomarker Research Unit, Swansea, UK; 64 NISCHR Haemostasis Biomarker Research Unit, Swansea, UK; 65 NISCHR Haemostasis Biomarker Research Unit, Swansea, UK; 66 Erasmus Medical Center, Rotterdam, Netherlands; 67 University Witten/Herdecke, Witten, Germany; 68 Maasstad Ziekenhuis, Rotterdam, Netherlands; 69 Intermountain Healthcare, Salt Lake City, USA; 70 Immunexpress, Seattle, USA; 71 Lewisham and Greenwich NHS Trust, London, UK; 72 Wirral trust, Merseyside, UK; 73 RLBUHT, Liverpool, UK; 74 Department of Emergency Medicine, Odense University Hospital, Odense C, Denmark; 75 Department of Respiratory Medicine, Odense University Hospital, Odense C, Denmark; 76 Department of Anaesthesiology and Intensive Care Medicine, Odense University Hospital, Odense C, Denmark; 77 Bucharest Clinical Emergency Hospital, Bucharest, Romania; 78 Fundeni Clinical Institute, Bucharest, Romania; 79 Amsterdam Medical Center, Amsterdam, Netherlands; 80 University Medical Center Utrecht, Utrecht, Netherlands; 81 Bakirkoy Dr.Sadi Konuk Training and Research Hospital, Istanbul, Turkey; 82 King Saud bin Abdulaziz University for Health Sciences and King Abdullah International Medical Research Center, Riyadh, Saudi Arabia; 83 Lewisham and Greenwich NHS Trust, London, UK; 84 Assaf Harofeh MC, Beer Yaakov, Israel; 85 Jena University Hopital, Jena, Germany; 86 Prince of Songkla University, Hat Yai, Thailand; 87 Division of Critical Care Medicine, Hat Yai, Thailand; 88 Glasgow Royal Infirmary, Glasgow, UK; 89 University Hospital and Faculty of Medicine Ostrava University, Ostrava, Czech Republic; 90 Institute of Biostatistics and analyses, Masaryk University, Brno, Czech Republic; 91 Università degli studi di Pavia, scuola di specialità: Anestesia e Rianimazione, Pavia, Italy; 92 UOC Anestesia e Rianimazione Ospedale Civile di Vigevano, AO Pavia, Vigevano, Italy; 93 Università degli studi di Pavia, Pavia, Italy; 94 Hospital O’horan, Mérida, Mexico; 95 Department of Anaesthesiology and Intensive Therapy, Medical University, Wroclaw, Poland; 96 AKPA, Waltham, USA; 97 St. Paul’s Hospital, Vancouver, Canada; 98 Fundeni Clinical Institute, Bucharest, Romania; 99 University of Medicine and Pharmacy “Carol Davila”, Bucharest, Romania; 100 Acedemisch Medisch Centrum, Amsterdam, Netherlands; 101 University Hospital of Greifswald, Greifswald, Germany; 102 Division of Infection Control and Prevention, Osaka University Graduate School of Medicine, Suita, Japan; 103 Department of Traumatology and Acute Critical Medicine, Osaka University Graduate School of Medicine, Suita, Japan; 104 Department of Emergency Medicine, Nagasaki University Graduate School of Biomedical Sciences, Nagasaki, Japan; 105 Hospital Clínico Universitario de Valladolid, Valladolid, Spain; 106 Jena University Hospital, Jena, Germany; 107 Medica Superspecialty Hospital, ?, West Bengal India; 108 Assaf Harofeh MC, Beer Yaakov, Israel; 109 DMC, Detroit, USA; 110 Wayne State University, Detroit, USA; 112 Scientific Institute of Public Health (WIV-ISP), Brussels, Belgium; 113 Ghent University, Ghent, Belgium; 114 Erasme University, Brussels, Belgium; 115 QualityLabs Bt GmbH, Nuremberg, Germany; 116 B.Braun Melsungen AG, Melsungen, Germany; 117 Dr. Roschke medical marketing GmbH, Cologne, Germany; 118 Bucharest Clinical Emergency Hospital, Bucharest, Romania; 119 Fundeni Clinical Institute, Bucharest, Romania; 120 Nakhonpathom hospital, Nakhonpathom, Thailand; 121 Lithuanian University of Health Sciences, Kaunas,, Lithuania; 122 University Hospital North Midlands, Stoke-on-Trent, UK; 123 EPS Charles-Nicolle, Bab Saadoun, Tunisia; 124 Dalin Tzu Chi Hospital, Buddhist Tzu Chi Medical Foundation, Chiayi County, Taiwan; 125 Academic Medical Center, University of Amsterdam, Amsterdam, Netherlands; 126 Academic Medical Center, University of Amsterdam, Center for Experimental and Molecular Medicine, Amsterdam, Netherlands; 127 Department of Intensive Care Medicine, University Medical Center Utrecht, Utrecht, Netherlands; 128 Department of Intensive Care Medicine, Academic Medical Center, University of Amsterdam, Amsterdam, Netherlands; 129 Department of Medical Microbiology, University Medical Center Utrecht, Utrecht, Netherlands; 130 Glasgow Royal Infirmary, Glasgow, UK; 131 NHS Greater Glasgow and Clyde, Glasgow, UK; 132 Health Protection Scotland, Glasgow, UK; 133 Public Health England, London, UK; 134 Fukuoka University Hospital, Fukuoka, Japan; 135 King Saud bin Abdulaziz University for Health Sciences, Riyadh, Saudi Arabia; 136 Toxicological Research Center, Department of Clinical Toxicology, Loghman-Hakim Hospital, Shahid Beheshti University of Medical Sciences, Tehran, Iran; 137 St. Elisabeth Hospital, Tilburg, Netherlands; 138 Mongi Slim University Hospital, La Marsa, Tunisia; 139 University of Athens, Medical School, Athens, Greece; 140 Korgialeneion Benakeion Hospital, Athens, Greece; 141 University of Thrace, Alexandroupolis, Greece; 142 Aghios Dimitrios Hospital, Thessaloniki, Greece; 143 Tzaneion Hospital, Piraeus, Greece; 144 G.Gennimatas General Hospital, Thessaloniki, Greece; 145 University of Ioannina, Ioannina, Greece; 146 G.Papanikolaou General Hospital, Thessaloniki, Greece; 147 University of Patras, Patras, Greece; 148 Hospital Universitario La Paz, Madrid, Spain; 149 Centro Hospitalar São João, Porto, Portugal; 150 PSMMC, Riyadh, Saudi Arabia; 151 UNIKLINIKUM JENA, JENA, Germany; 152 King Saud bin Abdulaziz University for Health Sciences, Riyadh, Saudi Arabia; 153 King Saud bin Abdulaziz University for Health Sciences and King Abdullah International Medical Research Center, Riyadh, Saudi Arabia; 154 King Fahad Medical City, Riyadh, Saudi Arabia; 155 Addenbrooke’s Hospital, Cambridge, UK; 156 University of Cambridge, Cambridge, UK; 157 Royal United Hospital, Bath, UK; 158 Hospital Clínico Universitario de Valladolid, Valladolid, Spain; 159 Salford Royal Hospital, London, UK; 160 Hennepin County Medical Center, Minneapolis, USA; 161 CHU Dupuytren, Limoges, France; 162 St Luc University Hospital, Brussels, Belgium; 163 CHUV, Lausanne, Switzerland; 164 Hospital Clinic of Barcelona, Barcelona, Spain; 165 Hospital Clínico San Carlos, Madrid, Spain; 166 Université François Rabelais and CHU Bretonneau, Tours, France; 167 Groupe Hospitalier Pitié-Salpêtrière, Paris, France; 168 MedImmune, Gaithersburg, USA; 169 Mongi Slim University Hospital, La Marsa, Tunisia; 170 Xanthi General Hospital, Xanthi, Greece; 171 Royal Infirmary of Edinburgh, Edinburgh, UK; 172 Health Protection Scotland, Glasgow, UK; 173 Western General Hospital, Edinburgh, UK; 174 Fondazione IRCCS Policlinico S. Matteo, University of Pavia, Pavia, Italy; 175 Centre Hospitalier Universitaire Dijon, Dijon, France; 176 Marmara University Pendik Teaching and Research Hospital, Istanbul, Turkey; 177 Xanthi General Hospital, Xanthi, Greece; 178 Sheffield Teaching Hospital NHS Foundation Trust, Sheffield, UK; 179 Groupe Hospitalier Pitié-Salpêtrière, Paris, France; 180 CHU Dupuytren, Limoges, France; 181 Hospital Clinic of Barcelona, Barcelona, Spain; 182 Hospital Clínico San Carlos, Madrid, Spain; 183 University College, London, UK; 184 MedImmune, Gaithersburg, USA; 185 Cairo University, Giza, Egypt; 186 University hospital Center of La Rabta, Tunis, Tunisia; 187 Marmara University Pendik Teaching and Research Hospital, Istanbul, Turkey; 188 V.A. Negovsky Research Institute of General Reanimatology, Moscow, Russia; 189 N.V. Sklifosofsky Institute of Emergency Medicine, Moscow, Russia; 190 NN Burdenko Main Military Hospital, Moscow, Russia; 191 Novartis Pharmaceuticals, San Carlos, USA; 192 Nektar Therapeutics, San Francisco, CA USA; 193 Hospital Clinic, Barcelona, Spain; 194 Academic Medical Center, Amsterdam, Netherlands; 195 Maine Medical Center, Portland, USA; 196 Medical University of Vienna, Vienna, Austria; 197 Toxicological Research Center, Department of Clinical Toxicology, Loghman-Hakim Hospital, Shahid Beheshti University of Medical Sciences, Tehran, Iran; 198 HIA Percy, Clamart, France; 199 Pherecydes Pharma, Romainville, France; 200 Hôpital Reine Astrid, Brussels, Belgium; 201 CHUV, Lausanne, Switzerland; 202 CHU Liege, Liege, Belgium; 203 CH Saint Jospeh Saint Luc, Lyon, France; 204 King Saud bin Abdulaziz University for Health Sciences, Riyadh, Saudi Arabia; 205 University hospital center of La Rabta, Tunis, Tunisia; 206 Hamad Medical Corporation, Doha, Qatar; 207 MUI, Innsbruck, Austria; 208 Academic Medical Centre, Amsterdam, Netherlands; 209 Academic Medical Center Amsterdam, Amsterdam, Netherlands; 210 Sanquin, Amsterdam, Netherlands; 211 KAT Hospital Athens, Kifisia, Greece; 212 ICU-B, KAT Hospital Kifisia, Athens, Greece; 213 Hennepin County Medical Center, Minneapolis, USA; 214 Hokkaido University Hospital, Sapporo, Japan; 215 Tohoku University Graduate School of Medicine, Sendai, Japan; 216 Tokyo Medical and Dental University Hospital of Medicine, Tokyo, Japan; 217 National Hospital Organization Disaster Medical Center, Tokyo, Japan; 218 Keio University School of Medicine, Tokyo, Japan; 219 Osaka University Graduate School of Medicine, Osaka, Japan; 220 Rinku General Medical Center, Osaka, Japan; 221 Kinki University Faculty of Medicine, Osaka, Japan; 222 Yokohama City University Graduate School of Medicine, Yokohama, Japan; 223 Faculty of Medicine, Fukuoka University, Fukuoka, Japan; 224 National Center For Global Health and Medicine, Tokyo, Japan; 225 Tokyo Women’s Medical University, Tokyo, Japan; 226 Odessa National Medical University, Odessa, Ukraine; 227 Hopital Sacré-Coeur de Montréal, Montreal, Canada; 228 Odessa National Medical University, Odessa, Ukraine; 229 Xanthi General Hospital, Xanth, Greece; 230 Maastricht UMC, Maastricht, Netherlands; 231 Second Faculty of Medicine, Charles University and University Hospital Motol, Prague, Czech Republic; 232 Sechenov First Moscow Stat Medical University, Moscow, Russia; 233 Sheba Medical Center, Tel-Hashomer, Israel; 234 Yokohama Municipal Citizen’s Hospital, Yokohama, Japan; 235 Hayama Heart Center, Hayama, Japan; 236 Yokohama City University Hospital, Yokohama, Japan; 237 Institute of Ageing and Chronic Disease, Liverpool, UK; 238 Institute of Infection and Global Health, Liverpool, UK; 239 Department of Haematology, Royal Liverpool University Hospital (RLUH), Liverpool, UK; 240 Hôpital du Sacré-Coeur de Montréal, Montreal, Canada; 241 Evelina London Children’s Hospital, London, UK; 242 St Thomas Hospital, London, UK; 243 G.Papanikolaou General Hospital, Thessaloniki, Greece; 244 Intensive Care Unit, University of Ferrara, Italy, Ferrara, Italy; 245 Department of Intensive Care, Erasme Hospital, Université Libre de Bruxelles, Bruxelles, Belgium; 246 Hippokration General Hospital Thessaloniki, Thessaloniki, Greece; 247 Morriston Hospital, Swansea, UK; 248 Central London School of Anaesthesia and Intensive Care Medicine, London, UK; 249 University of the Sunshine Coast, Maroochydore, Australia; 250 University of Edinburgh, Edinburgh, UK; 251 John Radcliffe Hospital, Oxford, UK; 252 Copenhagen University Hospital, Copenhagen, Denmark; 253 Peterborough NHS Trust, Peterborough, UK; 254 Aarhus University, Aarhus, Denmark; 255 Hospital de Sao Paulo, Sao Paulo, Brazil; 256 Royal Brompton & Harefield NHS Trust, London, UK; 257 Barts Heart Centre, London, UK; 258 Hamad medical corporation, Doha, Qatar; 259 G.Papanikolaou General Hospital, Thessaloniki, Greece; 260 University of Sao Paulo, Brazi, Sao Paulo, Brazil; 261 Juntendo University Hospital, Tokyo, Japan; 262 Saitama Medical Center, Jichi Medical University, Saitama, Japan; 263 Gunma University Hospital, Maebashi, Japan; 264 Yokohama City Minato Red Cross Hospital, Yokohama, Japan; 265 Tokyo Medical and Dental University Hospital of Medicine, Tokyo, Japan; 266 Hamad medical corporation, Doha, Qatar; 267 Research Institution for Cardiology, Tomsk, Russia; 268 Siberian State Medical University, Tomsk, Russia; 269 The Hillingdon Hospitals NHS Foundation Trust, Middlesex, UK; 270 University Hospital Rio Hortega, Valladolid, Spain; 271 Erasmus MC, Rotterdam, Netherlands; 272 Sheikh Khalifa Medical City, Abu Dhabi, United Arab Emirates; 273 Intensive Care Unit, St. Boniface Hospital, Verona, Italy; 274 Intensive Care Unit, St. Boniface Hospital, Verona, Italy; 275 University of Pittsburgh, Pittsburgh, USA; 276 Carnegie Mellon University, Pittsburgh, USA; 277 Valme University Hospital, Seville, Spain; 278 University Hospital Rio Hortega, Valladolid, Spain; 279 Erasmus MC, Rotterdam, Netherlands; 280 CHU Amiens, Amiens, France; 281 CH Beauvais, Beauvais, France; 282 Réanimation polyvalente, Le Havre, France; 283 CH Roubaix, Roubaix, France; 284 CH Cotentin, Cherbourg, France; 285 CHU Rouen, Rouen, France; 286 CHU Lille, Lille, France; 287 CHU Caen, Caen, France; 288 King Faisal Specialist Hospital & Research Center, Riyadh, Saudi Arabia; 289 CHU Amiens, Amiens, France; 290 University Medical Center Hamburg-Eppendorf, Hamburg, Germany; 291 Intensive Care Unit, St. Boniface Hospital, Verona, Italy; 292 Brugmann Hospital, Brussels, Belgium; 293 Hospital Universitario del Tajo, Aranjuez, Spain; 294 Hospital Universitario Príncipe de Asturias, Alcalá de Henares, Spain; 295 Hospital Universitario de San Carlos, Madrid, Spain; 296 Klinikum rechts der Isar, Technical University of Munich, Munich, Germany; 297 Department of Intensive Care Medicine, University Hospital Bern, Bern, Switzerland; 298 Institute of Clinical Sciences at the Sahlgrenska Academy, University of Gothenburg, Sahlgrenska University Hospital, Gothenburg, Sweden; 299 St George’s Healthcare NHS Trust, London, UK; 300 St George’s Healthcare NHS Trust, London, UK; 301 Karolinska Institutet, Stockholm, Sweden; 302 Karolinska Institutet, Stockholm, Sweden; 303 Wenzhou Medical University, Wenzhou, Zheijang China; 304 Department of Intensive Care, Austin Hospital, Melbourne, VIC Australia; 305 Hospital O’horan, Mérida, Mexico; 306 Policlinico Modena, Bologna, Italy; 307 Clinical Hospital Sveti Duh, Zagreb, Croatia; 308 St George’s Healthcare NHS Trust, London, UK; 309 The Canberra Hospital, Hughes, ACT Australia; 310 Australian National University Medical School, Canberra, Australia; 311 Lapeyronie University Hospital, Montpellier, France; 312 Brigham and Women’s Hospital, Boston, USA; 313 Trexin Medical, Chicago, USA; 314 University of Colorado and Denver Health, Denver, USA; 315 Beth Isreal Deaconess, Boston, USA; 316 Mongi Slim University Hospital, La Marsa, Tunisia; 317 Centro Hospitalar do Porto, Porto, Portugal; 318 University Hospital, Ghent, Belgium; 319 City Clinical Hospital 40, Yekaterinburg, Russia; 320 University Hospital Muenster, Muenster, Germany; 321 Marienhospital Osnabrück, Osnabrück, Germany; 322 Charité, University of Berlin, Berlin, Germany; 323 Sapienza University of Rome, Rome, Italy; 324 University Hospital of Greifswald, Greifswald, Germany; 325 University Hospital of Muenster, Muenster, Germany; 326 University Hospital of Greifswald, Greifswald, Germany; 327 Erasmus MC University Hospital Rotterdam, Rotterdam, Netherlands; 328 Medical Research Institute of New Zealand, Wellington, New Zealand; 329 Monash University, Melbourne, Australia; 330 Austin Hospital, Melbourne, Australia; 331 Wellington Regional Hospital, Wellington, New Zealand; 332 Medical Research Institute of New Zealand, Wellington, New Zealand; 333 Australian and New Zealand Intensive Care Research Centre, Melbourne, Australia; 334 Austin Hospital, Melbourne, Australia; 335 Princess of Wales Hospital, Bridgend, UK; 336 Imperial College London, London, UK; 337 Glan Clwyd Hospital, Bodelwyddan, UK; 338 University Hospital Galway, Galway, Ireland; 339 Albert Schweitzer State Hospital, Rio de Janeiro, Brazil; 340 Tan Tock Seng Hospital, Singapore, Singapore; 341 National Neuroscience Institute, Singapore, Singapore; 342 Erasmus Medical Centre, Rotterdam, Netherlands; 343 Centro Hospitalar Tamega e Sousa, Penafiel, Portugal; 344 Centro Hospitalar do Porto, Porto, Portugal; 345 Unidade Local de Saude do Alto Minho, Viana do Castelo, Portugal; 346 South Tees NHS Trust, Middlesbrough, UK; 347 Ikazia Hospital, Rotterdam, Netherlands; 348 Erasmus MC, Rotterdam, Netherlands; 349 Sheffiled Teaching Hospitals, Sheffield, UK; 350 Whiston Hospital, St Helens & Knowsley, UK; 351 University Hospital Duesseldorf, Düsseldorf, Germany; 352 Charite University Hospital, Berlin, Germany; 353 Royal London Hospital, London, UK; 354 Aurelia and European Hospital, Rome, Italy; 355 Istanbul University Cerrahpasa Medical School, Istanbul, Turkey; 356 Danube University Krems, Krems, Austria; 357 University Hospital St. Poelten, St. Poelten, Austria; 358 Teaching Hospital Policlinico S.Orsola-Malpighi, Bologna, Italy; 359 Department of Nephrology, Dialysis, Hypertension, Bologna, Italy; 360 Science and Technology Park for Medicine, Mirandola, Italy; 361 Aferetica, Bologna, Italy; 362 San Marco Hospital, Zingonia, Italy; 363 CytoSorbents, Monmouth Junction, USA; 364 Klinikum Emden, Emden, Germany; 365 University Hospital Eppendorf, Hamburg, Germany; 366 Aurelia Hospital /European Hospital, Rome, Italy; 367 Fukuoka University hospital, Fukuoka, Japan; 368 King Faisal Specialist Hospital and Research Center, Riyadh, Saudi Arabia; 369 GPR Klinikum Ruesselsheim, Ruesselsheim, Hessen Germany; 370 Hospital O’horan, Mérida, Mexico; 371 Jikei University School of Medicine, Tokyo, Japan; 372 Kyoto University, Kyoto, Japan; 373 Osaka University, Osaka, Japan; 374 Guy’s & St Thomas’ NHS Foundation Trust, London, UK; 375 King’s College, London, UK; 376 Hospital de Santa Maria, Lisbon, Portugal; 377 ErasmusMC, Rotterdam, Netherlands; 378 ErasmusMC, Rotterdam, Netherlands; 379 Gazi University Medical Faculty, Ankara, Turkey; 380 Gazi University School of Medicine, Critical Care Fellowship Programme, Ankara, Turkey; 381 Gazi University School of Medicine Biochemistry Department, Ankara, Turkey; 382 Fundación Clínica Medica Sur, Mexico City, Mexico; 383 Lund University and Skane University Hospital, Lund, Sweden; 384 CHU Amiens, Amiens, France; 385 Radboudumc, Nijmegen, Netherlands; 386 Erasme Hospital, Université Libre de Bruxelles, Brussels, Belgium; 387 The Royal Surrey County Hospital, Guildford, UK; 388 Royal National Orthopaedic Hospital, Middlesex, UK; 389 Barts Health NHS Trust, London, UK; 390 Queen Mary University of London, London, UK; 391 Asan medical center, Seoul, South Korea; 392 Barts Health NHS Trust, London, UK; 393 Queen Mary University of London, London, UK; 394 Hamad medical corporation, Doha, Qatar; 395 Barts Health NHS Trust, London, UK; 396 Queen Mary University of London, London, UK; 397 Trakya Univ, Edirne, Turkey; 398 General Directorate of Health Services, Ministry of Health, Ankara, Turkey; 399 AnkaraNKARA Research and Training Hospital, Ankara, Turkey; 400 Department of Biostatistics, Faculty of Medicine, Ankara University, Ankara, Turkey; 401 Ankara Research and Training Hospital, Ankara, Turkey; 402 MC Into-Sana, Odessa, Ukraine; 403 Glan Clwyd Hospital, Rhyl, UK; 404 Fondazione IRCCS Ca’ Granda, Maggiore Policlinico Hospital, Milan, Italy; 405 University of Ferrara, Sant’Anna Hospital, Ferrara, Italy; 406 Aerogen, Galway, Ireland; 407 Kaplan Medical Centre, Rehovot, Israel; 408 West Virginia University, ?, West Virginia USA; 409 Rihard Knafelj, Ljubljana, Slovenia; 410 Corporació Sanitària i Universitària Parc Taulí, Universitat Autònoma de Barcelona, CIBER Enfermedades Respiratorias, Sabadell, Barcelona, Spain; 411 Dalhousie University, Halifax, Canada; 412 Ernst-Moritz-Arndt-Universität, Greifswald, Germany; 413 Ghent University Hospital, Gent, Belgium; 414 Universidade de São Paulo, São Paulo, Brazil; 415 Hospital La Paz, Madrid, Spain; 416 Institute of Mother and Child, Chisinau mun., Moldova; 417 Institute of Emergency Medicine, Chisinau mun., Moldova; 418 State University of Medicine and Pharmacy, Chisinau mun., Moldova; 419 Aerogen, Galway, Ireland; 420 Aerogen, Galway, Ireland; 421 Cardarelli Hospital, Naples, Italy; 422 San Paolo Hospital, Naples, Italy; 423 Cardarelli Hospital, Naples, Italy; 424 National University of Ireland, Galway, Galway City, Ireland; 425 Mount Sinai Hospital, Toronto, Canada; 426 Centro Hospitalar São João, Porto, Portugal; 427 Gazi University School of Medicine Respiratory Medicine and Critical Care Department, Ankara, Turkey; 428 Gazi University School of Medicine, Internal Medicine Critical Care Department, Ankara, Turkey; 429 Gazi University School of Medicine, Geriatrics Department, Ankara, Turkey; 430 Gazi University School of Medicine Respiratory Medicine and Critical Care Department, Ankara, Turkey; 431 Chulalongkorn University, Bangkok, Thailand; 432 Carmel, Lady Davis Medical Center, Haifa, Israel; 433 Duke University, Durham, USA; 434 Mayo Clinic, Rochester, USA; 435 St Helens and Knoowsley, Prescot, UK; 436 Royal Infirmary, Edinburgh, UK; 437 Alexandria Universitry General Hospital, Alexandria, Egypt; 438 University Hospital North Middlands, Stoke On Trent, UK; 439 Mongi Slim University Hospital, La Marsa, Tunisia; 440 Medica Sur, Mexico City, Mexico; 441 Instituto Nacional de Rehabilitacion, Mexico City, Mexico; 442 University of Padua, Padua, Italy; 443 Sant Antony hospital, Padua, Italy; 444 HCMC, Minneapolis, USA; 445 University of Minnesota, Minneapolis, USA; 446 Viecuri Medical Center, Venlo, Netherlands; 447 University of Hawaii, John A. Burns School of Medicine, Honolulu, USA; 448 Respiratory Motion Inc., Waltham, USA; 449 University of Hawaii, John A. Burns School of Medicine, Honolulu, USA; 450 Respiratory Motion Inc., Waltham, USA; 451 Massachusetts General Hospital, Boston, USA; 452 University of Hawaii, John A. Burns School of Medicine, Honolulu, USA; 453 Respiratory Motion Inc., Waltham, USA; 454 Massachusetts General Hospital, Boston, USA; 455 AO Desio e Vimercate, Vimercate, Italy; 456 AO Crema, Crema, Italy; 457 University of Milan-Bicocca, Monza, Italy; 458 UT Health Science Center at Houston, Houston, TX USA; 459 University of Hawaii, John A. Burns School of Medicine, Honolulu, HI USA; 460 Respiratory Motion Inc., Waltham, MA USA; 461 Massachusetts General Hospital, Boston, MA USA; 462 Swisstom AG, Landquart, Switzerland; 463 Kliniken der Stadt Köln, Pneumology and Critical Care Medicine, Witten / Herdecke University, Ostmerheimer Str. 200, 51109 Cologne, Germany; 464 Swisstom AG, Landquart, Switzerland; 465 Kliniken der Stadt Köln, Pneumology and Critical Care Medicine, Witten/ Herdecke University, Ostmerheimer Str. 200, 51109 Cologne, Germany; 466 ARDS and ECMO Center Köln-Merheim, Cologne, Germany; 467 Swisstom, Chur, Switzerland; 468 Sahlgrenska Univ Hospital, Göteborg, Sweden; 469 Second University of Naples, Naples, Italy; 470 San Paolo Hospital, Naples, Italy; 471 University of Sassari, Sassari, Italy; 472 Cardarelli Hospital, Naples, Italy; 473 University of Saerno, Salerno, Italy; 474 East Surrey Hospital, Surrey and Sussex NHS Trust, Surrey, UK; 475 Mount Sinai Hospital and University of Toronto, Toronto, Canada; 476 University Health Network-Toronto General Hospital and Univeristy of Toronto, Toronto, Canada; 477 University of Cambridge, Cambridge, UK; 478 Papworth Hospital, Cambridge, UK; 479 King’s College Hospital NHS Foundation Trust, London, UK; 480 Meijer Heart & Vascular Institute, Grand Rapids, USA; 481 Glenfield Hospital, UHL, Leicester, UK; 482 National University Hospital, Singapore, Singapore; 483 National University Singapore, Singapore, Singapore; 484 Mayo Clinic, Phoenix, AZ USA; 485 University of Pittsburgh, Pittsburgh, PA USA; 486 Vall D’Hebron University Hospital, Barcelona, Spain; 487 Universitat Autonoma de Barcelona, Barcelona, Spain; 488 V.A. Negovsky Research Institute of General Reanimatology, Moscow, Russia; 489 NN Burdenko Main Military Hospital, Moscow, Russia; 490 George P. Livanos and Marianthi Simou Laboratories, Athens, Greece; 491 University of Patras, Rio, Achaia Greece; 492 University of Ferrara, Ferrara, Italy; 493 University of Bari, Bari, Italy; 494 CHU HJRA, Antananarivo, Madagascar; 495 Réanimation médicale, Centre Hospitalier Universitaire, Amiens, France; 496 Cardio-Pulmonary Department, Pulmonary Division, Heart Institute (Incor), University of São Paulo, São Paulo, Brazil; 497 CHU Amiens, Amiens, France; 498 Royal Brompton Hospital, London, UK; 499 Musashino Red Cross Hospital, Tokyo, Japan; 500 JSEPTIC Clinical Trial Group, Tokyo, Japan; 501 Centro Hospitalar do Porto, Porto, Portugal; 502 University of Liverpool, Liverpool, UK; 503 Royal Liverpool University Hospital, Liverpool, UK; 504 Dalin Tzu Chi Hospital, Buddhist Tzu Chi Medical Foundation, Chiayi County, Taiwan; 505 Hospital Clínico Universitario de Santiago de Compostela, Santiago de Compostela, Spain; 506 MUMC, Maastricht, Netherlands; 507 Royal Liverpool University Hospital, Liverpool, UK; 508 Vilnius University Hospital Santariskiu Clinics, Vilnius, Lithuania; 509 Vilnius University, Faculty of Medicine, Vilnius, Lithuania; 510 Mercy University Hospital, Cork, Ireland; 511 Hospital Meridional S.A., Cariacica, Brazil; 512 Geneva Universtiy Hospital, Geneva, Switzerland; 513 Medical University Department, Kantonsspital Aarau, Aarau, Switzerland; 514 Department of Endocrinology, Diabetes and Clinical Nutrition, University Hospital Bern, Bern, Switzerland; 515 Jessa Ziekenhuis, Hasselt, Belgium; 516 Centro Hospitalar do Porto, Porto, Portugal; 517 Unidade Local de Saude do Alto Minho, Viana do Castelo, Portugal; 518 Unidade de Saude Local de Castelo Branco, Castelo Branco, Portugal; 519 Centro Hospitalar e Universitario de Coimbra, Coimbra, Portugal; 520 Centro Hospitalar de Vila Nova de Gaia, Vila Nova de Gaia, Portugal; 521 Centro Hospitalar do Algarve, Faro, Portugal; 522 Instituto Portugues de Oncologia do Porto, Porto, Portugal; 523 Faculdade de Ciencias da Nutricao e Alimentacao da Universidade do Porto, Porto, Portugal; 524 Centro Hospitalar do Porto, Porto, Portugal; 525 Faculdade de Ciencias da Nutrição e Alimentação da Universidade do Porto, Porto, Portugal; 526 Centro Hospitalar Tamega e Sousa, Penafiel, Portugal; 527 Instituto Português de Oncologia do Porto, Porto, Portugal; 528 Unidade Local de Saude do Alto Minho, Viana do Castelo, Portugal; 529 Centro Hospitalar e Universitário de Coimbra, Coimbra, Portugal; 530 Unidade de Saude Local de Castelo Branco, Castelo Branco, Portugal; 531 Centro Hospitalar do Algarve, Faro, Portugal; 532 Centro Hospitalar do Baixo Vouga, Aveiro, Portugal; 533 Centro Hospitalar de Vila Nova de Gaia, Vila Nova de Gaia, Portugal; 534 Nottingham University Hospital NHS Trust, Nottingham, UK; 535 Prince of Songkla University, Songkla, Thailand; 536 Kantonsspital Aarau, Aarau, Switzerland; 537 Université de Sherbrooke, Sherbrooke, Canada; 538 University Hospital, Montevideo, Uruguay; 539 Bucharest Clinical Emergency Hospital, Bucharest, Romania; 540 Fundeni Clinical Institute, Bucharest, Romania; 541 University Hospital, Montevideo, Uruguay; 542 Université de Sherbrooke, Sherbrooke, Canada; 543 Queen’s University, Kingston, Canada; 544 University Medical Center Schleswig-Holstein, Kiel, Germany; 545 University of Jena, Jena, Germany; 546 Université de Sherbrooke, Sherbrooke, Canada; 547 Queen’s University, Kingston, Canada; 548 University Hospital, Montevideo, Uruguay; 549 Dalin Tzu Chi Hospital, Buddhist Tzu Chi Medical Foundation, Chiayi County, Taiwan; 550 Centro Hospitalar do Porto, Porto, Portugal; 551 Aida Hospital, Fukushima, Japan; 552 Keiyo Hospital, Tokyo, Japan; 553 Shisei Hospital, Saitama, Japan; 554 Prince of Songkla University, Hat Yai City, Songkhla Province Thailand; 555 Centro Hospitalar do Porto, Porto, Portugal; 556 Faculdade de Ciencias da Nutrição e Alimentação da Universidade do Porto, Porto, Portugal; 557 Centro Hospitalar Tamega e Sousa, Penafiel, Portugal; 558 Centro Hospitalar e Universitário de Coimbra, Coimbra, Portugal; 559 Instituto Português de Oncologia do Porto, Porto, Portugal; 560 Unidade Local de Saude do Alto Minho, Viana do Castelo, Portugal; 561 Centro Hospitalar Baixo Vouga, Aveiro, Portugal; 562 Unidade de Saude Local de Castelo Branco, Castelo Branco, Portugal; 563 Centro Hospitalar do Algarve, Faro, Portugal; 564 Centro Hospitalar de Vila Nova de Gaia, Vila Nova de Gaia, Portugal; 565 Department of Cardiothoracic Surgery, George Papanikolaou General Hospital, Thessaloniki, Greece; 566 Gülhane Military Medical Academy, Ankara, Turkey; 567 Ankara Mevki Military Hospital, Ankara, Turkey; 568 St Bartholomew’s Hospital, London, UK; 569 Aristotle Medical School, Thessaloniki, Greece; 570 Tokyo Medical University Hachiosi Medical Center, Tokyo, Japan; 571 Hôpital Louis Mourier, Colombes, France; 572 Laboratoire ILUMENS, Paris, France; 573 Institut de Hauts de Seine, Nanterre, France; 574 University Hospital Lewisham, London, UK; 575 Queen Elizabeth Hospital, London, UK; 576 Minet Green Health Practice, London, UK; 577 Department of Public Health, University of Liège, Liège, Belgium; 578 Department of Emergency Medicine, University Hospital of Liège, Liège, Belgium; 579 Department of Medical Biostatistics, University of Liège, Liège, Belgium; 580 University Hospital, Philipps University Marburg, Marburg, Germany; 581 Chulalongkorn University, Bangkok, Thailand; 582 Oslo University Hospital, Oslo, Norway; 583 William Harvey Hospital, Ashford, UK; 584 Kansai Medical University Takii Hospital, Moriguchi, Japan; 585 Aristotle Medical School, Thessaloniki, Greece; 586 Japanese Red Cross Musashino Hospital, Tokyo, Japan; 587 Academic Medical Center, Amsterdam, Netherlands; 588 Medisch Spectrum Twente, Enschede, Netherlands; 589 University Modena, Modena, Italy; 590 Nuovo Ospedale Civile Sant’Agostino Estense, Modena, Italy; 591 Faculty of Medicine Alexandria University, Alexandria, Egypt; 592 Victoria Hospital, Kirkcaldy, UK; 593 Ziekenhuis Oost-Limburg, Genk, Belgium; 594 Chulalongkorn University, Bangkok, Thailand; 595 The Canberra Hospital, Hughes, ACT Australia; 596 ANU Medical School, Canberra, Australia; 597 “Spirito Santo” Hospital, Pescara, Italy; 598 St Lukes International Hospital, Akashi-Chou Chuo-Ku, Japan; 599 Kangwon National University, Chuncheonsi, South Korea; 600 Military Medical Academy, Sofia, Bulgaria; 601 Kangwon National University, Chuncheonsi, South Korea; 602 Kangwon National University, Chuncheonsi, South Korea; 603 Nuovo Ospedale Civile Sant’Agostino Estense, Modena, Italy; 604 University Modena, Modena, Italy; 605 Istanbul University, Medical Faculty of Istanbul, Anesthesiology and Intensive Care, Istanbul, Turkey; 606 Istanbul University, Medical Faculty of Istanbul, Department of Neuroradiology, Istanbul, Turkey; 607 Istanbul University, Institute of Experimental Medicine, Neuroscience, Istanbul, Turkey; 608 Bucharest Clinical Emergency Hospital, Bucharest, Romania; 609 Fundeni Clinical Institute, Bucharest, Romania; 610 Academic Medical Center, Amsterdam, Netherlands; 611 Medisch Spectrum Twente, Enschede, Netherlands; 612 Helsinki University Hospital, Helsinki, Finland; 613 North Karelia Central Hospital, Joensuu, Finland; 614 Kuopio University Hospital, Kuopio, Finland; 615 King’s College Hospital, London, UK; 616 Hospital Universitario Puerta del Mar, Cadiz, Spain; 617 Hospital Infanta Cristina, Badajoz, Spain; 618 The Ottawa Hospital, Ottawa, Canada; 619 Ulaval, Quebec City, Canada; 620 UBC, Vancouver, Canada; 621 UManitoba, Winnipeg, Canada; 622 OHRI, Ottawa, Canada; 623 The Ottawa Hospital, Ottawa, Canada; 624 Ulaval, Quebec City, Canada; 625 UBC, Vancouver, Canada; 626 UManitoba, Winnipeg, Canada; 627 Ottawa Hospital Research Institute, Ottawa, Canada; 628 Cliniques St Luc, Université catholique de Louvain, Brussels, Belgium; 629 Fondazione IRCCS Ca’ Granda Ospedale Maggiore Policlinico, Milan, Italy; 630 Milan University, Milan, Italy; 631 CHUV, Lausanne, Switzerland; 632 CHU de Nice, Nice, France; 633 Hospital Infanta Cristina, Badajoz, Spain; 634 Hospital Universitario Puerta del Mar, Cadiz, Spain; 635 Regional Hospital, Liberec, Czech Republic; 636 Institute of Experimental Medicine, Prague, Czech Republic; 637 Hennepin County Medical Center, Minneapolis, USA; 638 University of Glasgow, Glasgow, UK; 639 Institute of Neurological Sciences, NHSGGC, Glasgow, UK; 640 Department of Clinical Physics and Bioengineering, NHSGGC, Glasgow, UK; 641 Academic Unit of Anaesthesia, Pain and Critical Care Medicine, University of Glasgow, Glasgow, UK; 642 Smartimplant Ltd., Tallinn, Estonia; 643 NEMC, Tallinn, Estonia; 644 Universite Laval, Quebec, Canada; 645 University of Manitoba, Winnipeg, Canada; 646 Ottawa Hospital Research Institute, Ottawa, Canada; 647 Universite de Montreal, Montreal, Canada; 648 University of British Columbia, Vancouver, Canada; 649 Universite Laval, Quebec, Canada; 650 Dalhousie University, Halifax, Canada; 651 Ottawa Hospital Research Institute, Ottawa, Canada; 652 University of Manitoba, Winnipeg, Canada; 653 Universite de Montreal, Montreal, Canada; 654 University of British Columbia, Vancouver, Canada; 655 Hospital Estadual Getulio Vargas, Rio de Janeiro, Brazil; 656 Yonsei University College of Medicine, Seoul, South Korea; 657 Kantonsspital Aarau, Aarau, Switzerland; 658 Emergency Department, Groupe Hospitalier Pitié-Salpêtrière, Paris, France; 659 Morton Plant Hospital, Clearwater, USA; 660 Department of Laboratory Medicine, Kantonsspital Aarau, Aarau, Switzerland; 661 Royal Cornwall Hospital Trust, Truro, UK; 662 Manchester Royal Infirmary, Manchester, UK; 663 Hamad Medical Corporation, Doha, Qatar; 664 University of Tsukuba, Tsukuba Medical Center Hospital, Tsukuba, Japan; 665 King’s College Hospital, London, UK; 666 Kent, Surrey & Sussex Air Ambulance Trust, Kent, UK; 667 Prometheus Delta-Tech, Herefordshire, UK; 668 King’s College Hospital, London, UK; 669 Stavanger University Hospital, Stavanger, Norway; 670 Royal United Hospital, Bath, UK; 671 Aseer Central Hospital, Abha, Saudi Arabia; 672 King Saud bin Abdulaziz University for Health Sciences and King Abdullah International Medical Research Center, Riyadh, Saudi Arabia; 673 King Fahad Medical City, Riyadh, Saudi Arabia; 674 HagaZiekenhuis, Den Haag, Netherlands; 675 Reinier de Graaf Gasthuis, Delft, Netherlands; 676 Toxicological Research Center, Department of Clinical Toxicology, Loghman-Hakim Hospital, Shahid Beheshti University of Medical Sciences, Tehran, Iran; 677 Santa Maria University Hospital, Lisboa, Portugal; 678 St. Vincent’s University Hospital, Dublin 4, Ireland; 679 Percy Military Teaching Hospital, Clamart, France; 680 Whiston Hospital, Prescot, UK; 681 Glasgow Royal Infirmary, Glasgow, UK; 682 King Abdulaziz Medical City, National Guard Hospital, Riyadh, Saudi Arabia; 683 Department of Management, College of Business Administration, King Saud University, Saudi Arabia, Riyadh, Saudi Arabia; 684 King Saud bin Abdulaziz University for Health Sciences and King Abdullah International Medical Research Center, Riyadh, Saudi Arabia; 685 Ege University Hospital, Izmir, Turkey; 686 Katip Celebi University, Health Sciences Faculty, Izmir, Turkey; 687 Milton Keynes University Hospital NHS Foundation Trust, Milton Keynes, UK; 688 Faculty of Pharmacy, Mahidol University, Bangkok, Thailand; 689 Faculty of Medicine, Ramathibodi Hospital, Mahidol University, Bangkok, Thailand; 690 Specialist Anesthesia, Doha, Qatar; 691 Xanthi General Hospital, Xanthi, Greece; 692 Royal United Hospital, Bath, UK; 693 University of Canterbury, Christchurch, New Zealand; 694 Christchurch Hospital, Christchurch, New Zealand; 695 Rihard Knafelj, Ljubljana, Slovenia; 696 Clinical Center Ljubljana, Ljubljana, Slovenia; 697 The Royal Marsden Hospital, London, UK; 698 Bogomolets National Medical University, Kiev, Ukraine; 699 Aristotle Medical School, Thessaloniki, Greece; 700 Royal Liverpool Intensive Care Unit, Liverpool, UK; 701 University of Liverpool, Liverpool, UK; 702 Homerton University Hospital, London, UK; 703 University of Sao Paulo, Sao Paulo, Brazil; 704 University of Leicester, Leicester, UK; 705 King’s College Hospital, London, UK; 706 Research and Education Institute, Sao Paulo, Brazil; 707 King’s College Hospital, London, UK; 708 Ipswich Hospital NHS Trust, England, UK, Ipswich, UK; 709 Phramongkutklao Hospital, Bangkok, Thailand; 710 Phramongkutklao College of Medicine, Bangkok, Thailand; 711 Kantonsspital Aarau, Aarau, Switzerland; 712 Pusan National University Hospital, Busan, South Korea; 713 Royal Liverpool University Hospital, Liverpool, UK; 714 University of Liverpool, Liverpool, UK; 715 PICU, Hippokration General Hospital, Thessaloniki, Greece; 716 King Faisal Specialist Hospital & Research Centre, Riyadh, Saudi Arabia; 717 The Jikei University School of Medicine, Tokyo, Japan; 718 Albert Schweitzer State Hospital, Rio de Janeiro, Brazil; 719 PPG Internal Medicine, Federal University of Rio de Janeiro, Rio de Janeiro, Brazil; 720 Hospital Esperanca Recife, Recife, Brazil; 721 Hospital Total Cor, Rio de Janeiro, Brazil; 722 Hospital viValle, São José dos Campos, Brazil; 723 Hospital Rios DOr, Rio de Janeiro, Brazil; 724 Hospital Norte DOr, Rio de Janeiro, Brazil; 725 Hospital Esperanca Olinda, Olinda, Brazil; 726 DOr Institute for Research and Education - IDOR, Rio de Janeiro, Brazil; 727 Stamford Hospital, Stamford, USA; 728 Darent Valley Hospital, Dartford, UK; 729 William Harvey Hospital, Ashford, UK; 730 St James’s University Hospital, Leeds, UK; 731 Hospital Maciel, Montevideo, Uruguay; 732 DOr Institute for Research and Education - IDOR, Rio de Janeiro, Brazil; 733 Hospital de Cancer de Barretos, Barretos, Brazil; 734 Hospital Sírio Libanês, Sao Paulo, Brazil; 735 Sta. Casa de Porto Alegre, Porto Alegre, Brazil; 736 University of Pittsburgh Medical Center, Pittsburgh, USA; 737 Hospital Sao Lucas, Rio de Janeiro, Brazil; 738 Hospital Meridional S.A., Cariacica, Brazil; 739 Sheffield Teaching Hospitals, Sheffield, UK; 740 Whiston Hospital, St Helens & Knowsley, UK; 741 Centro Hospitalar e Universitário de Coimbra, Coimbra, Portugal; 742 Instituto Português de Oncologia Francisco Gil - Porto, Porto, Portugal; 743 King Saud bin Abdulaziz University for Health Sciences, Riyadh, Saudi Arabia; 744 Hôpital Louis Mourier, Colombes, France; 745 Hôpital Bichat, Paris, France; 746 Morriston Hospital, Swansea, UK; 747 Abertawe Bro Morgannwg University Health Board, Swansea, UK; 748 Centro Hospitalar Porto, Porto, Portugal; 749 Rihard Knafelj, Ljubljana, Slovenia; 750 Apollo Speciality Hospital - OMR, Chennai, India; 751 Hospital Meridional S.A., Cariacica, Brazil; 752 Rihard Knafelj, Ljubljana, Slovenia; 753 The Ottawa Hospital, Ottawa, Canada; 754 Kingston General Hospital, Kingston, Canada; 755 Milton Keynes Hospital, Milton Keynes, UK; 756 Imperial College, London, UK; 757 Buckinghamshire Healthcare NHS Trust, Aylesbury, UK; 758 IPO -Porto, Porto, Portugal; 759 Glasgow Royal Infirmary, Glasgow, UK; 760 KU Leuven, Leuven, Belgium; 761 Glasgow Royal Infirmary, Glasgow, UK; 762 Glasgow Royal Infirmary, Glasgow, UK; 763 Ernesto Dornelles Hospital, Porto Alegre, Brazil; 764 Glasgow Royal Infirmary, Glasgow, UK; 765 Gelre Ziekenhuizen, Apeldoorn, Netherlands; 766 University of Canterbury, Christchurch, New Zealand; 767 Christchurch Hospital, Christchurch, New Zealand; 768 Surrey and Sussex NHS Trust, Redhill, UK; 769 The Canberra Hospital, Hughes, ACT Australia; 770 ANU Medical School, Canberra, Australia; 771 Ege University School of Medicine, Izmir, Turkey; 772 katip Celebi University, Izmir, Turkey; 773 Alexandria University Faculty of medicine, Alexandria, Egypt; 774 Fortis Escorts Heart Institute, New Delhi, India; 775 FMRI, Gurgaon, India; 776 Philips Research North America, Cambridge, USA; 777 King’s College Hospital NHS Foundation Trust, London, UK; 778 Hospital Sao Rafael, Salvador, Brazil; 779 Hospital das Clinicas, Sao Paulo, Brazil; 780 Brigham and Women’s Hospital, Boston, USA; 781 St Helens and Knowsley, Liverpool, UK; 782 STH, Sheffield, UK; 783 Sheffield Teaching Hospitals, Sheffield, UK; 784 Whiston Hospital, Prescot, UK; 785 Freeman Hospital, Newcastle upon Tyne, UK; 786 Hospital Sao Rafael, Salvador, Brazil; 787 Hospital das Clinicas, Sao Paulo, Brazil; 788 Hospital Nove de Julho, Sao Paulo, Brazil; 789 University Hospital Coventry and Warwickshire, Coventry, UK; 790 Sunnybrook Health Sciences Centre, Toronto, Canada; 791 NHS Scotland, Glasgow, UK; 792 Royal College of Surgeons of Ireland, Dublin, Ireland; 793 University Leiden, Leiden, Netherlands; 794 Gelre Hospitals, Apeldoorn, Netherlands; 795 VU University Amsterdam, Amsterdam, Netherlands; 796 Hospital de Santo António, Oporto Hospital Center, Porto, Portugal; 797 Erasmus MC, Rotterdam, Netherlands; 798 Faculty of Psychology and Educational Sciences, Heerlen, Netherlands; 799 University of Toronto at Scarborough, Scarborough, ON Canada; 800 Hamad medical corporation, Doha, Qatar; 801 University of Szeged, Szeged, Hungary; 802 Jahn Ferenc Hospital, Budapest, Hungary; 803 University of Pécs, Pécs, Hungary; 804 Jessa Ziekenhuis, Hasselt, Belgium; 805 Hospital Clinico Universidad de Chile, Santiago, Chile; 806 Royal Marsden Hospital, London, UK; 807 Sheffield Teaching Hospitals, Sheffield, UK; 808 Tan Tock Seng hospital, Singapore, Singapore; 809 Centre Hospitalier de Lens, Lens, France; 810 Hospital Sao Rafael, Salvador, Brazil; 811 Hospital das Clinicas, Sao Paulo, Brazil; 812 Ntra Sra de Candelaria University Hospital, Santa Cruz de Tenerife, Spain; 813 University of Texas at Austin, San Antonio, USA; 814 Beaumont Hospital, Dublin, Ireland; 815 St Helens and Knowsley teaching hospitals, Liverpool, UK; 816 Nuovo Ospedale Civile Sant’Agostino Estense, Modena, Italy; 817 PICU, Hippokration General Hospital, Thessaloniki, Greece; 818 Maastricht University, Maastricht, Netherlands; 819 Maastricht University Medical Center, Maastricht, Netherlands; 820 University Hospital Antwerp, Edegem, Belgium; 821 Academic Medical Center, Amsterdam, Netherlands; 822 St Helens and Knoowsley, Prescot, UK; 823 University of Calgary, Calgary, Canada; 824 Vrije Universiteit Brussel, Brussel, Belgium; 825 EMGO+/VU University medical center, Utrecht, Netherlands; 826 Universitair Ziekenhuis Brussel, Brussel, Belgium; 827 University Hospital Lewisham, London, UK; 828 Queen Elizabeth Hospital, London, UK; 829 Lewisham & Greenwich NHS Trust, London, UK; 830 University of Glasgow, Glasgow, UK; 831 NHS Greater Glasgow and Clyde, Glasgow, UK; 832 National Burn Unit, Montevideo, Uruguay; 833 Hospital Maciel, Montevideo, Uruguay; 834 St Elisabeth Ziekenhuis, Tilburg, Netherlands; 835 Fondazione IRCCS Ca’ Granda - Ospedale maggiore Policlinico, Milan, Italy; 836 Istituto per lo Studio e la Prevenzione Oncologica, Florence, Italy

Unfortunately, the original version of this supplement [1] contained errors in two of the abstracts; P037 and P127. Please see details below.

In P037, the image presented as Figure eight is incorrect. The correct figure is shown below (Fig. 1).

**Fig. 1:**
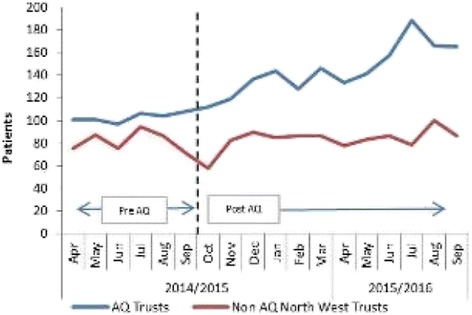
Mean number of patients per trust/month with an ICD-10 coding of sepsis

In P127, the author list presented is incorrect. The correct author list is as below.

R Iqbal^1^; Y Alhamdi^2^; N Venugopal^1^; S Abrams^2^; C Downey^3^; CH Toh^2^; ID Welters^1^

^1^ Institute of Ageing and Chronic Disease, Liverpool, UK;

^2^ Institute of Infection and Global Health, Liverpool, UK;

^3^ Department of Haematology, Royal Liverpool University Hospital (RLUH), Liverpool, UK
